# The EXIT Strategy: an Approach for Identifying Bacterial Proteins Exported during Host Infection

**DOI:** 10.1128/mBio.00333-17

**Published:** 2017-04-25

**Authors:** E. F. Perkowski, K. E. Zulauf, D. Weerakoon, J. D. Hayden, T. R. Ioerger, D. Oreper, S. M. Gomez, J. C. Sacchettini, M. Braunstein

**Affiliations:** a Department of Microbiology and Immunology, University of North Carolina—Chapel Hill, Chapel Hill, North Carolina, USA; b Department of Computer Science and Engineering, Texas A&M University, College Station, Texas, USA; c Joint Department of Biomedical Engineering at UNC—Chapel Hill and NC State University, Chapel Hill, North Carolina, USA; d Department of Biochemistry and Biophysics, Texas A&M University, College Station, Texas, USA; University of Michigan-Ann Arbor

**Keywords:** beta-lactamase reporter, EXIT, *Mycobacterium tuberculosis*, *in vivo*, membrane proteins, protein export, protein secretion, virulence

## Abstract

Exported proteins of bacterial pathogens function both in essential physiological processes and in virulence. Past efforts to identify exported proteins were limited by the use of bacteria growing under laboratory (*in vitro*) conditions. Thus, exported proteins that are exported only or preferentially in the context of infection may be overlooked. To solve this problem, we developed a genome-wide method, named EXIT (exported *in vivo* technology), to identify proteins that are exported by bacteria during infection and applied it to *Mycobacterium tuberculosis* during murine infection. Our studies validate the power of EXIT to identify proteins exported during infection on an unprecedented scale (593 proteins) and to reveal *in vivo* induced exported proteins (i.e., proteins exported significantly more during *in vivo* infection than *in vitro*). Our EXIT data also provide an unmatched resource for mapping the topology of *M. tuberculosis* membrane proteins. As a new approach for identifying exported proteins, EXIT has potential applicability to other pathogens and experimental conditions.

## IMPORTANCE

There is long-standing interest in identifying exported proteins of bacteria as they play critical roles in physiology and virulence and are commonly immunogenic antigens and targets of antibiotics. While significant effort has been made to identify the bacterial proteins that are exported beyond the cytoplasm to the membrane, cell wall, or host environment, current methods to identify exported proteins are limited by their use of bacteria growing under laboratory (*in vitro*) conditions. Because *in vitro* conditions do not mimic the complexity of the host environment, critical exported proteins that are preferentially exported in the context of infection may be overlooked. We developed a novel method to identify proteins that are exported by bacteria during host infection and applied it to identify *Mycobacterium tuberculosis* proteins exported in a mouse model of tuberculosis.

## INTRODUCTION

The bacterial exportome is the subset of proteins that are exported beyond the cytoplasm to the cytoplasmic membrane or the cell wall (CW) or are released (secreted) into the environment. There is long-standing interest in identifying exported proteins of bacteria as they play critical roles in physiology and virulence and are commonly immunogenic antigens and targets of antibiotics (1, 2). However, current approaches to identify exported proteins have limitations. Bioinformatic predictions of exported proteins are complicated by disagreement between prediction algorithms, which makes experimental validation critical. Mass spectrometry (MS)-based proteomics suffers from the intrinsic difficulty of isolating pure subcellular fractions, which can result in identification of contaminating proteins as false positives (3, 4). Genetic reporters (e.g., PhoA) of export nearly always require phenotypic screening of in-frame fusion proteins on a colony-by-colony basis, which limits the number of proteins identified, even in the most ambitious efforts (5–7). A further significant limitation of current methods is their use of bacteria grown in laboratory media (*in vitro*), which fails to recapitulate the complexity of the host environment (4). Thus, proteins that are preferentially or exclusively exported during infection are overlooked (8). The significance of studying pathogens in the context of the host is borne out by methods such as IVET (*in vivo* expression technology), STM (signature tagged mutagenesis), and TraSH (transposon site hybridization), which reveal virulence mechanisms overlooked by *in vitro*-based studies (9). Here, we report a novel genome-wide method that we refer to as EXIT (exported *in vivo* technology) that identifies proteins exported by a bacterial pathogen during *in vivo* infection.

**FIG 1:**
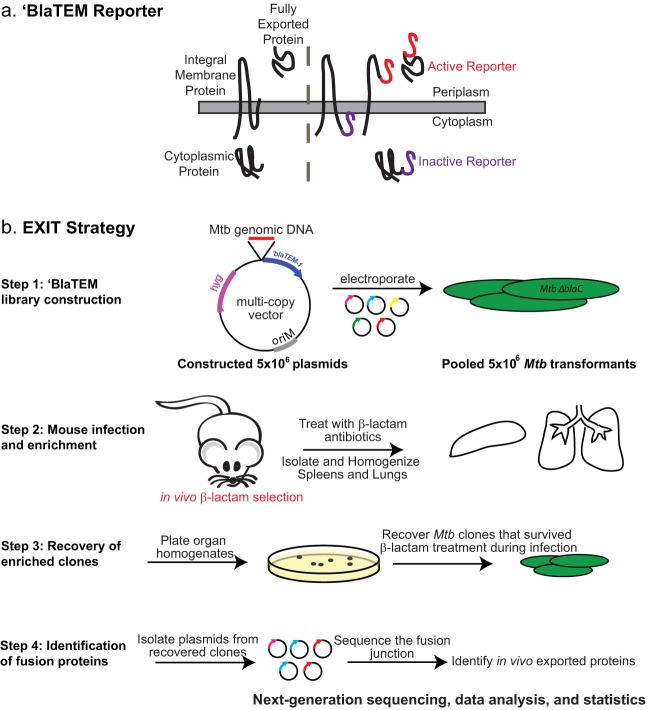
(a) The ‘BlaTEM reporter. The ‘BlaTEM reporter is compatible with proteins localized to the bacterial cytoplasmic membrane or cell wall or secreted from the bacterial cell. The right panel indicates in-frame fusions to categories of exported proteins that confer β-lactam resistance (red). In-frame fusions to cytoplasmic proteins or the cytoplasmic domain of integral membrane proteins (purple) do not confer β-lactam resistance. (b) EXIT strategy. In step 1, a comprehensive library of 5 × 10^6^ plasmids containing fragments of *M. tuberculosis* (Mtb) genomic DNA fused to the ‘*bla*_TEM_ reporter was constructed. The plasmid library was transformed into the *ΔblaC* β-lactamase-sensitive mutant of *M. tuberculosis*, and 5 × 10^6^ transformants were pooled to generate the EXIT library. In step 2, mice were infected by intravenous injection with the EXIT library and treated with β-lactam antibiotics (oral gavage twice daily) to select for EXIT clones exporting ‘BlaTEM fusion proteins. β-lactam treatment began 1 day after infection and continued to 2 weeks after infection. Mice were sacrificed, and spleens and lungs were harvested and homogenized. In step 3, organ homogenates were plated on 7H10 agar and grown to recover *M. tuberculosis* clones that survived β-lactam treatment during infection. Plates were scraped, and colonies were pooled separately for lungs and spleens. In step 4, plasmids from the recovered bacteria and the input samples were isolated and the fusion junction was sequenced using next-generation sequencing. Sequencing primers were designed to read out of the ‘*bla*_TEM_ reporter and sequence the immediately adjacent *M. tuberculosis* DNA. Sequences were aligned to the *M. tuberculosis* genome. Unique sequences were counted to identify the abundance of each fusion junction site within the population. The genes that were most highly abundant after *in vivo* β-lactam treatment were identified, and the results corresponded to plasmids producing in-frame exported ‘BlaTEM fusion proteins.

EXIT utilizes the ‘BlaTEM β-lactamase reporter of export (10). Because ‘BlaTEM lacks its native signal peptide for export, it is exported only to the extracytoplasmic space when fused in-frame to an export signal (i.e., signal peptide or transmembrane domain). When exported, ‘BlaTEM cleaves β-lactams and confers β-lactam resistance to bacteria (10). Importantly, ‘BlaTEM is a selectable reporter and bacteria exporting ‘BlaTEM can be collected by virtue of their ability to survive β-lactam treatment. ‘BlaTEM reporter fusions can identify cell wall and fully secreted proteins, as well as exported domains of integral membrane proteins (10, 11) (Fig. 1a).

Here, we used EXIT to identify ‘BlaTEM fusions to proteins that are exported by the pathogen *Mycobacterium tuberculosis* during infection of β-lactam-treated mice. By combining a comprehensive library of in-frame ‘BlaTEM fusions with the ability to select bacteria exporting fusion proteins *in vivo* and next-generation sequencing en masse of the recovered fusions, EXIT identified 593 proteins as exported by *M. tuberculosis* during infection. This list of EXIT proteins is significant in demonstrating *in vivo* export for 54% of the 1,040 *M. tuberculosis* open reading frames (ORFs) computationally predicted to be exported (see Materials and Methods). Moreover, for 100 proteins, EXIT provided the first experimental evidence for their export. EXIT also identified 32 proteins lacking *in silico* predicted export signals, which speaks to the unbiased nature of the approach. For the 337 integral membrane proteins identified, the sites of exported fusions are significant in providing protein topology information, which is notoriously difficult to predict computationally (12) but critical for membrane protein studies. Finally, 38 of the proteins identified were *in vivo* induced exported proteins (i.e., proteins exported significantly more during *in vivo* infection than *in vitro*). We showed that *M. tuberculosis* mutants defective in four of these proteins, all of unknown function, have intracellular growth defects in macrophages. Our studies validate the power of EXIT to identify proteins exported during infection, to reveal new virulence factors, and to provide valuable resources for functional studies of uncharacterized proteins.

## RESULTS

EXIT involves four steps (Fig. 1b; see Materials and Methods for details). In step 1, a comprehensive library of plasmids carrying random fragments of *M. tuberculosis* genomic DNA cloned in front of ‘*bla*_TEM_ was constructed. On average, the *M. tuberculosis* EXIT library contained a fusion junction every 26 bp in the genome and each gene was represented by 16 in-frame fusions. Because *M. tuberculosis* has an endogenous β-lactamase BlaC (13), the EXIT library was constructed in a *M. tuberculosis ΔblaC* mutant to enable selection for β-lactam-resistant fusions. In step 2, mice were infected with the pooled EXIT library and, starting 1 day after infection, treated with β-lactam antibiotics to select for *M. tuberculosis* exporting ‘BlaTEM fusion proteins *in vivo*. The efficacy of the β-lactam treatment in selecting strains expressing exported ‘BlaTEM fusions from a mixed population was initially confirmed in proof-of-principle experiments (see Fig. S1 in the supplemental material). After 2 weeks of treatment, mice were sacrificed, and spleens and lungs were harvested. In step 3, organ homogenates were plated on 7H10 agar to recover *M. tuberculosis* clones that survived β-lactam treatment during infection. In step 4, library plasmids were isolated from the bacteria that survived *in vivo* β-lactam treatment, as well as from the input library, and the fusion junctions were sequenced using next-generation sequencing. A pipeline was built to analyze the sequencing data, and the abundance of individual fusions was determined by read count. Using statistical modeling, highly abundant fusions recovered from the mice following *in vivo* β-lactam treatment were identified.

10.1128/mBio.00333-17.2 FIG S1  Proof of principle: the ‘BlaTEM reporter functions in β-lactam-treated mice. (A) Mice were infected with Δ*blaC M. tuberculosis* strains producing a ‘BlaTEM reporter fused in frame with a Sec signal peptide from the secreted Mpt63 protein (sp.-‘BlaTEM, red) or producing nonexported ‘BlaTEM reporter alone (‘BlaTEM, black). One group of mice infected with each strain was sacrificed to determine the initial bacterial burden in the spleens on day 1 after infection, and groups of mice (4 mice per group) were followed to day 14 after infection. Half of the mice were treated with the β-lactam antibiotic amoxicillin and with a synergistic drug, probenecid, twice daily by oral gavage, while half remained untreated. On day 14, mice were sacrificed and spleens were homogenized and plated on agar media to determine bacterial burden (CFU) with and without treatment. *, statistically significant (*P* < 0.05). (B) Mice were infected with a mix of Δ*blaC M. tuberculosis* strains producing the nonexported ‘BlaTEM reporter or the exported spMpt63-‘BlaTEM reporter in a 99:1 ratio (‘BlaTEM: sp.-‘BlaTEM). The input inoculum was plated in parallel on 7H10 agar with or without β-lactam to determine the starting β-lactam resistance frequency. Half of the mice were treated with the β-lactam antibiotic amoxicillin and a synergistic drug (probenecid) twice daily by oral gavage, while half remained untreated. On day 14, mice were sacrificed and spleens were homogenized and plated again in parallel on 7H10 agar and 7H10 agar containing β-lactam antibiotics to determine the percentage of β-lactam resistance following *in vivo* treatment or no treatment. Download FIG S1, EPS file, 0.8 MB. Copyright © 2017 Perkowski et al. 2017 Perkowski et al. This content is distributed under the terms of the Creative Commons Attribution 4.0 International license.

### EXIT in *M. tuberculosis*-infected mice.

**FIG 2:**
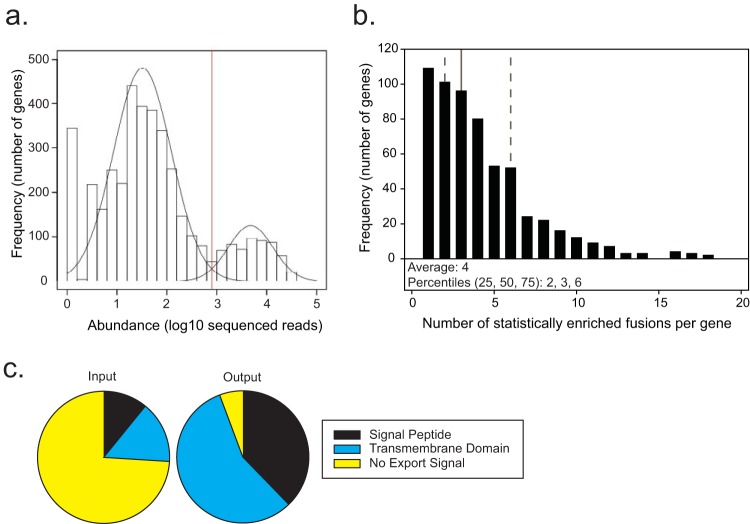
EXIT identified 593 proteins as exported during murine infection. (a) The most abundant fusion site within each annotated gene in the *M. tuberculosis* genome was identified individually within the output for each of two replicate experiments. The lower of these two numbers was plotted on a histogram. A two-component Gaussian mixture model (black line overlay) was used to generate a statistical model distinguishing between high-abundance genes (right) and low-abundance genes (left), with a statistical cutoff of log10 = 2.90 (red line). A total of 593 genes were identified in the high-abundance population corresponding to EXIT exported proteins. (b) Genes identified as encoding exported proteins were analyzed for the number of statistically enriched unique fusion sites after *in vivo* β-lactam treatment. On average, 4 unique fusion sites were enriched for each exported protein. Percentiles are shown with dotted lines representing the 25th and 75th percentiles and a solid line representing the 50th percentile. (c) The input EXIT library was composed of fusions in 99% of *M. tuberculosis* genes, with 74% encoding proteins with no predicted export signal (yellow), 15% encoding predicted integral membrane proteins (blue), and 11% encoding proteins containing predicted signal peptides (black). In contrast, 95% of the proteins in the EXIT output contained an export signal. The 593 proteins identified as exported in EXIT were composed of 57% predicted integral membrane proteins (blue), 38% proteins containing a predicted signal peptide (black), and 5% proteins with no predicted export signal (yellow). By analysis of all ORFs of *M. tuberculosis* H37Rv for *in silico* predicted export signals (see Materials and Methods), 26% (1,040 proteins) of the *M. tuberculosis* proteome were predicted to be exported. This compares well to predictions of exported proteins in other bacteria, which usually predict 20% to 30% of the proteome to be exported (77).

EXIT experiments were performed in duplicate on two independent occasions, with the results from each experiment being highly correlated (Fig. S2A to C). As done before in genome-wide screens of *M. tuberculosis in vivo* (14–16), in order to achieve maximal library representation we infected mice with the EXIT library using intravenous (i.v.) injection (~10^6^ CFU), which resulted in higher seeding of spleens versus lungs. Unless noted otherwise, the results described are from the more comprehensive spleen data set. On the basis of proof-of-principle experiments (Fig. S1), *M. tuberculosis* clones expressing in-frame ‘BlaTEM fusions to ORFs of exported proteins were expected to survive and replicate during *in vivo* β-lactam treatment and to be more abundant (assessed by sequenced read count) than strains not exporting the reporter in the output from treated mice. A Gaussian mixture model was constructed to describe the data as two populations of low-abundance and high-abundance genes (Fig. 2a). Using this statistical model, 593 genes were identified as highly abundant (in both of the replicate experiments) in the recovered population after *in vivo* β-lactam treatment and were thus predicted to encode exported proteins (see Table S1 in the supplemental material). For 82% of these 593 proteins, multiple unique fusion sites were enriched after passage through β-lactam-treated mice, providing confidence in the list of proteins identified as exported *in vivo* (Fig. 2b; Table S1). Note that there is no promoter sequence upstream of the reporter on the EXIT plasmid backbone (pDW31); therefore, an active ‘BlaTEM fusion requires in-frame fusion to a gene encoding an exported protein that is expressed from its native promoter.

10.1128/mBio.00333-17.3 FIG S2  Reproducibility and correlation between EXIT replicate experiments and correlation between abundances of EXIT protein fusions recovered from 7H10 agar with and without β-lactam antibiotics. (A) Raw read count values in the input used for each replicate experiment (A and B) were plotted for each fusion junction site on a log_2_ scale. A Pearson product moment correlation identified a significant correlation *r* value of 0.818. (B) Raw read count values from colonies recovered from the spleens of treated mice for each replicate experiment (A and B) were plotted for each fusion junction site on a log_2_ scale. A Pearson product moment correlation identified a significant correlation *r* value of 0.784. (C) Raw read count values from colonies recovered from the spleens of treated mice after plating was performed on agar media containing β-lactam (selection *in vivo* and *in vitro*) for each replicate experiment (A and B) were plotted for each fusion junction site on a log_2_ scale. Pearson product moment correlation identified a significant correlation *r* value of 0.858. (D) Raw read count values for each fusion junction site were compared between colonies recovered from the spleens of treated mice after plating on 7H10 agar lacking β-lactam (*in vivo* selection only) or after plating on 7H10 agar containing β-lactam (*in vivo* and *in vitro* selection). This high degree of correlation (Pearson product moment correlation *r* value of 0.857) demonstrated significant similarity for the majority of proteins between export during infection and export during *in vitro* growth. Download FIG S2, TIF file, 0.4 MB. Copyright © 2017 Perkowski et al. 2017 Perkowski et al. This content is distributed under the terms of the Creative Commons Attribution 4.0 International license.

10.1128/mBio.00333-17.5 TABLE S1  EXIT results: all 593 *in vivo* exported proteins. Download TABLE S1, DOCX file, 0.1 MB. Copyright © 2017 Perkowski et al. 2017 Perkowski et al. This content is distributed under the terms of the Creative Commons Attribution 4.0 International license.

### Validation of EXIT-identified proteins.

We assessed the accuracy of EXIT to select for *in vivo* exported proteins by searching for *in silico* predicted export signals (signal peptides and transmembrane domains) in the proteins identified (Fig. 2c). A total of 95% of the 593 proteins had export signals compared to only 26% of in-frame fusions in the input library. EXIT proteins with predicted Sec signal peptides, Tat signal peptides, lipoprotein signal peptides, and transmembrane domains were identified (Table S1). We also compared the proteins in the EXIT list to proteins previously demonstrated to be exported by *in vitro*-grown bacteria using MS-based subcellular proteomics or genetic reporters of export (Table S1). A total of 83% of EXIT proteins were previously identified as exported, providing further validation. For the remaining 17% (100 proteins), the identification by EXIT is significant in providing the first experimental evidence of their export.

### EXIT proteins lacking conventional signals for export.

**FIG 3:**
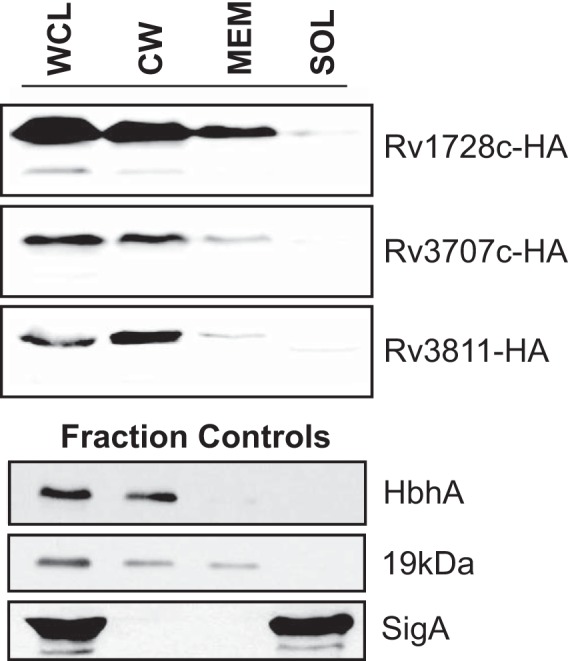
Validation of EXIT-identified exported proteins with no *in silico* predicted export signal. Three proteins with no *in silico* predicted export signal (Rv1728c, Rv3707c, and Rv3811) were engineered with C-terminal HA tags and expressed from the constitutive *hsp60* promoter in *M. tuberculosis*. Cells were irradiated, lysed by the use of a French pressure cell into whole-cell lysate (WCL), equalized by bicinchoninic acid (BCA) protein quantification, and fractionated by differential ultracentrifugation into cell wall (CW), membrane (MEM), and soluble/cytoplasmic (SOL) fractions. Fractions derived from equivalent amounts of starting cellular material were separated by SDS-PAGE, and HA-tagged proteins were detected by immunoblotting performed with anti-HA antibodies. The cell wall protein (HbhA), membrane protein (19-kDa lipoprotein), and cytoplasmic protein (SigA) were included as fractionation controls.

A small number of EXIT proteins (32 proteins) lack predicted signal peptides or transmembrane domains. These proteins are candidates for being nonconventional exported proteins or for being overlooked by the *in silico* algorithms used (see Materials and Methods) (Fig. 2c) (Table S2). To validate proteins on this list of unpredicted exported proteins, we used the *hsp60* promoter to constitutively express three of these proteins (Rv1728c, Rv3707c, and Rv3811) with a C-terminal hemagglutinin (HA) tag in *M. tuberculosis*. Subcellular fractions (cell wall, membrane, and soluble cytoplasm) prepared from these strains were then used to localize these proteins by immunoblotting. All three proteins were exported to the cell wall (CW) of *M. tuberculosis* (Fig. 3). These results confirm the ability of EXIT to identify exported proteins that are missed by heavily relied upon *in silico* prediction tools.

10.1128/mBio.00333-17.6 TABLE S2  EXIT proteins lacking *in silico* predicted export signals. Download TABLE S2, DOCX file, 0.1 MB. Copyright © 2017 Perkowski et al. 2017 Perkowski et al. This content is distributed under the terms of the Creative Commons Attribution 4.0 International license.

### EXIT fusions in the lungs.

As a consequence of low seeding of *M. tuberculosis* to the lungs following i.v. injection (17), we were unable to develop a formal statistical model to analyze the lung data. However, using a threshold cutoff of 3.5-fold enrichment of a gene in the lungs in both experimental replicates versus the input abundance (a threshold which agreed with the statistically defined threshold determined in the spleens), we identified 282 proteins as strong candidates for being exported in the lungs (Table S1). Of these, 274 (97%) were also on the list of 593 EXIT proteins exported in the spleen (Table S1). We predict that bottleneck effects prevented us from identifying a higher proportion of the 593 proteins as being exported in the lungs.

Eight proteins predicted to be exported in the lung, but not identified by EXIT as exported in the spleen, represent a potentially interesting group of proteins that may be regulated by the lung environment in either expression or export (Table S3). Four of these proteins are PE_PGRS proteins, a poorly understood class of repeat-containing proteins unique to mycobacteria (18). One of these proteins is PE_PGRS33, which contributes to *M. tuberculosis* entry into macrophages (19) and may additionally modulate the host cytokine response (18, 20). However, further studies will be required to confirm that these eight proteins are lung specific.

10.1128/mBio.00333-17.7 TABLE S3  EXIT exported proteins only identified in the lungs. Download TABLE S3, DOCX file, 0.01 MB. Copyright © 2017 Perkowski et al. 2017 Perkowski et al. This content is distributed under the terms of the Creative Commons Attribution 4.0 International license.

### EXIT-exported fusions provide topology information for membrane proteins.

**FIG 4:**
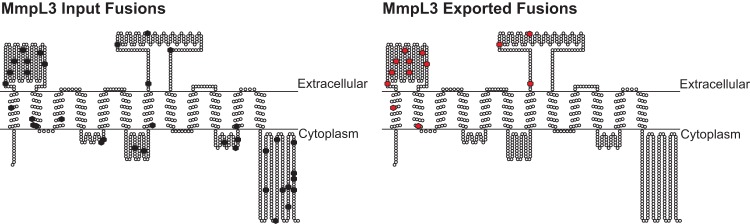
MmpL3 topology mapping using EXIT fusion site data. A total of 37 unique fusion sites in MmpL3 were represented in the input library (black hexagons). Of these, 13 fusion sites were enriched during β-lactam treatment of mice, indicating an extracytoplasmic location (red hexagons) corresponding to two large exported domains of the MmpL3 protein. Exported fusion sites were mapped onto the *in silico* topology prediction generated by TopPred (21).

Because ‘BlaTEM must be positioned in the extracytoplasmic space to produce β-lactam resistance, the behavior of individual EXIT fusions provides topological information. In the 593 EXIT proteins, there were 2,516 fusion sites that were enriched during β-lactam treatment (from a total of 10,711 in-frame fusions for these proteins in the input) (Table S1; Table S4). To validate the use of EXIT for topology mapping, we investigated fusion sites in the MmpL3 transporter protein. All 13 of the MmpL3 EXIT fusions enriched during β-lactam treatment *in vivo* mapped to two large domains, indicating an extracytoplasmic location, while other fusions in the input library, including many that mapped to the C-terminus, were not enriched (Table S1; Table S4). These data align with the TopPred (21) prediction of 12 transmembrane helices with two large extracytoplasmic domains and a cytoplasmic C-terminus for MmpL3 (Fig. 4), and they agree with results of recent MmpL3 structure and topology studies (22). Given that multiple topology models have been published for MmpL3 (23–32) (Fig. S3), this analysis is significant in demonstrating the ability of EXIT to distinguish between discordant models. Among 10 other MmpL proteins identified, there were 52 enriched EXIT fusion sites that mapped similarly to two large domains, suggesting that these extracytoplasmic domains are a conserved feature of MmpL transporters (Table S1; Table S4).

10.1128/mBio.00333-17.4 FIG S3  Topology models of membrane protein MmpL3 and Rv1002c. (A) Topology predictions for MmpL3 provided by TopPred (see reference 1 in Fig. S3), TMHMM (see reference 2 in Fig. S3), TMpred (see reference 3 in Fig. S3), and Memsat (see reference 4 in Fig. S3). The topology models disagreed on the number of transmembrane domains and the orientation of two of the larger domains and the C-terminal domain. All four of these MmpL3 topology predictions were previously published (see references 5 to 12 in Fig. S3). (B) Topology models for Rv1002c provided by HMMTOP (see reference 13 in Fig. S3), TopPred (see reference 1 in Fig. S3), TMHMM (see reference 2 in Fig. S3), TMpred (see reference 3 in Fig. S3), and Memsat (see reference 4 in Fig. S3). The topology models disagreed on the number of transmembrane domains, the orientation of the intervening domains, and the location of the N and C-termini. (C) A total of 22 unique fusion sites in Rv1002c were represented in the input library (black hexagons). Of these, 5 fusion sites were identified as exported in EXIT (red hexagons), corresponding to the first loop, largest loop, and the C-terminal domain as exported. Exported fusion sites were mapped onto the *in silico* topology predictions generated by HMMTOP (see reference 13 in Fig. S3). Download FIG S3, PDF file, 0.5 MB. Copyright © 2017 Perkowski et al. 2017 Perkowski et al. This content is distributed under the terms of the Creative Commons Attribution 4.0 International license.

10.1128/mBio.00333-17.8 TABLE S4  EXIT input library fusions (all *M. tuberculosis* proteins). Download TABLE S4, DOCX file, 0.3 MB. Copyright © 2017 Perkowski et al. 2017 Perkowski et al. This content is distributed under the terms of the Creative Commons Attribution 4.0 International license.

### Identification of *in vivo* induced exported proteins.

**FIG 5:**
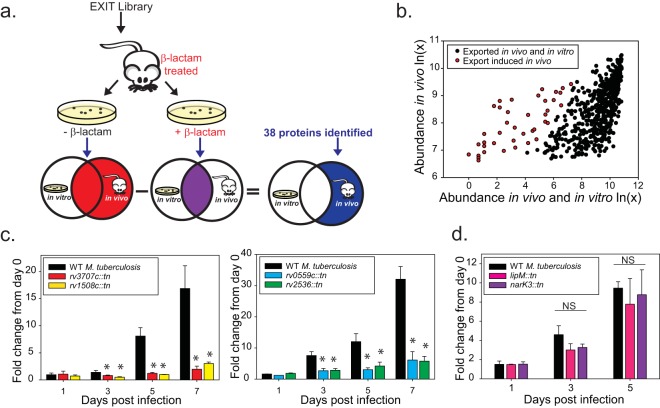
Strategy for identification of *in vivo* induced exported proteins. (a) Identification of *in vivo* induced exported proteins. Spleens from β-lactam-treated mice infected with the EXIT library were harvested after 2 weeks of infection. Spleen homogenates were plated in parallel on 7H10 agar without β-lactam to recover all clones (red Venn diagram) and on 7H10 agar containing β-lactam to recover clones exporting ‘BlaTEM fusion proteins during *in vivo* growth and *in vitro* growth (purple Venn diagram). The population of clones identified only or in significantly greater abundance on media lacking β-lactams represents proteins whose export was induced during infection (blue). (b) Sequenced read count values recovered from agar with or without β-lactam for the 593 EXIT proteins were plotted to compare abundances after β-lactam treatment *in vivo*, with the abundance after dual β-lactam treatment *in vivo* and *in vitro* indicated. The majority of proteins identified as exported *in vivo* remained highly abundant after additional β-lactam treatment *in vitro* (black). A total of 38 genes (highlighted in red) were identified as statistically less abundant after *in vitro* β-lactam selection, representing proteins exported significantly more *in vivo* than *in vitro* (see Materials and Methods for details on statistical analysis). (c) *In vivo* induced exported proteins with roles promoting growth in macrophages (*rv1508*::*tn*, *rv3707c:tn*, *rv0559c*::*tn*, and *rv2536*::*tn*). Murine bone marrow-derived macrophages were infected with *M. tuberculosis* CDC1551 transposon mutants lacking individual *in vivo* induced exported proteins. At specific times postinfection, macrophage lysates were plated to measure intracellular CFU. The fold change in CFU over the course of the infection is plotted relative to the bacterial burden at day 0 postinfection. Statistical significance was determined by one-way analysis of variance (ANOVA) with multiple comparisons performed by the use of the Holm-Sidak (normal by Shapiro-Wilk) or Student-Newman-Keuls (nonnormal) test (*, *P* < 0.05 [compared to wild-type {WT} CDC1551]). These data are representative of results of four independent experiments, each performed with triplicate wells of infected macrophages. (d) NarK3 and LipM [*lipM*::*tn* (*rv2284*::*tn*) and *narK3*::*tn* (*rv0261c*::*tn*)] mutants did not exhibit intracellular growth defects in macrophages. NS, not significant.

EXIT provides an opportunity to identify *M. tuberculosis* proteins that are exported more during *in vivo* infection than during *in vitro* growth. Such proteins, which we refer to as *in vivo* induced exported proteins, could result from transcriptional/posttranscriptional induction *in vivo* or from *in vivo* upregulation of the responsible protein export system. In either case, the *in vivo* regulation is suggestive of important functions during infection. To identify *in vivo* induced exported proteins, the EXIT bacteria surviving β-lactam treatment in mice were plated in parallel on 7H10 agar and 7H10 agar containing β-lactam (Fig. 5a). The clones recovered on regular agar represent the fusion proteins exported during infection (i.e., the 593 *in vivo* exported proteins discussed above). Clones recovered on β-lactam agar express fusions that are additionally expressed and exported under *in vitro* conditions. There was high correlation in the abundances of individual EXIT fusions recovered from 7H10 agar with and without β-lactam, indicating that the majority of EXIT proteins were exported similarly *in vivo* and under these *in vitro* conditions (Fig. S2D). To identify proteins that are exported significantly more *in vivo* than *in vitro*, genes with significantly lower recovery from β-lactam agar (*in vitro* plus *in vivo*) versus regular agar (*in vivo*) were identified, a multiple-comparison correction was applied, and the false-discovery rate (FDR) was set at 5% (see Materials and Methods). In this way, 38 of the 593 EXIT proteins were identified as *in vivo* induced exported proteins (Table 1) (Fig. 5b). Of the 38 *in vivo* induced exported proteins, 14 were previously shown to be transcriptionally upregulated during infection, which helps validate this approach (Table 1). Proteins with functions in regulation (SenX3, PknH), host defense (MmcO, Rv3654c), and cell wall lipid transport (DrrC, MmpL8) were among those identified. However, the largest category of *in vivo* induced exported proteins (19 of 38 proteins) consisted of proteins of unknown function. Another notable category of *in vivo* induced exported proteins consists of proteins lacking *in silico* predicted export signals. Eight of the 38 *in vivo* induced exported proteins identified by EXIT lack predicted export signals, including Rv3707c, which we confirmed to have been exported to the cell wall (Fig. 3).

**TABLE 1:** *In vivo* induced exported proteins identified by EXIT

ORF no.	Gene name(s)	Predicted or proven function	*In* *silico* export signal^a^	Predicted or proven essential *in vitro* or during infection	Transcriptional upregulation *in vivo*	*q* value
Rv0011c		Cell division	TM			0.000
Rv0261c	*narK3*	Nitrite extrusion protein	TM			0.003
Rv0490	*senX3*	Two-component sensor histidine kinase	TM	In mice (14, 78, 79)		0.028
Rv0506	*mmpS2*	Unknown	SP, TM			0.007
Rv0559c		Unknown	SP			0.008
Rv0593	*lprL*, *mce2E*	Mce family lipoprotein	Lipo, TM	In mice (80)		0.006
Rv0615		Unknown	TM			0.017
Rv0713		Unknown	TM			0.001
Rv0817c		Unknown	SP, TM	*In vitro* (81)		0.005
Rv0846c	*mmcO*	Multicopper oxidase	SP, Tat SP, Lipo			0.000
Rv0892		Probable monooxygenase	TM		In macrophages (82)	0.008
Rv1026	*ppx-2*	Polyphosphatase		*In vitro* (81), in mice (83)		0.022
Rv1145	*mmpL13a*	Unknown	TM			0.000
Rv1266c	*pknH*	Serine/threonine-protein kinase	TM	In mice (84)		0.029
Rv1508c		Unknown	TM		In mice (85)	0.007
Rv1517		Unknown	Tat SP, TM			0.015
Rv1639c		Unknown	TM			0.007
Rv1737c	*narK2*	Possible nitrate-nitrite transporter	TM	In mice (15)	In macrophages (82)	0.000
Rv1739c		Sulfate transporter	TM		In macrophages (82)	0.043
Rv1965	*yrbE3B*	Permease component of Mce system	TM	In macaques (86), in mice (87, 88)	In macrophages (82)	0.001
Rv1969	*mce3D*	Mce family protein	SP, TM	In mice (87, 88)		0.006
Rv2138	*lppL*	Probable conserved lipoprotein	SP, Lipo, TM	In mice (15), *in vitro* (81)		0.006
Rv2144c		Unknown	TM		In mice (85)	0.001
Rv2273		Unknown	TM		In macrophages (89)	0.000
Rv2284	*lipM*	Probable esterase	TM		In mice (90)	0.024
Rv2330c	*lppP*	Probable lipoprotein	SP, Lipo, TM	In macrophages (91)	In macrophages (82)	0.007
Rv2380c	*mbtE*	Mycobactin synthesis		In mice (15), *in vitro* (81)	In macrophages (82)	0.007
Rv2536		Unknown	TM	In mice (15)		0.017
Rv2938	*drrC*	Phthiocerol dimycocerosate transport	TM	In mice (15)		0.017
Rv3343c	*PPE54*	PPE family protein		*In vitro* (81)	In humans (92)	0.006
Rv3478	*PPE60*, *mtb39c*	PE family protein				0.017
Rv3526	*kshA*	Oxygenase component of 3-ketosteroid-9-alpha-hydroxylase		In mice (93)	In macrophages (82)	0.005
Rv3554	*fdxB*	Possible electron transfer	TM			0.001
Rv3596c	*clpC1*	ATP-dependent protease ATP-binding subunit		In macrophages (57, 91), *in vitro* (81)		0.006
Rv3654c		Unknown		In macrophages (34)	In humans (92)	0.000
Rv3701c		Ergothioneine biosynthesis		In macrophages (91), in mice (14)		0.039
Rv3707c		Unknown				0.039
Rv3823c	*mmpL8*	Sulfolipid-1 (SL-1) transporter	TM	In mice (15), *in vitro* (81)		0.027

a Lipo, lipoprotein signal peptide; SP, Sec signal peptide; Tat, Tat signal peptide; TM, transmembrane domain.

### In vivo induced exported proteins contribute to *M. tuberculosis* virulence.

Given the precedent for upregulation of virulence factors in the host (8), we predicted that the list of *in vivo* induced exported proteins would include proteins with roles in pathogenesis. In fact, 13 of the exported proteins on the list of those induced *in vivo* have demonstrated or predicted roles (based on TraSH/transposon sequencing [Tnseq] studies) in virulence (Table 1). To explore this possibility further, we obtained six *M. tuberculosis* mutants with transposon insertions in genes encoding *in vivo* exported proteins from the Biodefense and Emerging Infections Research Resources Repository (BEI Resources) (33) and tested them for intracellular growth in murine bone marrow macrophages. Intracellular growth of each mutant was compared to that of the parental *M. tuberculosis* CDC1551 strain by plating bacilli from macrophage lysates over time. Mutants carrying transposon insertions in *lipM* and *narK3* had no intracellular growth defect in macrophages (Fig. 5d). However, four mutants carrying transposon insertions in genes encoding *in vivo* induced exported proteins of unknown function (*rv3707c*, *rv1508c*, *rv0559c*, and *rv2536*) demonstrated significant defects in intracellular growth compared to the parental strain (Fig. 5c). None of these mutants exhibited a general growth defect during growth in culture (*in vitro*) (data not shown). This mutant analysis demonstrates how the functional genomics information provided by EXIT can be harnessed to reveal uncharacterized virulence factors.

## DISCUSSION

EXIT is a method for discovering bacterial proteins exported during *in vivo* infection. In applying this approach to *M. tuberculosis*, we identified an unprecedented total of 593 *in vivo* exported proteins and additionally identified *in vivo* induced exported proteins that include uncharacterized virulence factors. Moreover, the total number of EXIT proteins identified surpassed the number of exported proteins identified in past discovery efforts using genetic reporters with *in vitro*-grown bacteria (5–7, 11). EXIT increased the number of experimentally demonstrated *M. tuberculosis* exported proteins by 100, including examples lacking *in silico* predicted export signals, and it provided a database of enriched fusion sites for mapping protein topology. The broad effectiveness of EXIT can be attributed to the following factors: (i) the highly comprehensive library (99% of the genome represented with at least one in-frame fusion); (ii) the use of the ‘BlaTEM reporter as a selectable marker *in vivo*; and (iii) the use of next-generation sequencing and statistical analysis to identify exported fusions.

EXIT identified 32 proteins that lack export signals, with 8 being *in vivo* induced exported proteins. Although it remains possible that some of the EXIT protein identifications represent false positives, our validation of three of these proteins as exported (Fig. 3) argues for other proteins on this list being true exported proteins. EXIT identification of proteins lacking standard export signals may reflect the limitations of *in silico* algorithms or reflect the fact that these proteins are exported by unconventional pathways. For example, the *in vivo* induced exported Rv3654c protein lacks an obvious export signal but was previously suggested to be secreted during infection, on the basis of detection of Rv3654c in macrophage lysates (34). Our EXIT results provide important confirmation of Rv3654c being exported *in vivo*. Further, the *rv3654c* gene is located near genes for potential tight adherence (Tad) secretion system components (34), which could be responsible for Rv3654c export.

EXIT identified all types of exported proteins: cytoplasmic membrane proteins (e.g., MmpL3 [22], OmamA [35]), cell wall proteins (e.g., FbpA [36, 37], HbhA [38]), mycobacterial outer membrane proteins (e.g., OmpA [39], SpmT [40]), and fully secreted/extracellular proteins (SapM [41], Mpt63 [42]) (see Table S1 in the supplemental material). However, the small secreted ESAT-6/CFP-10-like proteins that are secreted by specialized ESX/type VII secretion systems (43), and SodA and PknG, which require the SecA2-dependent system for export, were not identified by EXIT (44–46), despite the presence of in-frame fusions in the input library. For any genetic reporter of export, some proteins may be missed due to incompatibility with specialized export systems; for example, ESAT-6/CFP-10 proteins are secreted as a dimer (43) and one possibility is that ‘BlaTEM fusions could disrupt ESAT-6/CFP-10 interactions. In addition, proteins may be missed due to the level of expression required for a positive export signal (β-lactam resistance), toxicity, or instability of certain fusion proteins. One of these factors is the likely explanation for the fact that no ESAT-6/CFP-10, SodA, or PknG proteins were identified by the ‘BlaTEM reporter in EXIT or in our past studies (11). Note that a study reporting the use of the ‘BlaTEM reporter with ESAT-6/CFP-10 secreted proteins was retracted (47, 48). However, EXIT was successful in identifying other examples of SecA2-dependent proteins (solute binding proteins and Mce proteins [45]), and it identified 10 PE, PPE, and PE_PGRS proteins representing another protein family exported by ESX/type VII secretion systems (49, 50). The YxxxD/E motif that exists in proteins exported by ESX/type VII secretion systems is present in 6 of the 10 EXIT-identified PE, PPE, and PE_PGRS proteins, although some of these proteins additionally have *in silico* predicted Sec signal peptides (Table S1; Table S3), which makes their route of export more difficult to predict.

As an unbiased genome-wide approach, EXIT has the potential to reveal unannotated/misannotated proteins. Along these lines, EXIT identified multiple enriched fusions in the same reading frame in six unannotated intragenic regions of the genome. We hypothesize that these fusions map to unannotated ORFs (Table S5). For example, a candidate unannotated ORF with a Sec signal peptide is in the region between Rv2304c and Rv2305 (labeled as downstream of Rv2307c). Future studies are warranted to confirm the existence of these putative proteins.

10.1128/mBio.00333-17.9 TABLE S5  EXIT exported fusions in unannotated regions. Download TABLE S5, DOCX file, 0.01 MB. Copyright © 2017 Perkowski et al. 2017 Perkowski et al. This content is distributed under the terms of the Creative Commons Attribution 4.0 International license.

Although protein topology is critical information for understanding membrane protein function, limited experimental topology data exist on a genome-wide level and the *in silico* prediction algorithms used to design experiments often disagree (12). EXIT proved valuable in discriminating between topology predictions for MmpL3, a protein of interest for its essentiality in *M. tuberculosis* and its association with resistance mutations to several TB drug candidates (25–27, 32). We similarly investigated EXIT fusions to Rv1002c, which O-mannosylates exported proteins and contributes to virulence (51, 52). As with MmpL3, different prediction programs generate discordant models for Rv1002c (see Fig. S3 in the supplemental material). In this case, of the five models consulted, the HMMTOP prediction (53) was the best match as it positioned the enriched EXIT fusions in two extracytoplasmic domains and the C-terminus (Fig. 4; Fig. S3); this model was also the most similar to the topology of the homologous yeast O-mannosyltransferase (54). It should be noted that our analysis did not identify any one prediction program as being better than others overall, including TMHMM (55) which is used on Tuberculist (56); rather, it emphasized the value of the EXIT data to select the best model. For each of the 593 EXIT proteins, the site of enriched fusions to the reporter as well as all the in-frame EXIT fusions in the input library are provided (Table S1; Table S4). The list of total fusions will be useful for identifying nonenriched fusions to predict cytoplasmic domains. However, there are alternate explanations besides a cytoplasmic location for unenriched ‘BlaTEM fusions (e.g., unstable fusion proteins). To definitively assign cytoplasmic domains will require testing fusions to cytoplasmic reporters of protein topology.

For the 38 proteins identified as *in vivo* induced exported proteins (Table 1), the combination of an exported location and host regulation makes them compelling candidates for being virulence factors. Using bone marrow macrophages, we showed that mutants of four of the *in vivo* induced exported proteins of unknown function (Rv0559c, Rv1508c, Rv2536, and Rv3707c) are defective for intracellular growth in macrophages. For Rv0559c and Rv1508c, this is the first indication that they function in *M. tuberculosis* virulence. For Rv2536, the protein is predicted by Tnseq to play a role during murine infection (15); however, our data are the first to suggest a specific role promoting *M. tuberculosis* growth in macrophages. Lastly, while the Rv3707c homolog in *Mycobacterium bovis* BCG is known to promote growth in macrophages (57), the protein remains unstudied in *M. tuberculosis*. The specific functions of all four of these *in vivo* induced proteins in macrophages remain a mystery and warrant further study. Future studies should explore the other *in vivo* induced exported proteins for potential virulence functions.

The list of *in vivo* induced exported proteins also sheds light on conditions encountered during infection that are not recapitulated during *in vitro* growth. For example, the identification of SenX3, a sensor histidine kinase of the SenX3-RegX3 two-component system that responds to low phosphate levels (58, 59), suggests that *M. tuberculosis* encounters phosphate-limiting conditions during murine infection. The identification of MmcO, a multicopper oxidase that protects against copper toxicity (60, 61), is consistent with *M. tuberculosis* experiencing a high-copper environment during infection (62).

Past efforts to identify bacterial proteins exported during infection focused on direct testing of preselected proteins for secretion into cultured cells through microscopy or subcellular fractionation (63–65). In comparison, EXIT provides a tool for large-scale discovery of *in vivo* exported proteins. A recent MS-based proteomics approach for identifying labeled bacterial proteins secreted into cultured cells holds promise as a potential alternate discovery strategy (66). However, as with other proteomics studies of secreted proteins, a challenge facing this new methodology is that of avoiding identification of cytoplasmic proteins released by unintended bacterial lysis (67).

In summary, here we introduce EXIT as an effective and robust method to identify bacterial proteins exported in a whole-animal model of infection. For the *M. tuberculosis* research community, the data generated during the course of this work represent a valuable functional genomics resource for assigning function to uncharacterized proteins. For the larger microbiology community, EXIT provides a method that could be adapted to other bacterial pathogens. This study focused on application of EXIT during acute murine infection with *M. tuberculosis*. However, the ‘BlaTEM reporter and EXIT methodology are theoretically compatible with any bacterium that is either naturally β-lactam sensitive or can be made so genetically. In the future, EXIT could be used to study the *in vivo* exportome of other pathogens or different stages of infection.

## MATERIALS AND METHODS

### Bacterial strains and growth.

Bacterial strains and plasmids are listed in Table S6 in the supplemental material. *M. tuberculosis* strains were grown with Middlebrook 7H9 broth or 7H10 agar (Difco) supplemented with 1× albumin dextrose saline (ADS), 0.5% glycerol, and 0.05% Tween 80 (7AGT) (68). As needed, growth medium was supplemented with 20 µg/ml kanamycin (Acros), 50 µg/ml hygromycin (Roche), or 50 µg/ml carbenicillin (Sigma). *Escherichia coli* strains were grown on Luria-Bertani medium (Fisher) supplemented as necessary with 40 μg/ml kanamycin, 150 μg/ml hygromycin, and 100 μg/ml carbenicillin.

10.1128/mBio.00333-17.10 TABLE S6  Reagents used in this study. Download TABLE S6, DOCX file, 0.03 MB. Copyright © 2017 Perkowski et al. 2017 Perkowski et al. This content is distributed under the terms of the Creative Commons Attribution 4.0 International license.

### EXIT library construction.

*M. tuberculosis* genomic DNA (gDNA) was prepared as previously described (69) from the *M. tuberculosis* Δ*blaC* mutant, named PM638 (13). Genomic DNA fragments between 500 bp and 5 kb in size were generated by partial digestion with AciI and HpaII and cloned into the multicopy, hygromycin-marked, EXIT library plasmid pDW31 (see Text S1 in the supplemental material for pDW31 construction) using the unique ClaI site located immediately upstream of the ‘*bla*_TEM_ reporter. Ligated plasmids were transformed into MegaX DH10 electrocompetent cells (Invitrogen). *E. coli* transformants (5.6 × 10^6^) were pooled, and plasmids were isolated using a QiaFilter Plasmid Giga kit (Qiagen). Plasmids isolated from *E. coli* were next transformed into PM638, *M. tuberculosis* H37Rv Δ*blaC* (13), as previously described (68). *M. tuberculosis* transformants (5.4 × 10^6^) from 50 transformations were pooled to produce the input EXIT library used to infect mice. The input library was subjected to next-generation sequencing using a primer at the fusion junction to ‘*bla*_TEM_ (Table S6, primers). On average, the library contained a fusion every 26 bp in the *M. tuberculosis* genome, with the largest nonrepresented region of the genome being only 110 nucleotides long. The complexity of the library was such that each gene was represented by an average of 16 in-frame fusions, and some genes contained more than 35 in-frame fusions. A total of 99% of the genes in the *M. tuberculosis* genome were represented by at least one in-frame fusion.

10.1128/mBio.00333-17.1 TEXT S1  Supplemental methods. Download TEXT S1, DOCX file, 0.02 MB. Copyright © 2017 Perkowski et al. 2017 Perkowski et al. This content is distributed under the terms of the Creative Commons Attribution 4.0 International license.

### Mouse infection with the EXIT library.

For each experiment, 8-to-10-week-old female BALB/c mice were intravenously infected, as previously described (44), with 3 × 10^6^ *M. tuberculosis* bacteria from the EXIT library, of which approximately 20% seeded the spleen and 1% seeded the lungs (data not shown), consistent with previous studies (17). For each experiment, 30 mice were infected. At 1 day after infection, organs from six mice were harvested to determine the initial dose and organ burden. At 1 day after infection, the remaining 24 mice began receiving treatment twice daily by oral gavage with 40 mg amoxicillin (MP Biomedicals 190145 or Sigma A8523) and 8 mg probenecid (Sigma P8761) administered in 0.25 M NaOH–phosphate-buffered saline (PBS). Probenecid, a synergistic drug, is used in conjunction with amoxicillin to reduce drug efflux in the kidneys, increasing the serum concentration (70). The use of 24 animals per experiment was based on calculations performed to achieve a 99.5% probability that any individual clone in the EXIT library would establish infection in the spleen of at least one mouse in each replicate experiment (by calculations using the binomial equation 1 − *P* = [*Q*]^*n*^, where *n* represents the number of mice, *Q* represents the probability of failure in each individual mouse, and *P* represents the probability of overall success). At 14 days postinfection, mice were euthanized, and spleens and lungs were harvested to collect surviving bacteria. Organ homogenates were plated undiluted onto 7H10 agar. These recovered fusions were used to identify *in vivo* exported proteins. For determining fusions exported both *in vivo* and *in vitro*, organ homogenates were plated in parallel onto 7H10 agar containing carbenicillin (a β-lactam). Plates were incubated at 37°C for 3 weeks, after which colonies were pooled for plasmid DNA isolation (see Text S1). All mice were maintained under specific-pathogen-free conditions in a biosafety level 3 (BSL-3) facility. Mice were assigned randomly to experimental groups, and the mouse studies were not performed in a blind fashion. All procedures involving the use of animals were in compliance with protocols approved by the University of North Carolina Chapel Hill Institutional Animal Care and Use Committee and Biosafety Committee.

### **Next-generation sequencing, data analysis, and statistical modeling (see** Text S1 **for additional details).**

Sample preparation and sequencing strategies for the ‘*bla*_TEM_ fusion junction that includes upstream *M. tuberculosis* genomic DNA are provided in Text S1. Samples were sequenced using next-generation sequencing (Illumina HiSeq), generating paired-end multiplexed sequencing reads. To identify fusion sites, reads were trimmed of adapter sequences and aligned to the H37Rv genome. For statistical analysis, unique reads for each fusion site were counted using read counts that were first normalized to the total number of sequenced reads in each sample as follows. (i) To identify proteins exported *in vivo*, fusions recovered on standard 7H10 agar from β-lactam-treated mice (*in vivo*) were subjected to statistical analysis. The most abundant fusion position within each annotated gene was identified individually within the output for each of the two EXIT experiments. The lower of these two numbers from comparisons between replicates was used as the abundance value for the gene to require that any identified gene was highly abundant in both samples. Log_10_ values were used to generate a histogram, which was bimodal. A Gaussian mixture model was then used to identify the mean and variance for each population and to determine the probability that a fusion was in the higher-abundance or lower-abundance population (Fig. 2a). The abundance levels in the unselected input library were relatively uniform; thus, computation of enrichment ratios was not required and the statistical analysis was done on the distribution of abundances. (ii) To identify the *in vivo* induced exported proteins, fusions recovered on β-lactam-containing agar (*in vitro*) were subjected to statistical analysis and, in this case, the higher abundance value from comparisons between replicates was used as a representative abundance value for the gene, to identify the most stringent list of proteins that were not exported *in vitro* in either experiment. The log_10_ value of the ratio between the abundance seen following *in vivo* treatment and that seen following *in vivo* plus *in vitro* treatment was calculated. The top and bottom 5% were trimmed for robustness. These data fit a normal unimodal distribution, where genes of interest had high ratios of *in vivo* reads versus *in vivo* plus *in vitro* reads. A normal fit distribution was used to identify outliers, with higher ratios than would be predicted by chance. The Benjamini-Hochberg procedure was used to correct for multiple comparisons and identified genes with a *P* value of <0.0005 (false-discovery rate, <0.05). Corrected *P* values (*q* values) are reported (Table 1). (iii) To identify all individual enriched fusion junctions in an ORF for topology determination, the number of reads for each fusion site in the output from β-lactam-treated mice was divided by the number of sequenced reads in the corresponding input for each experiment. Log_10_ enrichment values were used to generate histograms, which produced a unimodal distribution with a right shoulder of enriched sites. A Gaussian mixture model was fitted to the distribution using Mclust in R (71). The resulting mixture models had two peaks, one representing the majority of the sites and a second, smaller peak representing points in the right shoulder representing the enriched fusions. Fusion sites that were statistically enriched in both experiments were considered to be exported.

### Subcellular fractionation and immunoblotting.

*M. tuberculosis* cells were pelleted by centrifugation, sterilized by irradiation (JL Shepherd Mark I-137Cs irradiator), and removed from BSL-3 containment. Subcellular fractionation was performed by differential ultracentrifugation as previously described (35), generating clarified whole-cell lysates (WCL) and cell wall (CW), membrane (MEM), and soluble cytoplasmic (SOL) fractions. Fractions from equivalent original cell material were separated by SDS-PAGE and transferred to nitrocellulose membranes. Proteins were detected using the primary anti-HA antibody (Covance) (1:25,000), anti-SigA antibody (a gift from Murty Madiraju [72]) (1:20,000), 19kd (a gift from Douglas Young, Imperial College London, United Kingdom) (1:20,000), and HbhA (BEI Resources [38]) (1:5,000) and secondary anti-mouse- and anti-rabbit-conjugated horseradish peroxidase (HRP) (Bio-Rad). HRP signal was detected using an enhanced chemiluminescence kit (PerkinElmer).

### Identification of export signals.

Sequences were analyzed for transmembrane domains and signal peptides using TMHMM (55) and Signal P (73). Previous analyses of the *M. tuberculosis* genome performed with LipoP, TatP, TATFIND, and TigrFAM were used to identify proteins with lipoprotein or Tat signal peptides (74, 75). PE/PPE proteins were analyzed for YxxxD/E motifs (49).

### Macrophage infections.

The following reagents were obtained through BEI Resources, NIAID, NIH: *Mycobacterium tuberculosis* strain CDC1551 transposon mutants (33) (Table S6). *M. tuberculosis* mutants were validated by PCR and Southern blotting (data not shown). Bone marrow-derived macrophages were isolated utilizing C57BL/6 mice as described previously (76). The macrophages were infected with *M. tuberculosis* strains at a multiplicity of infection (MOI) of 1 for 4 h. After infection, the macrophages were washed three times to remove extracellular bacteria. At time points postinfection, the macrophages were lysed using 1% Triton X-100 (Sigma), and the lysates were diluted and plated for CFU determinations on 7H10 (Difco) or 7H11 (Sigma) plates supplemented with 0.05% Tween 80, 0.5% glycerol, 1× albumin dextrose saline (ADS), and 20 µg/ml kanamycin (Acros).

### Data availability.

Raw sequencing data will be made available upon request.

### Code availability.

The code developed for analyzing the sequencing data will be available through GitHub (http://github.com/gomezlab/exit), a publicly available repository, under an open source license.
