# Erratum for Perkowski et al., “The EXIT Strategy: an Approach for Identifying Bacterial Proteins Exported during Host Infection”

**DOI:** 10.1128/mBio.00872-17

**Published:** 2017-06-20

**Authors:** E. F. Perkowski, K. E. Zulauf, D. Weerakoon, J. D. Hayden, T. R. Ioerger, D. Oreper, S. M. Gomez, J. C. Sacchettini, M. Braunstein

**Affiliations:** a Department of Microbiology and Immunology, University of North Carolina—Chapel Hill, Chapel Hill, North Carolina, USA; b Department of Computer Science and Engineering, Texas A&M University, College Station, Texas, USA; c Joint Department of Biomedical Engineering at UNC—Chapel Hill and NC State University, Chapel Hill, North Carolina, USA; d Department of Biochemistry and Biophysics, Texas A&M University, College Station, Texas, USA

## ERRATUM

Volume 8, no. 2, e00333-17, 2017, https://doi.org/10.1128/mBio.00333-17. We correct the following error in our published paper. The sentence in the Discussion (page 9 of the PDF) listing HbhA as one of a set of examples of previously known exported proteins that we identified using the EXIT strategy is incorrect. HbhA was not identified in our study. This textual error does not impact the findings or the conclusions of our paper, and it does not change the list of 593 proteins (Table S1) that we did identify as exported *in vivo* in our study.

