# Supplementary material for: The EXIT Strategy: an Approach for Identifying Bacterial Proteins Exported during Host Infection
Source: mBio. 2017 Apr 25;8(2):e00333-17. doi: 10.1128/mBio.00333-17 (PMC5405230; doi:10.1128/mBio.00333-17)
Supplement: TABLE S1 [file mbo002173284st1.docx]

| **Supplemental Table 1. EXIT results** | | | | | | | | |
| --- | --- | --- | --- | --- | --- | --- | --- | --- |
| **ORF number** | **Name** | **Product** | **Exported fusion junctions (aa location)** | **ID in Lung** | ***in silico* Export signal** | **Subcellular localization by Mass Spec** | **Exported by Genetic Reporter** | **Predicted to be essential *in vitro* or during infection** |
| Rv0011c |  | PROBABLE CONSERVED TRANSMEMBRANE PROTEIN | 83 |  | TM | MEM 8, MEM 14, CW 14 |  |  |
| Rv0012 |  | PROBABLE CONSERVED MEMBRANE PROTEIN | 39, 46, 93, 102 |  | TM | MEM 12, MEM 15, MEM 14, CW 14 |  | Mouse 24 |
| Rv0014c | *pknB* | TRANSMEMBRANE SERINE/THREONINE-PROTEIN KINASE B PKNB (PROTEIN KINASE B) (STPK B) | 404, 421, 445, 446, 594 |  | TM | CF 8, CF 9, MEM 8, MEM 14, MEM 9, CW 15, CW 19, CW 14, SOL 9 |  | *in vitro* 25, *in vitro* 22 |
| Rv0015c | *pknA* | TRANSMEMBRANE SERINE/THREONINE-PROTEIN KINASE A PKNA (PROTEIN KINASE A) (STPK A) | 362 |  | TM | MEM 15, MEM 14, CW 14 |  | *in vitro* 25, *in vitro* 22 |
| Rv0016c | *pbpA* | PROBABLE PENICILLIN-BINDING PROTEIN PBPA | 29, 51 |  | SP, TM | MEM 8, MEM 14, CW 14 |  | Mouse 24 |
| Rv0017c | *rodA, ftsW* | PROBABLE CELL DIVISION PROTEIN RODA | 412 |  | TM | MEM 14, CW 14 |  | Macrophage 20, Mouse 24 |
| Rv0037c |  | PROBABLE CONSERVED INTEGRAL MEMBRANE PROTEIN | 122, 349, 377 |  | TM | MEM 8, MEM 14, CW 14 |  | Macrophage 20 |
| Rv0039c |  | POSSIBLE CONSERVED TRANSMEMBRANE PROTEIN | 45, 50, 51, 58, 60, 95 | Yes | SP, TM |  |  |  |
| Rv0040c | *mtc28* | SECRETED PROLINE RICH PROTEIN MTC28 (PROLINE RICH 28 KDA ANTIGEN) | 61, 85, 102, 178, 275, 283 | Yes | SP, TM | CF 8, CF 9, MEM 15, CW 14 |  | Mouse 24 |
| Rv0048c |  | POSSIBLE MEMBRANE PROTEIN | 123, 148, 201 |  | TM | CF 9, MEM 4, MEM 11, MEM 8, MEM 14, MEM 9, CW 15, CW 19, CW 14 |  |  |
| Rv0062 | *celA1, celA* | POSSIBLE CELLULASE CELA1 (ENDOGLUCANASE) (ENDO-1,4-BETA-GLUCANASE) (FI-CMCASE) (CARBOXYMETHYL CELLULASE) | 91, 99, 106, 168, 173, 183, 191, 268, 308 | Yes | TM | CF 8, MEM 15, CW 14 |  |  |
| Rv0064 |  | PROBABLE CONSERVED TRANSMEMBRANE PROTEIN | 275, 316, 423, 529 | Yes | TM | MEM 15, CW 15 | PhoA 17, BlaTEM 18 |  |
| Rv0072 |  | PROBABLE GLUTAMINE-TRANSPORT TRANSMEMBRANE PROTEIN ABC TRANSPORTER | 33, 59, 80, 83, 165, 275 | Yes | TM | MEM 4, MEM 11, MEM 8, MEM 14, MEM 9, CW 19, CW 14 | BlaTEM 18 |  |
| Rv0076c |  | PROBABLE MEMBRANE PROTEIN | 125 |  | TM |  |  |  |
| Rv0083 |  | PROBABLE OXIDOREDUCTASE | 303, 368, 385, 493, 498 |  | TM | MEM 14 |  |  |
| Rv0084 | *hycD, hevD* | POSSIBLE FORMATE HYDROGENLYASE HYCD (FHL) | 86, 174, 248, 250, 253, 306 | Yes | TM |  |  | Macaque 19 |
| Rv0086 | *hycQ* | POSSIBLE HYDROGENASE HYCQ | 58, 184, 189, 480 | Yes | TM |  |  | Mouse 24, *in vitro* 25 |
| Rv0092 | *ctpA* | PROBABLE CATION TRANSPORTER P-TYPE ATPASE A CTPA | 127, 374, 379, 444 | Yes | TM | MEM 14, CW 15, CW 14 | BlaTEM 18 | Macrophage 20 |
| Rv0093c |  | PROBABLE CONSERVED MEMBRANE PROTEIN | 161, 189, 191, 214 | Yes | TM | MEM 8, MEM 14, CW 15 |  |  |
| Rv0102 |  | PROBABLE CONSERVED INTEGRAL MEMBRANE PROTEIN | 52, 71, 75, 136, 150, 156, 198, 213, 331, 416 | Yes | TM | MEM 8, MEM 14, CW 14 |  | *in vitro* 25, *in vitro* 22 |
| Rv0103c | *ctpB* | PROBABLE CATION-TRANSPORTER P-TYPE ATPASE B CTPB | 130, 140, 213, 221 | Yes | TM | MEM 14, CW 14 |  |  |
| Rv0110 |  | PROBABLE CONSERVED INTEGRAL MEMBRANE PROTEIN | 72, 191 |  | TM | MEM 8 |  |  |
| Rv0111 |  | POSSIBLE TRANSMEMBRANE ACYLTRANSFERASE | 70, 125, 428, 439, 457 | Yes | TM | MEM 12, CW 12, CW 15 |  |  |
| Rv0116c |  | POSSIBLE CONSERVED MEMBRANE PROTEIN | 18, 26, 82, 120, 192 |  | SP, TM | CF 8, MEM 15 | BlaTEM 18 |  |
| Rv0125 | *pepA, mtb32a* | PROBABLE SERINE PROTEASE PEPA (SERINE PROTEINASE) (MTB32A) | 45, 77, 109, 132, 134, 144, 186, 204, 266, 299, 304 | Yes | SP, Tat SP, TM | CF 8, CF 9, MEM 15, MEM 8, MEM 14, CW 15, CW 19, CW 14, WCL 9, SOL 9 | PhoA 17, BlaTEM 18 | Mouse 24 |
| Rv0128 |  | PROBABLE CONSERVED TRANSMEMBRANE PROTEIN | 39, 195 |  | TM |  |  |  |
| Rv0143c |  | PROBABLE CONSERVED TRANSMEMBRANE PROTEIN | 94, 97, 245, 334, 342 | Yes | TM | MEM 8, MEM 14 |  |  |
| Rv0157 | *pntB* | PROBABLE NAD(P) TRANSHYDROGENASE (SUBUNIT BETA) PNTB [INTEGRAL MEMBRANE PROTEIN] (PYRIDINE NUCLEOTIDE TRANSHYDROGENASE SUBUNIT BETA) (NICOTINAMIDE NUCLEOTIDE TRANSHYDROGENASE SUBUNIT BETA) | 63, 190, 192, 253, 260 | Yes | TM | MEM 4, MEM 11, MEM 14, MEM 9, CW 19, CW 14, WCL 9 |  | Mouse 24 |
| Rv0167 | *yrbE1A* | CONSERVED HYPOTHETICAL INTEGRAL MEMBRANE PROTEIN YRBE1A | 88, 90, 110, 112, 183, 194 | Yes | TM | MEM 4, MEM 8, MEM 14, CW 14 |  |  |
| Rv0168 | *yrbE1B* | CONSERVED HYPOTHETICAL INTEGRAL MEMBRANE PROTEIN YRBE1B | 86, 108 |  | TM | MEM 8, MEM 14, CW 14 |  |  |
| Rv0169 | *mce1A, mce1* | MCE-FAMILY PROTEIN MCE1A | 37, 50, 59, 116, 179, 400 | Yes | TM | MEM 8, MEM 14, CW 15, CW 19, CW 14 | BlaTEM 18 | Macrophage 20, Macrophage 21, Mouse 22 |
| Rv0170 | *mce1B, mceD* | MCE-FAMILY PROTEIN MCE1B | 39, 46, 154 |  | TM | MEM 4, MEM 15, MEM 8, MEM 14, CW 19, CW 14, WCL 9 | BlaTEM 18 | Macrophage 20, Macrophage 21, Mouse 22 |
| Rv0171 | *mce1C* | MCE-FAMILY PROTEIN MCE1C | 48, 49, 60, 146, 242, 338, 372, 449, 461, 489, 503, 504 | Yes | SP, TM | MEM 8, MEM 14, CW 19, CW 14 | BlaTEM 18 | Macrophage 20, Mouse 22 |
| Rv0172 | *mce1D* | MCE-FAMILY PROTEIN MCE1D | 472 |  | TM | CF 8, CF 9, MEM 11, MEM 15, MEM 8, MEM 14, CW 15, CW 19, CW 14, WCL 9 | BlaTEM 18 | Macrophage 20 |
| Rv0173 | *lprK, mce1E* | POSSIBLE MCE-FAMILY LIPOPROTEIN LPRK (MCE-FAMILY LIPOPROTEIN MCE1E) | 30, 33, 41, 42 | Yes | Lipo, TM | MEM 11, MEM 15, MEM 8, MEM 14, CW 15, CW 19, CW 14, WCL 9 | BlaTEM 18 | Macaque 19, Macrophage 20, Mouse 22 |
| Rv0174 | *mce1F* | MCE-FAMILY PROTEIN MCE1F | 46, 60, 161 | Yes | TM | CF 8, CF 9, MEM 12, MEM 15, MEM 8, MEM 14, CW 15, CW 19, CW 14, WCL 9 | BlaTEM 18 | Macrophage 20 |
| Rv0175 |  | PROBABLE CONSERVED MCE ASSOCIATED MEMBRANE PROTEIN | 66 |  | TM | CF 9, MEM 11, MEM 14, MEM 9, CW 19, CW 14, WCL 9 | PhoA 17, BlaTEM 18 | Macrophage 20, Mouse 22 |
| Rv0176 |  | PROBABLE CONSERVED MCE ASSOCIATED TRANSMEMBRANE PROTEIN | 279 |  | TM | MEM 4, MEM 8, MEM 14, CW 14 |  | Macrophage 20, Macrophage 21, Mouse 22 |
| Rv0177 |  | PROBABLE CONSERVED MCE ASSOCIATED PROTEIN | 115 |  | TM | MEM 4, MEM 8, MEM 14, CW 14 |  | Macrophage 20, Macrophage 21, Mouse 24 |
| Rv0178 |  | PROBABLE CONSERVED MCE ASSOCIATED MEMBRANE PROTEIN | 119, 162, 177 | Yes | TM | CF 9, MEM 4, MEM 11, MEM 14, MEM 9, CW 19, CW 14, WCL 9 | BlaTEM 18 | Macrophage 20 |
| Rv0179c | *lprO* | POSSIBLE LIPOPROTEIN LPRO | 36, 40, 43, 67, 124, 159, 169, 183, 199, 273, 277, 296 | Yes | SP, Lipo, TM | CW 14 | PhoA 17 | Macaque 19, Macrophage 20 |
| Rv0180c |  | PROBABLE CONSERVED TRANSMEMBRANE PROTEIN | 40, 45, 66, 68, 79, 153, 180, 181, 191, 211 | Yes | TM | MEM 4, MEM 11, MEM 8, MEM 14, MEM 9, CW 19, CW 14, WCL 9 |  | Mouse 24 |
| Rv0191 |  | PROBABLE CONSERVED INTEGRAL MEMBRANE PROTEIN | 113, 310, 318, 382 | Yes | TM | MEM 14 |  |  |
| Rv0192A |  | CONSERVED SECRETED PROTEIN | 40, 46 |  | SP, Tat SP, TM |  |  |  |
| Rv0199 | *omamA* | PROBABLE CONSERVED MEMBRANE PROTEIN | 64, 70, 82, 155, 157 | Yes | TM | MEM 11, MEM 14, MEM 9, CW 19, CW 14, WCL 9 | BlaTEM 18 | Mouse 22, Mouse 24 |
| Rv0200 |  | POSSIBLE CONSERVED TRANSMEMBRANE PROTEIN | 22, 129, 145, 161, 172, 195 |  | TM | MEM 4, MEM 14, CW 14 |  | Macrophage 21 |
| Rv0202c | *mmpL11* | PROBABLE CONSERVED TRANSMEMBRANE TRANSPORT PROTEIN MMPL11 | 37, 39, 44, 166, 168, 407, 432, 433, 436 | Yes | SP, TM | MEM 14, CW 15, CW 14 |  | Macrophage 21 |
| Rv0203 |  | POSSIBLE EXPORTED PROTEIN | 23, 34, 119 | Yes | SP, Tat SP, TM | CF 8, CF 9, MEM 15 |  |  |
| Rv0205 |  | PROBABLE CONSERVED TRANSMEMBRANE PROTEIN | 87, 114, 127, 262, 324, 348 | Yes | TM | MEM 8, MEM 14 |  | Mouse 24, *in vitro* 25 |
| Rv0206c | *mmpL3* | POSSIBLE CONSERVED TRANSMEMBRANE TRANSPORT PROTEIN MMPL3 | 25, 40, 65, 76, 108, 117, 121, 163, 173, 205, 419, 444, 479 | Yes | TM | CF 9, MEM 11, MEM 8, MEM 14, MEM 9, CW 15, CW 19, CW 14, WCL 9 | PhoA 17 | *in vitro* 22 |
| Rv0218 |  | PROBABLE CONSERVED TRANSMEMBRANE PROTEIN | 64, 81, 90, 162, 179, 295 |  | Tat SP, TM | MEM 8 |  | Mouse 22 |
| Rv0219 |  | PROBABLE CONSERVED TRANSMEMBRANE PROTEIN | 130 |  | TM | MEM 15, MEM 14 |  |  |
| Rv0226c |  | PROBABLE CONSERVED TRANSMEMBRANE PROTEIN | 22, 169, 311, 343, 348 | Yes | Tat SP, TM | MEM 8, MEM 14 |  | *in vitro* 25, *in vitro* 22 |
| Rv0227c |  | PROBABLE CONSERVED MEMBRANE PROTEIN | 79, 219, 236, 326 | Yes | SP, TM | CF 9, MEM 4, MEM 11, MEM 12, MEM 8, MEM 14, MEM 9, CW 15, CW 19, CW 14, WCL 9 |  | *in vitro* 25, *in vitro* 22 |
| Rv0228 |  | PROBABLE INTEGRAL MEMBRANE ACYLTRANSFERASE | 212, 351 |  | TM |  |  | *in vitro* 25, *in vitro* 22 |
| Rv0236c |  | PROBABLE CONSERVED TRANSMEMBRANE PROTEIN | 24, 36, 191, 248, 329, 346, 425, 452, 510, 597, 1365 | Yes | SP, TM | MEM 12, MEM 14 |  | *in vitro* 25, *in vitro* 22 |
| Rv0237 | *lpqI* | PROBABLE CONSERVED LIPOPROTEIN LPQI | 22, 26, 32, 36, 88, 111 | Yes | SP, Lipo | CF 8, CF 9, MEM 11, MEM 15, MEM 8, MEM 14, MEM 9, CW 15, CW 19, CW 14 |  |  |
| Rv0249c |  | PROBABLE SUCCINATE DEHYDROGENASE [MEMBRANE ANCHOR SUBUNIT] (SUCCINIC DEHYDROGENASE) | 80, 180 | Yes | TM | MEM 8, MEM 14, MEM 9, CW 19, WCL 9 |  | Mouse 22, Mouse 24, *in vitro* 22 |
| Rv0261c | *narK3* | PROBABLE INTEGRAL MEMBRANE NITRITE EXTRUSION PROTEIN NARK3 (NITRITE FACILITATOR) | 62, 331 |  | TM |  |  |  |
| Rv0283 | *eccB3* | POSSIBLE CONSERVED MEMBRANE PROTEIN | 111, 154, 422, 526 | Yes | TM | CF 9, MEM 4, MEM 11, MEM 15, MEM 8, MEM 14, MEM 9, CW 15, CW 19, CW 14, WCL 9, SOL 9 |  | *in vitro* 25, *in vitro* 22 |
| Rv0291 | *mycP3* | PROBABLE MEMBRANE-ANCHORED MYCOSIN MYCP3 (SERINE PROTEASE) (SUBTILISIN-LIKE PROTEASE) (SUBTILASE-LIKE) (MYCOSIN-3) | 433 |  | SP, TM | CF 8, CF 9, MEM 4, MEM 11, MEM 15, MEM 8, MEM 14, MEM 9, CW 15, CW 19, CW 14, WCL 9, SOL 9 |  | *in vitro* 25, *in vitro* 22 |
| Rv0309 |  | POSSIBLE CONSERVED EXPORTED PROTEIN | 48, 49, 61, 63, 70, 101, 105 |  | SP, TM | CF 8, MEM 15, MEM 8, CW 15, CW 14 |  |  |
| Rv0312 |  | CONSERVED HYPOTHETICAL PROLINE AND THREONINE RICH PROTEIN | 479, 486, 551 |  | TM | MEM 14, CW 14 | BlaTEM 18 | Mouse 24, *in vitro* 25 |
| Rv0314c |  | POSSIBLE CONSERVED MEMBRANE PROTEIN | 132, 139, 192, 211 |  | TM | MEM 8, MEM 14, CW 14 |  |  |
| Rv0338c |  | PROBABLE IRON-SULFUR-BINDING REDUCTASE | 119, 203 |  | TM | MEM 4, MEM 11, MEM 8, MEM 14, MEM 9, CW 15, CW 19, CW 14, WCL 9 |  | *in vitro* 25, *in vitro* 22 |
| Rv0344c | *lpqJ* | PROBABLE LIPOPROTEIN LPQJ | 50, 68, 71, 90, 126 |  | SP, Lipo | MEM 14, CW 14 |  |  |
| Rv0346c | *ansP2, aroP2* | POSSIBLE L-ASPARAGINE PERMEASE ANSP2 (L-ASPARAGINE TRANSPORT PROTEIN) | 52, 118, 234, 273, 312, 376 |  | TM | MEM 8, MEM 14, CW 14 | BlaTEM 18 | Macrophage 21 |
| Rv0359 |  | PROBABLE CONSERVED INTEGRAL MEMBRANE PROTEIN | 42, 112 |  | TM | MEM 8, MEM 14 |  |  |
| Rv0361 |  | PROBABLE CONSERVED MEMBRANE PROTEIN | 182, 186 | Yes | TM | CF 9, MEM 11, MEM 8, MEM 14, MEM 9, CW 15, CW 19, CW 14, WCL 9, SOL 9 | BlaTEM 18 | Mouse 24 |
| Rv0398c |  | POSSIBLE SECRETED PROTEIN | 20, 24, 30, 41, 50, 206 |  | SP, TM | CF 8, CF 9, MEM 15, SOL 9 |  |  |
| Rv0399c | *lpqK* | POSSIBLE CONSERVED LIPOPROTEIN LPQK | 24 |  | SP, Lipo | MEM 8, MEM 14, CW 14 |  | *in vitro* 25 |
| Rv0402c | *mmpL1* | PROBABLE CONSERVED TRANSMEMBRANE TRANSPORT PROTEIN MMPL1 | 123, 788, 864 |  | TM | MEM 15, MEM 8, MEM 14, CW 15, CW 14 | BlaTEM 18 |  |
| Rv0403c | *mmpS1* | PROBABLE CONSERVED MEMBRANE PROTEIN MMPS1 | 40 |  | SP, TM | CF 8, MEM 14 |  | Mouse 24 |
| Rv0411c | *glnH* | PROBABLE GLUTAMINE-BINDING LIPOPROTEIN GLNH (GLNBP) | 25, 68 | Yes | SP, Tat SP, Lipo | CF 8, MEM 15, MEM 8, MEM 14, MEM 9, CW 14 |  | *in vitro* 25, *in vitro* 22 |
| Rv0412c |  | POSSIBLE CONSERVED MEMBRANE PROTEIN | 65, 90, 94, 103, 269 |  | TM | MEM 11, MEM 8, MEM 14, CW 14 | BlaTEM 18 | Mouse 24, *in vitro* 25, *in vitro* 22 |
| Rv0418 | *lpqL* | PROBABLE LIPOPROTEIN AMINOPEPTIDASE LPQL | 38, 105, 165, 188, 278, 459 | Yes | SP, Lipo, TM | CF 9, MEM 4, MEM 11, MEM 8, MEM 14, CW 14 | BlaTEM 18 |  |
| Rv0420c |  | POSSIBLE TRANSMEMBRANE PROTEIN | 65, 92, 113, 131 | Yes | Tat SP, TM |  |  |  |
| Rv0426c |  | POSSIBLE TRANSMEMBRANE PROTEIN | 83 | Yes | SP, TM | MEM 8, MEM 14, MEM 9, CW 19, CW 14, WCL 9 |  | Macrophage 20 |
| Rv0431 | *AT103* | PUTATIVE TUBERCULIN RELATED PEPTIDE | 71, 89, 100, 131, 155 | Yes | TM | CF 8, CF 9, MEM 11, MEM 8, MEM 14, MEM 9, CW 15, CW 19, CW 14 |  | *in vitro* 22 |
| Rv0432 | *sodC* | PROBABLE PERIPLASMIC SUPEROXIDE DISMUTASE [CU-ZN] SODC | 50, 53, 66, 85, 102, 109, 130, 131, 134, 139, 140, 153, 164, 189, 224, 233 | Yes | SP, Lipo | CF 9, MEM 4, MEM 11, MEM 8, MEM 14, MEM 9, CW 15, CW 19, CW 14, WCL 9 | BlaTEM 18 | Mouse 24 |
| Rv0446c |  | POSSIBLE CONSERVED TRANSMEMBRANE PROTEIN | 120, 123 |  | TM | MEM 15, MEM 14 |  |  |
| Rv0450c | *mmpL4* | PROBABLE CONSERVED TRANSMEMBRANE TRANSPORT PROTEIN MMPL4 | 149, 182, 185, 188, 239, 248, 301, 420, 427, 500 |  | TM | CF 9, MEM 12, MEM 8, MEM 14, MEM 9, CW 15, CW 14 | PhoA 17, BlaTEM 18 | *in vitro* 25, *in vitro* 22 |
| Rv0451c | *mmpS4* | PROBABLE CONSERVED MEMBRANE PROTEIN MMPS4 | 25, 60, 138 | Yes | TM | MEM 14 |  |  |
| Rv0455c |  | CONSERVED HYPOTHETICAL PROTEIN | 19, 138 |  | SP, TM | CF 8, CF 9, MEM 15, MEM 14, CW 19, CW 14, WCL 9, SOL 9 | PhoA 17 | *in vitro* 22 |
| Rv0461 |  | PROBABLE TRANSMEMBRANE PROTEIN | 105, 149 |  | TM | MEM 8, MEM 14 |  |  |
| Rv0476 |  | POSSIBLE CONSERVED TRANSMEMBRANE PROTEIN | 27, 67 |  | SP, TM | MEM 8, MEM 14 |  |  |
| Rv0477 |  | POSSIBLE CONSERVED SECRETED PROTEIN | 28 |  | SP, | CF 8, CF 9, MEM 15 |  |  |
| Rv0479c |  | PROBABLE CONSERVED MEMBRANE PROTEIN | 125, 155, 180 |  | TM | MEM 4, MEM 11, MEM 8, MEM 14, MEM 9, CW 19, CW 14, WCL 9 |  | *in vitro* 25, *in vitro* 22 |
| Rv0490 | *senX3* | PUTATIVE TWO COMPONENT SENSOR HISTIDINE KINASE SENX3 | 121 |  | TM | MEM 15, MEM 14, CW 14 |  | Mouse 22 |
| Rv0497 |  | PROBABLE CONSERVED TRANSMEMBRANE PROTEIN | 244 |  | TM | MEM 8, MEM 14, CW 15, CW 14 |  | Macrophage 21, Mouse 24, *in vitro* 22 |
| Rv0506 | *mmpS2* | PROBABLE CONSERVED MEMBRANE PROTEIN MMPS2 | 145 |  | SP, TM | CF 8, MEM 15, MEM 8, MEM 14, CW 14 | BlaTEM 18 |  |
| Rv0507 | *mmpL2* | PROBABLE CONSERVED TRANSMEMBRANE TRANSPORT PROTEIN MMPL2 | 188, 410 |  | TM | CF 8, CW 15, CW 14 |  | *in vitro* 22 |
| Rv0517 |  | POSSIBLE MEMBRANE ACYLTRANSFERASE | 152, 248, 294, 299 |  | TM | MEM 14, CW 14 | BlaTEM 18 |  |
| Rv0518 |  | POSSIBLE EXPORTED PROTEIN | 48, 53, 61, 85, 90, 101 | Yes |  | CF 8, CF 9, MEM 8 |  |  |
| Rv0522 | *gabP* | PROBABLE GABA PERMEASE GABP (4-AMINO BUTYRATE TRANSPORT CARRIER) (GAMA-AMINOBUTYRATE PERMEASE) | 149, 251, 271, 400 |  | TM |  |  |  |
| Rv0526 |  | POSSIBLE THIOREDOXIN PROTEIN (THIOL-DISULFIDE INTERCHANGE PROTEIN) | 31, 39, 47, 48 | Yes | SP, Tat SP, Lipo | CF 8, CF 9, MEM 4, MEM 11, MEM 15, MEM 8, MEM 14, CW 14 |  | *in vitro* 25, *in vitro* 22 |
| Rv0528 |  | PROBABLE CONSERVED TRANSMEMBRANE PROTEIN | 34, 42, 190 |  | SP, TM | MEM 14, CW 14 |  | *in vitro* 25, *in vitro* 22 |
| Rv0529 | *ccsA, ccsB* | POSSIBLE CYTOCHROME C-TYPE BIOGENESIS PROTEIN CCSA | 39, 41, 50, 78, 151, 159, 188 | Yes | TM | MEM 14, CW 14 |  | *in vitro* 25, *in vitro* 22 |
| Rv0534c | *menA* | 1,4-DIHYDROXY-2-NAPHTHOATE OCTAPRENYLTRANSFERASE MENA (DHNA-OCTAPRENYLTRANSFERASE) | 65, 102, 123, 243, 251, 256 | Yes | TM | MEM 14, CW 14 |  | *in vitro* 22 |
| Rv0537c |  | PROBABLE INTEGRAL MEMBRANE PROTEIN | 65, 98, 104, 116, 158, 177, 266 | Yes | Tat SP, TM | MEM 8, MEM 14, MEM 9, CW 19, CW 14, WCL 9 |  |  |
| Rv0541c |  | PROBABLE CONSERVED INTEGRAL MEMBRANE PROTEIN | 45, 47, 111 |  | TM |  |  | Mouse 24, *in vitro* 25, *in vitro* 22 |
| Rv0545c | *pitA* | PROBABLE LOW-AFFINITY INORGANIC PHOSPHATE TRANSPORTER INTEGRAL MEMBRANE PROTEIN PITA | 26, 36, 128, 161, 170, 174 | Yes | TM | MEM 14 |  | Mouse 24 |
| Rv0559c |  | POSSIBLE CONSERVED SECRETED PROTEIN | 79 |  | SP, | CF 8, CF 9, MEM 15, MEM 8, CW 15, CW 19 |  |  |
| Rv0583c | *lpqN* | PROBABLE CONSERVED LIPOPROTEIN LPQN | 79, 104, 111, 128, 133, 141, 148, 215 | Yes | SP, Lipo | CF 8, CF 9, MEM 4, MEM 11, MEM 15, MEM 8, MEM 14, MEM 9, CW 15, CW 19, CW 14, WCL 9, SOL 9 | BlaTEM 18 |  |
| Rv0585c |  | PROBABLE CONSERVED INTEGRAL MEMBRANE PROTEIN | 622, 642, 713 |  | TM | MEM 8 |  |  |
| Rv0587 | *yrbE2A* | CONSERVED HYPOTHETICAL INTEGRAL MEMBRANE PROTEIN YRBE2A | 88, 90 |  | TM |  |  |  |
| Rv0588 | *yrbE2B* | CONSERVED HYPOTHETICAL INTEGRAL MEMBRANE PROTEIN YRBE2B | 114 |  | TM |  |  | Mouse 24, *in vitro* 25 |
| Rv0590 | *mce2B* | MCE-FAMILY PROTEIN MCE2B | 36, 39, 50 | Yes | TM | MEM 14 |  | Mouse 24 |
| Rv0593 | *lprL, mce2E* | POSSIBLE MCE-FAMILY LIPOPROTEIN LPRL (MCE-FAMILY LIPOPROTEIN MCE2E) | 261 |  | Lipo, TM |  |  |  |
| Rv0594 | *mce2F* | MCE-FAMILY PROTEIN MCE2F | 48, 141 |  | TM | MEM 14 | BlaTEM 18 |  |
| Rv0601c |  | PROBABLE TWO COMPONENT SENSOR KINASE [FIRST PART] | 18, 29, 36 | Yes | TM |  |  |  |
| Rv0603 |  | POSSIBLE EXPORTED PROTEIN | 20, 35, 44, 54, 55, 62, 66, 101 |  | SP, TM | CF 9 |  |  |
| Rv0604 | *lpqO* | PROBABLE CONSERVED LIPOPROTEIN LPQO | 20, 34, 59, 88, 128, 129, 134, 153, 177, 182, 191, 201, 206, 267 | Yes | SP, Lipo | MEM 4, MEM 8, MEM 14, CW 14 |  |  |
| Rv0615 |  | PROBABLE INTEGRAL MEMBRANE PROTEIN | 47, 63 | Yes | TM |  |  |  |
| Rv0621 |  | POSSIBLE MEMBRANE PROTEIN | 108, 112, 260 | Yes | TM | MEM 14 |  |  |
| Rv0622 |  | POSSIBLE MEMBRANE PROTEIN | 144, 164 |  | TM | CW 15 |  | Mouse 24 |
| Rv0625c |  | PROBABLE CONSERVED TRANSMEMBRANE PROTEIN | 50 | Yes | TM | MEM 8, CW 14 |  |  |
| Rv0658c |  | PROBABLE CONSERVED INTEGRAL MEMBRANE PROTEIN | 38, 45, 53, 59, 60, 109, 118 | Yes | TM |  |  |  |
| Rv0671 | *lpqP* | POSSIBLE CONSERVED LIPOPROTEIN LPQP | 41, 65, 104, 111, 203, 258 |  | SP, Lipo, TM | CW 14 |  | Macaque 19, Mouse 24 |
| Rv0676c | *mmpL5* | PROBABLE CONSERVED TRANSMEMBRANE TRANSPORT PROTEIN MMPL5 | 179, 243, 272, 421, 432, 758 |  | TM | MEM 11, MEM 8, MEM 14, CW 14 | PhoA 17, BlaTEM 18 |  |
| Rv0677c | *mmpS5* | POSSIBLE CONSERVED MEMBRANE PROTEIN MMPS5 | 20, 60, 62, 140 | Yes | TM | CF 8, CF 9, MEM 15, MEM 8, MEM 14, CW 14 | BlaTEM 18 | Macrophage 20 |
| Rv0679c |  | CONSERVED HYPOTHETICAL THREONINE RICH PROTEIN | 45, 47, 58, 65, 70, 87 | Yes | SP, Lipo | CF 9, MEM 8, MEM 14, CW 14 |  |  |
| Rv0680c |  | PROBABLE CONSERVED TRANSMEMBRANE PROTEIN | 21, 37 |  | SP, TM | CF 8, MEM 15, MEM 8, CW 14 |  |  |
| Rv0713 |  | PROBABLE CONSERVED TRANSMEMBRANE PROTEIN | 136, 178 |  | TM | MEM 14, CW 14 |  |  |
| Rv0732 | *secY* | PROBABLE PREPROTEIN TRANSLOCASE SECY | 31, 36, 55, 66, 67, 85, 140, 212, 295, 398 | Yes | TM | MEM 15, MEM 8, MEM 14, MEM 9, CW 19, CW 14, WCL 9 |  | *in vitro* 25, *in vitro* 22 |
| Rv0774c |  | PROBABLE CONSERVED EXPORTED PROTEIN | 40 |  | SP, Tat SP, TM | CF 8, MEM 15 | BlaC 2 |  |
| Rv0779c |  | POSSIBLE CONSERVED TRANSMEMBRANE PROTEIN | 48, 60, 65, 85, 95, 97, 105 | Yes | TM | MEM 14, CW 14 |  |  |
| Rv0783c | *emrB* | POSSIBLE MULTIDRUG RESISTANCE INTEGRAL MEMBRANE EFFLUX PROTEIN EMRB | 352, 395, 520, 538 | Yes | TM | MEM 14 | BlaTEM 18 |  |
| Rv0787 |  | HYPOTHETICAL PROTEIN | 136 |  |  | CF 8, CF 9, MEM 15, MEM 14 |  |  |
| Rv0817c |  | PROBABLE CONSERVED EXPORTED PROTEIN | 83, 140, 151 |  | SP, TM | MEM 8, MEM 14, CW 14 |  | *in vitro* 25, *in vitro* 22 |
| Rv0822c |  | CONSERVED HYPOTHETICAL PROTEIN | 188, 218, 235, 243, 250, 471, 565 | Yes |  | CF 8, CW 14 |  |  |
| Rv0835 | *lpqQ* | POSSIBLE LIPOPROTEIN LPQQ | 33, 60 |  | SP, | CF 8, MEM 15, MEM 14 |  | Mouse 24 |
| Rv0838 | *lpqR* | PROBABLE CONSERVED LIPOPROTEIN LPQR | 50, 63, 69, 75, 88, 95, 241, 243 | Yes | SP, Lipo | CF 8, MEM 15, MEM 8 |  |  |
| Rv0846c | *mmcO* | PROBABLE OXIDASE | 41, 43, 44, 47 |  | SP, Tat SP, Lipo | MEM 8, MEM 14, CW 14 | BlaC 2 |  |
| Rv0870c |  | POSSIBLE CONSERVED INTEGRAL MEMBRANE PROTEIN | 60, 61, 63, 88 |  | TM | MEM 8, MEM 14, CW 14 |  |  |
| Rv0875c |  | POSSIBLE CONSERVED EXPORTED PROTEIN | 19, 21, 28, 101 |  | SP, TM | MEM 4, MEM 14, CW 14 |  | Mouse 24, *in vitro* 25, *in vitro* 22 |
| Rv0879c |  | POSSIBLE CONSERVED TRANSMEMBRANE PROTEIN | 53, 70 |  | TM | MEM 8, MEM 14 |  |  |
| Rv0888 | *spmT* | PROBABLE EXPORTED PROTEIN | 30, 53, 75, 143, 146 |  | SP, TM | CF 8, MEM 8, MEM 14, CW 14 |  | Macrophage 21 |
| Rv0892 |  | PROBABLE MONOOXYGENASE | 41 |  | TM | MEM 14, CW 15, CW 14 |  |  |
| Rv0899 | *ompA* | OUTER MEMBRANE PROTEIN A OMPA | 56, 59, 86, 100, 142, 287 | Yes | TM | CF 8, CF 9, MEM 11, MEM 8, MEM 14, MEM 9, CW 15, CW 14 |  |  |
| Rv0902c | *prrB* | TWO COMPONENT SENSOR HISTIDINE KINASE PRRB | 110 |  | SP, TM | MEM 11, MEM 14, MEM 9, CW 15, CW 19, CW 14, WCL 9 |  | *in vitro* 22 |
| Rv0907 |  | CONSERVED HYPOTHETICAL PROTEIN | 24, 35, 51, 73, 76, 85 | Yes |  | CF 8, CF 9, MEM 11, MEM 8, MEM 14, CW 14 |  | Mouse 24, *in vitro* 22 |
| Rv0912 |  | PROBABLE CONSERVED TRANSMEMBRANE PROTEIN | 31, 118, 119, 120, 135 | Yes | TM |  |  |  |
| Rv0917 | *betP* | POSSIBLE GLYCINE BETAINE TRANSPORT INTEGRAL MEMBRANE PROTEIN BETP | 53, 228, 315 |  | TM |  | BlaTEM 18 |  |
| Rv0924c | *mntH, Nramp, Mramp* | DIVALENT CATION-TRANSPORT INTEGRAL MEMBRANE PROTEIN MNTH (BRAMP) (MRAMP) | 183, 275, 426 |  | TM | MEM 14 |  | Macaque 19 |
| Rv0928 | *pstS3, phoS2* | PERIPLASMIC PHOSPHATE-BINDING LIPOPROTEIN PSTS3 (PBP-3) (PSTS3) (PHOS1) | 20, 29, 44, 74, 118, 165, 202, 280, 285, 304 | Yes | SP, Lipo | CF 8, CF 9, MEM 11, MEM 15, MEM 8, MEM 14, MEM 9, CW 15, CW 19, CW 14, WCL 9 |  | Macrophage 20, Mouse 24 |
| Rv0931c | *pknD, mbk* | TRANSMEMBRANE SERINE/THREONINE-PROTEIN KINASE D PKND (PROTEIN KINASE D) (STPK D) | 411, 419, 593, 653 |  | TM | MEM 4, MEM 11, MEM 8, MEM 14, MEM 9, CW 15, CW 19, CW 14, WCL 9, SOL 9 | BlaTEM 18 |  |
| Rv0932c | *pstS2* | PERIPLASMIC PHOSPHATE-BINDING LIPOPROTEIN PSTS2 (PBP-2) (PSTS2) | 22, 34, 98, 151, 172, 190, 280, 326 | Yes | SP, Lipo | CF 8, CF 9, MEM 4, MEM 11, MEM 15, MEM 8, MEM 14, MEM 9, CW 19, CW 14, WCL 9, SOL 9 |  |  |
| Rv0934 | *pstS1, phoS1, phoS* | PERIPLASMIC PHOSPHATE-BINDING LIPOPROTEIN PSTS1 (PBP-1) (PSTS1) | 29, 36, 38, 45, 55, 170, 188, 210, 235 | Yes | SP, Lipo | CF 7, CF 8, CF 9, MEM 4, MEM 11, MEM 15, MEM 8, MEM 14, MEM 9, CW 15, CW 19, CW 14, WCL 9, SOL 9 | PhoA 17, BlaTEM 18 |  |
| Rv0935 | *pstC1* | PHOSPHATE-TRANSPORT INTEGRAL MEMBRANE ABC TRANSPORTER PSTC1 | 172, 184 |  | TM | MEM 8, MEM 14, MEM 9 |  |  |
| Rv0936 | *pstA2* | PHOSPHATE-TRANSPORT INTEGRAL MEMBRANE ABC TRANSPORTER PSTA2 | 239 | Yes | TM | MEM 8, MEM 14, CW 14 |  |  |
| Rv0950c |  | CONSERVED HYPOTHETICAL PROTEIN | 143, 222 | Yes |  |  |  | Mouse 22, Mouse 24 |
| Rv0954 |  | PROBABLE CONSERVED TRANSMEMBRANE PROTEIN | 73, 90, 129, 159 |  | TM | MEM 11, MEM 8, MEM 14, MEM 9, CW 15, CW 19, WCL 9 |  | Macaque 19 |
| Rv0961 |  | PROBABLE INTEGRAL MEMBRANE PROTEIN | 31 |  | TM |  |  |  |
| Rv0962c | *lprP* | POSSIBLE LIPOPROTEIN LPRP | 33 |  | SP, Lipo |  |  |  |
| Rv0969 | *ctpV* | PROBABLE METAL CATION TRANSPORTER P-TYPE ATPASE CTPV | 169 |  | TM | MEM 4, MEM 11, MEM 8, MEM 14, MEM 9, CW 15, CW 19, CW 14, WCL 9, SOL 9 |  |  |
| Rv0982 | *mprB* | PROBABLE TWO COMPONENT SENSOR KINASE MPRB | 48, 82, 94, 100, 186 | Yes | SP, TM | MEM 15, MEM 14, CW 14 |  | *in vitro* 25, *in vitro* 22 |
| Rv0983 | *pepD, mtb32b* | PROBABLE SERINE PROTEASE PEPD (SERINE PROTEINASE) (MTB32B) | 116, 119, 130, 146, 245, 292, 313, 316, 370, 398, 402 | Yes | TM | CF 8, CF 9, MEM 8, MEM 14, MEM 9, CW 19, CW 14, WCL 9, SOL 9 | BlaTEM 18 | Mouse 24 |
| Rv0987 |  | PROBABLE ADHESION COMPONENT TRANSPORT TRANSMEMBRANE PROTEIN ABC TRANSPORTER | 58, 62, 73, 92, 533, 537 |  | TM | MEM 12, MEM 14, CW 14 |  | *in vitro* 22 |
| Rv0988 |  | POSSIBLE CONSERVED EXPORTED PROTEIN | 45 | Yes | SP, TM | CF 8, MEM 8, MEM 14, CW 14 |  | Mouse 24, *in vitro* 22 |
| Rv0999 |  | HYPOTHETICAL PROTEIN | 57, 60, 89, 93, 169, 201, 208 | Yes | SP, | CF 8, CF 9, MEM 4, MEM 11, MEM 15, MEM 8, MEM 14, CW 14 |  |  |
| Rv1002c |  | CONSERVED MEMBRANE PROTEIN | 75, 149, 285, 328, 483 |  | TM | MEM 8, MEM 14, CW 14 |  | *in vitro* 25, *in vitro* 22 |
| Rv1004c |  | PROBABLE MEMBRANE PROTEIN | 44, 49, 64, 71, 113, 119, 122, 168, 172, 192, 211, 218, 230, 237, 245, 287, 408 | Yes | SP, TM | MEM 8 | BlaTEM 18 |  |
| Rv1006 |  | HYPOTHETICAL PROTEIN | 33, 56, 72, 81, 128, 219, 263, 286, 305, 361, 400, 504 | Yes | SP, | MEM 4, MEM 11, MEM 8, MEM 14, MEM 9, CW 15, CW 19, CW 14, WCL 9 | PhoA 17 |  |
| Rv1009 | *rpfB* | Probable resuscitation-promoting factor rpfB | 15, 21, 33, 63, 65, 98, 117, 128, 145, 165, 177, 247, 271, 288, 292, 300 | Yes | SP, Lipo, TM |  | BlaTEM 18 | *in vitro* growth-defect 28 |
| Rv1016c | *lpqT* | PROBABLE CONSERVED LIPOPROTEIN LPQT | 69 |  | SP, Lipo | CF 8, CF 9, MEM 11, MEM 8, MEM 14, MEM 9, CW 15, CW 14 |  | Macrophage 20, Mouse 22 |
| Rv1022 | *lpqU* | PROBABLE CONSERVED LIPOPROTEIN LPQU | 39, 51, 54 | Yes | SP, TM | MEM 8, MEM 14, CW 14 |  |  |
| Rv1024 |  | POSSIBLE CONSERVED MEMBRANE PROTEIN | 187, 209, 225 | Yes | Tat SP | MEM 8, MEM 14, CW 14 |  | *in vitro* 25, *in vitro* 22 |
| Rv1026 |  | CONSERVED HYPOTHETICAL PROTEIN | 245 |  |  |  |  | *in vitro* 25 |
| Rv1029 | *kdpA* | Probable Potassium-transporting ATPase A chain KDPA (Potassium-translocating ATPase A chain) (ATP phosphohydrolase [potassium-transporting] A chain) (Potassium binding and translocating subunit A) | 37, 220, 236, 244, 327, 369, 443, 451, 483 | Yes | TM | CW 15 |  |  |
| Rv1030 | *kdpB* | Probable Potassium-transporting P-type ATPase B chain KDPB (Potassium-translocating ATPase B chain) (ATP phosphohydrolase [potassium-transporting] B chain) (Potassium binding and translocating subunit B) | 88, 262, 263 |  | TM | MEM 8, MEM 14, CW 14 |  |  |
| Rv1031 | *kdpC* | Probable Potassium-transporting ATPase C chain KDPC (Potassium-translocating ATPase C chain) (ATP phosphohydrolase [potassium-transporting] C chain) (Potassium binding and translocating subunit C) | 27, 52, 60 |  | TM | MEM 8, MEM 14, CW 14 |  |  |
| Rv1032c | *trcS* | TWO COMPONENT SENSOR HISTIDINE KINASE TRCS | 51, 82 |  | TM |  |  |  |
| Rv1064c | *lpqV* | POSSIBLE LIPOPROTEIN LPQV | 23, 27, 33, 40, 53, 62 |  | SP, Lipo |  |  |  |
| Rv1072 |  | PROBABLE CONSERVED TRANSMEMBRANE PROTEIN | 137, 200, 210 |  | TM | MEM 8, MEM 14, CW 14 | PhoA 17 | *in vitro* 22 |
| Rv1081c |  | PROBABLE CONSERVED MEMBRANE PROTEIN | 54 | Yes | TM | MEM 14 |  | *in vitro* 22 |
| Rv1085c |  | POSSIBLE HEMOLYSIN-LIKE PROTEIN | 63, 181 | Yes | TM | MEM 14 |  |  |
| Rv1096 |  | POSSIBLE GLYCOSYL HYDROLASE | 31, 84, 87, 119, 133, 143, 144, 154, 284 |  | TM | CF 8, CF 9, MEM 8, MEM 14, MEM 9, CW 15, CW 19, CW 14 | BlaTEM 18 | Macrophage 20, Mouse 24, *in vitro* growth-defect 28 |
| Rv1097c |  | PROBABLE MEMBRANE GLYCINE AND PROLINE RICH PROTEIN | 114, 207 |  | TM | CF 8, CF 9, MEM 11, MEM 8, MEM 14, MEM 9, CW 15, CW 19, CW 14 |  | Mouse 24 |
| Rv1100 |  | CONSERVED HYPOTHETICAL PROTEIN | 67, 101, 107, 110, 122, 230 | Yes | TM | CF 9, MEM 8, MEM 14, CW 15, CW 14 |  | Mouse 24, *in vitro* growth-defect 28 |
| Rv1111c |  | CONSERVED HYPOTHETICAL PROTEIN | 34, 44, 56 |  | TM | MEM 14, CW 14 |  | Mouse 22, Mouse 24, *in vitro* 22 |
| Rv1132 |  | CONSERVED MEMBRANE PROTEIN | 166, 180, 318 |  | TM | MEM 8, MEM 14, CW 14 |  |  |
| Rv1140 |  | PROBABLE INTEGRAL MEMBRANE PROTEIN | 101, 135 | Yes | TM | MEM 8, MEM 14, CW 14 |  |  |
| Rv1145 | *mmpL13a* | PROBABLE CONSERVED TRANSMEMBRANE TRANSPORT PROTEIN MMPL13A | 39, 63 |  | TM | MEM 14 |  |  |
| Rv1158c |  | CONSERVED HYPOTHETICAL ALA-, PRO-RICH PROTEIN | 23 |  | SP, TM | MEM 15 |  |  |
| Rv1159 | *pimE* | CONSERVED TRANSMEMBRANE PROTEIN | 277, 288 |  | TM |  |  | Mouse 24, *in vitro* 22 |
| Rv1166 | *lpqW* | PROBABLE CONSERVED LIPOPROTEIN LPQW | 25, 63, 78, 99, 112, 160, 161, 174, 187, 211, 251, 297, 485 | Yes | SP, Lipo | CF 8, MEM 15, MEM 8, MEM 14, CW 14 |  | *in vitro* 25, *in vitro* 22 |
| Rv1174c | *TB8.4* | LOW MOLECULAR WEIGHT T-CELL ANTIGEN TB8.4 | 26, 55, 83 | Yes | SP, | CF 8, CF 9, MEM 15, MEM 9, CW 19, WCL 9, SOL 9 | BlaTEM 18 |  |
| Rv1183 | *mmpL10* | PROBABLE CONSERVED TRANSMEMBRANE TRANSPORT PROTEIN MMPL10 | 50, 329, 862, 932 |  | SP, TM | MEM 12, MEM 15, MEM 8, MEM 14, CW 14 |  | Mouse 22 |
| Rv1184c |  | POSSIBLE EXPORTED PROTEIN | 16, 21, 28, 48, 88, 89, 134, 174, 190, 218, 253, 255, 354, 357 |  | SP, TM | MEM 8, CW 15, CW 14 |  | Mouse 22 |
| Rv1200 |  | PROBABLE CONSERVED INTEGRAL MEMBRANE TRANSPORT PROTEIN | 42, 102, 269, 323 |  | TM |  |  | Macaque 19 |
| Rv1217c |  | PROBABLE TETRONASIN-TRANSPORT INTEGRAL MEMBRANE PROTEIN ABC TRANSPORTER | 67, 70, 102, 174, 227, 352, 448, 513, 518 | Yes | TM | MEM 8, MEM 14, CW 14 |  |  |
| Rv1223 | *htrA, degP* | PROBABLE SERINE PROTEASE HTRA (DEGP PROTEIN) | 191, 194, 225, 254, 261, 294, 386, 387, 402, 403, 466, 470 | Yes | TM | CF 8, CF 9, MEM 11, MEM 8, MEM 14, MEM 9, CW 15, CW 19, CW 14, WCL 9 |  | *in vitro* 25, *in vitro* 22 |
| Rv1226c |  | PROBABLE TRANSMEMBRANE PROTEIN | 197, 206, 210 | Yes | TM | MEM 8, MEM 14, CW 14 |  |  |
| Rv1228 | *lpqX* | PROBABLE LIPOPROTEIN LPQX | 37, 56, 68, 74 | Yes | Lipo | CF 8, MEM 14, CW 14 |  |  |
| Rv1230c |  | POSSIBLE MEMBRANE PROTEIN | 53, 69, 74, 118, 130, 270, 271, 275, 305 | Yes | SP, TM | CW 15 | BlaTEM 18 |  |
| Rv1234 |  | PROBABLE TRANSMEMBRANE PROTEIN | 112, 116 |  | TM | MEM 4, MEM 11, MEM 8, MEM 14, MEM 9, CW 19, CW 14, WCL 9 |  | Macaque 19, Mouse 24 |
| Rv1236 | *sugA* | PROBABLE SUGAR-TRANSPORT INTEGRAL MEMBRANE PROTEIN ABC TRANSPORTER SUGA | 146, 246 |  | Tat SP, TM | MEM 8, MEM 14 |  | Macrophage 20, Macrophage 21, Mouse 22, Mouse 24 |
| Rv1237 | *sugB* | PROBABLE SUGAR-TRANSPORT INTEGRAL MEMBRANE PROTEIN ABC TRANSPORTER SUGB | 212, 219, 228, 237 |  | TM | MEM 8, MEM 14, CW 14 |  | Macrophage 20, Mouse 22 |
| Rv1244 | *lpqZ* | PROBABLE LIPOPROTEIN LPQZ | 26, 47, 65, 85, 123, 137, 154, 249, 274, 275 |  | SP, Lipo | MEM 8, MEM 14, CW 15, CW 14 |  | Macrophage 20, Mouse 22 |
| Rv1250 |  | PROBABLE DRUG-TRANSPORT INTEGRAL MEMBRANE PROTEIN | 104, 106, 111, 120, 386, 396, 438, 440, 441 |  | TM |  |  |  |
| Rv1252c | *lprE* | PROBABLE LIPOPROTEIN LPRE | 61, 72, 130, 157, 199, 200 | Yes | SP, Lipo | CF 8, MEM 15, MEM 8, MEM 14, CW 14 |  |  |
| Rv1258c |  | PROBABLE CONSERVED INTEGRAL MEMBRANE TRANSPORT PROTEIN | 372 |  | TM |  |  |  |
| Rv1266c | *pknH* | PROBABLE TRANSMEMBRANE SERINE/THREONINE-PROTEIN KINASE H PKNH (PROTEIN KINASE H) (STPK H) | 603 | Yes | TM | MEM 14, MEM 9, CW 15, CW 19, CW 14, WCL 9 |  |  |
| Rv1269c |  | CONSERVED PROBABLE SECRETED PROTEIN | 29, 34, 44 | Yes | SP, Tat SP | CF 8, CF 9, MEM 15, MEM 8, MEM 14, MEM 9, CW 15, CW 19, CW 14, WCL 9 | PhoA 16 |  |
| Rv1270c | *lprA* | POSSIBLE LIPOPROTEIN LPRA | 22, 30, 36, 60, 93, 123, 161, 238 | Yes | SP, Lipo, TM | CF 8, CF 9, MEM 4, MEM 11, MEM 15, MEM 8, MEM 14, MEM 9, CW 15, CW 19, CW 14, WCL 9, SOL 9 |  |  |
| Rv1271c |  | CONSERVED HYPOTHETICAL SECRETED PROTEIN | 28, 30 |  | SP, TM | CF 9 |  |  |
| Rv1273c |  | PROBABLE DRUGS-TRANSPORT TRANSMEMBRANE ATP-BINDING PROTEIN ABC TRANSPORTER | 38, 50, 64 |  | SP, TM | MEM 8, MEM 14, CW 15 |  | Macrophage 20, Mouse 24 |
| Rv1274 | *lprB* | POSSIBLE LIPOPROTEIN LPRB | 27, 55, 69, 74, 86, 127 | Yes | SP, Lipo | MEM 8, MEM 14, MEM 9, CW 19, WCL 9 |  | *in vitro* 25 |
| Rv1275 | *lprC* | POSSIBLE LIPOPROTEIN LPRC | 18, 27 | Yes | SP, Lipo | CF 8, MEM 4, MEM 8, MEM 14, MEM 9, CW 15, CW 19, CW 14, WCL 9 |  |  |
| Rv1280c | *oppA* | PROBABLE PERIPLASMIC OLIGOPEPTIDE-BINDING LIPOPROTEIN OPPA | 49, 54, 81, 252, 267, 400, 469 | Yes | Tat SP, Lipo, TM | MEM 11, MEM 8, MEM 14, MEM 9, CW 14 |  |  |
| Rv1283c | *oppB* | PROBABLE OLIGOPEPTIDE-TRANSPORT INTEGRAL MEMBRANE PROTEIN ABC TRANSPORTER OPPB | 82, 111, 169, 174, 177, 178, 258, 262, 282 | Yes | TM | MEM 14, CW 14 |  |  |
| Rv1290c |  | CONSERVED HYPOTHETICAL PROTEIN | 94, 184, 194 | Yes | Tat SP, TM | MEM 14, CW 14 |  |  |
| Rv1302 | *rfe, wecA* | PROBABLE UNDECAPAPRENYL-PHOSPHATE ALPHA-N-ACETYLGLUCOSAMINYLTRANSFERASE RFE (UDP-GlcNAc TRANSFERASE) | 106, 109, 114, 237, 360, 371, 382 | Yes | TM | MEM 8, MEM 14 |  | *in vitro* 22 |
| Rv1304 | *atpB* | PROBABLE ATP SYNTHASE A CHAIN ATPB (PROTEIN 6) | 145 |  | TM | MEM 4, MEM 11, MEM 8, MEM 14, MEM 9, CW 19, WCL 9 |  | Mouse 22, *in vitro* 22 |
| Rv1320c |  | POSSIBLE ADENYLATE CYCLASE (ATP PYROPHOSPHATE-LYASE) (ADENYLYL CYCLASE) | 151, 237 | Yes | TM | MEM 14, CW 14 |  |  |
| Rv1348 |  | PROBABLE DRUGS-TRANSPORT TRANSMEMBRANE ATP-BINDING PROTEIN ABC TRANSPORTER | 322, 342 | Yes | TM | MEM 4, MEM 14 |  | Mouse 24, *in vitro* 25, *in vitro* 22 |
| Rv1352 |  | CONSERVED HYPOTHETICAL PROTEIN | 31, 36 |  | SP, TM | CF 8, CF 9, MEM 15 |  |  |
| Rv1362c |  | POSSIBLE MEMBRANE PROTEIN | 95, 97, 103, 126, 132, 159 |  | TM | MEM 4, MEM 14, CW 14 |  |  |
| Rv1363c |  | POSSIBLE MEMBRANE PROTEIN | 119, 122, 139, 144, 173, 214, 228 | Yes | TM | MEM 8, MEM 14, CW 14 |  |  |
| Rv1368 | *lprF* | PROBABLE CONSERVED LIPOPROTEIN LPRF | 38, 50, 136, 226 | Yes | SP, Lipo, TM | CF 8, CF 9, MEM 4, MEM 11, MEM 8, MEM 14, MEM 9, CW 15, CW 19, CW 14 | BlaTEM 18 |  |
| Rv1411c | *lprG, P27* | PROBABLE CONSERVED LIPOPROTEIN LPRG | 24, 33, 168, 198 |  | SP, Lipo | CF 8, CF 9, MEM 4, MEM 11, MEM 8, MEM 14, MEM 9, CW 15, CW 19, CW 14, WCL 9, SOL 9 |  | Macrophage 20, Macrophage 21, Mouse 22, Mouse 24 |
| Rv1418 | *lprH* | PROBABLE LIPOPROTEIN LPRH | 43, 48, 208 | Yes | SP, Lipo, TM | MEM 8, MEM 14, CW 14 |  |  |
| Rv1419 |  | HYPOTHETICAL PROTEIN | 85, 130 |  | SP, | CF 8, CF 9, MEM 15 | BlaTEM 18 |  |
| Rv1424c |  | POSSIBLE MEMBRANE PROTEIN | 65, 67, 191, 217, 223 | Yes | SP, | MEM 8, MEM 14, CW 15, CW 14 |  |  |
| Rv1431 |  | CONSERVED MEMBRANE PROTEIN | 65, 164, 175, 177 | Yes | TM | MEM 8, MEM 14, CW 14 |  |  |
| Rv1433 |  | POSSIBLE CONSERVED EXPORTED PROTEIN | 44, 84, 124 |  | SP, TM |  |  | Macaque 19 |
| Rv1435c |  | Probable conserved Proline, Glycine, Valine-rich secreted protein | 71, 117, 167, 180 | Yes | SP, | CF 8, MEM 15 | BlaTEM 18 |  |
| Rv1451 | *ctaB* | PROBABLE CYTOCHROME C OXIDASE ASSEMBLY FACTOR CTAB | 47, 61, 65, 67, 87, 271 | Yes | TM | MEM 14, CW 15 |  | *in vitro* 22 |
| Rv1456c |  | PROBABLE UNIDENTIFIED ANTIBIOTIC-TRANSPORT INTEGRAL MEMBRANE ABC TRANSPORTER | 30, 37, 261, 270, 282, 291 | Yes | TM | MEM 14 |  | *in vitro* 25, *in vitro* 22 |
| Rv1457c |  | PROBABLE UNIDENTIFIED ANTIBIOTIC-TRANSPORT INTEGRAL MEMBRANE ABC TRANSPORTER | 71, 163 |  | TM |  |  | *in vitro* 25, *in vitro* 22 |
| Rv1459c |  | POSSIBLE CONSERVED INTEGRAL MEMBRANE PROTEIN | 158, 190, 388, 400, 418, 520 |  | TM | MEM 8, MEM 14, CW 14 |  | *in vitro* 25, *in vitro* 22 |
| Rv1477 |  | HYPOTHETICAL INVASION PROTEIN | 46, 279, 360, 373 | Yes | SP, | CF 8, CF 9, MEM 15, MEM 14, CW 19, CW 14 |  | *in vitro* 25, *in vitro* 22 |
| Rv1478 |  | HYPOTHETICAL INVASION PROTEIN | 27, 32, 48 | Yes | SP, |  |  |  |
| Rv1487 |  | CONSERVED MEMBRANE PROTEIN | 20, 29, 34 | Yes | TM | MEM 8, MEM 14, CW 14 |  |  |
| Rv1490 |  | PROBABLE MEMBRANE PROTEIN | 160, 342, 426 |  | TM |  |  | Mouse 24, *in vitro* 25, *in vitro* 22 |
| Rv1491c |  | CONSERVED MEMBRANE PROTEIN | 92, 110, 123, 202, 204 | Yes | TM |  |  |  |
| Rv1508c |  | Probable membrane protein | 232 |  | TM | MEM 8, MEM 14, MEM 9, CW 19, CW 14, WCL 9 |  |  |
| Rv1510 |  | conserved probable membrane protein | 205, 272, 349 |  | TM |  |  |  |
| Rv1517 |  | CONSERVED HYPOTHETICAL TRANSMEMBRANE PROTEIN | 69 |  | Tat SP, TM |  |  |  |
| Rv1522c | *mmpL12* | PROBABLE CONSERVED TRANSMEMBRANE TRANSPORT PROTEIN MMPL12 | 48, 449, 517, 953 | Yes | SP, TM | MEM 14, CW 15 |  | *in vitro* 22 |
| Rv1539 | *lspA* | PROBABLE LIPOPROTEIN SIGNAL PEPTIDASE LSPA | 59, 86 | Yes | TM | MEM 14 |  | *in vitro* 25, *in vitro* 22 |
| Rv1541c | *lprI* | Possible lipoprotein lprI | 27, 28, 60 | Yes | SP, Lipo | MEM 15 |  |  |
| Rv1554 | *frdC* | PROBABLE FUMARATE REDUCTASE [MEMBRANE ANCHOR SUBUNIT] FRDC (FUMARATE DEHYDROGENASE) (FUMARIC HYDROGENASE) | 48 |  | TM |  |  |  |
| Rv1565c |  | CONSERVED HYPOTHETICAL MEMBRANE PROTEIN | 71, 151, 226, 263, 440 |  | TM | MEM 14 |  | Mouse 24, *in vitro* 22 |
| Rv1566c |  | Possible inv protein | 22, 24, 48, 60, 69, 71, 83, 93 |  | SP, TM | MEM 8, CW 14 | PhoA 16, PhoA 17 | Mouse 24 |
| Rv1607 | *chaA* | Probable ionic transporter integral membrane protein chaA | 83, 84, 166, 168, 244 | Yes | TM | MEM 14 |  |  |
| Rv1610 |  | POSSIBLE CONSERVED MEMBRANE PROTEIN | 57, 117 |  | TM | MEM 8, MEM 14, CW 14 |  | *in vitro* 25 |
| Rv1616 |  | CONSERVED MEMBRANE PROTEIN | 51 |  | TM |  |  |  |
| Rv1619 |  | CONSERVED MEMBRANE PROTEIN | 65, 134 |  | TM | MEM 14 |  |  |
| Rv1622c | *cydB* | Probable integral membrane cytochrome D ubiquinol oxidase (subunit II) cydB (Cytochrome BD-I oxidase subunit II) | 172 |  | TM | MEM 8, MEM 14 |  | Mouse 24, *in vitro* 25, *in vitro* 22 |
| Rv1623c | *cydA, appC* | Probable integral membrane cytochrome D ubiquinol oxidase (subunit I) cydA (Cytochrome BD-I oxidase subunit I) | 44, 67, 138, 161, 177, 242, 389, 408, 425 | Yes | TM | MEM 8, MEM 14, CW 15, CW 14 |  | Mouse 24, *in vitro* 22 |
| Rv1625c | *cya* | MEMBRANE-ANCHORED ADENYLYL CYCLASE CYA (ATP PYROPHOSPHATE-LYASE) (ADENYLATE CYCLASE) | 70, 82, 132, 188 |  | TM | MEM 14, CW 14 |  |  |
| Rv1635c |  | Probable conserved transmembrane protein | 242, 397, 432, 446 |  | TM |  | BlaTEM 18 |  |
| Rv1639c |  | CONSERVED HYPOTHETICAL MEMBRANE PROTEIN | 77, 146, 153, 227, 239, 265, 338, 379, 422, 484 | Yes | TM |  |  |  |
| Rv1640c | *lysX* | Possible lysyl-tRNA synthetase 2 lysX | 162, 168, 171, 262 |  | TM | MEM 12, MEM 14, CW 15, CW 14 |  | Macrophage 20, Mouse 22 |
| Rv1648 |  | Probable transmembrane protein | 113 |  | SP, TM | CW 14 | BlaTEM 18 |  |
| Rv1672c |  | PROBABLE CONSERVED INTEGRAL MEMBRANE TRANSPORT PROTEIN | 53, 57, 59, 72, 125, 172, 175, 271, 275, 386, 393, 404 | Yes | TM | MEM 8 |  |  |
| Rv1677 | *dsbF* | PROBABLE CONSERVED LIPOPROTEIN DSBF | 32, 36, 48, 96 |  | SP, Lipo | CF 9, MEM 14, MEM 9 |  |  |
| Rv1678 |  | PROBABLE INTEGRAL MEMBRANE PROTEIN | 202, 220, 235, 256, 270, 275 |  | TM | MEM 14, CW 14 |  |  |
| Rv1686c |  | PROBABLE CONSERVED INTEGRAL MEMBRANE PROTEIN ABC TRANSPORTER | 23, 27, 31 | Yes | TM | MEM 8, MEM 14 |  |  |
| Rv1704c | *cycA* | PROBABLE D-SERINE/ALANINE/GLYCINE TRANSPORTER PROTEIN CYCA | 120, 436, 438 |  | TM | MEM 14 |  | Macaque 19 |
| Rv1707 |  | PROBABLE CONSERVED TRANSMEMBRANE PROTEIN | 28, 30, 46 |  | TM | MEM 8, MEM 14, CW 14 | BlaTEM 18 |  |
| Rv1728c |  | CONSERVED HYPOTHETICAL PROTEIN | 183, 234, 239 | Yes |  |  | BlaTEM 18 | Macrophage 21 |
| Rv1733c |  | PROBABLE CONSERVED TRANSMEMBRANE PROTEIN | 59, 62, 64, 83, 85, 100, 146 |  | SP, TM |  |  |  |
| Rv1736c | *narX* | PROBABLE NITRATE REDUCTASE NARX | 492, 587 |  | TM | MEM 14, CW 14 |  |  |
| Rv1737c | *narK2* | POSSIBLE NITRATE/NITRITE TRANSPORTER NARK2 | 247, 248 |  | TM | MEM 8, MEM 14 |  | Mouse 24 |
| Rv1739c |  | PROBABLE SULPHATE-TRANSPORT TRANSMEMBRANE PROTEIN ABC TRANSPORTER | 158, 238, 261 |  | TM |  |  |  |
| Rv1743 | *pknE* | PROBABLE TRANSMEMBRANE SERINE/THREONINE-PROTEIN KINASE E PKNE (PROTEIN KINASE E) (STPK E) |  |  | TM | CF 9, MEM 14, MEM 9, CW 19, CW 14, WCL 9, SOL 9 | BlaTEM 18 |  |
| Rv1747 |  | PROBABLE CONSERVED TRANSMEMBRANE ATP-BINDING PROTEIN ABC TRANSPORTER | 722, 781, 820 |  | TM | MEM 8, MEM 14, MEM 9, CW 15, CW 19, CW 14, WCL 9, SOL 9 |  |  |
| Rv1749c |  | POSSIBLE INTEGRAL MEMBRANE PROTEIN | 86, 89, 113 | Yes | TM | MEM 8, MEM 14, MEM 9, CW 19, WCL 9 |  |  |
| Rv1754c |  | CONSERVED HYPOTHETICAL PROTEIN | 95, 98, 129, 301, 394 | Yes | TM | MEM 8, MEM 14, CW 14 |  |  |
| Rv1779c |  | HYPOTHETICAL INTEGRAL MEMBRANE PROTEIN | 128, 174, 200, 278 | Yes | TM | MEM 8, MEM 14 | BlaTEM 18 |  |
| Rv1782 | *eccB5* | PROBABLE CONSERVED MEMBRANE PROTEIN | 92, 116, 135, 154, 380 | Yes | TM | CF 8, MEM 4, MEM 8, MEM 14, CW 15, CW 14 |  | *in vitro* 22 |
| Rv1804c |  | CONSERVED HYPOTHETICAL PROTEIN | 24 |  | SP, | MEM 15 |  | Macaque 19 |
| Rv1810 |  | CONSERVED HYPOTHETICAL PROTEIN | 31, 41, 45, 50, 63, 89 | Yes | SP, TM | CF 8, CF 9, MEM 15 |  | Macaque 19 |
| Rv1811 | *mgtC* | POSSIBLE Mg2+ TRANSPORT P-TYPE ATPASE C MGTC | 75, 117 |  | TM | MEM 14 |  |  |
| Rv1813c |  | CONSERVED HYPOTHETICAL PROTEIN | 47 |  | SP, Tat SP, TM | CF 9, MEM 14, CW 14 |  |  |
| Rv1814 | *erg3* | MEMBRANE-BOUND C-5 STEROL DESATURASE ERG3 (STEROL-C5-DESATURASE) | 86 |  | TM |  |  |  |
| Rv1823 |  | CONSERVED HYPOTHETICAL PROTEIN | 79, 94, 98, 112, 188, 264, 292 |  |  | MEM 8, MEM 14, CW 14 |  | Mouse 24 |
| Rv1824 |  | CONSERVED HYPOTHETICAL MEMBRANE PROTEIN | 29, 45, 96 |  | TM | MEM 14 |  | *in vitro* growth-defect 28 |
| Rv1825 |  | CONSERVED HYPOTHETICAL PROTEIN | 92, 135, 163, 176, 276 | Yes | TM | CF 8, CF 9, MEM 11, MEM 8, MEM 14, MEM 9, CW 14 | PhoA 17 | Mouse 24 |
| Rv1832 | *gcvB* | Probable glycine dehydrogenase gcvB (Glycine decarboxylase) (Glycine cleavage system P-protein) | 257 |  |  | CF 8, MEM 4, MEM 8, MEM 14, WCL 9, SOL 9 |  | *in vitro* 25, *in vitro* 22 |
| Rv1842c |  | CONSERVED HYPOTHETICAL MEMBRANE PROTEIN | 30, 42 |  | TM | MEM 14, CW 14 |  |  |
| Rv1845c |  | CONSERVED HYPOTHETICAL TRANSMEMBRANE PROTEIN | 51, 62, 76 | Yes | SP, TM | MEM 15, MEM 14 |  | Mouse 24, *in vitro* growth-defect 28, *in vitro* 22 |
| Rv1857 | *modA* | PROBABLE MOLYBDATE-BINDING LIPOPROTEIN MODA | 26, 31, 87, 96, 109, 124, 187 | Yes | SP, Lipo | MEM 8 |  |  |
| Rv1860 | *apa, mpt32, modD* | ALANINE AND PROLINE RICH SECRETED PROTEIN APA (FIBRONECTIN ATTACHMENT PROTEIN) (Immunogenic protein MPT32) (Antigen MPT-32) (45-kDa glycoprotein) (45/47 kDa antigen) | 35, 50, 73, 146 | Yes | SP, TM | CF 7, CF 8, CF 9, MEM 15, MEM 8, CW 19, CW 14, WCL 9, SOL 9 | BlaTEM 18 | Mouse 24 |
| Rv1861 |  | PROBABLE CONSERVED TRANSMEMBRANE PROTEIN | 74 |  | TM | MEM 14 |  |  |
| Rv1863c |  | PROBABLE CONSERVED INTEGRAL MEMBRANE PROTEIN | 59, 125, 148 | Yes | TM |  |  |  |
| Rv1877 |  | PROBABLE CONSERVED INTEGRAL MEMBRANE PROTEIN | 128, 134, 234 |  | TM |  |  |  |
| Rv1884c | *rpfC* | PROBABLE RESUSCITATION-PROMOTING FACTOR RPFC | 66, 80, 88, 94, 107, 139, 141, 153 |  | SP, | CF 8, CF 9, MEM 15, MEM 8, MEM 14, WCL 9, SOL 9 |  |  |
| Rv1885c |  | CONSERVED HYPOTHETICAL PROTEIN | 44 |  | SP, TM | CF 8, CF 9, MEM 8, CW 14 |  |  |
| Rv1887 |  | HYPOTHETICAL PROTEIN | 280, 331 |  |  | CF 9, MEM 15 | PhoA 17, BlaTEM 18 |  |
| Rv1891 |  | CONSERVED HYPOTHETICAL PROTEIN | 58, 77, 102 |  | Tat SP | CF 8, CF 9, MEM 15, MEM 8 | BlaTEM 18 |  |
| Rv1902c | *nanT* | PROBABLE SIALIC ACID-TRANSPORT INTEGRAL MEMBRANE PROTEIN NANT | 246, 253, 259 |  | TM |  |  | Macrophage 21 |
| Rv1903 |  | PROBABLE CONSERVED MEMBRANE PROTEIN | 28, 84 | Yes | TM | MEM 14 |  |  |
| Rv1906c |  | CONSERVED HYPOTHETICAL PROTEIN | 29, 62, 123 | Yes | SP, TM | CF 8, CF 9, MEM 15 | PhoA 16 |  |
| Rv1911c | *lppC* | PROBABLE LIPOPROTEIN LPPC | 25, 27, 51, 88, 189 | Yes | Lipo | CF 8, CF 9, MEM 15, MEM 9, CW 15, WCL 9 | PhoA 17 | Mouse 24 |
| Rv1921c | *lppF* | PROBABLE CONSERVED LIPOPROTEIN LPPF | 30 | Yes | SP, Lipo | MEM 14 |  |  |
| Rv1922 |  | PROBABLE CONSERVED LIPOPROTEIN | 25 |  | SP, Lipo | CF 8, MEM 14, CW 14 |  |  |
| Rv1926c | *mpt63, mpb63* | IMMUNOGENIC PROTEIN MPT63 (ANTIGEN MPT63/MPB63) (16 kDa IMMUNOPROTECTIVE EXTRACELLULAR PROTEIN) | 33, 66, 85, 90, 99, 118, 129, 137 | Yes | SP, TM | CF 7, CF 8, CF 9, MEM 15, MEM 8, MEM 14, CW 15, CW 19, CW 14, WCL 9, SOL 9 | PhoA 17 | Mouse 24 |
| Rv1965 | *yrbE3B* | CONSERVED HYPOTHETICAL INTEGRAL MEMBRANE PROTEIN YRBE3B | 90, 104 |  | TM |  |  | Macaque 19 |
| Rv1968 | *mce3C* | MCE-FAMILY PROTEIN MCE3C | 42, 48, 49, 53, 60 | Yes | TM |  |  |  |
| Rv1969 | *mce3D* | MCE-FAMILY PROTEIN MCE3D | 27, 30, 110 |  | SP, TM | CW 15 |  |  |
| Rv1973 |  | POSSIBLE CONSERVED MCE ASSOCIATED MEMBRANE PROTEIN | 35, 51 | Yes | TM |  |  |  |
| Rv1979c |  | POSSIBLE CONSERVED PERMEASE | 374 |  | TM |  |  |  |
| Rv1980c | *mpt64, mpb64* | IMMUNOGENIC PROTEIN MPT64 (ANTIGEN MPT64/MPB64) | 22, 36, 119, 210 |  | SP, TM | CF 7, CF 8, CF 9, MEM 15, MEM 8, CW 15, CW 19, CW 14, WCL 9, SOL 9 |  |  |
| Rv1984c | *cfp21* | PROBABLE CUTINASE PRECURSOR CFP21 | 23, 24, 25, 28, 43, 67, 132, 157, 184, 186, 215 | Yes | SP, | CF 7, CF 8, CF 9, MEM 15, MEM 8, CW 15, CW 19, CW 14 | BlaTEM 18 |  |
| Rv1986 |  | PROBABLE CONSERVED INTEGRAL MEMBRANE PROTEIN | 74, 76 | Yes | TM |  |  |  |
| Rv1987 |  | POSSIBLE CHITINASE | 51, 110, 116, 123 |  | SP, TM | CF 8, MEM 8, MEM 14 |  |  |
| Rv1997 | *ctpF* | PROBABLE METAL CATION TRANSPORTER P-TYPE ATPASE A CTPF | 278, 318 | Yes | TM | MEM 11, MEM 14, MEM 9, CW 19, CW 14 |  |  |
| Rv1999c |  | PROBABLE CONSERVED INTEGRAL MEMBRANE PROTEIN | 172, 399, 400 |  | Tat SP, TM |  |  |  |
| Rv2025c |  | POSSIBLE CONSERVED MEMBRANE PROTEIN | 70, 127 |  | TM |  |  |  |
| Rv2046 | *lppI* | Probable lipoprotein lppI | 20, 34, 45, 65, 103, 128, 142 | Yes | SP, Lipo | MEM 4, MEM 8, MEM 14 |  | Macaque 19 |
| Rv2051c | *ppm1* | Polyprenol-monophosphomannose synthase Ppm1 | 93, 306 | Yes | TM | MEM 4, MEM 8, MEM 14, CW 12, CW 14 |  | Mouse 24, *in vitro* 22 |
| Rv2053c | *fxsA* | PROBABLE TRANSMEMBRANE PROTEIN | 106 |  | TM | MEM 14 |  |  |
| Rv2060 |  | Possible conserved integral membrane protein | 70 |  | SP, TM | MEM 15 |  |  |
| Rv2080 | *lppJ* | Possible lipoprotein lppJ | 37, 47, 69 |  | SP, Lipo, TM | CF 8, MEM 15, MEM 8, MEM 14 | BlaTEM 18 |  |
| Rv2088 | *pknJ* | PROBABLE TRANSMEMBRANE SERINE/THREONINE-PROTEIN KINASE J PKNJ (PROTEIN KINASE J) (STPK J) | 399, 435 |  |  | MEM 8, MEM 14, CW 14 |  |  |
| Rv2091c |  | Probable membrane protein | 161, 176 |  | TM | CF 9, MEM 11, MEM 8, MEM 14, MEM 9, CW 15, CW 19, CW 14, WCL 9 |  | Macaque 19, Mouse 24 |
| Rv2093c | *tatC* | Probable Sec-independent protein translocase transmembrane protein tatC | 110, 241, 249 | Yes | TM | MEM 8 |  | *in vitro* 25, *in vitro* 22 |
| Rv2113 |  | Probable integral membrane protein | 67, 197 | Yes | TM | MEM 11, MEM 8, MEM 14, CW 14 | BlaTEM 18 |  |
| Rv2120c |  | PROBABLE CONSERVED INTEGRAL MEMBRANE PROTEIN | 16, 30, 96, 118 |  | SP, TM | MEM 8, MEM 14, MEM 9 |  |  |
| Rv2127 | *ansP1* | Probable L-asparagine permease ansP1 | 183, 193, 202, 236, 272, 276, 293, 364 |  | TM | MEM 8, MEM 14, CW 14 | BlaTEM 18 | Macrophage 21 |
| Rv2128 |  | PROBABLE CONSERVED TRANSMEMBRANE PROTEIN | 48 | Yes | TM |  |  |  |
| Rv2138 | *lppL* | Probable conserved lipoprotein LppL | 35 |  | SP, Lipo, TM | CF 8, MEM 8, MEM 14, CW 14 |  | Mouse 24, *in vitro* 25, *in vitro* 22 |
| Rv2144c |  | Probable transmembrane protein | 86 |  | TM |  |  |  |
| Rv2151c | *ftsQ* | POSSIBLE CELL DIVISION PROTEIN FTSQ | 144, 230 |  | TM | CF 9, MEM 11, MEM 14, MEM 9, CW 19, CW 14 |  | *in vitro* 25, *in vitro* 22 |
| Rv2154c | *ftsW* | FtsW-like protein FtsW | 149, 273, 388 |  | TM | CW 14 |  | *in vitro* 25, *in vitro* 22 |
| Rv2156c | *murX* | Probable phospho-N-acetylmuramoyl-pentappeptidetransferase MurX | 46, 191, 237 |  | TM | MEM 14, MEM 9, CW 19 |  | *in vitro* 25, *in vitro* 22 |
| Rv2171 | *lppM* | Probable conserved lipoprotein lppM | 22, 40, 45, 104, 105, 143 | Yes | SP, Lipo, TM | CF 8, MEM 8, MEM 14, CW 15, CW 14 |  |  |
| Rv2174 |  | Possible conserved integral membrane protein | 175, 199, 263, 290, 339, 347 |  | TM | MEM 8, MEM 14 |  | *in vitro* 25, *in vitro* 22 |
| Rv2181 |  | Probable conserved integral membrane protein | 64, 285, 401, 417 |  | TM | MEM 8, MEM 14 |  |  |
| Rv2190c |  | CONSERVED HYPOTHETICAL PROTEIN | 27, 107, 174, 229, 238, 258, 269 | Yes | SP, TM | MEM 15, MEM 14, CW 14 |  | *in vitro* 22 |
| Rv2193 | *ctaE* | PROBABLE CYTOCHROME C OXIDASE (SUBUNIT III) CTAE | 53, 143, 151, 152 | Yes | TM | MEM 14 |  | *in vitro* 25, *in vitro* 22 |
| Rv2194 | *qcrC* | Probable Ubiquinol-cytochrome C reductase QcrC(cytochrome C subunit) | 111, 139, 180, 197, 243 | Yes | SP, TM | CF 9, MEM 11, MEM 8, MEM 14, MEM 9, CW 19, CW 14, WCL 9 |  | *in vitro* 25, *in vitro* 22 |
| Rv2198c | *mmpS3* | PROBABLE CONSERVED MEMBRANE PROTEIN MMPS3 | 130, 208, 218, 235 |  | TM | CF 8, CF 9, MEM 11, MEM 8, MEM 14, MEM 9, CW 15, CW 19, CW 14 |  | Mouse 24, *in vitro* growth-defect 28, *in vitro* 22 |
| Rv2199c | *ctaF* | Possible conserved integral membrane protein | 30, 44, 45, 115, 125 | Yes | TM | MEM 9 |  |  |
| Rv2200c | *ctaC* | PROBABLE TRANSMEMBRANE CYTOCHROME C OXIDASE (SUBUNIT II) CTAC | 92, 287, 353 |  | TM | CF 9, MEM 4, MEM 11, MEM 15, MEM 8, MEM 14, MEM 9, CW 19, CW 14, WCL 9 | BlaTEM 18 | *in vitro* 25, *in vitro* 22 |
| Rv2203 |  | POSSIBLE CONSERVED MEMBRANE PROTEIN | 114, 137, 195, 225 | Yes | TM | MEM 8, MEM 14, MEM 9, CW 19, CW 14, WCL 9 | BlaTEM 18 |  |
| Rv2209 |  | Probable conserved integral membrane protein | 110, 187 |  | TM | MEM 14 |  |  |
| Rv2219 |  | PROBABLE CONSERVED TRANSMEMBRANE PROTEIN | 68 |  | TM | MEM 11, MEM 8, MEM 14, MEM 9, CW 19, CW 14 |  | *in vitro* 25, *in vitro* 22 |
| Rv2223c |  | Probable exported protease | 31, 37, 39, 84 | Yes | SP, TM | CF 8, MEM 14, CW 15 | PhoA 16 |  |
| Rv2224c |  | Probable exported protease | 64, 65, 110, 113, 141, 325, 360, 383, 478 | Yes | SP, Lipo, TM | CF 8, CF 9, MEM 4, MEM 11, MEM 15, MEM 8, MEM 14, MEM 9, CW 19, CW 14 |  | Macrophage 20, Mouse 22, Mouse 23, Mouse 24 |
| Rv2235 |  | PROBABLE CONSERVED TRANSMEMBRANE PROTEIN | 80, 112, 145, 198, 210 |  | TM | MEM 8, MEM 14, CW 14 |  | *in vitro* 25, *in vitro* 22 |
| Rv2240c |  | HYPOTHETICAL PROTEIN | 128, 177, 185, 262 | Yes |  | CF 8, CF 9, MEM 8, MEM 14, CW 14 | BlaTEM 18 |  |
| Rv2262c |  | CONSERVED HYPOTHETICAL PROTEIN | 88, 89, 227, 239, 244 |  | Tat SP, TM |  |  |  |
| Rv2264c |  | conserved hypothetical proline rich protein | 497, 535 |  |  | CW 14 | BlaTEM 18 |  |
| Rv2265 |  | Possible conserved integral membrane protein | 180, 188, 189, 191 |  | TM |  |  |  |
| Rv2270 | *lppN* | PROBABLE LIPOPROTEIN LPPN | 27 |  | SP, Lipo |  |  |  |
| Rv2272 |  | PROBABLE CONSERVED TRANSMEMBRANE PROTEIN | 52, 70, 120 |  | TM | MEM 14, CW 14 |  |  |
| Rv2273 |  | PROBABLE CONSERVED TRANSMEMBRANE PROTEIN | 102 |  | TM |  |  |  |
| Rv2281 | *pitB* | Putative phosphate-transport permease PitB | 62, 131, 137, 195 |  | TM | MEM 14 |  |  |
| Rv2284 | *lipM* | Probable esterase LipM | 62, 105 |  | TM | MEM 4, MEM 11, MEM 8, MEM 14, MEM 9, CW 19, CW 14 | BlaTEM 18 |  |
| Rv2287 | *yjcE* | Probable conserved integral membrane transport protein YjcE | 122, 196, 301 | Yes | TM | MEM 14, CW 14 |  |  |
| Rv2289 | *cdh* | Probable CDP-diacylglycerol pyrophosphatase Cdh (CDP-diacylglycerol diphosphatase) (CDP-diacylglycerol phosphatidylhydrolase) | 99, 115, 121 |  | SP, TM | MEM 4, MEM 11, MEM 8, MEM 14, CW 14 | PhoA 17 |  |
| Rv2290 | *lppO* | Probable conserved lipoprotein lppO | 44, 46, 59 |  | SP, Lipo | CF 8, CF 9, MEM 14, CW 14 | PhoA 16, BlaTEM 18 |  |
| Rv2300c |  | CONSERVED HYPOTHETICAL PROTEIN | 210 |  |  | MEM 8, MEM 14 |  |  |
| Rv2301 | *cut2, cfp25* | PROBABLE CUTINASE CUT2 | 30, 31, 34, 59, 118, 119, 147, 174, 208 |  | SP, Tat SP, TM | CF 7, CF 8, CF 9, MEM 15, MEM 8, CW 14 | BlaTEM 18 |  |
| Rv2307c |  | CONSERVED HYPOTHETICAL PROTEIN | 49, 50 | Yes | SP, TM | CW 15, CW 14 |  |  |
| Rv2316 | *uspA* | PROBABLE SUGAR-TRANSPORT INTEGRAL MEMBRANE PROTEIN ABC TRANSPORTER USPA | 71, 231, 252 |  | Tat SP, TM |  |  |  |
| Rv2317 | *uspB* | PROBABLE SUGAR-TRANSPORT INTEGRAL MEMBRANE PROTEIN ABC TRANSPORTER USPB | 36, 63 |  | TM |  |  | Mouse 24, *in vitro* 22 |
| Rv2318 | *uspC* | PROBABLE PERIPLASMIC SUGAR-BINDING LIPOPROTEIN USPC | 30, 35, 133 |  | SP, TM |  |  | *in vitro* 22 |
| Rv2320c | *rocE* | PROBABLE CATIONIC AMINO ACID TRANSPORT INTEGRAL MEMBRANE PROTEIN ROCE | 227, 231, 296, 297, 395, 450 | Yes | TM | MEM 8 | BlaTEM 18 |  |
| Rv2325c |  | CONSERVED HYPOTHETICAL PROTEIN | 67, 100, 109, 125 | Yes | TM | MEM 4, MEM 14, CW 14 |  | *in vitro* 25 |
| Rv2326c |  | POSSIBLE TRANSMEMBRANE ATP-BINDING PROTEIN ABC TRANSORTER | 59, 69, 99, 114, 147 | Yes | TM | MEM 4, MEM 11, MEM 8, MEM 14, CW 15, CW 14 |  |  |
| Rv2329c | *narK1* | PROBABLE NITRITE EXTRUSION PROTEIN 1 NARK1 (NITRITE FACILITATOR 1) | 154, 251, 380, 446 | Yes | TM |  |  |  |
| Rv2330c | *lppP* | PROBABLE LIPOPROTEIN LPPP | 28, 52 |  | SP, Lipo, TM | MEM 8, MEM 14, CW 14 |  | Macrophage 20 |
| Rv2333c | *stp* | PROBABLE CONSERVED INTEGRAL MEMBRANE TRANSPORT PROTEIN | 99, 290, 297, 388, 450, 503 | Yes | TM |  |  |  |
| Rv2339 | *mmpL9* | PROBABLE CONSERVED TRANSMEMBRANE TRANSPORT PROTEIN MMPL9 | 72, 193, 202, 406, 422, 877 | Yes | TM | MEM 8, MEM 14, CW 15, CW 14 | BlaTEM 18 | *in vitro* 22 |
| Rv2345 |  | POSSIBLE CONSERVED TRANSMEMBRANE PROTEIN | 15, 22, 45, 48, 74, 103 | Yes | SP, TM | CF 8, MEM 4, MEM 11, MEM 12, MEM 8, MEM 14, MEM 9, CW 15, CW 19, CW 14, WCL 9 |  |  |
| Rv2376c | *cfp2, mtb12* | LOW MOLECULAR WEIGHT ANTIGEN CFP2 (LOW MOLECULAR WEIGHT PROTEIN ANTIGEN 2) (CFP-2) | 21, 29, 51 |  | SP, TM | CF 7, CF 8, CF 9, MEM 15, MEM 8, CW 15, CW 19, WCL 9, SOL 9 |  |  |
| Rv2380c | *mbtE* | PEPTIDE SYNTHETASE MBTE (PEPTIDE SYNTHASE) |  |  |  | MEM 4, MEM 14, CW 19, SOL 9 |  | Mouse 24, *in vitro* 22 |
| Rv2387 |  | CONSERVED HYPOTHETICAL PROTEIN | 55, 146 | Yes | TM | MEM 8, MEM 14, CW 14 |  | Macaque 19, Mouse 22 |
| Rv2389c | *rpfD* | PROBABLE RESUSCITATION-PROMOTING FACTOR RPFD | 61 |  | SP, TM |  |  |  |
| Rv2390c |  | CONSERVED HYPOTHETICAL PROTEIN | 45, 50, 64 | Yes | TM |  |  |  |
| Rv2394 | *ggtB* | PROBABLE GAMMA-GLUTAMYLTRANSPEPTIDASE PRECURSOR GGTB (GAMMA-GLUTAMYLTRANSFERASE) (GLUTAMYL TRANSPEPTIDASE) | 18, 21, 31, 65, 121, 124, 126, 151, 213, 259, 266, 270, 271, 278, 396, 525, 578 | Yes | SP, Tat SP, Lipo | CF 8, CF 9, MEM 4, MEM 11, MEM 8, MEM 14, MEM 9, CW 19, CW 14 | BlaTEM 18 |  |
| Rv2395 |  | PROBABLE CONSERVED INTEGRAL MEMBRANE PROTEIN | 95, 342, 346, 347, 485, 515 |  | TM |  |  |  |
| Rv2399c | *cysT* | PROBABLE SULFATE-TRANSPORT INTEGRAL MEMBRANE PROTEIN ABC TRANSPORTER CYST | 52, 143, 145 |  | TM | MEM 14, CW 14 |  | *in vitro* 25, *in vitro* 22 |
| Rv2400c | *subI* | PROBABLE SULFATE-BINDING LIPOPROTEIN SUBI | 74, 99, 218, 232, 354 |  | Lipo | CF 8, CF 9, MEM 11, MEM 8, MEM 14, CW 14 |  | Mouse 24, *in vitro* 25, *in vitro* 22 |
| Rv2403c | *lppR* | PROBABLE CONSERVED LIPOPROTEIN LPPR | 24 |  | SP, Lipo, TM | MEM 14, CW 14 |  |  |
| Rv2434c |  | PROBABLE CONSERVED TRANSMEMBRANE PROTEIN | 143 |  | TM |  |  |  |
| Rv2435c |  | PROBABLE CYCLASE (ADENYLYL-OR GUANYLYL-)(ADENYLATE-OR GUANYLATE-) | 69, 148, 155 | Yes | TM | MEM 14 |  |  |
| Rv2437 |  | CONSERVED HYPOTHETICAL PROTEIN | 91 |  | TM |  |  | Mouse 22, *in vitro* 25 |
| Rv2443 | *dctA* | PROBABLE C4-DICARBOXYLATE-TRANSPORT TRANSMEMBRANE PROTEIN DCTA | 46, 130, 135 |  | TM | MEM 8, MEM 14 | BlaTEM 18 |  |
| Rv2450c | *rpfE* | PROBABLE RESUSCITATION-PROMOTING FACTOR RPFE | 22, 38, 108, 116, 127, 128 | Yes | SP, | MEM 15 | PhoA 16 |  |
| Rv2459 |  | PROBABLE CONSERVED INTEGRAL MEMBRANE TRANSPORT PROTEIN | 222, 306 |  | TM |  |  | Macaque 19 |
| Rv2473 |  | POSSIBLE ALANINE AND PROLINE RICH MEMBRANE PROTEIN | 115, 133 | Yes | SP, TM | MEM 8, MEM 14, CW 14 |  |  |
| Rv2507 |  | POSSIBLE CONSERVED PROLINE RICH MEMBRANE PROTEIN | 148, 167, 175, 183, 207, 234 | Yes | TM | MEM 8 |  | *in vitro* 25, *in vitro* 22 |
| Rv2508c |  | PROBABLE CONSERVED INTEGRAL MEMBRANE LEUCINE AND ALANINE RICH PROTEIN | 117, 179, 224, 232, 297, 348 | Yes | TM | MEM 8, MEM 14 |  |  |
| Rv2518c | *lppS* | PROBABLE CONSERVED LIPOPROTEIN LPPS | 34, 38, 81, 157, 402 | Yes | SP, Lipo, TM | MEM 8, MEM 14, CW 15, CW 14 |  | Mouse 24, *in vitro* 22 |
| Rv2519 | *PE26* | PE FAMILY PROTEIN | 255, 333 |  | SP,YxxxD/E |  |  |  |
| Rv2536 |  | PROBABLE CONSERVED TRANSMEMBRANE PROTEIN | 32, 82, 86, 102, 116 |  | TM | CF 9, MEM 11, MEM 8, MEM 14, MEM 9, CW 15, CW 19, CW 14, WCL 9 |  | Mouse 24 |
| Rv2543 | *lppA* | PROBABLE CONSERVED LIPOPROTEIN LPPA | 45 |  | Lipo |  |  | Mouse 24 |
| Rv2544 | *lppB* | PROBABLE CONSERVED LIPOPROTEIN LPPB | 45 |  | Lipo | MEM 15 |  | Mouse 24 |
| Rv2553c |  | PROBABLE CONSERVED MEMBRANE PROTEIN | 76, 78, 89, 127 | Yes | TM | CF 9, MEM 14, CW 14 |  | Mouse 22, Mouse 24, *in vitro* 22 |
| Rv2560 |  | PROBABLE PROLINE AND GLYCINE RICH TRANSMEMBRANE PROTEIN | 135, 170, 174 |  | TM | MEM 8, MEM 14 |  |  |
| Rv2563 |  | PROBABLE GLUTAMINE-TRANSPORT TRANSMEMBRANE PROTEIN ABC TRANSPORTER | 33, 59, 88, 108, 117, 165 | Yes | TM | MEM 4, MEM 11, MEM 15, MEM 8, MEM 14, MEM 9, CW 19, CW 14 |  | Mouse 24 |
| Rv2571c |  | PROBABLE TRANSMEMBRANE ALANINE AND VALINE AND LEUCINE RICH PROTEIN | 94 | Yes | TM | MEM 14, CW 14 |  |  |
| Rv2575 |  | POSSIBLE CONSERVED MEMBRANE GLYCINE RICH PROTEIN | 46, 63, 72, 100, 116, 154, 287 | Yes | Tat SP, TM | CF 8, CF 9, MEM 15, MEM 8, CW 14, SOL 9 |  |  |
| Rv2576c |  | POSSIBLE CONSERVED MEMBRANE PROTEIN | 95, 102, 115, 117, 144 | Yes | SP, TM | CF 8, CF 9, MEM 15, CW 15 |  |  |
| Rv2582 | *ppiB, ppi* | PROBABLE PEPTIDYL-PROLYL CIS-TRANS ISOMERASE B PPIB (CYCLOPHILIN) (PPIASE) (ROTAMASE) (PEPTIDYLPROLYL ISOMERASE) | 93, 95, 130, 204, 234, 242, 284, 287 | Yes | TM | CF 9, MEM 4, MEM 11, MEM 8, MEM 14, MEM 9, CW 19, CW 14, WCL 9 |  | Mouse 24, *in vitro* 25, *in vitro* 22 |
| Rv2586c | *secF* | PROBABLE PROTEIN-EXPORT MEMBRANE PROTEIN SECF | 94, 130 | Yes | TM | MEM 4, MEM 11, MEM 8, MEM 14, MEM 9, CW 14 |  | *in vitro* 22 |
| Rv2587c | *secD* | PROBABLE PROTEIN-EXPORT MEMBRANE PROTEIN SECD | 30, 35, 45, 52, 83, 99, 144, 268, 304, 348 | Yes | TM | MEM 11, MEM 8, MEM 14, MEM 9, CW 15, CW 19, CW 14, WCL 9 | PhoA 17 | *in vitro* 22 |
| Rv2599 |  | PROBABLE CONSERVED MEMBRANE PROTEIN | 108, 129, 136 |  | SP, TM | CF 8, MEM 8, MEM 14, CW 14 | BlaTEM 18 |  |
| Rv2615c | *PE_PGRS45* | PE-PGRS FAMILY PROTEIN | 303 |  | SP, YxxxD/E |  |  |  |
| Rv2617c |  | PROBABLE TRANSMEMBRANE PROTEIN | 109, 111 |  | TM | MEM 8, MEM 14, CW 14 |  |  |
| Rv2639c |  | PROBABLE CONSERVED INTEGRAL MEMBRANE PROTEIN | 62, 70, 107 |  | TM |  | BlaTEM 18 |  |
| Rv2643 | *arsC* | PROBABLE ARSENIC-TRANSPORT INTEGRAL MEMBRANE PROTEIN ARSC | 121, 256, 326 |  | TM | CW 14 |  |  |
| Rv2668 |  | POSSIBLE EXPORTED ALANINE AND VALINE RICH PROTEIN | 28, 44, 70, 107, 129 |  | SP | CF 8, CF 9, MEM 15 |  |  |
| Rv2672 |  | POSSIBLE SECRETED PROTEASE | 89, 135, 209, 223, 230, 327, 330, 334, 369, 373, 526 | Yes | SP, Lipo, TM | CF 8, CF 9, MEM 11, MEM 15, MEM 8, MEM 14, MEM 9, CW 19, CW 14 |  | Mouse 24 |
| Rv2684 | *arsA* | PROBABLE ARSENIC-TRANSPORT INTEGRAL MEMBRANE PROTEIN ARSA | 50 |  | TM | MEM 14 |  |  |
| Rv2686c |  | PROBABLE ANTIBIOTIC-TRANSPORT INTEGRAL MEMBRANE LEUCINE AND ALANINE AND VALINE RICH PROTEIN ABC TRANSPORTER | 135 |  | TM |  |  | Macaque 19 |
| Rv2690c |  | PROBABLE CONSERVED INTEGRAL MEMBRANE ALANINE AND VALINE AND LEUCINE RICH PROTEIN | 205, 283, 394, 463 | Yes | TM | MEM 14, CW 14 |  | Macaque 19, Mouse 24, *in vitro* 22 |
| Rv2698 |  | PROBABLE CONSERVED ALANINE RICH TRANSMEMBRANE PROTEIN | 58 | Yes | TM | MEM 8, MEM 14, CW 14 |  | *in vitro* 25, *in vitro* 22 |
| Rv2700 |  | POSSIBLE CONSERVED SECRETED ALANINE RICH PROTEIN | 84, 120, 128, 196, 204 |  | TM | CF 9, MEM 11, MEM 8, MEM 14, CW 14 |  | *in vitro* 25, *in vitro* 22 |
| Rv2719c |  | POSSIBLE CONSERVED MEMBRANE PROTEIN | 93, 94, 104 | Yes | TM |  |  |  |
| Rv2721c |  | POSSIBLE CONSERVED TRANSMEMBRANE ALANINE AND GLYCINE RICH PROTEIN | 46, 77, 78, 87, 104, 133, 152, 222, 235, 250, 269, 315, 334, 339, 370, 387, 395, 413 | Yes | SP, TM | CF 8, CF 9, MEM 11, MEM 15, MEM 8, MEM 14, MEM 9, CW 15, CW 19, CW 14 | BlaTEM 18 |  |
| Rv2723 |  | PROBABLE CONSERVED INTEGRAL MEMBRANE PROTEIN | 59, 122, 231, 287, 313 |  | TM | MEM 8 |  |  |
| Rv2729c |  | PROBABLE CONSERVED INTEGRAL MEMBRANE ALANINE VALINE AND LEUCINE RICH PROTEIN | 127, 226, 287, 288 |  | TM |  |  |  |
| Rv2732c |  | PROBABLE CONSERVED TRANSMEMBRANE PROTEIN | 53, 57, 68, 130 | Yes | Tat SP, TM | MEM 8, MEM 14, CW 14 |  |  |
| Rv2746c | *pgsA3* | PROBABLE PGP SYNTHASE PGSA3 (CDP-DIACYLGLYCEROL--GLYCEROL-3-PHOSPHATE 3-PHOSPHATIDYLTRANSFERASE) (PHOSPHATIDYLGLYCEROPHOSPHATE SYNTHASE) | 50, 55, 67, 179 | Yes | TM | MEM 8, MEM 14, CW 14 |  | *in vitro* 25, *in vitro* 22 |
| Rv2748c | *ftsK* | POSSIBLE CELL DIVISION TRANSMEMBRANE PROTEIN FTSK | 168, 246 | Yes | TM | MEM 14, CW 12, CW 15, CW 14 | PhoA 17 | *in vitro* 25, *in vitro* 22 |
| Rv2772c |  | PROBABLE CONSERVED TRANSMEMBRANE PROTEIN | 41, 64 |  | Tat SP, TM | MEM 8, MEM 14, CW 14 |  |  |
| Rv2784c | *lppU* | PROBABLE LIPOPROTEIN LPPU | 165, 166 |  | SP, Lipo | MEM 8, MEM 14 |  |  |
| Rv2796c | *lppV* | PROBABLE CONSERVED LIPOPROTEIN LPPV | 18, 27, 86 |  | SP, Lipo | MEM 14, CW 14 |  | Macaque 19 |
| Rv2799 |  | PROBABLE MEMBRANE PROTEIN | 47, 48, 57, 140, 144 |  | TM | CF 8, CF 9, MEM 15, CW 14 |  |  |
| Rv2806 |  | POSSIBLE MEMBRANE PROTEIN | 38 |  | TM |  |  |  |
| Rv2835c | *ugpA* | PROBABLE Sn-GLYCEROL-3-PHOSPHATE TRANSPORT INTEGRAL MEMBRANE PROTEIN ABC TRANSPORTER UGPA | 247, 271 |  | TM |  |  |  |
| Rv2843 |  | PROBABLE CONSERVED TRANSMEMBRANE ALANINE RICH PROTEIN | 44, 65 |  | SP, Tat SP, Lipo, TM | MEM 8, MEM 14, CW 14 | BlaC 2 |  |
| Rv2846c | *efpA* | POSSIBLE INTEGRAL MEMBRANE EFFLUX PROTEIN EFPA | 87, 94, 160, 186 |  | TM | MEM 8, MEM 14 |  | *in vitro* 25, *in vitro* 22 |
| Rv2856 | *nicT* | POSSIBLE NICKEL-TRANSPORT INTEGRAL MEMBRANE PROTEIN NICT | 51, 57, 152, 328 | Yes | TM |  |  | *in vitro* 25 |
| Rv2864c |  | POSSIBLE PENICILLIN-BINDING LIPOPROTEIN | 31, 40, 47, 49 | Yes | SP, Lipo | MEM 14, CW 15 |  |  |
| Rv2869c |  | PROBABLE CONSERVED TRANSMEMBRANE PROTEIN | 155, 165, 223, 238, 250, 259, 285, 392 |  | TM | CF 8, MEM 8, MEM 14, CW 14 |  | Mouse 24, *in vitro* 25, *in vitro* 22 |
| Rv2873 | *mpt83, mpb83* | CELL SURFACE LIPOPROTEIN MPT83 (LIPOPROTEIN P23) | 54, 118, 198 |  | SP, Lipo | CF 8, CF 9, MEM 4, MEM 11, MEM 15, MEM 8, MEM 14, MEM 9, CW 19, CW 14, WCL 9 |  |  |
| Rv2874 | *dipZ* | POSSIBLE INTEGRAL MEMBRANE C-TYPE CYTOCHROME BIOGENESIS PROTEIN DIPZ | 143, 353, 362, 396, 406, 412, 545, 583 | Yes | TM | MEM 15 |  |  |
| Rv2877c | *, merT* | PROBABLE CONSERVED INTEGRAL MEMBRANE PROTEIN | 75, 158 |  | TM | MEM 8, MEM 14 |  |  |
| Rv2903c | *lepB* | PROBABLE SIGNAL PEPTIDASE I LEPB (SPASE I) (LEADER PEPTIDASE I). | 179, 229 |  | TM | MEM 11, MEM 8, MEM 14, CW 14 | PhoA 17 | *in vitro* 25, *in vitro* 22 |
| Rv2905 | *lppW* | PROBABLE CONSERVED ALANINE RICH LIPOPROTEIN LPPW | 85, 115, 166, 212 |  | SP, Lipo | MEM 15, CW 14 | BlaTEM 18 |  |
| Rv2911 | *dacB2, dacB* | PROBABLE D-ALANYL-D-ALANINE CARBOXYPEPTIDASE DACB2 (PENICILLIN-BINDING PROTEIN) (DD-PEPTIDASE) (DD-CARBOXYPEPTIDASE) (PBP) (DD-TRANSPEPTIDASE) (SERINE-TYPE D-ALA-D-ALA CARBOXYPEPTIDASE) (D-AMINO ACID HYDROLASE) | 21, 31, 51, 56, 66, 127, 168, 169, 171, 243, 246 | Yes | SP, Tat SP | CF 8, CF 9, MEM 15, CW 19, CW 14, SOL 9 |  | Macaque 19 |
| Rv2914c | *pknI* | PROBABLE TRANSMEMBRANE SERINE/THREONINE-PROTEIN KINASE I PKNI (PROTEIN KINASE I) (STPK I) (PHOSPHORYLASE B KINASE KINASE) (HYDROXYALKYL-PROTEIN KINASE) | 376, 582 | Yes | TM | MEM 14, CW 14 |  |  |
| Rv2920c | *amt* | PROBABLE AMMONIUM-TRANSPORT INTEGRAL MEMBRANE PROTEIN AMT | 79, 105, 175, 179, 192, 195, 201, 257, 382, 428 | Yes | TM | MEM 8 |  | Macaque 19 |
| Rv2938 | *drrC* | PROBABLE DAUNORUBICIN-DIM-TRANSPORT INTEGRAL MEMBRANE PROTEIN ABC TRANSPORTER DRRC | 235 |  | TM | MEM 8, MEM 14, CW 15, CW 14 | BlaTEM 18 | Mouse 22 |
| Rv2942 | *mmpL7* | CONSERVED TRANSMEMBRANE TRANSPORT PROTEIN MMPL7 | 95, 276, 420, 438, 487 | Yes | TM | MEM 11, MEM 8, MEM 14, MEM 9, CW 14 |  | Macrophage 20, Mouse 22 |
| Rv2945c | *lppX* | PROBABLE CONSERVED LIPOPROTEIN LPPX | 47 | Yes | SP, Lipo, TM | CF 8, CF 9, MEM 4, MEM 11, MEM 15, MEM 8, MEM 14, MEM 9, CW 15, CW 19, CW 14, WCL 9, SOL 9 | PhoA 17, BlaTEM 18 | Mouse 22, Mouse 24 |
| Rv2963 |  | PROBABLE INTEGRAL MEMBRANE PROTEIN | 127, 347 |  | TM | MEM 8, MEM 14 |  |  |
| Rv2968c |  | PROBABLE CONSERVED INTEGRAL MEMBRANE PROTEIN | 64 |  | TM | MEM 12 |  | *in vitro* 25, *in vitro* 22 |
| Rv2969c |  | POSSIBLE CONSERVED MEMBRANE OR SECRETED PROTEIN | 56, 71, 91, 96 | Yes | TM | CF 8, CF 9, MEM 4, MEM 11, MEM 8, MEM 14, MEM 9, CW 15, CW 19, CW 14, WCL 9 |  | *in vitro* 25, *in vitro* 22 |
| Rv2972c |  | POSSIBLE CONSERVED MEMBRANE OR EXPORTED PROTEIN | 35, 45, 51 | Yes | SP, Tat SP, TM | MEM 14 |  |  |
| Rv2994 |  | PROBABLE CONSERVED INTEGRAL MEMBRANE PROTEIN | 42, 113, 114, 120, 248, 331, 392 | Yes | TM | MEM 15, MEM 8, MEM 14, CW 14 |  |  |
| Rv2999 | *lppY* | PROBABLE CONSERVED LIPOPROTEIN LPPY | 36, 68, 95, 160, 194, 246 | Yes | SP, Lipo | MEM 4, MEM 11, MEM 8, MEM 14, CW 14 |  | *in vitro* 25 |
| Rv3005c |  | CONSERVED HYPOTHETICAL PROTEIN | 166, 171 | Yes | TM | MEM 14, CW 14 |  | Mouse 24 |
| Rv3006 | *lppZ* | PROBABLE CONSERVED LIPOPROTEIN LPPZ | 21, 95, 118, 198, 232, 343 | Yes | SP, Lipo | CF 8, CF 9, MEM 4, MEM 11, MEM 15, MEM 8, MEM 14, MEM 9, CW 15, CW 19, CW 14, WCL 9 |  | *in vitro* 22 |
| Rv3016 | *lpqA* | PROBABLE LIPOPROTEIN LPQA | 24, 32, 55, 57, 129 |  | SP, Lipo | CF 8, MEM 15, CW 14 |  |  |
| Rv3033 |  | HYPOTHETICAL PROTEIN | 46, 52, 53, 167 |  | SP, | CF 8, CF 9, MEM 4, MEM 15, MEM 8, MEM 14, CW 19, CW 14 |  | Macrophage 20 |
| Rv3035 |  | CONSERVED HYPOTHETICAL PROTEIN | 2, 9, 20, 44, 48, 133, 149, 224, 254, 259, 285, 307, 328 | Yes |  | MEM 8, MEM 14, CW 14 |  | *in vitro* 22 |
| Rv3036c | *TB22.2* | PROBABLE CONSERVED SECRETED PROTEIN TB22.2 | 27, 30, 35, 100, 148, 168, 172, 178 | Yes | SP, | CF 7, CF 8, CF 9, MEM 15, CW 14, WCL 9, SOL 9 | BlaTEM 18 | Mouse 24 |
| Rv3043c | *ctaD* | PROBABLE CYTOCHROME C OXIDASE POLYPEPTIDE I CTAD (CYTOCHROME AA3 SUBUNIT 1) | 66, 68, 137, 143, 165, 371 |  | TM | MEM 11, MEM 8, MEM 14, MEM 9, CW 19, CW 14, WCL 9 |  | *in vitro* 25, *in vitro* 22 |
| Rv3044 | *fecB* | PROBABLE FEIII-DICITRATE-BINDING PERIPLASMIC LIPOPROTEIN FECB | 40, 60, 113, 135, 179, 195, 208, 276 |  | SP, Lipo | CF 8, CF 9, MEM 4, MEM 11, MEM 15, MEM 14, MEM 9, CW 19, CW 14, WCL 9 |  | Mouse 24, *in vitro* 22 |
| Rv3063 | *cstA* | PROBABLE CARBON STARVATION PROTEIN A HOMOLOG CSTA | 269, 348, 357, 441, 464, 477, 478, 578 | Yes | Tat SP, TM |  |  |  |
| Rv3064c |  | PROBABLE CONSERVED INTEGRAL MEMBRANE PROTEIN | 52, 56 |  | TM |  |  |  |
| Rv3067 |  | CONSERVED HYPOTHETICAL PROTEIN | 29, 41, 44, 67, 76 | Yes |  | CF 8 |  |  |
| Rv3090 |  | HYPOTHETICAL ALANINE AND VALINE RICH PROTEIN | 71, 119, 133, 166, 188, 197 |  | TM | MEM 4, MEM 11, MEM 8, MEM 14, MEM 9, CW 19, CW 14, SOL 9 | BlaTEM 18 |  |
| Rv3092c |  | PROBABLE CONSERVED INTEGRAL MEMBRANE PROTEIN | 113, 123, 134, 144 |  | TM | MEM 8, MEM 14, CW 14 |  |  |
| Rv3103c |  | HYPOTHETICAL PROLINE-RICH PROTEIN | 127 |  | TM |  | BlaTEM 18 | Mouse 22 |
| Rv3123 |  | HYPOTHETICAL PROTEIN | 145 |  |  | MEM 14 |  |  |
| Rv3152 | *nuoH* | PROBABLE NADH DEHYDROGENASE I (CHAIN H) NUOH (NADH-UBIQUINONE OXIDOREDUCTASE CHAIN H) | 280, 289 | Yes | Tat SP, TM | MEM 8, MEM 14, CW 14 |  |  |
| Rv3156 | *nuoL* | PROBABLE NADH DEHYDROGENASE I (CHAIN L) NUOL (NADH-UBIQUINONE OXIDOREDUCTASE CHAIN L) | 75, 76, 77, 211, 233, 354, 414, 422 |  | TM | MEM 8, MEM 14, CW 14 |  |  |
| Rv3157 | *nuoM* | PROBABLE NADH DEHYDROGENASE I (CHAIN M) NUOK (NADH-UBIQUINONE OXIDOREDUCTASE CHAIN M) | 74, 218, 233, 530 | Yes | SP, Tat SP, TM | MEM 8, MEM 14, MEM 9, CW 19, CW 14, WCL 9 |  | *in vitro* 22 |
| Rv3158 | *nuoN* | PROBABLE NADH DEHYDROGENASE I (CHAIN N) NUON (NADH-UBIQUINONE OXIDOREDUCTASE CHAIN N) | 368, 447, 450, 451 |  | TM | MEM 11, MEM 15, MEM 8, MEM 14, CW 14 |  |  |
| Rv3165c |  | HYPOTHETICAL PROTEIN | 29 | Yes | TM | MEM 8, MEM 14, CW 14 |  |  |
| Rv3166c |  | CONSERVED HYPOTHETICAL PROTEIN | 29, 43, 44, 58, 137 | Yes | TM | MEM 14, CW 14 |  | Mouse 24 |
| Rv3193c |  | PROBABLE CONSERVED TRANSMEMBRANE PROTEIN | 142, 239, 300, 328, 451, 529, 531, 678 | Yes | TM | CF 9, MEM 4, MEM 11, MEM 15, MEM 8, MEM 14, MEM 9, CW 15, CW 19, CW 14, WCL 9, SOL 9 |  | Mouse 24, *in vitro* 22 |
| Rv3194c |  | POSSIBLE CONSERVED SECRETED PROTEIN | 33, 111, 119, 152, 171, 194, 238, 264 | Yes | SP, TM | CF 9, MEM 11, MEM 15, MEM 8, MEM 14, CW 14 |  | Mouse 24 |
| Rv3207c |  | CONSERVED HYPOTHETICAL PROTEIN | 33, 50, 58, 120, 152, 204 |  | SP, TM | CF 8, MEM 14, CW 14 |  | Mouse 24 |
| Rv3209 |  | CONSERVED HYPOTHETICAL THREONIN AND PROLINE RICH PROTEIN | 70, 81 | Yes | SP, |  | BlaTEM 18 | Mouse 24 |
| Rv3217c |  | PROBABLE CONSERVED INTEGRAL MEMBRANE PROTEIN | 47, 55, 60, 61 | Yes | TM |  |  |  |
| Rv3236c | *, kefB* | PROBABLE CONSERVED INTEGRAL MEMBRANE TRANSPORT PROTEIN | 51, 53, 59 |  | TM | MEM 8, CW 14 |  | Mouse 22 |
| Rv3239c |  | PROBABLE CONSERVED TRANSMEMBRANE TRANSPORT PROTEIN | 446 |  | Tat SP, TM |  |  |  |
| Rv3244c | *lpqB* | PROBABLE CONSERVED LIPOPROTEIN LPQB | 174, 232, 336 |  | SP, Lipo | CF 8, CF 9, MEM 11, MEM 15, MEM 8, MEM 14, MEM 9, CW 15, CW 14, SOL 9 |  | *in vitro* 25, *in vitro* 22 |
| Rv3245c | *mtrB* | TWO COMPONENT SENSORY TRANSDUCTION HISTIDINE KINASE MTRB | 88, 116, 154, 175 |  | TM | MEM 14, CW 14 |  | Mouse 24, *in vitro* 25, *in vitro* 22 |
| Rv3252c | *alkB* | PROBABLE TRANSMEMBRANE ALKANE 1-MONOOXYGENASE ALKB (ALKANE 1-HYDROXYLASE) (LAURIC ACID OMEGA-HYDROXYLASE) (OMEGA-HYDROXYLASE) (FATTY ACID OMEGA-HYDROXYLASE) (ALKANE HYDROXYLASE-RUBREDOXIN) | 55 |  | TM | MEM 14 |  |  |
| Rv3253c |  | POSSIBLE CATIONIC AMINO ACID TRANSPORT INTEGRAL MEMBRANE PROTEIN | 49, 52, 199, 215, 227, 241, 314, 325, 334, 413, 453, 468 | Yes | TM | MEM 14 | BlaTEM 18 |  |
| Rv3267 |  | CONSERVED HYPOTHETICAL PROTEIN (CPSA-RELATED PROTEIN) | 28, 87 | Yes | SP, TM | CF 8, CF 9, MEM 15, MEM 14, MEM 9, CW 19, CW 14 | PhoA 17, BlaTEM 18 | Mouse 24, *in vitro* 25, *in vitro* 22 |
| Rv3271c |  | PROBABLE CONSERVED INTEGRAL MEMBRANE PROTEIN | 62, 68, 83, 89, 120, 203 | Yes | TM | MEM 14, CW 14 |  | *in vitro* 25 |
| Rv3273 |  | PROBABLE TRANSMEMBRANE CARBONIC ANHYDRASE (CARBONATE DEHYDRATASE) (CARBONIC DEHYDRATASE) | 55, 61, 69, 73, 366 | Yes | TM | MEM 4, MEM 11, MEM 8, MEM 14, MEM 9, CW 19, CW 14, WCL 9 |  |  |
| Rv3274c | *fadE25* | PROBABLE ACYL-CoA DEHYDROGENASE FADE25 | 316 |  |  | CF 8, CF 9, MEM 4, MEM 11, MEM 8, MEM 14, MEM 9, CW 15, CW 19, CW 14, WCL 9, SOL 9 |  | Mouse 24 |
| Rv3278c |  | PROBABLE CONSERVED TRANSMEMBRANE PROTEIN | 42 | Yes | TM | MEM 8, MEM 14, CW 14 |  |  |
| Rv3289c |  | POSSIBLE TRANSMEMBRANE PROTEIN | 52 |  | TM |  |  |  |
| Rv3298c | *lpqC* | POSSIBLE ESTERASE LIPOPROTEIN LPQC | 19, 49, 53, 63, 65, 67, 81, 120, 131, 137, 252 | Yes | SP, Lipo | MEM 11, MEM 8, MEM 14, CW 14 |  |  |
| Rv3310 | *sapM* | POSSIBLE ACID PHOSPHATASE (ACID PHOSPHOMONOESTERASE) (PHOSPHOMONOESTERASE) (GLYCEROPHOSPHATASE) | 78, 118, 131, 186, 258 |  | SP, TM | CF 8, CF 9, MEM 15, MEM 8 |  |  |
| Rv3312A | *mtp* | SECRETED PROTEIN ANTIGEN | 32, 48, 77 |  | SP, TM | CF 9, WCL 9, SOL 9 |  |  |
| Rv3316 | *sdhC* | PROBABLE SUCCINATE DEHYDROGENASE (CYTOCHROME B-556 SUBUNIT) SDHC (SUCCINIC DEHYDROGENASE) (FUMARATE REDUCTASE) (FUMARATE DEHYDROGENASE) (FUMARIC HYDROGENASE) | 24 |  | TM | MEM 8, MEM 14 |  | Mouse 24 |
| Rv3330 | *dacB1* | PROBABLE PENICILLIN-BINDING PROTEIN DACB1 (D-ALANYL-D-ALANINE CARBOXYPEPTIDASE) (DD-PEPTIDASE) (DD-CARBOXYPEPTIDASE) (PBP) (DD-TRANSPEPTIDASE) (SERINE-TYPE D-ALA-D-ALA CARBOXYPEPTIDASE) (D-AMINO ACID HYDROLASE) | 24, 26, 31, 52, 54, 69, 76, 80, 88, 108, 141, 158, 192, 215 | Yes | SP, TM | CF 8, MEM 14, CW 14 |  |  |
| Rv3331 | *sugI* | PROBABLE SUGAR-TRANSPORT INTEGRAL MEMBRANE PROTEIN SUGI | 200, 212, 312, 332 | Yes | TM | MEM 14, CW 14 |  |  |
| Rv3343c | *PPE54* | PPE FAMILY PROTEIN | 555 |  |  |  |  | *in vitro* 25, *in vitro* 22 |
| Rv3350c | *PPE56* | PPE FAMILY PROTEIN | 3387 |  |  |  |  | *in vitro* 22 |
| Rv3354 |  | CONSERVED HYPOTHETICAL PROTEIN | 26, 40, 45, 113, 126 |  | SP, TM | MEM 15 |  |  |
| Rv3365c |  | CONSERVED HYPOTHETICAL PROTEIN | 79, 104, 116, 145, 203, 215, 232, 370 | Yes | TM | MEM 14, CW 14 |  |  |
| Rv3390 | *lpqD* | PROBABLE CONSERVED LIPOPROTEIN LPQD | 25, 57, 59, 95, 96, 100, 118, 154, 164 | Yes | SP, Lipo, TM | MEM 4, MEM 11, MEM 8, MEM 14, MEM 9, CW 19, CW 14 | BlaTEM 18 |  |
| Rv3395A |  | PROBABLE MEMBRANE PROTEIN | 28, 30, 60, 103 | Yes | SP, TM | CF 8 |  | Mouse 24 |
| Rv3413c |  | HYPOTHETICAL ALANINE AND PROLINE RICH PROTEIN | 110, 200, 277 | Yes | TM | CF 8, CF 9, MEM 15 | BlaTEM 18 |  |
| Rv3434c |  | POSSIBLE CONSERVED TRANSMEMBRANE PROTEIN | 44, 191 |  | TM |  |  | Mouse 24 |
| Rv3435c |  | PROBABLE CONSERVED TRANSMEMBRANE PROTEIN | 27, 106, 116, 119 | Yes | TM | MEM 8, MEM 14, CW 14 |  |  |
| Rv3451 | *cut3* | PROBABLE CUTINASE PRECURSOR CUT3 | 52, 54, 58, 87, 134, 169, 198, 203, 227, 241 | Yes | SP, TM | CF 8, MEM 8 |  |  |
| Rv3452 | *cut4* | PROBABLE CUTINASE PRECURSOR CUT4 | 36, 55, 57, 124, 137, 142, 152, 159, 172 | Yes | SP, TM |  |  |  |
| Rv3476c | *kgtP* | PROBABLE DICARBOXYLIC ACID TRANSPORT INTEGRAL MEMBRANE PROTEIN KGTP (DICARBOXYLATE TRANSPORTER) | 284 |  | TM | MEM 8 | BlaTEM 18 |  |
| Rv3478 | *PPE60, mtb39c* | PE FAMILY PROTEIN | 155, 177 |  |  | MEM 8, MEM 14, CW 19, CW 14 |  |  |
| Rv3481c |  | PROBABLE INTEGRAL MEMBRANE PROTEIN | 62, 69, 73, 147, 158 |  | TM | MEM 8, MEM 14 |  |  |
| Rv3482c |  | PROBABLE CONSERVED MEMBRANE PROTEIN | 104, 109 |  | TM | MEM 11, MEM 14, CW 14 |  |  |
| Rv3483c |  | CONSERVED HYPOTHETICAL PROTEIN | 52, 70, 126, 154 |  | TM | MEM 11, MEM 8, MEM 14, CW 14 |  |  |
| Rv3484 | *cpsA* | POSSIBLE CONSERVED PROTEIN CPSA | 39, 51, 67, 105, 193, 206, 216, 231, 236, 237, 321, 348, 384, 395, 401, 452, 487 | Yes | TM | CF 8, MEM 15, MEM 8 | BlaTEM 18 | Macrophage 21, Mouse 22, Mouse 24, *in vitro* 22 |
| Rv3491 |  | HYPOTHETICAL PROTEIN | 24, 44, 47, 54, 101 |  | SP, TM | CF 8, MEM 15 |  | Mouse 24 |
| Rv3492c |  | CONSERVED HYPOTHETICAL MCE ASSOCIATED PROTEIN | 19, 21 | Yes | SP, TM | MEM 14, CW 14 |  |  |
| Rv3493c |  | CONSERVED HYPOTHETICAL MCE ASSOCIATED ALANINE AND VALINE RICH PROTEIN | 118, 150, 180, 230 |  | TM | MEM 11, MEM 14, CW 14 |  | Mouse 24 |
| Rv3494c | *mce4F* | MCE-FAMILY PROTEIN MCE4F | 31, 48, 60, 171, 354, 363 | Yes | TM | CF 9, MEM 14, CW 14 | BlaTEM 18 | Macaque 19, Mouse 24 |
| Rv3496c | *mce4D* | MCE-FAMILY PROTEIN MCE4D | 35, 418, 448 |  | TM | MEM 14, CW 15, CW 14 | BlaTEM 18 | Mouse 24 |
| Rv3497c | *mce4C* | MCE-FAMILY PROTEIN MCE4C | 53, 58, 78, 120, 303, 305, 316 | Yes | TM | MEM 11, MEM 8, MEM 14, CW 14 | BlaTEM 18 | Mouse 22, Mouse 24 |
| Rv3498c | *mce4B* | MCE-FAMILY PROTEIN MCE4B | 27, 63, 137, 302 | Yes | SP, TM | MEM 14, CW 14 | BlaTEM 18 | Mouse 24 |
| Rv3499c | *mce4A, mce4* | MCE-FAMILY PROTEIN MCE4A | 24, 49, 87, 102, 171, 196, 215, 216 | Yes | SP, TM | MEM 12, MEM 14, CW 14 |  | Mouse 22 |
| Rv3500c | *yrbE4B* | CONSERVED HYPOTHETICAL INTEGRAL MEMBRANE PROTEIN YRBE4B | 78, 99, 193, 272 |  | TM | MEM 8, MEM 14, CW 14 |  |  |
| Rv3501c | *yrbE4A* | CONSERVED HYPOTHETICAL INTEGRAL MEMBRANE PROTEIN YRBE4A | 74, 78, 80, 159, 160, 184, 244 | Yes | TM | MEM 8, MEM 14, CW 14 |  | Mouse 22 |
| Rv3507 | *PE_PGRS53* | PE-PGRS FAMILY PROTEIN | 820, 1320 |  | SP, Tat SP, YxxxD/E |  |  |  |
| Rv3524 |  | PROBABLE CONSERVED MEMBRANE PROTEIN | 122, 132, 149, 174, 248, 257, 274, 275, 300, 311 |  | TM | MEM 14, CW 14 |  |  |
| Rv3526 |  | POSSIBLE OXIDOREDUCTASE | 381 |  |  |  |  |  |
| Rv3554 | *fdxB* | POSSIBLE ELECTRON TRANSFER PROTEIN FDXB | 65 |  | TM | MEM 14, CW 14 |  |  |
| Rv3572 |  | HYPOTHETICAL PROTEIN | 24, 74, 129, 139 | Yes | SP, | CF 8, CF 9, MEM 15, MEM 8, CW 14 |  |  |
| Rv3576 | *lppH, pknM* | POSSIBLE CONSERVED LIPOPROTEIN LPPH | 18, 27, 34, 59, 102, 135, 163, 168, 190 | Yes | SP, Lipo | CF 9, MEM 8, MEM 14, CW 14 |  |  |
| Rv3578 | *arsB2* | POSSIBLE ARSENICAL PUMP INTEGRAL MEMBRANE PROTEIN ARSB2 | 293, 297, 302, 307 |  | SP, TM |  |  | Mouse 24 |
| Rv3584 | *lpqE* | POSSIBLE CONSERVED LIPOPROTEIN LPQE | 27, 106, 112, 114, 163 | Yes | SP, Lipo, TM | CF 8, CF 9, MEM 4, MEM 11, MEM 15, MEM 8, MEM 14, MEM 9, CW 15, CW 19, CW 14, WCL 9 | BlaTEM 18 |  |
| Rv3587c |  | PROBABLE CONSERVED MEMBRANE PROTEIN | 44, 95, 156 | Yes | SP, TM | CF 8, CF 9, MEM 15, MEM 8, MEM 14, CW 14 |  | *in vitro* 22 |
| Rv3593 | *lpqF* | PROBABLE CONSERVED LIPOPROTEIN LPQF | 38, 82, 104, 186, 231 | Yes | SP, Lipo |  |  | *in vitro* 25, *in vitro* 22 |
| Rv3596c | *clpC1, clpC* | PROBABLE ATP-DEPENDENT PROTEASE ATP-BINDING SUBUNIT CLPC1 | 598 |  |  | CF 9, MEM 4, MEM 12, MEM 8, MEM 14, MEM 9, CW 15, CW 19, CW 14, WCL 9, SOL 9 |  | Macrophage 20, Macrophage 21, *in vitro* 25, *in vitro* 22 |
| Rv3604c |  | PROBABLE CONSERVED TRANSMEMBRANE PROTEIN RICH IN ALANINE AND ARGININE AND PROLINE | 33 | Yes | TM | MEM 12, MEM 14, CW 14 |  | *in vitro* 25, *in vitro* 22 |
| Rv3610c | *ftsH* | MEMBRANE-BOUND PROTEASE FTSH (CELL DIVISION PROTEIN) | 30, 84 |  | TM | MEM 4, MEM 14, MEM 9, CW 19, CW 14, WCL 9 |  | Mouse 24, *in vitro* 25, *in vitro* 22 |
| Rv3623 | *lpqG* | PROBABLE CONSERVED LIPOPROTEIN LPQG | 42, 61, 112, 116, 144, 145, 214, 223 |  | SP, Lipo | MEM 4, MEM 11, MEM 8, MEM 14, MEM 9, CW 15, CW 19, CW 14, WCL 9, SOL 9 |  |  |
| Rv3627c |  | CONSERVED HYPOTHETICAL PROTEIN | 27, 62, 68, 72, 96, 139, 140, 163, 200, 228, 260, 335, 381, 401, 408, 438 | Yes | SP, TM | CF 8, MEM 11, MEM 15, MEM 8, MEM 14, CW 14 |  | *in vitro* 25, *in vitro* 22 |
| Rv3629c |  | PROBABLE CONSERVED INTEGRAL MEMBRANE PROTEIN | 27, 89, 124, 200, 333 | Yes | TM | MEM 15 |  |  |
| Rv3635 |  | PROBABLE CONSERVED TRANSMEMBRANE PROTEIN | 43, 64, 397, 423 |  | TM | MEM 14 |  | *in vitro* 25, *in vitro* 22 |
| Rv3654c |  | CONSERVED HYPOTHETICAL PROTEIN | 16, 18, 23 |  |  |  |  |  |
| Rv3655c |  | CONSERVED HYPOTHETICAL PROTEIN | 42 |  | SP, TM |  |  |  |
| Rv3664c | *dppC* | PROBABLE DIPEPTIDE-TRANSPORT INTEGRAL MEMBRANE PROTEIN ABC TRANSPORTER DPPC | 38 |  | SP, TM |  |  | Macrophage 21, Mouse 24 |
| Rv3665c | *dppB* | PROBABLE DIPEPTIDE-TRANSPORT INTEGRAL MEMBRANE PROTEIN ABC TRANSPORTER DPPB | 30, 70, 129 |  | TM |  |  | Mouse 24, *in vitro* 22 |
| Rv3666c | *dppA* | PROBABLE PERIPLASMIC DIPEPTIDE-BINDING LIPOPROTEIN DPPA | 22, 25, 38, 48, 64, 97, 154, 215, 254, 261, 288 | Yes | SP, Lipo, TM | CF 8, MEM 8, CW 15, CW 14 |  | Mouse 24, *in vitro* 25 |
| Rv3667 | *acs* | ACETYL-COENZYME A SYNTHETASE ACS (ACETATE--CoA LIGASE) (ACETYL-CoA SYNTHETASE) (ACETYL-CoA SYNTHASE) (ACYL-ACTIVATING ENZYME) (ACETATE THIOKINASE) (ACETYL-ACTIVATING ENZYME) (ACETATE--COENZYME A LIGASE) (ACETYL-COENZYME A SYNTHASE) |  |  |  | MEM 4, MEM 14, CW 19, CW 14, WCL 9, SOL 9 |  |  |
| Rv3668c |  | POSSIBLE PROTEASE | 25, 35, 92, 144 | Yes | SP, TM | CF 8, CF 9, MEM 15 | PhoA 16 | Mouse 24 |
| Rv3671c |  | POSSIBLE MEMBRANE-ASSOCIATED SERINE PROTEASE | 63, 166, 296, 305, 391 | Yes | TM | CF 8, CF 9, MEM 11, MEM 15, MEM 8, MEM 14, MEM 9, CW 15, CW 19, CW 14, WCL 9, SOL 9 |  | Mouse 24, *in vitro* 22 |
| Rv3673c |  | POSSIBLE MEMBRANE-ANCHORED THIOREDOXIN-LIKE PROTEIN (THIOL-DISULFIDE INTERCHANGE RELATED PROTEIN) | 44, 53, 92 |  | TM | MEM 8, MEM 14, CW 14 |  | *in vitro* 22 |
| Rv3682 | *ponA2* | PROBABLE BIFUNCTIONAL MEMBRANE-ASSOCIATED PENICILLIN-BINDING PROTEIN 1A/1B PONA2 (MUREIN POLYMERASE) [INCLUDES: PENICILLIN-INSENSITIVE TRANSGLYCOSYLASE (PEPTIDOGLYCAN TGASE) + PENICILLIN-SENSITIVE TRANSPEPTIDASE (DD-TRANSPEPTIDASE)] | 33, 117, 124, 130, 285, 290, 317, 335, 346, 379, 395, 405, 432, 466, 597, 613, 734, 746 | Yes | SP, TM | CF 8, CF 9, MEM 4, MEM 15, MEM 8, MEM 14, MEM 9, CW 15, CW 14 |  | Macrophage 20, Mouse 24 |
| Rv3691 |  | CONSERVED HYPOTHETICAL PROTEIN | 2 |  |  | MEM 4, MEM 8, MEM 14, CW 14 |  |  |
| Rv3694c |  | POSSIBLE CONSERVED TRANSMEMBRANE PROTEIN | 128, 153, 197 |  | TM | MEM 8, MEM 14, CW 14 |  |  |
| Rv3695 |  | POSSIBLE CONSERVED MEMBRANE PROTEIN | 45, 56, 123 |  | TM | MEM 8, MEM 14, CW 14 |  |  |
| Rv3701c |  | CONSERVED HYPOTHETICAL PROTEIN |  |  |  | MEM 14, CW 14 |  | Macrophage 20, Mouse 22 |
| Rv3705A |  | CONSERVED HYPOTHETICAL PROLINE RICH PROTEIN | 82, 93, 94, 103, 123 | Yes | TM | MEM 14 |  |  |
| Rv3705c |  | CONSERVED HYPOTHETICAL PROTEIN | 29, 153, 169, 194, 197, 199, 205 | Yes | SP, | CF 8, CF 9, MEM 15, MEM 8, SOL 9 |  |  |
| Rv3706c |  | CONSERVED HYPOTHETICAL PROLINE RICH PROTEIN | 43, 51, 54, 78, 90, 100, 103 |  | TM |  |  |  |
| Rv3707c |  | CONSERVED HYPOTHETICAL PROTEIN | 42 |  |  | MEM 14 |  |  |
| Rv3717 |  | CONSERVED HYPOTHETICAL PROTEIN | 15, 24, 48 | Yes | SP, | CF 8, CW 14 |  | Mouse 22, Mouse 24 |
| Rv3723 |  | PROBABLE CONSERVED TRANSMEMBRANE PROTEIN | 35, 97, 108, 109 | Yes | TM | MEM 11, MEM 8, MEM 14, MEM 9, CW 19, CW 14, WCL 9 |  | Macrophage 20, Mouse 22 |
| Rv3724A | *cut5a* | PROBABLE CUTINASE PRECURSOR [FIRST PART] CUT5A | 33 |  | SP, |  |  |  |
| Rv3732 |  | CONSERVED HYPOTHETICAL PROTEIN | 34, 90, 117, 122, 127, 149 |  | SP, TM | CF 9, MEM 11, MEM 8, MEM 14, MEM 9, CW 19, CW 14, WCL 9 |  |  |
| Rv3737 |  | PROBABLE CONSERVED TRANSMEMBRANE PROTEIN | 228, 233, 238, 292 | Yes | TM | MEM 14 |  |  |
| Rv3756c | *proZ* | POSSIBLE OSMOPROTECTANT (GLYCINE BETAINE/CARNITINE/CHOLINE/L-PROLINE) TRANSPORT INTEGRAL MEMBRANE PROTEIN ABC TRANSPORTER PROZ | 109 |  | TM | MEM 8 |  |  |
| Rv3757c | *proW* | POSSIBLE OSMOPROTECTANT (GLYCINE BETAINE/CARNITINE/CHOLINE/L-PROLINE) TRANSPORT INTEGRAL MEMBRANE PROTEIN ABC TRANSPORTER PROW | 167, 180 |  | TM |  |  | Macrophage 21, Mouse 24 |
| Rv3759c | *proX* | POSSIBLE OSMOPROTECTANT (GLYCINE BETAINE/CARNITINE/CHOLINE/L-PROLINE) BINDING LIPOPROTEIN PROX | 36, 46, 126, 146, 149, 165, 181, 205, 248 | Yes | SP, Lipo | CF 8, CF 9, MEM 15, MEM 14, CW 14 |  | Mouse 24 |
| Rv3760 |  | POSSIBLE CONSERVED MEMBRANE PROTEIN | 44 |  | TM | MEM 15, MEM 14, CW 14 |  |  |
| Rv3763 | *lpqH* | 19 KDA LIPOPROTEIN ANTIGEN PRECURSOR LPQH | 19, 30, 35, 43, 45, 47, 61, 68, 70, 78, 82, 134 | Yes | SP, Lipo | CF 7, CF 8, CF 9, MEM 4, MEM 11, MEM 8, MEM 14, MEM 9, CW 15, CW 19, CW 14, WCL 9, SOL 9 | PhoA 17 |  |
| Rv3779 |  | PROBABLE CONSERVED TRANSMEMBRANE PROTEIN ALANINE AND LEUCINE RICH | 61, 64, 303, 360, 439, 546, 561, 657 | Yes | TM | MEM 14, CW 14 | BlaTEM 18 |  |
| Rv3789 |  | POSSIBLE CONSERVED INTEGRAL MEMBRANE PROTEIN | 103 |  | TM | MEM 8 |  | *in vitro* 22 |
| Rv3792 |  | PROBABLE CONSERVED TRANSMEMBRANE PROTEIN | 141, 338, 491 | Yes | TM | MEM 12, MEM 8, MEM 14, CW 15, CW 14 |  | *in vitro* 25, *in vitro* 22 |
| Rv3793 | *embC* | INTEGRAL MEMBRANE INDOLYLACETYLINOSITOL ARABINOSYLTRANSFERASE EMBC (ARABINOSYLINDOLYLACETYLINOSITOL SYNTHASE) | 55, 437, 662, 794, 819, 1028 |  | TM | CF 9, MEM 8, MEM 14, MEM 9, CW 19, CW 14 |  | Macaque 19, *in vitro* 25, *in vitro* 22 |
| Rv3794 | *embA* | INTEGRAL MEMBRANE INDOLYLACETYLINOSITOL ARABINOSYLTRANSFERASE EMBA (ARABINOSYLINDOLYLACETYLINOSITOL SYNTHASE) | 39, 49, 58, 63, 82, 144, 145, 156, 158, 182, 198, 319, 577, 587, 656, 727 | Yes | TM | MEM 11, MEM 8, MEM 14, MEM 9, CW 19, CW 14 | PhoA 17 | Macrophage 20, Mouse 22, *in vitro* 22 |
| Rv3795 | *embB* | INTEGRAL MEMBRANE INDOLYLACETYLINOSITOL ARABINOSYLTRANSFERASE EMBB (ARABINOSYLINDOLYLACETYLINOSITOL SYNTHASE) | 79, 197, 599 |  | TM | MEM 8, MEM 14, CW 14 |  | *in vitro* 25, *in vitro* 22 |
| Rv3796 | *atsH* | CONSERVED HYPOTHETICAL PROTEIN | 67, 68 |  | Tat SP | CF 8, MEM 8, MEM 14, CW 14 |  |  |
| Rv3802c |  | PROBABLE CONSERVED MEMBRANE PROTEIN | 36, 290, 291 |  | SP, TM | CF 8, CF 9, MEM 11, MEM 8, MEM 14, MEM 9, CW 14 | BlaTEM 18 | Mouse 24, *in vitro* 25, *in vitro* 22 |
| Rv3804c | *fbpA, mpt44, 85A* | SECRETED ANTIGEN 85-A FBPA (MYCOLYL TRANSFERASE 85A) (FIBRONECTIN-BINDING PROTEIN A) (ANTIGEN 85 COMPLEX A) | 39, 76, 127, 129, 160, 288, 330 | Yes | SP, Tat SP, TM | CF 7, CF 8, CF 9, MEM 4, MEM 15, MEM 8, MEM 14, MEM 9, CW 15, CW 19, CW 14, WCL 9, SOL 9 | PhoA 17 | Mouse 24, *in vitro* 22 |
| Rv3805c |  | PROBABLE CONSERVED TRANSMEMBRANE PROTEIN | 417 |  | TM | MEM 14, CW 14 |  | Mouse 22, *in vitro* 25, *in vitro* 22 |
| Rv3811 | *csp* | CONSERVED HYPOTHETICAL PROTEIN | 15, 38, 178 |  |  |  |  |  |
| Rv3821 |  | PROBABLE CONSERVED INTEGRAL MEMBRANE PROTEIN | 62, 74 |  | Tat SP, TM |  |  |  |
| Rv3822 |  |  | 72, 74, 90, 152, 185, 249, 325, 341, 358, 362, 368 | Yes |  | CW 14 |  |  |
| Rv3823c | *mmpL8* | PROBABLE CONSERVED INTEGRAL MEMBRANE TRANSPORT PROTEIN MMPL8 | 89, 440 |  | TM | CW 15 |  | Mouse 24, *in vitro* 22 |
| Rv3831 |  | HYPOTHETICAL PROTEIN | 29, 67, 74 | Yes | TM |  |  |  |
| Rv3835 |  | PROBABLE CONSERVED MEMBRANE PROTEIN | 62, 72, 133, 146, 182, 202, 226, 255, 375, 425 | Yes | TM | CF 8, CF 9, MEM 12, MEM 15, MEM 8, MEM 14, CW 15, CW 14 | BlaTEM 18 |  |
| Rv3851 |  | POSSIBLE MEMBRANE PROTEIN | 48 |  | SP, TM | MEM 8 |  |  |
| Rv3869 | *eccB1* | POSSIBLE CONSERVED MEMBRANE PROTEIN | 70, 88, 130, 463, 476 | Yes | TM | CF 8, CF 9, MEM 8, MEM 14, CW 15, CW 14, WCL 9, SOL 9 | BlaTEM 18 | Mouse 22 |
| Rv3877 | *eccD1* | PROBABLE CONSERVED TRANSMEMBRANE PROTEIN | 223, 234 |  | TM | MEM 8, MEM 14, CW 15, CW 14 |  | Mouse 22 |
| Rv3883c | *mycP1* | MEMBRANE-ANCHORED MYCOSIN MYCP1 (SERINE PROTEASE) (SUBTILISIN-LIKE PROTEASE) (SUBTILASE-LIKE) (MYCOSIN-1) | 17, 79, 188, 201, 252, 256, 392 | Yes | SP, TM | CF 8, MEM 8, MEM 14, CW 14 |  |  |
| Rv3886c | *mycP2* | PROBABLE ALANINE AND PROLINE RICH MEMBRANE-ANCHORED MYCOSIN MYCP2 (SERINE PROTEASE) (SUBTILISIN-LIKE PROTEASE) (SUBTILASE-LIKE) (MYCOSIN-2) | 170, 510 | Yes | SP, TM | CF 9, MEM 11, MEM 14, MEM 9, CW 19, CW 14 |  |  |
| Rv3887c | *eccD2* | PROBABLE CONSERVED TRANSMEMBRANE PROTEIN | 172, 445 |  | TM | MEM 14, CW 14 |  |  |
| Rv3895c | *eccB2* | PROBABLE CONSERVED MEMBRANE PROTEIN | 65, 79, 98, 103, 122 | Yes | TM | MEM 8, MEM 14, CW 14 |  |  |
| Rv3901c |  | POSSIBLE MEMBRANE PROTEIN | 54, 67 |  | SP, TM |  | BlaTEM 18 |  |
| Rv3909 |  | CONSERVED HYPOTHETICAL PROTEIN | 76, 243, 420 | Yes | SP, | MEM 12, MEM 8, MEM 14, CW 14 |  | *in vitro* 22 |
| Rv3910 |  | PROBABLE CONSERVED TRANSMEMBRANE PROTEIN | 70, 353, 372, 430, 1022, 1033, 1049 | Yes | Tat SP, TM | CF 9, MEM 4, MEM 11, MEM 12, MEM 8, MEM 14, MEM 9, CW 19, CW 14, SOL 9 | BlaTEM 18 | Mouse 22, *in vitro* 22 |
| Rv3912 |  | HYPOTHETICAL ALANINE RICH PROTEIN | 177 |  |  | MEM 14 |  | Macrophage 21 |

**Supplemental Table 1. EXIT Results: all 593 *in vivo* exported proteins**. Proteins identified as exported in EXIT in the spleen are identified by their genome identification number, name, and function from the NCBI genome annotation (H37Rv RefSeq genome annotation released January 9 2012). Column 4 identifies all statistically enriched fusion sites identified by EXIT as exported in the spleen by the amino acid position of fusion to the ‘BlaTEM reporter. Column 5 identifies whether a given protein was also identified as exported in the lungs. Column 6 identifies all *in silico* predicted export signals: signal peptide (SP[^1^](#_ENREF_1)), twin-arginine translocation signal peptide (Tat SP[^2^](#_ENREF_2)), lipoprotein signal peptide (Lipo^[3](#_ENREF_3)^), transmembrane domain (TM[^4^](#_ENREF_4)), or YxxxD/E motif [^5^](#_ENREF_5)^,^[^6^](#_ENREF_6). Proteins with no *in silico* predicted export signals are highlighted in grey. Column 7 identifies all previous *in vitro* mass spectrometry based published methodologies which identified a given protein as exported, with the fractions in which the protein was identified: culture filtrate (CF), membrane (MEM), cell wall (CW), soluble/cytoplasm (SOL), or whole cell lysate (WCL). Column 8 identifies all previous genetic reporter based experiments that identified a given protein as exported, with the genetic reporter identified. Column 9 identifies all studies where a given gene was identified as essential for growth *in vitro* or during infection. Numbers given relate to the references and conditions as described below.

Mass spectrometry based approaches to identify exported proteins in fractions: CF: [^7^](#_ENREF_7), [^8^](#_ENREF_8), [^9^](#_ENREF_9). MEM:[^10^](#_ENREF_10),[^11^](#_ENREF_11),[^12^](#_ENREF_12),[^13^](#_ENREF_13),[^8^](#_ENREF_8),[^14^](#_ENREF_14), [^9^](#_ENREF_9). CW: [^12^](#_ENREF_12), [^15^](#_ENREF_15), [^9^](#_ENREF_9). WCL: ^[9](#_ENREF_9)^. SOL: [^9^](#_ENREF_9). Genetic reporter based approaches to identify exported proteins: [^16^](#_ENREF_16),[^17^](#_ENREF_17),[^2^](#_ENREF_2),[^18^](#_ENREF_18). Genes identified as essential for virulence in models of tuberculosis: Macaque: [^19^](#_ENREF_19), Macrophage: [^20^](#_ENREF_20), [^21^](#_ENREF_21), Mouse: [^22^](#_ENREF_22), [^23^](#_ENREF_23), [^24^](#_ENREF_24). Genes identified as essential for *in vitro* growth: [^25^](#_ENREF_25), [^22^](#_ENREF_22).

References

1 Petersen, T. N., Brunak, S., von Heijne, G. & Nielsen, H. SignalP 4.0: discriminating signal peptides from transmembrane regions. *Nature methods* **8**, 785-786, doi:10.1038/nmeth.1701 (2011).

2 McDonough, J. A. *et al.* Identification of functional Tat signal sequences in *Mycobacterium tuberculosis* proteins. *J Bacteriol* **190**, 6428-6438, doi:JB.00749-08 [pii]10.1128/JB.00749-08 (2008).

3 Sutcliffe, I. C. & Harrington, D. J. Lipoproteins of *Mycobacterium tuberculosis*: an abundant and functionally diverse class of cell envelope components. *FEMS Microbiol Rev* **28**, 645-659 (2004).

4 Krogh, A., Larsson, B., von Heijne, G. & Sonnhammer, E. L. Predicting transmembrane protein topology with a hidden Markov model: application to complete genomes. *J Mol Biol* **305**, 567-580, doi:10.1006/jmbi.2000.4315S0022-2836(00)94315-8 [pii] (2001).

5 Daleke, M. H. *et al.* General secretion signal for the mycobacterial type VII secretion pathway. *Proc Natl Acad Sci U S A* **109**, 11342-11347, doi:10.1073/pnas.1119453109 (2012).

6 Abdallah, A. M. *et al.* PPE and PE_PGRS proteins of *Mycobacterium marinum* are transported via the type VII secretion system ESX-5. *Mol Microbiol* **73**, 329-340, doi:MMI6783 [pii]10.1111/j.1365-2958.2009.06783.x (2009).

7 Rosenkrands, I. *et al.* Mapping and identification of *Mycobacterium tuberculosis* proteins by two-dimensional gel electrophoresis, microsequencing and immunodetection. *Electrophoresis* **21**, 935-948 (2000).

8 Malen, H., De Souza, G. A., Pathak, S., Softeland, T. & Wiker, H. G. Comparison of membrane proteins of *Mycobacterium tuberculosis* H37Rv and H37Ra strains. *BMC Microbiol* **11**, 18, doi:10.1186/1471-2180-11-18 (2011).

9 Bell, C., Smith, G. T., Sweredoski, M. J. & Hess, S. Characterization of the *Mycobacterium tuberculosis* proteome by liquid chromatography mass spectrometry-based proteomics techniques: a comprehensive resource for tuberculosis research. *J Proteome Res* **11**, 119-130, doi:10.1021/pr2007939 (2012).

10 Gu, S. *et al.* Comprehensive Proteomic Profiling of the Membrane Constituents of a *Mycobacterium tuberculosis* Strain. *Molecular & cellular proteomics : MCP* **2**, 1284-1296 (2003).

11 Xiong, Y., Chalmers, M. J., Gao, F. P., Cross, T. A. & Marshall, A. G. Identification of *Mycobacterium tuberculosis* H37Rv integral membrane proteins by one-dimensional gel electrophoresis and liquid chromatography electrospray ionization tandem mass spectrometry. *J Proteome Res* **4**, 855-861, doi:10.1021/pr0500049 (2005).

12 Mawuenyega, K. G. *et al.* *Mycobacterium tuberculosis* functional network analysis by global subcellular protein profiling. *Mol Biol Cell* **16**, 396-404, doi:10.1091/mbc.E04-04-0329 (2005).

13 Malen, H., Berven, F. S., Fladmark, K. E. & Wiker, H. G. Comprehensive analysis of exported proteins from *Mycobacterium tuberculosis* H37Rv. *Proteomics* **7**, 1702-1718 (2007).

14 Gunawardena, H. P. *et al.* Comparison of the membrane proteome of virulent *Mycobacterium tuberculosis* and the attenuated *Mycobacterium bovis* BCG vaccine strain by label-free quantitative proteomics. *J Proteome Res* **12**, 5463-5474, doi:10.1021/pr400334k (2013).

15 Wolfe, L. M., Mahaffey, S. B., Kruh, N. A. & Dobos, K. M. Proteomic definition of the cell wall of *Mycobacterium tuberculosis*. *J Proteome Res* **9**, 5816-5826, doi:10.1021/pr1005873 (2010).

16 Gomez, M., Johnson, S. & Gennaro, M. L. Identification of secreted proteins of *Mycobacterium tuberculosis* by a bioinformatic approach. *Infect Immun* **68**, 2323-2327 (2000).

17 Braunstein, M. *et al.* Identification of genes encoding exported *Mycobacterium tuberculosis* proteins using a Tn552'phoA in vitro transposition system. *J Bacteriol* **182**, 2732-2740 (2000).

18 McCann, J. R., McDonough, J. A., Sullivan, J. T., Feltcher, M. E. & Braunstein, M. Genome-wide identification of *Mycobacterium tuberculosis* exported proteins with roles in intracellular growth. *J Bacteriol* **193**, 854-861, doi:JB.01271-10 [pii]10.1128/JB.01271-10 (2011).

19 Dutta, N. K. *et al.* Genetic requirements for the survival of tubercle bacilli in primates. *J Infect Dis* **201**, 1743-1752, doi:10.1086/652497 (2010).

20 Rengarajan, J., Bloom, B. R. & Rubin, E. J. Genome-wide requirements for *Mycobacterium tuberculosis* adaptation and survival in macrophages. *Proc Natl Acad Sci U S A* **102**, 8327-8332 (2005).

21 Stewart, G. R., Patel, J., Robertson, B. D., Rae, A. & Young, D. B. Mycobacterial mutants with defective control of phagosomal acidification. *PLoS Pathog* **1**, 269-278 (2005).

22 Sassetti, C. M. & Rubin, E. J. Genetic requirements for mycobacterial survival during infection. *Proc Natl Acad Sci U S A* **100**, 12989-12994 (2003).

23 Lamichhane, G., Tyagi, S. & Bishai, W. R. Designer arrays for defined mutant analysis to detect genes essential for survival of *Mycobacterium tuberculosis* in mouse lungs. *Infect Immun* **73**, 2533-2540 (2005).

24 Zhang, Y. J. *et al.* Tryptophan biosynthesis protects mycobacteria from CD4 T-cell-mediated killing. *Cell* **155**, 1296-1308, doi:10.1016/j.cell.2013.10.045 (2013).

25 Sassetti, C. M., Boyd, D. H. & Rubin, E. J. Genes required for mycobacterial growth defined by high density mutagenesis. *Mol Microbiol* **48**, 77-84 (2003).
