# Supplementary material for: The EXIT Strategy: an Approach for Identifying Bacterial Proteins Exported during Host Infection
Source: mBio. 2017 Apr 25;8(2):e00333-17. doi: 10.1128/mBio.00333-17 (PMC5405230; doi:10.1128/mBio.00333-17)
Supplement: TABLE S2 [file mbo002173284st2.docx]

| **Supplemental Table 2. EXIT proteins lacking *in silico* predicted export signals** | | | |
| --- | --- | --- | --- |
| **ORF number** | **Name** | **Product** | **Subcellular localization by Mass Spec** |
| Rv0518 |  | POSSIBLE EXPORTED PROTEIN | CF ([1](#_ENREF_1)), CF ([2](#_ENREF_2)), MEM ([1](#_ENREF_1)) |
| Rv0787 |  | HYPOTHETICAL PROTEIN | CF ([1](#_ENREF_1)), CF ([2](#_ENREF_2)), MEM ([3](#_ENREF_3)), MEM ([4](#_ENREF_4)) |
| Rv0822c |  | CONSERVED HYPOTHETICAL PROTEIN | CF ([1](#_ENREF_1)), CW ([5](#_ENREF_5)) |
| Rv0907 |  | CONSERVED HYPOTHETICAL PROTEIN | CF ([1](#_ENREF_1)), CF ([2](#_ENREF_2)), MEM ([6](#_ENREF_6)), MEM ([1](#_ENREF_1)), MEM ([4](#_ENREF_4)), CW ([5](#_ENREF_5)) |
| Rv0950c |  | CONSERVED HYPOTHETICAL PROTEIN |  |
| Rv1026 |  | CONSERVED HYPOTHETICAL PROTEIN |  |
| Rv1728c |  | CONSERVED HYPOTHETICAL PROTEIN |  |
| Rv1823 |  | CONSERVED HYPOTHETICAL PROTEIN | MEM ([1](#_ENREF_1)), MEM ([4](#_ENREF_4)), CW ([5](#_ENREF_5)) |
| Rv1832 | *gcvB* | PROBABLE GLYCINE DEHYDROGENASE (GLYCINE CLEAVAGE SYSTEM P-PROTEIN) | CF ([1](#_ENREF_1)), MEM ([7](#_ENREF_7)), MEM ([1](#_ENREF_1)), MEM ([4](#_ENREF_4)), WCL ([2](#_ENREF_2)), SOL ([2](#_ENREF_2)) |
| Rv1887 |  | HYPOTHETICAL PROTEIN | CF ([2](#_ENREF_2)), MEM ([3](#_ENREF_3)) |
| Rv1891 |  | CONSERVED HYPOTHETICAL PROTEIN | CF ([1](#_ENREF_1)), CF ([2](#_ENREF_2)), MEM ([3](#_ENREF_3)), MEM ([1](#_ENREF_1)) |
| Rv2088 | *pknJ* | PROBABLE TRANSMEMBRANE SERINE/THREONINE-PROTEIN KINASE J PKNJ (PROTEIN KINASE J) (STPK J) | MEM ([1](#_ENREF_1)), MEM ([4](#_ENREF_4)), CW ([5](#_ENREF_5)) |
| Rv2240c |  | HYPOTHETICAL PROTEIN | CF ([1](#_ENREF_1)), CF ([2](#_ENREF_2)), MEM ([1](#_ENREF_1)), MEM ([4](#_ENREF_4)), CW ([5](#_ENREF_5)) |
| Rv2264c |  | CONSERVED HYPOTHETICAL PROLINE RICH PROTEIN | CW ([5](#_ENREF_5)) |
| Rv2300c |  | CONSERVED HYPOTHETICAL PROTEIN | MEM ([1](#_ENREF_1)), MEM ([4](#_ENREF_4)) |
| Rv2380c | *mbtE* | PEPTIDE SYNTHETASE MBTE (PEPTIDE SYNTHASE) | MEM ([7](#_ENREF_7)), MEM ([4](#_ENREF_4)), CW ([2](#_ENREF_2)), SOL ([2](#_ENREF_2)) |
| Rv3035 |  | CONSERVED HYPOTHETICAL PROTEIN | MEM ([1](#_ENREF_1)), MEM ([4](#_ENREF_4)), CW ([5](#_ENREF_5)) |
| Rv3067 |  | CONSERVED HYPOTHETICAL PROTEIN | CF ([1](#_ENREF_1)) |
| Rv3123 |  | HYPOTHETICAL PROTEIN | MEM ([4](#_ENREF_4)) |
| Rv3274c | *fadE25* | PROBABLE ACYL-CoA DEHYDROGENASE FADE25 | CF ([1](#_ENREF_1)), CF ([2](#_ENREF_2)), MEM ([7](#_ENREF_7)), MEM ([6](#_ENREF_6)), MEM ([1](#_ENREF_1)), MEM ([4](#_ENREF_4)), MEM ([2](#_ENREF_2)), CW ([8](#_ENREF_8)), CW ([2](#_ENREF_2)), CW ([5](#_ENREF_5)), WCL ([2](#_ENREF_2)), SOL ([2](#_ENREF_2)) |
| Rv3343c | *PPE54* | PPE FAMILY PROTEIN |  |
| Rv3350c | *PPE56* | PPE FAMILY PROTEIN |  |
| Rv3478 | *PPE60, mtb39c* | PE FAMILY PROTEIN | MEM ([1](#_ENREF_1)), MEM ([4](#_ENREF_4)), CW ([2](#_ENREF_2)), CW ([5](#_ENREF_5)) |
| Rv3526 |  | POSSIBLE OXIDOREDUCTASE |  |
| Rv3596c | *clpC1* | PROBABLE ATP-DEPENDENT PROTEASE ATP-BINDING SUBUNIT CLPC1 | CF ([2](#_ENREF_2)), MEM ([7](#_ENREF_7)), MEM ([9](#_ENREF_9)), MEM ([1](#_ENREF_1)), MEM ([4](#_ENREF_4)), MEM ([2](#_ENREF_2)), CW ([8](#_ENREF_8)), CW ([2](#_ENREF_2)), CW ([5](#_ENREF_5)), WCL ([2](#_ENREF_2)), SOL ([2](#_ENREF_2)) |
| Rv3654c |  | CONSERVED HYPOTHETICAL PROTEIN |  |
| Rv3667 | *acs* | ACETYL-COENZYME A SYNTHETASE ACS (ACETATE--CoA LIGASE) (ACYL-ACTIVATING ENZYME) (ACETATE THIOKINASE) (ACETATE--COENZYME A LIGASE) | MEM ([7](#_ENREF_7)), MEM ([4](#_ENREF_4)), CW ([2](#_ENREF_2)), CW ([5](#_ENREF_5)), WCL ([2](#_ENREF_2)), SOL ([2](#_ENREF_2)) |
| Rv3691 |  | CONSERVED HYPOTHETICAL PROTEIN | MEM ([7](#_ENREF_7)), MEM ([1](#_ENREF_1)), MEM ([4](#_ENREF_4)), CW ([5](#_ENREF_5)) |
| Rv3707c |  | CONSERVED HYPOTHETICAL PROTEIN | MEM ([4](#_ENREF_4)) |
| Rv3811 | *csp* | CONSERVED HYPOTHETICAL PROTEIN |  |
| Rv3822 |  | CONSERVED HYPOTHETICAL PROTEIN | CW ([5](#_ENREF_5)) |
| Rv3912 |  | HYPOTHETICAL ALANINE RICH PROTEIN | MEM ([4](#_ENREF_4)) |

**Supplemental Table 2. EXIT proteins lacking *in silico* predicted export signals**. Proteins identified as exported by EXIT were analyzed for export signals by SignalP([10](#_ENREF_10)), and TMHMM([11](#_ENREF_11)), as well as for Tat and lipoprotein signal peptides([12](#_ENREF_12)), ([13](#_ENREF_13)). Proteins are identified by their genome designation (column 1), name (column 2), and annotation from the H37Rv RefSeq genome annotation released January 9 2012. Column 4 identifies all previous mass spectrometry based proteomics methods that reported the protein as being localized to specific subcellular fractions: culture filtrate (CF), membrane (MEM), cell wall (CW), soluble cytoplasm (SOL), or whole cell lysate (WCL). Mass spectrometry based approaches to identify exported proteins in fractions: CF: ([1](#_ENREF_1), [2](#_ENREF_2)). MEM: ([1-4](#_ENREF_1), [6](#_ENREF_6), [7](#_ENREF_7), [9](#_ENREF_9)). CW: ([2](#_ENREF_2), [5](#_ENREF_5), [8](#_ENREF_8), [9](#_ENREF_9)). WCL: ([2](#_ENREF_2)). SOL: ([2](#_ENREF_2)).

**References**

1. Malen H, De Souza GA, Pathak S, Softeland T, Wiker HG. 2011. Comparison of membrane proteins of *Mycobacterium tuberculosis* H37Rv and H37Ra strains. BMC Microbiol 11:18.

2. Bell C, Smith GT, Sweredoski MJ, Hess S. 2012. Characterization of the *Mycobacterium tuberculosis* proteome by liquid chromatography mass spectrometry-based proteomics techniques: a comprehensive resource for tuberculosis research. J Proteome Res 11:119-30.

3. Malen H, Berven FS, Fladmark KE, Wiker HG. 2007. Comprehensive analysis of exported proteins from *Mycobacterium tuberculosis* H37Rv. Proteomics 7:1702-18.

4. Gunawardena HP, Feltcher ME, Wrobel JA, Gu S, Braunstein M, Chen X. 2013. Comparison of the membrane proteome of virulent *Mycobacterium tuberculosis* and the attenuated *Mycobacterium bovis* BCG vaccine strain by label-free quantitative proteomics. J Proteome Res 12:5463-74.

5. Feltcher ME, Gunawardena HP, Zulauf KE, Malik S, Griffin JE, Sassetti CM, Chen X, Braunstein M. 2015. Label-free quantitative proteomics reveals a role for the *Mycobacterium tuberculosis* SecA2 pathway in exporting solute binding proteins and Mce transporters to the cell wall. Mol Cell Proteomics doi:10.1074/mcp.M114.044685.

6. Xiong Y, Chalmers MJ, Gao FP, Cross TA, Marshall AG. 2005. Identification of *Mycobacterium tuberculosis* H37Rv integral membrane proteins by one-dimensional gel electrophoresis and liquid chromatography electrospray ionization tandem mass spectrometry. J Proteome Res 4:855-61.

7. Gu S, Chen J, Dobos KM, Bradbury EM, Belisle JT, Chen X. 2003. Comprehensive Proteomic Profiling of the Membrane Constituents of a *Mycobacterium tuberculosis* Strain. Mol Cell Proteomics 2:1284-1296.

8. Wolfe LM, Mahaffey SB, Kruh NA, Dobos KM. 2010. Proteomic definition of the cell wall of *Mycobacterium tuberculosis*. J Proteome Res 9:5816-26.

9. Mawuenyega KG, Forst CV, Dobos KM, Belisle JT, Chen J, Bradbury EM, Bradbury AR, Chen X. 2005. *Mycobacterium tuberculosis* functional network analysis by global subcellular protein profiling. Mol Biol Cell 16:396-404.

10. Petersen TN, Brunak S, von Heijne G, Nielsen H. 2011. SignalP 4.0: discriminating signal peptides from transmembrane regions. Nat Methods 8:785-6.

11. Krogh A, Larsson B, von Heijne G, Sonnhammer EL. 2001. Predicting transmembrane protein topology with a hidden Markov model: application to complete genomes. J Mol Biol 305:567-80.

12. McDonough JA, McCann JR, Tekippe EM, Silverman JS, Rigel NW, Braunstein M. 2008. Identification of functional Tat signal sequences in *Mycobacterium tuberculosis* proteins. J Bacteriol 190:6428-38.

13. Sutcliffe IC, Harrington DJ. 2004. Lipoproteins of *Mycobacterium tuberculosis*: an abundant and functionally diverse class of cell envelope components. FEMS Microbiol Rev 28:645-59.
