# Supplementary material for: The EXIT Strategy: an Approach for Identifying Bacterial Proteins Exported during Host Infection
Source: mBio. 2017 Apr 25;8(2):e00333-17. doi: 10.1128/mBio.00333-17 (PMC5405230; doi:10.1128/mBio.00333-17)
Supplement: TABLE S3 [file mbo002173284st3.docx]

| **Supplemental Table 3. EXIT proteins only identified in the lungs** | | | |
| --- | --- | --- | --- |
| **ORF number** | **Name** | **Product** | ***In silico* export signal** |
| Rv0187 |  | PROBABLE O-METHYLTRANSFERASE |  |
| Rv0578c | *PE_PGRS7* | PE-PGRS FAMILY PROTEIN | SP, YxxxD/E |
| Rv1091 | *PE_PGRS*  *22* | PE-PGRS FAMILY PROTEIN | YxxxD/E |
| Rv1371 |  | PROBABLE CONSERVED MEMBRANE PROTEIN | TM |
| Rv1818c | *PE_PGRS33* | PE-PGRS FAMILY PROTEIN | SP, YxxxD/E |
| Rv2196 | *qcrB* | Probable Ubiquinol-cytochrome C reductase QcrB (cytochrome B subunit) | TM |
| Rv2490c | *PE_PGRS43* | PE-PGRS FAMILY PROTEIN | SP, YxxxD/E |
| Rv3101c | *ftsX* | PUTATIVE CELL DIVISION PROTEIN FTSX (SEPTATION COMPONENT-TRANSPORT INTEGRAL MEMBRANE PROTEIN ABC TRANSPORTER) | TM |

**Supplemental Table 3. EXIT exported proteins only identified in the lungs**. Genes that displayed 3.5 fold higher abundance in the lungs after *in vivo* β-lactam treatment than the input in both experiments were identified as exported *in vivo* in the lungs. A total of 282 proteins were identified as exported in the lungs. The eight proteins reported above were only identified as exported in the lungs and not identified as exported in the spleen.
