# Supplementary material for: The EXIT Strategy: an Approach for Identifying Bacterial Proteins Exported during Host Infection
Source: mBio. 2017 Apr 25;8(2):e00333-17. doi: 10.1128/mBio.00333-17 (PMC5405230; doi:10.1128/mBio.00333-17)
Supplement: FIG S3 [file mbo002173284sf3.pdf]

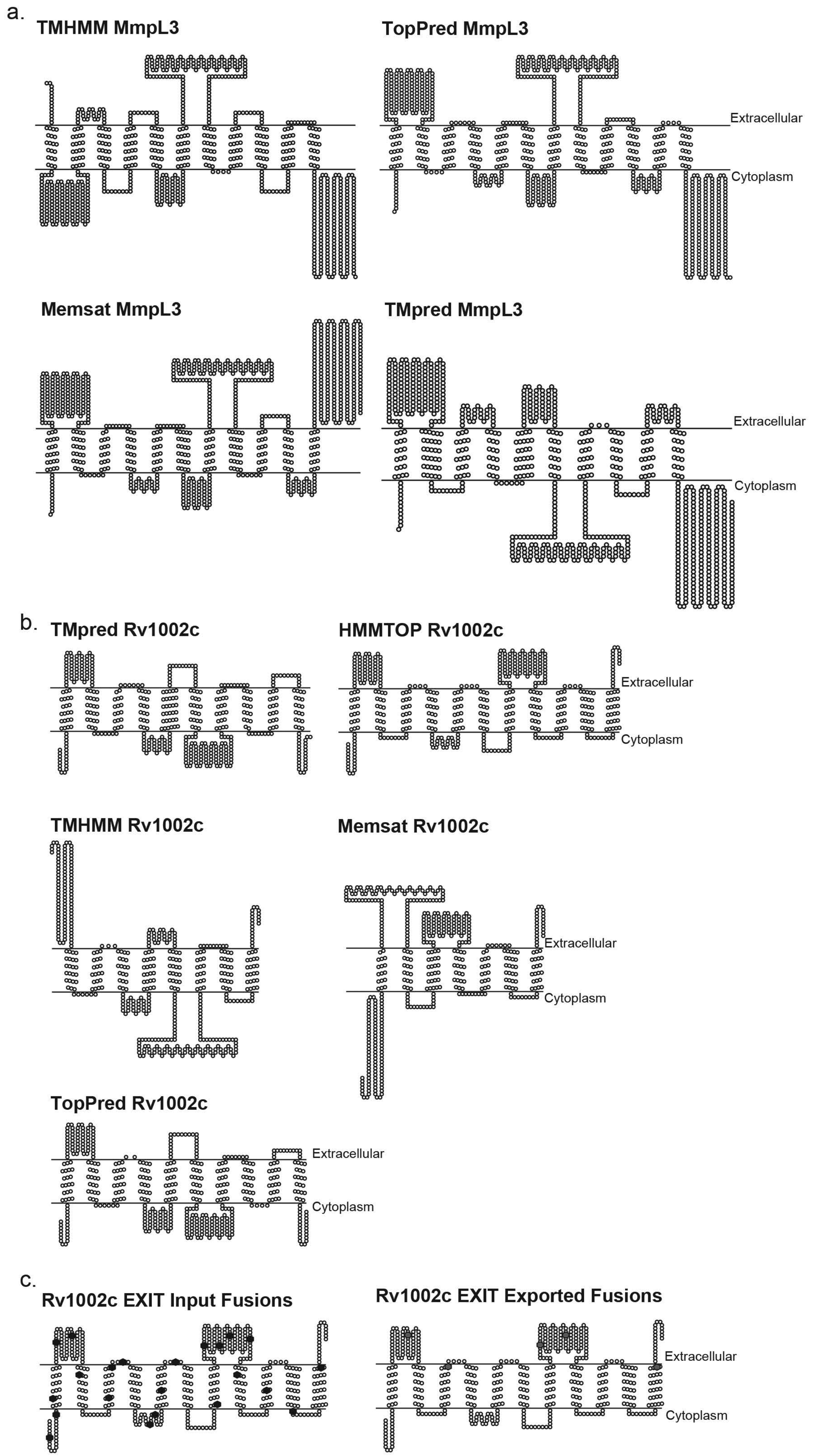
a.
TMHMM MmpL3
TopPred MmpL3
Extracellular
Cytoplasm
Memsat MmpL3
TMpred MmpL3
Extracellular
Cytoplasm
b.
TMpred Rv1002c
HMMTOP Rv1002c
Extracellular
Cytoplasm
TMHMM Rv1002c
Memsat Rv1002c
Extracellular
Cytoplasm
TopPred Rv1002c
Extracellular
Cytoplasm
c.
Rv1002c EXIT Input Fusions
Rv1002c EXIT Exported Fusions
Extracellular
Cytoplasm

References for Supplemental Figure 3

1. Claros MG, von Heijne G. 1994. TopPred II: an improved software for membrane protein structure predictions. Comput Appl Biosci 10:685-6.
2. Krogh A, Larsson B, von Heijne G, Sonnhammer EL. 2001. Predicting transmembrane protein topology with a hidden Markov model: application to complete genomes. J Mol Biol 305:567-80.
3. Hofmann K, Stoffel W. 1993. TMbase - A database of membrane spanning proteins segments. Biol Chem Hoppe-Seyler 374,:166-170.
4. Jones DT, Taylor WR, Thornton JM. 1994. A model recognition approach to the prediction of all-helical membrane protein structure and topology. Biochemistry 33:3038-49.
5. Li W, Upadhyay A, Fontes FL, North EJ, Wang Y, Crans DC, Grzegorzewicz AE, Jones V, Franzblau SG, Lee RE, Crick DC, Jackson M. 2014. Novel insights into the mechanism of inhibition of MmpL3, a target of multiple pharmacophores in *Mycobacterium tuberculosis*. Antimicrob Agents Chemother 58:6413-23.
6. Rayasam GV. 2014. MmpL3 a potential new target for development of novel anti-tuberculosis drugs. Expert Opin Ther Targets 18:247-56.
7. Remuinan MJ, Perez-Herran E, Rullas J, Alemparte C, Martinez-Hoyos M, Dow DJ, Afari J, Mehta N, Esquivias J, Jimenez E, Ortega-Muro F, Fraile-Gabaldon MT, Spivey VL, Loman NJ, Pallen MJ, Constantinidou C, Minick DJ, Cacho M, Rebollo-Lopez MJ, Gonzalez C, Sousa V, Angulo-Barturen I, Mendoza-Losana A, Barros D, Besra GS, Ballell L, Cammack N. 2013. Tetrahydropyrazolo[1,5-a]pyrimidine-3-carboxamide and N-benzyl-6',7'-dihydrospiro[piperidine-4,4'-thieno[3,2-c]pyran] analogues with bactericidal efficacy against *Mycobacterium tuberculosis* targeting MmpL3. PLoS One 8:e60933.
8. Poce G, Bates RH, Alfonso S, Cocozza M, Porretta GC, Ballell L, Rullas J, Ortega F, De Logu A, Agus E, La Rosa V, Pasca MR, De Rossi E, Wae B, Franzblau SG, Manetti F, Botta M, Biava M. 2013. Improved BM212 MmpL3 inhibitor analogue shows efficacy in acute murine model of tuberculosis infection. PLoS One 8:e56980.

9. Tahlan K, Wilson R, Kastrinsky DB, Arora K, Nair V, Fischer E, Barnes SW, Walker JR, Alland D, Barry CE, 3rd, Boshoff HI. 2012. SQ109 targets MmpL3, a membrane transporter of trehalose monomycolate involved in mycolic acid donation to the cell wall core of *Mycobacterium tuberculosis*. Antimicrob Agents Chemother 56:1797-809.

10. La Rosa V, Poce G, Canseco JO, Buroni S, Pasca MR, Biava M, Raju RM, Porretta GC, Alfonso S, Battilocchio C, Javid B, Sorrentino F, Ioerger TR, Sacchettini JC, Manetti F, Botta M, De Logu A, Rubin EJ, De Rossi E. 2012. MmpL3 is the cellular target of the antitubercular pyrrole derivative BM212. Antimicrob Agents Chemother 56:324-31.

11. Sandhu P, Akhter Y. 2015. The internal gene duplication and interrupted coding sequences in the MmpL genes of *Mycobacterium tuberculosis*: Towards understanding the multidrug transport in an evolutionary perspective. Int J Med Microbiol doi:10.1016/j.ijmm.2015.03.005.

12. Varela C, Rittmann D, Singh A, Krumbach K, Bhatt K, Eggeling L, Besra GS, Bhatt A. 2012. MmpL genes are associated with mycolic acid metabolism in mycobacteria and corynebacteria. Chem Biol 19:498-506.

13. Tusnady GE, Simon I. 2001. The HMMTOP transmembrane topology prediction server. Bioinformatics 17:849-50.
