# Supplementary material for: The EXIT Strategy: an Approach for Identifying Bacterial Proteins Exported during Host Infection
Source: mBio. 2017 Apr 25;8(2):e00333-17. doi: 10.1128/mBio.00333-17 (PMC5405230; doi:10.1128/mBio.00333-17)
Supplement: TABLE S4 [file mbo002173284st4.docx]

| **Supplemental Table 4. EXIT Input library fusions (all *M. tuberculosis* proteins)** | | | |
| --- | --- | --- | --- |
| **ORF number** | **Name** | **Product** | **Input library fusion junctions (aa location)** |
| Rv0001 | *dnaA* | chromosomal replication initiation protein | 159, 161, 187, 196, 209, 379, 419, 431, 433 |
| Rv0002 | *dnaN* | DNA polymerase III subunit beta | 47, 56, 63, 110, 135, 153, 154, 164, 178, 213, 219, 269, 326, 368 |
| Rv0003 | *recF* | recombination protein F | 128, 198, 236, 287, 344, 352, 359, 360, 376 |
| Rv0004 | *-* | hypothetical protein Rv0004 | 11, 38, 42, 56, 78, 86, 148, 162, 177 |
| Rv0005 | *gyrB* | DNA gyrase subunit B | 68, 75, 78, 97, 111, 120, 129, 143, 144, 215, 317, 321, 333, 351, 384, 393, 477, 501, 502, 507, 520, 549, 635, 679, 681 |
| Rv0006 | *gyrA* | DNA gyrase subunit A | 61, 178, 229, 230, 239, 247, 253, 263, 296, 391, 418, 443, 538, 591, 614, 645, 666, 734, 744, 758, 778, 779, 785 |
| Rv0007 | *-* | POSSIBLE CONSERVED MEMBRANE PROTEIN | 31, 74, 76, 86, 93, 96, 117, 129, 140, 145, 158, 161, 167, 183, 227, 247, 256, 261, 275, 293 |
| Rv0008c | *-* | POSSIBLE MEMBRANE PROTEIN | 43, 47, 49, 66, 76, 114, 115 |
| Rv0009 | *ppiA* | PROBABLE IRON-REGULATED PEPTIDYL-PROLYL CIS-TRANS ISOMERASE A PPIA (PPIase A) (ROTAMASE A) | 13, 15, 22, 61, 85, 89, 92, 117 |
| Rv0010c | *-* | PROBABLE CONSERVED MEMBRANE PROTEIN | 15, 17, 34, 39, 42, 107, 130, 132, 135 |
| Rv0011c | *-* | putative septation inhibitor protein | 83 |
| Rv0012 | *-* | PROBABLE CONSERVED MEMBRANE PROTEIN | 39, 46, 93, 102, 106, 160, 199, 204 |
| Rv0013 | *trpG* | para-aminobenzoate synthase component II | 40, 43, 53, 152, 161, 179, 191, 209, 219 |
| Rv0014c | *pknB* | TRANSMEMBRANE SERINE/THREONINE-PROTEIN KINASE B PKNB (PROTEIN KINASE B) (STPK B) | 62, 76, 82, 83, 165, 172, 210, 216, 243, 300, 304, 353, 369, 404, 421, 445, 446, 529, 530, 534, 566, 583, 594, 611 |
| Rv0015c | *pknA* | TRANSMEMBRANE SERINE/THREONINE-PROTEIN KINASE A PKNA (PROTEIN KINASE A) (STPK A) | 20, 64, 109, 124, 133, 151, 173, 179, 217, 256, 275, 291, 334, 342, 362, 378, 387, 400 |
| Rv0016c | *pbpA* | PROBABLE PENICILLIN-BINDING PROTEIN PBPA | 29, 51, 55, 87, 97, 124, 129, 144, 168, 204, 230, 233, 234, 254, 261, 265, 283, 292, 329, 352, 358, 395, 416, 426, 447, 467, 469 |
| Rv0017c | *rodA* | PROBABLE CELL DIVISION PROTEIN RODA | 28, 34, 64, 147, 158, 178, 184, 204, 210, 307, 321, 326, 337, 346, 387, 388, 403, 412, 422, 460 |
| Rv0018c | *ppp* | POSSIBLE SERINE/THREONINE PHOSPHATASE PPP | 15, 44, 54, 64, 74, 78, 101, 119, 166, 181, 223, 225, 250, 265, 267, 332, 397, 401, 432, 460, 465, 480, 496 |
| Rv0019c | *-* | hypothetical protein Rv0019c | 10, 35, 42, 109 |
| Rv0020c | *TB39.8* | hypothetical protein Rv0020c | 82, 115, 121, 163, 193, 198, 206, 235, 241, 247, 253, 282, 286, 291, 300, 307, 309, 312, 315, 351, 370, 392, 397, 398, 417, 421, 426, 430, 431, 443, 458, 469 |
| Rv0021c | *-* | hypothetical protein Rv0021c | 18, 25, 26, 31, 44, 59, 66, 98, 107, 108, 140, 162, 172, 173, 183, 190, 191, 286, 287, 290, 300, 305, 316 |
| Rv0022c | *whiB5* | PROBABLE TRANSCRIPTIONAL REGULATORY PROTEIN WHIB-LIKE WHIB5 | 23, 67, 72, 73, 93, 94, 121, 137 |
| Rv0023 | *-* | POSSIBLE TRANSCRIPTIONAL REGULATORY PROTEIN | 5, 25, 39, 69, 81, 93, 151, 161, 166, 180, 210 |
| Rv0024 | *-* | PUTATIVE SECRETED PROTEIN P60-RELATED PROTEIN | 16, 69, 71, 76, 89, 166, 169, 175, 197, 240 |
| Rv0025 | *-* | hypothetical protein Rv0025 | 4, 94 |
| Rv0026 | *-* | hypothetical protein Rv0026 | 23, 31, 39, 78, 96, 130, 142, 164, 173, 177, 196, 204, 207, 209, 211, 249, 265, 272, 280, 291, 295, 340, 364, 371, 379, 399, 407, 419 |
| Rv0027 | *-* | hypothetical protein Rv0027 | 14, 21, 52, 82 |
| Rv0028 | *-* | hypothetical protein Rv0028 | 5, 13, 22, 50, 74 |
| Rv0029 | *-* | hypothetical protein Rv0029 | 37, 48, 51, 127, 129, 130, 148, 159, 224, 242, 253, 268, 295, 299, 313, 337 |
| Rv0030 | *-* | hypothetical protein Rv0030 | 73, 104 |
| Rv0031 | *-* | POSSIBLE REMNANT OF A TRANSPOSASE |  |
| Rv0032 | *bioF2* | POSSIBLE 8-AMINO-7-OXONONANOATE SYNTHASE BIOF2 (AONS) (8-AMINO-7-KETOPELARGONATE SYNTHASE) (7-KETO-8-AMINO-PELARGONIC ACID SYNTHETASE) (7-KAP SYNTHETASE) (L-ALANINE--PIMELYL CoA LIGASE) | 77, 103, 392, 402, 411, 447, 476, 483, 498, 515, 519, 597, 620, 624, 643, 649, 673, 680, 694, 717, 757, 768 |
| Rv0033 | *acpA* | PROBABLE ACYL CARRIER PROTEIN ACPA (ACP) | 16, 19, 75 |
| Rv0034 | *-* | hypothetical protein Rv0034 | 14, 18, 47, 59, 61, 93, 108 |
| Rv0035 | *fadD34* | PROBABLE FATTY-ACID-CoA LIGASE FADD34 (FATTY-ACID-CoA SYNTHETASE) (FATTY-ACID-CoA SYNTHASE) | 55, 62, 125, 157, 159, 180, 184, 230, 260, 282, 313, 317, 354, 360, 361, 374, 380, 386, 391, 397, 450, 495, 509, 517 |
| Rv0036c | *-* | hypothetical protein Rv0036c | 62, 69, 71, 76, 82, 154, 194, 210, 212, 249, 252, 254 |
| Rv0037c | *-* | PROBABLE CONSERVED INTEGRAL MEMBRANE PROTEIN | 53, 87, 105, 112, 122, 172, 189, 195, 239, 256, 259, 283, 292, 300, 313, 331, 338, 339, 349, 356, 377, 399, 422, 433 |
| Rv0038 | *-* | hypothetical protein Rv0038 | 13, 48, 61, 139, 142 |
| Rv0039c | *-* | POSSIBLE CONSERVED TRANSMEMBRANE PROTEIN | 14, 45, 50, 51, 58, 60, 63, 80, 95, 99 |
| Rv0040c | *mtc28* | SECRETED PROLINE RICH PROTEIN MTC28 (PROLINE RICH 28 KDA ANTIGEN) | 12, 16, 25, 26, 61, 73, 85, 88, 99, 102, 178, 235, 248, 275, 283, 291, 299 |
| Rv0041 | *leuS* | leucyl-tRNA synthetase | 5, 9, 27, 121, 129, 203, 210, 218, 255, 280, 361, 375, 384, 391, 405, 432, 436, 465, 466, 472, 504, 507, 648, 656, 705, 717, 801, 838, 861, 901, 926, 936, 960 |
| Rv0042c | *-* | POSSIBLE TRANSCRIPTIONAL REGULATORY PROTEIN (PROBABLY MARR-FAMILY) | 47, 71, 79, 105, 164 |
| Rv0043c | *-* | PROBABLE TRANSCRIPTIONAL REGULATORY PROTEIN (PROBABLY GNTR-FAMILY) | 22, 27, 66, 97, 179, 222 |
| Rv0044c | *-* | POSSIBLE OXIDOREDUCTASE | 104, 105, 165, 167, 187, 188, 216, 221, 232, 254 |
| Rv0045c | *-* | POSSIBLE HYDROLASE | 7, 22, 45, 56, 86, 123, 131, 158, 162, 186, 198, 236, 243, 255, 291 |
| Rv0046c | *ino1* | MYO-INOSITOL-1-PHOSPHATE SYNTHASE INO1 (Inositol 1-phosphate synthetase) (D-glucose 6-phosphate cycloaldolase) (Glucose 6-phosphate cyclase) (Glucocycloaldolase) | 104, 163, 217, 230, 314, 333 |
| Rv0047c | *-* | hypothetical protein Rv0047c | 24, 55, 86, 167, 175 |
| Rv0048c | *-* | POSSIBLE MEMBRANE PROTEIN | 10, 73, 77, 92, 123, 145, 148, 192, 201, 220, 226, 269 |
| Rv0049 | *-* | hypothetical protein Rv0049 | 18, 33, 70, 73, 111, 122 |
| Rv0050 | *ponA1* | PROBABLE BIFUNCTIONAL PENICILLIN-BINDING PROTEIN 1A/1B PONA1 (MUREIN POLYMERASE) (PBP1): PENICILLIN-INSENSITIVE TRANSGLYCOSYLASE (PEPTIDOGLYCAN TGASE) + PENICILLIN-SENSITIVE TRANSPEPTIDASE (DD-TRANSPEPTIDASE) | 83, 95, 100, 116, 122, 309, 353, 385, 411, 419, 423, 469, 471, 496, 500, 539, 548, 579, 621, 669 |
| Rv0051 | *-* | PROBABLE CONSERVED TRANSMEMBRANE PROTEIN | 1, 16, 36, 86, 88, 175, 196, 215, 226, 262, 270, 290, 302, 320, 332, 347, 386, 479, 489, 496, 497, 519, 554 |
| Rv0052 | *-* | hypothetical protein Rv0052 | 12, 43, 75, 76, 83, 90, 123, 125, 129, 146, 164 |
| Rv0053 | *rpsF* | 30S ribosomal protein S6 | 33, 66 |
| Rv0054 | *ssb* | single-strand DNA-binding protein | 13, 23, 43, 79, 113, 122, 127, 155 |
| Rv0055 | *rpsR* | 30S ribosomal protein S18 | 34, 44 |
| Rv0056 | *rplI* | 50S ribosomal protein L9 | 32, 40, 95, 106 |
| Rv0057 | *-* | hypothetical protein Rv0057 | 4, 19, 22, 32, 35, 55, 138 |
| Rv0058 | *dnaB* | replicative DNA helicase | 8, 28, 35, 53, 84, 97, 112, 134, 144, 195, 199, 203, 401, 411, 423, 449, 461, 468, 540, 575, 670, 734, 800 |
| Rv0059 | *-* | hypothetical protein Rv0059 | 14, 41, 105, 108, 109, 111, 180, 225 |
| Rv0060 | *-* | hypothetical protein Rv0060 | 43, 51, 64, 93, 218, 247, 281, 322 |
| Rv0061 | *-* | hypothetical protein Rv0061 | 19, 32, 43, 130, 139 |
| Rv0062 | *celA1* | POSSIBLE CELLULASE CELA1 (ENDOGLUCANASE) (ENDO-1,4-BETA-GLUCANASE) (FI-CMCASE) (CARBOXYMETHYL CELLULASE) | 9, 13, 23, 91, 99, 106, 139, 146, 152, 168, 173, 183, 191, 237, 256, 268, 308, 310, 334, 348, 355, 357, 377 |
| Rv0063 | *-* | POSSIBLE OXIDOREDUCTASE | 13, 14, 19, 29, 42, 93, 102, 104, 135, 142, 144, 154, 156, 162, 176, 186, 202, 207, 209, 221, 223, 225, 241, 269, 297, 300, 305, 316, 346, 358, 413, 425, 436, 474 |
| Rv0064 | *-* | hypothetical protein Rv0064 | 73, 126, 193, 199, 216, 245, 247, 275, 288, 316, 423, 444, 465, 509, 514, 529, 531, 627, 674, 769, 837, 876, 896, 898, 903, 905, 909, 917, 918, 946, 972, 976 |
| Rv0065 | *-* | hypothetical protein Rv0065 | 15, 25, 63, 105, 120 |
| Rv0066c | *icd2* | PROBABLE ISOCITRATE DEHYDROGENASE | 96, 184, 205, 247, 355, 361, 443, 454, 482, 542, 544, 575, 576, 585, 614, 623, 637, 653, 674, 715 |
| Rv0067c | *-* | POSSIBLE TRANSCRIPTIONAL REGULATORY PROTEIN (POSSIBLY TETR-FAMILY) | 42, 84, 104, 111, 138, 142, 172 |
| Rv0068 | *-* | short chain dehydrogenase | 5, 12, 15, 23, 29, 62, 81, 100, 129, 140, 156, 173, 196, 245, 249, 252, 295 |
| Rv0069c | *sdaA* | PROBABLE L-SERINE DEHYDRATASE SDAA (L-SERINE DEAMINASE) (SDH) (L-SD) | 56, 58, 93, 99, 130, 147, 157, 160, 207, 232, 276, 282, 288, 289, 305, 335, 347, 352, 354, 361, 370, 387, 401, 402, 408, 414, 433, 446, 447 |
| Rv0070c | *glyA2* | PROBABLE SERINE HYDROXYMETHYLTRANSFERASE GLYA2 (SERINE METHYLASE 2) (SHMT 2) | 8, 15, 27, 99, 102, 111, 123, 135, 142, 172, 205, 209, 232, 235, 252, 260, 272, 297, 314, 315, 341, 373, 375, 412 |
| Rv0071 | *-* | POSSIBLE MATURASE | 27, 74, 117, 195, 211 |
| Rv0072 | *-* | PROBABLE GLUTAMINE-TRANSPORT TRANSMEMBRANE PROTEIN ABC TRANSPORTER | 22, 33, 59, 80, 83, 87, 165, 275, 320, 331, 341 |
| Rv0073 | *-* | PROBABLE GLUTAMINE-TRANSPORT ATP-BINDING PROTEIN ABC TRANSPORTER | 14, 31, 41, 43, 53, 100, 112, 140, 144, 170, 232, 263, 271 |
| Rv0074 | *-* | hypothetical protein Rv0074 | 50, 88, 103, 105, 109, 151, 159, 176, 189, 205, 209, 214, 223, 229, 233, 237, 254, 271, 303, 343, 346, 349, 381, 382, 393, 402 |
| Rv0075 | *-* | PROBABLE AMINOTRANSFERASE | 40, 129, 155, 159, 161, 213, 277, 309, 355, 382 |
| Rv0076c | *-* | PROBABLE MEMBRANE PROTEIN | 1, 60, 125 |
| Rv0077c | *-* | PROBABLE OXIDOREDUCTASE | 7, 15, 32, 44, 66, 73, 76, 84, 98, 99, 139, 148, 157, 164, 205, 208, 238 |
| Rv0078 | *-* | PROBABLE TRANSCRIPTIONAL REGULATORY PROTEIN | 11, 20, 81, 147, 175 |
| Rv0078A | *-* | hypothetical protein Rv0078A | 15, 52, 87, 91, 158, 177, 193 |
| Rv0079 | *-* | hypothetical protein Rv0079 | 27, 28, 53, 87, 89, 97, 152, 203, 222, 243, 245, 263 |
| Rv0080 | *-* | hypothetical protein Rv0080 | 77, 140 |
| Rv0081 | *-* | PROBABLE TRANSCRIPTIONAL REGULATORY PROTEIN | 77, 108, 109 |
| Rv0082 | *-* | PROBABLE OXIDOREDUCTASE | 15, 27, 28, 81, 89, 108, 139, 145, 156 |
| Rv0083 | *-* | PROBABLE OXIDOREDUCTASE | 1, 6, 7, 17, 42, 52, 99, 158, 165, 176, 249, 252, 263, 294, 303, 305, 325, 330, 332, 341, 357, 368, 385, 406, 442, 468, 481, 490, 493, 498, 502, 507, 534, 573, 587, 606, 617 |
| Rv0084 | *hycD* | POSSIBLE FORMATE HYDROGENLYASE HYCD (FHL) | 6, 10, 34, 36, 64, 71, 78, 86, 113, 117, 122, 133, 150, 151, 174, 190, 216, 227, 248, 250, 253, 259, 295, 306 |
| Rv0085 | *hycP* | POSSIBLE HYDROGENASE HYCP | 12, 44, 50, 87, 98, 105, 106, 111, 133, 155, 156, 168, 173, 174, 199 |
| Rv0086 | *hycQ* | POSSIBLE HYDROGENASE HYCQ | 1, 6, 13, 21, 31, 34, 58, 82, 140, 151, 180, 184, 186, 189, 201, 202, 206, 212, 213, 241, 245, 249, 271, 273, 298, 319, 321, 322, 329, 349, 354, 359, 365, 431, 451, 469, 480 |
| Rv0087 | *hycE* | POSSIBLE FORMATE HYDROGENASE HYCE (FHL) | 119, 121, 139, 151, 156, 190, 193, 202, 246, 277, 280, 286, 292, 330, 332, 353, 392, 395, 406, 412, 423, 432, 461 |
| Rv0088 | *-* | hypothetical protein Rv0088 | 34, 74, 84, 110, 143, 183, 184, 220 |
| Rv0089 | *-* | POSSIBLE METHYLTRANSFERASE/METHYLASE | 30, 92, 108, 113, 140, 153, 176 |
| Rv0090 | *-* | POSSIBLE MEMBRANE PROTEIN | 39, 50, 72, 99, 107, 127, 145, 215, 230, 241, 251 |
| Rv0091 | *mtn* | PROBABLE BIFUNCTIONAL MTA/SAH NUCLEOSIDASE MTN: 5'-METHYLTHIOADENOSINE NUCLEOSIDASE (METHYLTHIOADENOSINE METHYLTHIORIBOHYDROLASE) + S-ADENOSYLHOMOCYSTEINE NUCLEOSIDASE (S-ADENOSYL-L-HOMOCYSTEINE HOMOCYSTEINYLRIBOHYDROLASE) | 18, 36, 47, 49, 59, 78, 79, 114, 132, 152, 170, 189, 197, 221 |
| Rv0092 | *ctpA* | PROBABLE CATION TRANSPORTER P-TYPE ATPASE A CTPA | 5, 20, 63, 104, 127, 160, 173, 192, 277, 286, 295, 311, 325, 334, 363, 372, 374, 379, 389, 408, 416, 444, 458, 472, 477, 488, 504, 506, 509, 513, 558, 576, 589, 627, 642, 655, 663, 703, 708, 710, 718, 746, 755, 759 |
| Rv0093c | *-* | PROBABLE CONSERVED MEMBRANE PROTEIN | 7, 25, 32, 161, 189, 191, 194, 205, 214, 253, 266, 270, 279 |
| Rv0094c | *-* | hypothetical protein Rv0094c | 5, 11, 14, 48, 83, 85, 104, 123, 131, 155, 157, 162, 163, 185, 187, 223, 228, 243, 248, 260 |
| Rv0095c | *-* | hypothetical protein Rv0095c | 24, 108, 126 |
| Rv0096 | *PPE1* | PPE FAMILY PROTEIN | 13, 15, 23, 56, 75, 82, 90, 98, 107, 148, 173, 174, 177, 258, 278, 283, 303, 304, 312, 314, 329, 336, 341, 349, 356, 363, 382, 387, 397, 402, 407, 409, 418, 424, 426, 431, 433, 435 |
| Rv0097 | *-* | POSSIBLE OXIDOREDUCTASE | 91, 121, 136, 144, 192, 227, 266 |
| Rv0098 | *-* | hypothetical protein Rv0098 | 15, 63, 167, 171 |
| Rv0099 | *fadD10* | acyl-CoA synthetase | 54, 113, 127, 144, 160, 177, 238, 243, 244, 279, 308, 312, 321, 355, 363, 365, 367, 370, 394, 405, 452, 516 |
| Rv0100 | *-* | hypothetical protein Rv0100 | 6, 57 |
| Rv0101 | *nrp* | PROBABLE PEPTIDE SYNTHETASE NRP (PEPTIDE SYNTHASE) | 22, 43, 115, 128, 131, 193, 203, 205, 218, 303, 391, 447, 453, 477, 479, 507, 514, 540, 573, 583, 643, 648, 670, 697, 792, 818, 828, 845, 856, 876, 927, 935, 990, 1015, 1019, 1040, 1053, 1106, 1155, 1212, 1223, 1233, 1244, 1277, 1287, 1299, 1324, 1342, 1400, 1420, 1423, 1453, 1486, 1498, 1512, 1538, 1564, 1591, 1628, 1642, 1746, 1760, 1813, 1816, 1822, 1837, 1847, 1849, 1856, 1881, 1892, 1926, 1932, 1943, 1949, 1985, 1993, 2015, 2026, 2095, 2158, 2171, 2207, 2227, 2247, 2276, 2280, 2288, 2309, 2319, 2338, 2379, 2411, 2466, 2474 |
| Rv0102 | *-* | PROBABLE CONSERVED INTEGRAL MEMBRANE PROTEIN | 52, 71, 75, 100, 120, 128, 136, 150, 156, 195, 198, 213, 225, 307, 311, 313, 319, 331, 372, 386, 408, 414, 416, 489, 598 |
| Rv0103c | *ctpB* | PROBABLE CATION-TRANSPORTER P-TYPE ATPASE B CTPB | 40, 66, 72, 130, 140, 147, 176, 178, 213, 221, 233, 298, 328, 337, 382, 411, 417, 447, 479, 496, 513, 520, 545, 582, 590, 598, 603, 616, 651, 717, 719, 727, 729 |
| Rv0104 | *-* | hypothetical protein Rv0104 | 27, 110, 122, 129, 146, 147, 179, 190, 202, 229, 242, 251, 257, 266, 325, 339, 399, 418, 435, 495 |
| Rv0105c | *rpmB* | 50S ribosomal protein L28 | 1, 7, 74 |
| Rv0106 | *-* | hypothetical protein Rv0106 | 24, 121, 192, 193, 252, 283, 285, 292, 328, 335 |
| Rv0107c | *ctpI* | PROBABLE CATION-TRANSPORTER ATPASE I CTPI | 22, 31, 45, 63, 81, 102, 149, 183, 197, 204, 214, 241, 283, 302, 327, 332, 336, 342, 359, 366, 367, 374, 379, 384, 398, 403, 410, 430, 444, 475, 526, 530, 570, 577, 590, 605, 616, 621, 639, 651, 657, 678, 680, 686, 691, 706, 721, 722, 731, 753, 782, 807, 819, 871, 896, 928, 938, 942, 958, 977, 978, 980, 1006, 1022, 1054, 1072, 1087, 1093, 1123, 1146, 1149, 1266, 1272, 1290, 1306, 1330, 1344, 1357, 1359, 1363, 1392, 1403, 1420, 1441, 1495, 1499, 1501, 1514, 1549, 1554 |
| Rv0108c | *-* | hypothetical protein Rv0108c | 8, 18 |
| Rv0109 | *PE_PGRS1* | PE-PGRS FAMILY PROTEIN | 32, 88, 134, 140, 141, 151, 153, 154, 159, 163, 166, 172, 174, 175, 178, 187, 190, 192, 200, 202, 203, 205, 211, 217, 219, 220, 229, 232, 233, 236, 239, 242, 247, 253, 255, 256, 262, 264, 265, 267, 275, 280, 293, 295, 296, 301, 304, 307, 310, 313, 319, 321, 324, 325, 335, 338, 341, 347, 349, 350, 357, 367, 377, 379, 380, 383, 389, 392, 395, 405, 407, 408, 410, 411, 413, 417, 424, 427, 429, 436, 438, 440, 451, 464, 473, 475, 476, 479, 481, 489, 492 |
| Rv0110 | *-* | PROBABLE CONSERVED INTEGRAL MEMBRANE PROTEIN | 28, 68, 72, 191 |
| Rv0111 | *-* | POSSIBLE TRANSMEMBRANE ACYLTRANSFERASE | 30, 51, 54, 57, 70, 85, 91, 114, 125, 133, 201, 206, 218, 220, 249, 264, 327, 374, 384, 417, 428, 439, 457, 459, 489, 505, 585, 598 |
| Rv0112 | *gca* | POSSIBLE GDP-MANNOSE 4,6-DEHYDRATASE GCA (GDP-D-MANNOSE DEHYDRATASE) | 8, 21, 22, 94, 172, 230, 266, 313 |
| Rv0113 | *gmhA* | PROBABLE SEDOHEPTULOSE-7-PHOSPHATE ISOMERASE GMHA (PHOSPHOHEPTOSE ISOMERASE) | 2, 29, 39, 63, 112, 122, 131, 134, 149, 150, 168 |
| Rv0114 | *gmhB* | POSSIBLE D-ALPHA,BETA-D-HEPTOSE-1,7-BIPHOSPHATE PHOSPHATASE GMHB (D-GLYCERO-D-MANNO-HEPTOSE 7-PHOSPHATE KINASE) | 38, 65, 147, 150, 164 |
| Rv0115 | *hddA* | POSSIBLE D-ALPHA-D-HEPTOSE-7-PHOSPHATE KINASE HDDA | 4, 14, 16, 28, 46, 62, 109, 155, 163, 167, 204, 252, 260, 275, 287, 291, 297, 323, 335 |
| Rv0116c | *-* | POSSIBLE CONSERVED MEMBRANE PROTEIN | 18, 26, 82, 120, 148, 192, 202 |
| Rv0117 | *oxyS* | OXIDATIVE STRESS RESPONSE REGULATORY PROTEIN OXYS | 20, 49, 86, 89, 99, 186, 206, 233 |
| Rv0118c | *oxcA* | putative oxalyl-CoA decarboxylase | 44, 66, 72, 75, 82, 84, 91, 107, 119, 130, 149, 157, 160, 163, 164, 171, 185, 242, 324, 350, 422, 438, 442, 458, 480, 484, 494, 521, 561, 575, 578 |
| Rv0119 | *fadD7* | acyl-CoA synthetase | 28, 47, 53, 57, 103, 134, 137, 150, 156, 174, 175, 178, 200, 232, 233, 239, 241, 245, 285, 301, 344, 366, 382, 391, 402, 411, 417, 472, 501, 506 |
| Rv0120c | *fusA2* | elongation factor G | 11, 15, 19, 20, 33, 106, 110, 161, 178, 215, 237, 269, 282, 337, 357, 406, 452, 479, 508, 521, 541, 551, 576, 605, 613, 615, 656, 687, 703, 705, 710, 712 |
| Rv0121c | *-* | hypothetical protein Rv0121c | 44, 51, 98, 109 |
| Rv0122 | *-* | hypothetical protein Rv0122 | 1, 6, 69, 74 |
| Rv0123 | *-* | hypothetical protein Rv0123 | 7, 61, 66, 67 |
| Rv0124 | *PE_PGRS2* | PE-PGRS FAMILY PROTEIN | 12, 29, 36, 40, 55, 80, 88, 99, 127, 128, 138, 141, 143, 149, 152, 153, 156, 162, 164, 165, 167, 168, 170, 173, 174, 176, 177, 180, 187, 190, 192, 193, 203, 206, 209, 211, 212, 217, 218, 220, 224, 234, 241, 243, 245, 247, 254, 256, 257, 260, 268, 271, 272, 278, 280, 282, 288, 290, 291, 294, 297, 300, 306, 313, 317, 324, 326, 327, 330, 335, 336, 339, 342, 344, 345, 356, 357, 359, 360, 370, 371, 374, 376, 384, 386, 387, 390, 395, 398, 403, 404, 418, 419, 421, 422, 426, 434, 436, 443, 450, 455, 457, 458, 466, 476, 479, 482, 485 |
| Rv0125 | *pepA* | PROBABLE SERINE PROTEASE PEPA (SERINE PROTEINASE) (MTB32A) | 45, 52, 77, 79, 109, 132, 134, 144, 158, 162, 186, 200, 204, 208, 224, 266, 299, 304, 315, 326, 339 |
| Rv0126 | *treS* | TREHALOSE SYNTHASE TRES | 19, 33, 37, 64, 98, 124, 213, 272, 296, 399, 406, 447, 495, 506, 520, 556, 594 |
| Rv0127 | *-* | hypothetical protein Rv0127 | 23, 79, 97, 109, 116, 132, 163, 178, 225, 243, 247, 270, 361, 370, 385, 397, 430, 452 |
| Rv0128 | *-* | PROBABLE CONSERVED TRANSMEMBRANE PROTEIN | 19, 25, 30, 39, 59, 69, 100, 101, 108, 148, 163, 164, 184, 186, 195, 211, 251 |
| Rv0129c | *fbpC* | SECRETED ANTIGEN 85-C FBPC (85C) (ANTIGEN 85 COMPLEX C) (AG58C) (MYCOLYL TRANSFERASE 85C) (FIBRONECTIN-BINDING PROTEIN C) | 42, 49, 60, 73, 116, 149, 172, 174, 189, 215, 254, 263, 332, 338 |
| Rv0130 | *-* | hypothetical protein Rv0130 | 10, 12 |
| Rv0131c | *fadE1* | PROBABLE ACYL-CoA DEHYDROGENASE FADE1 | 6, 73, 81, 114, 156, 161, 167, 254, 268, 330 |
| Rv0132c | *fgd2* | PUTATIVE F420-DEPENDENT GLUCOSE-6-PHOSPHATE DEHYDROGENASE FGD2 | 1, 11, 12, 19, 23, 30, 41, 63, 109, 135, 144, 152, 179, 210, 212, 220, 241, 244, 250, 273, 276, 283, 284, 298, 317, 330, 345 |
| Rv0133 | *-* | PROBABLE ACETYLTRANSFERASE | 40, 57, 58, 65, 71, 127, 136 |
| Rv0134 | *ephF* | POSSIBLE EPOXIDE HYDROLASE EPHF (EPOXIDE HYDRATASE) (ARENE-OXIDE HYDRATASE) | 4, 27, 56, 68, 125, 178, 183, 207, 225, 243 |
| Rv0135c | *-* | POSSIBLE TRANSCRIPTIONAL REGULATORY PROTEIN | 4, 5, 121, 129, 165, 171 |
| Rv0136 | *cyp138* | PROBABLE CYTOCHROME P450 138 CYP138 | 5, 20, 50, 92, 134, 164, 165, 264, 265, 298, 338, 382, 415, 432 |
| Rv0137c | *msrA* | methionine sulfoxide reductase A | 9, 10, 24, 34, 116, 120, 130, 158, 166 |
| Rv0138 | *-* | hypothetical protein Rv0138 | 11, 24, 87, 101, 118 |
| Rv0139 | *-* | POSSIBLE OXIDOREDUCTASE | 38, 60, 68, 165, 176, 202, 224, 247, 251, 322, 336 |
| Rv0140 | *-* | hypothetical protein Rv0140 | 36, 86 |
| Rv0141c | *-* | hypothetical protein Rv0141c | 21 |
| Rv0142 | *-* | hypothetical protein Rv0142 | 17, 28, 38, 51, 55, 65, 73, 83, 88, 103, 117, 134, 154, 159, 208, 226, 264, 287, 295 |
| Rv0143c | *-* | PROBABLE CONSERVED TRANSMEMBRANE PROTEIN | 29, 30, 57, 91, 94, 97, 111, 128, 165, 168, 174, 179, 198, 206, 217, 245, 286, 307, 317, 334, 342, 373, 374, 377, 404, 440, 477, 482 |
| Rv0144 | *-* | PROBABLE TRANSCRIPTIONAL REGULATORY PROTEIN (POSSIBLY TETR-FAMILY) | 32, 41, 73, 75, 95, 149, 179, 182, 223, 227, 271 |
| Rv0145 | *-* | hypothetical protein Rv0145 | 15, 29, 42, 62, 105, 119, 176, 181, 201, 204, 213, 264, 312 |
| Rv0146 | *-* | hypothetical protein Rv0146 | 16, 47, 49, 72, 99, 100, 110, 122, 123, 158, 166, 172, 305 |
| Rv0147 | *-* | PROBABLE ALDEHYDE DEHYDROGENASE (NAD+) DEPENDENT | 60, 88, 111, 138, 140, 173, 186, 230, 243, 283, 308, 316, 338, 348, 354, 432 |
| Rv0148 | *-* | PROBABLE SHORT-CHAIN TYPE DEHYDROGENASE/REDUCTASE | 1, 13, 14, 26, 46, 84, 99, 125, 152, 276 |
| Rv0149 | *-* | POSSIBLE QUINONE OXIDOREDUCTASE (NADPH:QUINONE OXIDOREDUCTASE) (ZETA-CRYSTALLIN) | 9, 12, 23, 26, 35, 36, 60, 90, 113, 133, 146, 147, 148, 153, 215, 238, 270, 273, 303, 311 |
| Rv0150c | *-* | hypothetical protein Rv0150c | 10, 78 |
| Rv0151c | *PE1* | PE FAMILY PROTEIN | 13, 56, 74, 80, 120, 141, 165, 167, 207, 235, 284, 291, 449, 464, 475, 509, 529, 542, 560, 563 |
| Rv0152c | *PE2* | PE FAMILY PROTEIN | 36, 47, 60, 62, 66, 87, 123, 152, 179, 181, 201, 273, 368, 369, 386, 400, 405, 408, 481, 491 |
| Rv0153c | *ptbB* | PHOSPHOTYROSINE PROTEIN PHOSPHATASE PTPB (PROTEIN-TYROSINE-PHOSPHATASE) (PTPase) | 17, 19, 38, 63, 95, 110, 150, 151, 166, 239, 253, 259, 270 |
| Rv0154c | *fadE2* | PROBABLE ACYL-CoA DEHYDROGENASE FADE2 | 33, 39, 54, 79, 93, 97, 185, 200, 240, 352, 353 |
| Rv0155 | *pntAa* | PROBABLE NAD(P) TRANSHYDROGENASE (SUBUNIT ALPHA) PNTAA | 44, 58, 69, 88, 112, 164, 180, 223, 226, 257, 270, 287, 291, 299, 311, 333, 361 |
| Rv0156 | *pntAb* | PROBABLE NAD(P) TRANSHYDROGENASE (SUBUNIT ALPHA) PNTAB | 14, 79, 96 |
| Rv0157 | *pntB* | PROBABLE NAD(P) TRANSHYDROGENASE (SUBUNIT BETA) PNTB | 25, 29, 32, 37, 39, 63, 82, 95, 111, 114, 133, 156, 179, 190, 192, 206, 207, 234, 238, 248, 253, 260, 282, 285, 287, 293, 305, 308, 322, 357, 361, 438 |
| Rv0158 | *-* | PROBABLE TRANSCRIPTIONAL REGULATORY PROTEIN (POSSIBLY TETR-FAMILY) | 26, 71, 103, 142, 146, 175, 193 |
| Rv0159c | *PE3* | PE FAMILY PROTEIN | 14, 30, 36, 40, 42, 49, 81, 82, 84, 94, 100, 101, 104, 119, 124, 126, 205, 242, 276, 312, 329, 402, 410, 421 |
| Rv0160c | *PE4* | PE FAMILY PROTEIN | 26, 40, 42, 68, 81, 82, 123, 125, 126, 146, 209, 264, 271, 352, 377, 403, 413, 447, 486 |
| Rv0161 | *-* | POSSIBLE OXIDOREDUCTASE | 21, 28, 41, 65, 105, 110, 115, 132, 143, 144, 182, 187, 201, 219, 230, 250, 275, 289, 294, 297, 303, 339, 398, 426, 438, 443 |
| Rv0162c | *adhE1* | PROBABLE ZINC-TYPE ALCOHOL DEHYDROGENASE (E SUBUNIT) ADHE | 46, 82, 133, 139, 146, 191, 204, 223, 244, 261, 262, 266, 277, 281, 286, 288, 379 |
| Rv0163 | *-* | hypothetical protein Rv0163 | 44, 54, 115, 149 |
| Rv0164 | *TB18.5* | hypothetical protein Rv0164 | 25, 28, 69, 112, 114 |
| Rv0165c | *-* | POSSIBLE TRANSCRIPTIONAL REGULATORY PROTEIN (PROBABLY GNTR-FAMILY) | 27, 30, 70, 107, 118, 149, 168, 173, 206, 215, 218, 222, 224 |
| Rv0166 | *fadD5* | acyl-CoA synthetase | 1, 68, 70, 94, 152, 153, 170, 194, 208, 213, 237, 244, 275, 281, 329, 352, 363, 400, 406, 435, 436, 515 |
| Rv0167 | *yrbE1A* | CONSERVED HYPOTHETICAL INTEGRAL MEMBRANE PROTEIN YRBE1A | 7, 88, 90, 110, 112, 183, 194, 206, 226, 233 |
| Rv0168 | *yrbE1B* | CONSERVED HYPOTHETICAL INTEGRAL MEMBRANE PROTEIN YRBE1B | 2, 31, 67, 74, 86, 108, 112, 120, 171, 243, 244 |
| Rv0169 | *mce1A* | MCE-FAMILY PROTEIN MCE1A | 37, 50, 59, 116, 174, 179, 237, 256, 268, 283, 312, 314, 328, 353, 363, 371, 375, 393, 400, 420 |
| Rv0170 | *mce1B* | MCE-FAMILY PROTEIN MCE1B | 39, 46, 93, 154, 182, 239, 240, 249, 293, 325 |
| Rv0171 | *mce1C* | MCE-FAMILY PROTEIN MCE1C | 48, 49, 60, 112, 119, 144, 146, 173, 225, 230, 242, 338, 372, 431, 449, 461, 489, 500, 501, 503, 504 |
| Rv0172 | *mce1D* | MCE-FAMILY PROTEIN MCE1D | 31, 32, 80, 101, 181, 205, 327, 383, 410, 426, 432, 472, 522 |
| Rv0173 | *lprK* | POSSIBLE MCE-FAMILY LIPOPROTEIN LPRK (MCE-FAMILY LIPOPROTEIN MCE1E) | 30, 33, 41, 42, 46, 106, 123, 162, 164, 223, 263, 292, 341, 343, 346, 378 |
| Rv0174 | *mce1F* | MCE-FAMILY PROTEIN MCE1F | 46, 60, 113, 122, 144, 147, 161, 170, 200, 225, 228, 245, 254, 279, 292, 416, 427, 433, 482 |
| Rv0175 | *-* | PROBABLE CONSERVED MCE ASSOCIATED MEMBRANE PROTEIN | 10, 29, 66, 96, 124 |
| Rv0176 | *-* | PROBABLE CONSERVED MCE ASSOCIATED TRANSMEMBRANE PROTEIN | 35, 82, 91, 101, 156, 178, 279, 300, 318 |
| Rv0177 | *-* | PROBABLE CONSERVED MCE ASSOCIATED PROTEIN | 8, 32, 34, 39, 115, 150 |
| Rv0178 | *-* | PROBABLE CONSERVED MCE ASSOCIATED MEMBRANE PROTEIN | 7, 25, 37, 44, 46, 59, 65, 85, 119, 142, 147, 162, 177, 185 |
| Rv0179c | *lprO* | POSSIBLE LIPOPROTEIN LPRO | 24, 30, 36, 40, 43, 67, 87, 124, 159, 169, 183, 193, 199, 205, 257, 264, 273, 277, 296, 315, 321, 333, 338 |
| Rv0180c | *-* | PROBABLE CONSERVED TRANSMEMBRANE PROTEIN | 7, 40, 45, 66, 68, 79, 102, 135, 153, 173, 180, 181, 186, 191, 202, 211, 230, 386 |
| Rv0181c | *-* | hypothetical protein Rv0181c | 1, 80, 81, 84, 89, 97, 99, 126, 144, 154, 176, 202, 219, 222 |
| Rv0182c | *sigG* | RNA polymerase factor sigma-70 | 6, 18, 49, 54, 58, 142, 238, 243, 259, 274, 285, 303, 321, 329, 344 |
| Rv0183 | *-* | POSSIBLE LYSOPHOSPHOLIPASE | 26, 55, 65, 84, 99, 111, 149, 158, 193, 214, 251 |
| Rv0184 | *-* | hypothetical protein Rv0184 | 31, 55, 72, 144, 182, 204, 213, 223, 240, 244 |
| Rv0185 | *-* | hypothetical protein Rv0185 | 29, 89, 97, 109, 110 |
| Rv0186 | *bglS* | PROBABLE BETA-GLUCOSIDASE BGLS (GENTIOBIASE) (CELLOBIASE) (BETA-D-GLUCOSIDE GLUCOHYDROLASE) | 13, 34, 53, 64, 67, 72, 73, 100, 107, 136, 193, 209, 226, 234, 244, 250, 270, 316, 340, 347, 356, 382, 456, 460, 505, 585, 602, 615, 632, 662, 684 |
| Rv0187 | *-* | PROBABLE O-METHYLTRANSFERASE | 27, 55, 128, 157, 169, 195 |
| Rv0188 | *-* | PROBABLE CONSERVED TRANSMEMBRANE PROTEIN | 23, 137 |
| Rv0189c | *ilvD* | dihydroxy-acid dehydratase | 7, 30, 81, 82, 134, 141, 146, 164, 191, 208, 213, 218, 225, 253, 263, 266, 287, 340, 352, 363, 394, 413, 414, 424, 458, 460, 490, 494, 508, 567 |
| Rv0190 | *-* | hypothetical protein Rv0190 | 25, 74 |
| Rv0191 | *-* | PROBABLE CONSERVED INTEGRAL MEMBRANE PROTEIN | 4, 7, 34, 79, 112, 113, 142, 181, 185, 190, 232, 261, 266, 291, 301, 310, 318, 342, 364, 382, 409 |
| Rv0192 | *-* | hypothetical protein Rv0192 | 120, 199, 202, 209, 217, 224, 252, 253, 301, 341 |
| Rv0192A | *-* | CONSERVED SECRETED PROTEIN | 14, 40, 43, 46 |
| Rv0193c | *-* | hypothetical protein Rv0193c | 32, 56, 97, 154, 189, 224, 226, 239, 282, 300, 304, 315, 317, 373, 530, 545, 582 |
| Rv0194 | *-* | PROBABLE DRUGS-TRANSPORT TRANSMEMBRANE ATP-BINDING PROTEIN ABC TRANSPORTER | 9, 28, 64, 66, 80, 112, 180, 191, 194, 202, 249, 260, 264, 303, 358, 370, 420, 466, 471, 472, 506, 520, 542, 567, 588, 594, 603, 610, 623, 669, 671, 688, 691, 725, 728, 803, 842, 914, 959, 967, 977, 979, 1029, 1062, 1073, 1080, 1081 |
| Rv0195 | *-* | POSSIBLE TWO COMPONENT TRANSCRIPTIONAL REGULATORY PROTEIN (PROBABLY LUXR-FAMILY) | 53, 82, 95, 115, 139, 147 |
| Rv0196 | *-* | POSSIBLE TRANSCRIPTIONAL REGULATORY PROTEIN | 11, 23, 32, 45, 58, 63, 94, 99 |
| Rv0197 | *-* | POSSIBLE OXIDOREDUCTASE | 8, 17, 97, 105, 107, 114, 118, 139, 150, 154, 190, 204, 242, 253, 267, 276, 283, 315, 338, 344, 388, 452, 482, 508, 525, 528, 548, 565, 668, 685, 688, 699, 708, 725, 734 |
| Rv0198c | *-* | PROBABLE ZINC METALLOPROTEASE | 117, 126, 129, 153, 169, 197, 247, 315, 393, 421, 464, 480, 484, 524, 546, 565, 582, 590 |
| Rv0199 | *omamA* | PROBABLE CONSERVED MEMBRANE PROTEIN | 22, 25, 34, 54, 64, 70, 82, 136, 155, 157, 187 |
| Rv0200 | *-* | POSSIBLE CONSERVED TRANSMEMBRANE PROTEIN | 19, 22, 70, 81, 129, 135, 145, 161, 167, 172, 195 |
| Rv0201c | *-* | hypothetical protein Rv0201c | 8, 52, 76, 131, 163 |
| Rv0202c | *mmpL11* | PROBABLE CONSERVED TRANSMEMBRANE TRANSPORT PROTEIN MMPL11 | 37, 39, 44, 147, 166, 168, 200, 262, 272, 284, 293, 308, 407, 411, 432, 433, 436, 445, 502, 510, 513, 524, 564, 645, 654, 659, 734, 770, 825, 835, 852, 860, 895, 897, 912, 938 |
| Rv0203 | *-* | POSSIBLE EXPORTED PROTEIN | 4, 23, 34, 71, 119 |
| Rv0204c | *-* | PROBABLE CONSERVED TRANSMEMBRANE PROTEIN | 5, 25, 32, 78, 82, 100, 101, 116, 189, 223, 272, 275, 276, 290, 302, 314, 319, 342, 358, 405 |
| Rv0205 | *-* | PROBABLE CONSERVED TRANSMEMBRANE PROTEIN | 16, 28, 81, 87, 114, 127, 137, 165, 206, 227, 262, 318, 320, 324, 348 |
| Rv0206c | *mmpL3* | POSSIBLE CONSERVED TRANSMEMBRANE TRANSPORT PROTEIN MMPL3 | 25, 40, 65, 76, 108, 117, 121, 163, 173, 200, 204, 205, 217, 279, 280, 360, 381, 399, 419, 444, 479, 544, 654, 666, 669, 674, 763, 777, 816, 845, 868, 885, 892, 915, 926, 928, 932 |
| Rv0207c | *-* | hypothetical protein Rv0207c | 79, 84, 152, 174 |
| Rv0208c | *trmB* | tRNA (guanine-N(7))-methyltransferase | 10, 62, 84, 86, 110, 150, 191, 196, 203, 205, 241 |
| Rv0209 | *-* | hypothetical protein Rv0209 | 1, 19, 31, 43, 71, 96, 166, 233, 243, 245, 265, 272, 291, 310, 333, 348, 349 |
| Rv0210 | *-* | hypothetical protein Rv0210 | 9, 23, 39, 44, 47, 70, 82, 85, 97, 116, 130, 145, 160, 198, 260, 275, 339, 358, 376, 424, 458, 471 |
| Rv0211 | *pckA* | phosphoenolpyruvate carboxykinase | 6, 10, 54, 95, 104, 120, 164, 220, 266, 285, 336, 337, 417, 448, 503, 532, 533, 543, 557, 561, 591 |
| Rv0212c | *nadR* | POSSIBLE TRANSCRIPTIONAL REGULATORY PROTEIN NADR (PROBABLY ASNC-FAMILY) | 162, 192, 205, 240, 275, 278, 291, 301 |
| Rv0213c | *-* | POSSIBLE METHYLTRANSFERASE (METHYLASE) | 21, 36, 164, 171, 180, 182, 186, 230, 236, 304, 375, 428 |
| Rv0214 | *fadD4* | acyl-CoA synthetase | 14, 19, 25, 43, 50, 101, 113, 124, 148, 157, 159, 214, 234, 240, 251, 312, 341, 364, 388, 401, 428, 437, 438, 476, 483, 491, 522, 528 |
| Rv0215c | *fadE3* | PROBABLE ACYL-CoA DEHYDROGENASE FADE3 | 59, 77, 83, 87, 126, 127, 148, 165, 192, 229, 251, 306, 320, 343 |
| Rv0216 | *-* | hypothetical protein Rv0216 | 2, 10, 37, 94, 129, 156, 163, 182, 186, 199, 205, 208, 215, 242, 249, 279 |
| Rv0217c | *lipW* | POSSIBLE ESTERASE LIPW | 1, 54, 63, 64, 71, 78, 79, 85, 119, 139, 145, 149, 150, 165, 187, 254, 293 |
| Rv0218 | *-* | PROBABLE CONSERVED TRANSMEMBRANE PROTEIN | 16, 17, 64, 81, 90, 162, 179, 205, 246, 254, 269, 295, 297, 299, 302, 316, 329, 335, 379, 422, 425 |
| Rv0219 | *-* | PROBABLE CONSERVED TRANSMEMBRANE PROTEIN | 34, 56, 107, 116, 130, 145, 154 |
| Rv0220 | *lipC* | PROBABLE ESTERASE LIPC | 5, 6, 9, 22, 32, 47, 65, 74, 96, 118, 154, 163, 183, 211, 224, 237, 238, 242, 245, 248, 362, 376 |
| Rv0221 | *-* | hypothetical protein Rv0221 | 169, 178, 214, 239, 267, 300, 305, 331, 354, 381, 386, 391, 404, 405, 407, 438, 451, 463 |
| Rv0222 | *echA1* | enoyl-CoA hydratase | 37, 54, 62, 65, 88, 142, 143, 165, 188, 201 |
| Rv0223c | *-* | PROBABLE ALDEHYDE DEHYDROGENASE | 13, 43, 49, 52, 97, 120, 133, 172, 180, 187, 214, 230, 243, 255, 269, 278, 283, 354, 356, 435, 447, 452, 457 |
| Rv0224c | *-* | POSSIBLE METHYLTRANSFERASE (METHYLASE) | 40, 65, 78, 94, 95, 100, 103, 150, 165, 176, 180, 204, 213, 215, 217 |
| Rv0225 | *-* | POSSIBLE CONSERVED PROTEIN | 20, 34, 44, 48, 64, 65, 79, 130, 192, 236, 307, 319, 354, 380 |
| Rv0226c | *-* | PROBABLE CONSERVED TRANSMEMBRANE PROTEIN | 16, 22, 67, 95, 100, 138, 164, 169, 174, 185, 201, 231, 271, 311, 324, 326, 343, 348, 395, 415, 425, 451, 456, 471, 479, 481, 493, 499, 521, 525, 549, 554 |
| Rv0227c | *-* | PROBABLE CONSERVED MEMBRANE PROTEIN | 4, 6, 13, 18, 79, 102, 128, 129, 219, 236, 261, 324, 326, 333, 352, 362, 414 |
| Rv0228 | *-* | PROBABLE INTEGRAL MEMBRANE ACYLTRANSFERASE | 36, 58, 62, 65, 89, 92, 104, 157, 158, 212, 230, 259, 289, 318, 336, 351, 369 |
| Rv0229c | *-* | POSSIBLE CONSERVED MEMBRANE PROTEIN | 57, 81, 106, 118, 134, 177, 187, 189, 208, 211 |
| Rv0230c | *php* | PROBABLE PHOSPHOTRIESTERASE PHP (PARATHION HYDROLASE) (PTE) (ARYLDIALKYLPHOSPHATASE) (PARAOXONASE) (A-ESTERASE) (ARYLTRIPHOSPHATASE) (PARAOXON HYDROLASE) | 5, 7, 12, 47, 59, 83, 94, 136, 150, 170, 180, 184, 221, 222, 252, 294, 322 |
| Rv0231 | *fadE4* | PROBABLE ACYL-CoA DEHYDROGENASE FADE4 | 16, 21, 100, 119, 125, 154, 157, 181, 239, 316, 341, 406, 418, 524, 545 |
| Rv0232 | *-* | PROBABLE TRANSCRIPTIONAL REGULATORY PROTEIN (PROBABLY TETR/ACRR-FAMILY) | 14, 19, 31, 41, 158, 195, 215 |
| Rv0233 | *nrdB* | ribonucleotide-diphosphate reductase subunit beta | 9, 11, 24, 65, 79, 128, 152, 188, 217, 280, 293 |
| Rv0234c | *gabD1* | succinic semialdehyde dehydrogenase | 6, 40, 63, 107, 118, 159, 197, 203, 229, 261, 266, 274, 286, 301, 305, 308, 320, 377, 385, 482, 494 |
| Rv0235c | *-* | PROBABLE CONSERVED TRANSMEMBRANE PROTEIN | 17, 30, 72, 87, 131, 143, 164, 170, 229, 234, 271, 291, 298, 370, 394 |
| Rv0236c | *-* | PROBABLE CONSERVED TRANSMEMBRANE PROTEIN | 24, 36, 83, 113, 171, 174, 191, 213, 248, 262, 276, 283, 297, 300, 310, 329, 346, 347, 408, 419, 425, 447, 452, 509, 510, 556, 580, 587, 597, 600, 606, 616, 642, 648, 681, 707, 708, 721, 732, 734, 742, 751, 777, 807, 821, 823, 830, 847, 862, 895, 939, 947, 962, 969, 972, 997, 1080, 1081, 1090, 1133, 1155, 1157, 1169, 1220, 1239, 1240, 1243, 1278, 1293, 1299, 1304, 1312, 1344, 1349, 1365 |
| Rv0236A | *-* | SMALL SECRETED PROTEIN | 6, 8, 20, 38 |
| Rv0237 | *lpqI* | PROBABLE CONSERVED LIPOPROTEIN LPQI | 22, 26, 32, 36, 88, 110, 111, 125, 136, 161, 202, 205, 215, 225, 277, 287, 294, 334, 348, 360 |
| Rv0238 | *-* | POSSIBLE TRANSCRIPTIONAL REGULATORY PROTEIN (PROBABLY TETR-FAMILY) | 19, 140, 143, 161, 201 |
| Rv0239 | *-* | hypothetical protein Rv0239 | 32 |
| Rv0240 | *-* | hypothetical protein Rv0240 | 21, 23 |
| Rv0241c | *-* | hypothetical protein Rv0241c | 4, 14, 40, 50, 76, 83, 153, 169, 184, 187, 216, 246, 256, 273 |
| Rv0242c | *fabG* | 3-ketoacyl-(acyl-carrier-protein) reductase | 15, 44, 50, 52, 75, 83, 90, 96, 115, 122, 128, 153, 168, 171, 218, 222, 246, 254, 259, 261, 268, 295, 348, 351, 354, 364, 375, 378, 398, 417, 439 |
| Rv0243 | *fadA2* | acetyl-CoA acetyltransferase | 18, 41, 45, 54, 63, 98, 104, 108, 113, 114, 120, 123, 171, 178, 186, 189, 204, 256, 263, 300, 368, 386, 387, 394, 402, 415, 420, 428, 429, 434 |
| Rv0244c | *fadE5* | PROBABLE ACYL-CoA DEHYDROGENASE FADE5 | 36, 89, 128, 129, 152, 155, 168, 201, 219, 221, 240, 243, 276, 313, 338, 371, 424, 449, 482, 508, 511, 516, 563, 608 |
| Rv0245 | *-* | POSSIBLE OXIDOREDUCTASE | 20, 66, 82, 88, 96, 105 |
| Rv0246 | *-* | PROBABLE CONSERVED INTEGRAL MEMBRANE PROTEIN | 64, 78, 145, 214, 258, 261, 308, 319, 344, 356, 433 |
| Rv0247c | *-* | fumarate reductase iron-sulfur subunit | 10, 57, 167 |
| Rv0248c | *sdhA* | succinate dehydrogenase flavoprotein subunit | 15, 16, 29, 51, 55, 75, 121, 135, 184, 209, 225, 235, 268, 271, 334, 336, 341, 353, 391, 399, 401, 410, 414, 415, 424, 564, 572, 593, 596, 598 |
| Rv0249c | *-* | PROBABLE SUCCINATE DEHYDROGENASE | 80, 141, 180, 212, 213 |
| Rv0250c | *-* | hypothetical protein Rv0250c | 14, 21, 62 |
| Rv0251c | *hsp* | HEAT SHOCK PROTEIN HSP (HEAT-STRESS-INDUCED RIBOSOME-BINDING PROTEIN A) | 27, 37, 43, 60, 84, 95, 118, 132, 140 |
| Rv0252 | *nirB* | PROBABLE NITRITE REDUCTASE | 10, 78, 103, 118, 128, 133, 149, 157, 162, 168, 175, 203, 223, 258, 259, 279, 301, 313, 410, 420, 424, 463, 469, 471, 513, 521, 529, 547, 585, 610, 611, 635, 678, 701, 719, 722, 762, 837 |
| Rv0253 | *nirD* | PROBABLE NITRITE REDUCTASE | 11, 54, 61, 67 |
| Rv0254c | *cobU* | PROBABLE BIFUNCTIONAL COBALAMIN BIOSYNTHESIS PROTEIN COBU: COBINAMIDE KINASE + COBINAMIDE PHOSPHATE GUANYLYLTRANSFERASE | 6, 35, 99, 115, 118, 167 |
| Rv0255c | *cobQ1* | cobyric acid synthase | 12, 18, 28, 116, 125, 136, 170, 218, 250, 334, 369, 381, 388, 401, 404, 410, 451 |
| Rv0256c | *PPE2* | PPE FAMILY PROTEIN | 1, 19, 23, 48, 52, 56, 63, 69, 94, 97, 101, 104, 149, 161, 165, 230, 288, 333, 348, 365, 397, 403, 411, 417, 419, 443, 460, 483, 494, 511, 519, 534, 543 |
| Rv0257 | *-* | hypothetical protein Rv0257 | 26, 30, 32, 108, 112 |
| Rv0258c | *-* | hypothetical protein Rv0258c | 8, 33, 54, 55, 67, 72, 105, 149 |
| Rv0259c | *-* | hypothetical protein Rv0259c | 13, 22, 50, 56, 61, 66, 75, 90, 109, 122, 144, 155, 156, 199 |
| Rv0260c | *-* | bifunctional uroporphyrinogen-III synthetase/response regulator domain protein | 9, 17, 71, 76, 80, 103, 113, 114, 151, 163, 168, 186, 211, 286, 309, 321, 322, 335, 346 |
| Rv0261c | *narK3* | PROBABLE INTEGRAL MEMBRANE NITRITE EXTRUSION PROTEIN NARK3 (NITRITE FACILITATOR) | 19, 34, 61, 62, 71, 87, 99, 127, 131, 134, 139, 156, 164, 183, 202, 206, 264, 293, 302, 317, 331, 353, 397, 404, 452, 460, 462 |
| Rv0262c | *aac* | AMINOGLYCOSIDE 2'-N-ACETYLTRANSFERASE AAC (AAC(2')-IC) | 12, 28, 32, 61, 77, 107, 128, 143 |
| Rv0263c | *-* | hypothetical protein Rv0263c | 8, 20, 30, 32, 59, 66, 109, 118, 145, 167, 180, 226, 232, 253, 275 |
| Rv0264c | *-* | hypothetical protein Rv0264c | 23, 35, 39, 105, 109, 117, 119, 128, 156, 208 |
| Rv0265c | *-* | PROBABLE PERIPLASMIC IRON-TRANSPORT LIPOPROTEIN | 19, 36, 61, 99, 118, 130, 150, 151, 245, 259, 288, 301 |
| Rv0266c | *oplA* | PROBABLE 5-OXOPROLINASE OPLA (5-OXO-L-PROLINASE) (PYROGLUTAMASE) (5-OPASE) | 43, 46, 89, 201, 243, 247, 257, 258, 270, 281, 291, 303, 318, 319, 320, 339, 349, 351, 378, 385, 396, 400, 403, 448, 450, 451, 473, 477, 487, 500, 516, 546, 588, 607, 639, 664, 684, 688, 718, 768, 772, 784, 800, 801, 812, 819, 823, 827, 882, 949, 958, 1016, 1031, 1036, 1045, 1068, 1070, 1112, 1115, 1120, 1140, 1151, 1152, 1155, 1174, 1176, 1192, 1193, 1195 |
| Rv0267 | *narU* | PROBABLE INTEGRAL MEMBRANE NITRITE EXTRUSION PROTEIN NARU (NITRITE FACILITATOR) | 4, 71, 73, 75, 78, 81, 86, 98, 155, 156, 160, 188, 207, 216, 226, 310, 318, 323, 332, 360, 382, 383, 396, 397, 410, 430 |
| Rv0268c | *-* | hypothetical protein Rv0268c | 18, 35, 37, 105, 118 |
| Rv0269c | *-* | hypothetical protein Rv0269c | 4, 27, 53, 56, 70, 99, 108, 109, 129, 146, 170, 176, 199, 264, 288, 315, 343, 345, 351, 360 |
| Rv0270 | *fadD2* | acyl-CoA synthetase | 7, 24, 94, 109, 145, 210, 212, 227, 230, 248, 258, 277, 295, 342, 377, 428, 429, 442, 451, 457, 473, 481, 548 |
| Rv0271c | *fadE6* | PROBABLE ACYL-CoA DEHYDROGENASE FADE6 | 45, 61, 83, 111, 116, 122, 125, 144, 208, 218, 231, 267, 301, 314, 335, 340, 351, 355, 380, 411, 425, 426, 504, 592, 626, 649, 667, 692 |
| Rv0272c | *-* | hypothetical protein Rv0272c | 7, 20, 26, 49, 68, 124, 143, 145, 152, 158, 163, 170, 211, 278, 283, 289, 304, 324 |
| Rv0273c | *-* | POSSIBLE TRANSCRIPTIONAL REGULATORY PROTEIN | 7, 13, 17, 37, 40, 90, 92, 171 |
| Rv0274 | *-* | hypothetical protein Rv0274 | 47, 48, 58, 77, 86, 106, 138, 143 |
| Rv0275c | *-* | POSSIBLE TRANSCRIPTIONAL REGULATORY PROTEIN (POSSIBLY TETR-FAMILY) | 8, 12, 26, 50, 108, 142, 167, 178, 216, 227 |
| Rv0276 | *-* | hypothetical protein Rv0276 | 33, 35, 36, 49, 74, 97, 145, 220, 241, 300 |
| Rv0277c | *-* | hypothetical protein Rv0277c | 13, 83 |
| Rv0278c | *PE_PGRS3* | PE-PGRS FAMILY PROTEIN | 30, 40, 51, 81, 83, 88, 91, 109, 122, 124, 128, 131, 138, 140, 143, 146, 150, 152, 153, 156, 158, 159, 162, 171, 173, 174, 177, 185, 187, 188, 190, 191, 199, 200, 202, 203, 205, 206, 214, 216, 217, 219, 220, 223, 226, 232, 233, 236, 238, 239, 248, 251, 253, 254, 256, 257, 262, 263, 266, 268, 271, 275, 277, 280, 284, 289, 290, 292, 293, 296, 304, 309, 310, 312, 313, 319, 322, 324, 331, 332, 335, 337, 338, 341, 348, 350, 352, 353, 359, 364, 365, 368, 370, 371, 373, 374, 383, 384, 386, 387, 389, 390, 397, 400, 402, 406, 412, 413, 415, 416, 419, 426, 427, 433, 436, 442, 445, 454, 455, 458, 461, 463, 470, 473, 475, 483, 486, 489, 501, 526, 541, 545, 547, 548, 562, 565, 566, 568, 569, 576, 579, 586, 592, 594, 595, 597, 598, 604, 608, 610, 611, 615, 621, 623, 624, 626, 635, 636, 638, 639, 652, 654, 655, 657, 661, 668, 670, 671, 675, 677, 681, 684, 686, 687, 690, 696, 698, 701, 702, 707, 708, 711, 714, 720, 722, 723, 725, 731, 735, 738, 743, 744, 746, 747, 749, 750, 755, 759, 760, 762, 765, 768, 776, 779, 780, 782, 790, 792, 793, 795, 796, 806, 808, 809, 811, 812, 815, 825, 827, 828, 830, 831, 834, 840, 841, 843, 844, 846, 847, 849, 856, 858, 859, 861, 862, 868, 874, 943 |
| Rv0279c | *PE_PGRS4* | PE-PGRS FAMILY PROTEIN | 25, 30, 40, 81, 83, 87, 91, 109, 122, 124, 128, 131, 138, 140, 143, 146, 150, 152, 153, 156, 158, 159, 165, 167, 168, 170, 171, 178, 179, 181, 182, 184, 185, 187, 188, 194, 195, 198, 200, 201, 205, 210, 211, 213, 214, 216, 217, 220, 226, 231, 232, 237, 239, 240, 242, 243, 245, 246, 249, 254, 255, 257, 260, 261, 267, 270, 271, 274, 277, 282, 283, 285, 286, 289, 297, 302, 303, 306, 313, 314, 315, 317, 318, 320, 328, 331, 338, 344, 345, 347, 348, 351, 357, 358, 361, 363, 364, 374, 376, 377, 379, 380, 387, 388, 390, 393, 394, 397, 403, 406, 409, 416, 418, 419, 420, 422, 423, 425, 432, 438, 443, 444, 447, 449, 450, 453, 465, 489, 504, 510, 511, 518, 521, 526, 530, 533, 535, 536, 538, 539, 546, 548, 549, 551, 552, 560, 562, 563, 565, 566, 568, 574, 575, 577, 578, 580, 587, 588, 590, 591, 593, 597, 603, 606, 607, 610, 619, 620, 622, 623, 625, 628, 635, 637, 638, 640, 641, 647, 649, 658, 659, 661, 662, 671, 673, 677, 682, 683, 686, 689, 692, 697, 700, 702, 706, 714, 715, 717, 718, 720, 728, 730, 731, 734, 737, 740, 747, 749, 750, 752, 756, 761, 764, 765, 768, 771, 777, 780, 782, 783, 805, 808, 811, 814, 815, 818, 820, 821, 824 |
| Rv0280 | *PPE3* | PPE FAMILY PROTEIN | 17, 28, 39, 54, 62, 67, 87, 94, 99, 111, 147, 160, 209, 217, 309, 312, 315, 318, 348, 358, 362, 380, 382, 383, 394, 423, 442, 463, 478, 484, 496 |
| Rv0281 | *-* | hypothetical protein Rv0281 | 16, 48, 91, 93, 94, 188, 195, 201, 229, 252, 262, 280, 286, 298 |
| Rv0282 | *-* | hypothetical protein Rv0282 | 8, 20, 62, 68, 82, 85, 87, 97, 125, 134, 144, 151, 164, 171, 175, 220, 299, 315, 323, 330, 386, 388, 402, 455, 485, 537, 595, 597 |
| Rv0283 | *-* | POSSIBLE CONSERVED MEMBRANE PROTEIN | 33, 52, 53, 111, 154, 189, 200, 206, 211, 215, 223, 225, 256, 283, 422, 435, 445, 453, 462, 478, 504, 526 |
| Rv0284 | *-* | POSSIBLE CONSERVED MEMBRANE PROTEIN | 60, 81, 85, 118, 159, 175, 204, 248, 269, 303, 372, 380, 389, 406, 414, 433, 454, 468, 480, 500, 515, 548, 561, 581, 646, 686, 688, 725, 745, 788, 821, 828, 832, 862, 1018, 1020, 1025, 1051, 1097, 1128, 1155, 1179, 1192, 1213, 1232, 1242, 1278, 1282 |
| Rv0285 | *PE5* | PE FAMILY PROTEIN | 10, 24, 35, 49, 51, 64, 74, 81, 87, 91, 93, 100 |
| Rv0286 | *PPE4* | PPE FAMILY PROTEIN | 1, 69, 85, 94, 113, 150, 181, 280, 298, 311, 314, 315, 331, 354, 356, 366, 380, 387, 394, 427, 445, 451, 453, 466, 469 |
| Rv0287 | *esxG* | ESAT-6 LIKE PROTEIN ESXG (CONSERVED HYPOTHETICAL PROTEIN TB9.8) | 18, 52, 61, 78, 79, 84, 89, 94 |
| Rv0288 | *esxH* | LOW MOLECULAR WEIGHT PROTEIN ANTIGEN 7 ESXH (10 kDa ANTIGEN) (CFP-7) (PROTEIN TB10.4) | 8, 14, 18, 36, 46, 90, 94 |
| Rv0289 | *-* | hypothetical protein Rv0289 | 39, 108, 110, 117, 133, 178, 194, 214, 271 |
| Rv0290 | *-* | PROBABLE CONSERVED TRANSMEMBRANE PROTEIN | 1, 24, 34, 54, 66, 93, 94, 97, 108, 131, 141, 151, 155, 161, 170, 188, 218, 240, 253, 268, 285, 331, 364, 376, 390, 398, 400, 405, 447, 448 |
| Rv0291 | *mycP3* | PROBABLE MEMBRANE-ANCHORED MYCOSIN MYCP3 (SERINE PROTEASE) (SUBTILISIN-LIKE PROTEASE) (SUBTILASE-LIKE) (MYCOSIN-3) | 3, 32, 38, 50, 56, 95, 108, 130, 134, 137, 183, 206, 214, 234, 238, 241, 289, 290, 299, 306, 308, 338, 344, 348, 351, 359, 371, 373, 376, 387, 401, 404, 406, 408, 433, 436, 446 |
| Rv0292 | *-* | PROBABLE CONSERVED TRANSMEMBRANE PROTEIN | 7, 40, 52, 62, 76, 124, 126, 158, 180, 198, 224, 243, 244, 253, 265, 267, 315, 316 |
| Rv0293c | *-* | hypothetical protein Rv0293c | 19, 23, 54, 61, 124, 134, 151, 197, 220, 227, 230, 292, 322, 345, 373 |
| Rv0294 | *tam* | PROBABLE TRANS-ACONITATE METHYLTRANSFERASE TAM | 37, 39, 51, 67, 71, 77, 126, 135, 174, 190, 200 |
| Rv0295c | *-* | hypothetical protein Rv0295c | 15, 30, 43, 70, 75, 88, 97, 119, 177, 218, 236 |
| Rv0296c | *-* | PROBABLE SULFATASE | 41, 53, 66, 87, 146, 156, 157, 219, 247, 249, 273, 290, 318, 360, 392, 401, 427, 454, 457 |
| Rv0297 | *PE_PGRS5* | PE-PGRS FAMILY PROTEIN | 14, 31, 36, 42, 51, 63, 69, 91, 94, 100, 105, 125, 128, 129, 139, 142, 144, 147, 150, 151, 153, 154, 157, 166, 169, 171, 175, 177, 178, 180, 181, 188, 191, 193, 194, 199, 203, 206, 208, 209, 212, 215, 221, 224, 227, 229, 232, 251, 261, 263, 264, 266, 280, 283, 286, 300, 303, 304, 307, 309, 310, 315, 319, 331, 336, 342, 343, 346, 351, 352, 354, 355, 358, 362, 369, 371, 375, 378, 380, 381, 383, 384, 387, 390, 395, 406, 407, 410, 412, 420, 422, 423, 425, 432, 439, 445, 448, 451, 453, 460, 463, 466, 474, 478, 481, 484, 487, 490, 493, 501, 502, 504, 507, 511, 513, 514, 517, 519, 520, 522, 532, 535, 537, 538, 541, 544, 547, 549, 552, 553, 556, 558, 559, 561, 568, 580, 581, 589 |
| Rv0298 | *-* | hypothetical protein Rv0298 | 72 |
| Rv0299 | *-* | hypothetical protein Rv0299 | 3, 28, 31, 73, 85 |
| Rv0300 | *-* | hypothetical protein Rv0300 | 48, 59 |
| Rv0301 | *-* | hypothetical protein Rv0301 | 30, 49, 88, 102, 123, 126, 139 |
| Rv0302 | *-* | PROBABLE TRANSCRIPTIONAL REGULATORY PROTEIN (PROBABLY TETR/ACRR-FAMILY) | 3, 43, 62, 67, 74, 134, 164, 177 |
| Rv0303 | *-* | PROBABLE DEHYDROGENASE/REDUCTASE | 2, 4, 8, 12, 40, 89, 120, 137, 146, 156, 208, 214, 247, 277 |
| Rv0304c | *PPE5* | PPE FAMILY PROTEIN | 43, 47, 57, 92, 106, 157, 166, 200, 218, 220, 238, 243, 248, 263, 268, 273, 283, 293, 298, 303, 308, 318, 350, 353, 366, 405, 411, 442, 444, 445, 454, 465, 518, 573, 577, 627, 637, 661, 671, 689, 691, 699, 704, 709, 714, 719, 724, 734, 749, 754, 759, 805, 844, 853, 856, 869, 888, 901, 944, 968, 973, 1013, 1023, 1033, 1035, 1047, 1067, 1075, 1077, 1100, 1101, 1105, 1110, 1115, 1120, 1121, 1130, 1135, 1140, 1150, 1155, 1198, 1251, 1255, 1262, 1263, 1295, 1297, 1299, 1301, 1307, 1361, 1367, 1372, 1374, 1382, 1404, 1444, 1454, 1456, 1464, 1466, 1486, 1498, 1506, 1508, 1521, 1541, 1546, 1551, 1556, 1566, 1576, 1581, 1606, 1611, 1654, 1668, 1713, 1714, 1736, 1745, 1747, 1758, 1764, 1777, 1778, 1816, 1830, 1837, 1850, 1869, 1885, 1890, 1899, 1929, 1949, 1959, 1969, 1991, 1993, 2003, 2011, 2013, 2022, 2026, 2041, 2046, 2056, 2057, 2071, 2086, 2091, 2119, 2126, 2135, 2137, 2146, 2148, 2156, 2188 |
| Rv0305c | *PPE6* | PPE FAMILY PROTEIN | 18, 26, 57, 59, 85, 137, 148, 162, 184, 187, 189, 245, 254, 274, 296, 298, 308, 316, 318, 331, 336, 341, 342, 351, 356, 361, 371, 376, 386, 391, 396, 422, 442, 485, 496, 531, 533, 541, 595, 615, 620, 624, 645, 664, 694, 696, 728, 738, 748, 766, 781, 786, 791, 801, 806 |
| Rv0306 | *-* | PUTATIVE OXIDOREDUCTASE | 24, 25, 39, 42, 77, 165, 186 |
| Rv0307c | *-* | hypothetical protein Rv0307c | 40, 77, 90, 92, 94, 116 |
| Rv0308 | *-* | PROBABLE CONSERVED INTEGRAL MEMBRANE PROTEIN | 16, 77, 137, 188 |
| Rv0309 | *-* | POSSIBLE CONSERVED EXPORTED PROTEIN | 48, 49, 61, 63, 70, 87, 101, 105, 109, 111, 177, 186, 189 |
| Rv0310c | *-* | hypothetical protein Rv0310c | 45, 90, 93, 95, 116, 135 |
| Rv0311 | *-* | hypothetical protein Rv0311 | 111, 140, 228, 275, 289, 290, 308, 350, 361, 368, 391 |
| Rv0312 | *-* | CONSERVED HYPOTHETICAL PROLINE AND THREONINE RICH PROTEIN | 56, 92, 93, 107, 112, 131, 146, 168, 199, 226, 259, 281, 308, 330, 334, 365, 367, 368, 377, 416, 454, 479, 486, 551, 606 |
| Rv0313 | *-* | hypothetical protein Rv0313 | 38, 61, 62, 65 |
| Rv0314c | *-* | POSSIBLE CONSERVED MEMBRANE PROTEIN | 36, 41, 47, 57, 61, 69, 78, 132, 139, 157, 166, 175, 191, 192, 210, 211 |
| Rv0315 | *-* | POSSIBLE BETA-1,3-GLUCANASE PRECURSOR | 21, 24, 36, 45, 52, 65, 156, 168, 195, 218, 239, 256, 269, 273, 276 |
| Rv0316 | *-* | POSSIBLE MUCONOLACTONE ISOMERASE | 15, 59, 140 |
| Rv0317c | *glpQ2* | POSSIBLE GLYCEROPHOSPHORYL DIESTER PHOSPHODIESTERASE GLPQ2 (GLYCEROPHOSPHODIESTER PHOSPHODIESTERASE) | 62, 67, 97, 165, 192, 203, 249 |
| Rv0318c | *-* | PROBABLE CONSERVED INTEGRAL MEMBRANE PROTEIN | 29, 39, 43, 64, 70, 73, 150, 174, 176, 183, 197, 251 |
| Rv0319 | *pcp* | pyrrolidone-carboxylate peptidase | 6, 22, 32, 97, 108, 109, 130, 165, 177, 195, 208 |
| Rv0320 | *-* | POSSIBLE CONSERVED EXPORTED PROTEIN | 23, 30, 37, 69, 70, 127, 150, 152, 193, 195, 207, 212 |
| Rv0321 | *dcd* | deoxycytidine triphosphate deaminase | 13, 94, 113, 122, 141, 174 |
| Rv0322 | *udgA* | PROBABLE UDP-GLUCOSE 6-DEHYDROGENASE UDGA (UDP-GLC DEHYDROGENASE) (UDP-GLCDH) (UDPGDH) | 32, 39, 48, 60, 126, 134, 204, 211, 262, 277, 309, 311, 338, 401 |
| Rv0323c | *-* | hypothetical protein Rv0323c | 69, 107, 168, 186, 195 |
| Rv0324 | *-* | POSSIBLE TRANSCRIPTIONAL REGULATORY PROTEIN (POSSIBLY ARSR-FAMILY) | 47, 63, 107, 129, 145, 211 |
| Rv0325 | *-* | hypothetical protein Rv0325 | 45, 62 |
| Rv0326 | *-* | hypothetical protein Rv0326 | 20, 23, 41, 86, 93, 122, 149 |
| Rv0327c | *cyp135A1* | POSSIBLE CYTOCHROME P450 135A1 CYP135A1 | 10, 108, 113, 116, 181, 226, 261, 266, 268, 293, 316, 321, 376, 406, 408, 440 |
| Rv0328 | *-* | POSSIBLE TRANSCRIPTIONAL REGULATORY PROTEIN (POSSIBLY TETR/ACRR-FAMILY) | 21, 77, 78, 81, 100, 123, 124, 142, 175 |
| Rv0329c | *-* | hypothetical protein Rv0329c | 33, 46, 59, 67, 69, 74, 96, 144, 165, 176, 183, 203 |
| Rv0330c | *-* | hypothetical protein Rv0330c | 5, 10, 14, 77, 123, 160, 179, 185 |
| Rv0331 | *-* | POSSIBLE DEHYDROGENASE/REDUCTASE | 12, 16, 63, 68, 113, 153, 161, 171, 180, 229, 255, 266, 311, 332, 341 |
| Rv0332 | *-* | hypothetical protein Rv0332 | 53, 77, 93, 168, 170, 195, 205, 222, 240, 248 |
| Rv0333 | *-* | hypothetical protein Rv0333 | 17, 47, 56, 77, 86, 88 |
| Rv0334 | *rmlA* | ALPHA-D-GLUCOSE-1-PHOSPHATE THYMIDYLYLTRANSFERASE RMLA (DTDP-GLUCOSE SYNTHASE) (DTDP-GLUCOSE PYROPHOSPHORYLASE) | 1, 6, 7, 58, 190, 215, 222, 231, 274 |
| Rv0335c | *PE6* | PE FAMILY PROTEIN | 33, 88, 125, 136 |
| Rv0336 | *-* | CONSERVED 13E12 REPEAT FAMILY PROTEIN | 41, 60, 75, 112, 144, 242, 258, 296, 315, 344, 349, 362, 404, 425, 471, 482 |
| Rv0337c | *aspC* | aminotransferase AlaT | 18, 41, 48, 112, 164, 205, 269, 279, 315, 326, 327, 391, 396, 414 |
| Rv0338c | *-* | PROBABLE IRON-SULFUR-BINDING REDUCTASE | 15, 35, 40, 119, 152, 203, 277, 297, 309, 342, 345, 362, 525, 630, 648, 651, 679, 683, 703, 706, 734, 745, 750, 759, 769, 771, 781, 782, 796, 802, 812, 813, 818, 847, 863, 873 |
| Rv0339c | *-* | POSSIBLE TRANSCRIPTIONAL REGULATORY PROTEIN | 47, 60, 85, 117, 156, 167, 173, 222, 231, 239, 302, 318, 322, 338, 343, 362, 398, 404, 421, 478, 482, 498, 512, 534, 546, 557, 594, 620, 667, 685, 707, 717, 738, 740, 747, 826 |
| Rv0340 | *-* | hypothetical protein Rv0340 | 18, 22, 61, 63, 76, 111, 125 |
| Rv0341 | *iniB* | ISONIAZID INDUCTIBLE GENE PROTEIN INIB | 16, 22, 31, 56, 61, 68, 83, 104, 119, 122, 126, 165, 170, 181, 182, 190, 195, 198, 202, 224, 237, 242, 251, 264, 290, 298, 300, 302, 304, 338, 341, 344, 346, 349, 371, 390, 392, 393, 396, 476 |
| Rv0342 | *iniA* | ISONIAZID INDUCTIBLE GENE PROTEIN INIA | 2, 3, 75, 93, 122, 123, 129, 149, 168, 179, 491, 515, 624 |
| Rv0343 | *iniC* | ISONIAZID INDUCTIBLE GENE PROTEIN INIC | 54, 92, 96, 109, 163, 182, 196, 249, 255, 271, 323, 329, 332, 339, 363, 432, 440, 449, 452 |
| Rv0344c | *lpqJ* | PROBABLE LIPOPROTEIN LPQJ | 16, 20, 50, 56, 68, 71, 90, 125, 126, 127 |
| Rv0345 | *-* | hypothetical protein Rv0345 | 11, 31, 57, 60, 63, 117, 122 |
| Rv0346c | *ansP2* | POSSIBLE L-ASPARAGINE PERMEASE ANSP2 (L-ASPARAGINE TRANSPORT PROTEIN) | 37, 43, 44, 52, 61, 101, 118, 194, 208, 225, 234, 273, 304, 312, 317, 329, 334, 340, 345, 350, 351, 376, 400, 416, 425, 443 |
| Rv0347 | *-* | PROBABLE CONSERVED MEMBRANE PROTEIN | 1, 12, 122, 136, 197, 222, 244, 247 |
| Rv0348 | *-* | POSSIBLE TRANSCRIPTIONAL REGULATORY PROTEIN | 68, 114, 139, 149 |
| Rv0349 | *-* | hypothetical protein Rv0349 |  |
| Rv0350 | *dnaK* | molecular chaperone DnaK | 52, 86, 118, 132, 136, 148, 150, 199, 219, 236, 299, 319, 329, 330, 352, 378, 401, 421, 433, 460, 466, 545, 555, 596, 597, 601, 605 |
| Rv0351 | *grpE* | PROBABLE GRPE PROTEIN (HSP-70 COFACTOR) | 35, 58, 82, 127, 155, 208 |
| Rv0352 | *dnaJ1* | PROBABLE CHAPERONE PROTEIN DNAJ1 | 46, 47, 78, 79, 83, 89, 93, 96, 115, 119, 120, 134, 145, 160, 182, 221, 235, 250, 261, 266, 269, 329, 338, 340, 349, 367, 388 |
| Rv0353 | *hspR* | PROBABLE HEAT SHOCK PROTEIN TRANSCRIPTIONAL REPRESSOR HSPR (MERR FAMILY) | 17, 21, 43, 45, 72, 116 |
| Rv0354c | *PPE7* | PPE FAMILY PROTEIN | 54, 56, 88, 90, 100, 102, 121 |
| Rv0355c | *PPE8* | PPE FAMILY PROTEIN | 18, 28, 40, 68, 94, 137, 155, 176, 184, 228, 246, 257, 267, 277, 289, 295, 301, 311, 321, 335, 340, 350, 355, 360, 365, 375, 376, 390, 395, 400, 405, 420, 431, 447, 449, 453, 458, 476, 518, 558, 569, 580, 600, 610, 643, 653, 663, 674, 680, 683, 700, 710, 723, 738, 753, 754, 763, 773, 783, 788, 793, 843, 863, 888, 927, 934, 944, 1009, 1013, 1016, 1044, 1054, 1087, 1097, 1107, 1117, 1128, 1134, 1142, 1154, 1162, 1164, 1167, 1172, 1182, 1187, 1197, 1202, 1207, 1222, 1237, 1242, 1252, 1271, 1285, 1294, 1316, 1328, 1330, 1332, 1342, 1345, 1352, 1385, 1387, 1401, 1409, 1437, 1464, 1491, 1503, 1513, 1546, 1556, 1566, 1576, 1586, 1591, 1593, 1596, 1613, 1623, 1631, 1636, 1641, 1646, 1651, 1656, 1661, 1666, 1671, 1677, 1691, 1696, 1706, 1707, 1711, 1739, 1756, 1773, 1785, 1809, 1818, 1820, 1821, 1853, 1855, 1869, 1892, 1893, 1902, 1919, 1920, 1929, 1947, 1982, 2015, 2025, 2035, 2045, 2057, 2059, 2062, 2079, 2092, 2102, 2109, 2119, 2132, 2142, 2157, 2162, 2172, 2177, 2182, 2187, 2192, 2278, 2291, 2292, 2303, 2305, 2319, 2326, 2335, 2337, 2356, 2359, 2374, 2411, 2415, 2470, 2484, 2500, 2510, 2520, 2530, 2542, 2572, 2582, 2592, 2597, 2604, 2606, 2609, 2614, 2616, 2619, 2624, 2626, 2629, 2636, 2646, 2647, 2654, 2662, 2678, 2688, 2696, 2698, 2701, 2706, 2711, 2721, 2731, 2741, 2751, 2756, 2761, 2766, 2771, 2776, 2805, 2819, 2828, 2838, 2861, 2899, 2901, 2903, 2917, 2924, 2986, 3100, 3110, 3112, 3120, 3130, 3135, 3138, 3144, 3152, 3154, 3162, 3164, 3172, 3177, 3182, 3187, 3192, 3197, 3202, 3207, 3212, 3217, 3222, 3227, 3228, 3232, 3237, 3247, 3252, 3257, 3279 |
| Rv0356c | *-* | hypothetical protein Rv0356c | 28, 51, 63, 82, 86, 89, 124, 128, 153, 173, 210 |
| Rv0357c | *purA* | adenylosuccinate synthetase | 38, 46, 62, 76, 89, 93, 99, 128, 162, 182, 213, 274, 300, 317, 381, 390, 404 |
| Rv0358 | *-* | hypothetical protein Rv0358 | 7, 14, 20, 24, 78, 87, 110, 146, 166, 174, 177, 183 |
| Rv0359 | *-* | PROBABLE CONSERVED INTEGRAL MEMBRANE PROTEIN | 32, 42, 50, 66, 78, 105, 112, 161, 172, 244 |
| Rv0360c | *-* | hypothetical protein Rv0360c | 43, 74, 78, 114, 116, 136 |
| Rv0361 | *-* | PROBABLE CONSERVED MEMBRANE PROTEIN | 10, 13, 18, 22, 54, 68, 70, 105, 157, 182, 186 |
| Rv0362 | *mgtE* | POSSIBLE Mg2+ TRANSPORT TRANSMEMBRANE PROTEIN MGTE | 4, 85, 97, 135, 173, 304, 331, 332, 335, 349, 367, 376, 378, 401 |
| Rv0363c | *fba* | fructose-bisphosphate aldolase | 35, 43, 53, 60, 69, 152, 165, 195, 204, 216, 252, 287, 337 |
| Rv0364 | *-* | POSSIBLE CONSERVED TRANSMEMBRANE PROTEIN | 2, 25, 29, 39, 54, 55, 59, 85, 143, 160, 199 |
| Rv0365c | *-* | hypothetical protein Rv0365c | 11, 111, 135, 155, 198, 224, 256, 257, 268, 287, 292, 329, 330, 331, 364, 368 |
| Rv0366c | *-* | hypothetical protein Rv0366c | 7, 12, 27, 57, 88, 92, 117, 119, 167 |
| Rv0367c | *-* | hypothetical protein Rv0367c | 11, 80, 97 |
| Rv0368c | *-* | hypothetical protein Rv0368c | 25, 30, 32, 104, 116, 134, 137, 243, 299, 308, 320, 369, 389, 399 |
| Rv0369c | *-* | POSSIBLE MEMBRANE OXIDOREDUCTASE | 29, 54, 56, 60, 143 |
| Rv0370c | *-* | POSSIBLE OXIDOREDUCTASE | 21, 43, 50, 95, 125, 155, 205, 213, 216, 239, 260, 266 |
| Rv0371c | *-* | hypothetical protein Rv0371c | 1, 6, 11, 12, 43, 53, 61, 78, 96, 163 |
| Rv0372c | *-* | hypothetical protein Rv0372c | 6, 11, 49, 67, 87, 98, 99, 131, 160, 186, 233, 235, 248 |
| Rv0373c | *-* | PROBABLE CARBON MONOXYDE DEHYDROGENASE (LARGE CHAIN) | 21, 36, 38, 72, 84, 89, 178, 222, 245, 260, 271, 300, 313, 338, 345, 352, 359, 365, 373, 374, 461, 478, 565, 607, 621, 630, 660, 674, 689, 713, 723, 732 |
| Rv0374c | *-* | PROBABLE CARBON MONOXYDE DEHYDROGENASE (SMALL CHAIN) | 12, 32, 43, 66, 98, 108, 136 |
| Rv0375c | *-* | PROBABLE CARBON MONOXYDE DEHYDROGENASE (MEDIUM CHAIN) | 18, 19, 53, 100, 104, 150, 186, 188, 190, 201, 220, 227, 240, 251, 253, 261, 271, 273 |
| Rv0376c | *-* | hypothetical protein Rv0376c | 4, 5, 8, 27, 43, 54, 60, 74, 83, 206, 335, 349, 359 |
| Rv0377 | *-* | PROBABLE TRANSCRIPTIONAL REGULATORY PROTEIN (PROBABLY LYSR-FAMILY) | 20, 29, 50, 52, 58, 59, 77, 84, 94, 103, 178, 184, 197, 198, 210, 226, 251, 252, 259, 266, 281, 303 |
| Rv0378 | *-* | CONSERVED HYPOTHETICAL GLYCINE RICH PROTEIN | 6, 15, 21, 24, 25, 28, 30, 34, 36, 53, 56, 59, 62, 68, 71 |
| Rv0379 | *secE2* | POSSIBLE PROTEIN TRANSPORT PROTEIN SECE2 | 49 |
| Rv0380c | *-* | POSSIBLE RNA METHYLTRANSFERASE (RNA METHYLASE) | 52, 72, 90, 99, 118, 133, 141, 160, 163, 164 |
| Rv0381c | *-* | hypothetical protein Rv0381c | 9, 15, 26, 37, 56, 95, 101, 119, 138, 153, 158, 172, 203, 225, 228, 236, 241, 243, 295 |
| Rv0382c | *pyrE* | orotate phosphoribosyltransferase | 1, 46, 66, 77, 111, 124, 127, 153 |
| Rv0383c | *-* | POSSIBLE CONSERVED SECRETED PROTEIN | 27, 57, 67, 77, 153, 209, 214, 225, 227, 271 |
| Rv0384c | *clpB* | PROBABLE ENDOPEPTIDASE ATP BINDING PROTEIN (CHAIN B) CLPB (CLPB PROTEIN) (HEAT SHOCK PROTEIN F84.1) | 14, 22, 23, 30, 45, 56, 92, 116, 126, 143, 168, 207, 212, 224, 246, 251, 269, 285, 288, 295, 305, 327, 351, 382, 483, 498, 545, 549, 557, 579, 590, 608, 652, 660, 732, 795, 824 |
| Rv0385 | *-* | hypothetical protein Rv0385 | 50, 122, 123, 135, 137, 144, 214, 228, 235, 247, 254, 257, 321, 331, 338, 349, 359, 370 |
| Rv0386 | *-* | PROBABLE TRANSCRIPTIONAL REGULATORY PROTEIN (PROBABLY LUXR/UHPA-FAMILY) | 46, 74, 95, 107, 114, 199, 219, 221, 222, 238, 285, 368, 412, 413, 418, 465, 492, 538, 559, 613, 632, 634, 662, 675, 718, 727, 753, 755, 801, 805, 823, 826, 827, 884, 922, 936, 942, 954, 998, 1021, 1080 |
| Rv0387c | *-* | hypothetical protein Rv0387c | 29, 72, 75, 97, 100, 103, 107, 129, 145, 153, 155, 158, 161, 163, 166, 173, 176, 179, 181, 188, 197, 200, 201, 210, 214, 228 |
| Rv0388c | *PPE9* | PPE FAMILY PROTEIN | 11, 16, 18, 27, 38, 49, 52, 66, 78, 91, 159, 167 |
| Rv0389 | *purT* | phosphoribosylglycinamide formyltransferase 2 | 95, 136, 184, 191, 252, 275, 285, 309, 345, 362, 370, 385, 393 |
| Rv0390 | *-* | hypothetical protein Rv0390 | 3, 88 |
| Rv0391 | *metZ* | O-succinylhomoserine sulfhydrylase | 24, 35, 43, 93, 98, 110, 116, 185, 228, 249, 314, 316, 361, 375 |
| Rv0392c | *ndhA* | PROBABLE MEMBRANE NADH DEHYDROGENASE NDHA | 11, 24, 59, 117, 118, 135, 147, 156, 180, 215, 217, 249, 263, 277, 327, 330, 389, 427, 456 |
| Rv0393 | *-* | CONSERVED 13E12 REPEAT FAMILY PROTEIN | 15, 21, 78, 104, 136, 168, 187, 202, 234, 250, 251, 286, 367, 371, 381, 385, 404 |
| Rv0394c | *-* | POSSIBLE SECRETED PROTEIN | 13, 16, 52, 77, 94, 96, 131, 165, 180, 187, 208, 226 |
| Rv0395 | *-* | hypothetical protein Rv0395 | 33, 57 |
| Rv0396 | *-* | hypothetical protein Rv0396 | 58, 96, 110 |
| Rv0397 | *-* | CONSERVED 13E12 REPEAT FAMILY PROTEIN | 44, 58, 62, 81, 100, 103 |
| Rv0398c | *-* | POSSIBLE SECRETED PROTEIN | 10, 12, 20, 24, 30, 41, 50, 82, 84, 109, 113, 151, 179, 189, 206 |
| Rv0399c | *lpqK* | POSSIBLE CONSERVED LIPOPROTEIN LPQK | 17, 24, 63, 77, 83, 139, 202, 203, 224, 230, 235, 252, 285, 309, 337, 348, 351 |
| Rv0400c | *fadE7* | ACYL-CoA DEHYDROGENASE FADE7 | 6, 78, 94, 97, 128, 132, 151, 167, 186, 193, 205, 231, 237, 240, 286, 343, 349, 391 |
| Rv0401 | *-* | PROBABLE CONSERVED TRANSMEMBRANE PROTEIN | 10, 34, 45, 55, 61, 90, 98, 101, 110 |
| Rv0402c | *mmpL1* | PROBABLE CONSERVED TRANSMEMBRANE TRANSPORT PROTEIN MMPL1 | 6, 11, 123, 155, 196, 232, 285, 306, 343, 377, 382, 647, 669, 713, 728, 788, 855, 862, 864, 871 |
| Rv0403c | *mmpS1* | PROBABLE CONSERVED MEMBRANE PROTEIN MMPS1 | 40, 83, 96, 105, 140 |
| Rv0404 | *fadD30* | acyl-CoA synthetase | 28, 45, 63, 84, 111, 150, 203, 266, 321, 414, 488, 552, 560 |
| Rv0405 | *pks6* | PROBABLE MEMBRANE BOUND POLYKETIDE SYNTHASE PKS6 | 49, 155, 239, 240, 324, 330, 334, 404, 450, 467, 511, 537, 539, 621, 635, 726, 731, 744, 754, 755, 756, 805, 853, 887, 897, 899, 951, 982, 984, 1000, 1003, 1007, 1016, 1026, 1036, 1065, 1072, 1097, 1099, 1199, 1227, 1311, 1370 |
| Rv0406c | *-* | BETA LACTAMASE LIKE PROTEIN | 4, 9, 34, 47, 54, 57, 146, 150, 156, 161, 165, 166, 182, 195, 209, 215 |
| Rv0407 | *fgd1* | PROBABLE F420-DEPENDENT GLUCOSE-6-PHOSPHATE DEHYDROGENASE FGD1 | 25, 47, 58, 82, 106, 115, 143, 174, 175, 176, 184, 187, 195, 214, 216, 270 |
| Rv0408 | *pta* | phosphate acetyltransferase | 9, 29, 163, 174, 217, 232, 236, 253, 282, 291, 295, 306, 313, 317, 321, 334, 346, 400, 550, 567, 603, 620, 633 |
| Rv0409 | *ackA* | acetate kinase | 9, 23, 28, 29, 66, 78, 142, 174, 197, 203, 239, 255, 275, 311, 312, 380 |
| Rv0410c | *pknG* | SERINE/THREONINE-PROTEIN KINASE PKNG (PROTEIN KINASE G) (STPK G) | 11, 20, 23, 74, 130, 142, 147, 159, 185, 235, 319, 383, 388, 403, 444, 450, 548, 566, 576, 578, 582, 648, 663 |
| Rv0411c | *glnH* | PROBABLE GLUTAMINE-BINDING LIPOPROTEIN GLNH (GLNBP) | 25, 68, 82, 90, 111, 115, 122, 143, 148, 254, 284, 318 |
| Rv0412c | *-* | POSSIBLE CONSERVED MEMBRANE PROTEIN | 17, 32, 65, 90, 92, 94, 103, 125, 147, 199, 236, 269, 283, 298, 377, 406, 414, 422 |
| Rv0413 | *mutT3* | POSSIBLE MUTATOR PROTEIN MUTT3 (7,8-DIHYDRO-8-OXOGUANINE-TRIPHOSPHATASE) (8-OXO-DGTPASE) (DGTP PYROPHOSPHOHYDROLASE) | 23, 34, 47, 61, 68, 80, 92, 104, 108, 124, 154, 157, 165, 190 |
| Rv0414c | *thiE* | thiamine-phosphate pyrophosphorylase | 20, 29, 32, 33, 46, 61, 89, 129, 131, 162, 166, 176, 189 |
| Rv0415 | *thiO* | POSSIBLE THIAMINE BIOSYNTHESIS OXIDOREDUCTASE THIO | 6, 13, 25, 28, 46, 47, 58, 89, 114, 197, 256, 287, 311, 316, 337 |
| Rv0416 | *thiS* | sulfur carrier protein ThiS | 18 |
| Rv0417 | *thiG* | thiazole synthase | 54, 71, 77, 145, 159, 174, 188, 192, 212, 219, 223, 227, 228 |
| Rv0418 | *lpqL* | PROBABLE LIPOPROTEIN AMINOPEPTIDASE LPQL | 38, 40, 95, 105, 119, 147, 165, 173, 185, 188, 205, 230, 278, 367, 377, 390, 393, 420, 427, 435, 437, 459, 480 |
| Rv0419 | *lpqM* | POSSIBLE LIPOPROTEIN PEPTIDASE LPQM | 3, 19, 36, 60, 66, 209, 228, 237, 290, 302, 307, 341, 352, 366, 405, 414, 422, 437, 444, 453, 472, 478, 482, 485 |
| Rv0420c | *-* | POSSIBLE TRANSMEMBRANE PROTEIN | 65, 92, 113, 131 |
| Rv0421c | *-* | hypothetical protein Rv0421c | 6, 13, 27, 62, 66, 74, 84, 93, 99, 109, 163, 178 |
| Rv0422c | *thiD* | phosphomethylpyrimidine kinase | 9, 13, 15, 17, 60, 74, 77, 90, 126, 176, 205, 210, 217, 220, 252 |
| Rv0423c | *thiC* | thiamine biosynthesis protein ThiC | 26, 28, 43, 53, 66, 73, 88, 99, 115, 178, 240, 250, 255, 260, 266, 313, 394, 396, 426, 467, 483, 490, 507, 523, 524 |
| Rv0424c | *-* | hypothetical protein Rv0424c | 31, 67 |
| Rv0425c | *ctpH* | POSSIBLE METAL CATION TRANSPORTING P-TYPE ATPASE CTPH | 12, 16, 22, 33, 37, 54, 78, 82, 100, 147, 173, 177, 184, 223, 232, 255, 260, 290, 317, 325, 327, 345, 393, 395, 424, 429, 448, 457, 487, 534, 550, 563, 572, 588, 592, 597, 601, 623, 661, 669, 724, 751, 757, 775, 788, 804, 807, 834, 840, 842, 864, 889, 890, 895, 911, 918, 935, 966, 1021, 1030, 1032, 1035, 1043, 1048, 1052, 1084, 1099, 1155, 1161, 1163, 1180, 1220, 1235, 1239, 1257, 1272, 1293, 1309, 1330, 1348, 1362, 1368, 1380, 1396, 1414, 1420, 1451, 1491, 1495 |
| Rv0426c | *-* | POSSIBLE TRANSMEMBRANE PROTEIN | 4, 24, 31, 34, 38, 41, 43, 53, 58, 61, 83, 99, 116, 133, 139 |
| Rv0427c | *xthA* | PROBABLE EXODEOXYRIBONUCLEASE III PROTEIN XTHA (EXONUCLEASE III) (EXO III) (AP ENDONUCLEASE VI) | 7, 115, 127, 156, 170, 196, 228, 259, 289 |
| Rv0428c | *-* | hypothetical protein Rv0428c | 17, 18, 44, 68, 77, 83, 97, 104, 164, 234, 239, 241, 243, 259, 263, 271 |
| Rv0429c | *def* | peptide deformylase | 23, 45, 68, 74, 78, 109, 115, 124, 138, 149, 181, 187 |
| Rv0430 | *-* | hypothetical protein Rv0430 | 38, 71, 92 |
| Rv0431 | *-* | PUTATIVE TUBERCULIN RELATED PEPTIDE | 15, 71, 89, 100, 131, 155, 162 |
| Rv0432 | *sodC* | PROBABLE PERIPLASMIC SUPEROXIDE DISMUTASE | 3, 18, 24, 26, 50, 53, 66, 85, 102, 109, 130, 131, 134, 139, 140, 141, 150, 153, 155, 164, 185, 189, 195, 224, 227, 233, 238 |
| Rv0433 | *-* | hypothetical protein Rv0433 | 6, 40, 65, 71, 84, 92, 178, 193, 194, 216, 272, 291, 336 |
| Rv0434 | *-* | hypothetical protein Rv0434 | 42, 59, 142, 165, 207 |
| Rv0435c | *-* | PUTATIVE CONSERVED ATPASE | 12, 24, 48, 53, 57, 60, 64, 103, 141, 168, 192, 199, 243, 255, 284, 285, 303, 317, 332, 336, 337, 388, 469, 498, 506, 508, 520, 555, 572, 586, 635, 643, 661, 668, 678, 681, 689, 693, 699 |
| Rv0436c | *pssA* | PROBABLE CDP-DIACYLGLYCEROL--SERINE O-PHOSPHATIDYLTRANSFERASE PSSA (PS SYNTHASE) (PHOSPHATIDYLSERINE SYNTHASE) | 5, 29, 41, 47, 86, 129, 140, 182, 200, 212 |
| Rv0437c | *psd* | phosphatidylserine decarboxylase | 18, 26, 32, 33, 48, 55, 79, 123, 169, 221 |
| Rv0438c | *moeA2* | PROBABLE MOLYBDOPTERIN BIOSYNTHESIS PROTEIN MOEA2 | 33, 48, 78, 79, 97, 103, 116, 141, 142, 161, 207, 215, 220, 222, 223, 247, 252, 283, 301, 338, 345, 394 |
| Rv0439c | *-* | short chain dehydrogenase | 1, 19, 26, 30, 36, 43, 59, 66, 89, 107, 136, 147, 180, 211, 215, 254, 262, 275, 304 |
| Rv0440 | *groEL* | chaperonin GroEL | 12, 83, 107, 116, 145, 240, 265, 276, 293, 333, 340, 370, 378, 380, 410, 434, 454, 478, 487, 529, 531 |
| Rv0441c | *-* | hypothetical protein Rv0441c | 11, 83, 104, 107 |
| Rv0442c | *PPE10* | PPE FAMILY PROTEIN | 19, 21, 31, 63, 89, 97, 194, 196, 211, 214, 216, 221, 224, 226, 244, 246, 254, 262, 268, 278, 286, 288, 301, 331, 346, 347, 351, 352, 366, 372, 392, 400, 409, 411, 414, 447, 455, 457, 472 |
| Rv0443 | *-* | hypothetical protein Rv0443 | 5, 64, 88, 103, 126, 162 |
| Rv0444c | *-* | hypothetical protein Rv0444c | 33, 40, 62, 94, 138, 187, 205, 213, 215, 221 |
| Rv0445c | *sigK* | RNA polymerase sigma factor SigK | 1, 23, 47, 70, 74, 96, 111, 122, 150 |
| Rv0446c | *-* | POSSIBLE CONSERVED TRANSMEMBRANE PROTEIN | 43, 78, 91, 120, 123, 165, 220, 233 |
| Rv0447c | *ufaA1* | PROBABLE CYCLOPROPANE-FATTY-ACYL-PHOSPHOLIPID SYNTHASE UFAA1 (CYCLOPROPANE FATTY ACID SYNTHASE) (CFA SYNTHASE) | 25, 29, 61, 95, 160, 183, 203, 216, 226, 250, 252, 294, 335, 348, 352 |
| Rv0448c | *-* | hypothetical protein Rv0448c | 58, 60, 70, 109, 138, 200 |
| Rv0449c | *-* | hypothetical protein Rv0449c | 16, 19, 40, 52, 107, 113, 139, 181, 186, 221, 224, 328, 356, 399, 412 |
| Rv0450c | *mmpL4* | PROBABLE CONSERVED TRANSMEMBRANE TRANSPORT PROTEIN MMPL4 | 130, 149, 172, 182, 185, 188, 239, 248, 268, 286, 292, 299, 301, 313, 420, 427, 500, 654, 676, 734, 753, 794, 862, 869, 871, 924, 941, 953 |
| Rv0451c | *mmpS4* | PROBABLE CONSERVED MEMBRANE PROTEIN MMPS4 | 25, 60, 138 |
| Rv0452 | *-* | POSSIBLE TRANSCRIPTIONAL REGULATORY PROTEIN | 30, 52, 74, 109, 170, 209, 216, 224 |
| Rv0453 | *PPE11* | PPE FAMILY PROTEIN | 29, 42, 57, 70, 87, 97, 101, 192, 210, 211, 220, 234, 304, 313, 316, 326, 341, 348, 355, 356, 360, 364, 366, 378, 379, 388, 399, 400, 407, 448, 468, 473, 475, 485, 515 |
| Rv0454 | *-* | hypothetical protein Rv0454 | 47, 49, 69 |
| Rv0455c | *-* | hypothetical protein Rv0455c | 9, 19, 138 |
| Rv0456c | *echA2* | enoyl-CoA hydratase | 13, 40, 57, 59, 64, 65, 70, 96, 100, 126, 159, 182, 186, 243, 262, 270 |
| Rv0456A | *-* | hypothetical protein Rv0456A | 2, 14, 35, 63 |
| Rv0457c | *-* | PROBABLE PEPTIDASE | 19, 66, 77, 81, 119, 121, 138, 184, 191, 230, 291, 296, 304, 319, 348, 360, 369, 392, 442, 472, 482, 484, 508, 517, 521, 529, 542, 568, 619, 647, 671 |
| Rv0458 | *-* | PROBABLE ALDEHYDE DEHYDROGENASE | 6, 8, 9, 43, 64, 75, 95, 115, 129, 181, 189, 194, 210, 223, 239, 264, 277, 368, 376, 380, 381, 391, 432, 445, 451, 463, 469, 474 |
| Rv0459 | *-* | hypothetical protein Rv0459 | 3, 9, 12, 31, 32, 38, 92, 94, 128, 134 |
| Rv0460 | *-* | CONSERVED HYDROPHOBIC PROTEIN | 46 |
| Rv0461 | *-* | PROBABLE TRANSMEMBRANE PROTEIN | 5, 16, 54, 64, 105, 149 |
| Rv0462 | *lpd* | dihydrolipoamide dehydrogenase | 10, 12, 13, 21, 37, 71, 94, 127, 141, 176, 230, 240, 326, 331, 431 |
| Rv0463 | *-* | PROBABLE CONSERVED MEMBRANE PROTEIN | 21, 56, 60, 67 |
| Rv0464c | *-* | hypothetical protein Rv0464c | 1, 29, 58, 67, 145, 175, 179 |
| Rv0465c | *-* | PROBABLE TRANSCRIPTIONAL REGULATORY PROTEIN | 16, 21, 118, 122, 161, 209, 230, 269, 317, 339, 346, 389, 395, 434, 450 |
| Rv0466 | *-* | hypothetical protein Rv0466 | 107 |
| Rv0467 | *icl* | isocitrate lyase | 30, 50, 72, 82, 95, 101, 112, 155, 158, 172, 190, 194, 249, 285, 292, 348, 381, 388, 398, 415, 418 |
| Rv0468 | *fadB2* | 3-hydroxybutyryl-CoA dehydrogenase | 12, 17, 26, 41, 57, 129, 164, 166, 188, 209 |
| Rv0469 | *umaA* | POSSIBLE MYCOLIC ACID SYNTHASE UMAA | 62, 71, 200, 265 |
| Rv0470c | *pcaA* | MYCOLIC ACID SYNTHASE PCAA (CYCLOPROPANE SYNTHASE) | 63, 72, 99, 200, 222, 266 |
| Rv0470A | *-* | hypothetical protein Rv0470A | 86, 98, 138 |
| Rv0471c | *-* | hypothetical protein Rv0471c | 48, 108, 110, 115, 126 |
| Rv0472c | *-* | PROBABLE TRANSCRIPTIONAL REGULATORY PROTEIN (POSSIBLY TETR-FAMILY) | 72, 156, 160, 161, 172, 205, 210, 211 |
| Rv0473 | *-* | POSSIBLE CONSERVED TRANSMEMBRANE PROTEIN | 44, 65, 93, 106, 178, 246, 335, 402 |
| Rv0474 | *-* | PROBABLE TRANSCRIPTIONAL REGULATORY PROTEIN | 7, 43, 55, 61, 83 |
| Rv0475 | *hbhA* | IRON-REGULATED HEPARIN BINDING HEMAGGLUTININ HBHA (ADHESIN) | 15, 75, 100, 103, 150, 168, 174, 186 |
| Rv0476 | *-* | POSSIBLE CONSERVED TRANSMEMBRANE PROTEIN | 27, 63, 67 |
| Rv0477 | *-* | POSSIBLE CONSERVED SECRETED PROTEIN | 19, 28, 77, 96, 114, 136 |
| Rv0478 | *deoC* | deoxyribose-phosphate aldolase | 10, 28, 34, 55, 86, 102, 117, 120, 148, 152, 166, 167, 190, 195, 205, 213 |
| Rv0479c | *-* | PROBABLE CONSERVED MEMBRANE PROTEIN | 33, 38, 41, 74, 125, 155, 180, 249, 322, 337 |
| Rv0480c | *-* | POSSIBLE AMIDOHYDROLASE | 9, 13, 24, 27, 29, 77, 83, 95, 126, 151, 160, 173, 197, 199, 209, 219, 222, 254 |
| Rv0481c | *-* | hypothetical protein Rv0481c | 50, 70, 98, 115, 143 |
| Rv0482 | *murB* | UDP-N-acetylenolpyruvoylglucosamine reductase | 3, 10, 57, 88, 101, 118, 130, 166, 214, 220, 272, 287, 295, 296, 303, 314, 331, 334 |
| Rv0483 | *lprQ* | PROBABLE CONSERVED LIPOPROTEIN LPRQ | 49, 74, 106, 131, 187, 203, 279, 319, 320, 347, 383, 427, 439 |
| Rv0484c | *-* | PROBABLE SHORT-CHAIN TYPE OXIDOREDUCTASE | 13, 17, 23, 45, 52, 54, 64, 83, 125, 137, 144, 146, 149, 163, 202, 209 |
| Rv0485 | *-* | POSSIBLE TRANSCRIPTIONAL REGULATORY PROTEIN | 14, 36, 89, 142, 144, 180, 217, 261, 275, 277, 300, 308, 311, 313, 324, 400, 408, 431 |
| Rv0486 | *-* | MANNOSYLTRANSFERASE | 19, 27, 30, 61, 64, 65, 92, 104, 132, 133, 175, 180, 239, 256, 286, 290, 297, 298, 303, 364, 370, 374, 385, 390, 409, 424, 455, 475 |
| Rv0487 | *-* | hypothetical protein Rv0487 | 29, 33, 108, 152, 181 |
| Rv0488 | *-* | PROBABLE CONSERVED INTEGRAL MEMBRANE PROTEIN | 31, 40, 44, 63, 75, 83, 136, 140 |
| Rv0489 | *gpm1* | phosphoglyceromutase | 23, 41, 66, 144, 208, 218, 225, 236, 239, 243, 247 |
| Rv0490 | *senX3* | PUTATIVE TWO COMPONENT SENSOR HISTIDINE KINASE SENX3 | 9, 13, 62, 96, 121, 274, 289, 300, 338, 341, 367 |
| Rv0491 | *regX3* | TWO COMPONENT SENSORY TRANSDUCTION PROTEIN REGX3 (TRANSCRIPTIONAL REGULATORY PROTEIN) (PROBABLY LUXR-FAMILY) | 78, 118, 132, 168, 174, 218 |
| Rv0492c | *-* | PROBABLE OXIDOREDUCTASE GMC-TYPE | 17, 25, 53, 66, 86, 153, 156, 167, 195, 201, 228, 287, 334, 351, 377, 378, 390, 411, 422, 456, 491, 521, 522, 529, 532, 558, 565, 567, 568, 584 |
| Rv0492A | *-* | hypothetical protein Rv0492A | 12, 27, 30, 48, 69, 87 |
| Rv0493c | *-* | hypothetical protein Rv0493c | 11, 26, 29, 46, 59, 60, 86, 89, 91, 93, 99, 109, 124, 142, 151, 171, 203, 255, 298, 307, 317 |
| Rv0494 | *-* | PROBABLE TRANSCRIPTIONAL REGULATORY PROTEIN (PROBABLY GNTR-FAMILY) | 77, 90, 124, 148, 183, 190, 212, 217, 219 |
| Rv0495c | *-* | hypothetical protein Rv0495c | 3, 18, 85, 166, 169, 172, 226, 240, 244 |
| Rv0496 | *-* | hypothetical protein Rv0496 | 5, 34, 83, 120, 144, 183, 203, 210, 254, 294 |
| Rv0497 | *-* | PROBABLE CONSERVED TRANSMEMBRANE PROTEIN | 10, 27, 38, 50, 63, 125, 145, 150, 191, 196, 201, 228, 244 |
| Rv0498 | *-* | hypothetical protein Rv0498 | 20, 89, 119, 145, 187, 214, 217, 224, 226, 231, 239, 241 |
| Rv0499 | *-* | hypothetical protein Rv0499 | 17, 41, 51, 54, 59, 125, 130, 136, 162, 167, 187, 203, 215, 229, 238, 245 |
| Rv0500 | *proC* | pyrroline-5-carboxylate reductase | 11, 161, 175, 178, 195, 210, 244, 250, 256, 259, 268, 281 |
| Rv0500A | *-* | hypothetical protein Rv0500A | 11, 50 |
| Rv0500B | *-* | hypothetical protein Rv0500B |  |
| Rv0501 | *galE2* | POSSIBLE UDP-GLUCOSE 4-EPIMERASE GALE2 (GALACTOWALDENASE) (UDP-GALACTOSE 4-EPIMERASE) (URIDINE DIPHOSPHATE GALACTOSE 4-EPIMERASE) (URIDINE DIPHOSPHO-GALACTOSE 4-EPIMERASE) | 6, 9, 13, 28, 35, 39, 97, 106, 107, 125, 214, 246, 249, 269, 336, 338, 372 |
| Rv0502 | *-* | hypothetical protein Rv0502 | 27, 53, 85, 96, 178, 191, 211, 215, 240, 246, 315, 349 |
| Rv0503c | *cmaA2* | CYCLOPROPANE-FATTY-ACYL-PHOSPHOLIPID SYNTHASE 2 CMAA2 (CYCLOPROPANE FATTY ACID SYNTHASE) (CFA SYNTHASE) (CYCLOPROPANE MYCOLIC ACID SYNTHASE 2) (MYCOLIC ACID TRANS-CYCLOPROPANE SYNTHETASE) | 8, 71, 80, 153, 156, 216, 232 |
| Rv0504c | *-* | hypothetical protein Rv0504c | 47, 50, 53 |
| Rv0505c | *serB1* | POSSIBLE PHOSPHOSERINE PHOSPHATASE SERB1 (PSP) (O-PHOSPHOSERINE PHOSPHOHYDROLASE) (PSPASE) | 8, 19, 26, 51, 56, 77, 107, 109, 120, 122, 142, 145, 177, 225, 233, 250, 265, 276, 297, 314, 344, 358, 359 |
| Rv0506 | *mmpS2* | PROBABLE CONSERVED MEMBRANE PROTEIN MMPS2 | 6, 67, 70, 101, 145 |
| Rv0507 | *mmpL2* | PROBABLE CONSERVED TRANSMEMBRANE TRANSPORT PROTEIN MMPL2 | 5, 33, 59, 107, 136, 176, 188, 197, 217, 233, 280, 295, 307, 410, 464, 515, 661, 670, 714, 749, 767, 788, 852, 856, 865, 872, 956 |
| Rv0508 | *-* | hypothetical protein Rv0508 | 11 |
| Rv0509 | *hemA* | glutamyl-tRNA reductase | 63, 76, 104, 155, 160, 183, 189, 203, 271, 319, 326, 332, 358, 374, 410, 411, 428, 444, 452 |
| Rv0510 | *hemC* | porphobilinogen deaminase | 15, 28, 79, 124, 152, 185, 198, 220, 234, 260, 275, 280, 291 |
| Rv0511 | *hemD* | PROBABLE UROPORPHYRIN-III C-METHYLTRANSFERASE HEMD (UROPORPHYRINOGEN III METHYLASE) (UROGEN III METHYLASE) (SUMT) (UROGEN III METHYLASE) (UROM) | 16, 21, 29, 35, 60, 64, 75, 76, 86, 88, 92, 104, 112, 153, 156, 165, 192, 194, 202, 227, 256, 259, 267, 306, 334, 378, 405, 457, 478, 498, 514, 517, 531, 545 |
| Rv0512 | *hemB* | delta-aminolevulinic acid dehydratase | 66, 69, 72, 73, 135, 179, 187, 188, 199, 219, 232, 254, 264, 274, 298, 305, 310 |
| Rv0513 | *-* | POSSIBLE CONSERVED TRANSMEMBRANE PROTEIN | 3, 15, 39, 41, 104, 125, 135, 139, 177 |
| Rv0514 | *-* | POSSIBLE TRANSMEMBRANE PROTEIN | 6, 15, 18, 63, 93, 94 |
| Rv0515 | *-* | CONSERVED 13E12 REPEAT FAMILY PROTEIN | 41, 60, 75, 112, 144, 242, 258, 296, 315, 344, 349, 362, 404, 425, 471, 482 |
| Rv0516c | *-* | hypothetical protein Rv0516c | 17, 20, 48, 81, 95, 96, 106 |
| Rv0517 | *-* | POSSIBLE MEMBRANE ACYLTRANSFERASE | 1, 8, 32, 75, 130, 152, 230, 248, 255, 270, 294, 299, 333, 349, 405, 412 |
| Rv0518 | *-* | POSSIBLE EXPORTED PROTEIN | 48, 53, 61, 80, 85, 90, 101, 148, 160 |
| Rv0519c | *-* | POSSIBLE CONSERVED MEMBRANE PROTEIN | 12, 31, 33, 37, 38, 73, 81, 94, 97, 113, 143, 153, 154, 203, 218, 222, 240, 251, 273, 278, 291 |
| Rv0520 | *-* | POSSIBLE METHYLTRANSFERASE/METHYLASE (FRAGMENT) | 25, 46, 76, 77 |
| Rv0521 | *-* | POSSIBLE METHYLTRANSFERASE/METHYLASE (FRAGMENT) | 3, 61, 88 |
| Rv0522 | *gabP* | PROBABLE GABA PERMEASE GABP (4-AMINO BUTYRATE TRANSPORT CARRIER) (GAMA-AMINOBUTYRATE PERMEASE) | 4, 22, 33, 48, 66, 91, 92, 116, 134, 149, 200, 251, 271, 283, 292, 332, 386, 400 |
| Rv0523c | *-* | hypothetical protein Rv0523c | 22, 76, 78, 79 |
| Rv0524 | *hemL* | glutamate-1-semialdehyde aminotransferase | 13, 23, 29, 30, 43, 91, 94, 103, 109, 127, 140, 143, 152, 197, 202, 230, 238, 246, 248, 258, 272, 288, 299, 303, 305, 308, 326, 337, 338, 345, 352, 362, 399, 418, 434, 449, 460 |
| Rv0525 | *-* | hypothetical protein Rv0525 | 18, 26, 31, 33, 38, 64, 69, 96, 131, 139, 141, 162 |
| Rv0526 | *-* | POSSIBLE THIOREDOXIN PROTEIN (THIOL-DISULFIDE INTERCHANGE PROTEIN) | 8, 19, 28, 31, 39, 47, 48 |
| Rv0527 | *ccdA* | POSSIBLE CYTOCHROME C-TYPE BIOGENESIS PROTEIN CCDA | 1, 7, 37, 55, 69, 163, 223, 232, 236 |
| Rv0528 | *-* | PROBABLE CONSERVED TRANSMEMBRANE PROTEIN | 9, 23, 34, 42, 66, 97, 114, 117, 121, 129, 145, 188, 190, 198, 205, 231, 238, 271, 322, 358, 371, 374, 378, 407, 419, 440, 475, 484, 492, 502, 520, 523 |
| Rv0529 | *ccsA* | POSSIBLE CYTOCHROME C-TYPE BIOGENESIS PROTEIN CCSA | 39, 41, 50, 78, 97, 119, 123, 151, 159, 188, 206, 291, 297 |
| Rv0530 | *-* | hypothetical protein Rv0530 | 15, 18, 40, 67, 126, 132, 147, 167, 178, 191, 223, 231, 271, 273, 304, 389, 393 |
| Rv0531 | *-* | POSSIBLE CONSERVED MEMBRANE PROTEIN | 75, 80 |
| Rv0532 | *PE_PGRS6* | PE-PGRS FAMILY PROTEIN | 31, 40, 41, 80, 91, 123, 125, 129, 132, 139, 142, 147, 151, 153, 154, 156, 157, 163, 165, 166, 170, 175, 176, 179, 182, 185, 191, 193, 202, 205, 206, 208, 211, 219, 222, 228, 232, 234, 245, 256, 259, 262, 265, 274, 283, 296, 297, 299, 300, 302, 319, 328, 331, 333, 345, 348, 349, 352, 359, 361, 362, 365, 368, 376, 379, 381, 382, 384, 386, 395, 398, 400, 402, 404, 410, 412, 414, 423, 435, 436, 439, 441, 444, 447, 455, 458, 469, 471, 472, 474, 475, 480, 483, 490, 493, 495, 505, 508, 510, 511, 513, 514, 516, 517, 569, 574, 585, 590 |
| Rv0533c | *fabH* | 3-oxoacyl-(acyl carrier protein) synthase III | 7, 46, 54, 75, 99, 122, 137, 160, 171, 212, 223, 243, 244, 286, 304, 309, 320, 325 |
| Rv0534c | *menA* | 1,4-dihydroxy-2-naphthoate octaprenyltransferase | 8, 24, 26, 28, 65, 90, 102, 123, 129, 130, 223, 243, 251, 256, 258 |
| Rv0535 | *pnp* | 5'-methylthioadenosine phosphorylase | 11, 13, 98, 122, 126, 141, 144, 149, 170, 172, 180, 206, 216, 221, 236, 254 |
| Rv0536 | *galE3* | PROBABLE UDP-GLUCOSE 4-EPIMERASE GALE3 (GALACTOWALDENASE) (UDP-GALACTOSE 4-EPIMERASE) (URIDINE DIPHOSPHATE GALACTOSE 4-EPIMERASE) (URIDINE DIPHOSPHO-GALACTOSE 4-EPIMERASE) | 5, 8, 34, 63, 72, 77, 81, 85, 88, 104, 105, 154, 180, 194, 208, 217, 220, 239, 252, 257, 270, 276, 287, 293, 302, 307, 317, 320 |
| Rv0537c | *-* | PROBABLE INTEGRAL MEMBRANE PROTEIN | 17, 65, 98, 104, 116, 127, 158, 172, 174, 177, 199, 229, 255, 266, 268, 342, 359, 367, 393, 417 |
| Rv0538 | *-* | POSSIBLE CONSERVED MEMBRANE PROTEIN | 23, 54, 83, 96, 106, 170, 207, 209, 265, 305, 343, 348, 365, 382, 412, 490, 532, 538, 545 |
| Rv0539 | *-* | PROBABLE DOLICHYL-PHOSPHATE SUGAR SYNTHASE (DOLICHOL-PHOSPHATE SUGAR SYNTHETASE) (DOLICHOL-PHOSPHATE SUGAR TRANSFERASE) (SUGAR PHOSPHORYLDOLICHOL SYNTHASE) | 15, 19, 34, 38, 51, 59, 63, 80, 98, 166, 184, 185, 191 |
| Rv0540 | *-* | hypothetical protein Rv0540 | 24, 32, 36, 48, 53, 60, 65, 69, 85, 117, 132, 141, 167 |
| Rv0541c | *-* | PROBABLE CONSERVED INTEGRAL MEMBRANE PROTEIN | 19, 45, 47, 68, 95, 97, 111, 114, 128, 157, 176, 178, 234, 260, 288, 316, 371, 377, 388, 415, 418, 434, 435 |
| Rv0542c | *menE* | O-succinylbenzoic acid--CoA ligase | 51, 61, 67, 82, 93, 103, 129, 143, 154, 165, 167, 169, 184, 202, 203, 209, 261, 272, 299, 326, 341, 356 |
| Rv0543c | *-* | hypothetical protein Rv0543c | 83 |
| Rv0544c | *-* | POSSIBLE CONSERVED TRANSMEMBRANE PROTEIN | 19, 35, 36, 43, 81 |
| Rv0545c | *pitA* | PROBABLE LOW-AFFINITY INORGANIC PHOSPHATE TRANSPORTER INTEGRAL MEMBRANE PROTEIN PITA | 26, 36, 60, 83, 87, 110, 118, 128, 141, 161, 170, 174, 229, 230, 257, 263, 269, 283, 289, 294, 303, 317, 335, 347, 349, 375 |
| Rv0546c | *-* | hypothetical protein Rv0546c | 35, 41, 49, 62 |
| Rv0547c | *-* | short chain dehydrogenase | 15, 30, 38, 45, 54, 84, 87, 115, 124, 158, 162, 196, 242, 246, 252 |
| Rv0548c | *menB* | naphthoate synthase | 4, 87, 96, 102, 103, 112, 118, 121, 130, 158, 159, 192, 219, 278 |
| Rv0549c | *-* | hypothetical protein Rv0549c | 39, 61 |
| Rv0550c | *-* | hypothetical protein Rv0550c | 44, 53, 67, 80 |
| Rv0551c | *fadD8* | acyl-CoA synthetase | 2, 3, 12, 14, 30, 74, 94, 96, 116, 120, 125, 143, 191, 194, 204, 218, 219, 222, 231, 263, 275, 410, 416, 460, 461, 498, 512, 518, 547 |
| Rv0552 | *-* | hypothetical protein Rv0552 | 8, 41, 55, 96, 99, 105, 108, 121, 146, 156, 166, 185, 197, 219, 299, 308, 311, 343, 382, 412, 421, 424, 425, 449, 479, 526 |
| Rv0553 | *menC* | O-succinylbenzoate synthase | 26, 40, 53, 66, 89, 149, 152, 155, 185, 204, 216, 240, 245, 246, 249, 251, 261, 265, 266, 276 |
| Rv0554 | *bpoC* | POSSIBLE PEROXIDASE BPOC (NON-HAEM PEROXIDASE) | 10, 18, 20, 23, 68, 112, 177, 193, 199, 236, 250 |
| Rv0555 | *menD* | 2-succinyl-6-hydroxy-2,4-cyclohexadiene-1-carboxylic acid synthase/2-oxoglutarate decarboxylase | 16, 43, 56, 57, 78, 80, 113, 162, 168, 229, 253, 299, 313, 320, 325, 353, 367, 385, 390, 401, 409, 424, 447, 470, 526, 528, 547 |
| Rv0556 | *-* | PROBABLE CONSERVED TRANSMEMBRANE PROTEIN | 38, 73, 127, 134, 145, 147 |
| Rv0557 | *pimB* | MANNOSYLTRANSFERASE PIMB | 33, 88, 110, 180, 229, 256, 277, 302, 303, 314, 344, 376 |
| Rv0558 | *ubiE* | ubiquinone/menaquinone biosynthesis methyltransferase | 3, 50, 58, 61, 97, 139, 157, 204, 215, 216, 223, 227 |
| Rv0559c | *-* | POSSIBLE CONSERVED SECRETED PROTEIN | 79, 92 |
| Rv0560c | *-* | POSSIBLE BENZOQUINONE METHYLTRANSFERASE (METHYLASE) | 26, 29, 53, 62, 70, 73, 93, 106, 122, 200, 209, 212, 215 |
| Rv0561c | *-* | POSSIBLE OXIDOREDUCTASE | 13, 16, 21, 36, 45, 78, 89, 116, 210, 259, 280, 290, 308, 312, 335, 364, 391, 393 |
| Rv0562 | *grcC1* | PROBABLE POLYPRENYL-DIPHOSPHATE SYNTHASE GRCC1 (POLYPRENYL PYROPHOSPHATE SYNTHETASE) | 18, 53, 54, 75, 81, 106, 130, 155, 162, 182, 189, 197, 211, 216, 238 |
| Rv0563 | *htpX* | heat shock protein HtpX | 19, 96, 104, 111, 158, 167, 171, 216, 230, 231, 284 |
| Rv0564c | *gpsA* | NAD(P)H-dependent glycerol-3-phosphate dehydrogenase | 13, 28, 40, 73, 90, 130, 138, 152, 240, 243, 289, 331 |
| Rv0565c | *-* | PROBABLE MONOOXYGENASE | 6, 16, 19, 46, 55, 142, 162, 178, 187, 190, 225, 270, 310, 326, 332, 343, 345, 353, 355, 413, 420, 439, 452 |
| Rv0566c | *-* | nucleotide-binding protein | 23, 34, 60, 81, 94 |
| Rv0567 | *-* | PROBABLE METHYLTRANSFERASE/METHYLASE | 11, 22, 38, 73, 96, 119, 129, 203, 226, 287, 306, 328, 329 |
| Rv0568 | *cyp135B1* | POSSIBLE CYTOCHROME P450 135B1 CYP135B1 | 1, 15, 59, 66, 74, 82, 118, 125, 130, 166, 225, 237, 265, 295, 299, 332, 338, 381, 416, 431, 454, 458, 460, 462 |
| Rv0569 | *-* | hypothetical protein Rv0569 |  |
| Rv0570 | *nrdZ* | PROBABLE RIBONUCLEOSIDE-DIPHOSPHATE REDUCTASE (LARGE SUBUNIT) NRDZ (RIBONUCLEOTIDE REDUCTASE) | 55, 74, 133, 190, 211, 219, 221, 230, 231, 239, 243, 255, 262, 285, 324, 332, 343, 346, 350, 376, 412, 455, 527, 530, 579, 593, 597, 611, 619, 628, 667, 681 |
| Rv0571c | *-* | hypothetical protein Rv0571c | 21, 32, 84, 91, 113, 121, 134, 169, 221, 236, 278, 280, 315, 324, 345, 358, 399, 408, 436, 441 |
| Rv0572c | *-* | hypothetical protein Rv0572c | 42, 71 |
| Rv0573c | *-* | nicotinate phosphoribosyltransferase | 49, 50, 87, 92, 97, 109, 141, 146, 149, 212, 235, 260, 276, 277, 296, 321, 335, 363, 375, 421, 450 |
| Rv0574c | *-* | hypothetical protein Rv0574c | 12, 28, 42, 75, 89, 143, 158, 183, 196, 223, 241, 243, 255, 275, 283, 294, 313, 315, 371 |
| Rv0575c | *-* | hypothetical protein Rv0575c | 5, 7, 21, 36, 37, 76, 101, 105, 141, 210, 227, 251, 274, 284, 291, 295, 306, 312, 373 |
| Rv0576 | *-* | PROBABLE TRANSCRIPTIONAL REGULATORY PROTEIN (POSSIBLY ARSR-FAMILY) | 51, 66, 90, 134, 141, 156, 164, 183, 196, 208, 212, 229, 232, 235, 265, 369, 387, 398, 402, 421, 426, 431 |
| Rv0577 | *TB27.3* | hypothetical protein Rv0577 | 22, 42, 44, 59, 94, 96, 109, 119, 184, 190, 198, 220, 224, 256 |
| Rv0578c | *PE_PGRS7* | PE-PGRS FAMILY PROTEIN | 29, 32, 39, 79, 81, 87, 99, 122, 124, 128, 131, 138, 141, 150, 152, 153, 162, 167, 168, 173, 174, 176, 180, 183, 192, 196, 198, 202, 205, 207, 208, 210, 211, 217, 219, 222, 228, 241, 243, 245, 247, 248, 251, 254, 256, 257, 259, 260, 267, 270, 272, 273, 276, 278, 279, 281, 282, 287, 300, 301, 304, 310, 312, 313, 316, 318, 319, 328, 333, 334, 337, 339, 343, 346, 349, 354, 363, 364, 366, 370, 375, 378, 381, 387, 397, 403, 407, 410, 419, 422, 432, 438, 440, 443, 446, 449, 450, 459, 462, 463, 465, 466, 468, 469, 471, 472, 475, 479, 482, 484, 488, 490, 491, 494, 496, 507, 509, 511, 515, 516, 519, 525, 527, 528, 531, 533, 534, 539, 550, 553, 555, 556, 559, 565, 569, 570, 582, 586, 589, 592, 594, 595, 598, 604, 610, 616, 620, 623, 630, 635, 638, 639, 642, 645, 651, 653, 654, 656, 657, 665, 673, 677, 679, 680, 685, 688, 689, 698, 699, 701, 702, 704, 705, 708, 713, 729, 730, 732, 733, 736, 738, 739, 748, 750, 756, 761, 762, 764, 765, 770, 771, 774, 777, 779, 788, 792, 796, 798, 799, 801, 802, 805, 808, 810, 814, 816, 822, 827, 828, 831, 833, 836, 837, 840, 843, 858, 861, 864, 867, 870, 872, 873, 876, 878, 885, 895, 898, 901, 909, 914, 915, 918, 921, 927, 929, 932, 933, 936, 939, 947, 950, 952, 955, 956, 959, 965, 968, 971, 974, 976, 986, 988, 991, 995, 998, 1005, 1011, 1014, 1016, 1017, 1020, 1028, 1031, 1034, 1045, 1050, 1051, 1054, 1056, 1057, 1059, 1060, 1062, 1066, 1068, 1069, 1084, 1085, 1090, 1093, 1094, 1096, 1097, 1099, 1100, 1103, 1119, 1122, 1125, 1127, 1128, 1130, 1131, 1133, 1136, 1137, 1139, 1140, 1142, 1149, 1155, 1158, 1164, 1167, 1171, 1175, 1176, 1178, 1179, 1182, 1184, 1185, 1192, 1193, 1196, 1199, 1202, 1210, 1214, 1217, 1226, 1230, 1236, 1242, 1243, 1252, 1257, 1260, 1261, 1263, 1275, 1278, 1284, 1289, 1292, 1296, 1298, 1301, 1302 |
| Rv0579 | *-* | hypothetical protein Rv0579 | 21, 58, 67, 72, 85, 123, 145, 195, 223 |
| Rv0580c | *-* | hypothetical protein Rv0580c | 33, 46, 73, 83, 94, 114 |
| Rv0581 | *-* | hypothetical protein Rv0581 | 18, 22, 46 |
| Rv0582 | *-* | hypothetical protein Rv0582 | 13, 19, 28, 63, 64, 72, 74, 80, 126 |
| Rv0583c | *lpqN* | PROBABLE CONSERVED LIPOPROTEIN LPQN | 79, 104, 111, 128, 133, 141, 148, 153, 162, 215 |
| Rv0584 | *-* | POSSIBLE CONSERVED EXPORTED PROTEIN | 10, 50, 54, 64, 84, 94, 141, 149, 151, 158, 183, 192, 204, 209, 213, 244, 254, 262, 270, 288, 305, 410, 412, 416, 439, 451, 465, 486, 511, 514, 532, 541, 548, 579, 583, 613, 617, 642, 650, 698, 705, 710, 740, 749, 758, 762, 790, 824, 858 |
| Rv0585c | *-* | PROBABLE CONSERVED INTEGRAL MEMBRANE PROTEIN | 10, 103, 104, 161, 227, 232, 240, 242, 249, 256, 273, 287, 291, 339, 341, 389, 422, 425, 446, 489, 521, 574, 577, 592, 597, 622, 642, 695, 713, 728, 734, 739, 753, 780 |
| Rv0586 | *-* | PROBABLE TRANSCRIPTIONAL REGULATORY PROTEIN (GNTR-FAMILY) | 24, 29, 48, 59, 161, 189, 211, 216 |
| Rv0587 | *yrbE2A* | CONSERVED HYPOTHETICAL INTEGRAL MEMBRANE PROTEIN YRBE2A | 84, 88, 90, 110, 151, 156, 183, 194, 220, 233 |
| Rv0588 | *yrbE2B* | CONSERVED HYPOTHETICAL INTEGRAL MEMBRANE PROTEIN YRBE2B | 20, 27, 73, 80, 92, 93, 114, 118, 131, 136, 141, 144, 249, 251 |
| Rv0589 | *mce2A* | MCE-FAMILY PROTEIN MCE2A | 122, 171, 181, 249, 260, 268, 276, 282, 312, 320, 321, 346, 370 |
| Rv0590 | *mce2B* | MCE-FAMILY PROTEIN MCE2B | 36, 39, 46, 50, 93, 111, 114, 140, 179, 236, 245 |
| Rv0590A | *-* | MCE-FAMILY RELATED PROTEIN | 40, 77 |
| Rv0591 | *mce2C* | MCE-FAMILY PROTEIN MCE2C | 8, 73, 84, 112, 167, 188, 225, 290, 299 |
| Rv0592 | *mce2D* | MCE-FAMILY PROTEIN MCE2D | 22, 32, 35, 80, 101, 122, 123, 176, 181, 199, 300, 345, 354, 402, 432, 443, 471, 491 |
| Rv0593 | *lprL* | POSSIBLE MCE-FAMILY LIPOPROTEIN LPRL (MCE-FAMILY LIPOPROTEIN MCE2E) | 2, 20, 121, 138, 150, 177, 178, 199, 213, 220, 261, 320, 333, 340, 356, 393 |
| Rv0594 | *mce2F* | MCE-FAMILY PROTEIN MCE2F | 48, 68, 116, 141, 166, 210, 218, 225, 228, 291, 303, 306, 311, 323, 339, 370, 388, 405, 408, 434, 483, 502, 514 |
| Rv0595c | *-* | hypothetical protein Rv0595c | 48, 52, 88, 119 |
| Rv0596c | *-* | hypothetical protein Rv0596c | 5, 13, 21, 22 |
| Rv0597c | *-* | hypothetical protein Rv0597c | 13, 57, 137, 149, 171, 221, 224, 244, 292, 299, 334, 351, 387, 409 |
| Rv0598c | *-* | hypothetical protein Rv0598c | 20, 34, 37, 62, 106, 135 |
| Rv0599c | *-* | hypothetical protein Rv0599c | 42, 58, 74 |
| Rv0600c | *-* | PROBABLE TWO COMPONENT SENSOR KINASE -SECOND PART | 14, 42, 59, 76, 95, 97, 100, 110, 117, 128 |
| Rv0601c | *-* | PROBABLE TWO COMPONENT SENSOR KINASE -FIRST PART | 7, 18, 29, 36, 54, 57, 64, 148 |
| Rv0602c | *tcrA* | PROBABLE TWO COMPONENT DNA BINDING TRANSCRIPTIONAL REGULATORY PROTEIN TCRA | 10, 16, 63, 76, 100, 102, 114, 139, 150, 186, 202, 235, 237 |
| Rv0603 | *-* | POSSIBLE EXPORTED PROTEIN | 9, 12, 20, 35, 44, 52, 54, 55, 62, 66, 101 |
| Rv0604 | *lpqO* | PROBABLE CONSERVED LIPOPROTEIN LPQO | 9, 15, 20, 34, 59, 88, 128, 129, 134, 153, 158, 177, 182, 191, 201, 206, 222, 234, 241, 255, 267, 269, 307, 312 |
| Rv0605 | *-* | POSSIBLE RESOLVASE | 23, 143, 193 |
| Rv0606 | *-* | POSSIBLE TRANSPOSASE (FRAGMENT) | 21, 56, 233 |
| Rv0607 | *-* | hypothetical protein Rv0607 | 20, 50, 88, 100 |
| Rv0608 | *-* | hypothetical protein Rv0608 | 20, 30, 45, 55 |
| Rv0609 | *-* | hypothetical protein Rv0609 | 17, 40, 89, 92 |
| Rv0609A | *-* | hypothetical protein Rv0609A | 49, 64 |
| Rv0610c | *-* | hypothetical protein Rv0610c | 13, 17, 65, 118, 140, 155, 170, 179, 211, 216, 228, 311, 328, 369 |
| Rv0611c | *-* | hypothetical protein Rv0611c | 23, 51, 74, 85, 94, 107 |
| Rv0612 | *-* | hypothetical protein Rv0612 | 21, 55, 57, 71, 107, 128, 142, 145, 191 |
| Rv0613c | *-* | hypothetical protein Rv0613c | 32, 68, 118, 137, 138, 153, 164, 176, 195, 199, 239, 299, 302, 306, 340, 341, 344, 410, 415, 423, 454, 493, 516, 532, 542, 583, 600, 610, 611, 619, 679, 763, 773, 786, 806, 816, 834, 835, 841, 850 |
| Rv0614 | *-* | hypothetical protein Rv0614 | 58, 101, 106, 108, 123, 145, 148, 149, 185, 219, 226, 242, 252, 281, 297, 300, 307, 309 |
| Rv0615 | *-* | PROBABLE INTEGRAL MEMBRANE PROTEIN | 33, 47, 63 |
| Rv0616c | *-* | hypothetical protein Rv0616c |  |
| Rv0617 | *-* | hypothetical protein Rv0617 | 22, 32, 54, 81, 115 |
| Rv0618 | *galTa* | PROBABLE GALACTOSE-1-PHOSPHATE URIDYLYLTRANSFERASE GALTA | 6, 19, 54, 83, 90, 102, 112, 119, 136, 138, 153, 170, 192, 199 |
| Rv0619 | *galTb* | PROBABLE GALACTOSE-1-PHOSPHATE URIDYLYLTRANSFERASE GALTB | 22, 58, 63, 69, 154 |
| Rv0620 | *galK* | galactokinase | 7, 42, 46, 68, 75, 84, 89, 90, 121, 123, 155, 163, 200, 201, 215, 229, 261, 269, 271, 276, 303, 311, 313, 341, 342, 354, 356 |
| Rv0621 | *-* | POSSIBLE MEMBRANE PROTEIN | 8, 46, 67, 108, 112, 124, 149, 156, 157, 179, 193, 196, 205, 216, 233, 236, 246, 248, 260, 296, 332, 347 |
| Rv0622 | *-* | POSSIBLE MEMBRANE PROTEIN | 6, 16, 19, 38, 50, 60, 123, 144, 149, 152, 164, 230, 236, 267 |
| Rv0623 | *-* | hypothetical protein Rv0623 | 20, 30, 53, 69, 79 |
| Rv0624 | *-* | hypothetical protein Rv0624 | 5, 22, 52, 77, 80, 108 |
| Rv0625c | *-* | PROBABLE CONSERVED TRANSMEMBRANE PROTEIN | 6, 22, 33, 41, 50, 76, 79, 99, 110, 120, 134, 146, 175, 183, 207 |
| Rv0626 | *-* | hypothetical protein Rv0626 | 13, 58, 64, 74 |
| Rv0627 | *-* | hypothetical protein Rv0627 | 5, 6, 19, 48, 77, 91, 121 |
| Rv0628c | *-* | hypothetical protein Rv0628c | 8, 15, 20, 61, 73, 82, 90, 93, 104, 111, 118, 152, 156, 175, 183, 205, 216, 241, 264, 314, 328, 348, 353, 357, 358, 374 |
| Rv0629c | *recD* | PROBABLE EXONUCLEASE V (ALPHA CHAIN) RECD (EXODEOXYRIBONUCLEASE V ALPHA CHAIN) (EXODEOXYRIBONUCLEASE V POLYPEPTIDE) | 12, 21, 39, 59, 136, 147, 169, 173, 193, 203, 206, 305, 346, 416, 433, 443, 463, 468, 471, 481, 488, 532, 552, 562 |
| Rv0630c | *recB* | PROBABLE EXONUCLEASE V (BETA CHAIN) RECB (EXODEOXYRIBONUCLEASE V BETA CHAIN)(EXODEOXYRIBONUCLEASE V POLYPEPTIDE) (CHI-SPECIFIC ENDONUCLEASE) | 22, 24, 31, 41, 66, 83, 92, 124, 134, 138, 199, 210, 298, 309, 311, 349, 356, 384, 401, 410, 415, 451, 461, 478, 483, 493, 522, 525, 530, 534, 544, 572, 586, 599, 647, 651, 698, 705, 709, 736, 738, 760, 798, 799, 811, 813, 815, 824, 842, 846, 892, 894, 918, 923, 943, 958, 980, 997, 1044, 1055, 1063, 1067 |
| Rv0631c | *recC* | PROBABLE EXONUCLEASE V (GAMMA CHAIN) RECC (EXODEOXYRIBONUCLEASE V GAMMA CHAIN)(EXODEOXYRIBONUCLEASE V POLYPEPTIDE) | 39, 65, 80, 131, 142, 153, 157, 168, 205, 253, 272, 293, 320, 326, 327, 388, 396, 401, 424, 436, 446, 503, 517, 532, 547, 550, 570, 592, 614, 624, 700, 706, 712, 751, 764, 768, 772, 774, 785, 789, 792, 853, 860, 867, 872, 892, 918, 926, 953, 999, 1028, 1046, 1072, 1082, 1091 |
| Rv0632c | *echA3* | enoyl-CoA hydratase | 51, 58, 68, 73, 80, 100, 113, 157, 167, 170, 171, 190, 195, 203, 219 |
| Rv0633c | *-* | POSSIBLE EXPORTED PROTEIN | 15, 19, 26, 85, 105, 126, 172, 242 |
| Rv0634c | *-* | POSSIBLE GLYOXALASE II (HYDROXYACYLGLUTATHIONE HYDROLASE) (GLX II) | 12, 38, 50, 78, 143, 160, 175, 176, 200, 224, 235 |
| Rv0634A | *-* | hypothetical protein Rv0634A | 6, 55 |
| Rv0634B | *rpmG* | 50S ribosomal protein L33 | 44 |
| Rv0635 | *-* | hypothetical protein Rv0635 | 44, 78, 145 |
| Rv0636 | *-* | hypothetical protein Rv0636 | 30, 53, 65, 122, 125, 126 |
| Rv0637 | *-* | hypothetical protein Rv0637 | 7, 44, 98, 149, 158, 164 |
| Rv0638 | *secE* | preprotein translocase subunit SecE | 21, 23, 41, 63, 69, 145 |
| Rv0639 | *nusG* | transcription antitermination protein NusG | 8, 34, 45, 105, 120, 168, 173 |
| Rv0640 | *rplK* | 50S ribosomal protein L11 | 7, 27, 45, 51, 75, 83, 130, 132 |
| Rv0641 | *rplA* | 50S ribosomal protein L1 | 9, 11, 59, 88, 105, 112, 133, 141, 145, 157 |
| Rv0642c | *mmaA4* | METHOXY MYCOLIC ACID SYNTHASE 4 MMAA4 (METHYL MYCOLIC ACID SYNTHASE 4) (MMA4) (HYDROXY MYCOLIC ACID SYNTHASE) | 81, 167, 188, 209, 210, 226 |
| Rv0643c | *mmaA3* | METHOXY MYCOLIC ACID SYNTHASE 3 MMAA3 (METHYL MYCOLIC ACID SYNTHASE 3) (MMA3) (HYDROXY MYCOLIC ACID SYNTHASE) | 78, 164, 175, 185, 207, 223, 260, 272 |
| Rv0644c | *mmaA2* | METHOXY MYCOLIC ACID SYNTHASE 2 MMAA2 (METHYL MYCOLIC ACID SYNTHASE 2) (MMA2) (HYDROXY MYCOLIC ACID SYNTHASE) | 63, 72, 99, 169, 266 |
| Rv0645c | *mmaA1* | METHOXY MYCOLIC ACID SYNTHASE 1 MMAA1 (METHYL MYCOLIC ACID SYNTHASE 1) (MMA1) (HYDROXY MYCOLIC ACID SYNTHASE) | 71, 74, 106, 200, 215, 238, 265 |
| Rv0646c | *lipG* | PROBABLE LIPASE/ESTERASE LIPG | 4, 6, 9, 101, 104, 124, 132, 205, 228, 257, 299 |
| Rv0647c | *-* | hypothetical protein Rv0647c | 66, 73, 129, 137, 182, 200, 292, 298, 316, 374, 375, 394, 467 |
| Rv0648 | *-* | ALPHA-MANNOSIDASE | 2, 39, 56, 59, 68, 79, 98, 126, 160, 185, 210, 225, 240, 244, 270, 283, 304, 308, 338, 351, 380, 391, 409, 424, 425, 471, 513, 528, 557, 582, 602, 619, 623, 662, 667, 677, 686, 696, 721, 741, 773, 780, 784, 794, 816, 818, 824, 872, 878, 886, 952, 972, 991, 1005, 1008, 1017, 1028, 1052, 1065, 1066, 1088, 1124, 1125, 1162, 1169, 1188 |
| Rv0649 | *fabD2* | POSSIBLE MALONYL COA-ACYL CARRIER PROTEIN TRANSACYLASE FABD2 (MCT) | 7, 28, 51, 56, 79, 82, 88, 93, 112, 126, 129, 151, 155, 166, 206, 211 |
| Rv0650 | *-* | POSSIBLE SUGAR KINASE | 8, 13, 20, 26, 35, 67, 74, 86, 95, 121, 135, 138, 152, 155, 172, 183, 197, 200, 202, 213, 224, 226, 230, 248, 286, 288, 289 |
| Rv0651 | *rplJ* | 50S ribosomal protein L10 | 7, 20, 28, 63, 76, 82, 88, 106, 136, 147, 162 |
| Rv0652 | *rplL* | 50S ribosomal protein L7/L12 | 34, 36, 42, 44, 46, 49, 51, 69, 85, 121, 122 |
| Rv0653c | *-* | POSSIBLE TRANSCRIPTIONAL REGULATORY PROTEIN (PROBABLY TETR-FAMILY) | 4, 13, 32, 47, 50, 54, 73, 85, 150, 163, 180, 195 |
| Rv0654 | *-* | PROBABLE DIOXYGENASE | 2, 5, 32, 63, 89, 93, 107, 112, 122, 131, 156, 159, 180, 245, 346, 347, 363, 367, 406, 421, 461 |
| Rv0655 | *mkl* | POSSIBLE RIBONUCLEOTIDE-TRANSPORT ATP-BINDING PROTEIN ABC TRANSPORTER MKL | 50, 51, 63, 80, 124, 160, 164, 165, 171, 198, 290, 295 |
| Rv0656c | *-* | hypothetical protein Rv0656c | 6 |
| Rv0657c | *-* | hypothetical protein Rv0657c | 19 |
| Rv0658c | *-* | PROBABLE CONSERVED INTEGRAL MEMBRANE PROTEIN | 38, 45, 53, 59, 60, 70, 85, 96, 109, 118, 154, 205, 206, 222 |
| Rv0659c | *-* | hypothetical protein Rv0659c | 8, 74 |
| Rv0660c | *-* | hypothetical protein Rv0660c | 37, 40 |
| Rv0661c | *-* | hypothetical protein Rv0661c | 39, 59, 84, 106, 109, 112, 135 |
| Rv0662c | *-* | hypothetical protein Rv0662c | 19, 59, 84, 85, 90, 98, 120 |
| Rv0663 | *atsD* | POSSIBLE ARYLSULFATASE ATSD (ARYL-SULFATE SULPHOHYDROLASE) (ARYLSULPHATASE) | 15, 73, 85, 103, 104, 107, 114, 141, 166, 168, 287, 388, 434, 510, 625, 633, 647, 674, 680, 692, 694, 699, 765 |
| Rv0664 | *-* | hypothetical protein Rv0664 | 27, 33, 42, 48, 82 |
| Rv0665 | *-* | hypothetical protein Rv0665 | 20, 89, 106 |
| Rv0666 | *-* | POSSIBLE MEMBRANE PROTEIN | 8 |
| Rv0667 | *rpoB* | DNA-directed RNA polymerase subunit beta | 9, 26, 66, 93, 129, 137, 176, 201, 352, 418, 453, 454, 461, 525, 584, 621, 631, 640, 691, 837, 856, 879, 935, 941, 949, 1008, 1039, 1051, 1164 |
| Rv0668 | *rpoC* | DNA-directed RNA polymerase subunit beta' | 61, 77, 97, 199, 252, 331, 386, 391, 440, 582, 592, 595, 602, 623, 653, 693, 718, 726, 804, 807, 817, 862, 866, 869, 894, 932, 971, 984, 998, 1005, 1024, 1049, 1070, 1196, 1199, 1201, 1259, 1261, 1300 |
| Rv0669c | *-* | POSSIBLE HYDROLASE | 5, 17, 29, 30, 98, 101, 104, 114, 141, 215, 231, 237, 244, 278, 291, 315, 327, 334, 383, 466, 512, 515, 518, 553, 589, 600, 605, 615 |
| Rv0670 | *end* | endonuclease IV | 13, 42, 47, 130, 131, 132, 154, 168, 183, 210, 225, 233, 249 |
| Rv0671 | *lpqP* | POSSIBLE CONSERVED LIPOPROTEIN LPQP | 9, 41, 65, 104, 111, 137, 199, 201, 203, 226, 258 |
| Rv0672 | *fadE8* | PROBABLE ACYL-CoA DEHYDROGENASE FADE8 | 18, 91, 99, 173, 174, 205, 280, 306, 344, 353, 361, 376, 386, 389, 397, 405, 439, 502, 518, 526 |
| Rv0673 | *echA4* | enoyl-CoA hydratase | 20, 64, 66, 72, 77, 86, 88, 126, 148, 149, 162, 199, 204, 208, 251, 271 |
| Rv0674 | *-* | hypothetical protein Rv0674 | 4, 18, 22, 50, 56, 151, 155, 167, 182, 202, 207, 213 |
| Rv0675 | *echA5* | enoyl-CoA hydratase | 30, 46, 97, 100, 105, 139, 195, 218, 232, 243, 246 |
| Rv0676c | *mmpL5* | PROBABLE CONSERVED TRANSMEMBRANE TRANSPORT PROTEIN MMPL5 | 103, 134, 136, 145, 179, 186, 189, 198, 243, 272, 288, 303, 305, 347, 383, 389, 421, 432, 634, 666, 702, 739, 753, 758, 760, 778, 781, 800, 863, 867, 876, 881, 883 |
| Rv0677c | *mmpS5* | POSSIBLE CONSERVED MEMBRANE PROTEIN MMPS5 | 20, 60, 62, 97, 126, 128, 140 |
| Rv0678 | *-* | hypothetical protein Rv0678 | 23, 63, 85, 160 |
| Rv0679c | *-* | CONSERVED HYPOTHETICAL THREONINE RICH PROTEIN | 24, 30, 45, 47, 55, 58, 65, 70, 87, 101 |
| Rv0680c | *-* | PROBABLE CONSERVED TRANSMEMBRANE PROTEIN | 6, 11, 21, 26, 37, 62 |
| Rv0681 | *-* | PROBABLE TRANSCRIPTIONAL REGULATORY PROTEIN (POSSIBLY TETR-FAMILY) | 86, 109, 116, 123, 160, 192 |
| Rv0682 | *rpsL* | 30S ribosomal protein S12 | 20, 67, 82, 98 |
| Rv0683 | *rpsG* | 30S ribosomal protein S7 | 5, 53, 70, 80 |
| Rv0684 | *fusA1* | elongation factor G | 23, 36, 61, 189, 235, 262, 296, 375, 389, 449, 455, 522, 536, 537, 550, 562, 600 |
| Rv0685 | *tuf* | elongation factor Tu | 28, 60, 95, 97, 108, 222, 224, 229, 234, 258, 297, 318, 340, 354, 387 |
| Rv0686 | *-* | PROBABLE MEMBRANE PROTEIN | 14, 63, 67, 82, 106, 138, 158, 176, 245 |
| Rv0687 | *fabG* | 3-ketoacyl-(acyl-carrier-protein) reductase | 15, 17, 46, 107, 143, 148, 223, 253 |
| Rv0688 | *-* | PUTATIVE FERREDOXIN REDUCTASE | 21, 29, 151, 161, 173, 259, 272, 314, 377, 380, 387, 391 |
| Rv0689c | *-* | hypothetical protein Rv0689c | 26, 30, 53, 67 |
| Rv0690c | *-* | hypothetical protein Rv0690c | 20, 38, 64, 85, 86, 101, 167, 243, 253, 256, 278, 287, 293, 314 |
| Rv0691c | *-* | PROBABLE TRANSCRIPTIONAL REGULATORY PROTEIN | 42, 183, 190 |
| Rv0692 | *-* | hypothetical protein Rv0692 | 9, 24, 77, 79, 97 |
| Rv0693 | *pqqE* | PROBABLE COENZYME PQQ SYNTHESIS PROTEIN E PQQE (COENZYME PQQ SYNTHESIS PROTEIN III) | 45, 71, 89, 136, 138, 157, 195, 197, 208, 228, 245, 250, 252, 278, 285, 305, 312, 320, 329, 364 |
| Rv0694 | *lldD1* | POSSIBLE L-LACTATE DEHYDROGENASE (CYTOCHROME) LLDD1 | 80, 87, 88, 98, 119, 132, 147, 214, 217, 247, 267, 293, 309, 339, 344, 354, 381 |
| Rv0695 | *-* | hypothetical protein Rv0695 | 53, 60, 79, 86, 114, 128, 148, 157, 162, 201, 219 |
| Rv0696 | *-* | PROBABLE MEMBRANE SUGAR TRANSFERASE | 1, 39, 104, 143, 148, 232, 245, 253, 297, 311, 318, 369, 374 |
| Rv0697 | *-* | PROBABLE DEHYDROGENASE | 40, 90, 92, 97, 102, 163, 208, 229, 232, 256, 278, 279, 331, 337, 342, 360, 401, 441, 460 |
| Rv0698 | *-* | hypothetical protein Rv0698 | 16, 79, 94, 153, 191, 194, 196 |
| Rv0699 | *-* | hypothetical protein Rv0699 | 62, 67, 69 |
| Rv0700 | *rpsJ* | 30S ribosomal protein S10 | 31 |
| Rv0701 | *rplC* | 50S ribosomal protein L3 | 30, 70, 72, 90, 101, 123, 132, 151, 201 |
| Rv0702 | *rplD* | 50S ribosomal protein L4 | 44, 60, 65, 76, 90, 91, 113, 117, 169 |
| Rv0703 | *rplW* | 50S ribosomal protein L23 | 58, 70 |
| Rv0704 | *rplB* | 50S ribosomal protein L2 | 13, 39, 54, 88, 118, 137, 158, 178, 208, 233, 240, 241, 273 |
| Rv0705 | *rpsS* | 30S ribosomal protein S19 |  |
| Rv0706 | *rplV* | 50S ribosomal protein L22 | 51, 53, 71, 90, 140, 154, 158, 163, 166, 172, 177, 182 |
| Rv0707 | *rpsC* | 30S ribosomal protein S3 | 68, 93, 157, 208, 215, 237, 245, 252, 262 |
| Rv0708 | *rplP* | 50S ribosomal protein L16 | 17, 21, 60, 77, 115 |
| Rv0709 | *rpmC* | 50S ribosomal protein L29 | 37 |
| Rv0710 | *rpsQ* | 30S ribosomal protein S17 | 6, 17, 54, 105 |
| Rv0711 | *atsA* | POSSIBLE ARYLSULFATASE ATSA (ARYL-SULFATE SULPHOHYDROLASE) (ARYLSULPHATASE) | 55, 61, 79, 91, 121, 172, 192, 233, 249, 324, 344, 361, 364, 425, 440, 492, 495, 508, 525, 526, 552, 570, 619, 634, 644, 648, 693, 731, 733, 741, 756, 757 |
| Rv0712 | *-* | hypothetical protein Rv0712 | 8, 9, 51, 65, 78, 87, 121, 156, 246, 257, 282, 297 |
| Rv0713 | *-* | PROBABLE CONSERVED TRANSMEMBRANE PROTEIN | 1, 136, 178, 184, 247, 261 |
| Rv0714 | *rplN* | 50S ribosomal protein L14 | 13, 25, 32, 48, 82, 93 |
| Rv0715 | *rplX* | 50S ribosomal protein L24 | 44, 90 |
| Rv0716 | *rplE* | 50S ribosomal protein L5 | 49, 67, 98, 165 |
| Rv0717 | *rpsN* | 30S ribosomal protein S14 | 26, 53 |
| Rv0718 | *rpsH* | 30S ribosomal protein S8 | 74, 82, 98, 123 |
| Rv0719 | *rplF* | 50S ribosomal protein L6 | 11, 12, 134, 145 |
| Rv0720 | *rplR* | 50S ribosomal protein L18 | 5, 26, 37, 55, 65, 88, 89, 98, 104, 108, 113 |
| Rv0721 | *rpsE* | 30S ribosomal protein S5 | 5, 29, 64, 88, 108, 120, 121, 133, 137, 172, 187, 197, 210 |
| Rv0722 | *rpmD* | 50S ribosomal protein L30 | 43, 57, 60 |
| Rv0723 | *rplO* | 50S ribosomal protein L15 | 9, 31, 50, 117, 123, 128, 136, 139 |
| Rv0724 | *sppA* | POSSIBLE PROTEASE IV SPPA (ENDOPEPTIDASE IV) (SIGNAL PEPTIDE PEPTIDASE) | 14, 45, 78, 91, 96, 103, 259, 357, 358, 390, 417, 419, 439, 446, 524, 531, 538, 542, 551 |
| Rv0724A | *-* | hypothetical protein Rv0724A |  |
| Rv0725c | *-* | hypothetical protein Rv0725c | 17, 23, 66, 98, 108, 121, 142, 149, 170, 195, 225, 270 |
| Rv0726c | *-* | hypothetical protein Rv0726c | 3, 22, 36, 63, 127, 156, 163, 172, 185, 209, 266, 287, 331, 342, 365 |
| Rv0727c | *fucA* | L-fuculose-phosphate aldolase | 8, 13, 26, 27, 61, 62, 113, 123, 133, 135, 152, 175, 181 |
| Rv0728c | *serA2* | POSSIBLE D-3-PHOSPHOGLYCERATE DEHYDROGENASE SERA2 (PHOSPHOGLYCERATE DEHYDROGENASE) (PGDH) | 50, 53, 77, 80, 89, 92, 94, 126, 133, 148, 153, 157, 174, 190, 235, 252, 256, 258, 264, 287, 310, 314 |
| Rv0729 | *xylB* | POSSIBLE D-XYLULOSE KINASE XYLB (XYLULOKINASE) (XYLULOSE KINASE) | 14, 20, 75, 82, 86, 93, 121, 129, 136, 175, 180, 201, 203, 221, 233, 244, 304, 328, 334, 350, 361, 377, 402, 442 |
| Rv0730 | *-* | hypothetical protein Rv0730 | 20, 32, 47, 51, 67, 136, 137, 141, 145, 154, 172, 205, 229, 233, 239 |
| Rv0731c | *-* | hypothetical protein Rv0731c | 33, 37, 103, 113, 126, 147, 170, 175, 183, 195, 207, 275, 306 |
| Rv0732 | *secY* | preprotein translocase subunit SecY | 31, 36, 55, 66, 67, 85, 140, 168, 170, 183, 212, 218, 223, 256, 269, 293, 295, 352, 364, 398, 410 |
| Rv0733 | *adk* | adenylate kinase | 8, 30, 54, 147 |
| Rv0734 | *mapA* | methionine aminopeptidase | 17, 24, 31, 43, 61, 92, 98, 106, 143, 146, 167, 180, 205, 211, 239 |
| Rv0735 | *sigL* | RNA polymerase sigma factor SigL | 6, 8, 11, 22, 33, 60, 108, 123, 136, 140 |
| Rv0736 | *-* | PROBABLE CONSERVED MEMBRANE PROTEIN | 13, 51, 64, 67, 79, 84, 121, 124, 139, 167, 213, 216, 219, 220, 231, 237, 240 |
| Rv0737 | *-* | POSSIBLE TRANSCRIPTIONAL REGULATORY PROTEIN | 9, 18, 161 |
| Rv0738 | *-* | hypothetical protein Rv0738 | 14, 45, 61, 69, 75, 86, 89, 100, 120, 123, 139, 150, 168 |
| Rv0739 | *-* | hypothetical protein Rv0739 | 18, 38, 66, 89, 95, 165, 172 |
| Rv0740 | *-* | hypothetical protein Rv0740 | 11, 77, 81, 171 |
| Rv0741 | *-* | PROBABLE TRANSPOSASE (FRAGMENT) | 31, 32, 43, 86, 90 |
| Rv0742 | *PE_PGRS8* | PE-PGRS FAMILY PROTEIN | 29, 40, 80, 88, 91, 94, 128, 130, 131, 132, 134, 135, 142, 145, 147, 150, 151, 153, 154, 161, 163, 164, 166, 169, 170 |
| Rv0743c | *-* | hypothetical protein Rv0743c | 43, 89, 127, 128, 138, 143 |
| Rv0744c | *-* | POSSIBLE TRANSCRIPTIONAL REGULATORY PROTEIN | 9, 26, 80 |
| Rv0745 | *-* | hypothetical protein Rv0745 | 51, 63, 74, 78, 103, 105 |
| Rv0746 | *PE_PGRS9* | PE-PGRS FAMILY PROTEIN | 18, 30, 40, 55, 91, 92, 110, 142, 151, 152, 154, 156, 162, 163, 165, 166, 168, 169, 174, 175, 180, 181, 191, 194, 204, 208, 211, 215, 217, 220, 221, 226, 227, 233, 236, 238, 239, 245, 252, 254, 259, 263, 266, 268, 269, 271, 272, 286, 289, 295, 296, 298, 301, 302, 304, 312, 315, 316, 319, 326, 328, 331, 332, 341, 342, 344, 345, 348, 353, 357, 359, 360, 365, 371, 373, 374, 377, 384, 386, 387, 389, 396, 397, 399, 400, 402, 403, 412, 415, 418, 420, 423, 427, 470, 480, 485, 488, 491, 499, 501, 502, 504, 512, 513, 516, 519, 524, 525, 527, 528, 530, 537, 540, 543, 544, 546, 547, 554, 556, 557, 560, 567, 569, 570, 573, 581, 584, 585, 587, 590, 597, 599, 602, 603, 605, 606, 610, 615, 616, 620, 622, 623, 631, 634, 636, 637, 640, 644, 647, 649, 650, 652, 653, 661, 665, 667, 668, 677, 680, 681, 683, 684, 693, 695, 699, 705, 708, 709, 712, 716, 717, 723, 724, 727, 733, 735, 739, 742, 747, 748, 751, 754, 756, 760, 763, 765, 766, 768, 769 |
| Rv0747 | *PE_PGRS10* | PE-PGRS FAMILY PROTEIN | 40, 81, 82, 88, 91, 109, 122, 124, 128, 130, 131, 138, 140, 141, 146, 151, 154, 156, 157, 162, 165, 166, 173, 174, 176, 179, 180, 182, 183, 186, 192, 193, 196, 199, 204, 207, 208, 211, 213, 214, 217, 222, 226, 228, 236, 238, 239, 241, 242, 245, 253, 255, 256, 258, 259, 264, 268, 270, 271, 273, 274, 277, 283, 284, 286, 287, 289, 290, 300, 301, 302, 304, 305, 308, 314, 317, 320, 324, 325, 331, 332, 334, 335, 337, 344, 347, 349, 352, 362, 364, 377, 402, 417, 421, 423, 424, 431, 434, 440, 444, 446, 447, 449, 450, 452, 453, 459, 460, 462, 465, 468, 471, 474, 477, 479, 480, 482, 488, 489, 491, 492, 494, 497, 504, 506, 507, 510, 516, 517, 519, 520, 522, 525, 532, 534, 535, 538, 546, 547, 549, 555, 559, 562, 568, 573, 574, 576, 577, 579, 580, 587, 589, 590, 593, 596, 601, 602, 604, 605, 607, 612, 614, 617, 621, 623, 624, 627, 635, 636, 638, 639, 641, 647, 649, 653, 656, 658, 665, 666, 668, 669, 671, 680, 682, 683, 685, 686, 695, 696, 698, 699, 701, 704, 706, 708, 710, 711, 714, 716, 717, 720, 731, 732, 735, 738, 744, 747, 750, 759, 762, 765, 768, 771, 774, 778, 781, 783, 784, 786, 787 |
| Rv0748 | *-* | hypothetical protein Rv0748 | 13, 38, 43, 54, 56 |
| Rv0749 | *-* | hypothetical protein Rv0749 | 13, 73, 83 |
| Rv0749A | *-* | hypothetical protein Rv0749A |  |
| Rv0750 | *-* | hypothetical protein Rv0750 | 16, 77 |
| Rv0751c | *mmsB* | PROBABLE 3-HYDROXYISOBUTYRATE DEHYDROGENASE MMSB (HIBADH) | 37, 39, 66, 79, 118, 119, 124, 126, 134, 157, 159, 160, 162, 165, 194, 203, 206, 233, 237, 252, 254, 264, 272 |
| Rv0752c | *fadE9* | PROBABLE ACYL-CoA DEHYDROGENASE FADE9 | 15, 18, 31, 49, 58, 81, 127, 131, 146, 159, 161, 179, 209, 220, 226, 233, 240, 244, 259, 275, 293, 305, 343, 382 |
| Rv0753c | *mmsA* | PROBABLE METHYLMALONATE-SEMIALDEHYDE DEHYDROGENASE MMSA (METHYLMALONIC ACID SEMIALDEHYDE DEHYDROGENASE) (MMSDH) | 13, 14, 28, 38, 49, 99, 104, 127, 129, 166, 237, 240, 248, 272, 277, 294, 326, 342, 343, 462, 467 |
| Rv0754 | *PE_PGRS11* | PE-PGRS FAMILY PROTEIN | 13, 30, 36, 42, 105, 123, 158, 168, 169, 178, 181, 182, 188, 196, 199, 203, 206, 209, 211, 212, 219, 225, 228, 231, 233, 243, 246, 248, 249, 252, 261, 267, 268, 276, 296, 305, 307, 310, 331, 344, 360, 367, 378, 417, 434, 474, 485, 504, 530 |
| Rv0755c | *PPE12* | PPE FAMILY PROTEIN | 53, 54, 63, 68, 77, 102, 152, 156, 192, 208, 226, 236, 252, 292, 297, 313, 337, 347, 357, 365, 369, 371, 381, 391, 414, 419, 424, 444, 454, 459, 464, 494, 505, 514, 532, 547, 585 |
| Rv0755A | *-* | PUTATIVE TRANSPOSASE (FRAGMENT) | 56 |
| Rv0756c | *-* | hypothetical protein Rv0756c | 13, 19, 23, 28, 42, 43, 93, 94, 103, 104, 111, 114, 124, 154, 182, 218, 222 |
| Rv0757 | *phoP* | POSSIBLE TWO COMPONENT SYSTEM RESPONSE TRANSCRIPTIONAL POSITIVE REGULATOR PHOP | 48, 74, 236 |
| Rv0758 | *phoR* | POSSIBLE TWO COMPONENT SYSTEM RESPONSE SENSOR KINASE MEMBRANE ASSOCIATED PHOR | 105, 121, 171, 193, 234, 238, 268, 278, 329, 350, 428, 431, 446, 453 |
| Rv0759c | *-* | hypothetical protein Rv0759c | 29, 60 |
| Rv0760c | *-* | hypothetical protein Rv0760c | 51, 100, 107, 117 |
| Rv0761c | *adhB* | POSSIBLE ZINC-CONTAINING ALCOHOL DEHYDROGENASE NAD DEPENDENT ADHB | 59, 91, 113, 127, 168, 172, 240, 279, 315, 332, 364 |
| Rv0762c | *-* | hypothetical protein Rv0762c | 1, 25, 112, 120, 137, 146 |
| Rv0763c | *-* | POSSIBLE FERREDOXIN | 30, 45 |
| Rv0764c | *cyp51* | CYTOCHROME P450 51 CYP51 (CYPL1) (P450-L1A1) (STEROL 14-ALPHA DEMETHYLASE) (LANOSTEROL 14-ALPHA DEMETHYLASE) (P450-14DM) | 36, 45, 53, 102, 105, 130, 173, 235, 246, 255, 388, 439 |
| Rv0765c | *-* | short chain dehydrogenase | 7, 15, 25, 30, 32, 160, 163, 179, 193, 240, 259 |
| Rv0766c | *cyp123* | PROBABLE CYTOCHROME P450 123 CYP123 | 91, 238, 254, 344, 389 |
| Rv0767c | *-* | hypothetical protein Rv0767c | 130, 134, 161, 178, 180, 201 |
| Rv0768 | *aldA* | PROBABLE ALDEHYDE DEHYDROGENASE NAD DEPENDENT ALDA (ALDEHYDE DEHYDROGENASE | 38, 52, 102, 174, 235, 240, 248, 274, 288, 321, 327, 331, 354, 359, 431, 458, 463, 485 |
| Rv0769 | *-* | short chain dehydrogenase | 9, 40, 51, 66, 79, 133, 143, 171, 182, 195, 233, 241 |
| Rv0770 | *-* | PROBABLE DEHYDROGENASE/REDUCTASE | 1, 30, 48, 51, 80, 91, 126, 132, 165, 187, 189, 217, 218, 246, 278, 279 |
| Rv0771 | *-* | POSSIBLE 4-CARBOXYMUCONOLACTONE DECARBOXYLASE (CMD) | 7, 43, 59 |
| Rv0772 | *purD* | phosphoribosylamine--glycine ligase | 31, 36, 49, 84, 91, 112, 116, 139, 152, 153, 176, 219, 223, 224, 263, 273, 275, 308, 311, 338, 358, 371, 381, 386 |
| Rv0773c | *ggtA* | PROBABLE BIFUNCTIONAL ACYLASE GGTA: CEPHALOSPORIN ACYLASE (GL-7ACA ACYLASE) + GAMMA-GLUTAMYLTRANSPEPTIDASE (GGT) | 15, 26, 32, 70, 83, 98, 122, 152, 160, 161, 167, 197, 208, 217, 232, 267, 325, 330, 336, 357, 362, 364, 371, 414 |
| Rv0774c | *-* | PROBABLE CONSERVED EXPORTED PROTEIN | 17, 40, 72, 81, 93, 109, 118, 130, 186, 202, 215, 216, 221, 239, 250, 258, 273, 278, 290 |
| Rv0775 | *-* | hypothetical protein Rv0775 | 20, 28, 42, 92, 151, 154, 183 |
| Rv0776c | *-* | hypothetical protein Rv0776c | 10, 15, 31, 64, 71, 82, 100, 116, 170, 177, 200, 221, 232 |
| Rv0777 | *purB* | adenylosuccinate lyase | 88, 142, 149, 191, 200, 260, 299, 309, 385, 415, 429, 438, 440 |
| Rv0778 | *cyp126* | POSSIBLE CYTOCHROME P450 126 CYP126 | 2, 4, 73, 90, 138, 182, 197, 210, 252, 262, 263, 313, 391, 401, 411 |
| Rv0779c | *-* | POSSIBLE CONSERVED TRANSMEMBRANE PROTEIN | 11, 48, 60, 65, 85, 90, 95, 97, 105, 117, 150, 169, 184, 191, 197, 198, 203 |
| Rv0780 | *hemH* | phosphoribosylaminoimidazole-succinocarboxamide synthase | 2, 12, 53, 70, 106, 116, 120, 145, 182, 189, 230, 247, 251 |
| Rv0781 | *ptrBa* | PROBABLE PROTEASE II PTRBA | 4, 56, 126 |
| Rv0782 | *ptrBb* | PROBABLE PROTEASE II PTRBB | 6, 57, 177, 218, 235, 264, 328, 338, 340, 389, 405, 486, 506, 521, 538 |
| Rv0783c | *emrB* | POSSIBLE MULTIDRUG RESISTANCE INTEGRAL MEMBRANE EFFLUX PROTEIN EMRB | 10, 21, 43, 44, 84, 97, 208, 251, 267, 352, 395, 412, 426, 438, 443, 474, 511, 520, 538 |
| Rv0784 | *-* | hypothetical protein Rv0784 | 52, 56, 57, 75, 92, 185, 192 |
| Rv0785 | *-* | putative FAD-binding dehydrogenase | 12, 20, 28, 35, 58, 66, 128, 213, 263, 266, 280, 322, 333, 368, 377, 391, 394, 418, 436, 456, 460, 483, 519, 524, 528, 531, 533, 552, 556, 558, 560 |
| Rv0786c | *-* | hypothetical protein Rv0786c | 17, 49, 56, 66 |
| Rv0787 | *-* | hypothetical protein Rv0787 | 46, 49, 60, 106, 115, 136, 156, 212, 215, 217, 228, 231, 256, 260, 286, 299, 306 |
| Rv0787A | *-* | phosphoribosylformylglycinamidine synthase subunit PurS |  |
| Rv0788 | *purQ* | phosphoribosylformylglycinamidine synthase subunit I | 10, 18, 48, 49, 59, 75, 78, 95, 142, 179, 203 |
| Rv0789c | *-* | hypothetical protein Rv0789c | 62, 93, 105, 110, 132, 147, 172, 183, 188, 197 |
| Rv0790c | *-* | hypothetical protein Rv0790c | 7, 96, 97, 157, 221, 228 |
| Rv0791c | *-* | hypothetical protein Rv0791c | 53, 60, 63, 66, 68, 70, 74, 75, 93, 143, 165, 186, 212, 222, 243, 253, 273, 310 |
| Rv0792c | *-* | PROBABLE TRANSCRIPTIONAL REGULATORY PROTEIN (PROBABLY GNTR-FAMILY) | 40, 54, 94, 119, 158, 198, 199, 255, 259 |
| Rv0793 | *-* | hypothetical protein Rv0793 | 48, 67 |
| Rv0794c | *-* | PROBABLE OXIDOREDUCTASE | 1, 13, 96, 112, 127, 144, 151, 160, 198, 199, 207, 239, 244, 285, 325, 326, 346, 348, 354, 355, 370, 396, 425, 449, 461 |
| Rv0795 | *-* | PUTATIVE TRANSPOSASE FOR INSERTION SEQUENCE ELEMENT IS6110 (FRAGMENT) | 24, 60, 64, 71, 93 |
| Rv0796 | *-* | PUTATIVE TRANSPOSASE FOR INSERTION SEQUENCE ELEMENT IS6110 | 68, 105, 109, 125, 259, 273, 289, 309, 310 |
| Rv0797 | *-* | IS1547 transposase | 34, 72, 168, 201, 220, 240, 241, 250, 256, 268, 269, 284, 311, 347, 349 |
| Rv0798c | *cfp29* | 29 KDa ANTIGEN CFP29 | 13, 31, 41, 46, 51, 124, 162, 218, 255 |
| Rv0799c | *-* | hypothetical protein Rv0799c | 17, 41, 72, 86, 91, 96, 172, 290, 293, 294, 321 |
| Rv0800 | *pepC* | putative aminopeptidase 2 | 31, 65, 95, 130, 189, 198, 236, 251, 290, 350, 376, 384, 388, 397, 404, 415, 426 |
| Rv0801 | *-* | hypothetical protein Rv0801 | 18, 34, 56, 81, 82 |
| Rv0802c | *-* | hypothetical protein Rv0802c | 70 |
| Rv0803 | *purL* | phosphoribosylformylglycinamidine synthase II | 7, 80, 86, 118, 121, 145, 160, 163, 175, 185, 209, 211, 222, 228, 271, 281, 283, 294, 295, 298, 316, 413, 445, 456, 465, 502, 521, 532, 546, 556, 582, 588, 611, 631, 644, 667, 704, 713 |
| Rv0804 | *-* | hypothetical protein Rv0804 | 9, 10, 55, 62, 69, 103, 111, 114, 135, 160, 163, 174, 190, 194 |
| Rv0805 | *-* | hypothetical protein Rv0805 | 5, 50, 60, 136, 204, 266, 277, 287 |
| Rv0806c | *cpsY* | POSSIBLE UDP-GLUCOSE-4-EPIMERASE CPSY (GALACTOWALDENASE) (UDP-GALACTOSE-4-EPIMERASE) (URIDINE DIPHOSPHATE GALACTOSE-4-EPIMERASE) (URIDINE DIPHOSPHO-GALACTOSE-4-EPIMERASE) | 8, 24, 32, 78, 86, 132, 293, 338, 389, 412, 437, 469, 485, 511 |
| Rv0807 | *-* | hypothetical protein Rv0807 | 75, 94, 111, 115 |
| Rv0808 | *purF* | amidophosphoribosyltransferase | 11, 19, 34, 60, 64, 65, 110, 125, 129, 142, 146, 236, 290, 296, 322, 325, 334, 376, 403, 478, 493, 509, 511, 517 |
| Rv0809 | *purM* | phosphoribosylaminoimidazole synthetase | 7, 21, 27, 48, 55, 61, 107, 133, 142, 148, 151, 168, 186, 219, 248, 260, 261, 265, 282, 287, 297, 332, 347 |
| Rv0810c | *-* | hypothetical protein Rv0810c | 2, 35 |
| Rv0811c | *-* | hypothetical protein Rv0811c | 8, 124, 152, 161, 162, 185, 203, 243, 245, 294, 295, 327, 331, 338, 348, 354, 363 |
| Rv0812 | *-* | 4-amino-4-deoxychorismate lyase | 22, 82, 94, 98, 130, 162, 167, 197, 256, 282 |
| Rv0813c | *-* | hypothetical protein Rv0813c | 2, 9, 11, 12, 19, 25, 34, 48, 51, 75, 81, 99, 113, 152, 185, 210 |
| Rv0814c | *sseC2* | CONSERVED HYPOTHETICAL PROTEIN SSEC2 | 23, 35, 49, 57, 64, 66, 77 |
| Rv0815c | *cysA2* | PROBABLE THIOSULFATE SULFURTRANSFERASE CYSA2 (RHODANESE-LIKE PROTEIN) (THIOSULFATE CYANIDE TRANSSULFURASE) (THIOSULFATE THIOTRANSFERASE) | 8, 39, 83, 90, 110, 135, 169, 175, 186, 200, 220 |
| Rv0816c | *thiX* | PROBABLE THIOREDOXIN THIX | 43, 49, 51, 122, 127 |
| Rv0817c | *-* | PROBABLE CONSERVED EXPORTED PROTEIN | 10, 83, 140, 150, 151, 156, 162, 167, 200, 204, 208, 246 |
| Rv0818 | *-* | TRANSCRIPTIONAL REGULATORY PROTEIN | 55, 66, 79, 98, 100, 114, 139, 164, 223, 228, 238 |
| Rv0819 | *-* | hypothetical protein Rv0819 | 7, 10, 52, 57, 88, 92, 102, 103, 116, 120, 122, 171, 174, 183, 246, 265, 276, 305 |
| Rv0820 | *phoT* | PROBABLE PHOSPHATE-TRANSPORT ATP-BINDING PROTEIN ABC TRANSPORTER PHOT | 40, 57, 75, 97, 107, 129, 144, 145, 253 |
| Rv0821c | *phoY2* | PROBABLE PHOSPHATE-TRANSPORT SYSTEM TRANSCRIPTIONAL REGULATORY PROTEIN PHOY2 | 2, 19, 73, 146, 178, 208 |
| Rv0822c | *-* | hypothetical protein Rv0822c | 6, 29, 49, 63, 81, 83, 134, 139, 154, 156, 163, 171, 180, 187, 188, 218, 235, 243, 249, 250, 292, 324, 382, 391, 471, 481, 502, 565, 568, 573, 604, 610, 614, 677 |
| Rv0823c | *-* | POSSIBLE TRANSCRIPTIONAL REGULATORY PROTEIN | 29, 52, 61, 79, 100, 127, 129, 145, 176, 185, 194, 200, 202, 229, 239, 261, 265, 290, 317, 348, 359 |
| Rv0824c | *desA1* | PROBABLE ACYL- | 49, 103, 176, 209, 232, 278, 328 |
| Rv0825c | *-* | hypothetical protein Rv0825c | 2, 10, 27, 28, 34, 37, 39, 50, 85, 106, 160, 164, 189, 208 |
| Rv0826 | *-* | hypothetical protein Rv0826 | 31, 34, 35, 65, 109, 121, 169, 247, 311, 317, 321, 330 |
| Rv0827c | *-* | PROBABLE TRANSCRIPTIONAL REGULATORY PROTEIN | 53, 102, 104, 113, 114 |
| Rv0828c | *-* | POSSIBLE DEAMINASE | 17, 27, 86, 128 |
| Rv0829 | *-* | POSSIBLE TRANSPOSASE (FRAGMENT) | 37, 56, 66, 80 |
| Rv0830 | *-* | hypothetical protein Rv0830 | 22, 105, 135, 162, 199, 204, 216 |
| Rv0831c | *-* | hypothetical protein Rv0831c | 62, 83, 97, 139, 164, 165, 175, 210, 213, 229 |
| Rv0832 | *PE_PGRS12* | PE-PGRS FAMILY PROTEIN | 7, 40, 42, 55, 80, 92, 110, 112, 127 |
| Rv0833 | *PE_PGRS13* | PE-PGRS FAMILY PROTEIN | 4, 6, 7, 9, 12, 18, 19, 21, 31, 33, 37, 46, 50, 58, 61, 62, 64, 67, 68, 70, 76, 79, 82, 83, 88, 91, 95, 102, 105, 106, 108, 117, 121, 124, 127, 129, 130, 132, 141, 147, 151, 153, 154, 156, 157, 159, 168, 169, 174, 178, 181, 187, 188, 193, 197, 205, 208, 210, 211, 213, 216, 218, 219, 221, 222, 228, 234, 237, 240, 244, 250, 254, 256, 263, 269, 272, 275, 276, 286, 287, 289, 290, 292, 293, 296, 302, 307, 314, 317, 320, 322, 323, 325, 326, 329, 335, 338, 341, 348, 350, 353, 376, 383, 393, 408, 411, 412, 415, 420, 424, 425, 427, 430, 434, 439, 440, 446, 447, 449, 450, 453, 459, 462, 463, 465, 468, 469, 477, 480, 483, 484, 487, 490, 493, 495, 497, 500, 503, 509, 513, 515, 516, 518, 521, 525, 528, 530, 531, 533, 538, 540, 542, 543, 545, 546, 556, 558, 567, 568, 570, 571, 573, 574, 576, 585, 586, 594, 597, 598, 600, 601, 603, 610, 611, 613, 614, 616, 617, 620, 626, 627, 632, 633, 645, 646, 649, 655, 656, 659, 661, 662, 664, 670, 673, 675, 676, 679, 685, 688, 690, 691, 693, 694, 697, 701, 703, 706, 713, 716, 721, 724, 728, 730, 731, 733, 744 |
| Rv0834c | *PE_PGRS14* | PE-PGRS FAMILY PROTEIN | 25, 31, 36, 40, 80, 88, 111, 113, 131, 134, 145, 148, 156, 159, 162, 163, 169, 171, 174, 177, 184, 186, 187, 193, 197, 199, 200, 202, 203, 206, 219, 222, 225, 227, 229, 234, 237, 239, 242, 243, 252, 255, 256, 258, 263, 264, 266, 267, 269, 281, 283, 284, 294, 297, 298, 300, 306, 317, 319, 320, 323, 329, 331, 332, 334, 335, 337, 338, 352, 355, 365, 366, 368, 369, 372, 388, 390, 391, 398, 400, 401, 403, 404, 406, 411, 412, 422, 424, 425, 428, 430, 434, 435, 438, 440, 441, 449, 451, 452, 454, 455, 457, 458, 460, 465, 469, 471, 472, 481, 484, 490, 492, 496, 497, 500, 502, 505, 507, 511, 514, 516, 520, 527, 528, 530, 533, 534, 547, 550, 552, 561, 564, 568, 575, 577, 580, 581, 586, 588, 589, 592, 595, 599, 606, 610, 611, 612, 620, 622, 623, 630, 642, 648, 650, 653, 656, 657, 659, 660, 676, 678, 679, 684, 686, 691, 692, 694, 695, 698, 700, 715, 717, 718, 721, 723, 725, 727, 728, 733, 735, 737, 750, 752, 753, 764, 767, 770, 773, 784, 786, 787, 789, 790, 792, 795, 801, 804, 817, 820, 822, 828, 829, 832, 854, 860, 862, 863, 865, 869 |
| Rv0835 | *lpqQ* | POSSIBLE LIPOPROTEIN LPQQ | 33, 60, 113, 135 |
| Rv0836c | *-* | hypothetical protein Rv0836c | 16, 51, 74, 105, 129, 148, 172, 175, 191 |
| Rv0837c | *-* | hypothetical protein Rv0837c | 31, 67, 68, 92, 116, 123, 168, 171, 188, 235, 245, 269, 272 |
| Rv0838 | *lpqR* | PROBABLE CONSERVED LIPOPROTEIN LPQR | 17, 27, 50, 63, 69, 75, 88, 95, 111, 181, 221, 225, 232, 241, 243 |
| Rv0839 | *-* | hypothetical protein Rv0839 | 28, 29, 47, 49, 57, 66, 136, 173, 178, 237 |
| Rv0840c | *pip* | PROBABLE PROLINE IMINOPEPTIDASE PIP (PROLYL AMINOPEPTIDASE) (PAP) | 18, 58, 61, 154, 159, 164 |
| Rv0841 | *-* | PROBABLE CONSERVED TRANSMEMBRANE PROTEIN | 15, 49, 56, 58 |
| Rv0842 | *-* | PROBABLE CONSERVED INTEGRAL MEMBRANE PROTEIN | 3, 17, 29, 37, 96, 104, 119, 163, 164, 167, 208, 214, 301, 314, 326, 341, 342, 351, 371, 400, 415 |
| Rv0843 | *-* | PROBABLE DEHYDROGENASE | 58, 96, 114, 142, 163, 235, 255, 262, 285, 314, 323 |
| Rv0844c | *narL* | POSSIBLE NITRATE/NITRITE RESPONSE TRANSCRIPTIONAL REGULATORY PROTEIN NARL | 43, 64, 88, 104, 105, 140, 167, 172, 203 |
| Rv0845 | *-* | POSSIBLE TWO COMPONENT SENSOR KINASE | 10, 55, 112, 211, 249, 262, 272, 309, 348, 352, 376, 400, 401, 402 |
| Rv0846c | *mmcO* | PROBABLE OXIDASE | 6, 18, 23, 27, 32, 41, 43, 44, 47, 65, 92, 134, 141, 142, 238, 278, 281, 288, 323, 326, 348, 350, 374, 395, 454, 493 |
| Rv0847 | *lpqS* | PROBABLE LIPOPROTEIN LPQS | 17, 20, 76, 90, 94, 103, 105, 116 |
| Rv0848 | *cysK2* | POSSIBLE CYSTEINE SYNTHASE A CYSK2 (O-ACETYLSERINE SULFHYDRYLASE) (O-ACETYLSERINE (THIOL)-LYASE) (CSASE) | 60, 67, 83, 85, 91, 127, 131, 143, 161, 235, 242, 277, 283, 295, 309, 336 |
| Rv0849 | *-* | PROBABLE CONSERVED INTEGRAL MEMBRANE TRANSPORT PROTEIN | 39, 45, 82, 88, 89, 106, 113, 119, 122, 129, 169, 171, 216, 220, 262, 372, 384, 395, 411 |
| Rv0850 | *-* | PUTATIVE TRANSPOSASE (FRAGMENT) | 9, 29, 49 |
| Rv0851c | *-* | short chain dehydrogenase | 4, 6, 12, 21, 28, 90, 95, 134, 140, 144, 150, 174, 213, 268 |
| Rv0852 | *fadD16* | POSSIBLE FATTY-ACID-CoA LIGASE FADD16 (FATTY-ACID-CoA SYNTHETASE) (FATTY-ACID-CoA SYNTHASE) | 10, 45, 56, 68, 85, 157, 183, 195, 206, 246, 252 |
| Rv0853c | *pdc* | PROBABLE PYRUVATE OR INDOLE-3-PYRUVATE DECARBOXYLASE PDC | 35, 63, 66, 109, 153, 187, 195, 196, 209, 241, 269, 279, 289, 290, 299, 333, 343, 345, 356, 385, 425, 430, 447, 526 |
| Rv0854 | *-* | hypothetical protein Rv0854 | 42, 57, 58, 116 |
| Rv0855 | *far* | PROBABLE FATTY-ACID-CoA RACEMASE FAR | 2, 23, 75, 87, 113, 135, 159, 220, 251, 257, 267, 293, 308, 322, 329, 331, 340, 348 |
| Rv0856 | *-* | hypothetical protein Rv0856 | 99, 113, 125 |
| Rv0857 | *-* | hypothetical protein Rv0857 | 86, 89 |
| Rv0858c | *-* | aminotransferase | 17, 44, 50, 51, 58, 70, 98, 128, 142, 171, 236, 251, 255, 264, 343, 353, 394 |
| Rv0859 | *fadA* | acetyl-CoA acetyltransferase | 14, 65, 71, 82, 83, 93, 100, 115, 116, 130, 183, 228, 231, 252, 253, 263, 270, 272, 273, 288, 291, 300, 313, 363, 391 |
| Rv0860 | *fadB* | PROBABLE FATTY OXIDATION PROTEIN FADB | 23, 52, 65, 66, 80, 114, 128, 149, 198, 215, 228, 229, 277, 291, 311, 312, 337, 346, 359, 395, 398, 445, 449, 506, 541, 573, 574, 595, 612, 616, 686, 689, 691, 705 |
| Rv0861c | *ercc3* | PROBABLE DNA HELICASE ERCC3 | 15, 20, 50, 51, 137, 150, 155, 181, 188, 190, 199, 207, 212, 303, 315, 372, 444, 465, 496, 514 |
| Rv0862c | *-* | hypothetical protein Rv0862c | 14, 36, 40, 43, 77, 93, 115, 142, 151, 155, 176, 178, 214, 218, 224, 243, 270, 300, 307, 327, 360, 394, 395, 397, 439, 461, 509, 570, 607, 618, 625, 630, 639, 674, 709, 710, 725, 735 |
| Rv0863 | *-* | hypothetical protein Rv0863 | 17 |
| Rv0864 | *moaC* | molybdenum cofactor biosynthesis protein C | 40, 57, 67, 92, 125, 157, 160 |
| Rv0865 | *mog* | PROBABLE MOLYBDOPTERIN BIOSYNTHESIS MOG PROTEIN | 4, 16, 23, 60, 68, 69, 71, 92, 101, 113, 116, 153 |
| Rv0866 | *moaE2* | PROBABLE MOLYBDENUM COFACTOR BIOSYNTHESIS PROTEIN E2 MOAE2 (MOLYBDOPTERIN CONVERTING FACTOR LARGE SUBUNIT) (MOLYBDOPTERIN | 6, 52, 57, 92 |
| Rv0867c | *rpfA* | POSSIBLE RESUSCITATION-PROMOTING FACTOR RPFA | 20, 26, 32, 36, 52, 59, 75, 79, 107, 115, 127, 152, 162, 167, 178, 184, 190, 192, 207, 218, 224, 230, 232, 238, 240, 246, 248, 263, 274, 280, 285, 287, 293, 295, 301, 303, 309, 311, 317, 324, 330, 335, 359, 366, 391 |
| Rv0868c | *moaD2* | PROBABLE MOLYBDENUM COFACTOR BIOSYNTHESIS PROTEIN D 2 MOAD2 (MOLYBDOPTERIN CONVERTING FACTOR SMALL SUBUNIT) (MOLYBDOPTERIN | 7, 8, 19, 23, 26, 77, 78, 89, 90 |
| Rv0869c | *moaA* | molybdenum cofactor biosynthesis protein A | 3, 65, 94, 95, 170, 173, 178, 193, 208, 254, 266, 272, 295, 317, 331, 337, 338 |
| Rv0870c | *-* | hypothetical protein Rv0870c | 60, 61, 63, 88 |
| Rv0871 | *cspB* | PROBABLE COLD SHOCK-LIKE PROTEIN B CSPB | 2, 41, 51, 54 |
| Rv0872c | *PE_PGRS15* | PE-PGRS FAMILY PROTEIN | 29, 36, 40, 55, 69, 91, 127, 130, 131, 141, 144, 146, 147, 152, 153, 155, 156, 159, 165, 167, 170, 173, 177, 180, 190, 192, 193, 198, 201, 205, 213, 214, 223, 226, 229, 234, 244, 246, 252, 259, 264, 269, 272, 278, 279, 282, 285, 298, 310, 313, 315, 316, 319, 331, 333, 334, 340, 341, 346, 347, 350, 365, 368, 370, 380, 381, 383, 384, 387, 389, 392, 395, 403, 405, 406, 409, 414, 420, 421, 423, 426, 430, 432, 438, 449, 452, 461, 465, 468, 471, 477, 484, 486, 490, 493, 495, 501, 507, 510, 513, 524, 529, 530, 533, 536, 539, 543, 546, 548, 549, 563, 567, 572, 573, 576, 579, 582, 592, 595, 601, 604 |
| Rv0873 | *fadE10* | PROBABLE ACYL-CoA DEHYDROGENASE FADE10 | 104, 146, 161, 238, 288, 298, 336, 386, 389, 393, 398, 400, 442, 457, 461, 464, 541, 617 |
| Rv0874c | *-* | hypothetical protein Rv0874c | 15, 61, 73, 82, 90, 93, 104, 111, 118, 148, 152, 156, 175, 183, 186, 205, 216, 264, 273, 296, 309, 314, 348, 353, 357, 358, 374 |
| Rv0875c | *-* | POSSIBLE CONSERVED EXPORTED PROTEIN | 2, 19, 21, 28, 101, 126, 130 |
| Rv0876c | *-* | POSSIBLE CONSERVED TRANSMEMBRANE PROTEIN | 75, 94, 96, 134, 136, 184, 198, 252, 262, 303, 331, 394, 425, 428, 483, 517, 526 |
| Rv0877 | *-* | hypothetical protein Rv0877 | 1, 32, 56, 81, 98, 119, 137, 147, 178, 193, 200, 215, 237 |
| Rv0878c | *PPE13* | PPE FAMILY PROTEIN | 16, 34, 65, 69, 71, 90, 96, 101, 106, 182, 186, 223, 226, 253, 256, 273, 276, 296, 312, 321, 341, 349, 354, 364, 374, 381, 441 |
| Rv0879c | *-* | POSSIBLE CONSERVED TRANSMEMBRANE PROTEIN | 34, 37, 53, 70, 77, 81 |
| Rv0880 | *-* | POSSIBLE TRANSCRIPTIONAL REGULATORY PROTEIN (POSSIBLY MARR-FAMILY) | 130 |
| Rv0881 | *-* | POSSIBLE RRNA METHYLTRANSFERASE (RRNA METHYLASE) | 42, 83, 112, 126, 162, 201, 222, 243, 269, 285 |
| Rv0882 | *-* | PROBABLE TRANSMEMBRANE PROTEIN | 17, 52 |
| Rv0883c | *-* | hypothetical protein Rv0883c | 69, 106, 126, 132, 172, 176, 178, 180, 214, 223, 234, 249 |
| Rv0884c | *serC* | phosphoserine aminotransferase | 12, 39, 90, 113, 131, 155, 178, 181, 205, 215, 224, 226, 280, 304, 322, 354 |
| Rv0885 | *-* | hypothetical protein Rv0885 | 105, 167, 301, 335, 338 |
| Rv0886 | *fprB* | PROBABLE NADPH:ADRENODOXIN OXIDOREDUCTASE FPRB (ADRENODOXIN REDUCTASE) (AR) (FERREDOXIN-NADP(+) REDUCTASE) | 53, 85, 86, 102, 119, 121, 126, 153, 173, 224, 226, 276, 286, 299, 302, 319, 387, 388, 395, 415, 428, 443, 448, 458, 461, 524 |
| Rv0887c | *-* | hypothetical protein Rv0887c | 6, 37, 46, 65, 76, 105, 143 |
| Rv0888 | *spmT* | PROBABLE EXPORTED PROTEIN | 30, 53, 75, 91, 143, 146, 221, 341, 342, 355, 384, 443 |
| Rv0889c | *citA* | citrate synthase 2 | 9, 28, 62, 76, 85, 106, 125, 172, 183, 206, 227, 240, 265, 329, 371 |
| Rv0890c | *-* | PROBABLE TRANSCRIPTIONAL REGULATORY PROTEIN (PROBABLY LUXR-FAMILY) | 13, 15, 16, 48, 123, 152, 182, 205, 206, 245, 250, 256, 257, 307, 324, 351, 435, 449, 457, 459, 469, 512, 584, 593, 599, 604, 618, 621, 622, 665, 694, 712, 730, 733, 748, 750, 766, 792, 805, 807, 815, 877 |
| Rv0891c | *-* | POSSIBLE TRANSCRIPTIONAL REGULATORY PROTEIN | 113, 153, 235 |
| Rv0892 | *-* | PROBABLE MONOOXYGENASE | 1, 15, 41, 77, 120, 133, 176, 185, 200, 289, 290, 305, 306, 347, 433, 442 |
| Rv0893c | *-* | hypothetical protein Rv0893c | 21, 46, 66, 93, 94, 203, 229, 263, 290, 306 |
| Rv0894 | *-* | POSSIBLE TRANSCRIPTIONAL REGULATORY PROTEIN (POSSIBLY LUXR-FAMILY) | 65, 151, 214, 235, 259, 298, 299, 388 |
| Rv0895 | *-* | hypothetical protein Rv0895 | 33, 63, 72, 121, 123, 135, 155, 183, 210, 248, 254, 265, 299, 329, 373, 376, 391, 404, 451, 469, 491 |
| Rv0896 | *gltA* | type II citrate synthase | 6, 39, 52, 65, 71, 106, 174, 207, 267, 283, 299, 333, 368, 392, 417 |
| Rv0897c | *-* | PROBABLE OXIDOREDUCTASE | 13, 22, 28, 97, 107, 120, 128, 138, 163, 168, 184, 193, 228, 236, 258, 340, 354, 361, 384, 390, 462, 463, 501, 507, 515, 519 |
| Rv0898c | *-* | hypothetical protein Rv0898c | 66, 77, 85 |
| Rv0899 | *ompA* | OUTER MEMBRANE PROTEIN A OMPA | 10, 56, 59, 86, 100, 142, 162, 204, 263, 287, 290 |
| Rv0900 | *-* | POSSIBLE MEMBRANE PROTEIN |  |
| Rv0901 | *-* | POSSIBLE CONSERVED EXPORTED OR MEMBRANE PROTEIN | 38, 52, 82, 85, 93, 120 |
| Rv0902c | *prrB* | TWO COMPONENT SENSOR HISTIDINE KINASE PRRB | 25, 55, 110, 122, 168, 210, 246, 312, 340, 357, 365, 371, 382, 398, 422, 424, 442 |
| Rv0903c | *prrA* | TWO COMPONENT RESPONSE TRANSCRIPTIONAL REGULATORY PROTEIN PRRA | 1, 27, 32, 38, 88, 98, 102, 128, 149, 178, 196, 215, 216, 226 |
| Rv0904c | *accD3* | PUTATIVE ACETYL-COENZYME A CARBOXYLASE CARBOXYL TRANSFERASE (SUBUNIT BETA) ACCD3 (ACCASE BETA CHAIN) | 39, 45, 53, 74, 86, 93, 186, 190, 233, 289, 303, 311, 319, 324, 338, 340, 349, 352, 378, 387, 393, 418, 423, 448, 464 |
| Rv0905 | *echA6* | enoyl-CoA hydratase | 38, 49, 53, 62, 67, 95, 99, 157, 164 |
| Rv0906 | *-* | hypothetical protein Rv0906 | 9, 11, 16, 29, 58, 257, 260, 353, 360 |
| Rv0907 | *-* | hypothetical protein Rv0907 | 4, 10, 24, 35, 51, 73, 76, 85, 159, 172, 216, 224, 226, 286, 361, 368, 388, 398, 461, 503, 512 |
| Rv0908 | *ctpE* | PROBABLE METAL CATION TRANSPORTER ATPASE P-TYPE CTPE | 8, 108, 128, 152, 174, 176, 194, 225, 286, 302, 332, 335, 361, 373, 465, 533, 687, 704, 713, 736 |
| Rv0909 | *-* | hypothetical protein Rv0909 |  |
| Rv0910 | *-* | hypothetical protein Rv0910 | 4, 110, 123, 142 |
| Rv0911 | *-* | hypothetical protein Rv0911 | 38, 44, 49, 52, 57, 90, 92, 103, 136, 223, 229, 239 |
| Rv0912 | *-* | PROBABLE CONSERVED TRANSMEMBRANE PROTEIN | 13, 31, 74, 84, 118, 119, 120, 135 |
| Rv0913c | *-* | POSSIBLE DIOXYGENASE | 22, 45, 104, 105, 153, 192, 227, 321, 351, 383, 385, 403, 420, 458, 474 |
| Rv0914c | *-* | acetyl-CoA acetyltransferase | 8, 38, 44, 48, 90, 94, 126, 129, 136, 140, 193, 226, 323, 325, 340, 348, 354, 361, 374, 377 |
| Rv0915c | *PPE14* | PPE FAMILY PROTEIN | 16, 25, 28, 34, 39, 62, 66, 76, 159, 173, 176, 178, 180, 182, 188, 213, 216, 239, 264, 286, 287, 318, 339, 357, 373, 381, 383, 387, 389, 417 |
| Rv0916c | *PE7* | PE FAMILY PROTEIN | 12, 22, 55, 68, 82, 97 |
| Rv0917 | *betP* | POSSIBLE GLYCINE BETAINE TRANSPORT INTEGRAL MEMBRANE PROTEIN BETP | 5, 20, 53, 56, 57, 68, 77, 108, 133, 136, 228, 248, 251, 277, 315, 434, 479, 512, 551, 560 |
| Rv0918 | *-* | hypothetical protein Rv0918 | 16, 46, 124, 131, 148 |
| Rv0919 | *-* | hypothetical protein Rv0919 | 1, 20 |
| Rv0920c | *-* | PROBABLE TRANSPOSASE | 11, 26, 48, 64, 80, 82, 103, 147, 191, 214, 231, 273, 313, 317, 408, 434 |
| Rv0921 | *-* | POSSIBLE RESOLVASE | 15, 28, 41, 46, 74, 93, 162, 172 |
| Rv0922 | *-* | POSSIBLE TRANSPOSASE | 10, 17, 69, 71, 94, 129, 201, 213, 248, 289, 306, 308, 332, 341, 390, 408, 425, 503, 517, 520, 523, 534, 536, 548 |
| Rv0923c | *-* | hypothetical protein Rv0923c | 26, 44, 53, 141, 168, 188, 201, 262, 280, 289, 296, 310, 330, 333, 339 |
| Rv0924c | *mntH* | manganese transport protein MntH | 48, 57, 71, 76, 90, 128, 144, 148, 162, 172, 183, 198, 213, 264, 265, 275, 285, 314, 325, 350, 387, 390, 394, 426 |
| Rv0925c | *-* | hypothetical protein Rv0925c | 8, 65, 82, 97, 135, 141, 171, 186, 188, 189, 217, 218, 232 |
| Rv0926c | *-* | hypothetical protein Rv0926c | 9, 29, 39, 47, 67, 152, 247, 283, 287, 295, 316, 321, 334, 349 |
| Rv0927c | *-* | short chain dehydrogenase | 15, 16, 19, 32, 56, 64, 73, 95, 120, 136, 137, 157, 161, 200, 240, 248 |
| Rv0928 | *pstS3* | PERIPLASMIC PHOSPHATE-BINDING LIPOPROTEIN PSTS3 (PBP-3) (PSTS3) (PHOS1) | 7, 20, 29, 44, 74, 118, 165, 202, 218, 233, 280, 285, 304, 340, 343 |
| Rv0929 | *pstC2* | PHOSPHATE-TRANSPORT INTEGRAL MEMBRANE ABC TRANSPORTER PSTC2 | 17, 20, 28, 31, 32, 173, 174, 180, 233, 260, 271, 281, 292, 312, 317, 318 |
| Rv0930 | *pstA1* | PROBABLE PHOSPHATE-TRANSPORT INTEGRAL MEMBRANE ABC TRANSPORTER PSTA1 | 73, 81, 95, 98, 109, 133, 230, 269, 291 |
| Rv0931c | *pknD* | TRANSMEMBRANE SERINE/THREONINE-PROTEIN KINASE D PKND (PROTEIN KINASE D) (STPK D) | 23, 65, 122, 127, 146, 161, 170, 172, 186, 208, 223, 238, 257, 270, 289, 309, 312, 316, 328, 331, 335, 343, 351, 372, 385, 394, 396, 411, 419, 435, 454, 457, 495, 537, 548, 561, 593, 603, 621, 632, 653 |
| Rv0932c | *pstS2* | PERIPLASMIC PHOSPHATE-BINDING LIPOPROTEIN PSTS2 (PBP-2) (PSTS2) | 5, 13, 14, 22, 34, 42, 54, 83, 85, 98, 108, 118, 151, 172, 190, 225, 280, 326, 336, 363 |
| Rv0933 | *pstB* | PHOSPHATE-TRANSPORT ATP-BINDING PROTEIN ABC TRANSPORTER PSTB | 6, 9, 16, 44, 55, 74, 79, 85, 122, 191, 224, 257, 262, 273 |
| Rv0934 | *pstS1* | PERIPLASMIC PHOSPHATE-BINDING LIPOPROTEIN PSTS1 (PBP-1) (PSTS1) | 12, 29, 36, 38, 45, 55, 81, 85, 89, 94, 96, 114, 127, 136, 170, 175, 177, 188, 210, 231, 235, 282, 303, 325, 327, 358 |
| Rv0935 | *pstC1* | PHOSPHATE-TRANSPORT INTEGRAL MEMBRANE ABC TRANSPORTER PSTC1 | 4, 53, 131, 172, 184, 236, 260, 268, 279, 318, 320, 329, 335 |
| Rv0936 | *pstA2* | PHOSPHATE-TRANSPORT INTEGRAL MEMBRANE ABC TRANSPORTER PSTA2 | 6, 75, 85, 103, 112, 120, 127, 154, 173, 193, 207, 211, 239 |
| Rv0937c | *-* | hypothetical protein Rv0937c | 23, 65, 96, 188, 194, 233 |
| Rv0938 | *-* | ATP-dependent DNA ligase | 19, 21, 33, 60, 84, 96, 120, 171, 207, 220, 231, 262, 319, 385, 390, 398, 421, 468, 501, 506, 596, 650, 653, 667, 675, 680, 709 |
| Rv0939 | *-* | POSSIBLE BIFUNCTIONAL ENZYME: 2-HYDROXYHEPTA-2,4-DIENE-1,7-DIOATE ISOMERASE (HHDD ISOMERASE) + CYCLASE/DEHYDRASE | 17, 36, 53, 92, 123, 125, 143, 166, 187, 207, 228, 301, 320, 333, 357, 361, 383, 421, 443, 464, 484, 495, 499, 509, 514, 557 |
| Rv0940c | *-* | POSSIBLE OXIDOREDUCTASE | 99, 133, 149, 199 |
| Rv0941c | *-* | hypothetical protein Rv0941c | 27, 53, 92, 96, 104, 139, 162, 183, 223, 229 |
| Rv0942 | *-* | hypothetical protein Rv0942 | 20, 26, 37, 45, 80 |
| Rv0943c | *-* | PROBABLE MONOOXYGENASE | 1, 9, 40, 51, 53, 79, 96, 135, 151, 152, 182, 186, 226, 243, 272, 308, 313, 328, 338 |
| Rv0944 | *-* | POSSIBLE FORMAMIDOPYRIMIDINE-DNA GLYCOSYLASE (FAPY-DNA GLYCOSYLASE) | 1, 22, 26, 43, 65, 69, 113, 120, 142 |
| Rv0945 | *-* | short chain dehydrogenase | 2, 12, 20, 27, 84, 93, 119, 134, 156, 163, 217, 223, 226 |
| Rv0946c | *pgi* | glucose-6-phosphate isomerase | 2, 9, 39, 85, 100, 137, 141, 179, 188, 200, 222, 235, 269, 296, 307, 373, 416, 420, 443, 448, 453, 463, 526 |
| Rv0948c | *-* | hypothetical protein Rv0948c | 63 |
| Rv0949 | *uvrD1* | PROBABLE ATP-DEPENDENT DNA HELICASE II UVRD1 | 10, 48, 62, 78, 174, 264, 281, 336, 338, 395, 441, 458, 468, 514, 552, 565, 599, 611, 684, 697, 740 |
| Rv0950c | *-* | hypothetical protein Rv0950c | 118, 123, 143, 165, 222, 245, 248, 249, 297, 308, 330 |
| Rv0951 | *sucC* | succinyl-CoA synthetase subunit beta | 50, 55, 61, 120, 167, 270, 275, 286, 295, 326, 376, 380 |
| Rv0952 | *sucD* | succinyl-CoA synthetase subunit alpha | 19, 27, 39, 56, 75, 116, 143, 148, 156, 158, 229, 246, 249, 259, 264, 279, 280, 291 |
| Rv0953c | *-* | POSSIBLE OXIDOREDUCTASE | 11, 16, 18, 83, 131, 222, 223, 267, 274 |
| Rv0954 | *-* | PROBABLE CONSERVED TRANSMEMBRANE PROTEIN | 7, 14, 66, 73, 90, 129, 155, 159, 198, 204, 213, 220, 245, 259, 272, 289, 297 |
| Rv0955 | *-* | PROBABLE CONSERVED INTEGRAL MEMBRANE PROTEIN | 12, 36, 51, 91, 117, 160, 162, 174, 188, 190, 200, 269, 279, 328, 339, 376, 377, 415 |
| Rv0956 | *purN* | phosphoribosylglycinamide formyltransferase | 21, 31, 51, 55, 81, 84, 125, 141, 154 |
| Rv0957 | *purH* | bifunctional phosphoribosylaminoimidazolecarboxamide formyltransferase/IMP cyclohydrolase | 20, 32, 49, 60, 79, 91, 118, 132, 139, 161, 165, 166, 206, 232, 239, 269, 287, 313, 317, 345, 362, 367, 376, 401, 407, 449, 456, 462, 482, 487, 492, 508, 515 |
| Rv0958 | *-* | POSSIBLE MAGNESIUM CHELATASE | 14, 15, 32, 61, 71, 94, 114, 208, 297, 314, 318, 342, 349, 367, 370, 376, 385, 389, 430, 450 |
| Rv0959 | *-* | hypothetical protein Rv0959 | 30, 31, 75, 155, 179, 186, 212, 249, 251, 293, 296, 301, 307, 359, 381, 410, 421, 423, 428, 456, 481, 559, 582, 611, 632 |
| Rv0960 | *-* | hypothetical protein Rv0960 | 7, 10, 39, 114 |
| Rv0961 | *-* | PROBABLE INTEGRAL MEMBRANE PROTEIN | 31 |
| Rv0962c | *lprP* | POSSIBLE LIPOPROTEIN LPRP | 8, 15, 19, 33, 142, 172, 181, 187, 191, 215, 216 |
| Rv0963c | *-* | hypothetical protein Rv0963c | 40, 49, 76, 107, 141, 153, 164, 233, 239, 248, 252 |
| Rv0964c | *-* | hypothetical protein Rv0964c | 6, 9, 28, 37, 46, 58, 70, 93, 154 |
| Rv0965c | *-* | hypothetical protein Rv0965c | 13, 88, 104, 116, 120, 126 |
| Rv0966c | *-* | hypothetical protein Rv0966c | 15, 93, 98, 115, 160, 180 |
| Rv0967 | *-* | hypothetical protein Rv0967 | 5, 92, 99, 101 |
| Rv0968 | *-* | hypothetical protein Rv0968 | 15, 43, 57, 79 |
| Rv0969 | *ctpV* | PROBABLE METAL CATION TRANSPORTER P-TYPE ATPASE CTPV | 5, 25, 50, 69, 79, 88, 89, 110, 115, 119, 169, 178, 207, 218, 245, 289, 304, 312, 327, 342, 348, 397, 405, 431, 461, 489, 506, 511, 517, 522, 525, 535, 567, 570, 594, 612, 620, 665, 678, 693, 725, 768 |
| Rv0970 | *-* | PROBABLE CONSERVED INTEGRAL MEMBRANE PROTEIN | 11, 17, 84, 86, 107, 144, 196 |
| Rv0971c | *echA7* | enoyl-CoA hydratase | 10, 12, 59, 60, 65, 72, 73, 114, 115, 128, 160, 167, 176, 191, 194, 202, 209, 217 |
| Rv0972c | *fadE12* | PROBABLE ACYL-CoA DEHYDROGENASE FADE12 | 62, 64, 79, 81, 131, 141, 178, 183, 193, 221, 303, 342 |
| Rv0973c | *accA2* | PROBABLE ACETYL-/PROPIONYL-COENZYME A CARBOXYLASE ALPHA CHAIN (ALPHA SUBUNIT) ACCA2: BIOTIN CARBOXYLASE + BIOTIN CARBOXYL CARRIER PROTEIN (BCCP) | 10, 37, 66, 70, 90, 95, 121, 123, 138, 150, 152, 154, 171, 178, 193, 259, 298, 312, 321, 336, 366, 401, 440, 458, 467, 488, 524, 565, 593, 609, 626 |
| Rv0974c | *accD2* | PROBABLE ACETYL-/PROPIONYL-CoA CARBOXYLASE (BETA SUBUNIT) ACCD2 | 10, 20, 35, 36, 48, 88, 94, 109, 140, 141, 154, 155, 168, 181, 183, 188, 206, 215, 225, 265, 267, 320, 337, 369, 380, 411, 417, 434, 457, 464, 471, 487, 510 |
| Rv0975c | *fadE13* | PROBABLE ACYL-CoA DEHYDROGENASE FADE13 | 38, 44, 46, 47, 52, 61, 78, 82, 91, 101, 116, 128, 129, 159, 168, 172, 175, 177, 178, 190, 208, 229, 246, 260, 307, 322, 326, 339, 363, 370, 373 |
| Rv0976c | *-* | hypothetical protein Rv0976c | 14, 21, 22, 29, 51, 71, 79, 84, 85, 95, 142, 149, 163, 177, 182, 188, 193, 194, 209, 230, 231, 240, 277, 296, 307, 324, 330, 350, 374, 387, 389, 392, 403, 408, 464, 468 |
| Rv0977 | *PE_PGRS16* | PE-PGRS FAMILY PROTEIN | 18, 40, 42, 130, 137, 144, 147, 149, 152, 156, 159, 162, 168, 171, 179, 181, 184, 188, 195, 197, 201, 203, 204, 206, 207, 210, 219, 220, 225, 226, 231, 232, 235, 241, 243, 245, 247, 248, 254, 257, 260, 263, 279, 281, 282, 288, 291, 296, 308, 311, 316, 317, 319, 320, 330, 339, 342, 345, 347, 348, 351, 354, 356, 360, 361, 364, 367, 370, 373, 379, 382, 385, 388, 390, 393, 398, 406, 408, 412, 419, 423, 425, 426, 429, 432, 435, 446, 455, 456, 458, 459, 462, 464, 465, 467, 471, 480, 486, 489, 491, 494, 495, 499, 509, 514, 515, 520, 521, 523, 524, 535, 541, 545, 548, 551, 553, 557, 563, 568, 571, 572, 575, 578, 580, 583, 586, 587, 589, 593, 599, 602, 608, 611, 622, 623, 625, 626, 633, 645, 646, 676, 686, 689, 710, 711, 714, 715, 759, 781, 784, 808, 809, 839, 840, 855, 882, 891, 892, 897, 914 |
| Rv0978c | *PE_PGRS17* | PE-PGRS FAMILY PROTEIN | 13, 26, 29, 39, 50, 111, 126, 129, 130, 133, 140, 143, 148, 151, 152, 154, 155, 158, 166, 167, 169, 170, 178, 181, 182, 184, 186, 190, 193, 199, 201, 204, 205, 227, 237, 245, 251, 262, 270, 294, 302, 305, 313, 322 |
| Rv0979c | *-* | hypothetical protein Rv0979c | 34 |
| Rv0979A | *rpmF* | 50S ribosomal protein L32 | 21, 32, 45 |
| Rv0980c | *PE_PGRS18* | PE-PGRS FAMILY PROTEIN | 13, 26, 29, 39, 111, 126, 129, 130, 133, 140, 143, 148, 151, 152, 154, 155, 158, 166, 167, 170, 178, 182, 184, 185, 187, 195, 197, 201, 203, 204, 206, 213, 214, 219, 220, 226, 235, 238, 240, 241, 243, 244, 252, 254, 255, 256, 258, 259, 263, 264, 267, 273, 275, 278, 279, 301, 325, 335, 343, 367, 374, 384, 408, 427, 444 |
| Rv0981 | *mprA* | MYCOBACTERIAL PERSISTENCE REGULATOR MRPA (TWO COMPONENT RESPONSE TRANSCRIPTIONAL REGULATORY PROTEIN) | 68, 70, 80, 90, 94, 125, 218 |
| Rv0982 | *mprB* | PROBABLE TWO COMPONENT SENSOR KINASE MPRB | 48, 82, 94, 100, 129, 176, 179, 186, 246, 309, 352, 366, 373, 395, 438, 449, 462, 472 |
| Rv0983 | *pepD* | PROBABLE SERINE PROTEASE PEPD (SERINE PROTEINASE) (MTB32B) | 112, 113, 116, 119, 129, 130, 133, 146, 181, 201, 222, 245, 292, 295, 309, 313, 316, 346, 370, 398, 402, 429, 449 |
| Rv0984 | *moaB2* | POSSIBLE PTERIN-4-ALPHA-CARBINOLAMINE DEHYDRATASE MOAB2 (PHS) (4-ALPHA-HYDROXY-TETRAHYDROPTERIN DEHYDRATASE) (PTERIN-4-A-CARBINOLAMINE DEHYDRATASE) (PHENYLALANINE HYDROXYLASE-STIMULATING PROTEIN) (PHS) (PTERIN CARBINOLAMINE DEHYDRATASE) (PCD) | 3, 44, 55, 82, 91, 93, 123, 126, 132, 136, 142 |
| Rv0985c | *mscL* | large-conductance mechanosensitive channel | 61, 132 |
| Rv0986 | *-* | PROBABLE ADHESION COMPONENT TRANSPORT ATP-BINDING PROTEIN ABC TRANSPORTER | 34, 115, 147, 148, 173, 180, 220 |
| Rv0987 | *-* | PROBABLE ADHESION COMPONENT TRANSPORT TRANSMEMBRANE PROTEIN ABC TRANSPORTER | 58, 62, 73, 74, 92, 103, 210, 235, 261, 314, 356, 370, 489, 533, 537, 685, 694, 776, 780, 783, 830, 838 |
| Rv0988 | *-* | POSSIBLE CONSERVED EXPORTED PROTEIN | 6, 45, 76, 101, 267, 286, 291, 337, 339, 378 |
| Rv0989c | *grcC2* | PROBABLE POLYPRENYL-DIPHOSPHATE SYNTHASE GRCC2 (POLYPRENYL PYROPHOSPHATE SYNTHETASE) | 12, 47, 48, 117, 149, 158, 176, 183, 191, 193, 222, 225, 261, 290, 293 |
| Rv0990c | *-* | hypothetical protein Rv0990c | 31, 33, 39, 58, 64, 66, 70, 124, 154, 204 |
| Rv0991c | *-* | CONSERVED HYPOTHETICAL SERINE RICH PROTEIN | 49, 104, 106 |
| Rv0992c | *-* | hypothetical protein Rv0992c | 25, 52, 78, 79, 80, 94, 95, 115, 141, 187, 194 |
| Rv0993 | *galU* | PROBABLE UTP--GLUCOSE-1-PHOSPHATE URIDYLYLTRANSFERASE GALU (UDP-GLUCOSE PYROPHOSPHORYLASE) (UDPGP) (ALPHA-D-GLUCOSYL-1-PHOSPHATE URIDYLYLTRANSFERASE) (URIDINE DIPHOSPHOGLUCOSE PYROPHOSPHORYLASE) | 16, 40, 46, 50, 52, 144, 158, 216, 237, 272 |
| Rv0994 | *moeA1* | PROBABLE MOLYBDOPTERIN BIOSYNTHESIS PROTEIN MOEA1 | 12, 43, 48, 61, 66, 94, 112, 131, 140, 157, 262, 263, 266, 356, 372, 381, 405 |
| Rv0995 | *rimJ* | POSSIBLE RIBOSOMAL-PROTEIN-ALANINE ACETYLTRANSFERASE RIMJ (ACETYLATING ENZYME FOR N-TERMINAL OF RIBOSOMAL PROTEIN S5) | 84, 112, 117, 196 |
| Rv0996 | *-* | PROBABLE CONSERVED TRANSMEMBRANE PROTEIN | 58, 120, 124, 156, 192, 209, 221, 223, 248, 266 |
| Rv0997 | *-* | hypothetical protein Rv0997 | 1, 11, 53, 66, 88, 115 |
| Rv0998 | *-* | hypothetical protein Rv0998 | 7, 42, 108, 114, 124, 133, 139, 162, 176, 259, 269 |
| Rv0999 | *-* | hypothetical protein Rv0999 | 9, 57, 60, 89, 93, 113, 119, 126, 129, 153, 163, 169, 196, 201, 208, 247 |
| Rv1000c | *-* | hypothetical protein Rv1000c | 5, 38, 39, 98, 107, 108, 129, 155, 172 |
| Rv1001 | *arcA* | arginine deiminase | 61, 79, 81, 88, 122, 146, 199, 209, 328, 351, 378, 384, 387, 396 |
| Rv1002c | *-* | hypothetical protein Rv1002c | 6, 22, 27, 48, 75, 107, 136, 149, 152, 187, 191, 208, 221, 261, 285, 315, 328, 361, 376, 410, 450, 483 |
| Rv1003 | *-* | hypothetical protein Rv1003 | 24, 28, 86, 94, 113, 116, 120, 125, 200, 211, 225, 278, 279 |
| Rv1004c | *-* | PROBABLE MEMBRANE PROTEIN | 19, 23, 44, 49, 64, 71, 102, 113, 119, 122, 136, 161, 168, 172, 174, 192, 211, 218, 230, 237, 245, 261, 262, 285, 287, 317, 368, 384, 408 |
| Rv1005c | *pabB* | para-aminobenzoate synthase component I | 39, 64, 95, 96, 101, 105, 117, 129, 130, 131, 152, 216, 220, 234, 239, 243, 261, 310, 350, 358, 377, 381, 391, 406, 415, 419, 443 |
| Rv1006 | *-* | hypothetical protein Rv1006 | 33, 56, 72, 81, 128, 178, 201, 219, 263, 286, 305, 341, 361, 400, 411, 446, 453, 461, 482, 504, 507, 515, 545, 561 |
| Rv1007c | *metG* | methionyl-tRNA synthetase | 7, 45, 57, 65, 112, 121, 149, 197, 198, 236, 253, 278, 295, 384, 389, 398, 470, 487, 495, 503 |
| Rv1008 | *tatD* | PROBABLE DEOXYRIBONUCLEASE TATD (YJJV PROTEIN) | 10, 25, 28, 31, 42, 93, 126, 172, 179, 186 |
| Rv1009 | *rpfB* | Probable resuscitation-promoting factor rpfB | 15, 21, 33, 63, 65, 98, 117, 128, 145, 165, 177, 247, 271, 288, 292, 300, 319 |
| Rv1010 | *ksgA* | dimethyladenosine transferase | 4, 49, 51, 76, 81, 124, 128, 169, 193, 209, 272, 275, 291, 292, 300 |
| Rv1011 | *ispE* | 4-diphosphocytidyl-2-C-methyl-D-erythritol kinase | 2, 10, 99, 100, 103, 133, 148, 154, 176, 181, 202, 228, 242, 246, 254, 290, 294 |
| Rv1012 | *-* | hypothetical protein Rv1012 | 24, 85 |
| Rv1013 | *pks16* | acyl-CoA synthetase | 13, 43, 46, 60, 76, 160, 163, 249, 269, 282, 289, 329, 349, 381, 383, 391, 397, 442, 455, 473, 492, 522 |
| Rv1014c | *pth* | peptidyl-tRNA hydrolase | 32, 45, 55, 71, 108, 112, 122, 140, 168, 182 |
| Rv1015c | *rplY* | 50S ribosomal protein L25/general stress protein Ctc | 3, 16, 27, 31, 47, 58, 69, 115, 155, 156, 163, 183, 200, 204 |
| Rv1016c | *lpqT* | PROBABLE CONSERVED LIPOPROTEIN LPQT | 1, 29, 69, 184, 218 |
| Rv1017c | *prsA* | ribose-phosphate pyrophosphokinase | 15, 56, 93, 115, 125, 153, 159, 178, 221, 234, 235, 239, 250, 265, 273 |
| Rv1018c | *glmU* | Probable UDP-N-acetylglucosamine pyrophosphorylase glmU | 6, 13, 15, 31, 88, 94, 97, 104, 181, 189, 193, 235, 256, 261, 298, 334, 344, 346, 367, 413, 431, 433, 465 |
| Rv1019 | *-* | PROBABLE TRANSCRIPTIONAL REGULATORY PROTEIN (PROBABLY TETR-FAMILY) | 20, 136, 173 |
| Rv1020 | *mfd* | PROBABLE TRANSCRIPTION-REPAIR COUPLING FACTOR MFD (TRCF) | 1, 32, 65, 67, 75, 102, 184, 190, 254, 258, 262, 269, 325, 372, 399, 404, 423, 424, 427, 434, 436, 450, 479, 492, 498, 519, 560, 583, 593, 626, 654, 674, 721, 736, 763, 846, 872, 920, 956, 972, 1000, 1002, 1011, 1015, 1040, 1048, 1085, 1126, 1149, 1159, 1177, 1199 |
| Rv1021 | *-* | nucleoside triphosphate pyrophosphohydrolase | 22, 47, 69, 70, 98, 99, 128, 235, 314 |
| Rv1022 | *lpqU* | PROBABLE CONSERVED LIPOPROTEIN LPQU | 15, 39, 50, 51, 54, 82, 136, 137, 148, 154, 214, 234 |
| Rv1023 | *eno* | phosphopyruvate hydratase | 15, 34, 42, 53, 58, 105, 150, 156, 198, 204, 211, 214, 231, 251, 261, 294, 354, 381 |
| Rv1024 | *-* | POSSIBLE CONSERVED MEMBRANE PROTEIN | 20, 26, 33, 39, 78, 86, 187, 202, 209, 225 |
| Rv1025 | *-* | hypothetical protein Rv1025 | 24, 77, 102, 139, 145 |
| Rv1026 | *-* | hypothetical protein Rv1026 | 6, 10, 24, 28, 50, 63, 87, 107, 110, 113, 118, 137, 148, 201, 225, 245, 265, 279, 296, 317 |
| Rv1027c | *kdpE* | PROBABLE TRANSCRIPTIONAL REGULATORY PROTEIN KDPE | 29, 39, 64, 119, 139, 167, 173 |
| Rv1028c | *kdpD* | PROBABLE SENSOR PROTEIN KDPD | 25, 36, 54, 71, 137, 161, 207, 219, 247, 261, 262, 299, 327, 367, 368, 378, 384, 401, 424, 441, 445, 475, 517, 522, 570, 573, 592, 600, 606, 619, 669, 684, 698, 726, 731, 749, 796, 816, 826, 833, 843 |
| Rv1028A | *kdpF* | Probable membrane protein kdpF |  |
| Rv1029 | *kdpA* | potassium-transporting ATPase subunit A | 1, 18, 23, 37, 57, 109, 123, 135, 144, 146, 147, 220, 236, 244, 327, 328, 351, 369, 385, 387, 405, 443, 451, 483, 486, 515, 527, 550, 556 |
| Rv1030 | *kdpB* | potassium-transporting ATPase subunit B | 8, 10, 88, 108, 175, 186, 190, 208, 226, 262, 263, 288, 314, 325, 352, 395, 401, 409, 418, 446, 447, 497, 503, 511, 535, 550, 563, 571, 686 |
| Rv1031 | *kdpC* | Probable Potassium-transporting ATPase C chain KDPC (Potassium-translocating ATPase C chain) (ATP phosphohydrolase | 6, 18, 27, 52, 60, 102, 122, 126 |
| Rv1032c | *trcS* | TWO COMPONENT SENSOR HISTIDINE KINASE TRCS | 51, 82, 95, 99, 124, 140, 160, 192, 197, 216, 218, 408, 462, 479, 507 |
| Rv1033c | *trcR* | TWO COMPONENT TRANSCRIPTIONAL REGULATOR TRCR | 109, 119, 143, 155, 246 |
| Rv1034c | *-* | PROBABLE TRANSPOSASE (FRAGMENT) | 22, 30, 33, 81, 92, 98, 102, 127 |
| Rv1035c | *-* | PROBABLE TRANSPOSASE (FRAGMENT) | 13, 68, 70, 76, 78, 92, 116, 122, 131, 154, 158, 160 |
| Rv1036c | *-* | truncated IS1560 transposase | 2 |
| Rv1037c | *esxI* | PUTATIVE ESAT-6 LIKE PROTEIN ESXI (ESAT-6 LIKE PROTEIN 1) | 20, 36, 42, 44, 77 |
| Rv1038c | *esxJ* | ESAT-6 LIKE PROTEIN ESXJ (ESAT-6 LIKE PROTEIN 2) | 35 |
| Rv1039c | *PPE15* | PPE FAMILY PROTEIN | 11, 16, 25, 26, 28, 34, 41, 66, 78, 81, 85, 94, 155, 159, 160, 176, 179, 182, 189, 191, 227, 252, 259, 263, 290, 294, 329, 334, 335, 343, 348, 352, 365, 369, 373 |
| Rv1040c | *PE8* | PE FAMILY PROTEIN | 14, 30, 83, 97, 135, 140, 149, 150, 162, 170, 183, 188, 198, 207, 210, 228, 230, 234, 241, 249, 254 |
| Rv1041c | *-* | PROBABLE IS LIKE-2 TRANSPOSASE | 124, 147, 151, 193, 220, 221, 229, 240, 249, 264 |
| Rv1042c | *-* | PROBABLE IS LIKE-2 TRANSPOSASE | 25, 53, 83 |
| Rv1043c | *-* | hypothetical protein Rv1043c | 56, 105, 131, 145, 162, 190, 216, 256, 261, 278, 305, 314, 331 |
| Rv1044 | *-* | hypothetical protein Rv1044 | 23, 51, 77, 120, 195 |
| Rv1045 | *-* | hypothetical protein Rv1045 | 52, 61, 111, 129, 151, 171 |
| Rv1046c | *-* | hypothetical protein Rv1046c | 134 |
| Rv1047 | *-* | PROBABLE TRANSPOSASE | 25, 33, 44, 71, 157, 164, 187, 204, 209, 221, 224, 235, 239, 246, 305, 313, 322, 401 |
| Rv1048c | *-* | hypothetical protein Rv1048c | 114, 172, 183, 209, 251, 256, 262, 293, 313, 314, 353, 356 |
| Rv1049 | *-* | PROBABLE TRANSCRIPTIONAL REPRESSOR PROTEIN | 32, 52, 62, 101, 116, 123 |
| Rv1050 | *-* | PROBABLE OXIDOREDUCTASE | 13, 17, 23, 53, 92, 155, 159, 165, 178, 200, 294 |
| Rv1051c | *-* | hypothetical protein Rv1051c | 5, 67, 109, 146, 158 |
| Rv1052 | *-* | hypothetical protein Rv1052 | 6, 42, 50, 63 |
| Rv1053c | *-* | hypothetical protein Rv1053c | 15, 54 |
| Rv1054 | *-* | PROBABLE INTEGRASE (FRAGMENT) | 1, 42, 46, 61 |
| Rv1055 | *-* | POSSIBLE INTEGRASE (FRAGMENT) | 31 |
| Rv1056 | *-* | hypothetical protein Rv1056 | 22, 82, 220, 226 |
| Rv1057 | *-* | hypothetical protein Rv1057 | 22, 26, 35, 145, 193, 194, 262, 290, 299, 329, 350, 364, 374 |
| Rv1058 | *fadD14* | acyl-CoA synthetase | 12, 19, 29, 60, 177, 185, 188, 271, 301, 361, 388, 407, 412, 436, 437, 459 |
| Rv1059 | *-* | hypothetical protein Rv1059 | 41, 86, 109, 115, 118, 126, 130, 146, 175, 183, 208, 223, 235, 239, 277, 286, 305, 311, 316, 329, 344, 347, 349 |
| Rv1060 | *-* | hypothetical protein Rv1060 | 65, 108, 111, 128, 144, 151 |
| Rv1061 | *-* | hypothetical protein Rv1061 | 8, 14, 37, 71, 84, 141, 153, 177, 198, 251 |
| Rv1062 | *-* | hypothetical protein Rv1062 | 9, 10, 14, 24, 48, 53, 79, 144, 150, 184, 191, 207, 213, 214, 223, 234, 266 |
| Rv1063c | *-* | hypothetical protein Rv1063c | 1, 22, 40, 48, 62, 91, 120, 128, 136, 150, 166, 178, 195, 197, 206, 218, 225, 246, 263, 276, 296, 353 |
| Rv1064c | *lpqV* | POSSIBLE LIPOPROTEIN LPQV | 23, 27, 33, 40, 53, 62, 76, 85, 87, 104, 131, 137 |
| Rv1065 | *-* | hypothetical protein Rv1065 | 9, 37, 74, 84, 94, 114, 141 |
| Rv1066 | *-* | hypothetical protein Rv1066 | 9, 28, 117, 118, 129 |
| Rv1067c | *PE_PGRS19* | PE-PGRS FAMILY PROTEIN | 12, 18, 30, 39, 41, 47, 55, 58, 80, 122, 130, 138, 146, 147, 150, 153, 154, 157, 163, 168, 169, 171, 175, 178, 181, 187, 191, 193, 194, 196, 198, 199, 211, 212, 214, 218, 224, 227, 230, 233, 236, 240, 242, 245, 249, 252, 254, 255, 258, 260, 261, 270, 271, 274, 276, 277, 279, 280, 285, 288, 295, 297, 301, 303, 306, 310, 313, 317, 320, 323, 326, 328, 331, 332, 333, 335, 338, 345, 347, 348, 350, 351, 353, 354, 356, 357, 360, 373, 376, 378, 379, 384, 385, 387, 388, 395, 396, 405, 408, 410, 413, 416, 424, 426, 427, 429, 432, 436, 438, 442, 449, 451, 454, 457, 459, 460, 462, 463, 466, 473, 475, 476, 478, 482, 484, 485, 487, 488, 491, 502, 505, 508, 511, 514, 517, 520, 523, 529, 532, 533, 535, 536, 541, 542, 544, 547, 554, 558, 559, 560, 563, 565, 569, 575, 578, 582, 584, 586, 587, 590, 592, 596, 600, 602, 603, 609, 612, 615, 618, 627, 630, 633, 638, 642, 647, 648, 650, 651, 661 |
| Rv1068c | *PE_PGRS20* | PE-PGRS FAMILY PROTEIN | 31, 33, 41, 55, 122, 130, 138, 146, 147, 150, 153, 154, 157, 163, 168, 169, 171, 175, 178, 181, 187, 191, 193, 194, 196, 198, 199, 211, 212, 214, 218, 224, 226, 227, 229, 230, 233, 235, 236, 238, 242, 245, 249, 252, 255, 257, 258, 260, 264, 267, 270, 273, 280, 282, 283, 285, 288, 289, 291, 292, 295, 300, 304, 307, 313, 316, 319, 322, 325, 332, 334, 335, 337, 343, 344, 346, 347, 349, 353, 356, 357, 366, 368, 369, 371, 374, 375, 380, 383, 386, 387, 390, 393, 397, 399, 405, 411, 414, 423, 426, 432, 434, 438, 446, 447, 450, 454, 457, 460 |
| Rv1069c | *-* | hypothetical protein Rv1069c | 17, 25, 49, 51, 78, 81, 155, 267, 275, 301, 312, 326, 328, 337, 347, 373, 407, 428, 446, 557, 563 |
| Rv1070c | *echA8* | enoyl-CoA hydratase | 54, 61, 62, 79, 90, 106, 120, 123, 140, 160, 166, 172 |
| Rv1071c | *echA9* | enoyl-CoA hydratase | 1, 13, 56, 58, 61, 65, 66, 114, 148, 157, 164, 172, 200, 201, 208, 220, 231, 246, 275 |
| Rv1072 | *-* | PROBABLE CONSERVED TRANSMEMBRANE PROTEIN | 16, 25, 67, 137, 200, 210 |
| Rv1073 | *-* | hypothetical protein Rv1073 | 26, 60, 108, 160, 191, 251 |
| Rv1074c | *fadA3* | acetyl-CoA acetyltransferase | 36, 60, 63, 105, 113, 146, 149, 150, 174, 182, 253, 289, 314, 392 |
| Rv1075c | *-* | CONSERVED EXPORTED PROTEIN | 10, 55, 99, 105, 125, 173, 189, 217, 241, 250, 311 |
| Rv1076 | *lipU* | POSSIBLE LIPASE LIPU | 38, 46, 70, 139, 141, 254, 295 |
| Rv1077 | *cbs* | Probable cystathionine beta-synthase CBS (Serine sulfhydrase) (Beta-thionase) (Hemoprotein H-450) | 26, 30, 39, 53, 57, 71, 74, 85, 160, 181, 182, 186, 196, 214, 216, 266, 269, 275, 283, 296, 337, 341, 378, 382, 418, 425 |
| Rv1078 | *pra* | Probable Proline-rich antigen homolog pra | 6, 7, 20, 27, 31, 32, 44, 56, 67, 68, 75, 154, 188 |
| Rv1079 | *metB* | cystathionine gamma-synthase | 28, 47, 51, 58, 64, 84, 87, 99, 111, 141, 188, 210, 238, 242, 252, 293, 298, 303, 306, 315, 355 |
| Rv1080c | *greA* | transcription elongation factor GreA | 31, 47, 51, 82, 87 |
| Rv1081c | *-* | PROBABLE CONSERVED MEMBRANE PROTEIN | 34, 36, 54, 97 |
| Rv1082 | *mca* | Mycothiol conjugate amidase Mca (Mycothiol S-conjugate amidase) | 39, 43, 87, 134, 155, 197, 250, 273, 283 |
| Rv1083 | *-* | hypothetical protein Rv1083 | 11, 13, 16 |
| Rv1084 | *-* | hypothetical protein Rv1084 | 6, 41, 54, 73, 105, 108, 125, 171, 198, 201, 241, 248, 286, 295, 298, 343, 359, 372, 386, 398, 414, 426, 431, 455, 462, 473, 492, 497, 508, 514, 581, 593, 619, 625, 641, 645, 666, 671 |
| Rv1085c | *-* | POSSIBLE HEMOLYSIN-LIKE PROTEIN | 1, 6, 9, 36, 43, 44, 46, 50, 63, 74, 94, 114, 139, 143, 174, 181, 184, 192, 199, 225 |
| Rv1086 | *-* | SHORT (C15) CHAIN Z-ISOPRENYL DIPHOSPHATE SYNTHASE (Z-FPP SYNTHASE) (Z-FARNESYL DIPHOSPHATE SYNTHASE) (Z-FPP SYNTHETASE) (Z-FARNESYL DIPHOSPHATE SYNTHETASE) (GERANYLTRANSTRANSFERASE) (FARNESYL PYROPHOSPHATE SYNTHETASE) | 0 |
| Rv1087 | *PE_PGRS21* | PE-PGRS FAMILY PROTEIN | 13, 29, 36, 47, 82, 83, 106, 127, 133, 135, 136, 146, 155, 156, 161, 162, 172, 174, 176, 179, 180, 183, 185, 186, 193, 195, 196, 198, 199, 205, 208, 209, 211, 212, 214, 215, 217, 218, 221, 223, 224, 233, 238, 243, 244, 252, 253, 255, 256, 259, 261, 262, 265, 270, 271, 278, 280, 287, 289, 290, 292, 293, 299, 301, 304, 307, 311, 313, 316, 317, 321, 325, 326, 331, 332, 335, 352, 356, 358, 359, 361, 364, 367, 370, 376, 380, 381, 383, 384, 389, 390, 393, 395, 396, 398, 406, 408, 410, 411, 414, 417, 420, 423, 426, 429, 444, 447, 450, 453, 458, 459, 465, 468, 471, 474, 477, 479, 480, 482, 485, 491, 504, 505, 507, 511, 513, 514, 517, 519, 525, 531, 532, 535, 537, 543, 545, 548, 549, 551, 552, 563, 566, 568, 569, 570, 573, 577, 591, 595, 606, 610, 612, 615, 616, 621, 628, 631, 639, 640, 642, 643, 646, 650, 652, 653, 662, 665, 669, 672, 675, 689, 696, 701, 709, 716, 718, 721, 725, 734, 737, 739, 740, 742, 751, 752, 755 |
| Rv1087A | *-* | hypothetical protein Rv1087A | 45, 52 |
| Rv1088 | *PE9* | PE FAMILY PROTEIN | 29, 33, 36, 40, 41, 83 |
| Rv1089 | *PE10* | PE FAMILY PROTEIN | 2, 6, 27, 34, 36, 39, 71, 79 |
| Rv1089A | *celA2a* | PROBABLE CELLULASE CELA2A (ENDO-1,4-BETA-GLUCANASE) (ENDOGLUCANASE) (CARBOXYMETHYL CELLULASE) |  |
| Rv1090 | *celA2b* | PROBABLE CELLULASE CELA2B (ENDO-1,4-BETA-GLUCANASE) (ENDOGLUCANASE) (CARBOXYMETHYL CELLULASE) | 25, 43, 112, 129 |
| Rv1091 | *PE_PGRS22* | PE-PGRS FAMILY PROTEIN | 29, 40, 81, 83, 91, 127, 133, 135, 143, 146, 151, 152, 155, 156, 161, 162, 168, 170, 172, 174, 176, 179, 180, 183, 186, 193, 195, 196, 198, 205, 208, 209, 212, 215, 217, 223, 224, 226, 230, 241, 245, 253, 254, 260, 262, 266, 269, 272, 274, 281, 285, 290, 293, 294, 302, 303, 305, 309, 311, 312, 317, 318, 321, 325, 328, 330, 333, 336, 344, 346, 347, 351, 353, 358, 366, 368, 371, 374, 375, 377, 383, 384, 386, 398, 399, 401, 402, 407, 408, 410, 411, 414, 419, 424, 426, 427, 430, 433, 436, 438, 439, 442, 444, 445, 452, 455, 458, 460, 461, 463, 467, 470, 472, 474, 479, 482, 485, 488, 490, 497, 499, 501, 502, 504, 505, 512, 515, 518, 520, 522, 523, 524, 526, 527, 530, 531, 533, 537, 540, 543, 546, 549, 554, 562, 564, 565, 570, 571, 573, 574, 576, 586, 589, 590, 596, 598, 601, 602, 604, 608, 614, 616, 617, 622, 623, 625, 629, 632, 634, 641, 644, 647, 650, 653, 655, 656, 658, 665, 666, 671, 672, 675, 677, 681, 684, 686, 689, 696, 699, 701, 702, 705, 707, 711, 715, 725, 727, 728, 730, 734, 738, 739, 743, 750, 755, 762, 767, 768, 775, 777, 778, 783, 787, 789, 792, 793, 795, 796, 799, 811, 820, 822, 828, 829, 831, 832, 835, 845 |
| Rv1092c | *coaA* | pantothenate kinase | 37, 64, 73, 95, 105, 166, 207, 267 |
| Rv1093 | *glyA* | serine hydroxymethyltransferase | 56, 62, 99, 108, 132, 158, 169, 206, 229, 233, 252, 256, 264, 273, 287, 304, 310, 366, 371, 387, 391 |
| Rv1094 | *desA2* | POSSIBLE ACYL- | 49, 79, 103, 160, 169, 178, 215, 218 |
| Rv1095 | *phoH2* | PROBABLE PHOH-LIKE PROTEIN PHOH2 (PHOSPHATE STARVATION-INDUCIBLE PROTEIN PSIH) | 78, 94, 110, 114, 139, 151, 161, 163, 187, 193, 224, 245, 248, 253, 283, 291, 367, 392, 418, 429 |
| Rv1096 | *-* | POSSIBLE GLYCOSYL HYDROLASE | 16, 21, 28, 31, 84, 87, 88, 119, 130, 133, 143, 144, 154, 284 |
| Rv1097c | *-* | PROBABLE MEMBRANE GLYCINE AND PROLINE RICH PROTEIN | 4, 30, 44, 58, 70, 89, 114, 116, 123, 124, 146, 170, 193, 207, 231 |
| Rv1098c | *fumC* | fumarate hydratase | 41, 43, 80, 82, 101, 122, 153, 170, 200, 208, 226, 228, 232, 252, 277, 303, 336, 344, 391, 421 |
| Rv1099c | *glpX* | fructose 1,6-bisphosphatase II | 8, 12, 19, 24, 43, 102, 123, 130, 171, 184, 194, 203, 211, 219, 240, 255, 279, 280, 293, 312, 317 |
| Rv1100 | *-* | hypothetical protein Rv1100 | 20, 67, 101, 107, 110, 122, 139, 172, 197, 200, 208, 210, 230 |
| Rv1101c | *-* | hypothetical protein Rv1101c | 57, 61, 70, 129, 155, 156, 160, 210, 226, 228, 234, 248, 256, 275, 317, 382 |
| Rv1102c | *-* | hypothetical protein Rv1102c |  |
| Rv1103c | *-* | hypothetical protein Rv1103c | 9, 10, 94, 100 |
| Rv1104 | *-* | POSSIBLE PARA-NITROBENZYL ESTERASE (FRAGMENT) | 31, 95, 126, 128, 182, 194 |
| Rv1105 | *-* | POSSIBLE PARA-NITROBENZYL ESTERASE (FRAGMENT) | 5, 21, 53, 77, 105 |
| Rv1106c | *-* | PROBABLE CHOLESTEROL DEHYDROGENASE | 19, 20, 36, 70, 82, 90, 108, 116, 120, 136, 141, 142, 185, 234, 245, 270, 280, 282, 301, 364 |
| Rv1107c | *xseB* | exodeoxyribonuclease VII small subunit | 40, 77 |
| Rv1108c | *xseA* | exodeoxyribonuclease VII large subunit | 70, 84, 91, 147, 152, 207, 254, 260, 268, 336, 346, 355, 360, 375 |
| Rv1109c | *-* | hypothetical protein Rv1109c | 138 |
| Rv1110 | *ispH* | 4-hydroxy-3-methylbut-2-enyl diphosphate reductase | 9, 76, 96, 259, 263, 298, 307 |
| Rv1111c | *-* | hypothetical protein Rv1111c | 34, 44, 56, 61, 77, 91, 112, 128, 157, 164, 235, 287 |
| Rv1112 | *-* | translation-associated GTPase | 64, 73, 83, 115, 172, 178, 183, 223, 231, 245, 257, 277, 293, 298, 322, 332 |
| Rv1113 | *-* | hypothetical protein Rv1113 | 47, 54 |
| Rv1114 | *-* | hypothetical protein Rv1114 | 14, 28, 96, 114 |
| Rv1115 | *-* | POSSIBLE EXPORTED PROTEIN | 39, 44, 45, 72, 77, 88, 117, 124, 157, 196, 199, 210, 213 |
| Rv1116 | *-* | hypothetical protein Rv1116 | 12 |
| Rv1116A | *-* | CONSERVED HYPOTHETICAL PROTEIN (FRAGMENT) | 22, 29, 72, 77, 80 |
| Rv1117 | *-* | hypothetical protein Rv1117 | 25, 60, 61 |
| Rv1118c | *-* | hypothetical protein Rv1118c | 28, 31, 70, 77, 124, 129, 144, 183, 187, 192, 203, 258, 263, 284 |
| Rv1119c | *-* | hypothetical protein Rv1119c | 1, 10, 43 |
| Rv1120c | *-* | hypothetical protein Rv1120c | 14, 34, 65, 95, 119, 151, 159 |
| Rv1121 | *zwf1* | glucose-6-phosphate 1-dehydrogenase | 3, 17, 148, 215, 308, 316, 321, 399, 436, 443, 449, 452, 461 |
| Rv1122 | *gnd2* | 6-phosphogluconate dehydrogenase-like protein | 39, 45, 58, 68, 74, 86, 121, 136, 158, 182, 226, 262, 276, 288, 298, 304, 331 |
| Rv1123c | *bpoB* | POSSIBLE PEROXIDASE BPOB (NON-HAEM PEROXIDASE) | 27, 43, 57, 67, 84, 86, 93, 125, 136, 178, 219, 247, 280, 282 |
| Rv1124 | *ephC* | PROBABLE EPOXIDE HYDROLASE EPHC (EPOXIDE HYDRATASE) | 4, 38, 52, 66, 105, 122, 124, 127, 186, 213, 280, 281, 291, 314 |
| Rv1125 | *-* | hypothetical protein Rv1125 | 1, 125, 174, 190, 226, 236, 240, 247, 309, 339, 349, 365 |
| Rv1126c | *-* | hypothetical protein Rv1126c | 32, 39, 41, 78, 114, 196 |
| Rv1127c | *ppdK* | pyruvate phosphate dikinase | 23, 65, 67, 72, 113, 137, 159, 199, 214, 219, 229, 240, 241, 249, 258, 274, 303, 363, 370, 372, 407, 418, 437, 439, 446 |
| Rv1128c | *-* | hypothetical protein Rv1128c | 16, 43, 69, 94, 104, 113, 115, 171, 193, 209, 228, 300, 302, 307, 308, 370, 397 |
| Rv1129c | *-* | PROBABLE TRANSCRIPTIONAL REGULATOR PROTEIN | 17, 28, 128, 141, 148, 182, 186, 187, 214, 234, 269, 281, 294, 341, 349, 359, 397, 429 |
| Rv1130 | *-* | hypothetical protein Rv1130 | 79, 124, 136, 159, 165, 169, 179, 199, 200, 246, 259, 280, 291, 309, 353, 440, 498 |
| Rv1131 | *gltA1* | methylcitrate synthase | 1, 17, 32, 39, 121, 136, 162, 171, 190, 214, 230, 242, 324, 334, 336, 358, 361, 381 |
| Rv1132 | *-* | hypothetical protein Rv1132 | 100, 138, 166, 180, 204, 206, 230, 279, 284, 311, 318, 395, 401, 414, 431, 472, 518, 545, 550 |
| Rv1133c | *metE* | 5-methyltetrahydropteroyltriglutamate--homocysteine methyltransferase | 10, 14, 34, 46, 58, 60, 79, 102, 140, 156, 201, 222, 230, 271, 278, 284, 290, 382, 408, 412, 415, 454, 558, 578, 595, 596, 635, 705, 715, 731, 742, 750 |
| Rv1134 | *-* | hypothetical protein Rv1134 | 1, 11, 16, 25, 32, 35, 41 |
| Rv1135c | *PPE16* | PPE FAMILY PROTEIN | 11, 16, 25, 34, 48, 54, 65, 86, 92, 98, 110, 144, 155, 161, 180, 184, 186, 199, 220, 227, 237, 240, 247, 250, 257, 267, 287, 297, 300, 350, 362, 372, 382, 392, 402, 407, 417, 424, 467, 477, 488, 492, 497, 510, 517, 525, 530, 537, 550, 551, 563, 572, 577, 586, 592, 597, 603, 607, 612 |
| Rv1135A | *-* | POSSIBLE ACETYL-CoA ACETYLTRANSFERASE (ACETOACETYL-CoA THIOLASE) | 38, 68 |
| Rv1136 | *-* | POSSIBLE ENOYL-CoA HYDRATASE | 40, 42, 49, 73, 86 |
| Rv1137c | *-* | hypothetical protein Rv1137c | 13, 28, 48, 64, 87 |
| Rv1138c | *-* | POSSIBLE OXIDOREDUCTASE | 11, 14, 15, 44, 50, 86, 128, 130, 156, 157, 225, 253, 260, 271, 288, 302 |
| Rv1139c | *-* | hypothetical protein Rv1139c | 30, 47, 139 |
| Rv1140 | *-* | PROBABLE INTEGRAL MEMBRANE PROTEIN | 5, 14, 19, 101, 119, 135, 144, 204, 209, 254 |
| Rv1141c | *echA11* | enoyl-CoA hydratase | 3, 6, 63, 67, 71, 82, 87, 101, 150, 157, 160, 208, 224, 265 |
| Rv1142c | *echA10* | enoyl-CoA hydratase | 65, 67, 71, 150, 158, 177, 192, 208, 265 |
| Rv1143 | *mcr* | PROBABLE ALPHA-METHYLACYL-CoA RACEMASE MCR (2-methylacyl-CoA racemase) (2-arylpropionyl-CoA epimerase ) | 5, 57, 85, 111, 116, 123, 157, 169, 177, 218, 249, 255, 323, 336, 345 |
| Rv1144 | *-* | PROBABLE SHORT-CHAIN TYPE DEHYDROGENASE/REDUCTASE | 10, 37, 43, 60, 82, 84, 100, 134, 140, 153, 160 |
| Rv1145 | *mmpL13a* | PROBABLE CONSERVED TRANSMEMBRANE TRANSPORT PROTEIN MMPL13A | 25, 39, 63, 74, 112, 132, 159, 160, 201, 202 |
| Rv1146 | *mmpL13b* | PROBABLE CONSERVED TRANSMEMBRANE TRANSPORT PROTEIN MMPL13B | 8, 16, 149, 168, 171, 207, 214, 239, 250, 268, 291, 328, 330, 333, 343, 356, 361, 372, 446, 453 |
| Rv1147 | *-* | hypothetical protein Rv1147 | 5, 24, 40, 51, 84, 136, 148, 183, 197 |
| Rv1148c | *-* | hypothetical protein Rv1148c | 37, 79, 98, 113, 133, 142, 144, 146, 169, 179, 222, 238, 257, 297, 305, 329, 331, 336, 426, 468 |
| Rv1149 | *-* | POSSIBLE TRANSPOSASE | 25, 53, 83 |
| Rv1151c | *-* | NAD-dependent deacetylase | 11, 14, 72, 94, 96, 139, 185, 194, 209, 232 |
| Rv1152 | *-* | PROBABLE TRANSCRIPTIONAL REGULATORY PROTEIN | 30, 35, 46, 87, 91 |
| Rv1153c | *omt* | PROBABLE O-METHYLTRANSFERASE OMT | 5, 13, 18, 106, 151, 152, 153, 158, 184, 224 |
| Rv1154c | *-* | hypothetical protein Rv1154c | 29, 30, 45, 50, 77, 86, 88, 143, 150, 156 |
| Rv1155 | *-* | hypothetical protein Rv1155 | 143 |
| Rv1156 | *-* | hypothetical protein Rv1156 | 36, 39, 53, 105, 106, 122, 151, 157, 169 |
| Rv1157c | *-* | CONSERVED HYPOTHETICAL ALA-, PRO-RICH PROTEIN | 25, 33, 85, 91, 156, 163, 166, 179, 184, 210, 224, 261, 268, 274, 276, 278, 281, 293, 313, 338 |
| Rv1158c | *-* | CONSERVED HYPOTHETICAL ALA-, PRO-RICH PROTEIN | 9, 17, 23, 48, 76, 78, 81, 84, 93, 101, 108, 127, 136, 146, 153, 160, 209, 212, 216, 223 |
| Rv1159 | *pimE* | mannosyltransferase | 28, 42, 75, 83, 149, 152, 156, 216, 229, 277, 288, 399, 415 |
| Rv1159A | *phhB* | pterin-4-alpha-carbinolamine dehydratase | 10, 21, 36 |
| Rv1160 | *mutT2* | PROBABLE MUTATOR PROTEIN MUTT (7,8-dihydro-8-oxoguanine-triphosphatase) (8-OXO-DGTPASE) | 7, 12, 34, 35, 39, 40, 46, 90, 119, 132 |
| Rv1161 | *narG* | PROBABLE RESPIRATORY NITRATE REDUCTASE (ALPHA CHAIN) NARG | 17, 36, 64, 162, 180, 196, 210, 220, 250, 283, 308, 309, 313, 361, 366, 381, 486, 491, 509, 513, 530, 539, 562, 569, 585, 604, 632, 636, 672, 721, 783, 823, 846, 869, 890, 933, 956, 975, 1014, 1036, 1111, 1178, 1196, 1212 |
| Rv1162 | *narH* | PROBABLE RESPIRATORY NITRATE REDUCTASE (BETA CHAIN) NARH | 62, 78, 121, 129, 146, 218, 225, 238, 283, 310, 324, 398, 471, 486, 493, 517, 522, 523 |
| Rv1163 | *narJ* | PROBABLE RESPIRATORY NITRATE REDUCTASE (DELTA CHAIN) NARJ | 51, 74, 75, 81, 90, 95, 115, 122, 138, 160, 179 |
| Rv1164 | *narI* | PROBABLE RESPIRATORY NITRATE REDUCTASE (GAMMA CHAIN) NARI | 12, 98, 99, 238 |
| Rv1165 | *typA* | POSSIBLE GTP-BINDING TRANSLATION ELONGATION FACTOR TYPA (TYROSINE PHOSPHORYLATED PROTEIN A) (GTP-BINDING PROTEIN) | 26, 32, 58, 89, 120, 162, 177, 180, 289, 296, 308, 310, 347, 381, 440, 454, 466, 482, 504, 533, 569, 612, 614 |
| Rv1166 | *lpqW* | PROBABLE CONSERVED LIPOPROTEIN LPQW | 25, 63, 78, 99, 112, 140, 160, 161, 174, 187, 211, 251, 259, 263, 297, 336, 340, 364, 367, 372, 407, 414, 431, 454, 484, 485, 499, 517, 525, 530, 555, 568, 581, 605, 626, 632 |
| Rv1167c | *-* | PROBABLE TRANSCRIPTIONAL REGULATORY PROTEIN | 5, 14, 32, 33, 36, 49, 52, 59, 67, 94, 101, 147, 159, 174, 194 |
| Rv1168c | *PPE17* | PPE FAMILY PROTEIN | 24, 75, 127, 156, 193, 217, 266, 303, 306, 322, 335, 342, 344 |
| Rv1169c | *PE11* | PE FAMILY PROTEIN | 14, 41 |
| Rv1170 | *mshB* | N-Acetyl-1-D-myo-Inosityl-2-Amino-2-Deoxy-alpha- D-Glucopyranoside Deacetylase mshB (GlcNAc-Ins deacetylase) | 56, 72, 87, 95, 98, 139, 154, 164, 166, 170, 234, 252, 255, 275, 276, 278, 293, 298, 300 |
| Rv1171 | *-* | hypothetical protein Rv1171 | 17, 42, 73, 84, 101 |
| Rv1172c | *PE12* | PE FAMILY PROTEIN | 13, 18, 26, 36, 42, 51, 55, 89, 94, 105, 139, 145, 148, 152, 155, 163, 167, 170, 179, 182, 184, 217, 273, 289 |
| Rv1173 | *fbiC* | FO synthase | 9, 48, 50, 61, 72, 78, 184, 247, 256, 300, 303, 308, 348, 354, 371, 380, 383, 392, 421, 459, 496, 505, 506, 518, 528, 559, 593, 602, 611, 644, 658, 666, 695, 732, 733, 753, 758, 807, 819 |
| Rv1174c | *TB8.4* | LOW MOLECULAR WEIGHT T-CELL ANTIGEN TB8.4 | 5, 9, 22, 26, 55, 73, 83, 103 |
| Rv1175c | *fadH* | PROBABLE NADPH DEPENDENT 2,4-DIENOYL-COA REDUCTASE FADH (2,4-dienoyl coenzyme A reductase) (4-enoyl-CoA reductase) | 29, 50, 58, 66, 106, 136, 155, 186, 205, 249, 267, 296, 361, 386, 394, 421, 451, 456, 465, 475, 490, 504, 505, 541, 552, 574, 626, 654 |
| Rv1176c | *-* | hypothetical protein Rv1176c | 33, 73, 91, 93, 112, 118, 125, 139, 155, 164 |
| Rv1177 | *fdxC* | PROBABLE FERREDOXIN FDXC | 82 |
| Rv1178 | *-* | N-succinyldiaminopimelate aminotransferase | 19, 51, 58, 59, 61, 106, 128, 158, 210, 225, 249, 263, 283, 291, 301, 309, 317 |
| Rv1179c | *-* | hypothetical protein Rv1179c | 12, 32, 76, 185, 199, 212, 220, 273, 306, 369, 378, 399, 401, 428, 451, 455, 479, 492, 497, 511, 513, 514, 531, 572, 601, 692, 697, 720, 729, 735, 744, 747, 760, 780, 789, 791, 797, 826, 851, 871, 922 |
| Rv1180 | *pks3* | PROBABLE POLYKETIDE BETA-KETOACYL SYNTHASE PKS3 | 2, 4, 19, 33, 42, 89, 136, 149, 157, 169, 175, 179, 201, 225, 240, 269, 314, 328, 403, 444, 464 |
| Rv1181 | *pks4* | PROBABLE POLYKETIDE BETA-KETOACYL SYNTHASE PKS4 | 31, 38, 45, 59, 88, 105, 132, 155, 166, 196, 255, 268, 288, 314, 325, 329, 338, 347, 387, 414, 418, 436, 446, 478, 482, 499, 517, 525, 533, 562, 644, 657, 720, 755, 771, 790, 836, 856, 863, 872, 877, 887, 904, 930, 961, 965, 981, 984, 1010, 1012, 1017, 1019, 1044, 1057, 1058, 1065, 1112, 1114, 1126, 1207, 1209, 1212, 1222, 1246, 1257, 1265, 1289, 1290, 1298, 1304, 1315, 1322, 1362, 1376, 1424, 1430, 1436, 1454, 1488, 1563, 1575 |
| Rv1182 | *papA3* | PROBABLE CONSERVED POLYKETIDE SYNTHASE ASSOCIATED PROTEIN PAPA3 | 76, 118, 195, 282, 294, 302, 329, 339, 346, 349, 400, 404, 464 |
| Rv1183 | *mmpL10* | PROBABLE CONSERVED TRANSMEMBRANE TRANSPORT PROTEIN MMPL10 | 50, 143, 154, 207, 213, 219, 276, 278, 314, 320, 329, 335, 346, 424, 446, 465, 473, 483, 504, 573, 599, 653, 671, 862, 904, 915, 919, 932, 966 |
| Rv1184c | *-* | POSSIBLE EXPORTED PROTEIN | 16, 21, 28, 48, 88, 89, 134, 174, 190, 218, 253, 255, 338, 348, 354, 357 |
| Rv1185c | *fadD21* | acyl-CoA synthetase | 13, 20, 32, 60, 84, 149, 163, 233, 265, 367, 394, 512, 513, 529, 547, 555, 561 |
| Rv1186c | *-* | hypothetical protein Rv1186c | 3, 26, 49, 94, 99, 136, 186, 206, 270, 316, 328, 334, 359, 374, 383, 490 |
| Rv1187 | *rocA* | PROBABLE PYRROLINE-5-CARBOXYLATE DEHYDROGENASE ROCA | 46, 66, 80, 82, 86, 103, 109, 114, 122, 194, 199, 229, 230, 234, 258, 263, 293, 316, 350, 388, 395, 449, 456, 469, 498, 501, 509 |
| Rv1188 | *-* | PROBABLE PROLINE DEHYDROGENASE | 1, 14, 15, 32, 40, 52, 77, 93, 205, 213, 278, 280 |
| Rv1189 | *sigI* | RNA polymerase sigma factor SigI | 83, 120, 203, 231, 241, 255, 264, 287 |
| Rv1190 | *-* | hypothetical protein Rv1190 | 6, 31, 116, 119, 123, 127, 181, 224, 280 |
| Rv1191 | *-* | hypothetical protein Rv1191 | 68, 107, 116, 117, 125, 126, 130, 136, 142, 159, 275 |
| Rv1192 | *-* | hypothetical protein Rv1192 | 7, 15, 46, 60, 96, 126, 156, 187 |
| Rv1193 | *fadD36* | acyl-CoA synthetase | 11, 43, 55, 63, 68, 76, 91, 127, 137, 140, 154, 201, 207, 237, 238, 249, 266, 301, 312, 328, 343, 355, 387 |
| Rv1194c | *-* | hypothetical protein Rv1194c | 76, 105, 148, 151, 200, 237, 265, 274, 358, 375, 401 |
| Rv1195 | *PE13* | PE FAMILY PROTEIN | 29, 41, 55 |
| Rv1196 | *PPE18* | PPE FAMILY PROTEIN | 12, 17, 26, 41, 79, 86, 148, 155, 184, 193, 273, 279, 301, 305, 324, 345, 350, 355, 366, 389 |
| Rv1197 | *esxK* | ESAT-6 LIKE PROTEIN ESXK (ESAT-6 LIKE PROTEIN 3) | 35, 40 |
| Rv1198 | *esxL* | PUTATIVE ESAT-6 LIKE PROTEIN ESXL (ESAT-6 LIKE PROTEIN 4) | 20, 36, 42, 44, 77 |
| Rv1199c | *-* | POSSIBLE TRANSPOSASE | 25, 33, 44, 71, 157, 164, 187, 204, 209, 221, 224, 235, 239, 246, 305, 313, 322, 401 |
| Rv1200 | *-* | PROBABLE CONSERVED INTEGRAL MEMBRANE TRANSPORT PROTEIN | 23, 42, 102, 120, 124, 135, 150, 239, 240, 269, 287, 291, 305, 323, 340, 374, 375, 383, 416, 417 |
| Rv1201c | *-* | PROBABLE TRANSFERASE | 15, 26, 51, 78, 103, 104, 136, 157, 189, 214, 225, 243, 257, 262, 263, 281, 294 |
| Rv1202 | *dapE* | dipeptidase | 4, 11, 66, 90, 97, 106, 145, 155, 156, 174, 226, 229, 252, 274, 276, 285, 293, 294, 295, 311, 341 |
| Rv1203c | *-* | hypothetical protein Rv1203c | 14, 22, 29, 69, 100, 107, 136, 163, 170, 176 |
| Rv1204c | *-* | hypothetical protein Rv1204c | 16, 46, 55, 73, 75, 82, 94, 124, 165, 202, 223, 272, 273, 340, 346, 353, 355, 396, 409, 469, 484, 496, 500, 522 |
| Rv1205 | *-* | hypothetical protein Rv1205 | 7, 29, 37, 63, 113, 159 |
| Rv1206 | *fadD6* | acyl-CoA synthetase | 5, 36, 56, 85, 89, 91, 95, 119, 123, 131, 160, 164, 185, 200, 210, 320, 346, 363, 364, 380, 388, 399, 439, 451, 496, 497, 500, 527, 571 |
| Rv1207 | *folP2* | PROBABLE DIHYDROPTEROATE SYNTHASE 2 FOLP2 (DHPS 2) (Dihydropteroate pyrophosphorylase 2) | 8, 17, 26, 32, 84, 88, 111, 155, 167, 168, 186, 198, 206, 241, 274, 279, 280, 291 |
| Rv1208 | *-* | hypothetical protein Rv1208 | 7, 11, 28, 40, 80, 93, 111, 125, 153, 180, 181, 195, 209, 226, 285, 294, 295 |
| Rv1209 | *-* | hypothetical protein Rv1209 | 63, 109, 120 |
| Rv1210 | *tagA* | PROBABLE DNA-3-METHYLADENINE GLYCOSYLASE I TAGA (TAG I) (3-methyladenine-DNA glycosylase I, constitutive) (DNA-3-methyladenine glycosidase I ) | 1, 158, 167, 175 |
| Rv1211 | *-* | hypothetical protein Rv1211 | 9, 27, 39, 50, 62 |
| Rv1212c | *-* | PUTATIVE GLYCOSYL TRANSFERASE | 17, 66, 74, 88, 95, 106, 122, 134, 138, 180, 182, 185, 219, 302, 310, 321, 333, 359 |
| Rv1213 | *glgC* | glucose-1-phosphate adenylyltransferase | 12, 13, 23, 34, 99, 101, 143, 145, 150, 160, 170, 184, 292, 306, 308, 316, 349, 355, 372, 391, 392 |
| Rv1214c | *PE14* | PE FAMILY PROTEIN | 20, 27, 31, 71, 74 |
| Rv1215c | *-* | hypothetical protein Rv1215c | 15, 26, 114, 125, 137, 145, 190, 216, 217, 230, 273, 284, 293, 295, 304, 315, 329, 332, 349, 360, 370, 408, 412, 455, 486, 487, 506, 515, 526, 531 |
| Rv1216c | *-* | PROBABLE CONSERVED INTEGRAL MEMBRANE PROTEIN | 25, 38, 56, 65, 106, 133, 160, 184 |
| Rv1217c | *-* | PROBABLE TETRONASIN-TRANSPORT INTEGRAL MEMBRANE PROTEIN ABC TRANSPORTER | 10, 18, 23, 67, 70, 74, 102, 145, 158, 174, 186, 194, 197, 217, 227, 261, 274, 280, 287, 289, 291, 292, 308, 340, 350, 352, 371, 387, 396, 414, 423, 427, 439, 448, 513, 518 |
| Rv1218c | *-* | PROBABLE TETRONASIN-TRANSPORT ATP-BINDING PROTEIN ABC TRANSPORTER | 12, 62, 69, 97, 110, 163, 183, 209, 215, 275 |
| Rv1219c | *-* | PROBABLE TRANSCRIPTIONAL REGULATORY PROTEIN | 6, 103, 104, 129, 148, 150 |
| Rv1220c | *-* | PROBABLE METHYLTRANSFERASE | 27, 38, 56, 66, 68, 71, 116, 156, 157, 168, 171 |
| Rv1221 | *sigE* | RNA polymerase sigma factor SigE | 4, 68, 74, 102, 166, 230 |
| Rv1222 | *-* | hypothetical protein Rv1222 | 3, 62, 84, 142 |
| Rv1223 | *htrA* | PROBABLE SERINE PROTEASE HTRA (DEGP PROTEIN) | 32, 36, 62, 116, 126, 138, 140, 142, 150, 159, 161, 172, 191, 194, 201, 225, 233, 254, 261, 294, 386, 387, 402, 403, 412, 466, 470 |
| Rv1224 | *tatB* | sec-independent translocase | 44, 93 |
| Rv1225c | *-* | hypothetical protein Rv1225c | 7, 26, 28, 62, 68, 75, 88, 105, 132, 173, 178, 184, 187, 189, 202, 226, 239, 252 |
| Rv1226c | *-* | PROBABLE TRANSMEMBRANE PROTEIN | 40, 77, 115, 120, 123, 153, 197, 202, 206, 210, 246, 249, 289, 298, 317, 342, 358, 383, 410, 423, 461, 469, 471 |
| Rv1227c | *-* | PROBABLE TRANSMEMBRANE PROTEIN | 10, 37, 65, 67, 70, 102, 124, 168, 171 |
| Rv1228 | *lpqX* | PROBABLE LIPOPROTEIN LPQX | 16, 37, 56, 68, 74, 98, 116, 148 |
| Rv1229c | *mrp* | PROBABLE MRP-RELATED PROTEIN MRP | 10, 23, 48, 63, 132, 135, 147, 153, 212, 241, 243, 268, 276, 311, 342, 343, 375 |
| Rv1230c | *-* | POSSIBLE MEMBRANE PROTEIN | 50, 53, 64, 67, 69, 74, 118, 128, 130, 142, 154, 160, 162, 204, 231, 238, 251, 270, 271, 275, 286, 305, 315, 353, 375, 384, 409 |
| Rv1231c | *-* | PROBABLE MEMBRANE PROTEIN | 40, 85 |
| Rv1232c | *-* | hypothetical protein Rv1232c | 64, 75, 106, 112, 193, 248, 269, 272, 287, 317, 356, 380, 424, 430 |
| Rv1233c | *-* | hypothetical protein Rv1233c | 4, 14, 54, 62, 66, 74, 80, 85, 109, 126, 176 |
| Rv1234 | *-* | PROBABLE TRANSMEMBRANE PROTEIN | 10, 14, 16, 25, 87, 112, 116, 120, 137, 150 |
| Rv1235 | *lpqY* | PROBABLE SUGAR-BINDING LIPOPROTEIN LPQY | 4, 20, 25, 47, 58, 84, 136, 226, 246, 269, 340, 349, 388, 417, 453 |
| Rv1236 | *sugA* | PROBABLE SUGAR-TRANSPORT INTEGRAL MEMBRANE PROTEIN ABC TRANSPORTER SUGA | 7, 9, 15, 31, 63, 121, 146, 188, 246, 247, 252, 296, 299 |
| Rv1237 | *sugB* | PROBABLE SUGAR-TRANSPORT INTEGRAL MEMBRANE PROTEIN ABC TRANSPORTER SUGB | 174, 192, 212, 219, 228, 237, 242, 264 |
| Rv1238 | *sugC* | PROBABLE SUGAR-TRANSPORT ATP-BINDING PROTEIN ABC TRANSPORTER SUGC | 38, 63, 173, 225, 247, 355, 372, 382 |
| Rv1239c | *corA* | POSSIBLE MAGNESIUM AND COBALT TRANSPORT TRANSMEMBRANE PROTEIN CORA | 36, 41, 46, 52, 64, 139, 160, 244, 275, 292, 309, 346 |
| Rv1240 | *mdh* | malate dehydrogenase | 10, 12, 13, 26, 66, 110, 120, 129, 132, 168, 171, 201, 226, 234, 239, 242, 289, 296, 305, 312 |
| Rv1241 | *-* | hypothetical protein Rv1241 | 58, 63 |
| Rv1242 | *-* | hypothetical protein Rv1242 | 14, 57, 62, 111 |
| Rv1243c | *PE_PGRS23* | PE-PGRS FAMILY PROTEIN | 12, 14, 25, 40, 55, 83, 91, 123, 139, 142, 151, 152, 154, 158, 166, 169, 170, 172, 176, 179, 182, 192, 205, 211, 214, 225, 231, 254, 263, 272, 275, 279, 282, 294, 297, 298, 301, 309, 313, 318, 321, 328, 331, 333, 334, 337, 339, 340, 342, 346, 367, 368, 371, 376, 379, 380, 382, 397, 403, 404, 407, 418, 419, 424, 427, 435, 437, 438, 441, 453, 455, 459, 462, 476, 479, 482, 485, 487, 488, 490, 494, 497, 499, 500, 503, 512, 514, 517, 521, 524, 527, 530, 532, 535, 539, 542, 551 |
| Rv1244 | *lpqZ* | PROBABLE LIPOPROTEIN LPQZ | 26, 47, 65, 82, 85, 117, 123, 137, 142, 154, 162, 167, 176, 189, 193, 203, 209, 220, 249, 265, 274, 275 |
| Rv1245c | *-* | PROBABLE SHORT-CHAIN TYPE DEHYDROGENASE/REDUCTASE | 4, 11, 90, 95, 131, 162, 186, 187, 191, 198, 224, 255, 257, 267 |
| Rv1246c | *-* | hypothetical protein Rv1246c | 11, 26, 60 |
| Rv1247c | *-* | hypothetical protein Rv1247c | 71, 86 |
| Rv1248c | *kgd* | alpha-ketoglutarate decarboxylase | 63, 66, 77, 79, 89, 107, 109, 118, 152, 188, 215, 250, 259, 273, 287, 381, 426, 489, 574, 584, 601, 649, 652, 672, 687, 748, 771, 831, 858, 908, 942, 943, 957, 997, 1080, 1127, 1196 |
| Rv1249c | *-* | POSSIBLE MEMBRANE PROTEIN | 15, 26, 28, 29, 70, 105, 109, 111, 121, 168, 203 |
| Rv1250 | *-* | PROBABLE DRUG-TRANSPORT INTEGRAL MEMBRANE PROTEIN | 8, 73, 100, 104, 106, 111, 120, 140, 161, 162, 227, 240, 386, 396, 419, 427, 431, 438, 440, 441, 469, 479, 487, 534, 539 |
| Rv1251c | *-* | hypothetical protein Rv1251c | 3, 16, 48, 81, 82, 87, 105, 140, 200, 203, 248, 260, 264, 275, 308, 322, 339, 388, 447, 452, 469, 507, 537, 540, 542, 556, 559, 618, 650, 653, 674, 690, 707, 712, 720, 735, 737, 750, 774, 825, 844, 845, 850, 888, 917, 933, 967, 1022, 1046, 1049, 1064, 1078, 1084, 1105 |
| Rv1252c | *lprE* | PROBABLE LIPOPROTEIN LPRE | 1, 22, 26, 28, 61, 72, 110, 130, 157, 175, 189, 199, 200 |
| Rv1253 | *deaD* | PROBABLE COLD-SHOCK DEAD-BOX PROTEIN A HOMOLOG DEAD (ATP-dependent RNA helicase deaD homolog) | 10, 58, 60, 117, 126, 130, 139, 193, 220, 275, 280, 311, 340, 343, 348, 400, 426, 434, 553 |
| Rv1254 | *-* | PROBABLE ACYLTRANSFERASE | 7, 10, 25, 31, 44, 81, 84, 104, 126, 234, 286 |
| Rv1255c | *-* | PROBABLE TRANSCRIPTIONAL REGULATORY PROTEIN | 7, 14, 21, 62, 105, 108, 119, 123, 133, 166, 190, 195 |
| Rv1256c | *cyp130* | PROBA BLE CYTOCHROME P450 130 CYP130 | 12, 105, 135, 183, 197, 207, 217, 218, 226, 227, 241, 242, 248, 252, 309, 357, 387, 388 |
| Rv1257c | *-* | PROBABLE OXIDOREDUCTASE | 21, 37, 71, 73, 77, 80, 84, 104, 109, 119, 140, 147, 148, 173, 186, 213, 239, 244, 289, 293, 350, 369, 382, 385, 406, 410, 424, 450 |
| Rv1258c | *-* | PROBABLE CONSERVED INTEGRAL MEMBRANE TRANSPORT PROTEIN | 5, 19, 61, 63, 86, 96, 113, 130, 168, 176, 188, 195, 209, 235, 267, 268, 292, 344, 357, 358, 366, 372, 389, 397 |
| Rv1259 | *-* | hypothetical protein Rv1259 | 20, 35, 45, 47, 64, 71, 135, 143, 148, 163, 174, 186, 228, 259, 276 |
| Rv1260 | *-* | hypothetical protein Rv1260 | 6, 11, 33, 60, 90, 91, 312, 318, 349 |
| Rv1261c | *-* | hypothetical protein Rv1261c | 34, 45 |
| Rv1262c | *-* | HYPOTHETICAL HIT-LIKE PROTEIN | 9, 21, 34, 88, 126, 139 |
| Rv1263 | *amiB2* | amidase | 8, 10, 23, 59, 66, 86, 95, 100, 152, 155, 164, 176, 186, 242, 244, 248, 267, 310, 349, 373, 375, 408, 411, 444 |
| Rv1264 | *-* | ADENYLYL CYCLASE (ATP PYROPHOSPHATE-LYASE) (ADENYLATE CYCLASE) | 20, 22, 67, 110, 140, 147, 200, 205, 241, 245, 252, 298, 302, 309, 324, 356, 380, 382, 383, 392 |
| Rv1265 | *-* | hypothetical protein Rv1265 | 50, 51, 208 |
| Rv1266c | *pknH* | PROBABLE TRANSMEMBRANE SERINE/THREONINE-PROTEIN KINASE H PKNH (PROTEIN KINASE H) (STPK H) | 24, 47, 67, 128, 173, 209, 219, 251, 264, 270, 283, 296, 310, 318, 344, 351, 377, 385, 386, 405, 407, 494, 496, 499, 526, 603, 615 |
| Rv1267c | *embR* | PROBABLE TRANSCRIPTIONAL REGULATORY PROTEIN EMBR | 56, 85, 119, 125, 127, 139, 174, 212, 242, 264, 268, 270, 298, 304, 358, 385 |
| Rv1268c | *-* | hypothetical protein Rv1268c | 7, 11, 17, 18, 28, 42, 60, 68, 90, 136, 140, 143, 151, 175, 187, 201 |
| Rv1269c | *-* | CONSERVED PROBABLE SECRETED PROTEIN | 15, 20, 29, 34, 44, 64, 81, 83, 87, 102, 113 |
| Rv1270c | *lprA* | POSSIBLE LIPOPROTEIN LPRA | 9, 12, 22, 30, 36, 60, 93, 123, 161, 175, 187, 238 |
| Rv1271c | *-* | CONSERVED HYPOTHETICAL SECRETED PROTEIN | 18, 27, 28, 30, 31, 70, 73 |
| Rv1272c | *-* | PROBABLE DRUGS-TRANSPORT TRANSMEMBRANE ATP-BINDING PROTEIN ABC TRANSPORTER | 1, 10, 49, 103, 116, 175, 254, 266, 279, 294, 323, 330, 356, 364, 393, 433, 450, 505, 530, 605, 611, 621 |
| Rv1273c | *-* | PROBABLE DRUGS-TRANSPORT TRANSMEMBRANE ATP-BINDING PROTEIN ABC TRANSPORTER | 38, 50, 64, 81, 105, 131, 137, 144, 154, 201, 229, 233, 265, 314, 360, 364, 370, 388, 422, 432, 451, 474, 475, 501, 534, 568 |
| Rv1274 | *lprB* | POSSIBLE LIPOPROTEIN LPRB | 27, 55, 69, 74, 86, 100, 127 |
| Rv1275 | *lprC* | POSSIBLE LIPOPROTEIN LPRC | 8, 12, 18, 27, 34, 89, 140, 164 |
| Rv1276c | *-* | hypothetical protein Rv1276c | 26, 29, 58, 63, 75, 112, 116, 137, 148 |
| Rv1277 | *-* | hypothetical protein Rv1277 | 7, 11, 52, 74, 85, 179, 185, 212, 240, 246, 261, 326, 335, 378, 388, 390, 396 |
| Rv1278 | *-* | hypothetical protein Rv1278 | 11, 29, 78, 108, 137, 141, 158, 159, 187, 191, 195, 203, 206, 252, 257, 272, 274, 277, 281, 285, 327, 363, 384, 405, 414, 420, 437, 447, 455, 459, 467, 495, 499, 515, 519, 539, 552, 568, 583, 602, 628, 667, 706, 714, 727, 793, 806, 860 |
| Rv1279 | *-* | PROBABLE DEHYDROGENASE FAD flavoprotein GMC oxidoreductase | 11, 14, 106, 109, 129, 136, 140, 143, 158, 164, 171, 192, 205, 216, 266, 311, 315, 326, 392, 415, 489, 521 |
| Rv1280c | *oppA* | PROBABLE PERIPLASMIC OLIGOPEPTIDE-BINDING LIPOPROTEIN OPPA | 49, 54, 68, 81, 148, 182, 194, 223, 238, 252, 267, 297, 311, 334, 391, 400, 422, 469, 471, 522, 533, 560, 583 |
| Rv1281c | *oppD* | PROBABLE OLIGOPEPTIDE-TRANSPORT ATP-BINDING PROTEIN ABC TRANSPORTER OPPD | 21, 24, 32, 47, 126, 203, 205, 229, 236, 262, 300, 323, 327, 395, 496, 500, 501, 535, 574 |
| Rv1282c | *oppC* | PROBABLE OLIGOPEPTIDE-TRANSPORT INTEGRAL MEMBRANE PROTEIN ABC TRANSPORTER OPPC | 109, 153, 165, 179, 207, 212, 219, 243, 245, 247, 256, 270 |
| Rv1283c | *oppB* | PROBABLE OLIGOPEPTIDE-TRANSPORT INTEGRAL MEMBRANE PROTEIN ABC TRANSPORTER OPPB | 82, 111, 169, 174, 177, 178, 202, 204, 225, 258, 262, 282, 299, 306 |
| Rv1284 | *-* | hypothetical protein Rv1284 | 15, 58, 108, 146, 154 |
| Rv1285 | *cysD* | sulfate adenylyltransferase subunit 2 | 10, 31, 33, 56, 85, 97, 113, 115, 130, 283, 308 |
| Rv1286 | *cysN* | bifunctional sulfate adenylyltransferase subunit 1/adenylylsulfate kinase protein | 100, 104, 179, 211, 267, 329, 347, 363, 394, 448, 451, 453, 472, 520, 576 |
| Rv1287 | *-* | hypothetical protein Rv1287 | 19, 58, 104, 130 |
| Rv1288 | *-* | hypothetical protein Rv1288 | 30, 33, 59, 81, 84, 110, 132, 181, 187, 200, 212, 231, 232, 243, 290, 317, 343, 379, 399, 411, 422, 444 |
| Rv1289 | *-* | hypothetical protein Rv1289 | 34, 122, 137 |
| Rv1290c | *-* | hypothetical protein Rv1290c | 29, 41, 50, 94, 172, 184, 194, 205, 300, 301, 313, 359, 395, 479, 490, 504, 515 |
| Rv1290A | *-* | hypothetical protein Rv1290A |  |
| Rv1291c | *-* | CONSERVED HYPOTHETICAL SECRETED PROTEIN | 6, 16, 22, 27, 29, 84, 98 |
| Rv1292 | *argS* | arginyl-tRNA synthetase | 2, 12, 14, 20, 25, 64, 83, 94, 107, 118, 128, 132, 138, 182, 259, 260, 273, 309, 321, 343, 345, 395, 442, 478, 496, 521, 544 |
| Rv1293 | *lysA* | PROBABLE DIAMINOPIMELATE DECARBOXYLASE LYSA (DAP DECARBOXYLASE) | 56, 69, 93, 123, 128, 151, 175, 188, 226, 256, 300, 305, 336, 344, 392, 399, 401, 418 |
| Rv1294 | *thrA* | homoserine dehydrogenase | 1, 54, 97, 113, 119, 148, 156, 173, 195, 203, 220, 239, 281, 296, 308, 312, 317, 323, 332, 368, 411, 438 |
| Rv1295 | *thrC* | threonine synthase | 16, 64, 92, 103, 106, 185, 197, 201, 227, 247, 273, 281, 296, 298, 325, 334 |
| Rv1296 | *thrB* | homoserine kinase | 47, 57, 84, 85, 99, 104, 110, 148, 192, 200, 243, 249, 266, 290, 314 |
| Rv1297 | *rho* | transcription termination factor Rho | 19, 33, 51, 133, 135, 142, 151, 177, 182, 194, 204, 208, 226, 241, 245, 269, 298, 460, 477, 484, 497, 512 |
| Rv1298 | *rpmE* | 50S ribosomal protein L31 | 15, 17, 45, 53, 77 |
| Rv1299 | *prfA* | peptide chain release factor 1 | 24, 55, 62, 74, 112, 119, 123, 179, 230, 233, 242, 275, 345 |
| Rv1300 | *hemK* | PROBABLE HEMK PROTEIN HOMOLOG HEMK | 2, 18, 50, 58, 67, 80, 123, 233, 242, 254, 290 |
| Rv1301 | *-* | hypothetical protein Rv1301 | 47, 50, 94, 178, 186 |
| Rv1302 | *rfe* | PROBABLE UNDECAPAPRENYL-PHOSPHATE ALPHA-N-ACETYLGLUCOSAMINYLTRANSFERASE RFE (UDP-GlcNAc TRANSFERASE) | 11, 14, 23, 25, 38, 47, 106, 109, 114, 137, 198, 199, 237, 258, 268, 273, 275, 319, 360, 371, 382, 393 |
| Rv1303 | *-* | hypothetical protein Rv1303 | 29, 36, 70, 104, 149 |
| Rv1304 | *atpB* | F0F1 ATP synthase subunit A | 6, 35, 57, 145, 150, 192, 193 |
| Rv1305 | *atpE* | F0F1 ATP synthase subunit C | 6, 11, 17, 23, 30, 36 |
| Rv1306 | *atpF* | F0F1 ATP synthase subunit B | 9, 17, 78, 127, 164 |
| Rv1307 | *atpH* | PROBABLE ATP SYNTHASE DELTA CHAIN ATPH | 30, 46, 202, 241, 372, 413, 417 |
| Rv1308 | *atpA* | F0F1 ATP synthase subunit alpha | 6, 22, 36, 59, 93, 120, 150, 163, 175, 178, 194, 216, 225, 236, 241, 242, 251, 291, 297, 320, 377, 389, 406, 414, 422, 451, 452, 495, 513, 515 |
| Rv1309 | *atpG* | F0F1 ATP synthase subunit gamma | 8, 41, 60, 76, 88, 109, 149, 174, 248 |
| Rv1310 | *atpD* | F0F1 ATP synthase subunit beta | 19, 36, 85, 92, 149, 163, 170, 172, 198, 223, 321, 354, 433 |
| Rv1311 | *atpC* | F0F1 ATP synthase subunit epsilon | 66, 92 |
| Rv1312 | *-* | CONSERVED HYPOTHETICAL SECRETED PROTEIN | 56, 60, 127 |
| Rv1313c | *-* | POSSIBLE TRANSPOSASE | 28, 51, 60, 105, 125, 184, 190, 211, 221, 279, 283, 371, 383, 426 |
| Rv1314c | *-* | hypothetical protein Rv1314c | 11, 43, 72, 141, 185 |
| Rv1315 | *murA* | UDP-N-acetylglucosamine 1-carboxyvinyltransferase | 19, 74, 114, 152, 172, 216, 235, 299, 371, 373, 396 |
| Rvnr01 | *rrs* | 16S rRNA |  |
| Rv1316c | *ogt* | PROBABLE METHYLATED-DNA--PROTEIN-CYSTEINE METHYLTRANSFERASE OGT (6-O-methylguanine-DNA methyltransferase) (O-6-methylguanine-DNA-alkyltransferase) | 16, 43, 58, 104, 106, 132, 136, 139, 157 |
| Rv1317c | *alkA* | PROBABLE ADA REGULATORY PROTEIN ALKA (Regulatory protein of adaptative response) (Methylated-DNA--protein-cysteine methyltransferase) (O-6-methylguanine-DNA alkyltransferase) (O-6-methylguanine-DNA methyltransferase) (3-methyladenine DNA glycosylase II) | 29, 55, 103, 126, 139, 157, 158, 186, 193, 219, 221, 306, 333, 343, 371, 400, 408, 415, 421, 428, 446 |
| Rv1318c | *-* | POSSIBLE ADENYLATE CYCLASE (ATP PYROPHOSPHATE-LYASE) (ADENYLYL CYCLASE) | 21, 28, 54, 59, 178, 184, 198, 204, 321, 342, 344, 353, 430, 448, 459, 491, 503 |
| Rv1319c | *-* | POSSIBLE ADENYLATE CYCLASE (ATP PYROPHOSPHATE-LYASE) (ADENYLYL CYCLASE) | 21, 63, 153, 179, 199, 233, 343, 345, 354, 360, 379, 411, 428, 431, 449, 460, 492, 522 |
| Rv1320c | *-* | POSSIBLE ADENYLATE CYCLASE (ATP PYROPHOSPHATE-LYASE) (ADENYLYL CYCLASE) | 28, 151, 198, 237, 298, 342, 353, 427, 431, 448, 459, 502, 521, 547 |
| Rv1321 | *-* | hypothetical protein Rv1321 | 19, 24, 62, 63, 75, 98, 133, 187, 202, 215, 219 |
| Rv1322 | *-* | hypothetical protein Rv1322 | 56, 71, 90 |
| Rv1322A | *-* | hypothetical protein Rv1322A | 66, 89, 126, 139, 140 |
| Rv1323 | *fadA4* | acetyl-CoA acetyltransferase | 53, 56, 60, 68, 85, 107, 108, 220, 222, 225, 238, 239, 250, 278, 374, 377, 387 |
| Rv1324 | *-* | POSSIBLE THIOREDOXIN | 12, 18, 42, 85, 88, 90, 137, 153, 186, 218, 237, 286, 293 |
| Rv1325c | *PE_PGRS24* | PE-PGRS FAMILY PROTEIN | 5, 13, 26, 29, 41, 55, 91, 93, 127, 130, 131, 133, 134, 141, 143, 144, 146, 149, 152, 153, 165, 170, 181, 183, 184, 186, 187, 193, 194, 196, 199, 200, 208, 211, 214, 217, 222, 226, 228, 229, 237, 238, 240, 241, 244, 251, 252, 253, 255, 256, 258, 259, 267, 270, 271, 273, 274, 276, 283, 285, 286, 288, 289, 294, 297, 301, 303, 307, 311, 314, 316, 317, 319, 320, 324, 330, 333, 335, 336, 339, 342, 349, 352, 354, 355, 357, 364, 365, 367, 368, 374, 377, 379, 385, 386, 388, 391, 403, 404, 407, 409, 412, 415, 422, 424, 425, 427, 428, 438, 441, 442, 444, 445, 451, 456, 457, 469, 470, 472, 473, 476, 477, 483, 485, 486, 488, 489, 494, 495, 501, 504, 511, 513, 514, 516, 517, 522, 528, 534, 540, 543, 545, 546, 554, 557, 559, 560, 572, 575, 586, 587, 589, 590, 599 |
| Rv1326c | *glgB* | glycogen branching enzyme | 7, 224, 300, 333, 369, 425, 433, 478, 552, 553, 566, 587, 638, 699, 714, 729 |
| Rv1327c | *glgE* | PROBABLE GLUCANASE GLGE | 14, 28, 32, 99, 142, 165, 169, 178, 185, 192, 241, 242, 257, 266, 294, 311, 331, 451, 523, 534, 617, 659 |
| Rv1328 | *glgP* | PROBABLE GLYCOGEN PHOSPHORYLASE GLGP | 17, 49, 101, 104, 110, 130, 136, 142, 159, 167, 259, 262, 271, 289, 310, 328, 329, 347, 442, 622, 662, 683, 728, 761, 782, 800, 815, 817, 820, 851 |
| Rv1329c | *dinG* | PROBABLE ATP-DEPENDENT HELICASE DING | 20, 25, 31, 40, 48, 50, 52, 61, 148, 158, 174, 212, 258, 270, 332, 355, 365, 420, 458, 469, 483, 498, 530, 580, 583, 591, 603, 625 |
| Rv1330c | *-* | nicotinate phosphoribosyltransferase | 5, 19, 55, 63, 65, 103, 117, 125, 158, 159, 176, 187, 190, 204, 208, 221, 230, 241, 254, 261, 263, 274, 301, 310, 313, 328, 334, 359, 360, 403, 413, 421, 435 |
| Rv1331 | *clpS* | ATP-dependent Clp protease adaptor protein ClpS | 6, 73 |
| Rv1332 | *-* | PROBABLE TRANSCRIPTIONAL REGULATORY PROTEIN | 11, 75, 79, 136, 148, 163, 193 |
| Rv1333 | *-* | PROBABLE HYDROLASE | 25, 38, 47, 74, 75, 82, 96, 102, 119, 137, 154, 159, 170, 182, 191, 241, 254, 280, 289, 293, 303, 319, 328, 338 |
| Rv1334 | *-* | hypothetical protein Rv1334 | 33, 37, 65 |
| Rv1335 | *-* | 9.5 KDA CULTURE FILTRATE ANTIGEN CFP10A | 14, 15, 23, 70, 71, 87, 90 |
| Rv1336 | *cysM* | PROBABLE CYSTEINE SYNTHASE B CYSM (CSASE B) (O-acetylserine sulfhydrylase B) (O-acetylserine (Thiol)-lyase B) | 46, 78, 81, 126, 134, 139, 154, 162, 164, 180, 236, 265, 277, 282, 295, 302, 306, 314 |
| Rv1337 | *-* | PROBABLE INTEGRAL MEMBRANE PROTEIN | 38, 59, 109, 118, 125, 182, 218, 230, 234 |
| Rv1338 | *murI* | glutamate racemase | 12, 38, 106, 140, 153, 157, 191, 211, 250 |
| Rv1339 | *-* | hypothetical protein Rv1339 | 25, 53, 66, 170, 177, 180, 191, 219, 223, 226, 271 |
| Rv1340 | *rph* | ribonuclease PH | 19, 41, 55, 61, 85, 128, 132, 162, 196, 205, 232 |
| Rv1341 | *-* | putative deoxyribonucleotide triphosphate pyrophosphatase | 26, 48, 65, 67, 76, 81, 84, 95, 102, 105, 120, 134, 140, 144, 152, 155, 186 |
| Rv1342c | *-* | hypothetical protein Rv1342c | 1, 6, 22, 90, 95 |
| Rv1343c | *lprD* | PROBABLE CONSERVED LIPOPROTEIN LPRD | 8, 74, 75, 77, 83, 124 |
| Rv1344 | *-* | acyl carrier protein |  |
| Rv1345 | *fadD33* | acyl-CoA synthetase | 15, 53, 68, 76, 84, 127, 152, 158, 167, 216, 247, 301, 322, 335, 348, 356, 374, 396, 405, 437, 449, 456, 465, 484 |
| Rv1346 | *fadE14* | POSSIBLE ACYL-CoA DEHYDROGENASE FADE14 | 2, 10, 25, 45, 76, 112, 123, 124, 145, 185, 204, 222, 241, 313, 354, 367 |
| Rv1347c | *-* | hypothetical protein Rv1347c | 5, 173, 182 |
| Rv1348 | *-* | PROBABLE DRUGS-TRANSPORT TRANSMEMBRANE ATP-BINDING PROTEIN ABC TRANSPORTER | 2, 18, 49, 78, 127, 128, 211, 239, 322, 324, 335, 342, 348, 384, 386, 413, 454, 487, 511, 531, 533, 570, 638, 644, 646, 698, 711, 747, 772, 823, 831, 833, 842, 851 |
| Rv1349 | *-* | PROBABLE DRUGS-TRANSPORT TRANSMEMBRANE ATP-BINDING PROTEIN ABC TRANSPORTER | 60, 65, 73, 105, 109, 119, 161, 173, 182, 189, 207, 227, 255, 258, 287, 311, 317, 342, 344, 369, 386, 465, 470, 471, 486, 505, 555 |
| Rv1350 | *fabG* | 3-ketoacyl-(acyl-carrier-protein) reductase | 12, 13, 51, 75, 81, 90, 159, 168, 212, 234, 242 |
| Rv1351 | *-* | hypothetical protein Rv1351 | 45, 60 |
| Rv1352 | *-* | hypothetical protein Rv1352 | 19, 31, 36 |
| Rv1353c | *-* | PROBABLE TRANSCRIPTIONAL REGULATORY PROTEIN | 12, 22, 83, 158, 175, 178, 188, 233, 245 |
| Rv1354c | *-* | hypothetical protein Rv1354c | 4, 119, 127, 174, 189, 207, 233, 234, 260, 268, 276, 295, 312, 327, 334, 335, 343, 349, 381, 437, 457, 492, 514, 551, 574, 596, 605 |
| Rv1355c | *moeY* | hypothetical protein Rv1355c | 6, 9, 96, 115, 124, 181, 214, 229, 334, 348, 431, 484, 489, 498, 564, 587, 621, 663 |
| Rv1356c | *-* | hypothetical protein Rv1356c | 3, 14, 47, 90, 124, 127, 146, 161, 182, 254, 257 |
| Rv1357c | *-* | hypothetical protein Rv1357c | 66, 150, 157, 170, 173, 201, 279, 289 |
| Rv1358 | *-* | PROBABLE TRANSCRIPTIONAL REGULATORY PROTEIN | 3, 66, 122, 134, 169, 175, 181, 183, 204, 292, 295, 308, 327, 333, 402, 460, 484, 524, 529, 535, 537, 538, 613, 634, 647, 653, 742, 750, 839, 869, 885, 902, 939, 957, 987, 991, 1012, 1015, 1028, 1045, 1049, 1065, 1071, 1084, 1086, 1120, 1153 |
| Rv1359 | *-* | PROBABLE TRANSCRIPTIONAL REGULATORY PROTEIN | 61, 74, 77, 92, 123, 135, 218 |
| Rv1360 | *-* | PROBABLE OXIDOREDUCTASE | 121, 167, 177, 236, 304, 324, 332 |
| Rv1361c | *PPE19* | PPE FAMILY PROTEIN | 12, 17, 26, 41, 79, 86, 148, 155, 177, 178, 193, 278, 308, 327, 348, 351, 353, 358, 368, 370, 373, 392, 394 |
| Rv1362c | *-* | POSSIBLE MEMBRANE PROTEIN | 23, 27, 44, 95, 97, 103, 126, 132, 159 |
| Rv1363c | *-* | POSSIBLE MEMBRANE PROTEIN | 21, 26, 33, 42, 81, 86, 110, 116, 119, 122, 139, 143, 144, 173, 214, 228 |
| Rv1364c | *-* | hypothetical protein Rv1364c | 45, 57, 80, 98, 166, 173, 178, 184, 188, 190, 191, 218, 278, 287, 298, 305, 307, 333, 341, 353, 356, 382, 465, 468, 495, 513, 514, 555, 568, 600, 607, 625 |
| Rv1365c | *rsfA* | ANTI-ANTI-SIGMA FACTOR RSFA (ANTI-SIGMA FACTOR ANTAGONIST) (REGULATOR OF SIGMA F A) | 37, 51, 55, 108, 122 |
| Rv1366 | *-* | hypothetical protein Rv1366 | 54, 131, 142, 155, 196, 233 |
| Rv1367c | *-* | hypothetical protein Rv1367c | 75, 76, 84, 99, 182, 194, 224, 225, 231, 285, 307, 315, 347, 361 |
| Rv1368 | *lprF* | PROBABLE CONSERVED LIPOPROTEIN LPRF | 8, 38, 50, 136, 188, 193, 226 |
| Rv1369c | *-* | PROBABLE TRANSPOSASE | 50, 87, 91, 107, 241, 255, 271, 291, 292 |
| Rv1370c | *-* | PROBABLE TRANSPOSASE | 24, 60, 64, 71, 93 |
| Rv1371 | *-* | PROBABLE CONSERVED MEMBRANE PROTEIN | 12, 16, 21, 46, 47, 61, 139, 191, 224, 272, 299, 308, 324, 357, 390, 406, 430 |
| Rv1372 | *-* | hypothetical protein Rv1372 | 6, 12, 51, 62, 125, 131, 143, 175, 183, 281, 286, 329 |
| Rv1373 | *-* | GLYCOLIPID SULFOTRANSFERASE | 40, 60, 120, 237, 281 |
| Rv1374c | *-* | hypothetical protein Rv1374c | 41 |
| Rv1375 | *-* | hypothetical protein Rv1375 | 1, 9, 84, 117, 148, 160, 173, 187, 218, 222, 248, 261, 275, 294, 299, 307, 326, 335, 396 |
| Rv1376 | *-* | hypothetical protein Rv1376 | 3, 9, 16, 161, 185, 188, 243, 294, 301, 328, 334, 456, 479 |
| Rv1377c | *-* | PUTATIVE TRANSFERASE | 14, 40, 41, 53, 70, 77, 81, 89, 104, 107, 116, 131, 185, 209 |
| Rv1378c | *-* | hypothetical protein Rv1378c | 30, 65, 79, 80, 107, 125, 145, 184, 195, 214, 254, 277, 280, 363, 394, 398, 416, 427, 438, 445, 450, 473 |
| Rv1379 | *pyrR* | pyrimidine regulatory protein PyrR | 6, 17, 36, 66, 73, 106, 123, 129 |
| Rv1380 | *pyrB* | aspartate carbamoyltransferase catalytic subunit | 7, 17, 78, 91, 98, 108, 110, 112, 120, 132, 155, 202, 217, 234, 258, 312 |
| Rv1381 | *pyrC* | dihydroorotase | 44, 50, 72, 75, 77, 80, 83, 107, 127, 139, 141, 168, 222, 251, 317, 321, 340, 389, 408, 412, 417, 421 |
| Rv1382 | *-* | PROBABLE EXPORT OR MEMBRANE PROTEIN | 2, 28, 59, 113, 119, 122, 127, 140 |
| Rv1383 | *carA* | carbamoyl phosphate synthase small subunit | 14, 21, 32, 35, 54, 61, 78, 95, 111, 125, 160, 165, 185, 202, 232, 237, 251, 281, 292, 298, 320, 352, 354 |
| Rv1384 | *carB* | carbamoyl phosphate synthase large subunit | 14, 28, 98, 102, 109, 129, 143, 147, 179, 196, 198, 248, 283, 284, 319, 327, 371, 410, 420, 457, 494, 509, 512, 552, 617, 641, 643, 649, 670, 688, 697, 698, 707, 736, 789, 798, 802, 899, 929, 942, 961, 1005, 1028, 1041, 1055, 1070, 1083, 1091, 1094 |
| Rv1385 | *pyrF* | orotidine 5'-phosphate decarboxylase | 1, 14, 41, 70, 95, 116, 139, 162, 186, 188, 209, 221, 228, 231, 233, 240, 249, 251, 258 |
| Rv1386 | *PE15* | PE FAMILY PROTEIN | 20, 24, 28, 64, 86, 98 |
| Rv1387 | *PPE20* | PPE FAMILY PROTEIN | 28, 68, 88, 100, 112, 148, 164, 185, 197, 202, 244, 326, 337, 348, 356, 363, 369, 373, 379, 382, 384, 402, 404, 420, 425, 510, 515, 524 |
| Rv1388 | *mihF* | PUTATIVE INTEGRATION HOST FACTOR MIHF | 97, 104, 119, 188 |
| Rv1389 | *gmk* | guanylate kinase | 11, 26, 29, 93, 108, 122, 154, 168 |
| Rv1390 | *rpoZ* | DNA-directed RNA polymerase subunit omega | 10, 13 |
| Rv1391 | *dfp* | bifunctional phosphopantothenoylcysteine decarboxylase/phosphopantothenate synthase | 15, 19, 44, 51, 58, 91, 100, 108, 144, 151, 157, 161, 198, 199, 217, 228, 231, 242, 248, 249, 272, 287, 292, 315, 339 |
| Rv1392 | *metK* | S-adenosylmethionine synthetase | 49, 94, 113, 131, 247, 253, 259, 262, 272, 278, 283, 307, 308, 357, 374 |
| Rv1393c | *-* | PROBABLE MONOXYGENASE | 11, 14, 18, 35, 40, 49, 76, 105, 122, 134, 158, 168, 177, 224, 225, 290, 337, 353, 356, 378, 418 |
| Rv1394c | *cyp132* | PROBABLE CYTOCHROME P450 132 CYP132 | 2, 32, 41, 87, 88, 104, 117, 131, 136, 176, 243, 255, 273, 278, 313, 324, 357, 403 |
| Rv1395 | *-* | PROBABLE TRANSCRIPTIONAL REGULATORY PROTEIN | 26, 30, 58, 71, 124, 151, 165, 255, 342 |
| Rv1396c | *PE_PGRS25* | PE-PGRS FAMILY PROTEIN | 31, 33, 40, 41, 47, 55, 91, 125, 128, 129, 131, 132, 139, 142, 144, 147, 150, 151, 154, 157, 163, 165, 166, 171, 174, 177, 178, 180, 181, 188, 190, 191, 194, 199, 202, 206, 208, 209, 211, 212, 214, 215, 220, 223, 224, 226, 227, 234, 237, 240, 246, 248, 250, 259, 261, 262, 265, 268, 270, 278, 280, 281, 283, 284, 288, 293, 296, 297, 299, 318, 320, 321, 323, 324, 330, 332, 333, 336, 342, 344, 345, 347, 357, 359, 360, 363, 370, 373, 376, 377, 387, 389, 393, 403, 406, 408, 409, 411, 412, 418, 420, 421, 423, 424, 435, 440, 441, 443, 447, 453, 454, 456, 457, 459, 460, 463, 473, 475, 476, 478, 479, 482, 484, 485, 493, 494, 496, 497, 500, 502, 503, 508, 509, 512, 514, 515, 520, 527, 529, 532, 533, 538, 544, 547, 549, 553, 555, 563, 566, 569, 572, 574 |
| Rv1397c | *-* | hypothetical protein Rv1397c | 38, 43, 47, 73, 97 |
| Rv1398c | *-* | hypothetical protein Rv1398c | 19, 40, 69 |
| Rv1399c | *lipH* | PROBABLE LIPASE LIPH | 26, 89, 94, 129, 145, 149, 158, 163, 178, 246, 250, 255, 274, 299, 305, 307 |
| Rv1400c | *lipI* | PROBABLE LIPASE LIPH | 6, 11, 91, 131, 139, 161, 164, 165, 166, 170, 219, 240, 257, 274, 276, 296, 300 |
| Rv1401 | *-* | POSSIBLE MEMBRANE PROTEIN | 3, 14, 16, 38, 94, 127, 138, 146, 173, 181, 193 |
| Rv1402 | *priA* | primosome assembly protein PriA | 15, 83, 95, 127, 133, 161, 167, 187, 202, 215, 279, 293, 319, 328, 331, 341, 348, 359, 388, 391, 442, 452, 468, 469, 502, 508, 511, 562, 592, 601, 627 |
| Rv1403c | *-* | PUTATIVE METHYLTRANSFERASE | 26, 44, 48, 56, 93, 111, 142, 269 |
| Rv1404 | *-* | PROBABLE TRANSCRIPTIONAL REGULATORY PROTEIN | 75, 149 |
| Rv1405c | *-* | PUTATIVE METHYLTRANSFERASE | 42, 56, 65, 90, 131, 143, 212, 241, 269 |
| Rv1406 | *fmt* | methionyl-tRNA formyltransferase | 5, 10, 35, 37, 55, 92, 122, 125, 131, 160, 231, 251, 261, 275, 288, 307 |
| Rv1407 | *fmu* | PROBABLE FMU PROTEIN (SUN PROTEIN) | 38, 44, 47, 51, 65, 75, 107, 116, 136, 159, 203, 207, 208, 216, 231, 268, 276, 279, 282, 286, 297, 314, 344, 377, 384, 385, 449 |
| Rv1408 | *rpe* | ribulose-phosphate 3-epimerase | 4, 18, 29, 116, 181, 194, 201, 212, 222, 224 |
| Rv1409 | *ribG* | PROBABLE BIFUNCTIONAL riboflavin biosynthesis protein RIBG : Diaminohydroxyphosphoribosylaminopyrimidine deaminase (Riboflavin-specific deaminase) + 5-amino-6-(5-phosphoribosylamino) uracil reductase (HTP reductase) | 46, 47, 52, 65, 66, 70, 129, 138, 150, 168, 181, 200, 211, 277, 299, 303 |
| Rv1410c | *-* | AMINOGLYCOSIDES/TETRACYCLINE-TRANSPORT INTEGRAL MEMBRANE PROTEIN | 59, 82, 91, 94, 114, 135, 139, 143, 177, 244, 263, 272, 279, 280, 307, 324, 331, 339, 418, 425, 457, 478 |
| Rv1411c | *lprG* | PROBABLE CONSERVED LIPOPROTEIN LPRG | 14, 19, 24, 33, 48, 76, 84, 124, 131, 139, 168, 198, 210 |
| Rv1412 | *ribC* | riboflavin synthase subunit alpha | 2, 8, 12, 26, 31, 34, 59, 77, 86, 94, 105, 148, 174, 199 |
| Rv1413 | *-* | hypothetical protein Rv1413 | 48, 55, 62, 79, 107, 116, 119, 145, 151, 155 |
| Rv1414 | *-* | hypothetical protein Rv1414 | 22, 33, 42, 126, 128 |
| Rv1415 | *ribA2* | bifunctional 3,4-dihydroxy-2-butanone 4-phosphate synthase/GTP cyclohydrolase II protein | 36, 53, 124, 141, 146, 154, 158, 242, 246, 265, 276, 282, 292, 304, 320, 417, 421 |
| Rv1416 | *ribH* | riboflavin synthase subunit beta | 36, 37, 77, 142 |
| Rv1417 | *-* | POSSIBLE CONSERVED MEMBRANE PROTEIN | 1, 49, 63, 79, 103, 104, 134 |
| Rv1418 | *lprH* | PROBABLE LIPOPROTEIN LPRH | 43, 48, 115, 157, 208 |
| Rv1419 | *-* | hypothetical protein Rv1419 | 7, 27, 85, 130 |
| Rv1420 | *uvrC* | excinuclease ABC subunit C | 3, 8, 10, 62, 120, 179, 203, 219, 237, 276, 281, 297, 319, 323, 348, 351, 362, 395, 505, 619, 622, 643 |
| Rv1421 | *-* | hypothetical protein Rv1421 | 13, 22, 25, 27, 29, 61, 62, 78, 81, 146, 170, 173, 216, 265 |
| Rv1422 | *-* | hypothetical protein Rv1422 | 8, 28, 81, 87, 138, 160, 193, 210, 234, 250 |
| Rv1423 | *whiA* | PROBABLE TRANSCRIPTIONAL REGULATORY PROTEIN WHIA | 30, 31, 37, 75, 98, 104, 117, 176, 235, 240, 261, 290 |
| Rv1424c | *-* | POSSIBLE MEMBRANE PROTEIN | 4, 65, 67, 166, 191, 217, 223 |
| Rv1425 | *-* | hypothetical protein Rv1425 | 12, 15, 60, 91, 126, 144, 146, 214, 239, 268, 282, 311, 343, 362, 369, 386, 395, 456 |
| Rv1426c | *lipO* | PROBABLE ESTERASE LIPO | 21, 29, 31, 63, 94, 138, 184, 185, 207, 243, 246, 255, 259, 260, 278, 400 |
| Rv1427c | *fadD12* | acyl-CoA synthetase | 31, 45, 55, 72, 77, 85, 99, 123, 179, 182, 189, 200, 203, 213, 311, 343, 348, 356, 360, 364, 382, 403, 409, 438, 439, 461, 483, 517, 520 |
| Rv1428c | *-* | hypothetical protein Rv1428c | 62, 100, 107, 123, 131, 132, 183, 213, 223, 265 |
| Rv1429 | *-* | hypothetical protein Rv1429 | 5, 76, 83, 92, 118, 132, 159, 194, 220, 249, 275, 301, 306, 307, 389, 398 |
| Rv1430 | *PE16* | PE FAMILY PROTEIN | 13, 28, 40, 42, 81, 91, 94, 99, 102, 120, 148, 153, 160, 175, 180, 188, 216, 344, 370, 379, 404, 429, 436, 438, 443, 464, 483, 484, 488, 509, 512, 521 |
| Rv1431 | *-* | hypothetical protein Rv1431 | 55, 56, 58, 65, 68, 100, 102, 106, 156, 164, 175, 177, 208, 210, 308, 400, 406, 456, 491, 548, 553, 570, 582 |
| Rv1432 | *-* | PROBABLE DEHYDROGENASE | 2, 8, 14, 36, 41, 47, 94, 105, 107, 124, 125, 160, 176, 178, 191, 201, 212, 214, 222, 229, 234, 257, 280, 311, 366, 370, 382, 408, 413, 445, 448, 450, 460 |
| Rv1433 | *-* | POSSIBLE CONSERVED EXPORTED PROTEIN | 44, 81, 84, 124, 222 |
| Rv1434 | *-* | hypothetical protein Rv1434 | 4, 43 |
| Rv1435c | *-* | Probable conserved Proline, Glycine, Valine-rich secreted protein | 15, 53, 54, 60, 65, 71, 116, 117, 130, 154, 167, 180, 190, 197 |
| Rv1436 | *gap* | glyceraldehyde-3-phosphate dehydrogenase | 28, 35, 103, 118, 128, 205, 215, 243, 268 |
| Rv1437 | *pgk* | phosphoglycerate kinase | 60, 110, 111, 177, 204, 228, 257, 280, 317, 336, 347, 349, 358, 362, 364, 381, 382, 396, 407 |
| Rv1438 | *tpiA* | triosephosphate isomerase | 7, 75, 83, 114, 117, 119, 156, 178, 184, 215, 238, 255 |
| Rv1439c | *-* | hypothetical protein Rv1439c |  |
| Rv1440 | *secG* | preprotein translocase subunit SecG | 27, 34, 42, 59 |
| Rv1441c | *PE_PGRS26* | PE-PGRS FAMILY PROTEIN | 30, 40, 41, 80, 87, 91, 129, 135, 136, 143, 146, 148, 151, 156, 159, 162, 168, 170, 171, 173, 179, 181, 184, 187, 188, 190, 193, 198, 201, 203, 204, 207, 212, 216, 218, 224, 228, 233, 236, 245, 246, 248, 251, 252, 255, 257, 258, 260, 266, 273, 276, 292, 294, 295, 298, 300, 307, 314, 317, 320, 322, 323, 325, 329, 335, 339, 341, 342, 344, 345, 348, 350, 351, 353, 354, 364, 366, 367, 370, 375, 376, 389, 391, 392, 395, 398, 400, 401, 403, 404, 416, 419, 422, 426, 431, 433, 435, 437, 438, 441, 443, 444, 447, 450, 456, 459, 462, 467, 468, 471, 480 |
| Rv1442 | *bisC* | PROBABLE BIOTIN SULFOXIDE REDUCTASE BISC (BDS reductase) (BSO reductase) | 13, 18, 25, 41, 53, 57, 70, 113, 131, 134, 142, 174, 181, 188, 192, 193, 196, 210, 225, 228, 272, 295, 299, 302, 303, 339, 352, 372, 400, 422, 454, 476, 505, 551, 560, 581, 600, 613, 617, 640, 661, 678, 700, 706, 728, 738 |
| Rv1443c | *-* | hypothetical protein Rv1443c | 16, 46, 129, 134 |
| Rv1444c | *-* | hypothetical protein Rv1444c | 7, 10, 23, 119 |
| Rv1445c | *devB* | 6-phosphogluconolactonase | 40, 42, 105, 112, 117, 127, 130, 160, 201, 210, 214, 224, 240 |
| Rv1446c | *opcA* | PUTATIVE OXPP CYCLE PROTEIN OPCA | 10, 87, 89, 98, 102, 162, 166, 201, 213, 272, 291 |
| Rv1447c | *zwf2* | glucose-6-phosphate 1-dehydrogenase | 5, 25, 28, 36, 54, 131, 133, 134, 158, 248, 275, 311, 315, 316, 320, 343, 355, 420, 506 |
| Rv1448c | *tal* | transaldolase | 1, 8, 12, 69, 71, 148, 151, 183, 191, 229, 232, 272, 317, 369 |
| Rv1449c | *tkt* | transketolase | 7, 33, 82, 96, 116, 125, 131, 152, 191, 222, 238, 278, 292, 322, 385, 396, 426, 448, 460, 542, 567, 575, 591, 647, 672, 690, 692 |
| Rv1450c | *PE_PGRS27* | PE-PGRS FAMILY PROTEIN | 12, 40, 81, 94, 129, 131, 134, 135, 137, 138, 147, 148, 153, 157, 159, 160, 162, 163, 168, 169, 171, 174, 177, 179, 180, 183, 186, 197, 199, 200, 206, 209, 210, 212, 213, 216, 222, 224, 225, 227, 228, 230, 231, 233, 234, 237, 239, 242, 246, 252, 253, 258, 259, 261, 271, 277, 283, 288, 289, 297, 300, 310, 314, 317, 320, 322, 326, 330, 332, 335, 336, 338, 342, 344, 345, 347, 348, 350, 363, 368, 369, 371, 374, 375, 377, 387, 393, 397, 399, 400, 409, 412, 413, 416, 418, 419, 421, 422, 428, 430, 433, 434, 439, 445, 450, 452, 456, 459, 462, 465, 472, 475, 477, 478, 481, 484, 489, 492, 506, 509, 514, 515, 517, 520, 524, 539, 540, 542, 543, 546, 548, 549, 552, 555, 566, 574, 577, 580, 589, 591, 594, 601, 607, 611, 613, 614, 623, 624, 626, 627, 629, 630, 633, 636, 642, 644, 645, 648, 653, 656, 662, 666, 669, 673, 676, 679, 681, 682, 685, 692, 694, 697, 698, 714, 717, 720, 722, 726, 732, 734, 735, 753, 754, 759, 760, 763, 766, 781, 784, 787, 790, 792, 793, 796, 798, 801, 817, 821, 824, 827, 834, 837, 840, 843, 846, 849, 852, 855, 857, 882, 883, 888, 889, 891, 892, 894, 898, 903, 904, 907, 910, 913, 917, 918, 928, 931, 932, 935, 938, 940, 941, 944, 946, 947, 950, 960, 962, 965, 966, 969, 972, 978, 988, 989, 991, 995, 998, 1001, 1004, 1006, 1007, 1009, 1010, 1012, 1022, 1029, 1032, 1035, 1041, 1045, 1047, 1048, 1054, 1057, 1059, 1063, 1069, 1072, 1074, 1077, 1090, 1093, 1096, 1099, 1102, 1105, 1110, 1112, 1113, 1118, 1119, 1122, 1125, 1143, 1145, 1148, 1154, 1161, 1170, 1175, 1176, 1178, 1179, 1181, 1182, 1184, 1185, 1191, 1194, 1195, 1198, 1201, 1204, 1209, 1210, 1212, 1213, 1216, 1218, 1219, 1221, 1237, 1240, 1245, 1251, 1256, 1259, 1260, 1262, 1272, 1275, 1282, 1284, 1294, 1297, 1309, 1310, 1311, 1313, 1315, 1320, 1323 |
| Rv1451 | *ctaB* | protoheme IX farnesyltransferase | 3, 37, 47, 61, 65, 67, 87, 158, 171, 234, 254, 267, 271 |
| Rv1452c | *PE_PGRS28* | PE-PGRS FAMILY PROTEIN | 11, 40, 81, 94, 129, 131, 134, 135, 137, 138, 147, 148, 153, 157, 159, 160, 162, 163, 168, 169, 171, 174, 177, 179, 180, 183, 186, 197, 199, 200, 206, 209, 210, 212, 213, 216, 222, 224, 225, 227, 228, 230, 231, 233, 234, 237, 239, 242, 246, 252, 253, 258, 259, 261, 271, 277, 283, 288, 289, 297, 300, 310, 314, 317, 320, 322, 326, 330, 332, 335, 336, 338, 342, 344, 345, 347, 348, 350, 363, 369, 372, 375, 378, 380, 381, 383, 384, 391, 395, 396, 398, 399, 401, 402, 408, 411, 413, 416, 425, 426, 428, 429, 432, 435, 437, 438, 440, 443, 444, 446, 450, 462, 466, 468, 469, 477, 485, 487, 488, 491, 497, 499, 500, 507, 524, 528, 531, 533, 534, 537, 541, 544, 546, 550, 552, 570, 581, 587, 589, 590, 596, 600, 603, 606, 612, 615, 621, 628, 639, 643, 646, 655, 656, 658, 662, 664, 665, 671, 674, 682, 684, 689, 690, 693, 695, 708, 710, 722, 729, 731, 734 |
| Rv1453 | *-* | POSSIBLE TRANSCRIPTIONAL ACTIVATOR PROTEIN | 37, 46, 65, 71, 76, 200, 226, 227, 237, 239, 250, 274, 291, 358, 375 |
| Rv1454c | *qor* | PROBABLE QUINONE REDUCTASE QOR (NADPH:quinone reductase) (Zeta-crystallin homolog protein) | 8, 9, 24, 47, 63, 74, 78, 106, 150, 151, 184, 200, 207, 208, 210, 234, 242, 244, 257, 258, 274, 282, 299 |
| Rv1455 | *-* | hypothetical protein Rv1455 | 21, 22, 37, 66, 110, 136, 156, 164, 167, 177, 206, 209, 212 |
| Rv1456c | *-* | PROBABLE UNIDENTIFIED ANTIBIOTIC-TRANSPORT INTEGRAL MEMBRANE ABC TRANSPORTER | 17, 25, 30, 37, 83, 124, 180, 261, 270, 273, 282, 284, 291 |
| Rv1457c | *-* | PROBABLE UNIDENTIFIED ANTIBIOTIC-TRANSPORT INTEGRAL MEMBRANE ABC TRANSPORTER | 6, 9, 27, 71, 86, 89, 119, 156, 163, 174, 194, 249, 252 |
| Rv1458c | *-* | PROBABLE UNIDENTIFIED ANTIBIOTIC-TRANSPORT ATP-BINDING PROTEIN ABC TRANSPORTER | 14, 24, 64, 92, 100, 111, 129, 138, 139, 164, 216, 226, 235, 278, 306 |
| Rv1459c | *-* | POSSIBLE CONSERVED INTEGRAL MEMBRANE PROTEIN | 31, 42, 54, 86, 110, 158, 190, 252, 266, 273, 292, 369, 388, 400, 418, 432, 459, 466, 495, 520, 535, 565 |
| Rv1460 | *-* | PROBABLE TRANSCRIPTIONAL REGULATORY PROTEIN | 34, 53, 57, 58, 69, 70, 90, 98, 101, 108, 110, 134, 163, 179, 189, 258 |
| Rv1461 | *-* | hypothetical protein Rv1461 | 47, 58, 109, 141, 188, 211, 254, 255, 261, 279, 289, 315, 537, 560, 562, 612, 660, 685, 699, 707, 729, 731, 801, 814 |
| Rv1462 | *-* | hypothetical protein Rv1462 | 53, 57, 77, 85, 93, 102, 123, 153, 168, 172, 207, 220, 289, 318, 324, 330, 344, 357, 377 |
| Rv1463 | *-* | PROBABLE CONSERVED ATP-BINDING PROTEIN ABC TRANSPORTER | 15, 26, 34, 86, 99, 112, 115, 133, 151, 178, 201, 226, 232, 251, 253, 260, 263 |
| Rv1464 | *csd* | PROBABLE CYSTEINE DESULFURASE CSD | 34, 53, 120, 145, 183, 226, 229, 257, 258, 281, 293, 305, 316, 323, 362, 383, 385, 395, 404 |
| Rv1465 | *-* | POSSIBLE NITROGEN FIXATION RELATED PROTEIN | 39, 71, 102, 119 |
| Rv1466 | *-* | hypothetical protein Rv1466 |  |
| Rv1467c | *fadE15* | PROBABLE ACYL-CoA DEHYDROGENASE FADE15 | 26, 89, 155, 168, 197, 204, 219, 252, 271, 276, 283, 307, 308, 371, 422, 496, 509, 514, 549, 554, 587 |
| Rv1468c | *PE_PGRS29* | PE-PGRS FAMILY PROTEIN | 30, 36, 40, 68, 80, 81, 87, 124, 132, 133, 140, 146, 148, 150, 151, 154, 156, 157, 163, 166, 175, 177, 178, 180, 181, 188, 190, 191, 194, 197, 200, 201, 204, 206, 212, 213, 225, 232, 239, 248, 252, 253, 264, 265, 270, 277, 280, 287, 289, 290, 292, 294, 302, 304, 305, 308, 314, 317, 320, 324, 327, 339, 342, 345, 348, 351, 354, 357, 364 |
| Rv1469 | *ctpD* | PROBABLE CATION TRANSPORTER P-TYPE ATPASE D CTPD | 3, 9, 29, 49, 73, 74, 99, 123, 134, 199, 209, 215, 330, 348, 380, 406, 416, 436, 449, 453, 487, 509, 532, 548, 551, 555, 561, 569, 650 |
| Rv1470 | *trxA* | PROBABLE THIOREDOXIN TRXA | 15, 66, 87, 92, 111 |
| Rv1471 | *trxB1* | PROBABLE THIOREDOXIN TRXB1 | 7, 29, 65, 87, 109 |
| Rv1472 | *echA12* | enoyl-CoA hydratase | 10, 69, 122, 127, 152, 153, 160, 185, 217, 241, 262, 270 |
| Rv1473 | *-* | PROBABLE MACROLIDE-TRANSPORT ATP-BINDING PROTEIN ABC TRANSPORTER | 27, 39, 56, 62, 87, 89, 132, 140, 162, 177, 215, 241, 277, 283, 351, 359, 371, 381, 388, 393, 426, 432, 441, 449, 453, 454, 463, 469, 508, 511 |
| Rv1473A | *-* | POSSIBLE TRANSCRIPTIONAL REGULATORY PROTEIN | 20, 31, 50, 52 |
| Rv1474c | *-* | PROBABLE TRANSCRIPTIONAL REGULATORY PROTEIN | 44, 87, 113, 119, 165, 173 |
| Rv1475c | *acn* | aconitate hydratase | 32, 80, 89, 108, 119, 174, 222, 248, 256, 306, 323, 368, 442, 473, 526, 551, 570, 574, 604, 642, 671, 704, 723, 724, 729, 732, 754, 779, 780, 790, 799, 814, 882, 919, 928 |
| Rv1476 | *-* | POSSIBLE MEMBRANE PROTEIN | 1, 23, 30, 111, 118, 124, 163, 173 |
| Rv1477 | *-* | HYPOTHETICAL INVASION PROTEIN | 12, 37, 46, 82, 88, 109, 145, 185, 195, 218, 252, 257, 279, 300, 319, 348, 360, 364, 373, 375, 418 |
| Rv1478 | *-* | HYPOTHETICAL INVASION PROTEIN | 14, 18, 27, 32, 48, 62, 85, 94, 97, 118, 160, 170, 176, 182, 187, 196, 214, 217, 228 |
| Rv1479 | *moxR1* | PROBABLE TRANSCRIPTIONAL REGULATORY PROTEIN MOXR1 | 7, 10, 11, 29, 36, 79, 95, 165, 293, 369, 371, 374 |
| Rv1480 | *-* | hypothetical protein Rv1480 | 16, 66, 78, 111, 132, 134, 146, 155, 178, 195, 249, 281, 283 |
| Rv1481 | *-* | PROBABLE MEMBRANE PROTEIN | 11, 26, 83, 120, 173, 175, 181, 195, 226, 230, 273 |
| Rv1482c | *-* | hypothetical protein Rv1482c | 40, 55, 59, 77, 129, 163, 166, 173, 190, 264 |
| Rv1483 | *fabG1* | 3-OXOACYL- | 20, 48, 88, 91, 113, 141, 157, 176, 213, 217, 232, 240 |
| Rv1484 | *inhA* | enoyl-(acyl carrier protein) reductase | 12, 38, 74, 100, 139, 178, 189, 203, 210, 261 |
| Rv1485 | *hemH* | ferrochelatase | 11, 30, 107, 126, 154, 156, 161, 169, 176, 199, 201, 231, 236 |
| Rv1486c | *-* | hypothetical protein Rv1486c | 16, 48, 69, 91, 105, 106, 118, 136, 157, 181, 229, 240, 266, 275 |
| Rv1487 | *-* | hypothetical protein Rv1487 | 20, 29, 34 |
| Rv1488 | *-* | POSSIBLE EXPORTED CONSERVED PROTEIN | 33, 126, 145, 152, 193, 215, 222, 243, 261, 301, 342 |
| Rv1489 | *-* | hypothetical protein Rv1489 | 52 |
| Rv1489A | *-* | hypothetical protein Rv1489A | 37, 45, 47, 49, 52, 55 |
| Rv1490 | *-* | PROBABLE MEMBRANE PROTEIN | 10, 80, 83, 91, 97, 160, 342, 354, 373, 426, 428 |
| Rv1491c | *-* | hypothetical protein Rv1491c | 3, 86, 91, 92, 110, 123, 168, 169, 189, 191, 202, 204 |
| Rv1492 | *mutA* | PROBABLE METHYLMALONYL-CoA MUTASE SMALL SUBUNIT MUTA (MCM) | 14, 22, 48, 59, 77, 87, 96, 110, 138, 151, 171, 194, 209, 230, 241, 252, 268, 299, 332, 350, 351, 358, 384, 385, 386, 413, 423, 430, 445, 456, 460, 472, 518, 519, 541, 550, 569, 570, 596 |
| Rv1493 | *mutB* | methylmalonyl-CoA mutase | 11, 20, 27, 34, 36, 63, 67, 87, 105, 109, 122, 123, 138, 154, 196, 208, 256, 263, 288, 352, 369, 446, 465, 474, 525, 533, 540, 556, 585, 600, 630, 637, 662, 676, 677, 716, 717, 720 |
| Rv1494 | *-* | hypothetical protein Rv1494 | 7, 36, 63, 67, 85 |
| Rv1495 | *-* | hypothetical protein Rv1495 | 32, 60, 97 |
| Rv1496 | *-* | arginine/ornithine transport system ATPase | 2, 18, 54, 63, 84, 148, 182, 216, 264, 266, 312 |
| Rv1497 | *lipL* | PROBABLE ESTERASE LIPL | 4, 49, 72, 81, 137, 140, 165, 172, 191, 235, 252, 253, 261, 279, 300, 358, 368, 401, 425 |
| Rv1498c | *-* | PROBABLE METHYLTRANSFERASE | 5, 15, 25, 110, 111, 202 |
| Rv1498A | *-* | hypothetical protein Rv1498A | 24 |
| Rv1499 | *-* | hypothetical protein Rv1499 | 8, 16, 28, 78, 111, 124 |
| Rv1500 | *-* | PROBABLE GLYCOSYLTRANSFERASE | 77, 124, 125, 140, 177, 240, 316, 320 |
| Rv1501 | *-* | hypothetical protein Rv1501 | 61, 133, 152, 203 |
| Rv1502 | *-* | hypothetical protein Rv1502 | 40, 60, 74, 93, 201, 227, 289 |
| Rv1503c | *-* | hypothetical protein Rv1503c | 131 |
| Rv1504c | *-* | hypothetical protein Rv1504c | 13, 26, 30, 47, 59, 87, 94, 132, 188, 191 |
| Rv1505c | *-* | hypothetical protein Rv1505c | 9, 44, 84, 134, 157 |
| Rv1506c | *-* | hypothetical protein Rv1506c | 35, 47, 102 |
| Rv1507c | *-* | hypothetical protein Rv1507c | 93, 159, 206 |
| Rv1507A | *-* | hypothetical protein Rv1507A | 97, 110, 161 |
| Rv1508c | *-* | Probable membrane protein | 58, 88, 92, 167, 169, 175, 183, 189, 196, 232, 254, 326, 434, 551, 569, 589 |
| Rv1508A | *-* | hypothetical protein Rv1508A | 6, 26, 76 |
| Rv1509 | *-* | hypothetical protein Rv1509 | 67, 85, 278 |
| Rv1510 | *-* | conserved probable membrane protein | 7, 26, 41, 78, 112, 151, 164, 187, 198, 203, 205, 220, 230, 238, 240, 245, 272, 320, 329, 333, 349, 369, 371, 424 |
| Rv1511 | *gmdA* | GDP-D-mannose dehydratase gmdA (GDP-mannose 4,6 dehydratase) (GMD) | 6, 9, 100, 104, 154, 187, 205, 248, 250, 260, 265, 336 |
| Rv1512 | *epiA* | probable nucleotide-sugar epimerase epiA | 18, 26, 54, 76, 102, 135, 151, 185, 213, 247, 267 |
| Rv1513 | *-* | hypothetical protein Rv1513 | 57, 72, 107, 220 |
| Rv1514c | *-* | hypothetical protein Rv1514c | 44, 53, 68, 91, 96, 109, 132, 144, 174, 183, 240 |
| Rv1515c | *-* | hypothetical protein Rv1515c | 20, 26, 56, 64, 80, 140, 214, 232 |
| Rv1516c | *-* | probable sugar transferase | 81, 86, 88, 249, 276, 296, 300, 312, 331 |
| Rv1517 | *-* | hypothetical protein Rv1517 | 40, 69, 98, 163 |
| Rv1518 | *-* | hypothetical protein Rv1518 | 60, 63, 69, 94, 109, 181, 193, 269, 276 |
| Rv1519 | *-* | hypothetical protein Rv1519 | 86 |
| Rv1520 | *-* | probable sugar transferase | 21, 50, 77, 186, 209, 226, 250, 261, 324, 334, 341 |
| Rv1521 | *fadD25* | acyl-CoA synthetase | 53, 62, 86, 112, 135, 156, 161, 182, 241, 269, 372, 394, 399, 440, 560 |
| Rv1522c | *mmpL12* | PROBABLE CONSERVED TRANSMEMBRANE TRANSPORT PROTEIN MMPL12 | 8, 35, 48, 58, 80, 149, 162, 179, 203, 240, 302, 346, 352, 395, 449, 495, 516, 517, 541, 555, 581, 611, 717, 791, 924, 936, 953, 1048, 1074, 1130 |
| Rv1523 | *-* | Probable methyltransferase | 3, 36, 41, 63, 159, 170, 172, 206, 241, 254, 316, 332, 334 |
| Rv1524 | *-* | Probable glycosyltransferase | 10, 26, 45, 101, 102, 109, 129, 145, 187, 195, 216, 288, 318, 323, 325, 329, 394, 401 |
| Rv1525 | *wbbL2* | POSSIBLE RHAMNOSYL TRANSFERASE WBBL2 | 40, 61, 94, 105, 140, 158, 183, 202, 231 |
| Rv1526c | *-* | Probable glycosyltransferase | 10, 17, 48, 197, 211, 244, 245, 322, 325, 327, 348, 367, 383, 398, 401 |
| Rv1527c | *pks5* | Probable polyketide synthase pks5 | 50, 64, 67, 117, 164, 176, 182, 189, 208, 209, 232, 241, 247, 277, 293, 306, 359, 391, 407, 443, 453, 474, 502, 509, 513, 516, 528, 535, 542, 549, 570, 574, 581, 584, 593, 599, 626, 637, 639, 643, 666, 668, 688, 707, 718, 794, 799, 824, 831, 836, 852, 857, 858, 860, 870, 943, 948, 956, 988, 994, 1014, 1078, 1117, 1153, 1155, 1183, 1217, 1233, 1262, 1271, 1272, 1275, 1281, 1285, 1295, 1368, 1381, 1386, 1395, 1418, 1427, 1436, 1453, 1484, 1492, 1504, 1535, 1540, 1544, 1557, 1565, 1567, 1568, 1573, 1581, 1588, 1622, 1623, 1635, 1637, 1641, 1649, 1724, 1735, 1745, 1769, 1786, 1788, 1821, 1829, 1838, 1845, 1851, 1885, 1900, 1912, 1953, 1977, 2073, 2078, 2106 |
| Rv1528c | *papA4* | PROBABLE CONSERVED POLYKETIDE SYNTHASE ASSOCIATED PROTEIN PAPA4 | 63, 74, 118, 148, 156 |
| Rv1529 | *fadD24* | acyl-CoA synthetase | 7, 32, 60, 84, 96, 112, 136, 162, 169, 183, 295, 325, 331, 371, 416, 448, 455, 456, 490, 534, 553, 561 |
| Rv1530 | *adh* | Probable alcohol dehydrogenase adh | 39, 42, 54, 84, 104, 132, 150, 195, 202, 204, 210, 241, 247, 258, 274, 275, 281, 288, 314, 318, 325, 351 |
| Rv1531 | *-* | hypothetical protein Rv1531 | 111 |
| Rv1532c | *-* | hypothetical protein Rv1532c | 5, 27, 69, 70, 72, 87, 97, 99, 124 |
| Rv1533 | *-* | hypothetical protein Rv1533 | 31, 78, 107, 118, 166, 169, 193, 197, 220, 222, 265, 278, 321, 322, 325 |
| Rv1534 | *-* | Probable transcriptional regulator | 25, 48, 71, 89, 102, 137, 153, 157, 168, 191, 217, 221 |
| Rv1535 | *-* | hypothetical protein Rv1535 | 1, 27, 43, 65 |
| Rv1536 | *ileS* | isoleucyl-tRNA synthetase | 9, 10, 65, 79, 95, 118, 211, 275, 292, 329, 390, 571, 594, 652, 724, 771, 811, 888, 898, 905, 920, 934, 940, 947, 980, 1010 |
| Rv1537 | *dinX* | DNA polymerase IV | 33, 42, 76, 92, 114, 136, 176, 206, 223, 282, 312, 375, 427, 453, 454 |
| Rv1538c | *ansA* | Probable L-aparaginase ansA | 26, 27, 38, 44, 82, 100, 117, 126, 141, 145, 156, 174, 177, 186, 230, 245, 247, 273, 283 |
| Rv1539 | *lspA* | lipoprotein signal peptidase | 17, 26, 59, 86, 124, 142 |
| Rv1540 | *-* | CONSERVED HYPOTHETICAL PROTEIN MEMBER OF yabO/yceC/yfiI FAMILY | 18, 29, 47, 55, 67, 99, 107, 113, 116, 118, 125, 142, 182, 193, 241, 248 |
| Rv1541c | *lprI* | Possible lipoprotein lprI | 8, 16, 27, 28, 60, 76, 114, 116, 134, 162 |
| Rv1542c | *glbN* | Probable hemoglobin glbN | 19, 46, 63, 66, 72, 83, 94, 102, 104 |
| Rv1543 | *-* | POSSIBLE FATTY ACYL-CoA REDUCTASE | 26, 68, 75, 76, 105, 131, 178, 211, 262, 284, 292, 310 |
| Rv1544 | *-* | Possible ketoacyl reductase | 14, 18, 29, 31, 86, 125, 133, 135, 217, 220, 224, 241, 252, 261 |
| Rv1545 | *-* | hypothetical protein Rv1545 | 14, 55, 60, 72 |
| Rv1546 | *-* | hypothetical protein Rv1546 | 6, 81, 99, 110 |
| Rv1547 | *dnaE* | DNA polymerase III subunit alpha | 1, 24, 40, 59, 63, 99, 101, 115, 155, 233, 245, 267, 326, 344, 379, 381, 385, 430, 472, 509, 528, 530, 570, 629, 640, 653, 667, 787, 788, 805, 810, 823, 956, 958, 1007, 1026, 1053, 1136, 1147 |
| Rv1548c | *PPE21* | PPE FAMILY PROTEIN | 18, 31, 34, 53, 68, 78, 144, 148, 177, 178, 203, 238, 248, 251, 268, 277, 291, 297, 301, 308, 311, 321, 331, 338, 361, 371, 385, 437, 442, 447, 462, 467, 472, 482, 488, 493, 501, 511, 526, 531, 536, 546, 559, 574, 594, 607, 627 |
| Rv1549 | *fadD11.1* | POSSIBLE FATTY-ACID-CoA LIGASE FADD11.1 (FATTY-ACID-CoA SYNTHETASE) (FATTY-ACID-CoA SYNTHASE) | 48, 61, 62 |
| Rv1550 | *fadD11* | PROBABLE FATTY-ACID-CoA LIGASE FADD11 (FATTY-ACID-CoA SYNTHETASE) (FATTY-ACID-CoA SYNTHASE) | 4, 37, 68, 101, 112, 114, 139, 188, 201, 211, 233, 304, 338, 355, 370, 385, 397, 423, 438, 457, 480, 494, 495, 522, 523, 539, 543, 561 |
| Rv1551 | *plsB1* | glycerol-3-phosphate acyltransferase | 73, 85, 147, 163, 186, 306, 330, 368, 370, 389, 419, 494, 531, 573 |
| Rv1552 | *frdA* | fumarate reductase flavoprotein subunit | 1, 10, 18, 49, 51, 55, 70, 116, 126, 176, 191, 192, 194, 208, 233, 245, 250, 369, 373, 406, 464 |
| Rv1553 | *frdB* | PROBABLE FUMARATE REDUCTASE | 60, 84, 196, 221, 230, 245 |
| Rv1554 | *frdC* | PROBABLE FUMARATE REDUCTASE | 48, 95, 100 |
| Rv1555 | *frdD* | fumarate reductase subunit D | 24, 29 |
| Rv1556 | *-* | Possible regulatory protein | 69, 84, 148, 165, 200 |
| Rv1557 | *mmpL6* | PROBABLE CONSERVED TRANSMEMBRANE TRANSPORT PROTEIN MMPL6 | 11, 75, 89, 97, 118, 141, 174, 177, 194, 197, 283, 290, 292, 293, 297, 299, 304 |
| Rv1558 | *-* | hypothetical protein Rv1558 | 24, 25, 47, 97, 146 |
| Rv1559 | *ilvA* | threonine dehydratase | 1, 24, 67, 82, 128, 155, 170, 196, 214, 225, 229, 249, 260, 263, 265, 297, 303, 312, 322, 355, 385, 397 |
| Rv1560 | *-* | hypothetical protein Rv1560 | 17 |
| Rv1561 | *-* | hypothetical protein Rv1561 | 15, 19, 78, 84, 88, 129 |
| Rv1562c | *treZ* | Maltooligosyltrehalose trehalohydrolase TreZ | 10, 77, 115, 140, 143, 216, 255, 298, 303, 316, 319, 346, 395, 412, 456, 479, 481, 527, 546 |
| Rv1563c | *treY* | Maltooligosyltrehalose synthase TreY | 13, 51, 79, 168, 170, 174, 176, 194, 206, 247, 285, 313, 351, 359, 377, 380, 405, 410, 422, 448, 458, 486, 516, 542, 545, 593, 609, 664, 679, 687, 688, 691, 700, 718, 737 |
| Rv1564c | *treX* | Probable Maltooligosyltrehalose synthase TreX | 5, 11, 13, 33, 80, 198, 215, 260, 277, 290, 303, 308, 325, 327, 445, 454, 466, 503, 618, 645, 684, 701 |
| Rv1565c | *-* | hypothetical protein Rv1565c | 27, 38, 56, 57, 66, 71, 77, 107, 151, 179, 226, 259, 263, 272, 300, 308, 323, 351, 368, 388, 389, 425, 440, 441, 446, 454, 515, 525, 597, 644 |
| Rv1566c | *-* | Possible inv protein | 6, 22, 24, 28, 48, 53, 60, 61, 69, 71, 73, 83, 91, 93, 101, 225 |
| Rv1567c | *-* | Probable hypothetical membrane protein |  |
| Rv1568 | *bioA* | adenosylmethionine--8-amino-7-oxononanoate transaminase | 5, 39, 65, 73, 122, 139, 141, 144, 153, 171, 182, 196, 210, 214, 226, 257, 262, 267, 271, 285, 286, 292, 297, 306, 309, 348, 349, 352, 353, 357, 362, 370, 389 |
| Rv1569 | *bioF1* | 8-amino-7-oxononanoate synthase | 2, 26, 36, 56, 61, 71, 74, 80, 100, 120, 160, 213, 216, 268, 277, 306, 329, 337, 350, 359, 366 |
| Rv1570 | *bioD* | dithiobiotin synthetase | 6, 8, 24, 28, 40, 42, 44, 62, 74, 76, 80, 85, 100, 110, 131, 139, 174, 183, 193, 196, 197, 199, 204, 221 |
| Rv1571 | *-* | hypothetical protein Rv1571 | 24, 26, 108, 116, 120, 152 |
| Rv1572c | *-* | hypothetical protein Rv1572c |  |
| Rv1573 | *-* | Probable phiRV1 phage protein | 53, 61, 77, 78, 79, 83, 89, 92, 97, 106, 126 |
| Rv1574 | *-* | Probable phiRV1 phage related protein | 24, 25, 58 |
| Rv1575 | *-* | Probable phiRV1 phage protein | 20, 21, 53, 54, 66, 75, 114, 115, 124, 149 |
| Rv1576c | *-* | Probable phiRV1 phage protein | 26, 30, 54, 67, 74, 78, 79, 94, 115, 122, 138, 167, 173, 176, 189, 296, 303, 309, 316, 351, 386, 442, 445, 471 |
| Rv1577c | *-* | Probable phiRv1 phage protein | 47, 134, 145 |
| Rv1578c | *-* | Probable phiRv1 phage protein | 17, 21, 22, 73, 101, 132 |
| Rv1579c | *-* | Probable phiRv1 phage protein | 50, 60, 80 |
| Rv1580c | *-* | Probable phiRv1 phage protein | 44 |
| Rv1581c | *-* | Probable phiRv1 phage protein | 1, 9, 10, 32, 39, 56, 67, 69, 94, 110, 121, 124 |
| Rv1582c | *-* | Probable phiRv1 phage protein | 34, 55, 72, 107, 110, 116, 123, 155, 164, 190, 197, 238, 259, 285, 318, 363, 458 |
| Rv1583c | *-* | Probable phiRv1 phage protein | 1, 2, 4, 5, 21, 33, 54, 77, 107, 111, 121 |
| Rv1584c | *-* | Possible phiRv1 phage protein | 7, 11, 26, 33, 45, 69 |
| Rv1585c | *-* | Possible phage phiRv1 protein | 93, 94, 123, 126 |
| Rv1586c | *-* | Probable phiRv1 integrase | 131, 145, 152, 165, 189, 203, 209, 228, 263, 271, 275, 286, 291, 294, 299, 353, 383, 425, 454 |
| Rv1587c | *-* | Partial REP13E12 repeat protein | 5, 7, 26, 45, 53, 77, 79, 84, 85, 107, 109, 145, 150, 165, 170, 182 |
| Rv1588c | *-* | Partial REP13E12 repeat protein | 70, 88, 95, 105, 114, 116, 118, 143 |
| Rv1589 | *bioB* | biotin synthase | 11, 15, 32, 67, 81, 118, 121, 130, 133, 145, 166, 220, 237, 257, 277, 291, 309, 326 |
| Rv1590 | *-* | hypothetical protein Rv1590 | 14, 21, 46 |
| Rv1591 | *-* | PROBABLE TRANSMEMBRANE PROTEIN | 4, 7, 13, 34, 102, 116, 145, 188, 196 |
| Rv1592c | *-* | hypothetical protein Rv1592c | 3, 10, 42, 49, 71, 147, 173, 204, 205, 213, 217, 251, 254, 266, 300, 364, 365, 399, 422, 436 |
| Rv1593c | *-* | hypothetical protein Rv1593c | 5, 17, 23, 43, 50, 108, 172, 196, 202, 205, 227, 232 |
| Rv1594 | *nadA* | quinolinate synthetase | 25, 34, 44, 56, 75, 86, 94, 111, 132, 188, 197, 204, 239, 254, 255, 307, 328, 344, 345 |
| Rv1595 | *nadB* | L-aspartate oxidase | 1, 3, 19, 26, 30, 46, 53, 75, 121, 136, 147, 148, 155, 167, 170, 180, 192, 193, 209, 238, 241, 253, 284, 294, 304, 323, 336, 342, 360, 367, 387, 390, 393, 395, 398, 401, 419, 423, 439, 441, 451, 477, 479 |
| Rv1596 | *nadC* | nicotinate-nucleotide pyrophosphorylase | 41, 49, 53, 97, 118, 121, 128, 142, 154, 156, 167, 176, 178, 230, 247, 261 |
| Rv1597 | *-* | hypothetical protein Rv1597 | 11, 17, 57, 63, 99, 135, 178 |
| Rv1598c | *-* | hypothetical protein Rv1598c | 1, 40, 48, 61, 86, 96, 108, 109 |
| Rv1599 | *hisD* | histidinol dehydrogenase | 18, 22, 44, 48, 76, 98, 146, 149, 175, 189, 200, 216, 225, 252, 259, 309, 323, 332, 340, 379, 396, 412 |
| Rv1600 | *hisC1* | histidinol-phosphate aminotransferase | 3, 79, 83, 86, 113, 177, 194, 236, 237, 247, 268, 272, 285, 323, 331, 341, 348 |
| Rv1601 | *hisB* | imidazoleglycerol-phosphate dehydratase | 32, 39, 49, 84, 113, 118, 126, 138 |
| Rv1602 | *hisH* | imidazole glycerol phosphate synthase subunit HisH | 32, 40, 63, 73, 74, 91, 102, 127, 150, 181, 193 |
| Rv1603 | *hisA* | 1-(5-phosphoribosyl)-5- | 7, 24, 36, 59, 80, 81, 90, 95, 140, 142, 148, 178, 186, 199, 217, 240 |
| Rv1604 | *impA* | PROBABLE INOSITOL-MONOPHOSPHATASE IMPA (IMP) | 19, 61, 71, 93, 94, 113, 126, 191, 217, 225, 226, 247 |
| Rv1605 | *hisF* | imidazole glycerol phosphate synthase subunit HisF | 39, 46, 63, 67, 79, 90, 115, 161, 166, 197, 209, 218, 221, 232, 259 |
| Rv1606 | *hisI* | phosphoribosyl-AMP cyclohydrolase | 19, 27, 67, 93, 98, 106 |
| Rv1607 | *chaA* | Probable ionic transporter integral membrane protein chaA | 39, 44, 83, 84, 98, 108, 129, 131, 160, 166, 168, 244, 286, 334 |
| Rv1608c | *bcpB* | Probable peroxidoxin BcpB | 2, 15, 97, 104 |
| Rv1609 | *trpE* | anthranilate synthase component I | 44, 96, 205, 248, 322, 327, 393, 401, 407, 412, 435, 439, 448, 459, 472, 501, 506 |
| Rv1610 | *-* | POSSIBLE CONSERVED MEMBRANE PROTEIN | 4, 27, 28, 34, 57, 63, 74, 83, 117, 163, 191 |
| Rv1611 | *trpC* | indole-3-glycerol-phosphate synthase | 18, 32, 49, 83, 143, 180, 219, 224, 231, 247, 252, 259 |
| Rv1612 | *trpB* | tryptophan synthase subunit beta | 14, 15, 41, 123, 125, 134, 152, 205, 233, 246, 271, 277, 281, 285, 289, 314, 332, 364, 369, 399 |
| Rv1613 | *trpA* | tryptophan synthase subunit alpha | 32, 50, 81, 90, 98, 99, 126, 176, 188, 225, 239, 256, 261, 268 |
| Rv1614 | *lgt* | prolipoprotein diacylglyceryl transferase | 12, 31, 47, 51, 68, 87, 90, 172, 234, 246, 251, 303, 309, 344, 360, 384, 395, 434 |
| Rv1615 | *-* | Probable hypothetical membrane protein | 34, 37, 65, 77, 89, 99, 113 |
| Rv1616 | *-* | hypothetical protein Rv1616 | 3, 10, 12, 19, 51, 53, 130 |
| Rv1617 | *pykA* | pyruvate kinase | 3, 26, 57, 79, 117, 147, 159, 164, 309, 338, 375, 395 |
| Rv1618 | *tesB1* | Probable acyl-CoA thioesterase II tesB1 | 27, 72, 115, 118, 143, 257, 289 |
| Rv1619 | *-* | hypothetical protein Rv1619 | 4, 65, 76, 94, 108, 110, 116, 134, 184, 186, 201, 271, 317, 323, 338, 360, 361, 362, 376, 393, 407 |
| Rv1620c | *cydC* | PROBABLE 'COMPONENT LINKED WITH THE ASSEMBLY OF CYTOCHROME' TRANSPORT TRANSMEMBRANE ATP-BINDING PROTEIN ABC TRANSPORTER CYDC | 15, 45, 78, 92, 96, 97, 115, 121, 155, 157, 167, 178, 186, 189, 221, 244, 257, 270, 292, 301, 303, 341, 350, 373, 376, 378, 388, 437, 457, 461, 510, 524, 560 |
| Rv1621c | *cydD* | PROBABLE 'COMPONENT LINKED WITH THE ASSEMBLY OF CYTOCHROME' TRANSPORT TRANSMEMBRANE ATP-BINDING PROTEIN ABC TRANSPORTER CYDD | 2, 7, 22, 27, 65, 75, 80, 83, 96, 113, 122, 168, 174, 185, 197, 256, 280, 286, 303, 309, 311, 334, 339, 346, 351, 361, 364, 374, 424, 435, 443, 475, 510, 512 |
| Rv1622c | *cydB* | Probable integral membrane cytochrome D ubiquinol oxidase (subunit II) cydB (Cytochrome BD-I oxidase subunit II) | 66, 67, 73, 76, 84, 107, 117, 143, 168, 172, 173, 189, 307, 338 |
| Rv1623c | *cydA* | Probable integral membrane cytochrome D ubiquinol oxidase (subunit I) cydA (Cytochrome BD-I oxidase subunit I) | 44, 67, 138, 161, 177, 187, 192, 193, 198, 223, 234, 237, 242, 306, 363, 381, 389, 408, 425 |
| Rv1624c | *-* | Probable conserved membrane protein | 62, 78, 86, 88, 119, 149 |
| Rv1625c | *cya* | MEMBRANE-ANCHORED ADENYLYL CYCLASE CYA (ATP PYROPHOSPHATE-LYASE) (ADENYLATE CYCLASE) | 5, 50, 58, 68, 70, 82, 107, 132, 145, 155, 156, 188, 266, 297, 305, 336, 348, 404, 426, 429, 433 |
| Rv1626 | *-* | Probable two-component system transcriptional regulator | 1, 9, 43, 75, 91, 105, 135, 179 |
| Rv1627c | *-* | lipid-transfer protein | 13, 32, 35, 40, 51, 65, 76, 85, 89, 103, 126, 266, 288, 305, 329, 335, 344, 354, 369, 373, 385 |
| Rv1628c | *-* | hypothetical protein Rv1628c | 19, 26, 32, 49, 62, 91, 112, 148 |
| Rv1629 | *polA* | DNA polymerase I | 33, 40, 52, 145, 193, 196, 204, 207, 213, 235, 302, 304, 316, 322, 333, 355, 360, 371, 380, 419, 497, 517, 540, 543, 578, 594, 623, 637, 639, 692, 703, 724, 770, 864, 879 |
| Rv1630 | *rpsA* | 30S ribosomal protein S1 | 23, 63, 144, 148, 228, 428, 435, 442, 458, 477 |
| Rv1631 | *coaE* | dephospho-CoA kinase/unknown domain fusion protein | 6, 7, 11, 23, 79, 117, 144, 234, 247, 282, 283, 313, 326, 356, 382 |
| Rv1632c | *-* | hypothetical protein Rv1632c | 82, 83, 144 |
| Rv1633 | *uvrB* | excinuclease ABC subunit B | 2, 3, 15, 20, 42, 44, 50, 71, 126, 191, 197, 231, 245, 251, 318, 322, 348, 404, 405, 416, 542, 547, 607, 613, 615, 621, 629, 635, 644, 659, 665, 694 |
| Rv1634 | *-* | Possible drug efflux membrane protein | 7, 25, 111, 119, 124, 161, 182, 195, 201, 222, 230, 240, 244, 246, 272, 310, 315, 343, 380, 407, 417, 422, 425, 434, 451 |
| Rv1635c | *-* | Probable conserved transmembrane protein | 5, 11, 18, 19, 35, 42, 55, 68, 72, 143, 145, 176, 183, 199, 242, 259, 314, 321, 326, 345, 367, 369, 390, 397, 432, 441, 446, 468 |
| Rv1636 | *TB15.3* | IRON-REGULATED CONSERVED HYPOTHETICAL PROTEIN TB15.3 | 27, 48, 62, 79 |
| Rv1637c | *-* | hypothetical protein Rv1637c | 11, 28, 31, 47, 52, 61, 85, 93, 131, 134, 138, 175, 179, 187, 191, 199, 205, 206, 240, 261 |
| Rv1638 | *uvrA* | excinuclease ABC subunit A | 30, 33, 123, 211, 223, 319, 328, 469, 501, 502, 516, 577, 587, 601, 606, 637, 652, 668, 682, 689, 717, 728, 736, 740, 749, 756, 816, 838, 843, 844, 861, 920, 922, 923, 944, 953, 958 |
| Rv1638A | *-* | hypothetical protein Rv1638A |  |
| Rv1639c | *-* | hypothetical protein Rv1639c | 7, 38, 61, 77, 97, 116, 146, 153, 163, 166, 195, 227, 233, 239, 251, 254, 265, 266, 338, 357, 379, 408, 412, 420, 422, 463, 484, 486 |
| Rv1640c | *lysS* | lysyl-tRNA synthetase | 14, 80, 83, 136, 157, 162, 168, 171, 217, 262, 299, 304, 307, 335, 352, 380, 399, 417, 422, 468, 480, 509, 565, 651, 664, 698, 728, 782, 790, 798, 842, 873, 876, 907, 958, 961, 990, 1014, 1025, 1026, 1036, 1076, 1093, 1116, 1117, 1140, 1141 |
| Rv1641 | *infC* | translation initiation factor IF-3 | 37, 152, 168, 184, 192, 197 |
| Rv1642 | *rpmI* | 50S ribosomal protein L35 | 7, 16, 18 |
| Rv1643 | *rplT* | 50S ribosomal protein L20 | 24, 84, 85, 106, 124, 127 |
| Rv1644 | *tsnR* | Possible 23S rRNA methyltransferase tsnR | 40, 61, 85, 103, 133, 139, 155, 171, 179, 186, 243, 245 |
| Rv1645c | *-* | hypothetical protein Rv1645c | 7, 26, 45, 81, 129, 213, 220, 269, 288, 319, 327, 338 |
| Rv1646 | *PE17* | PE FAMILY PROTEIN | 13, 14, 27, 42, 81, 84, 91, 105, 124, 183, 185, 203, 208, 213, 218, 224, 232, 237, 299, 303 |
| Rv1647 | *-* | hypothetical protein Rv1647 | 21, 29, 40, 77, 85, 168, 169, 219, 226, 237, 284 |
| Rv1648 | *-* | Probable transmembrane protein | 113, 145, 171, 177, 196, 239, 240, 255 |
| Rv1649 | *pheS* | phenylalanyl-tRNA synthetase subunit alpha | 12, 58, 64, 68, 105, 154, 222, 260, 272, 274, 280, 282, 293, 301 |
| Rv1650 | *pheT* | phenylalanyl-tRNA synthetase subunit beta | 61, 100, 105, 137, 144, 146, 149, 172, 179, 196, 235, 250, 282, 292, 295, 308, 323, 344, 365, 380, 393, 429, 470, 494, 500, 529, 599, 634, 697, 702, 750, 754, 780, 811, 815 |
| Rv1651c | *PE_PGRS30* | PE-PGRS FAMILY PROTEIN | 14, 27, 29, 36, 41, 81, 87, 91, 127, 131, 132, 135, 137, 145, 148, 150, 151, 154, 156, 157, 163, 165, 166, 168, 169, 175, 178, 180, 181, 183, 184, 191, 193, 194, 200, 203, 206, 207, 216, 219, 224, 225, 227, 228, 238, 243, 248, 252, 258, 269, 280, 283, 289, 290, 294, 302, 304, 305, 308, 311, 314, 316, 319, 320, 322, 323, 331, 332, 335, 338, 340, 351, 354, 356, 357, 360, 367, 369, 372, 373, 386, 389, 391, 395, 406, 410, 424, 426, 427, 429, 430, 432, 433, 436, 443, 448, 452, 462, 464, 465, 467, 470, 479, 484, 487, 496, 499, 501, 502, 504, 507, 513, 516, 520, 534, 535, 537, 538, 540, 541, 544, 546, 547, 556, 560, 566, 575, 576, 579, 581, 591, 594, 596, 599, 605, 610, 613, 616, 619, 622, 629, 631, 632, 634, 635, 638, 640, 642, 646, 647, 649, 653, 656, 670, 673, 674, 676, 680, 686, 688, 691, 692, 702, 767, 800, 816, 842, 871, 910, 924, 959, 988, 989, 990, 994 |
| Rv1652 | *argC* | N-acetyl-gamma-glutamyl-phosphate reductase | 14, 17, 20, 45, 50, 108, 117, 129, 147, 155, 160, 168, 171, 177, 185, 213, 289, 315, 324, 325, 327 |
| Rv1653 | *argJ* | bifunctional ornithine acetyltransferase/N-acetylglutamate synthase protein | 4, 16, 19, 23, 24, 27, 28, 33, 49, 51, 73, 83, 84, 90, 101, 106, 116, 126, 140, 151, 155, 159, 184, 209, 213, 222, 247, 290, 292, 301, 313, 327, 348, 358 |
| Rv1654 | *argB* | acetylglutamate kinase | 35, 49, 58, 67, 73, 87, 127, 135, 161, 194, 196, 233, 260, 286, 292 |
| Rv1655 | *argD* | acetylornithine aminotransferase | 1, 31, 50, 62, 111, 126, 133, 149, 162, 177, 184, 196, 202, 207, 216, 227, 232, 255, 269, 283, 289, 331, 333, 342, 352, 356, 386 |
| Rv1656 | *argF* | ornithine carbamoyltransferase | 39, 64, 72, 86, 146, 167, 170, 191, 200, 207, 213, 215, 268 |
| Rv1657 | *argR* | arginine repressor | 5, 14, 18, 44, 68, 72, 74, 87, 91, 110, 143, 157, 162 |
| Rv1658 | *argG* | argininosuccinate synthase | 9, 10, 25, 39, 103, 109, 118, 186, 223, 247, 274, 281, 321, 335, 344, 375, 378, 391 |
| Rv1659 | *argH* | argininosuccinate lyase | 9, 13, 21, 39, 51, 64, 89, 104, 109, 118, 134, 147, 156, 163, 205, 217, 225, 233, 239, 279, 297, 300, 308, 347, 366, 387, 391, 402, 415, 444, 459 |
| Rv1660 | *pks10* | Possible chalcone synthase pks10 | 90, 102, 106, 118, 139, 142, 152, 191, 196, 234, 277, 329, 332 |
| Rv1661 | *pks7* | Probable polyketide synthase pks7 | 73, 95, 103, 154, 185, 203, 251, 262, 266, 272, 295, 439, 447, 543, 546, 582, 610, 613, 686, 688, 689, 726, 788, 794, 795, 822, 838, 849, 866, 889, 920, 985, 1017, 1033, 1058, 1157, 1200, 1206, 1207, 1224, 1240, 1244, 1245, 1272, 1356, 1372, 1418, 1419, 1439, 1507, 1509, 1510, 1568, 1578, 1591, 1626, 1644, 1688, 1701, 1704, 1727, 1744, 1780, 1830, 1861, 1891, 1924, 1927, 1942, 1951, 1985, 2012, 2022, 2024, 2052, 2054, 2108 |
| Rv1662 | *pks8* | Probable polyketide synthase pks8 | 72, 87, 94, 106, 153, 158, 165, 182, 226, 235, 257, 259, 263, 269, 292, 339, 383, 435, 443, 471, 486, 529, 540, 564, 660, 675, 683, 684, 692, 766, 782, 786, 826, 827, 911, 961, 972, 996, 1025, 1143, 1156, 1194, 1198, 1201, 1257, 1276, 1278, 1326, 1351, 1352, 1397, 1399, 1418, 1486, 1489, 1512, 1547, 1557, 1571, 1597 |
| Rv1663 | *pks17* | Probable polyketide synthase pks17 | 2, 61, 68, 114, 157, 197, 228, 274, 291, 294, 307, 349, 356, 387, 466, 472, 485 |
| Rv1664 | *pks9* | Probable polyketide synthase pks9 | 20, 84, 141, 167, 191, 209, 233, 240, 245, 253, 264, 281, 318, 321, 325, 348, 361, 406, 446, 483, 484, 507, 555, 576, 603, 611, 618, 626, 729, 769, 771, 792, 814, 847, 900, 915, 942, 968, 978 |
| Rv1665 | *pks11* | Possible chalcone synthase pks11 | 28, 42, 102, 106, 118, 172, 184, 192, 208, 277, 329, 332 |
| Rv1666c | *cyp139* | Probable cytochrome P450 139 CYP139 | 15, 21, 63, 109, 127, 170, 232, 239, 253, 272, 296, 356, 365, 397, 410 |
| Rv1667c | *-* | PROBABLE SECOND PART OF MACROLIDE-TRANSPORT ATP-BINDING PROTEIN ABC TRANSPORTER | 6, 39, 116, 117, 156, 170 |
| Rv1668c | *-* | PROBABLE FIRST PART OF MACROLIDE-TRANSPORT ATP-BINDING PROTEIN ABC TRANSPORTER | 50, 57, 64, 107, 123, 172, 173, 205, 221, 241, 256, 278, 325, 335, 340, 348, 366 |
| Rv1669 | *-* | hypothetical protein Rv1669 | 11, 20, 36, 94 |
| Rv1670 | *-* | hypothetical protein Rv1670 |  |
| Rv1671 | *-* | PROBABLE MEMBRANE PROTEIN | 5, 73, 119 |
| Rv1672c | *-* | PROBABLE CONSERVED INTEGRAL MEMBRANE TRANSPORT PROTEIN | 15, 53, 57, 59, 72, 82, 121, 125, 172, 175, 229, 235, 271, 275, 287, 315, 319, 342, 346, 349, 356, 360, 381, 386, 393, 404, 409 |
| Rv1673c | *-* | hypothetical protein Rv1673c | 5, 8, 157, 201, 215, 223, 286 |
| Rv1674c | *-* | PROBABLE TRANSCRIPTIONAL REGULATORY PROTEIN | 1, 20, 61, 127, 128, 143, 144, 148, 167, 202 |
| Rv1675c | *-* | PROBABLE TRANSCRIPTIONAL REGULATORY PROTEIN | 38, 63, 73, 103, 176, 200 |
| Rv1676 | *-* | hypothetical protein Rv1676 | 49, 72, 121, 191, 203 |
| Rv1677 | *dsbF* | PROBABLE CONSERVED LIPOPROTEIN DSBF | 27, 32, 36, 48, 72, 96, 108, 114 |
| Rv1678 | *-* | PROBABLE INTEGRAL MEMBRANE PROTEIN | 5, 19, 20, 26, 51, 96, 104, 111, 130, 156, 163, 179, 202, 220, 235, 238, 253, 256, 265, 270, 275 |
| Rv1679 | *fadE16* | POSSIBLE ACYL-CoA DEHYDROGENASE FADE16 | 37, 49, 53, 82, 87, 103, 163, 186, 195, 213, 214, 241, 244, 246, 253, 281, 290, 315, 327, 330, 354, 367 |
| Rv1680 | *-* | hypothetical protein Rv1680 | 24, 54, 74, 75, 98, 109, 117, 156, 169, 183, 187, 210, 216 |
| Rv1681 | *moeX* | POSSIBLE MOLYBDOPTERIN BIOSYNTHESIS PROTEIN MOEX | 16, 37, 49, 58, 74, 129, 130, 194, 219, 225, 244, 257, 276, 286, 319, 323 |
| Rv1682 | *-* | Probable coiled-coil structural protein | 25, 30, 117, 172 |
| Rv1683 | *-* | acyl-CoA synthetase | 33, 98, 115, 158, 181, 226, 287, 317, 322, 362, 384, 408, 515, 534, 559, 606, 641, 675, 683, 736, 782, 802, 808, 827, 832, 837, 862, 870, 886, 899, 918, 946, 973, 974, 990, 995 |
| Rv1684 | *-* | hypothetical protein Rv1684 |  |
| Rv1685c | *-* | hypothetical protein Rv1685c | 10, 13, 14, 45, 48, 52, 67, 109, 149 |
| Rv1686c | *-* | PROBABLE CONSERVED INTEGRAL MEMBRANE PROTEIN ABC TRANSPORTER | 23, 27, 31, 77, 81, 156, 195 |
| Rv1687c | *-* | PROBABLE CONSERVED ATP-BINDING PROTEIN ABC TRANSPORTER | 15, 41, 45, 51, 115, 121, 136, 143, 144, 207, 231, 251, 253 |
| Rv1688 | *mpg* | 3-methyladenine DNA glycosylase | 12, 22, 37, 47, 53, 85, 92, 98, 112, 123, 171 |
| Rv1689 | *tyrS* | tyrosyl-tRNA synthetase | 11, 24, 29, 36, 41, 47, 62, 70, 71, 74, 127, 202, 224, 235, 236, 259, 313, 364, 417 |
| Rv1690 | *lprJ* | PROBABLE LIPOPROTEIN LPRJ | 25, 31, 54, 79 |
| Rv1691 | *-* | hypothetical protein Rv1691 | 9, 17, 28, 65, 75, 91, 100, 104, 135, 148, 156, 159, 167, 168, 197, 225 |
| Rv1692 | *-* | PROBABLE PHOSPHATASE | 25, 26, 63, 65, 81, 103, 128, 136, 163, 176, 186, 188, 219, 229, 313, 325 |
| Rv1693 | *-* | hypothetical protein Rv1693 | 21, 49 |
| Rv1694 | *tlyA* | CYTOTOXIN\|HAEMOLYSIN HOMOLOGUE TLYA | 22, 28, 43, 49, 64, 92, 104, 109, 144, 164, 193 |
| Rv1695 | *ppnK* | inorganic polyphosphate/ATP-NAD kinase | 1, 12, 20, 82, 147, 196, 199, 265, 301, 304 |
| Rv1696 | *recN* | PROBABLE DNA REPAIR PROTEIN RECN (RECOMBINATION PROTEIN N) | 16, 21, 27, 30, 32, 39, 56, 114, 116, 119, 124, 157, 232, 241, 270, 301, 325, 359, 369, 393, 429, 457, 458, 491, 494, 524, 536, 542, 561, 579 |
| Rv1697 | *-* | hypothetical protein Rv1697 | 34, 49, 91, 110, 114, 121, 139, 149, 189, 213, 242, 249, 274, 296, 297, 345 |
| Rv1698 | *-* | hypothetical protein Rv1698 | 25, 108, 109, 113, 127, 142, 157, 191, 212, 213, 220, 231, 237, 244, 253, 279, 302 |
| Rv1699 | *pyrG* | CTP synthetase | 6, 14, 26, 90, 97, 110, 207, 325, 326, 350, 364, 384, 402, 436, 442, 451, 490, 495 |
| Rv1700 | *-* | hypothetical protein Rv1700 | 74, 89, 112, 127, 170, 180, 200 |
| Rv1701 | *xerD* | site-specific tyrosine recombinase XerD | 64, 69, 75, 87, 94, 133, 156, 181, 216, 226, 246, 249, 250 |
| Rv1702c | *-* | hypothetical protein Rv1702c | 10, 17, 50, 70, 85, 95, 105, 114, 116, 118, 122, 127, 160, 229, 231, 236, 244, 259, 269, 287, 303, 308, 309, 350, 361, 398, 407 |
| Rv1703c | *-* | Probable catechol-o-methyltransferase | 54, 80, 152 |
| Rv1704c | *cycA* | PROBABLE D-SERINE/ALANINE/GLYCINE TRANSPORTER PROTEIN CYCA | 36, 49, 88, 111, 120, 128, 230, 287, 290, 302, 310, 315, 337, 341, 343, 361, 410, 436, 438, 481, 515, 542 |
| Rv1705c | *PPE22* | PPE FAMILY PROTEIN | 11, 16, 25, 28, 34, 40, 78, 94, 98, 101, 129, 159, 176, 183, 189, 193, 220, 223, 276, 280, 289, 292, 331, 352, 357, 361, 383 |
| Rv1706c | *PPE23* | PPE FAMILY PROTEIN | 24, 31, 52, 56, 62, 70, 115, 129, 135, 184, 193, 196, 213, 232, 261, 273, 279, 284, 291, 296, 317, 330, 333, 346, 351, 372 |
| Rv1706A | *-* | hypothetical protein Rv1706A | 13, 23, 39 |
| Rv1707 | *-* | PROBABLE CONSERVED TRANSMEMBRANE PROTEIN | 28, 30, 46, 57, 64, 72, 90, 150, 263, 264, 268, 271, 273, 289, 305, 393, 467, 484 |
| Rv1708 | *-* | PUTATIVE INITIATION INHIBITOR PROTEIN | 37, 44, 54, 86, 107, 154, 218, 241 |
| Rv1709 | *-* | hypothetical protein Rv1709 | 82, 96, 137, 263 |
| Rv1710 | *-* | hypothetical protein Rv1710 | 19, 48, 70, 74, 112, 135, 149, 161, 214 |
| Rv1711 | *-* | hypothetical protein Rv1711 | 29, 82, 92, 187, 210, 211 |
| Rv1712 | *cmk* | cytidylate kinase | 14, 16, 36, 50, 83, 92, 101, 149, 196, 197 |
| Rv1713 | *engA* | GTP-binding protein EngA | 22, 57, 71, 80, 81, 104, 114, 119, 124, 132, 139, 152, 183, 191, 195, 244, 253, 276, 280, 308, 350, 377, 396, 410, 426, 458 |
| Rv1714 | *-* | Probable oxidoreductase | 27, 30, 37, 51, 52, 56, 65, 70, 138, 148, 158, 164, 165, 189, 205, 256, 264, 267 |
| Rv1715 | *fadB3* | PROBABLE 3-HYDROXYBUTYRYL-CoA DEHYDROGENASE FADB3 (BETA-HYDROXYBUTYRYL-CoA DEHYDROGENASE) (BHBD) | 9, 14, 22, 44, 49, 53, 61, 67, 148, 259, 260, 267, 275, 283, 291 |
| Rv1716 | *-* | hypothetical protein Rv1716 | 8, 59, 87, 102, 115, 123, 178, 197, 227, 234, 264, 271 |
| Rv1717 | *-* | hypothetical protein Rv1717 | 44, 45 |
| Rv1718 | *-* | hypothetical protein Rv1718 | 9, 38, 40, 80, 144, 173, 176, 178, 191, 208, 215, 268 |
| Rv1719 | *-* | PROBABLE TRANSCRIPTIONAL REGULATORY PROTEIN | 1, 29, 30, 64, 151, 225, 237 |
| Rv1720c | *-* | hypothetical protein Rv1720c |  |
| Rv1721c | *-* | hypothetical protein Rv1721c | 1 |
| Rv1722 | *-* | biotin carboxylase-like protein | 3, 104, 187, 192, 200, 209, 263, 274, 279, 297, 323, 391, 396, 431, 449, 462, 481 |
| Rv1723 | *-* | PROBABLE HYDROLASE | 1, 6, 37, 46, 72, 74, 84, 122, 136, 144, 168, 184, 189, 206, 225, 227, 256, 261, 266, 290, 296, 306, 315, 324, 325, 348, 357, 362 |
| Rv1724c | *-* | hypothetical protein Rv1724c | 88, 106 |
| Rv1725c | *-* | hypothetical protein Rv1725c | 43, 71, 79, 104, 126, 169, 180, 186, 190 |
| Rv1726 | *-* | PROBABLE OXIDOREDUCTASE | 1, 21, 63, 67, 71, 77, 79, 84, 128, 129, 135, 138, 143, 167, 169, 189, 191, 213, 224, 228, 234, 261, 273, 278, 315, 357, 359, 362, 411, 417 |
| Rv1727 | *-* | hypothetical protein Rv1727 | 58, 67, 135, 183 |
| Rv1728c | *-* | hypothetical protein Rv1728c | 6, 57, 183, 234, 239 |
| Rv1729c | *-* | hypothetical protein Rv1729c | 17, 33, 96, 108, 149, 169, 170, 202, 207, 217, 249, 260, 278, 293 |
| Rv1730c | *-* | POSSIBLE PENICILLIN-BINDING PROTEIN | 8, 12, 16, 81, 82, 163, 176, 199, 219, 253, 279, 324, 332, 343, 353, 381, 400, 488, 496 |
| Rv1731 | *gabD2* | succinic semialdehyde dehydrogenase | 15, 21, 33, 135, 169, 173, 210, 218, 230, 235, 243, 255, 283, 318, 339, 355, 419, 422, 429, 455, 482, 514 |
| Rv1732c | *-* | hypothetical protein Rv1732c | 12, 23, 35, 53, 54, 115, 150, 162, 176 |
| Rv1733c | *-* | PROBABLE CONSERVED TRANSMEMBRANE PROTEIN | 59, 62, 64, 83, 85, 100, 124, 135, 146, 166, 175 |
| Rv1734c | *-* | hypothetical protein Rv1734c | 50, 60, 75 |
| Rv1735c | *-* | HYPOTHETICAL MEMBRANE PROTEIN | 9, 26, 49, 88, 121, 127 |
| Rv1736c | *narX* | PROBABLE NITRATE REDUCTASE NARX | 6, 17, 36, 64, 100, 104, 124, 162, 165, 180, 196, 210, 220, 250, 275, 287, 305, 328, 329, 335, 355, 369, 382, 389, 407, 491, 492, 510, 516, 528, 561, 587, 649 |
| Rv1737c | *narK2* | POSSIBLE NITRATE/NITRITE TRANSPORTER NARK2 | 60, 68, 109, 129, 133, 143, 168, 173, 200, 219, 247, 248, 261, 279, 281, 287, 313, 314, 326, 333, 337, 340, 343, 372, 380 |
| Rv1738 | *-* | hypothetical protein Rv1738 | 1 |
| Rv1739c | *-* | PROBABLE SULPHATE-TRANSPORT TRANSMEMBRANE PROTEIN ABC TRANSPORTER | 7, 25, 30, 35, 55, 82, 84, 96, 97, 143, 158, 207, 238, 261, 274, 279, 301, 302, 317, 355, 364, 406, 434, 449, 533, 551 |
| Rv1740 | *-* | hypothetical protein Rv1740 | 26, 39, 55, 65, 68 |
| Rv1741 | *-* | hypothetical protein Rv1741 | 59, 80 |
| Rv1742 | *-* | hypothetical protein Rv1742 | 12, 19, 68, 73, 117, 176 |
| Rv1743 | *pknE* | PROBABLE TRANSMEMBRANE SERINE/THREONINE-PROTEIN KINASE E PKNE (PROTEIN KINASE E) (STPK E) | 3, 23, 66, 101, 128, 132, 209, 263, 276, 316, 351, 376, 411, 424, 456, 475, 488, 522, 535, 540 |
| Rv1744c | *-* | PROBABLE MEMBRANE PROTEIN | 18, 27, 32, 39, 44, 51 |
| Rv1745c | *idi* | isopentenyl-diphosphate delta-isomerase | 19, 33, 59, 74, 84, 113, 159, 160, 198 |
| Rv1746 | *pknF* | ANCHORED-MEMBRANE SERINE/THREONINE-PROTEIN KINASE PKNF (PROTEIN KINASE F) (STPK F) | 9, 19, 20, 62, 77, 109, 139, 162, 169, 172, 204, 212, 222, 278, 295, 297, 305, 310, 348, 356, 372, 375, 381, 397, 402, 432, 457 |
| Rv1747 | *-* | PROBABLE CONSERVED TRANSMEMBRANE ATP-BINDING PROTEIN ABC TRANSPORTER | 6, 23, 109, 124, 136, 141, 155, 168, 177, 191, 199, 226, 258, 316, 321, 343, 347, 415, 469, 481, 501, 526, 527, 530, 582, 651, 667, 692, 722, 781, 788, 810, 820, 833 |
| Rv1748 | *-* | hypothetical protein Rv1748 | 6, 66, 105, 149, 162, 166, 177, 200, 230, 237 |
| Rv1749c | *-* | POSSIBLE INTEGRAL MEMBRANE PROTEIN | 47, 78, 86, 89, 113, 133 |
| Rv1750c | *fadD1* | acyl-CoA synthetase | 71, 74, 91, 158, 161, 177, 215, 217, 227, 306, 362, 400, 488, 503, 525 |
| Rv1751 | *-* | hypothetical protein Rv1751 | 33, 35, 58, 62, 162, 178, 184, 388, 432, 451, 453 |
| Rv1752 | *-* | hypothetical protein Rv1752 | 44, 47, 48, 107, 115, 142, 144, 146 |
| Rv1753c | *PPE24* | PPE FAMILY PROTEIN | 16, 26, 40, 49, 53, 76, 86, 155, 183, 188, 191, 193, 201, 204, 214, 240, 278, 283, 303, 311, 317, 327, 335, 350, 370, 385, 400, 415, 474, 490, 505, 516, 531, 542, 557, 568, 583, 594, 609, 620, 624, 630, 675, 742, 743, 772, 783, 788, 793, 823, 848, 873, 898, 933, 948, 958, 967, 970, 991, 995, 1003, 1014, 1034 |
| Rv1754c | *-* | hypothetical protein Rv1754c | 75, 95, 98, 112, 129, 134, 150, 160, 218, 229, 244, 257, 301, 309, 338, 390, 394, 427, 430, 504, 525, 526 |
| Rv1755c | *plcD* | PROBABLE PHOSPHOLIPASE C 4 (FRAGMENT) PLCD | 115, 206, 229, 276 |
| Rv1756c | *-* | PUTATIVE TRANSPOSASE | 50, 87, 91, 107, 241, 255, 271, 291, 292 |
| Rv1757c | *-* | PUTATIVE TRANSPOSASE | 24, 60, 64, 71, 93 |
| Rv1758 | *cut1* | PROBABLE CUTINASE CUT1 | 39, 63, 76, 101, 145, 164, 168, 169 |
| Rv1759c | *wag22* | PE-PGRS FAMILY PROTEIN | 40, 41, 81, 88, 91, 107, 109, 122, 124, 131, 140, 141, 143, 150, 158, 159, 165, 167, 168, 170, 171, 173, 175, 181, 183, 187, 189, 196, 198, 199, 201, 202, 213, 215, 216, 219, 222, 226, 228, 229, 231, 232, 234, 235, 242, 246, 248, 249, 251, 257, 258, 261, 263, 270, 273, 275, 276, 278, 279, 284, 285, 288, 291, 301, 302, 304, 305, 310, 316, 317, 320, 323, 329, 330, 333, 339, 346, 347, 350, 351, 353, 357, 366, 368, 369, 371, 372, 375, 381, 382, 385, 387, 388, 393, 394, 395, 408, 410, 411, 413, 414, 417, 422, 425, 437, 443, 446, 449, 453, 455, 459, 461, 471, 474, 480, 483, 486, 489, 526, 541, 545, 547, 548, 555, 558, 560, 563, 570, 572, 576, 582, 585, 586, 588, 589, 596, 597, 599, 602, 603, 605, 611, 612, 614, 618, 620, 624, 625, 628, 630, 631, 634, 644, 647, 652, 658, 660, 661, 664, 667, 675, 676, 678, 679, 681, 684, 685, 688, 689, 691, 692, 694, 695, 703, 704, 710, 715, 719, 721, 722, 724, 730, 731, 734, 736, 737, 743, 744, 747, 750, 762, 764, 765, 767, 776, 779, 781, 782, 791, 792, 794, 795, 806, 809, 811, 814, 818, 822, 825, 827, 828, 830, 831, 836, 838, 839, 842, 844, 845, 848, 854, 859, 860, 866, 871, 872, 881, 884, 887, 890, 896, 897, 899 |
| Rv1760 | *-* | hypothetical protein Rv1760 | 5, 29, 38, 69, 78, 124, 126, 140, 158, 180, 204, 206, 252, 302, 335, 354, 362, 363, 372, 387, 408, 418 |
| Rv1761c | *-* | hypothetical protein Rv1761c | 14, 19, 20, 30, 39, 120 |
| Rv1762c | *-* | hypothetical protein Rv1762c | 35, 56, 107, 118, 123, 126, 142, 152, 214, 237, 245, 247 |
| Rv1763 | *-* | PUTATIVE TRANSPOSASE | 24, 60, 64, 71, 93 |
| Rv1764 | *-* | PUTATIVE TRANSPOSASE | 50, 87, 91, 107, 241, 255, 271, 291, 292 |
| Rv1765c | *-* | hypothetical protein Rv1765c | 3, 28, 123, 133, 223, 250, 286, 311, 356, 359 |
| Rv1765A | *-* | PUTATIVE TRANSPOSASE (FRAGMENT) | 17, 67 |
| Rv1766 | *-* | hypothetical protein Rv1766 | 8, 22, 42, 51, 65, 70 |
| Rv1767 | *-* | hypothetical protein Rv1767 | 37, 72, 76 |
| Rv1768 | *PE_PGRS31* | PE-PGRS FAMILY PROTEIN | 30, 41, 42, 51, 80, 81, 82, 93, 132, 135, 142, 147, 148, 153, 157, 166, 168, 169, 171, 172, 178, 187, 190, 198, 200, 201, 203, 207, 214, 219, 222, 223, 226, 229, 236, 238, 242, 245, 251, 260, 261, 269, 270, 279, 280, 283, 285, 289, 300, 303, 306, 309, 312, 321, 322, 327, 328, 330, 332, 339, 342, 344, 345, 348, 357, 359, 360, 362, 365, 373, 381, 384, 391, 394, 395, 398, 413, 415, 416, 419, 436, 459, 462, 465, 475, 478, 484, 489, 490, 494, 497, 506, 507, 509, 512, 513, 521, 534, 537, 539, 540, 542, 543, 547, 548, 550, 551, 564, 566, 569, 574, 578, 587, 594, 599, 601, 605, 615 |
| Rv1769 | *-* | hypothetical protein Rv1769 | 55, 98, 161, 166, 167, 218, 260, 263, 279, 280, 300, 312, 318, 327, 360, 380 |
| Rv1770 | *-* | hypothetical protein Rv1770 | 14, 31, 53, 103, 116, 120, 156, 173, 176, 193, 213, 216, 222, 230, 238, 252, 259, 294, 361, 377, 381 |
| Rv1771 | *-* | PROBABLE OXIDOREDUCTASE | 46, 64, 113, 116, 118, 123, 141, 143, 234, 255, 334, 343, 358, 377, 389, 404 |
| Rv1772 | *-* | hypothetical protein Rv1772 | 15, 20, 30, 45, 53 |
| Rv1773c | *-* | PROBABLE TRANSCRIPTIONAL REGULATORY PROTEIN | 23, 85, 90, 115, 146, 163, 186, 200, 222, 234, 238 |
| Rv1774 | *-* | PROBABLE OXIDOREDUCTASE | 5, 21, 64, 70, 105, 137, 160, 179, 181, 183, 243, 260, 323, 393 |
| Rv1775 | *-* | hypothetical protein Rv1775 | 42, 52, 84, 88, 125, 127, 153, 200, 211, 242 |
| Rv1776c | *-* | POSSIBLE TRANSCRIPTIONAL REGULATORY PROTEIN | 8, 21, 55, 56, 69, 118, 138, 141, 142 |
| Rv1777 | *cyp144* | Probable cytochrome p450 144 CYP144 | 23, 57, 73, 93, 103, 113, 121, 218, 237, 251, 262, 272, 273, 274, 321, 339, 361, 414 |
| Rv1778c | *-* | hypothetical protein Rv1778c | 16, 61, 82, 138, 139, 147 |
| Rv1779c | *-* | HYPOTHETICAL INTEGRAL MEMBRANE PROTEIN | 13, 23, 42, 50, 53, 91, 99, 102, 128, 174, 200, 203, 278, 305, 315, 329, 341, 401, 423, 485, 571 |
| Rv1780 | *-* | hypothetical protein Rv1780 | 26, 46, 47, 97, 105, 108, 136, 169, 184 |
| Rv1781c | *malQ* | PROBABLE 4-ALPHA-GLUCANOTRANSFERASE MALQ (Amylomaltase) (Disproportionating enzyme) (D-enzyme) | 40, 45, 57, 76, 132, 153, 210, 224, 226, 315, 392, 425, 464, 469, 516, 555, 583, 584, 588, 611, 687 |
| Rv1782 | *-* | PROBABLE CONSERVED MEMBRANE PROTEIN | 5, 23, 32, 78, 92, 116, 135, 154, 188, 205, 275, 369, 380, 423 |
| Rv1783 | *-* | PROBABLE CONSERVED MEMBRANE PROTEIN | 50, 59, 65, 85, 88, 140, 293, 351, 369 |
| Rv1784 | *-* | hypothetical protein Rv1784 | 29, 38, 41, 43, 122, 140, 213, 220, 229, 232, 259, 318, 406, 408, 417, 418, 452, 467, 496, 510, 513, 575, 608, 624, 690, 751, 769, 798, 807, 818, 850, 869, 898, 903, 908, 918 |
| Rv1785c | *cyp143* | PROBABLE CYTOCHROME P450 143 CYP143 | 3, 8, 104, 292, 335 |
| Rv1786 | *-* | PROBABLE FERREDOXIN | 51 |
| Rv1787 | *PPE25* | PPE FAMILY PROTEIN | 16, 27, 42, 52, 78, 89, 92, 98, 175, 261, 262, 272, 331, 343, 363 |
| Rv1788 | *PE18* | PE FAMILY PROTEIN | 14, 22, 42, 68, 91, 95, 97 |
| Rv1789 | *PPE26* | PPE FAMILY PROTEIN | 16, 34, 42, 77, 91, 94, 101, 134, 147, 176, 182, 187, 189, 191, 201, 294, 302, 307, 334, 351, 357, 362, 370, 372, 391 |
| Rv1790 | *PPE27* | PPE FAMILY PROTEIN | 16, 27, 42, 52, 78, 89, 92, 98, 175, 246, 247, 257, 310 |
| Rv1791 | *PE19* | PE FAMILY PROTEIN | 30, 36, 40, 42, 50, 68, 95, 97 |
| Rv1793 | *esxN* | PUTATIVE ESAT-6 LIKE PROTEIN ESXN (ESAT-6 LIKE PROTEIN 5) | 36, 42, 44, 77 |
| Rv1794 | *-* | hypothetical protein Rv1794 | 78, 90, 150, 166, 194, 195, 205, 209, 218, 247, 262 |
| Rv1795 | *-* | hypothetical protein Rv1795 | 1, 26, 70, 120, 148, 154, 173, 220, 246, 303, 312, 332, 360, 382, 392, 416, 444 |
| Rv1796 | *mycP5* | PROBABLE PROLINE RICH MEMBRANE-ANCHORED MYCOSIN MYCP5 (SERINE PROTEASE) (SUBTILISIN-LIKE PROTEASE) (SUBTILASE-LIKE) (MYCOSIN-5) | 5, 17, 21, 31, 55, 74, 98, 123, 129, 227, 229, 238, 273, 347, 352, 467, 472, 497, 501, 503, 545, 556, 557, 561, 567, 573 |
| Rv1797 | *-* | hypothetical protein Rv1797 | 16, 122, 169, 194, 274, 328, 348, 354, 359 |
| Rv1798 | *-* | hypothetical protein Rv1798 | 5, 16, 37, 61, 70, 294, 301, 358, 360, 374, 413, 537, 545 |
| Rv1799 | *lppT* | PROBABLE LIPOPROTEIN LPPT | 10, 60 |
| Rv1800 | *PPE28* | PPE FAMILY PROTEIN | 18, 30, 70, 88, 93, 103, 135, 177, 179, 205, 221, 288, 289, 337, 356, 394, 503, 533, 568, 578, 631 |
| Rv1801 | *PPE29* | PPE FAMILY PROTEIN | 25, 52, 56, 67, 77, 109, 146, 153, 158, 182, 187, 190, 192, 193, 205, 218, 241, 245, 251, 257, 290, 317, 338, 355, 382, 383, 386, 393, 396, 400, 402 |
| Rv1802 | *PPE30* | PPE FAMILY PROTEIN | 11, 16, 18, 25, 52, 85, 128, 147, 153, 175, 192, 209, 225, 287, 302, 313, 336, 342, 367, 372, 374, 384, 389, 447, 448 |
| Rv1803c | *PE_PGRS32* | PE-PGRS FAMILY PROTEIN | 105, 118, 133, 140, 150, 152, 153, 159, 162, 170, 174, 177, 185, 188, 194, 195, 198, 200, 213, 214, 223, 234, 235, 243, 251, 268, 272, 274, 287, 300, 302, 303, 318, 319, 322, 325, 328, 337, 339, 343, 348, 358, 360, 367, 370, 373, 379, 382, 383, 389, 401, 405, 407, 429, 440, 454, 464, 470, 472, 482, 487, 494, 496, 502, 505, 514, 524, 526, 533, 536, 537, 540, 542, 543, 546, 552, 555, 561, 571, 573, 577, 579, 582, 583, 585, 586, 592, 594, 595, 601, 607, 611, 617, 619, 623, 626 |
| Rv1804c | *-* | hypothetical protein Rv1804c | 24, 60, 76, 90 |
| Rv1805c | *-* | hypothetical protein Rv1805c | 1, 67, 102 |
| Rv1806 | *PE20* | PE FAMILY PROTEIN | 14, 25, 42, 58, 91 |
| Rv1807 | *PPE31* | PPE FAMILY PROTEIN | 16, 25, 34, 52, 66, 68, 78, 85, 92, 98, 130, 154, 159, 176, 189, 205, 227, 260, 277, 285, 295, 309, 312, 317, 337, 346, 362, 365, 373, 395, 397 |
| Rv1808 | *PPE32* | PPE FAMILY PROTEIN | 20, 25, 34, 39, 65, 78, 79, 154, 179, 188, 189, 191, 220, 256, 276, 283, 285, 296, 331, 345, 347, 349, 352, 369, 376, 382, 389, 406 |
| Rv1809 | *PPE33* | PPE FAMILY PROTEIN | 11, 20, 62, 65, 68, 91, 98, 129, 159, 176, 179, 183, 189, 193, 200, 254, 298, 313, 356, 374, 380, 383, 442 |
| Rv1810 | *-* | hypothetical protein Rv1810 | 22, 29, 31, 41, 45, 50, 63, 89 |
| Rv1811 | *mgtC* | POSSIBLE Mg2+ TRANSPORT P-TYPE ATPASE C MGTC | 18, 43, 75, 87, 99, 105, 107, 117, 185, 226 |
| Rv1812c | *-* | PROBABLE DEHYDROGENASE | 8, 69, 75, 79, 92, 112, 141, 142, 157, 160, 220, 225, 230, 249, 291, 315, 391 |
| Rv1813c | *-* | hypothetical protein Rv1813c | 8, 12, 13, 47, 65, 88, 102, 114, 116, 133 |
| Rv1814 | *erg3* | MEMBRANE-BOUND C-5 STEROL DESATURASE ERG3 (STEROL-C5-DESATURASE) | 30, 33, 38, 62, 63, 74, 86, 134, 143, 214, 291 |
| Rv1815 | *-* | hypothetical protein Rv1815 | 17, 23, 33, 58, 64, 88, 118, 181, 183, 195, 198 |
| Rv1816 | *-* | POSSIBLE TRANSCRIPTIONAL REGULATORY PROTEIN | 34, 102, 109, 130, 146, 151, 179 |
| Rv1817 | *-* | hypothetical protein Rv1817 | 5, 30, 31, 39, 41, 51, 55, 58, 61, 69, 77, 129, 144, 161, 171, 172, 174, 185, 206, 210, 220, 232, 233, 272, 273, 296, 317, 360, 380, 382, 416, 421, 440, 461, 475 |
| Rv1818c | *PE_PGRS33* | PE-PGRS FAMILY PROTEIN | 26, 30, 40, 42, 55, 80, 81, 88, 91, 107, 127, 131, 134, 141, 143, 144, 146, 149, 152, 156, 158, 165, 167, 168, 170, 176, 178, 182, 184, 185, 187, 188, 194, 195, 197, 198, 200, 201, 204, 209, 214, 215, 217, 218, 221, 227, 228, 230, 231, 234, 240, 243, 246, 249, 256, 258, 259, 261, 262, 264, 269, 270, 272, 273, 275, 276, 279, 285, 286, 289, 292, 301, 303, 304, 306, 309, 310, 312, 321, 322, 324, 325, 333, 335, 338, 339, 341, 348, 350, 351, 360, 361, 363, 364, 367, 370, 376, 377, 379, 380, 382, 386, 390, 393, 395, 396, 399, 408, 411, 412, 417, 419, 422, 426, 429, 435, 438, 440, 445, 449, 455, 464, 467, 470, 481, 482, 484, 485 |
| Rv1819c | *-* | PROBABLE DRUGS-TRANSPORT TRANSMEMBRANE ATP-BINDING PROTEIN ABC TRANSPORTER | 195, 196, 199, 295, 303, 358, 414, 437, 451, 458, 463, 487, 517, 552, 582, 608, 623, 635 |
| Rv1820 | *ilvG* | hypothetical protein Rv1820 | 4, 33, 58, 62, 76, 79, 105, 134, 181, 189, 194, 246, 250, 302, 319, 326, 329, 372, 392, 407, 417, 444, 501, 510, 526 |
| Rv1821 | *secA2* | preprotein translocase subunit SecA | 19, 38, 65, 97, 110, 115, 132, 140, 147, 152, 153, 158, 194, 204, 262, 410, 415, 418, 419, 494, 497, 529, 531, 538, 572, 575, 581, 667, 748, 761, 803 |
| Rv1822 | *pgsA2* | PROBABLE CDP-DIACYLGLYCEROL--GLYCEROL-3-PHOSPHATE 3-PHOSPHATIDYLTRANSFERASE PGSA2 (PGP SYNTHASE) (PHOSPHATIDYLGLYCEROPHOSPHATE SYNTHASE) (3-PHOSPHATIDYL-1'-GLYCEROL-3'PHOSPHATE SYNTHASE) | 90 |
| Rv1823 | *-* | hypothetical protein Rv1823 | 8, 14, 49, 74, 79, 84, 94, 98, 112, 155, 173, 188, 230, 231, 264, 292 |
| Rv1824 | *-* | hypothetical protein Rv1824 | 29, 45, 96, 110 |
| Rv1825 | *-* | hypothetical protein Rv1825 | 13, 92, 135, 151, 163, 176, 192, 219, 245, 262, 276 |
| Rv1826 | *gcvH* | glycine cleavage system protein H | 10, 49, 52, 53, 98, 128 |
| Rv1827 | *cfp17* | CONSERVED HYPOTHETICAL PROTEIN CFP17 | 45, 65, 147, 158 |
| Rv1828 | *-* | hypothetical protein Rv1828 | 6, 9, 54, 106, 111, 117, 149, 154, 157, 189, 202, 211, 225 |
| Rv1829 | *-* | hypothetical protein Rv1829 | 40, 97, 124, 138 |
| Rv1830 | *-* | hypothetical protein Rv1830 | 40, 44, 49, 65, 99, 122, 171, 201 |
| Rv1831 | *-* | hypothetical protein Rv1831 | 42 |
| Rv1832 | *gcvB* | glycine dehydrogenase | 49, 51, 110, 138, 152, 166, 182, 204, 219, 222, 257, 279, 283, 297, 335, 354, 357, 371, 392, 441, 444, 501, 525, 543, 592, 595, 598, 658, 681, 698, 785, 800, 806, 832, 867, 878, 883, 907, 915 |
| Rv1833c | *-* | haloalkane dehalogenase | 126, 171, 193, 208, 277 |
| Rv1834 | *-* | Probable hydrolase | 55, 96, 165, 257, 280 |
| Rv1835c | *-* | hypothetical protein Rv1835c | 3, 12, 18, 48, 54, 58, 89, 101, 111, 170, 191, 192, 196, 228, 247, 250, 269, 317, 322, 327, 350, 351, 359, 412, 421, 435, 446, 481, 491, 519, 555, 591, 602 |
| Rv1836c | *-* | hypothetical protein Rv1836c | 36, 53, 56, 61, 85, 98, 103, 130, 150, 155, 198, 216, 234, 236, 274, 284, 306, 310, 316, 348, 357, 378, 481, 484, 501, 542, 555, 569, 601, 652, 669 |
| Rv1837c | *glcB* | malate synthase G | 102, 111, 112, 190, 208, 213, 217, 220, 254, 263, 276, 280, 307, 320, 322, 323, 367, 389, 448, 457, 463, 473, 498, 501, 502, 505, 534, 538, 560, 693, 700, 724, 734 |
| Rv1838c | *-* | hypothetical protein Rv1838c | 32 |
| Rv1839c | *-* | hypothetical protein Rv1839c | 44, 56, 76, 78 |
| Rv1840c | *PE_PGRS34* | PE-PGRS FAMILY PROTEIN | 18, 40, 80, 81, 87, 94, 124, 125, 127, 128, 135, 138, 141, 146, 147, 149, 159, 165, 167, 170, 173, 176, 177, 180, 187, 190, 192, 193, 197, 203, 207, 209, 212, 220, 222, 223, 225, 226, 231, 235, 238, 240, 241, 243, 244, 251, 254, 261, 264, 267, 279, 284, 287, 292, 295, 298, 305, 307, 320, 321, 324, 330, 333, 342, 345, 347, 348, 355, 358, 372, 375, 387, 390, 392, 393, 395, 400, 401, 405, 420, 421, 423, 437, 439, 440, 442, 446, 449, 458, 460, 461, 464, 469, 471, 472, 475, 484, 489, 493, 496, 498, 502, 506, 512 |
| Rv1841c | *-* | hypothetical protein Rv1841c | 4, 60, 92, 232, 237, 246, 256, 315 |
| Rv1842c | *-* | hypothetical protein Rv1842c | 16, 30, 42, 76, 137, 143, 194, 197, 244, 246, 277, 315, 323, 325, 356, 375, 388, 401, 443, 448 |
| Rv1843c | *guaB1* | inositol-5-monophosphate dehydrogenase | 9, 37, 53, 56, 67, 159, 197, 202, 237, 246, 287, 298, 308, 316, 336, 349, 350, 362, 368, 396, 425, 475, 476 |
| Rv1844c | *gnd1* | 6-phosphogluconate dehydrogenase | 7, 69, 83, 102, 109, 134, 137, 138, 151, 152, 154, 169, 186, 219, 243, 254, 271, 278, 287, 307, 309, 320, 323, 351, 371, 403, 442, 446 |
| Rv1845c | *-* | hypothetical protein Rv1845c | 1, 32, 43, 51, 62, 76, 110, 158, 172, 202, 232, 236, 237, 251, 283, 285, 291 |
| Rv1846c | *-* | POSSIBLE TRANSCRIPTIONAL REGULATORY PROTEIN | 79, 92, 97, 123 |
| Rv1847 | *-* | hypothetical protein Rv1847 | 7, 51, 76, 100, 127 |
| Rv1848 | *ureA* | urease subunit gamma | 15, 25, 58 |
| Rv1849 | *ureB* | urease subunit beta | 2, 16, 29, 85, 91, 98 |
| Rv1850 | *ureC* | urease subunit alpha | 16, 42, 49, 75, 103, 126, 133, 154, 162, 165, 167, 178, 216, 245, 259, 270, 282, 283, 383, 391, 403, 489, 492, 564 |
| Rv1851 | *ureF* | Urease accessory protein uref | 23, 30, 31, 35, 60, 83, 94, 107, 122, 129, 146, 151, 160, 183 |
| Rv1852 | *ureG* | Urease accessory protein ureG | 12, 30, 35, 38, 86, 87, 92, 99, 110, 122, 123, 146, 147, 155, 219 |
| Rv1853 | *ureD* | Probable urease accessory protein ureD | 14, 21, 32, 37, 58, 65, 81, 94, 153, 194 |
| Rv1854c | *ndh* | PROBABLE NADH DEHYDROGENASE NDH | 24, 59, 94, 117, 135, 180, 185, 215, 277, 299, 311, 351, 387, 425, 448 |
| Rv1855c | *-* | POSSIBLE OXIDOREDUCTASE | 82, 104, 113, 180, 191, 215, 248, 274, 290, 295 |
| Rv1856c | *-* | short chain dehydrogenase | 7, 8, 35, 41, 84, 123, 124, 137, 142, 156, 162, 171, 177, 186, 209 |
| Rv1857 | *modA* | PROBABLE MOLYBDATE-BINDING LIPOPROTEIN MODA | 26, 31, 61, 87, 96, 109, 124, 154, 165, 187, 215, 233, 244 |
| Rv1858 | *modB* | PROBABLE MOLBDENUM-TRANSPORT INTEGRAL MEMBRANE PROTEIN ABC TRANSPORTER MODB | 56, 79, 122, 130, 155, 156, 171, 189, 209 |
| Rv1859 | *modC* | PROBABLE MOLYBDENUM-TRANSPORT ATP-BINDING PROTEIN ABC TRANSPORTER MODC | 21, 38, 66, 104, 109, 139, 166, 171, 173, 187, 213, 227, 238, 257, 309, 316, 328, 335, 343 |
| Rv1860 | *apa* | ALANINE AND PROLINE RICH SECRETED PROTEIN APA (FIBRONECTIN ATTACHMENT PROTEIN) (Immunogenic protein MPT32) (Antigen MPT-32) (45-kDa glycoprotein) (45/47 kDa antigen) | 33, 35, 50, 56, 73, 132, 146, 152, 184, 245, 252, 265, 299, 306 |
| Rv1861 | *-* | PROBABLE CONSERVED TRANSMEMBRANE PROTEIN | 9, 28, 31, 42, 60, 74, 85 |
| Rv1862 | *adhA* | Probable alcohol dehydrogenase adhA | 17, 22, 47, 70, 89, 108, 116, 128, 152, 165, 180, 190, 196, 212, 231, 241, 254, 262, 268, 308, 334, 338, 340 |
| Rv1863c | *-* | PROBABLE CONSERVED INTEGRAL MEMBRANE PROTEIN | 59, 87, 125, 148, 196, 203, 212, 215, 226, 246 |
| Rv1864c | *-* | hypothetical protein Rv1864c | 44, 60, 83, 170, 182, 187 |
| Rv1865c | *-* | short chain dehydrogenase | 22, 23, 34, 97, 138, 150, 182, 202, 260, 282 |
| Rv1866 | *-* | hypothetical protein Rv1866 | 18, 19, 31, 64, 79, 107, 133, 134, 148, 197, 202, 236, 261, 285, 294, 325, 329, 339, 346, 370, 387, 388, 489, 512, 514, 565, 590, 661, 676, 682, 686, 720, 742, 757 |
| Rv1867 | *-* | acetyl-CoA acetyltransferase | 35, 41, 66, 81, 84, 87, 104, 107, 146, 153, 222, 247, 297, 323, 328, 346, 352, 353, 375, 382, 385, 411, 418, 466, 478, 489 |
| Rv1868 | *-* | hypothetical protein Rv1868 | 22, 27, 57, 75, 80, 89, 92, 102, 118, 193, 244, 334, 345, 374, 410, 441, 450, 459, 500, 503, 549, 551, 564, 587, 591, 595, 609 |
| Rv1869c | *-* | Probable reductase | 10, 14, 28, 53, 88, 123, 124, 153, 161, 176, 183, 221, 232, 252, 259, 281, 304, 309, 352, 369, 388 |
| Rv1870c | *-* | hypothetical protein Rv1870c | 25, 60, 66, 110, 121, 137, 151, 176 |
| Rv1871c | *-* | hypothetical protein Rv1871c | 44, 47, 75, 82, 89, 103, 111 |
| Rv1872c | *lldD2* | POSSIBLE L-LACTATE DEHYDROGENASE (CYTOCHROME) LLDD2 | 32, 90, 108, 121, 127, 176, 192, 290, 301, 332, 363 |
| Rv1873 | *-* | hypothetical protein Rv1873 | 29, 33, 45, 81, 130 |
| Rv1874 | *-* | hypothetical protein Rv1874 | 71, 102, 185 |
| Rv1875 | *-* | hypothetical protein Rv1875 | 6, 43 |
| Rv1876 | *bfrA* | PROBABLE BACTERIOFERRITIN BFRA | 19 |
| Rv1877 | *-* | PROBABLE CONSERVED INTEGRAL MEMBRANE PROTEIN | 11, 15, 30, 41, 48, 108, 128, 134, 139, 165, 199, 225, 234, 252, 266, 315, 333, 384, 425, 442, 468, 552, 554, 603, 618, 642, 654 |
| Rv1878 | *glnA3* | PROBABLE GLUTAMINE SYNTHETASE GLNA3 (GLUTAMINE SYNTHASE) (GS-I) | 6, 8, 28, 29, 69, 85, 98, 99, 110, 122, 127, 160, 174, 207, 221, 237, 242, 259, 270, 273, 280, 290, 295, 301, 305, 325, 332, 386 |
| Rv1879 | *-* | hypothetical protein Rv1879 | 3, 4, 100, 101, 110, 117, 123, 150, 189, 196, 204, 256, 273, 287, 323, 368 |
| Rv1880c | *cyp140* | Probable cytochrome p450 140 CYP140 | 14, 98, 144, 156, 247, 263, 269, 319, 336, 346, 351, 367, 375, 406, 409, 419 |
| Rv1881c | *lppE* | POSSIBLE CONSERVED LIPOPROTEIN LPPE | 8, 19, 21, 31, 45, 73, 97, 114 |
| Rv1882c | *-* | short chain dehydrogenase | 6, 8, 71, 75, 85, 90, 100, 118, 179, 184, 202, 209 |
| Rv1883c | *-* | hypothetical protein Rv1883c | 31, 48, 50, 100 |
| Rv1884c | *rpfC* | PROBABLE RESUSCITATION-PROMOTING FACTOR RPFC | 30, 46, 49, 61, 66, 80, 88, 94, 104, 107, 139, 141, 153 |
| Rv1885c | *-* | chorismate mutase | 11, 44, 69, 155 |
| Rv1886c | *fbpB* | SECRETED ANTIGEN 85-B FBPB (85B) (ANTIGEN 85 COMPLEX B) (MYCOLYL TRANSFERASE 85B) (FIBRONECTIN-BINDING PROTEIN B) (EXTRACELLULAR ALPHA-ANTIGEN) | 31, 32, 36, 67, 112, 126, 149, 157, 167, 175, 185, 210, 211, 215, 250, 259, 264, 285, 287, 323 |
| Rv1887 | *-* | hypothetical protein Rv1887 | 37, 46, 61, 91, 154, 164, 175, 249, 256, 280, 326, 331, 368 |
| Rv1888c | *-* | POSSIBLE TRANSMEMBRANE PROTEIN | 42, 116, 140, 175 |
| Rv1888A | *-* | hypothetical protein Rv1888A | 48 |
| Rv1889c | *-* | hypothetical protein Rv1889c | 23, 57, 58, 83 |
| Rv1890c | *-* | hypothetical protein Rv1890c | 38, 44, 65, 71, 85, 127, 134, 195 |
| Rv1891 | *-* | hypothetical protein Rv1891 | 7, 58, 77, 102, 125, 127 |
| Rv1892 | *-* | PROBABLE MEMBRANE PROTEIN | 25, 51, 82, 99 |
| Rv1893 | *-* | hypothetical protein Rv1893 | 29, 34, 39, 42, 64 |
| Rv1894c | *-* | hypothetical protein Rv1894c | 168, 171, 192, 195, 196, 228, 245, 255, 258, 263, 313, 363, 372 |
| Rv1895 | *-* | POSSIBLE DEHYDROGENASE | 8, 22, 32, 34, 37, 67, 86, 88, 104, 111, 115, 126, 152, 166, 189, 223, 228, 253, 270, 325 |
| Rv1896c | *-* | hypothetical protein Rv1896c | 24, 53, 74, 106, 107, 120, 169, 189, 225, 229, 243 |
| Rv1897c | *-* | D-tyrosyl-tRNA deacylase | 62, 82, 92, 119 |
| Rv1898 | *-* | hypothetical protein Rv1898 | 31, 64, 80 |
| Rv1899c | *lppD* | POSSIBLE LIPOPROTEIN LPPD | 4, 13, 19, 23, 25, 28, 31, 34, 37, 38, 43, 49, 52, 55, 58, 59, 65, 68, 70, 85, 201, 211, 212, 236, 262, 265, 290, 335, 339 |
| Rv1900c | *lipJ* | PROBABLE LIGNIN PEROXIDASE LIPJ | 14, 78, 120, 182, 194, 198, 259, 270, 282, 285, 309, 336, 343, 356, 369, 396, 410, 426 |
| Rv1901 | *cinA* | competence damage-inducible protein A | 11, 17, 45, 49, 70, 76, 89, 115, 119, 127, 137, 147, 149, 163, 187, 232, 245, 287, 292, 304, 359, 361, 364, 400, 423 |
| Rv1902c | *nanT* | PROBABLE SIALIC ACID-TRANSPORT INTEGRAL MEMBRANE PROTEIN NANT | 6, 53, 114, 121, 137, 195, 214, 228, 246, 253, 259, 274, 333, 349, 381, 402 |
| Rv1903 | *-* | PROBABLE CONSERVED MEMBRANE PROTEIN | 7, 10, 28, 84, 104 |
| Rv1904 | *-* | hypothetical protein Rv1904 | 9, 25, 34, 43, 62, 99, 117, 119, 133 |
| Rv1905c | *aao* | PROBABLE D-AMINO ACID OXIDASE AAO | 12, 15, 62, 79, 81, 97, 102, 119, 164, 183, 211, 212, 228, 270, 317 |
| Rv1906c | *-* | hypothetical protein Rv1906c | 15, 23, 29, 57, 62, 123 |
| Rv1907c | *-* | hypothetical protein Rv1907c | 27, 68, 106, 128, 140, 148, 199 |
| Rv1908c | *katG* | CATALASE-PEROXIDASE-PEROXYNITRITASE T KATG | 12, 14, 31, 64, 91, 109, 118, 121, 123, 167, 210, 242, 267, 277, 305, 307, 314, 353, 358, 360, 368, 474, 488, 529, 549, 689 |
| Rv1909c | *furA* | FERRIC UPTAKE REGULATION PROTEIN FURA (FUR) | 66, 99 |
| Rv1910c | *-* | PROBABLE EXPORTED PROTEIN | 51, 90, 120, 126, 128, 136, 139, 142, 150, 183 |
| Rv1911c | *lppC* | PROBABLE LIPOPROTEIN LPPC | 11, 23, 25, 27, 51, 88, 105, 114, 153, 187, 189 |
| Rv1912c | *fadB5* | POSSIBLE OXIDOREDUCTASE FADB5 | 87, 118, 126, 139, 147, 149, 170, 210, 255, 321 |
| Rv1913 | *-* | hypothetical protein Rv1913 | 30, 37, 145, 147, 161, 204 |
| Rv1914c | *-* | hypothetical protein Rv1914c | 7, 55, 76, 129 |
| Rv1915 | *aceAa* | PROBABLE ISOCITRATE LYASE aceAa | 22, 41, 49, 135, 144, 177, 179, 182, 209, 218, 234, 245, 338, 355 |
| Rv1916 | *aceAb* | isocitrate lyase | 31, 38, 67, 79, 93, 128, 155, 169, 201, 207, 213, 250, 268, 297 |
| Rv1917c | *PPE34* | PPE FAMILY PROTEIN | 16, 26, 65, 76, 89, 137, 155, 178, 183, 190, 217, 230, 231, 250, 270, 278, 284, 294, 304, 333, 373, 378, 383, 388, 413, 419, 451, 475, 510, 543, 550, 610, 635, 660, 683, 715, 718, 729, 743, 780, 790, 796, 826, 836, 886, 915, 927, 929, 937, 957, 962, 977, 997, 1022, 1032, 1063, 1196, 1247, 1248, 1270, 1293, 1316, 1325, 1330, 1337, 1340, 1377, 1384, 1399, 1434, 1456 |
| Rv1918c | *PPE35* | PPE FAMILY PROTEIN | 16, 20, 49, 53, 110, 114, 130, 134, 137, 155, 179, 181, 186, 198, 203, 238, 291, 309, 313, 333, 343, 353, 358, 378, 404, 408, 418, 467, 472, 477, 602, 622, 627, 669, 676, 831, 861, 876, 900, 911, 928, 940, 947, 955, 967 |
| Rv1919c | *-* | hypothetical protein Rv1919c | 46, 49, 51, 52, 106 |
| Rv1920 | *-* | PROBABLE MEMBRANE PROTEIN | 31, 77, 124, 132, 133, 246, 254, 256 |
| Rv1921c | *lppF* | PROBABLE CONSERVED LIPOPROTEIN LPPF | 30, 60, 62, 108, 125, 131, 137, 185, 190, 202, 275, 279, 285, 331, 332, 371, 392 |
| Rv1922 | *-* | PROBABLE CONSERVED LIPOPROTEIN | 16, 22, 25, 63, 77, 84, 104, 106, 114, 147, 178, 289, 299, 306, 308, 314, 331 |
| Rv1923 | *lipD* | PROBABLE LIPASE LIPD | 9, 10, 46, 57, 95, 101, 107, 117, 151, 198, 205, 267, 301, 382 |
| Rv1924c | *-* | hypothetical protein Rv1924c | 14, 42 |
| Rv1925 | *fadD31* | acyl-CoA synthetase | 12, 25, 61, 62, 90, 106, 259, 287, 292, 302, 308, 369, 384, 419, 474, 483, 497, 520, 529, 533, 591, 599, 613 |
| Rv1926c | *mpt63* | IMMUNOGENIC PROTEIN MPT63 (ANTIGEN MPT63/MPB63) (16 kDa IMMUNOPROTECTIVE EXTRACELLULAR PROTEIN) | 33, 66, 85, 90, 99, 111, 118, 129, 137 |
| Rv1927 | *-* | hypothetical protein Rv1927 | 1, 4, 11, 58, 86, 109, 163, 187, 214 |
| Rv1928c | *-* | short chain dehydrogenase | 16, 20, 33, 40, 82, 95, 125, 128, 139, 241 |
| Rv1929c | *-* | hypothetical protein Rv1929c | 37, 85, 117, 138, 150, 159, 175, 197 |
| Rv1930c | *-* | hypothetical protein Rv1930c | 9, 12, 49, 77, 78, 87, 91, 113, 125, 128, 143 |
| Rv1931c | *-* | PROBABLE TRANSCRIPTIONAL REGULATORY PROTEIN | 6, 16, 19, 23, 38, 40, 80, 100, 144, 146, 168, 200, 227, 230, 234 |
| Rv1932 | *tpx* | thiol peroxidase | 29, 92, 102, 124, 160 |
| Rv1933c | *fadE18* | PROBABLE ACYL-CoA DEHYDROGENASE FADE18 | 16, 30, 49, 58, 67, 107, 111, 140, 143, 161, 174, 178, 203, 208, 227, 245, 282, 303, 311, 313 |
| Rv1934c | *fadE17* | PROBABLE ACYL-CoA DEHYDROGENASE FADE17 | 25, 28, 30, 50, 83, 121, 135, 164, 167, 197, 208, 225, 238, 247, 253, 272, 307, 311, 315, 347, 350, 376, 381 |
| Rv1935c | *echA13* | enoyl-CoA hydratase | 37, 52, 60, 78, 79, 81, 84, 85, 91, 100, 116, 184, 206, 226, 266, 287, 288, 305, 311 |
| Rv1936 | *-* | POSSIBLE MONOOXYGENASE | 107, 149, 183, 187, 195, 226, 321, 363, 365 |
| Rv1937 | *-* | POSSIBLE OXYGENASE | 42, 53, 54, 98, 103, 109, 118, 129, 136, 145, 162, 187, 213, 216, 281, 295, 303, 334, 358, 360, 362, 367, 415, 416, 443, 451, 457, 526, 595, 622, 711, 831 |
| Rv1938 | *ephB* | PROBABLE EPOXIDE HYDROLASE EPHB (EPOXIDE HYDRATASE) | 47, 50, 62, 121, 131, 133, 156, 158, 193, 222, 262, 265, 297, 322, 351 |
| Rv1939 | *-* | PROBABLE OXIDOREDUCTASE | 28, 104, 113, 119, 121 |
| Rv1940 | *ribA1* | Probable Riboflavin biosynthesis protein ribA1 (GTP cyclohydrolase II) | 16, 23, 35, 38, 86, 88, 90, 111, 136, 213, 240, 265, 281, 290, 292, 296, 350 |
| Rv1941 | *-* | PROBABLE SHORT-CHAIN TYPE DEHYDROGENASE/REDUCTASE | 6, 17, 43, 46, 77, 80, 89, 130, 148, 150, 158, 167, 183, 193, 225, 240, 248, 252 |
| Rv1942c | *-* | hypothetical protein Rv1942c | 1, 6, 29, 57, 66 |
| Rv1943c | *-* | hypothetical protein Rv1943c | 39, 58 |
| Rv1944c | *-* | hypothetical protein Rv1944c | 13, 23, 25, 50, 54, 68, 82, 88, 121, 149, 173, 184, 193 |
| Rv1945 | *-* | hypothetical protein Rv1945 | 9, 51, 70, 85, 105, 114, 116, 118, 141, 151, 194, 210, 229, 269, 272, 277, 301, 303, 308, 398 |
| Rv1946c | *lppG* | POSSIBLE LIPOPROTEIN | 7, 19, 41, 78, 83 |
| Rv1947 | *-* | hypothetical protein Rv1947 | 8, 42, 43, 128 |
| Rv1948c | *-* | hypothetical protein Rv1948c | 18, 55 |
| Rv1949c | *-* | hypothetical protein Rv1949c | 30, 83, 101, 106, 108, 182, 233, 245, 250, 268, 278, 303 |
| Rv1950c | *-* | hypothetical protein Rv1950c | 49 |
| Rv1951c | *-* | hypothetical protein Rv1951c | 2, 20, 46, 60, 73, 83 |
| Rv1952 | *-* | hypothetical protein Rv1952 | 32, 47 |
| Rv1953 | *-* | hypothetical protein Rv1953 | 10, 19 |
| Rv1954c | *-* | hypothetical protein Rv1954c | 6, 20, 56, 58, 72, 91, 137 |
| Rv1955 | *-* | hypothetical protein Rv1955 | 10, 23, 51, 82, 97 |
| Rv1956 | *-* | POSSIBLE TRANSCRIPTIONAL REGULATORY PROTEIN | 11, 67, 116 |
| Rv1957 | *-* | hypothetical protein Rv1957 | 34, 54, 99, 128, 131, 148, 169 |
| Rv1958c | *-* | hypothetical protein Rv1958c | 39, 50, 51, 79, 96, 174, 182 |
| Rv1959c | *-* | hypothetical protein Rv1959c | 9, 59, 66 |
| Rv1960c | *-* | hypothetical protein Rv1960c | 21, 34, 55, 59 |
| Rv1961 | *-* | hypothetical protein Rv1961 | 24, 89, 114, 116, 136, 157 |
| Rv1962c | *-* | hypothetical protein Rv1962c | 77, 99, 100, 107, 119, 130 |
| Rv1963c | *mce3R* | PROBABLE TRANSCRIPTIONAL REPRESSOR (PROBABLY TETR-FAMILY) MCE3R | 43, 46, 67, 99, 135, 189, 204, 207, 224, 247, 295, 350, 381, 397 |
| Rv1964 | *yrbE3A* | CONSERVED HYPOTHETICAL INTEGRAL MEMBRANE PROTEIN YRBE3A | 8, 85, 89, 91, 116, 165, 189, 214, 227, 230, 255 |
| Rv1965 | *yrbE3B* | CONSERVED HYPOTHETICAL INTEGRAL MEMBRANE PROTEIN YRBE3B | 25, 35, 49, 69, 90, 104, 117, 120, 153, 172, 178, 225, 228, 231 |
| Rv1966 | *mce3A* | MCE-FAMILY PROTEIN MCE3A | 2, 50, 56, 63, 109, 130, 168, 175, 198, 203, 219, 229, 304, 310, 345, 346, 351, 370, 374, 382, 410, 418 |
| Rv1967 | *mce3B* | MCE-FAMILY PROTEIN MCE3B | 59, 82, 114, 115, 122, 168, 181, 246, 248, 273, 277, 322, 331, 335 |
| Rv1968 | *mce3C* | MCE-FAMILY PROTEIN MCE3C | 20, 25, 42, 48, 49, 53, 60, 113, 141, 145, 202, 231, 322, 344, 347, 362, 374, 377, 389, 393, 394, 398, 401 |
| Rv1969 | *mce3D* | MCE-FAMILY PROTEIN MCE3D | 13, 27, 30, 48, 85, 88, 99, 110, 111, 143, 152, 164, 169, 225, 256, 265, 275, 310, 326, 336, 400, 407, 409, 412 |
| Rv1970 | *lprM* | POSSIBLE MCE-FAMILY LIPOPROTEIN LPRM (MCE-FAMILY LIPOPROTEIN MCE3E) | 16, 19, 27, 88, 150, 165, 168, 175, 185, 192, 259, 279, 305, 312, 328, 355 |
| Rv1971 | *mce3F* | MCE-FAMILY PROTEIN MCE3F | 51, 57, 63, 78, 104, 118, 149, 168, 171, 230, 247, 298, 302, 333, 354, 370, 419 |
| Rv1972 | *-* | PROBABLE CONSERVED MCE ASSOCIATED MEMBRANE PROTEIN | 20, 135, 141, 158 |
| Rv1973 | *-* | POSSIBLE CONSERVED MCE ASSOCIATED MEMBRANE PROTEIN | 12, 35, 51, 90, 105, 112, 135 |
| Rv1974 | *-* | PROBABLE CONSERVED MEMBRANE PROTEIN | 12, 13, 22, 57, 107, 116 |
| Rv1975 | *-* | hypothetical protein Rv1975 | 11, 14, 22, 46, 61, 79, 92, 93, 96, 107, 112, 124, 162, 171, 178, 209, 215 |
| Rv1976c | *-* | hypothetical protein Rv1976c | 55, 71, 85, 125 |
| Rv1977 | *-* | hypothetical protein Rv1977 | 4, 22, 26, 29, 139, 164, 185, 199, 209, 231, 257, 265, 266, 273, 294 |
| Rv1978 | *-* | hypothetical protein Rv1978 | 118, 136, 235, 246, 279 |
| Rv1979c | *-* | POSSIBLE CONSERVED PERMEASE | 29, 83, 100, 179, 232, 240, 282, 327, 332, 374 |
| Rv1980c | *mpt64* | IMMUNOGENIC PROTEIN MPT64 (ANTIGEN MPT64/MPB64) | 22, 36, 101, 119, 120, 169, 179, 210, 220 |
| Rv1981c | *nrdF* | ribonucleotide-diphosphate reductase subunit beta | 1, 29, 63, 70, 145, 166, 183, 290, 300, 302 |
| Rv1982c | *-* | hypothetical protein Rv1982c | 23, 26, 46, 72, 76, 105, 128 |
| Rv1983 | *PE_PGRS35* | PE-PGRS FAMILY PROTEIN | 12, 29, 32, 36, 40, 88, 99, 126, 134, 144, 149, 156, 159, 160, 162, 169, 171, 174, 175, 180, 183, 184, 194, 197, 199, 212, 213, 215, 216, 218, 228, 230, 231, 244, 246, 249, 255, 263, 287, 297, 299, 322, 327, 330, 352, 355, 431, 475, 489, 504, 505, 524, 529, 546 |
| Rv1984c | *cfp21* | PROBABLE CUTINASE PRECURSOR CFP21 | 23, 24, 25, 28, 43, 67, 132, 139, 157, 184, 186, 215 |
| Rv1985c | *-* | chromosome replication initiation inhibitor protein | 11, 25, 62, 71, 131, 183, 227, 232, 289 |
| Rv1986 | *-* | PROBABLE CONSERVED INTEGRAL MEMBRANE PROTEIN | 16, 51, 52, 74, 76, 164, 168, 174 |
| Rv1987 | *-* | POSSIBLE CHITINASE | 1, 51, 110, 116, 120, 123 |
| Rv1988 | *-* | PROBABLE METHYLTRANSFERASE | 1, 27, 31, 40, 83, 89, 116, 162, 172 |
| Rv1989c | *-* | hypothetical protein Rv1989c | 15, 28, 36, 61, 79, 148, 155 |
| Rv1990c | *-* | PROBABLE TRANSCRIPTIONAL REGULATORY PROTEIN | 38 |
| Rv1990A | *-* | POSSIBLE DEHYDROGENASE (FRAGMENT) | 41, 98 |
| Rv1991c | *-* | hypothetical protein Rv1991c | 57, 63 |
| Rv1992c | *ctpG* | PROBABLE METAL CATION TRANSPORTER P-TYPE ATPASE G CTPG | 17, 25, 45, 70, 89, 101, 123, 157, 160, 165, 170, 217, 230, 304, 307, 312, 315, 325, 331, 345, 347, 388, 393, 395, 415, 435, 445, 447, 463, 467, 492, 508, 514, 525, 541, 546, 559, 586, 591, 595, 596, 604, 610, 612, 618, 645, 659, 678, 751, 758 |
| Rv1993c | *-* | hypothetical protein Rv1993c | 10, 15, 48, 72, 82 |
| Rv1994c | *-* | PROBABLE TRANSCRIPTIONAL REGULATORY PROTEIN | 60, 106, 108 |
| Rv1995 | *-* | hypothetical protein Rv1995 | 3, 32, 34, 49, 65, 98, 132, 148, 158, 205, 227 |
| Rv1996 | *-* | hypothetical protein Rv1996 | 26, 48, 98, 102, 135, 195, 239, 269, 280, 284, 298 |
| Rv1997 | *ctpF* | PROBABLE METAL CATION TRANSPORTER P-TYPE ATPASE A CTPF | 35, 74, 78, 79, 87, 110, 141, 149, 154, 163, 173, 201, 206, 211, 217, 232, 241, 278, 294, 318, 334, 357, 389, 396, 420, 423, 474, 509, 513, 515, 533, 545, 556, 564, 570, 598, 640, 661, 670, 685, 691, 717, 754, 770, 860, 868, 884 |
| Rv1998c | *-* | hypothetical protein Rv1998c | 44, 55, 63, 97, 112, 116, 159, 192, 209 |
| Rv1999c | *-* | PROBABLE CONSERVED INTEGRAL MEMBRANE PROTEIN | 33, 37, 42, 46, 57, 72, 122, 137, 142, 147, 163, 168, 172, 195, 203, 256, 280, 283, 313, 363, 399, 400, 402, 437 |
| Rv2000 | *-* | hypothetical protein Rv2000 | 81, 96, 115, 174, 229, 253, 256, 292, 313, 326, 327, 379, 383, 394, 418, 426, 517, 518, 528, 530 |
| Rv2001 | *-* | hypothetical protein Rv2001 | 108, 109, 110, 156, 215 |
| Rv2002 | *fabG3* | POSSIBLE 20-BETA-HYDROXYSTEROID DEHYDROGENASE FABG3 (Cortisone reductase) ((R)-20-hydroxysteroid dehydrogenase) | 12, 16, 47, 64, 73, 76, 88, 113, 131, 144, 153, 166, 173, 200, 227, 235 |
| Rv2003c | *-* | hypothetical protein Rv2003c | 15, 61, 62, 77, 87, 133, 168, 181, 191, 205, 217, 252, 259, 261 |
| Rv2004c | *-* | hypothetical protein Rv2004c | 27, 33, 72, 86, 110, 111, 168, 184, 191, 239, 260, 269, 332, 339, 362, 366, 369, 374, 449, 456, 464, 472 |
| Rv2005c | *-* | hypothetical protein Rv2005c | 21, 139, 185, 254, 269, 283 |
| Rv2006 | *otsB1* | PROBABLE TREHALOSE-6-PHOSPHATE PHOSPHATASE OTSB1 (TREHALOSE-PHOSPHATASE) (TPP) | 9, 39, 57, 74, 85, 87, 121, 139, 144, 159, 174, 186, 199, 210, 214, 218, 238, 248, 254, 257, 277, 324, 343, 361, 385, 428, 485, 502, 543, 582, 617, 651, 652, 715, 741, 765, 794, 810, 836, 860, 865, 870, 878, 912, 949, 978, 1009, 1028, 1225, 1249, 1294, 1316 |
| Rv2007c | *fdxA* | PROBABLE FERREDOXIN FDXA | 72, 92 |
| Rv2008c | *-* | hypothetical protein Rv2008c | 21, 46, 59, 76, 81, 93, 118, 171, 180, 191, 213, 238, 257, 310, 422, 429 |
| Rv2009 | *-* | hypothetical protein Rv2009 | 18, 56 |
| Rv2010 | *-* | hypothetical protein Rv2010 | 27, 51, 76, 85, 99, 123 |
| Rv2011c | *-* | hypothetical protein Rv2011c | 52, 73, 121 |
| Rv2012 | *-* | hypothetical protein Rv2012 | 29, 54, 117, 123, 157 |
| Rv2013 | *-* | POSSIBLE TRANSPOSASE | 22, 133, 137, 142 |
| Rv2014 | *-* | POSSIBLE TRANSPOSASE | 6, 10, 13, 18, 29, 64, 69, 102, 113, 126, 136, 173 |
| Rv2015c | *-* | hypothetical protein Rv2015c | 3, 28, 123, 133, 223, 250, 286, 311, 357, 396 |
| Rv2016 | *-* | hypothetical protein Rv2016 | 100, 111, 159 |
| Rv2017 | *-* | POSSIBLE TRANSCRIPTIONAL REGULATORY PROTEIN | 118, 125, 154, 220, 223, 284, 304, 314, 321, 325 |
| Rv2018 | *-* | hypothetical protein Rv2018 | 61, 127, 145, 188, 198, 211 |
| Rv2019 | *-* | hypothetical protein Rv2019 | 31, 59, 62, 117 |
| Rv2020c | *-* | hypothetical protein Rv2020c | 96, 97 |
| Rv2021c | *-* | POSSIBLE TRANSCRIPTIONAL REGULATORY PROTEIN | 69, 80, 83 |
| Rv2022c | *-* | hypothetical protein Rv2022c | 9, 32, 84, 110, 157, 158, 162 |
| Rv2023c | *-* | hypothetical protein Rv2023c | 34, 40, 91 |
| Rv2024c | *-* | hypothetical protein Rv2024c | 19, 59, 70, 74, 97, 180, 195, 197, 202, 211, 215, 238, 328, 358, 376, 403, 430, 433, 455, 457, 461 |
| Rv2025c | *-* | POSSIBLE CONSERVED MEMBRANE PROTEIN | 25, 53, 70, 82, 96, 112, 127, 150, 175, 198, 199, 227, 246, 255, 282, 309 |
| Rv2026c | *-* | hypothetical protein Rv2026c | 133, 148, 223, 243, 264, 268, 277 |
| Rv2027c | *-* | Probable histidine kinase response regulator | 71, 86, 137, 190, 193, 225, 273, 288, 317, 319, 327, 332, 355, 369, 408, 415, 444, 466, 531, 537, 550, 560 |
| Rv2028c | *-* | hypothetical protein Rv2028c | 22, 48, 51, 61, 124, 150, 189, 194, 266 |
| Rv2029c | *pfkB* | Probable phosphofructokinase PfkB (PHOSPHOHEXOKINASE) | 20, 46, 47, 49, 61, 70, 71, 74, 109, 135, 141, 180, 187, 215, 255, 258, 262, 268, 300 |
| Rv2030c | *-* | hypothetical protein Rv2030c | 3, 32, 57, 87, 124, 127, 136, 149, 225, 233, 250, 286, 331, 337, 368, 426, 567, 642, 669, 678 |
| Rv2031c | *hspX* | HEAT SHOCK PROTEIN HSPX (ALPHA-CRSTALLIN HOMOLOG) (14 kDa ANTIGEN) (HSP16.3) | 28, 57 |
| Rv2032 | *acg* | Conserved hypothetical protein Acg | 54, 62, 75, 79, 144, 250, 255, 291 |
| Rv2033c | *-* | hypothetical protein Rv2033c | 10, 12, 35, 59, 65, 77, 85, 92, 154, 182, 185, 211, 241, 276 |
| Rv2034 | *-* | Probable ArsR-type repressor protein | 44, 55, 63 |
| Rv2035 | *-* | hypothetical protein Rv2035 | 60, 108, 111, 142 |
| Rv2036 | *-* | hypothetical protein Rv2036 | 41, 109, 135, 174, 179, 181, 190, 200, 202, 208 |
| Rv2037c | *-* | POSSIBLE CONSERVED TRANSMEMBRANE PROTEIN | 14, 32, 58, 90, 103, 112, 128, 142, 190, 272, 284, 294, 315 |
| Rv2038c | *-* | Probable sugar-transport ATP-binding protein ABC transporter | 39, 136, 137, 170, 178, 284, 286, 311 |
| Rv2039c | *-* | Probable sugar-transport integral membrane protein ABC transporter | 81, 85, 86, 95, 174, 196, 278 |
| Rv2040c | *-* | Probable sugar-transport integral membrane protein ABC transporter | 72, 91, 177, 195, 235, 266 |
| Rv2041c | *-* | Probable sugar-binding lipoprotein | 26, 72, 74, 123, 152, 166, 172, 187, 215, 280, 285, 311, 314, 322, 344, 404, 425 |
| Rv2042c | *-* | hypothetical protein Rv2042c | 19, 25, 32, 75, 98, 109, 140, 155, 157, 162, 181, 188, 218, 226, 228, 242, 252 |
| Rv2043c | *pncA* | PYRAZINAMIDASE/NICOTINAMIDAS PNCA (PZase) | 21, 24, 58, 73, 76, 99, 103, 122, 169, 176 |
| Rv2044c | *-* | hypothetical protein Rv2044c | 10, 11, 36, 55, 71, 76, 77, 81 |
| Rv2045c | *lipT* | Probable carboxylesterase LipT | 21, 138, 191, 209, 217, 241, 259, 268, 311, 361, 440, 442, 447 |
| Rv2046 | *lppI* | Probable lipoprotein lppI | 3, 20, 34, 45, 65, 103, 128, 142, 187, 194 |
| Rv2047c | *-* | hypothetical protein Rv2047c | 5, 8, 13, 16, 52, 82, 92, 94, 102, 146, 147, 177, 183, 195, 294, 324, 341, 342, 353, 398, 444, 445, 513, 517, 521, 570, 582, 590, 668, 707, 716, 731, 736, 741, 746, 762, 776, 782, 790, 796, 830 |
| Rv2048c | *pks12* | Probable polyketide synthase pks12 | 78, 107, 138, 143, 146, 150, 154, 159, 174, 227, 257, 268, 306, 324, 339, 381, 386, 424, 492, 524, 532, 543, 562, 578, 609, 655, 659, 667, 679, 685, 692, 768, 784, 834, 841, 872, 916, 935, 937, 938, 968, 977, 992, 998, 1000, 1042, 1074, 1083, 1086, 1091, 1105, 1128, 1212, 1223, 1239, 1250, 1261, 1331, 1336, 1340, 1384, 1395, 1415, 1427, 1446, 1451, 1461, 1469, 1478, 1499, 1501, 1502, 1522, 1558, 1570, 1598, 1665, 1673, 1684, 1687, 1713, 1718, 1728, 1730, 1766, 1767, 1816, 1829, 1838, 1863, 1864, 1868, 1884, 1893, 1904, 1910, 1940, 1999, 2009, 2011, 2038, 2048, 2068, 2069, 2095, 2100, 2109, 2117, 2118, 2129, 2160, 2165, 2168, 2172, 2176, 2187, 2197, 2250, 2268, 2280, 2286, 2291, 2329, 2347, 2362, 2404, 2409, 2447, 2515, 2547, 2555, 2566, 2585, 2600, 2644, 2681, 2696, 2705, 2714, 2748, 2787, 2803, 2837, 2848, 2890, 2921, 2930, 2952, 2974, 2991, 3006, 3012, 3014, 3029, 3032, 3044, 3056, 3062, 3088, 3116, 3119, 3124, 3263, 3272, 3298, 3314, 3360, 3375, 3392, 3393, 3413, 3421, 3424, 3449, 3476, 3498, 3508, 3517, 3529, 3531, 3532, 3552, 3588, 3600, 3628, 3695, 3703, 3714, 3717, 3743, 3748, 3758, 3760, 3796, 3797, 3846, 3859, 3868, 3893, 3894, 3923, 3934, 3948, 3972, 4031, 4041, 4043, 4058, 4062, 4082, 4118, 4127 |
| Rv2049c | *-* | hypothetical protein Rv2049c | 10, 34, 40, 46, 61, 71 |
| Rv2050 | *-* | hypothetical protein Rv2050 | 50, 109 |
| Rv2051c | *ppm1* | Polyprenol-monophosphomannose synthase Ppm1 | 30, 93, 107, 167, 181, 184, 196, 210, 219, 232, 235, 275, 306, 311, 348, 350, 378, 384, 389, 393, 463, 487, 518, 557, 575, 580, 595, 636, 651, 663, 672, 748, 765, 824 |
| Rv2052c | *-* | hypothetical protein Rv2052c | 22, 27, 69, 71, 76, 82, 123, 134, 144, 149, 162, 171, 180, 203, 205, 214, 219, 257, 263, 276, 313, 314, 329, 357, 361, 382, 441, 454, 459, 475, 483 |
| Rv2053c | *fxsA* | FxsA | 59, 106, 109, 114, 131, 137, 165 |
| Rv2054 | *-* | hypothetical protein Rv2054 | 8, 9, 20, 64, 90, 102, 113, 128, 172, 178, 187, 196, 211, 230 |
| Rv2055c | *rpsR* | 30S ribosomal protein S18 | 1, 4, 30, 49 |
| Rv2056c | *rpsN* | 30S ribosomal protein S14 | 24, 42, 70, 93 |
| Rv2057c | *rpmG* | 50S ribosomal protein L33 | 17 |
| Rv2058c | *rpmB* | 50S ribosomal protein L28 | 1, 7 |
| Rv2059 | *-* | hypothetical protein Rv2059 | 8, 13, 17, 64, 100, 118, 119, 129, 142, 145, 158, 170, 230, 235, 254, 270, 303, 318, 327, 344 |
| Rv2060 | *-* | Possible conserved integral membrane protein | 30, 32, 44, 51, 70, 74, 83, 95, 98 |
| Rv2061c | *-* | hypothetical protein Rv2061c | 31, 37, 69, 80, 114, 132 |
| Rv2062c | *cobN* | cobaltochelatase | 21, 54, 61, 68, 69, 77, 81, 94, 96, 107, 126, 147, 150, 165, 168, 181, 182, 195, 216, 217, 223, 227, 228, 327, 368, 393, 407, 426, 441, 459, 474, 497, 504, 538, 542, 558, 603, 607, 611, 623, 691, 697, 733, 738, 744, 749, 756, 767, 785, 793, 801, 821, 857, 904, 931, 968, 990, 993, 1020, 1042, 1057, 1093, 1119, 1120, 1164, 1166 |
| Rv2063 | *-* | hypothetical protein Rv2063 | 17, 35 |
| Rv2064 | *cobG* | Possible cobalamin biosynthesis protein CobG | 10, 18, 29, 30, 34, 50, 56, 58, 64, 79, 99, 102, 103, 129, 144, 164, 168, 192, 207, 237, 245, 253, 279, 304, 307, 313, 320, 329, 341, 346, 353, 354 |
| Rv2065 | *cobH* | precorrin-8X methylmutase | 7, 28, 42, 66, 67, 79, 80, 88, 106, 116, 126, 137, 158, 178, 186, 189, 195, 197 |
| Rv2066 | *cobI* | Probable bifunctional protein, CobI-CobJ fusion protein: S-adenosyl-L-methionine-precorrin-2 methyl transferase + precorrin-3 methylase | 13, 23, 46, 55, 74, 75, 78, 98, 133, 140, 151, 197, 211, 212, 219, 237, 245, 255, 270, 279, 302, 323, 333, 354, 387, 391, 394, 438, 442, 458, 498 |
| Rv2067c | *-* | hypothetical protein Rv2067c | 61, 63, 128, 241, 242, 315, 327, 330, 345, 364, 374, 403 |
| Rv2068c | *blaC* | CLASS A BETA-LACTAMASE BLAC | 158, 189, 231, 241, 250, 252, 268, 281, 282, 302 |
| Rv2069 | *sigC* | RNA polymerase sigma factor SigC | 1, 11, 68, 100, 115, 151, 155, 183 |
| Rv2070c | *cobK* | cobalt-precorrin-6x reductase | 7, 9, 46, 49, 55, 78, 81, 85, 102, 104, 130, 151, 156, 168, 193, 204, 233 |
| Rv2071c | *cobM* | Probable precorrin-4 C11-methyltransferase CobM | 9, 11, 18, 46, 53, 80, 113, 115, 146, 151, 165, 202, 209, 220, 230 |
| Rv2072c | *cobL* | Probable precorrin-6y methyltransferase CobL | 12, 43, 55, 72, 100, 123, 128, 132, 140, 158, 170, 239, 265, 283, 293, 303, 312, 329, 331, 345, 370, 373 |
| Rv2073c | *-* | Probable shortchain dehydrogenase | 12, 18, 27, 34, 45, 67, 85, 130, 131, 159, 166, 175, 197, 208, 211 |
| Rv2074 | *-* | hypothetical protein Rv2074 | 55, 56, 129 |
| Rv2075c | *-* | Possible hypothetical exported or envelope protein | 21, 26, 33, 39, 87, 100, 172, 209, 278, 294, 300, 310, 324, 368, 399, 401, 418, 443, 472, 474, 475 |
| Rv2076c | *-* | hypothetical protein Rv2076c | 6, 22, 63 |
| Rv2077c | *-* | POSSIBLE CONSERVED TRANSMEMBRANE PROTEIN | 19, 25, 48, 82, 102, 125, 135, 153, 157, 160, 201, 220, 242, 288, 294, 304, 310 |
| Rv2077A | *-* | hypothetical protein Rv2077A | 27, 35, 69, 73, 90 |
| Rv2078 | *-* | hypothetical protein Rv2078 | 17, 79, 91 |
| Rv2079 | *-* | hypothetical protein Rv2079 | 14, 15, 47, 77, 84, 116, 146, 180, 226, 304, 305, 346, 351, 360, 364, 369, 377, 396, 403, 472, 493, 546, 547, 590, 609 |
| Rv2080 | *lppJ* | Possible lipoprotein lppJ | 4, 18, 37, 47, 69, 116, 144 |
| Rv2081c | *-* | POSSIBLE TRANSMEMBRANE PROTEIN | 4, 14, 68, 76, 85 |
| Rv2082 | *-* | hypothetical protein Rv2082 | 6, 27, 60, 70, 109, 119, 148, 183, 190, 221, 247, 266, 283, 294, 319, 327, 329, 365, 369, 416, 419, 424, 445, 449, 451, 458, 461, 473, 493, 498, 500, 502, 509, 527, 533, 550, 560, 587, 604, 607, 645, 712 |
| Rv2083 | *-* | hypothetical protein Rv2083 | 14, 29, 71, 102, 110, 126, 163, 167, 171, 176, 180, 185, 202, 215, 216, 228, 245, 256, 311 |
| Rv2084 | *-* | hypothetical protein Rv2084 | 20, 28, 112, 129, 153, 199, 275, 281, 287, 289, 307, 311, 372 |
| Rv2085 | *-* | hypothetical protein Rv2085 | 12, 36, 58, 62, 65 |
| Rv2086 | *-* | hypothetical protein Rv2086 | 2, 62, 110, 125, 127, 134, 148, 175 |
| Rv2087 | *-* | hypothetical protein Rv2087 | 2, 39, 69 |
| Rv2088 | *pknJ* | PROBABLE TRANSMEMBRANE SERINE/THREONINE-PROTEIN KINASE J PKNJ (PROTEIN KINASE J) (STPK J) | 11, 22, 65, 79, 95, 146, 148, 172, 210, 216, 217, 273, 291, 315, 321, 353, 399, 435, 468, 502, 552, 570, 587 |
| Rv2089c | *pepE* | Probable dipeptidase PepE | 24, 33, 52, 59, 72, 77, 104, 110, 157, 182, 204, 231, 284, 293, 303, 319, 327 |
| Rv2090 | *-* | Probable 5'-3' exonuclease | 1, 100, 111, 218, 241, 265, 284, 293, 310, 328, 335, 367, 391 |
| Rv2091c | *-* | Probable membrane protein | 1, 15, 28, 69, 87, 108, 114, 118, 139, 161, 176 |
| Rv2092c | *helY* | PROBABLE ATP-DEPENDENT DNA HELICASE HELY | 10, 30, 40, 58, 92, 172, 251, 257, 282, 286, 315, 337, 340, 359, 360, 408, 411, 414, 491, 508, 548, 552, 563, 632, 639, 646, 647, 670, 697, 776, 837, 845, 852, 887, 897, 904 |
| Rv2093c | *tatC* | Probable Sec-independent protein translocase transmembrane protein tatC | 2, 3, 42, 95, 110, 111, 153, 179, 226, 241, 249 |
| Rv2094c | *tatA* | twin argininte translocase protein A |  |
| Rv2095c | *-* | hypothetical protein Rv2095c | 28, 35, 65, 89, 102, 118, 121, 125, 137, 143, 157, 177, 203, 209, 301, 304 |
| Rv2096c | *-* | hypothetical protein Rv2096c | 111, 122, 123, 132, 148, 163, 226, 233, 250, 264, 269, 272, 273, 301, 312, 327 |
| Rv2097c | *-* | hypothetical protein Rv2097c | 65, 80, 102, 130, 151, 153, 162, 225, 259, 267, 361, 363, 375, 400, 406 |
| Rv2100 | *-* | hypothetical protein Rv2100 | 1, 10, 31, 59, 83, 87, 104, 119, 140, 169, 175, 199, 223, 225, 237, 271, 272, 279, 281, 288, 290, 296, 297, 319, 347, 358, 359, 364, 384, 413, 418, 470, 481, 494, 510, 522 |
| Rv2101 | *helZ* | PROBABLE HELICASE HELZ | 10, 11, 33, 42, 47, 54, 56, 82, 102, 106, 111, 152, 201, 231, 285, 307, 349, 353, 370, 397, 463, 473, 478, 485, 499, 512, 514, 529, 538, 582, 642, 689, 800, 805, 817, 831, 869, 875, 900, 914, 916, 921, 962 |
| Rv2102 | *-* | hypothetical protein Rv2102 | 29, 33, 77, 89, 97, 99, 120, 153, 208, 213, 223, 236 |
| Rv2103c | *-* | hypothetical protein Rv2103c | 74, 100, 111, 140 |
| Rv2104c | *-* | hypothetical protein Rv2104c | 38, 81 |
| Rv2105 | *-* | PROBABLE TRANSPOSASE | 24, 60, 64, 71, 93 |
| Rv2106 | *-* | PROBABLE TRANSPOSASE | 68, 105, 109, 125, 259, 273, 289, 309, 310 |
| Rv2107 | *PE22* | PE FAMILY PROTEIN | 29, 65 |
| Rv2108 | *PPE36* | PPE FAMILY PROTEIN | 26, 78, 85 |
| Rv2109c | *prcA* | proteasome (alpha subunit) PrcA | 35, 75, 84, 189, 225, 227 |
| Rv2110c | *prcB* | proteasome (beta subunit) PrcB | 17, 41, 48, 65, 66, 97, 99, 133, 181, 182, 183, 230, 235, 237, 238, 287 |
| Rv2111c | *-* | hypothetical protein Rv2111c | 8, 10, 18, 21, 61 |
| Rv2112c | *-* | hypothetical protein Rv2112c | 4, 10, 23, 29, 42, 68, 83, 85, 86, 116, 150, 173, 182, 184, 220, 233, 247, 270, 361, 459, 487, 499, 511, 549 |
| Rv2113 | *-* | Probable integral membrane protein | 49, 67, 106, 122, 129, 137, 148, 150, 197, 216, 246, 258, 290, 369, 383, 387, 391 |
| Rv2114 | *-* | hypothetical protein Rv2114 | 7, 10, 22, 25, 46, 61, 89, 124, 168, 205 |
| Rv2115c | *-* | Probable ATPase | 22, 151, 215, 225, 254, 294, 317, 381, 427, 514, 536, 588 |
| Rv2116 | *lppK* | Probable conserved lipoprotein lppK | 10, 20, 47, 54, 88, 96, 150, 158 |
| Rv2117 | *-* | hypothetical protein Rv2117 | 39, 42, 59, 77 |
| Rv2118c | *-* | POSSIBLE RNA METHYLTRANSFERASE | 3, 28, 77, 98, 107, 109, 123, 144, 194, 195, 248, 251 |
| Rv2119 | *-* | hypothetical protein Rv2119 | 52, 61, 83, 89, 94, 109, 140, 149, 151, 191, 234, 262 |
| Rv2120c | *-* | PROBABLE CONSERVED INTEGRAL MEMBRANE PROTEIN | 16, 30, 90, 96, 103, 118, 131, 148, 154 |
| Rv2121c | *hisG* | ATP phosphoribosyltransferase | 22, 66, 74, 94, 96, 97, 107, 113, 184 |
| Rv2122c | *hisE* | phosphoribosyl-ATP pyrophosphatase | 35, 48 |
| Rv2123 | *PPE37* | PPE FAMILY PROTEIN | 19, 23, 28, 37, 86, 89, 95, 97, 101, 149, 235, 267, 270, 282, 303, 319, 321, 326, 335, 339, 364, 370, 374, 378, 422, 423, 434, 442, 446, 452 |
| Rv2124c | *metH* | Probable 5-methyltetrahydrofolate--homocystein methyltransferase MetH (Methionine synthase, vitamin-B12 dependent isozyme) (MS) | 33, 42, 103, 150, 157, 187, 202, 258, 282, 297, 309, 332, 421, 479, 495, 516, 557, 682, 693, 817, 859, 891, 896, 935, 951, 954, 989, 1028, 1109, 1131 |
| Rv2125 | *-* | hypothetical protein Rv2125 | 27, 31, 88, 98, 149, 167, 170, 180, 225, 258, 275 |
| Rv2126c | *PE_PGRS37* | PE-PGRS FAMILY PROTEIN | 7, 12, 23, 30, 31, 37, 47, 49, 50, 52, 56, 61, 64, 65, 71, 89, 98, 102, 107, 108, 111, 114, 116, 117, 124, 127, 129, 130, 133, 135, 136, 139, 144, 145, 147, 149, 165, 169, 177, 178, 180, 184, 186, 190, 193, 208, 212, 214, 215, 217, 221, 227, 239, 248, 254 |
| Rv2127 | *ansP1* | Probable L-asparagine permease ansP1 | 14, 31, 36, 42, 49, 60, 96, 117, 163, 183, 193, 202, 233, 236, 272, 276, 293, 303, 316, 326, 333, 339, 344, 349, 364, 375, 399, 415, 452, 473 |
| Rv2128 | *-* | PROBABLE CONSERVED TRANSMEMBRANE PROTEIN | 34, 48 |
| Rv2129c | *-* | short chain dehydrogenase | 11, 66, 70, 80, 89, 125, 131, 141, 147, 187, 222, 276 |
| Rv2130c | *cysS* | cysteinyl-tRNA synthetase | 33, 42, 145, 190, 196, 209, 212, 248, 269, 281, 295, 320, 338, 346, 348, 351, 352, 391, 395 |
| Rv2131c | *cysQ* | POSSIBLE MONOPHOSPHATASE CYSQ | 4, 18, 47, 126, 145, 147, 150, 204, 212, 221, 222 |
| Rv2132 | *-* | hypothetical protein Rv2132 | 11, 35, 74 |
| Rv2133c | *-* | hypothetical protein Rv2133c | 53, 93, 103, 112, 130, 161, 166, 195, 208, 215, 223, 248 |
| Rv2134c | *-* | hypothetical protein Rv2134c | 15, 21, 141 |
| Rv2135c | *-* | hypothetical protein Rv2135c | 14, 19, 22, 35, 113, 114, 174, 198, 211, 225, 231 |
| Rv2136c | *uppP* | undecaprenyl pyrophosphate phosphatase | 9, 35, 38, 43, 52, 119, 125, 162, 167, 184, 199, 235, 269, 273 |
| Rv2137c | *-* | hypothetical protein Rv2137c | 38, 62, 92, 130 |
| Rv2138 | *lppL* | Probable conserved lipoprotein LppL | 35, 43, 57, 64, 91, 114, 127, 129, 139, 142, 156, 182, 188, 190, 238, 246, 287, 298, 310, 343, 345 |
| Rv2139 | *pyrD* | dihydroorotate dehydrogenase 2 | 25, 28, 64, 72, 91, 96, 151, 234, 258, 259, 291, 314, 319, 338 |
| Rv2140c | *TB18.6* | hypothetical protein Rv2140c | 8, 35, 41, 74, 103, 112, 124 |
| Rv2141c | *-* | hypothetical protein Rv2141c | 4, 32, 61, 64, 66, 80, 105, 138, 157, 184, 205, 218, 234, 267, 299, 307, 317, 367, 371, 388 |
| Rv2142c | *-* | hypothetical protein Rv2142c |  |
| Rv2143 | *-* | hypothetical protein Rv2143 | 6, 97, 102, 156, 168, 210, 248, 270, 284 |
| Rv2144c | *-* | Probable transmembrane protein | 86 |
| Rv2145c | *wag31* | CONSERVED HYPOTHETICAL PROTEIN WAG31 | 60, 62, 65, 112, 119, 145, 248 |
| Rv2146c | *-* | Possible conserved transmembrane protein | 37 |
| Rv2147c | *-* | hypothetical protein Rv2147c | 53, 80, 84, 96, 129, 196, 203, 234 |
| Rv2148c | *-* | hypothetical protein Rv2148c | 28, 107, 116, 127, 161, 168, 246 |
| Rv2149c | *yfiH* | conserved hypothetical protein YfiH | 9, 49, 59, 65, 99, 110, 123, 126, 131, 138, 164, 174, 192, 194, 197, 203, 239 |
| Rv2150c | *ftsZ* | cell division protein FtsZ | 18, 63, 67, 90, 98, 99, 103, 105, 107, 171, 215, 217, 219, 261, 262, 281, 309, 314, 316, 325, 326, 364 |
| Rv2151c | *ftsQ* | POSSIBLE CELL DIVISION PROTEIN FTSQ | 16, 56, 62, 76, 85, 89, 144, 230, 245 |
| Rv2152c | *murC* | UDP-N-acetylmuramate--L-alanine ligase | 18, 32, 37, 45, 70, 81, 96, 112, 120, 139, 148, 154, 217, 228, 231, 293, 307, 323, 338, 420, 423, 449, 451, 463, 475, 486 |
| Rv2153c | *murG* | N-acetylglucosaminyl transferase | 8, 9, 11, 18, 21, 40, 41, 44, 45, 50, 68, 84, 100, 101, 144, 146, 179, 190, 207, 248, 250, 255, 257, 297, 306, 315, 343, 344, 352, 364, 370, 374, 377, 388, 395, 401, 403 |
| Rv2154c | *ftsW* | FtsW-like protein FtsW | 63, 106, 124, 149, 178, 197, 246, 252, 273, 338, 346, 355, 380, 388, 389, 395, 416, 461, 470, 483 |
| Rv2155c | *murD* | UDP-N-acetylmuramoyl-L-alanyl-D-glutamate synthetase | 13, 18, 64, 73, 77, 85, 118, 136, 144, 181, 202, 210, 211, 216, 224, 244, 285, 296, 297, 336, 351, 428, 438, 442, 450, 459, 467, 478 |
| Rv2156c | *mraY* | phospho-N-acetylmuramoyl-pentapeptide-transferase | 46, 52, 58, 91, 94, 125, 140, 190, 191, 198, 235, 237, 249, 251, 265, 305, 337, 341, 354 |
| Rv2157c | *murF* | Probable UDP-N-acetylmuramoylalanyl-D-glutamyl-2,6-diaminopimelate- D-alanyl-D-alanyl ligase MurF | 24, 30, 43, 60, 68, 73, 88, 94, 110, 117, 120, 125, 126, 134, 147, 160, 196, 214, 240, 250, 259, 271, 305, 316, 332, 335, 350, 370, 426, 466, 477, 489, 504 |
| Rv2158c | *murE* | UDP-N-acetylmuramoylalanyl-D-glutamate--2,6-diaminopimelate ligase | 36, 62, 73, 78, 81, 97, 106, 107, 113, 124, 127, 134, 151, 154, 165, 170, 174, 185, 211, 275, 284, 292, 300, 304, 318, 323, 327, 328, 382, 419, 420, 435, 469, 493, 500, 506, 509, 511, 525 |
| Rv2159c | *-* | hypothetical protein Rv2159c | 14, 15, 47, 48, 71, 99, 105, 111, 125, 140, 146, 179, 221, 224, 232, 267, 271, 284, 297, 319 |
| Rv2160A | *-* | hypothetical protein Rv2160A | 16, 35, 62, 74, 80 |
| Rv2160c | *-* | hypothetical protein Rv2160c | 9, 10, 16, 18, 33, 35, 38, 41, 88 |
| Rv2161c | *-* | hypothetical protein Rv2161c | 25, 47, 66, 68, 79, 103, 139, 213, 218, 224, 233, 245, 264, 277 |
| Rv2162c | *PE_PGRS38* | PE-PGRS FAMILY PROTEIN | 13, 26, 41, 81, 91, 123, 129, 130, 140, 142, 145, 150, 153, 162, 164, 171, 177, 180, 189, 193, 197, 198, 205, 207, 208, 210, 211, 217, 219, 220, 222, 223, 227, 232, 233, 235, 241, 249, 251, 252, 263, 267, 270, 272, 273, 275, 276, 284, 285, 288, 291, 296, 299, 305, 306, 308, 314, 320, 328, 331, 332, 334, 337, 340, 343, 353, 356, 358, 359, 375, 376, 378, 381, 382, 385, 392, 395, 397, 401, 406, 409, 413, 416, 418, 419, 422, 427, 429, 431, 434, 437, 446, 449, 451, 452, 461, 463, 467, 470, 487, 496, 499, 501, 502, 513, 514 |
| Rv2163c | *pbpB* | Probable penicillin-binding membrane protein pbpB | 3, 18, 35, 60, 79, 89, 113, 121, 134, 190, 199, 276, 294, 346, 391, 393, 416, 470, 475, 479, 510, 554, 577, 581, 584, 589, 592, 594, 602, 615, 646, 669 |
| Rv2164c | *-* | PROBABLE CONSERVED PROLINE RICH MEMBRANE PROTEIN | 22, 42, 48, 73, 104, 145, 197, 239, 257, 260, 279, 288, 293, 333, 345 |
| Rv2165c | *mraW* | S-adenosyl-methyltransferase MraW | 8, 26, 48, 71, 104, 107, 152, 183, 197, 216, 244, 289 |
| Rv2166c | *-* | hypothetical protein Rv2166c | 72, 73, 131 |
| Rv2167c | *-* | PROBABLE TRANSPOSASE | 102, 139, 143, 159, 293, 307, 323, 343, 344 |
| Rv2168c | *-* | PROBABLE TRANSPOSASE | 24, 60, 64, 71, 93 |
| Rv2169c | *-* | PROBABLE CONSERVED TRANSMEMBRANE PROTEIN | 30, 37, 57, 89, 94, 100, 115 |
| Rv2170 | *-* | hypothetical protein Rv2170 | 48, 66, 75, 91, 94, 109, 150, 185, 190 |
| Rv2171 | *lppM* | Probable conserved lipoprotein lppM | 6, 22, 40, 45, 55, 104, 105, 143, 186, 188, 195, 199, 200, 218 |
| Rv2172c | *-* | hypothetical protein Rv2172c | 35, 88, 106, 126, 224, 237, 241, 278 |
| Rv2173 | *idsA2* | PROBABLE GERANYLGERANYL PYROPHOSPHATE SYNTHETASE IDSA2 (GGPPSASE) (GGPP SYNTHETASE) (GERANYLGERANYL DIPHOSPHATE SYNTHASE) | 1, 19, 39, 70, 89, 97, 144, 167, 179, 194, 208, 251, 309, 316, 328, 337, 350 |
| Rv2174 | *-* | Possible conserved integral membrane protein | 30, 34, 42, 48, 50, 54, 105, 175, 199, 210, 232, 249, 262, 263, 289, 290, 320, 339, 347, 350, 363, 413, 416, 430, 458, 486, 503 |
| Rv2175c | *-* | conserved hypothetical regulatory protein | 16, 73 |
| Rv2176 | *pknL* | PROBABLE TRANSMEMBRANE SERINE/THREONINE-PROTEIN KINASE L PKNL (PROTEIN KINASE L) (STPK L) | 26, 100, 127, 131, 135, 167, 174, 180, 218, 294, 329, 347, 351, 380, 385, 395 |
| Rv2177c | *-* | POSSIBLE TRANSPOSASE | 104, 129, 154, 178, 219 |
| Rv2178c | *aroG* | Probable 3-deoxy-D-arabino-heptulosonate 7-phosphate synthase AroG (DAHP synthetase, phenylalanine-repressible) | 27, 83, 127, 147, 158, 209, 239, 274, 356, 405, 412, 432 |
| Rv2179c | *-* | hypothetical protein Rv2179c | 42, 57, 163 |
| Rv2180c | *-* | Probable conserved integral membrane protein | 43, 77, 104, 191, 254, 275, 282, 288 |
| Rv2181 | *-* | Probable conserved integral membrane protein | 25, 64, 121, 133, 188, 202, 217, 257, 285, 304, 358, 401, 406, 417 |
| Rv2182c | *-* | 1-acylglycerol-3-phosphate O-acyltransferase | 67, 69, 83, 95, 132, 141, 186, 216, 226, 228, 237, 244 |
| Rv2183c | *-* | hypothetical protein Rv2183c | 1, 24, 38, 40, 58, 99, 103, 107 |
| Rv2184c | *-* | hypothetical protein Rv2184c | 21, 36, 38, 84, 124, 190, 266, 276, 302, 321 |
| Rv2185c | *TB16.3* | hypothetical protein Rv2185c |  |
| Rv2186c | *-* | hypothetical protein Rv2186c | 15, 20, 53, 57, 82 |
| Rv2187 | *fadD15* | Probable long-chain-fatty-acid-CoA ligase fadD15 (FATTY-ACID-CoA SYNTHETASE) (FATTY-ACID-CoA SYNTHASE) | 6, 56, 68, 135, 139, 148, 171, 192, 243, 285, 290, 293, 302, 305, 315, 347, 350, 351, 364, 366, 377, 401, 416, 422, 442, 457, 466, 468, 474, 570 |
| Rv2188c | *-* | hypothetical protein Rv2188c | 14, 56, 95, 161, 228, 234, 258, 259, 267, 283, 285, 300, 302, 308, 312, 354, 355, 370, 378 |
| Rv2189c | *-* | hypothetical protein Rv2189c | 51, 72, 73, 81, 84, 95, 170, 209, 213, 233, 243 |
| Rv2190c | *-* | hypothetical protein Rv2190c | 15, 27, 31, 76, 107, 153, 174, 180, 229, 238, 258, 269, 292, 294, 295, 335 |
| Rv2191 | *-* | hypothetical protein Rv2191 | 11, 74, 100, 124, 143, 179, 255, 266, 308, 367, 390, 395, 407, 413, 428, 433, 436, 463, 478, 485, 494, 507, 509, 514, 527, 540, 568, 578, 594, 621 |
| Rv2192c | *trpD* | anthranilate phosphoribosyltransferase | 12, 53, 62, 74, 93, 107, 144, 189, 230, 275, 276, 298, 319, 320, 327, 352 |
| Rv2193 | *ctaE* | PROBABLE CYTOCHROME C OXIDASE (SUBUNIT III) CTAE | 53, 54, 92, 143, 151, 152 |
| Rv2194 | *qcrC* | Probable Ubiquinol-cytochrome C reductase QcrC(cytochrome C subunit) | 8, 25, 26, 37, 63, 96, 104, 111, 139, 164, 180, 183, 188, 197, 205, 243, 249, 253, 267 |
| Rv2195 | *qcrA* | Probable Rieske iron-sulfur protein QcrA | 14, 27, 42, 51, 54, 65, 236, 264, 282 |
| Rv2196 | *qcrB* | Probable Ubiquinol-cytochrome C reductase QcrB (cytochrome B subunit) | 60, 95, 104, 116, 134, 199, 203, 243, 265, 280, 332, 349, 359, 378, 451, 498, 511, 524, 531 |
| Rv2197c | *-* | Probable conserved transmembrane protein | 5, 28, 35, 40, 56, 72, 94, 105, 111, 148, 186, 190 |
| Rv2198c | *mmpS3* | PROBABLE CONSERVED MEMBRANE PROTEIN MMPS3 | 1, 27, 35, 37, 65, 66, 93, 104, 108, 130, 208, 218, 234, 235 |
| Rv2199c | *-* | Possible conserved integral membrane protein | 11, 30, 44, 45, 97, 114, 115, 125 |
| Rv2200c | *ctaC* | PROBABLE TRANSMEMBRANE CYTOCHROME C OXIDASE (SUBUNIT II) CTAC | 3, 18, 20, 92, 207, 238, 287, 353 |
| Rv2201 | *asnB* | Probable asparagine synthetase AsnB | 11, 35, 50, 120, 134, 165, 167, 168, 284, 308, 344, 390, 427, 434, 451, 460, 480, 498, 556, 577, 593, 600, 611 |
| Rv2202c | *cbhK* | Probable carbohydrate kinase CbhK | 5, 45, 49, 83, 95, 117, 166, 205, 252, 260, 268, 290, 301, 315 |
| Rv2203 | *-* | POSSIBLE CONSERVED MEMBRANE PROTEIN | 1, 8, 37, 54, 58, 114, 137, 194, 195, 196, 200, 213, 225 |
| Rv2204c | *-* | hypothetical protein Rv2204c | 19, 65, 107, 112 |
| Rv2205c | *-* | hypothetical protein Rv2205c | 24, 43, 64, 83, 98, 105, 119, 124, 150, 166, 212, 213, 215, 225, 226, 237, 265, 298, 306, 315, 323, 351 |
| Rv2206 | *-* | PROBABLE CONSERVED TRANSMEMBRANE PROTEIN | 20, 30, 34, 40, 45, 85, 114, 211, 212, 230 |
| Rv2207 | *cobT* | nicotinate-nucleotide--dimethylbenzimidazole phosphoribosyltransferase | 16, 62, 70, 80, 90, 128, 130, 144, 146, 150, 161, 168, 177, 179, 199, 204, 211, 234, 235, 240, 249, 265, 275, 315, 317, 326, 340, 353 |
| Rv2208 | *cobS* | cobalamin synthase | 30, 61, 84, 106, 142, 146, 168, 172, 190, 207, 220, 223 |
| Rv2209 | *-* | Probable conserved integral membrane protein | 1, 28, 34, 77, 110, 119, 124, 140, 153, 160, 187, 193, 210, 257, 303, 334, 340, 378, 400, 451, 463, 481, 484, 506 |
| Rv2210c | *ilvE* | branched-chain amino acid aminotransferase | 2, 98, 134, 136, 137, 172, 178, 197, 199, 205, 209, 218, 251, 263, 268, 282, 300, 302, 311, 313, 349 |
| Rv2211c | *gcvT* | glycine cleavage system aminomethyltransferase T | 38, 47, 58, 84, 95, 107, 108, 130, 133, 143, 146, 172, 200, 203, 230, 232, 235, 236, 291, 305, 310, 346, 359 |
| Rv2212 | *-* | hypothetical protein Rv2212 | 11, 12, 21, 43, 50, 57, 66, 144, 206, 217, 225, 237, 290, 318, 326, 373 |
| Rv2213 | *pepB* | leucyl aminopeptidase | 34, 40, 69, 108, 109, 112, 157, 162, 188, 192, 206, 235, 238, 284, 285, 303, 306, 389, 414, 458, 478, 484, 496 |
| Rv2214c | *ephD* | short chain dehydrogenase | 95, 118, 131, 144, 213, 221, 233, 291, 318, 329, 333, 339, 363, 368, 408, 433, 452, 462, 493, 494, 503, 514, 519, 553, 585 |
| Rv2215 | *dlaT* | dihydrolipoamide acetyltransferase | 51, 69, 81, 84, 112, 188, 202, 204, 208, 224, 234, 259, 262, 264, 291, 294, 296, 327, 428, 434, 458, 459, 489, 503, 530 |
| Rv2216 | *-* | hypothetical protein Rv2216 | 19, 49, 96, 102, 144, 152, 161, 189, 216, 222, 234, 274, 279, 299 |
| Rv2217 | *lipB* | lipoyltransferase | 9, 23, 37, 38, 49, 53, 54, 75, 123, 130, 135, 170, 175, 203, 208 |
| Rv2218 | *lipA* | lipoyl synthase | 57, 76, 91, 94, 120, 130, 136, 149, 171, 204, 209, 230, 238, 280 |
| Rv2219 | *-* | PROBABLE CONSERVED TRANSMEMBRANE PROTEIN | 6, 11, 23, 68, 107, 110, 125, 128, 130, 143, 167, 223 |
| Rv2219A | *-* | PROBABLE CONSERVED MEMBRANE PROTEIN | 9, 18, 81 |
| Rv2220 | *glnA1* | GLUTAMINE SYNTHETASE GLNA1 (GLUTAMINE SYNTHASE) (GS-I) | 58, 126, 163, 183, 208, 221, 237, 251, 272, 295, 306, 331, 355, 412 |
| Rv2221c | *glnE* | GLUTAMATE-AMMONIA-LIGASE ADENYLYLTRANSFERASE GLNE (Glutamine-synthetase adenylyltransferase) | 57, 71, 87, 160, 165, 180, 189, 203, 219, 263, 315, 346, 358, 397, 409, 460, 493, 505, 511, 520, 532, 535, 558, 584, 628, 650, 740, 794, 816, 840, 861, 873, 895, 911, 916, 929, 941, 951, 962 |
| Rv2222c | *glnA2* | PROBABLE GLUTAMINE SYNTHETASE GLNA2 (GLUTAMINE SYNTHASE) (GS-II) | 84, 119, 139, 153, 163, 191, 275, 301, 307, 312, 316, 354 |
| Rv2223c | *-* | Probable exported protease | 21, 31, 37, 39, 84, 97, 113, 122, 126, 176, 206, 238, 276, 282, 355, 359, 404, 422, 424, 443, 447, 467 |
| Rv2224c | *-* | Probable exported protease | 18, 21, 64, 65, 107, 110, 113, 117, 141, 234, 278, 325, 360, 383, 424, 449, 469, 478, 509, 515 |
| Rv2225 | *panB* | 3-methyl-2-oxobutanoate hydroxymethyltransferase | 11, 14, 52, 111, 115, 124, 133, 145, 166, 173, 176, 190, 201, 209, 236, 239, 243, 269, 270, 274 |
| Rv2226 | *-* | hypothetical protein Rv2226 | 32, 68, 69, 73, 81, 94, 135, 149, 153, 158, 184, 200, 202, 205, 220, 326, 343, 395, 401, 402, 428, 467, 470, 471, 498 |
| Rv2227 | *-* | hypothetical protein Rv2227 | 114, 115, 161, 162, 179, 218 |
| Rv2228c | *-* | hypothetical protein Rv2228c | 8, 14, 17, 25, 50, 54, 67, 81, 131, 154, 157, 160, 185, 202, 208, 225, 228, 326 |
| Rv2229c | *-* | hypothetical protein Rv2229c | 2, 45, 131, 145, 189, 191, 195, 212, 232 |
| Rv2230c | *-* | hypothetical protein Rv2230c | 30, 57, 90, 96, 128, 155, 157, 173, 184, 205, 218, 239, 262, 269, 273, 274, 287, 290, 299, 340, 343 |
| Rv2231c | *cobC* | hypothetical protein Rv2231c | 8, 25, 37, 60, 119, 131, 148, 197, 224, 268, 270, 286, 326 |
| Rv2232 | *-* | hypothetical protein Rv2232 | 8, 37, 54, 56, 66, 94, 137, 152, 172, 173, 187, 248, 278 |
| Rv2234 | *ptpA* | PHOSPHOTYROSINE PROTEIN PHOSPHATASE PTPA (PROTEIN-TYROSINE-PHOSPHATASE) (PTPase) (LMW PHOSPHATASE) | 44, 58, 72, 103, 143, 146 |
| Rv2235 | *-* | PROBABLE CONSERVED TRANSMEMBRANE PROTEIN | 9, 80, 112, 145, 174, 178, 181, 197, 198, 210 |
| Rv2236c | *cobD* | cobalamin biosynthesis protein | 38, 52, 75, 84, 93, 100, 124, 132, 139, 148, 162, 225, 235, 243 |
| Rv2237 | *-* | hypothetical protein Rv2237 | 27, 41, 114, 165, 181 |
| Rv2238c | *ahpE* | Probable peroxiredoxin AhpE | 6, 23, 64, 83 |
| Rv2239c | *-* | hypothetical protein Rv2239c | 9, 60, 111, 114, 119 |
| Rv2240c | *-* | hypothetical protein Rv2240c | 18, 128, 177, 185, 209, 212, 262 |
| Rv2241 | *aceE* | pyruvate dehydrogenase subunit E1 | 26, 42, 70, 111, 124, 130, 156, 171, 214, 269, 283, 285, 330, 362, 371, 375, 445, 550, 576, 585, 611, 617, 631, 632, 638, 653, 676, 682, 737, 742, 752, 758, 842, 877, 896, 898 |
| Rv2242 | *-* | hypothetical protein Rv2242 | 87, 133, 149, 178, 181, 190, 197, 209, 230, 252, 284, 295, 301, 304, 342, 357, 385 |
| Rv2243 | *fabD* | acyl-carrier-protein S-malonyltransferase | 6, 23, 35, 46, 55, 64, 80, 87, 100, 104, 112, 115, 128, 135, 157, 158, 164, 168, 189, 199, 207, 213, 217, 231, 233, 253, 269, 274, 292 |
| Rv2244 | *acpP* | acyl carrier protein | 9, 19, 64 |
| Rv2245 | *kasA* | 3-oxoacyl-(acyl carrier protein) synthase II | 5, 8, 16, 37, 40, 60, 90, 96, 97, 113, 131, 149, 161, 194, 241, 267, 279, 286, 297, 319, 328, 333, 385, 404 |
| Rv2246 | *kasB* | 3-oxoacyl-(acyl carrier protein) synthase II | 26, 36, 42, 60, 80, 117, 133, 151, 181, 192, 211, 214, 227, 243, 244, 262, 307, 322, 333, 351, 426 |
| Rv2247 | *accD6* | ACETYL/PROPIONYL-CoA CARBOXYLASE (BETA SUBUNIT) ACCD6 | 39, 64, 121, 134, 135, 136, 138, 159, 168, 187, 224, 327, 364, 406, 421, 428, 429, 464 |
| Rv2248 | *-* | hypothetical protein Rv2248 | 12, 50, 52, 70, 99, 101, 123, 252, 269 |
| Rv2249c | *glpD1* | PROBABLE GLYCEROL-3-PHOSPHATE DEHYDROGENASE GLPD1 | 14, 18, 32, 36, 47, 73, 80, 93, 128, 132, 194, 212, 255, 271, 278, 305, 328, 352, 353, 361, 382, 407, 428, 432, 451 |
| Rv2250c | *-* | Possible transcriptional regulatory protein | 10, 128, 142, 162, 172 |
| Rv2250A | *-* | POSSIBLE FLAVOPROTEIN | 8, 38, 46 |
| Rv2251 | *-* | POSSIBLE FLAVOPROTEIN | 11, 17, 31, 76, 112, 119, 122, 238, 249, 251, 264, 280, 281, 311, 325, 329, 339, 349, 361, 372, 390, 400, 412, 424, 464 |
| Rv2252 | *-* | diacylglycerol kinase | 2, 14, 25, 49, 60, 73, 74, 98, 118, 151, 215, 275, 294, 298 |
| Rv2253 | *-* | Possible secreted unknown protein | 1, 27, 46, 132, 139, 148 |
| Rv2254c | *-* | Probable integral membrane protein | 77, 91, 122, 132, 138 |
| Rv2255c | *-* | hypothetical protein Rv2255c | 33, 35 |
| Rv2256c | *-* | hypothetical protein Rv2256c | 18, 27, 72, 113, 141, 162, 165 |
| Rv2257c | *-* | hypothetical protein Rv2257c | 1, 15, 21, 31, 67, 92, 141, 143, 144, 182, 189, 214, 230, 260 |
| Rv2258c | *-* | Possible transcriptional regulatory protein | 1, 21, 33, 40, 66, 70, 90, 97, 126, 145, 151, 170, 179, 198, 266, 311, 329 |
| Rv2259 | *adhE2* | Probable zinc-dependent alcohol dehydrogenase AdhE2 | 5, 26, 28, 36, 38, 50, 64, 76, 80, 92, 100, 153, 156, 161, 165, 169, 173, 174, 187, 193, 196, 198, 240, 271, 297, 350 |
| Rv2260 | *-* | hypothetical protein Rv2260 | 1, 16, 39, 51, 86, 113, 139, 146, 153, 158, 173, 187 |
| Rv2261c | *-* | hypothetical protein Rv2261c | 19, 46, 49, 56, 120 |
| Rv2262c | *-* | hypothetical protein Rv2262c | 21, 24, 29, 50, 66, 88, 89, 98, 117, 139, 176, 188, 216, 227, 239, 244, 339 |
| Rv2263 | *-* | short chain dehydrogenase | 12, 19, 23, 59, 80, 100, 130, 155, 196, 204, 253, 258, 272, 278, 279, 288, 290, 308 |
| Rv2264c | *-* | conserved hypothetical proline rich protein | 95, 97, 98, 103, 111, 149, 176, 178, 185, 193, 202, 227, 262, 294, 321, 341, 350, 359, 379, 433, 444, 452, 470, 488, 497, 500, 533, 535, 574, 580 |
| Rv2265 | *-* | Possible conserved integral membrane protein | 45, 55, 63, 68, 93, 128, 130, 143, 147, 168, 174, 180, 188, 189, 191, 235, 238, 262, 272, 337, 357, 364, 365 |
| Rv2266 | *cyp124* | Probable cytochrome P450 124 CYP124 | 60, 223, 266, 302, 328, 333, 334, 372, 408 |
| Rv2267c | *-* | hypothetical protein Rv2267c | 90, 106, 109, 386 |
| Rv2268c | *cyp128* | PROBABLE CYTOCHROME P450 128 CYP128 | 1, 9, 17, 30, 69, 80, 88, 106, 109, 121, 208, 215, 223, 243, 263, 273, 429, 473 |
| Rv2269c | *-* | hypothetical protein Rv2269c | 47 |
| Rv2270 | *lppN* | PROBABLE LIPOPROTEIN LPPN | 27, 40, 45, 107, 119, 147, 162, 169 |
| Rv2271 | *-* | hypothetical protein Rv2271 | 25, 54 |
| Rv2272 | *-* | PROBABLE CONSERVED TRANSMEMBRANE PROTEIN | 7, 31, 37, 52, 70, 120 |
| Rv2273 | *-* | PROBABLE CONSERVED TRANSMEMBRANE PROTEIN | 10, 52, 85, 102 |
| Rv2274c | *-* | hypothetical protein Rv2274c | 26, 39, 75, 80, 93 |
| Rv2275 | *-* | hypothetical protein Rv2275 | 36, 58, 131, 144, 160, 286 |
| Rv2276 | *cyp121* | CYTOCHROME P450 121 CYP121 | 1, 37, 64, 67, 106, 115, 175, 189, 199, 212, 232, 237, 243, 267, 288, 339, 366 |
| Rv2277c | *-* | Possible glycerolphosphodiesterase | 16, 88, 167, 221, 239, 242, 261 |
| Rv2278 | *-* | PROBABLE TRANSPOSASE | 24, 60, 64, 71, 93 |
| Rv2279 | *-* | PROBABLE TRANSPOSASE | 68, 105, 109, 125, 259, 273, 289, 309, 310 |
| Rv2280 | *-* | Probable dehydrogenase | 18, 42, 57, 70, 72, 77, 100, 109, 118, 120, 123, 139, 146, 147, 170, 176, 177, 185, 240, 357, 359, 368, 372, 375, 421 |
| Rv2281 | *pitB* | Putative phosphate-transport permease PitB | 15, 62, 84, 116, 121, 122, 131, 137, 157, 179, 192, 195, 197, 221, 277, 329, 331, 439, 483, 484 |
| Rv2282c | *-* | Probable transcription regulator (lysR family) | 7, 21, 25, 27, 66, 86, 121, 126, 136, 217, 249, 276 |
| Rv2283 | *-* | hypothetical protein Rv2283 | 11, 23, 25, 44, 59 |
| Rv2284 | *lipM* | Probable esterase LipM | 21, 23, 62, 105, 118, 121, 128, 129, 132, 136, 176, 186, 187, 192, 196, 248, 257, 261, 262, 269, 342, 425 |
| Rv2285 | *-* | hypothetical protein Rv2285 | 61, 62, 83, 90, 122, 177, 189, 195, 260, 279, 295, 323, 353, 431, 440 |
| Rv2286c | *-* | hypothetical protein Rv2286c | 92, 98, 137, 139, 166, 216 |
| Rv2287 | *yjcE* | Probable conserved integral membrane transport protein YjcE | 23, 46, 105, 122, 135, 139, 154, 163, 184, 196, 200, 205, 242, 301, 361, 364, 381, 439, 479, 537 |
| Rv2288 | *-* | hypothetical protein Rv2288 | 73, 81 |
| Rv2289 | *cdh* | CDP-diacylglycerol pyrophosphatase | 21, 58, 94, 99, 115, 121, 210 |
| Rv2290 | *lppO* | Probable conserved lipoprotein lppO | 14, 29, 32, 44, 46, 59, 145, 156 |
| Rv2291 | *sseB* | Probable thiosulfate sulfurtransferase SseB | 9, 19, 64, 84, 101, 103, 123, 126, 130, 132, 137, 156, 167, 184, 194, 198, 205, 240 |
| Rv2292c | *-* | hypothetical protein Rv2292c | 2, 13, 29, 35, 59, 71 |
| Rv2293c | *-* | hypothetical protein Rv2293c | 12, 25, 55, 76, 85, 98, 112, 120, 123, 150, 196, 216 |
| Rv2294 | *-* | Probable aminotransferase | 58, 108, 160, 163, 182, 187, 212, 223, 241, 250, 278, 293, 311, 352, 375, 377 |
| Rv2295 | *-* | hypothetical protein Rv2295 | 19, 48, 70, 125, 139, 142, 169, 174, 192, 201 |
| Rv2296 | *-* | haloalkane dehalogenase | 27, 69, 71, 150, 174, 188, 189, 193, 206, 264, 284 |
| Rv2297 | *-* | hypothetical protein Rv2297 | 77, 111, 141 |
| Rv2298 | *-* | hypothetical protein Rv2298 | 34, 36, 54, 137, 143, 158, 214, 250, 268, 282, 298, 307 |
| Rv2299c | *htpG* | heat shock protein 90 | 102, 116, 157, 231, 448, 562, 571, 592, 612, 625 |
| Rv2300c | *-* | hypothetical protein Rv2300c | 21, 31, 57, 85, 95, 114, 143, 156, 161, 177, 197, 210, 218, 222, 225, 231, 239, 246 |
| Rv2301 | *cut2* | PROBABLE CUTINASE CUT2 | 14, 19, 30, 31, 34, 53, 59, 88, 118, 119, 147, 154, 174, 208, 225 |
| Rv2302 | *-* | hypothetical protein Rv2302 | 29, 51, 59, 71, 74 |
| Rv2303c | *-* | PROBABLE ANTIBIOTIC-RESISTANCE PROTEIN | 1, 19, 72, 99, 113, 119, 137, 149, 153, 174, 208, 231, 264, 265 |
| Rv2304c | *-* | hypothetical protein Rv2304c | 23, 35, 38, 52 |
| Rv2305 | *-* | hypothetical protein Rv2305 | 7, 31, 40, 45, 63, 133, 167, 180, 199, 221, 238, 255, 270, 300, 304, 307, 313, 315, 316, 363, 369, 404, 411, 419 |
| Rv2306A | *-* | POSSIBLE CONSERVED MEMBRANE PROTEIN | 21, 63, 76, 77, 79, 98, 102 |
| Rv2306B | *-* | POSSIBLE CONSERVED MEMBRANE PROTEIN | 10, 47, 71, 78, 94, 101, 119 |
| Rv2307c | *-* | hypothetical protein Rv2307c | 49, 50, 74, 78, 87, 113, 116, 128, 133, 139, 146, 221, 228, 249 |
| Rv2307A | *-* | HYPOTHETICAL GLYCINE RICH PROTEIN |  |
| Rv2307B | *-* | HYPOTHETICAL GLYCINE RICH PROTEIN | 60, 80, 87, 133, 135, 138 |
| Rv2307D | *-* | hypothetical protein Rv2307D | 17 |
| Rv2308 | *-* | hypothetical protein Rv2308 | 27, 28, 30, 41, 118, 150, 180, 201, 209 |
| Rv2309c | *-* | POSSIBLE INTEGRASE (FRAGMENT) | 1, 3, 43 |
| Rv2309A | *-* | hypothetical protein Rv2309A | 86 |
| Rv2310 | *-* | POSSIBLE EXCISIONASE | 6, 24, 41, 45, 103 |
| Rv2311 | *-* | hypothetical protein Rv2311 | 15, 52, 62, 65, 117, 134, 152 |
| Rv2312 | *-* | hypothetical protein Rv2312 | 12, 15, 24, 45, 64, 70 |
| Rv2313c | *-* | hypothetical protein Rv2313c | 72, 105, 121, 133, 178, 183, 207 |
| Rv2314c | *-* | hypothetical protein Rv2314c | 72, 81, 93, 97, 100, 107, 120, 126, 129, 137, 169, 178, 219, 243, 245, 252, 258, 315, 328, 338, 345, 346, 360, 381, 437 |
| Rv2315c | *-* | hypothetical protein Rv2315c | 21, 26, 29, 55, 88, 90, 134, 146, 197, 212, 220, 286, 309, 313, 329, 390, 421, 457, 465, 470, 475, 479 |
| Rv2316 | *uspA* | PROBABLE SUGAR-TRANSPORT INTEGRAL MEMBRANE PROTEIN ABC TRANSPORTER USPA | 62, 71, 89, 100, 172, 184, 214, 219, 231, 252 |
| Rv2317 | *uspB* | PROBABLE SUGAR-TRANSPORT INTEGRAL MEMBRANE PROTEIN ABC TRANSPORTER USPB | 36, 63, 72, 90, 169, 194, 216 |
| Rv2318 | *uspC* | PROBABLE PERIPLASMIC SUGAR-BINDING LIPOPROTEIN USPC | 11, 30, 35, 89, 104, 119, 133, 145, 158, 159, 173, 194, 212, 227, 228, 242, 288, 310, 311, 313, 361, 396, 397, 399, 402, 403 |
| Rv2319c | *-* | hypothetical protein Rv2319c | 14, 61, 63, 113, 124, 155, 158, 170, 237 |
| Rv2320c | *rocE* | PROBABLE CATIONIC AMINO ACID TRANSPORT INTEGRAL MEMBRANE PROTEIN ROCE | 18, 24, 50, 74, 77, 90, 95, 126, 162, 168, 227, 231, 248, 249, 272, 281, 296, 297, 317, 318, 377, 395, 421, 434, 450 |
| Rv2321c | *rocD2* | PROBABLE ORNITHINE AMINOTRANSFERASE (C-terminus part) ROCD2 (ORNITHINE--OXO-ACID AMINOTRANSFERASE) | 36, 56, 64, 69, 89, 112, 139, 149, 160 |
| Rv2322c | *rocD1* | PROBABLE ORNITHINE AMINOTRANSFERASE (N-terminus part) ROCD1 (ORNITHINE--OXO-ACID AMINOTRANSFERASE) | 62, 65, 92, 98, 115, 131, 162, 199 |
| Rv2323c | *-* | hypothetical protein Rv2323c | 64, 97, 105, 181, 203, 273, 288 |
| Rv2324 | *-* | PROBABLE TRANSCRIPTIONAL REGULATORY PROTEIN (PROBABLY ASNC-FAMILY) | 32, 46, 84, 118 |
| Rv2325c | *-* | hypothetical protein Rv2325c | 35, 67, 76, 96, 100, 109, 116, 125, 191, 220, 238, 241, 243, 268 |
| Rv2326c | *-* | POSSIBLE TRANSMEMBRANE ATP-BINDING PROTEIN ABC TRANSORTER | 5, 22, 27, 48, 59, 69, 88, 99, 114, 134, 147, 161, 168, 180, 225, 262, 276, 298, 319, 321, 350, 370, 374, 424, 438, 457, 487, 494, 513, 527, 534, 573, 596, 607, 617, 642, 655 |
| Rv2327 | *-* | hypothetical protein Rv2327 | 4, 6, 13, 43, 65, 91, 113, 152 |
| Rv2328 | *PE23* | PE FAMILY PROTEIN | 12, 30, 40, 48, 69, 127, 134, 138, 141, 162, 164, 175, 176, 180, 186, 191, 198, 211, 227, 232, 233, 239, 264, 267, 275, 290, 321, 328, 329, 343, 356, 357, 374 |
| Rv2329c | *narK1* | PROBABLE NITRITE EXTRUSION PROTEIN 1 NARK1 (NITRITE FACILITATOR 1) | 15, 114, 154, 157, 184, 251, 291, 343, 380, 425, 431, 446, 461, 463, 508 |
| Rv2330c | *lppP* | PROBABLE LIPOPROTEIN LPPP | 28, 52, 150 |
| Rv2331 | *-* | hypothetical protein Rv2331 | 111, 119, 124 |
| Rv2331A | *-* | hypothetical protein Rv2331A | 34 |
| Rv2332 | *mez* | PROBABLE | 10, 13, 34, 111, 114, 173, 174, 175, 212, 267, 273, 277, 282, 298, 418, 433, 459, 494, 519 |
| Rv2333c | *-* | PROBABLE CONSERVED INTEGRAL MEMBRANE TRANSPORT PROTEIN | 88, 99, 137, 151, 196, 290, 297, 360, 362, 377, 380, 388, 411, 450, 492, 503 |
| Rv2334 | *cysK1* | PROBABLE CYSTEINE SYNTHASE A CYSK1 (O-ACETYLSERINE SULFHYDRYLASE A) (O-ACETYLSERINE (THIOL)-LYASE A) (CSASE A) | 71, 74, 85, 157, 178, 183, 204, 224, 268, 283 |
| Rv2335 | *cysE* | PROBABLE SERINE ACETYLTRANSFERASE CYSE (SAT) | 20, 63, 69, 83, 90, 106, 112, 127, 163, 174, 175, 204, 218 |
| Rv2336 | *-* | hypothetical protein Rv2336 | 40, 82, 83, 110, 125, 130, 144, 157, 180, 213, 277, 287 |
| Rv2337c | *-* | hypothetical protein Rv2337c | 2, 29, 41, 55, 94, 149, 200, 209, 274, 298, 326 |
| Rv2338c | *moeW* | hypothetical protein Rv2338c | 2, 209, 239, 279 |
| Rv2339 | *mmpL9* | PROBABLE CONSERVED TRANSMEMBRANE TRANSPORT PROTEIN MMPL9 | 25, 72, 148, 181, 192, 193, 202, 267, 300, 312, 343, 349, 355, 377, 383, 406, 422, 426, 453, 469, 675, 678, 775, 868, 870, 877 |
| Rv2340c | *PE_PGRS39* | PE-PGRS FAMILY PROTEIN | 4, 14, 18, 29, 32, 41, 42, 91, 117, 121, 123, 127, 133, 139, 153, 163, 168, 172, 180, 185, 186, 202, 208, 217, 228, 231, 237, 240, 244, 253, 257, 266, 272, 277, 281, 309, 326, 347, 372, 384, 391, 409 |
| Rv2341 | *lppQ* | PROBABLE CONSERVED LIPOPROTEIN LPPQ | 20, 31, 35, 79, 85, 89 |
| Rv2342 | *-* | hypothetical protein Rv2342 | 17, 33, 44, 69, 74, 82 |
| Rv2343c | *dnaG* | DNA primase | 1, 31, 64, 67, 100, 110, 118, 122, 152, 184, 211, 278, 282, 287, 289, 292, 310, 322, 325, 359, 381, 396, 422, 465, 466, 500, 528, 535, 576, 632, 637 |
| Rv2344c | *dgt* | deoxyguanosinetriphosphate triphosphohydrolase-like protein | 24, 48, 99, 126, 240, 246, 267, 278, 307, 314, 316, 386, 391 |
| Rv2345 | *-* | POSSIBLE CONSERVED TRANSMEMBRANE PROTEIN | 14, 15, 22, 45, 48, 74, 103, 113, 141, 208, 249, 360, 365, 389, 393, 401, 419, 456, 459, 477, 479, 484, 509, 523, 539, 559, 582, 595, 608, 622, 627, 638, 650, 654, 656 |
| Rv2346c | *esxO* | PUTATIVE ESAT-6 LIKE PROTEIN ESXO (ESAT-6 LIKE PROTEIN 6) | 20, 36, 42, 44, 77 |
| Rv2347c | *esxP* | PUTATIVE ESAT-6 LIKE PROTEIN ESXP (ESAT-6 LIKE PROTEIN 7) | 35 |
| Rv2348c | *-* | hypothetical protein Rv2348c | 24, 106 |
| Rv2349c | *plcC* | PROBABLE PHOSPHOLIPASE C 3 PLCC | 12, 22, 32, 90, 101, 120, 127, 180, 208, 228, 241, 260, 333, 343, 353, 359, 375, 376, 398, 435, 496, 498, 504 |
| Rv2350c | *plcB* | PROBABLE MEMBRANE-ASSOCIATED PHOSPHOLIPASE C 2 PLCB | 9, 11, 14, 15, 36, 66, 91, 92, 109, 129, 183, 204, 231, 284, 300, 308, 336, 346, 362, 375, 400, 430, 447, 500, 508 |
| Rv2351c | *plcA* | PROBABLE MEMBRANE-ASSOCIATED PHOSPHOLIPASE C 1 PLCA (MTP40 ANTIGEN) | 32, 36, 70, 89, 90, 196, 197, 228, 305, 318, 327, 333, 343, 356, 359, 372, 374, 397, 427, 503, 507 |
| Rv2352c | *PPE38* | PPE FAMILY PROTEIN | 18, 51, 81, 92, 183, 188, 234, 262, 278, 290, 343, 364, 369 |
| Rv2353c | *PPE39* | PPE FAMILY PROTEIN | 14, 24, 34, 54, 57, 67, 69, 87, 95, 101, 109, 111, 119, 121, 129, 144, 149, 159, 161, 179, 204, 252, 254, 292, 315, 320, 340 |
| Rv2354 | *-* | PROBABLE TRANSPOSASE | 24, 60, 64, 71, 93 |
| Rv2355 | *-* | PROBABLE TRANSPOSASE | 68, 105, 109, 125, 259, 273, 289, 309, 310 |
| Rv2356c | *PPE40* | PPE FAMILY PROTEIN | 97, 137, 183, 191, 219, 223, 233, 239, 249, 252, 254, 257, 259, 269, 274, 279, 289, 308, 312, 318, 332, 334, 352, 360, 374, 404, 409, 424, 434, 449, 459, 469, 479, 489, 494, 499, 519, 532, 535, 550, 560, 580, 600 |
| Rv2357c | *glyS* | glycyl-tRNA synthetase | 29, 80, 129, 132, 165, 302, 402 |
| Rv2358 | *-* | PROBABLE TRANSCRIPTIONAL REGULATORY PROTEIN (PROBABLY ARSR-FAMILY) | 8, 20, 25, 36, 47, 55, 101, 129 |
| Rv2359 | *furB* | PROBABLE FERRIC UPTAKE REGULATION PROTEIN FURB | 3, 37, 56, 66, 87 |
| Rv2360c | *-* | hypothetical protein Rv2360c | 98, 102, 112, 114 |
| Rv2361c | *-* | LONG (C50) CHAIN Z-ISOPRENYL DIPHOSPHATE SYNTHASE (Z-DECAPRENYL DIPHOSPHATE SYNTHASE) | 57, 104, 195, 212 |
| Rv2362c | *recO* | DNA repair protein RecO | 91, 98, 103, 109, 146, 171, 182, 196, 205, 222, 254, 262 |
| Rv2363 | *amiA2* | amidase | 12, 64, 78, 102, 126, 148, 168, 175, 176, 180, 188, 194, 207, 241, 243, 280, 292, 328, 371, 416, 463 |
| Rv2364c | *era* | GTP-binding protein Era | 16, 61, 93, 112, 158, 170, 175, 177, 235, 261 |
| Rv2365c | *-* | hypothetical protein Rv2365c | 31, 33, 58, 73, 82, 91, 99 |
| Rv2366c | *-* | PROBABLE CONSERVED TRANSMEMBRANE PROTEIN | 1, 20, 24, 72, 74, 94, 152, 154, 218, 228, 262, 309, 374, 387 |
| Rv2367c | *-* | hypothetical protein Rv2367c | 48, 62, 101, 108 |
| Rv2368c | *phoH1* | PROBABLE PHOH-LIKE PROTEIN PHOH1 (PHOSPHATE STARVATION-INDUCIBLE PROTEIN PSIH) | 56, 63, 85, 151, 153, 180, 184, 275, 285, 333, 345, 348 |
| Rv2369c | *-* | hypothetical protein Rv2369c | 15, 36, 64 |
| Rv2370c | *-* | hypothetical protein Rv2370c | 8, 37, 43, 46, 65, 71, 93, 148, 151, 200, 219, 274, 358, 375, 401 |
| Rv2371 | *PE_PGRS40* | PE-PGRS FAMILY PROTEIN | 29, 41, 42 |
| Rv2372c | *-* | hypothetical protein Rv2372c | 13, 27, 35, 56, 59, 100, 166, 167, 209, 214, 230, 232 |
| Rv2373c | *dnaJ2* | PROBABLE CHAPERONE PROTEIN DNAJ2 | 66, 73, 76, 84, 95, 140, 164, 167, 191, 222, 233, 240, 300, 311, 365, 367, 368, 379 |
| Rv2374c | *hrcA* | heat-inducible transcription repressor | 63, 99, 155, 205, 207, 208, 236, 238, 275, 303, 309 |
| Rv2375 | *-* | hypothetical protein Rv2375 | 100 |
| Rv2376c | *cfp2* | LOW MOLECULAR WEIGHT ANTIGEN CFP2 (LOW MOLECULAR WEIGHT PROTEIN ANTIGEN 2) (CFP-2) | 7, 11, 18, 21, 29, 51, 57, 84, 101, 120, 121, 125, 149 |
| Rv2377c | *mbtH* | PUTATIVE CONSERVED PROTEIN MBTH | 33, 45 |
| Rv2378c | *mbtG* | LYSINE-N-OXYGENASE MBTG (L-LYSINE 6-MONOOXYGENASE) (LYSINE N6-HYDROXYLASE) | 48, 98, 143, 164, 166, 175, 190, 191, 206, 210, 237, 358, 385, 391, 409, 423 |
| Rv2379c | *mbtF* | PEPTIDE SYNTHETASE MBTF (PEPTIDE SYNTHASE) | 35, 38, 42, 57, 70, 129, 179, 196, 246, 252, 276, 288, 295, 321, 349, 366, 372, 398, 403, 458, 460, 470, 535, 555, 578, 606, 609, 724, 730, 763, 797, 809, 829, 836, 861, 871, 888, 902, 985, 1004, 1034, 1043, 1087, 1122, 1127, 1144, 1159, 1165, 1185, 1192, 1263, 1279, 1286, 1292, 1346, 1348, 1362, 1422, 1440 |
| Rv2380c | *mbtE* | PEPTIDE SYNTHETASE MBTE (PEPTIDE SYNTHASE) | 9, 22, 66, 161, 163, 189, 192, 202, 210, 213, 224, 234, 260, 290, 297, 337, 385, 404, 421, 429, 455, 511, 548, 576, 579, 641, 651, 690, 695, 760, 781, 784, 795, 802, 827, 869, 872, 876, 900, 912, 920, 938, 967, 1000, 1003, 1054, 1088, 1098, 1106, 1107, 1111, 1155, 1170, 1177, 1226, 1239, 1241, 1251, 1274, 1283, 1284, 1296, 1321, 1385, 1402, 1414, 1419, 1433, 1478, 1505, 1507, 1519, 1529, 1542, 1572, 1604, 1620, 1625, 1639, 1674 |
| Rv2381c | *mbtD* | POLYKETIDE SYNTHETASE MBTD (POLYKETIDE SYNTHASE) | 63, 77, 78, 101, 123, 129, 134, 148, 179, 245, 246, 263, 279, 284, 322, 325, 371, 384, 391, 428, 439, 462, 538, 539, 548, 583, 586, 589, 633, 636, 662, 671, 700, 756, 763, 820, 843, 888, 933, 977, 994 |
| Rv2382c | *mbtC* | POLYKETIDE SYNTHETASE MBTC (POLYKETIDE SYNTHASE) | 16, 21, 66, 108, 116, 133, 147, 155, 165, 184, 191, 199, 200, 208, 234, 236, 242, 254, 279, 282, 301, 312, 331, 334, 336, 355, 365, 369, 408, 412, 417, 432 |
| Rv2383c | *mbtB* | PHENYLOXAZOLINE SYNTHASE MBTB (PHENYLOXAZOLINE SYNTHETASE) | 27, 61, 74, 75, 77, 85, 90, 119, 232, 297, 303, 304, 334, 343, 393, 412, 419, 462, 483, 498, 516, 517, 528, 531, 545, 574, 623, 624, 625, 646, 675, 683, 689, 701, 713, 729, 750, 754, 755, 798, 811, 841, 880, 889, 895, 897, 910, 925, 950, 961, 967, 979, 985, 993, 996, 1009, 1028, 1041, 1090, 1101, 1119, 1124, 1131, 1168, 1181, 1186, 1195, 1196, 1199, 1288, 1296, 1298, 1303, 1316, 1344, 1362, 1382 |
| Rv2384 | *mbtA* | BIFUNCTIONAL ENZYME MBTA: SALICYL-AMP LIGASE (SAL-AMP LIGASE) + SALICYL-S-ArCP SYNTHETASE | 14, 23, 27, 33, 48, 61, 82, 126, 130, 143, 152, 192, 194, 203, 212, 213, 237, 253, 254, 274, 285, 288, 298, 328, 342, 397, 406, 433, 448, 463, 480, 520 |
| Rv2385 | *mbtJ* | PUTATIVE ACETYL HYDROLASE MBTJ | 6, 25, 68, 70, 72, 81, 102, 121, 137, 140, 147, 150, 166, 213, 217, 227, 228, 259, 260 |
| Rv2386c | *mbtI* | salicylate synthase MbtI | 12, 20, 21, 25, 56, 57, 166, 184, 199, 241, 268, 277, 328, 350, 362, 385, 395, 399, 410, 417, 419 |
| Rv2387 | *-* | hypothetical protein Rv2387 | 55, 96, 107, 146, 173, 185, 188, 191, 203, 255, 379, 411 |
| Rv2388c | *hemN* | coproporphyrinogen III oxidase | 1, 18, 51, 66, 116, 147, 182, 186, 192, 214, 235, 249, 294, 358 |
| Rv2389c | *rpfD* | PROBABLE RESUSCITATION-PROMOTING FACTOR RPFD | 28, 61, 62, 69, 95 |
| Rv2390c | *-* | hypothetical protein Rv2390c | 13, 15, 22, 24, 45, 50, 63, 64, 70, 98 |
| Rv2391 | *nirA* | PROBABLE FERREDOXIN-DEPENDENT NITRITE REDUCTASE NIRA | 1, 13, 39, 88, 109, 115, 131, 168, 181, 217, 251, 357, 375, 403, 425, 494, 505, 512 |
| Rv2392 | *cysH* | phosphoadenosine phosphosulfate reductase | 1, 16, 18, 27, 43, 45, 46, 50, 53, 75, 89, 156, 160, 229, 237, 248 |
| Rv2393 | *-* | hypothetical protein Rv2393 | 1, 14, 40, 50, 60, 75, 176, 200, 242, 248, 263, 275 |
| Rv2394 | *ggtB* | PROBABLE GAMMA-GLUTAMYLTRANSPEPTIDASE PRECURSOR GGTB (GAMMA-GLUTAMYLTRANSFERASE) (GLUTAMYL TRANSPEPTIDASE) | 18, 21, 31, 65, 76, 83, 101, 108, 121, 124, 126, 151, 206, 213, 251, 259, 266, 270, 271, 278, 322, 327, 338, 361, 396, 397, 413, 525, 536, 569, 578, 626, 627 |
| Rv2395 | *-* | PROBABLE CONSERVED INTEGRAL MEMBRANE PROTEIN | 1, 13, 26, 46, 75, 95, 109, 126, 137, 147, 192, 237, 270, 288, 342, 346, 347, 363, 367, 388, 400, 414, 434, 485, 493, 515, 548, 655 |
| Rv2396 | *PE_PGRS41* | PE-PGRS FAMILY PROTEIN | 32, 48, 80, 81, 83, 88, 91, 93, 131, 134, 135, 137, 138, 145, 147, 148, 154, 156, 158, 162, 165, 167, 168, 174, 176, 177, 180, 190, 195, 196, 200, 202, 204, 205, 207, 214, 218, 220, 227, 231, 233, 234, 236, 237, 240, 255, 256, 259, 265, 266, 269, 272, 274, 282, 284, 285, 288, 294, 297, 300, 306, 307, 309, 313, 316, 319, 325, 328, 336, 337, 341, 343, 347 |
| Rv2397c | *cysA1* | PROBABLE SULFATE-TRANSPORT ATP-BINDING PROTEIN ABC TRANSPORTER CYSA1 | 28, 32, 38, 50, 57, 124, 135, 261, 266, 267, 270, 296, 297, 301, 306, 331, 340 |
| Rv2398c | *cysW* | PROBABLE SULFATE-TRANSPORT INTEGRAL MEMBRANE PROTEIN ABC TRANSPORTER CYSW | 85, 116, 173 |
| Rv2399c | *cysT* | PROBABLE SULFATE-TRANSPORT INTEGRAL MEMBRANE PROTEIN ABC TRANSPORTER CYST | 18, 20, 44, 50, 52, 66, 79, 143, 145, 203, 223, 247, 250, 281 |
| Rv2400c | *subI* | PROBABLE SULFATE-BINDING LIPOPROTEIN SUBI | 23, 31, 39, 67, 74, 99, 124, 190, 218, 232, 281, 299, 309, 343, 354 |
| Rv2401 | *-* | hypothetical protein Rv2401 | 9, 43 |
| Rv2401A | *-* | POSSIBLE CONSERVED MEMBRANE PROTEIN | 18, 40 |
| Rv2402 | *-* | hypothetical protein Rv2402 | 58, 91, 136, 227, 271, 329, 346, 360, 430, 485, 547, 636 |
| Rv2403c | *lppR* | PROBABLE CONSERVED LIPOPROTEIN LPPR | 17, 24, 86, 135, 138, 161, 188 |
| Rv2404c | *lepA* | GTP-binding protein LepA | 75, 97, 150, 182, 190, 208, 211, 250, 274, 306, 327, 330, 335, 389, 474, 478, 512, 522, 527, 579, 604, 645, 646 |
| Rv2405 | *-* | hypothetical protein Rv2405 | 36, 42, 52, 64, 83, 99, 178 |
| Rv2406c | *-* | hypothetical protein Rv2406c |  |
| Rv2407 | *-* | ribonuclease Z | 26, 35, 37, 50, 78, 89, 126, 166, 179, 180, 216, 219, 239, 249, 254 |
| Rv2408 | *PE24* | POSSIBLE PE FAMILY-RELATED PROTEIN | 11, 20, 36, 46, 120, 140, 214 |
| Rv2409c | *-* | hypothetical protein Rv2409c | 9, 89, 141, 162, 223 |
| Rv2410c | *-* | hypothetical protein Rv2410c | 21, 141, 204, 270 |
| Rv2411c | *-* | hypothetical protein Rv2411c | 19, 25, 48, 169, 178, 320, 328, 397, 469, 488, 504, 529 |
| Rv2412 | *rpsT* | 30S ribosomal protein S20 | 29, 46, 71 |
| Rv2413c | *-* | hypothetical protein Rv2413c | 65, 74, 80, 86, 97, 147, 171, 177, 185, 201, 217, 242, 249, 276, 301, 313 |
| Rv2414c | *-* | hypothetical protein Rv2414c | 24, 44, 67, 102, 136, 179, 187, 193, 210, 229, 328, 362, 377, 387, 390, 427, 436, 454, 461, 465, 507 |
| Rv2415c | *-* | hypothetical protein Rv2415c | 5, 23, 77, 86, 133, 165, 166, 167, 250, 256 |
| Rv2416c | *eis* | hypothetical protein Rv2416c | 19, 33, 51, 78, 79, 93, 110, 122, 132, 154, 171, 187, 205, 207, 244, 302, 325, 369, 397 |
| Rv2417c | *-* | hypothetical protein Rv2417c | 54, 63, 74, 97, 103, 124, 128, 163, 175, 218, 252, 264 |
| Rv2418c | *-* | hypothetical protein Rv2418c | 4, 23, 24, 76, 83, 84, 137, 200, 237, 245 |
| Rv2419c | *-* | PROBABLE PHOSPHOGLYCERATE MUTASE (PHOSPHOGLYCEROMUTASE) | 40, 71, 118, 163, 181 |
| Rv2420c | *-* | hypothetical protein Rv2420c | 1, 13, 14, 17, 31 |
| Rv2421c | *nadD* | nicotinic acid mononucleotide adenyltransferase | 14, 32, 43, 56, 71, 97, 126, 205 |
| Rv2422 | *-* | hypothetical protein Rv2422 | 40, 85, 86 |
| Rv2423 | *-* | hypothetical protein Rv2423 | 49, 154, 156, 166, 243, 263, 273, 286 |
| Rv2424c | *-* | PROBABLE TRANSPOSASE | 27, 37, 109, 216, 241, 266, 290, 331 |
| Rv2425c | *-* | hypothetical protein Rv2425c | 1, 17, 30, 42, 84, 102, 128, 152, 198, 267, 290, 291, 428, 446, 465 |
| Rv2426c | *-* | hypothetical protein Rv2426c | 3, 23, 31, 50, 51, 68, 99, 124, 168, 233, 267, 284 |
| Rv2427c | *proA* | gamma-glutamyl phosphate reductase | 17, 24, 72, 89, 90, 155, 160, 173, 203, 212, 232, 267, 287, 292, 334, 358, 390, 408 |
| Rv2428 | *ahpC* | ALKYL HYDROPEROXIDE REDUCTASE C PROTEIN AHPC (ALKYL HYDROPEROXIDASE C) | 10, 15, 19, 30, 120, 149, 150 |
| Rv2429 | *ahpD* | ALKYL HYDROPEROXIDE REDUCTASE D PROTEIN AHPD (ALKYL HYDROPEROXIDASE D) | 7, 42, 73, 87, 99, 124, 156 |
| Rv2430c | *PPE41* | PPE FAMILY PROTEIN | 11, 16, 18, 26 |
| Rv2431c | *PE25* | PE FAMILY PROTEIN | 36, 88 |
| Rv2432c | *-* | hypothetical protein Rv2432c | 25, 39, 43, 53, 87 |
| Rv2433c | *-* | hypothetical protein Rv2433c | 21, 27, 52, 81, 87 |
| Rv2434c | *-* | PROBABLE CONSERVED TRANSMEMBRANE PROTEIN | 20, 110, 143, 187, 202, 204, 217, 226, 262, 266, 302, 325, 375, 412, 413, 422, 471 |
| Rv2435c | *-* | PROBABLE CYCLASE (ADENYLYL-OR GUANYLYL-)(ADENYLATE-OR GUANYLATE-) | 69, 114, 126, 148, 155, 187, 195, 241, 281, 291, 312, 331, 387, 390, 393, 502, 570, 591, 609, 611, 642, 654, 659, 684, 689 |
| Rv2436 | *rbsK* | RIBOKINASE RBSK | 35, 49, 69, 77, 99, 102, 150, 154, 172, 183, 213, 227, 236, 237, 239, 279, 287 |
| Rv2437 | *-* | hypothetical protein Rv2437 | 21, 59, 91, 121, 124 |
| Rv2438c | *nadE* | NAD synthetase | 4, 14, 29, 83, 105, 111, 137, 161, 166, 200, 230, 367, 399, 403, 430, 458, 472, 479, 505, 548, 601 |
| Rv2438A | *-* | hypothetical protein Rv2438A | 10, 25, 35, 67, 73, 85 |
| Rv2439c | *proB* | gamma-glutamyl kinase | 10, 12, 27, 36, 61, 62, 111, 189, 199, 209, 218, 230, 233, 241, 245, 247, 262, 275, 279, 286, 305, 308, 312, 329, 351 |
| Rv2440c | *obgE* | GTPase ObgE | 16, 32, 36, 42, 66, 81, 82, 116, 118, 122, 127, 137, 169, 247, 282, 312, 321, 363, 378, 446, 467 |
| Rv2441c | *rpmA* | 50S ribosomal protein L27 | 15, 26, 32, 40, 46, 53, 63, 72 |
| Rv2442c | *rplU* | 50S ribosomal protein L21 | 9, 10, 61, 100 |
| Rv2443 | *dctA* | PROBABLE C4-DICARBOXYLATE-TRANSPORT TRANSMEMBRANE PROTEIN DCTA | 1, 35, 46, 83, 90, 130, 135, 184, 239, 312, 321, 360, 363, 371, 372, 405, 431, 450, 457, 474, 481 |
| Rv2444c | *rne* | POSSIBLE RIBONUCLEASE E RNE | 43, 75, 161, 166, 168, 170, 197, 213, 226, 273, 279, 308, 398, 406, 417, 429, 456, 469, 501, 536, 568, 596, 604, 631, 649, 653, 655, 684, 738, 754, 766, 775, 805, 824, 829, 832, 840, 851, 903, 914, 926, 945 |
| Rv2445c | *ndk* | nucleoside diphosphate kinase | 88, 89, 99, 123, 133 |
| Rv2446c | *-* | PROBABLE CONSERVED INTEGRAL MEMBRANE PROTEIN | 40, 62, 83, 99 |
| Rv2447c | *folC* | PROBABLE FOLYLPOLYGLUTAMATE SYNTHASE PROTEIN FOLC (FOLYLPOLY-GAMMA-GLUTAMATE SYNTHETASE) (FPGS) | 5, 14, 86, 92, 155, 177, 209, 212, 272, 282, 314, 327, 330, 335, 351, 356, 420, 431, 449, 456, 463, 468, 473 |
| Rv2448c | *valS* | valyl-tRNA synthetase | 8, 25, 45, 82, 121, 125, 162, 180, 225, 267, 274, 304, 347, 370, 476, 499, 558, 562, 595, 668, 672, 673, 698, 712, 720, 766, 812, 823, 832 |
| Rv2449c | *-* | hypothetical protein Rv2449c | 1, 15, 22, 29, 55, 63, 76, 98, 102, 103, 111, 128, 131, 139, 162, 177, 180, 181, 193, 214, 239, 243, 292, 322, 324, 333, 346, 360, 392, 394, 406, 407 |
| Rv2450c | *rpfE* | PROBABLE RESUSCITATION-PROMOTING FACTOR RPFE | 9, 13, 22, 36, 38, 108, 116, 122, 127, 128, 139, 170 |
| Rv2451 | *-* | HYPOTHETICAL PROLINE AND SERINE RICH PROTEIN | 10, 16, 39, 54, 83, 94, 129 |
| Rv2452c | *-* | hypothetical protein Rv2452c | 20 |
| Rv2453c | *mobA* | molybdopterin-guanine dinucleotide biosynthesis protein A | 11, 33, 58, 85, 87, 95, 141, 158, 198 |
| Rv2454c | *-* | 2-oxoglutarate ferredoxin oxidoreductase subunit beta | 3, 11, 14, 58, 85, 110, 114, 129, 138, 222, 227, 280, 351, 354, 356 |
| Rv2455c | *-* | PROBABLE OXIDOREDUCTASE (ALPHA SUBUNIT) | 5, 7, 18, 36, 43, 65, 76, 92, 93, 118, 145, 223, 242, 247, 257, 266, 270, 309, 317, 358, 362, 400, 448, 472, 479, 522, 593, 622, 625 |
| Rv2456c | *-* | PROBABLE CONSERVED INTEGRAL MEMBRANE TRANSPORT PROTEIN | 55, 82, 88, 90, 110, 147, 156, 173, 203, 298, 300, 324, 360, 367, 387, 389 |
| Rv2457c | *clpX* | ATP-dependent protease ATP-binding subunit | 15, 27, 29, 79, 97, 122, 147, 152, 169, 179, 204, 253, 364, 366, 369 |
| Rv2458 | *mmuM* | homocysteine methyltransferase | 12, 21, 47, 72, 100, 113, 122, 146, 189, 200, 240, 258, 261, 277, 296 |
| Rv2459 | *-* | PROBABLE CONSERVED INTEGRAL MEMBRANE TRANSPORT PROTEIN | 48, 148, 151, 222, 223, 247, 306, 365, 366, 385, 388, 389, 399, 417, 470, 477, 484, 486, 491, 505 |
| Rv2460c | *clpP2* | ATP-dependent Clp protease proteolytic subunit | 79, 107, 116, 117, 153, 171, 175, 189, 209, 212 |
| Rv2461c | *clpP* | ATP-dependent Clp protease proteolytic subunit | 51, 72, 95, 126, 129, 157, 175 |
| Rv2462c | *tig* | trigger factor | 41, 45, 106, 152, 181, 191, 206, 214, 219, 223, 315, 344, 415, 442, 455 |
| Rv2463 | *lipP* | PROBABLE ESTERASE/LIPASE LIPP | 48, 50, 53, 54, 69, 85, 119, 125, 144, 154, 201, 226, 266, 303, 329, 345, 386 |
| Rv2464c | *-* | POSSIBLE DNA GLYCOSYLASE | 18, 36, 59, 87, 105, 161, 182, 184, 208, 238 |
| Rv2465c | *-* | ribose-5-phosphate isomerase B | 12, 27, 51, 68, 77, 82, 94, 110, 157 |
| Rv2466c | *-* | hypothetical protein Rv2466c | 76, 91, 116, 119, 125, 141, 172, 176 |
| Rv2467 | *pepN* | PROBABLE AMINOPEPTIDASE N PEPN (LYSYL AMINOPEPTIDASE) (LYS-AP) (ALANINE AMINOPEPTIDASE) | 14, 35, 52, 61, 84, 89, 108, 131, 155, 157, 170, 193, 263, 269, 366, 373, 426, 447, 472, 476, 499, 513, 527, 552, 572, 592, 593, 621, 678, 698, 702, 726, 727, 732, 768, 771, 773, 791, 821, 834 |
| Rv2468c | *-* | hypothetical protein Rv2468c | 34, 43, 86, 124, 126, 137 |
| Rv2469c | *-* | hypothetical protein Rv2469c | 7, 12, 19, 21, 67, 91, 127, 140, 156 |
| Rv2470 | *glbO* | POSSIBLE GLOBIN (OXYGEN-BINDING PROTEIN) GLBO | 9, 41, 56, 65, 118 |
| Rv2471 | *aglA* | PROBABLE ALPHA-GLUCOSIDASE AGLA (MALTASE) (GLUCOINVERTASE) (GLUCOSIDOSUCRASE) (MALTASE-GLUCOAMYLASE) (LYSOSOMAL ALPHA-GLUCOSIDASE) (ACID MALTASE) | 11, 101, 138, 174, 224, 283, 323, 335, 361, 417, 455, 463, 491, 503, 512, 540 |
| Rv2472 | *-* | hypothetical protein Rv2472 | 9, 30, 42, 46, 58, 61, 95 |
| Rv2473 | *-* | POSSIBLE ALANINE AND PROLINE RICH MEMBRANE PROTEIN | 19, 21, 29, 31, 78, 115, 133, 153, 157, 172, 182 |
| Rv2474c | *-* | hypothetical protein Rv2474c | 29, 39, 59, 70, 89, 95, 105, 168, 176, 208 |
| Rv2475c | *-* | hypothetical protein Rv2475c | 49, 111 |
| Rv2476c | *gdh* | PROBABLE NAD-DEPENDENT GLUTAMATE DEHYDROGENASE GDH (NAD-GDH) (NAD-DEPENDENT GLUTAMIC DEHYDROGENASE) | 4, 17, 47, 60, 75, 88, 102, 163, 209, 210, 214, 254, 263, 414, 436, 464, 485, 513, 566, 624, 633, 652, 719, 721, 724, 809, 845, 856, 859, 879, 892, 914, 944, 957, 990, 1008, 1023, 1052, 1083, 1106, 1161, 1162, 1173, 1174, 1195, 1254, 1275, 1351, 1352, 1415, 1481, 1502, 1523, 1563, 1595, 1606, 1617 |
| Rv2477c | *-* | putative ABC transporter ATP-binding protein | 41, 51, 65, 85, 160, 205, 274, 374, 439, 443, 444, 459, 488, 543, 556 |
| Rv2478c | *-* | hypothetical protein Rv2478c | 34, 38, 54, 112, 116, 121, 125, 128, 130 |
| Rv2479c | *-* | PROBABLE TRANSPOSASE | 102, 139, 143, 159, 293, 307, 323, 343, 344 |
| Rv2480c | *-* | POSSIBLE TRANSPOSASE | 24, 60, 64, 71, 93 |
| Rv2481c | *-* | hypothetical protein Rv2481c | 34, 63, 94, 96 |
| Rv2482c | *plsB2* | glycerol-3-phosphate acyltransferase | 71, 95, 136, 198, 203, 216, 225, 244, 262, 300, 360, 405, 462, 486, 492, 499, 501, 572, 606 |
| Rv2483c | *plsC* | POSSIBLE TRANSMEMBRANE PHOSPHOLIPID BIOSYNTHESIS BIFUNCTIONNAL ENZYME PLSC: PUTATIVE L-3-PHOSPHOSERINE PHOSPHATASE (O-PHOSPHOSERINE PHOSPHOHYDROLASE) (PSP) (PSPASE) + 1-ACYL-SN-GLYCEROL-3-PHOSPHATE ACYLTRANSFERASE (1-AGP ACYLTRANSFERASE) (1-AGPAT) (LYSOPHOSPHATIDIC ACID ACYLTRANSFERASE) (LPAAT) | 2, 22, 33, 49, 77, 95, 100, 137, 178, 238, 254, 267, 277, 286, 291, 308, 321, 325, 341, 382, 498, 508, 520, 530, 534, 540, 568 |
| Rv2484c | *-* | hypothetical protein Rv2484c | 3, 73, 96, 98, 151, 199, 206, 212, 269, 272, 273, 318, 319, 324, 355, 379, 402, 416, 428, 463, 480 |
| Rv2485c | *lipQ* | PROBABLE CARBOXYLESTERASE LIPQ | 12, 33, 53, 62, 110, 120, 129, 146, 147, 163, 175, 236, 245, 246, 248, 249, 254, 257, 279, 363, 381 |
| Rv2486 | *echA14* | enoyl-CoA hydratase | 55, 63, 72, 101, 110, 117, 123, 126, 142, 171, 187, 193, 194, 196, 198, 217, 246, 251 |
| Rv2487c | *PE_PGRS42* | PE-PGRS FAMILY PROTEIN | 30, 82, 91, 123, 129, 130, 143, 148, 149, 151, 155, 161, 164, 166, 169, 172, 173, 175, 186, 189, 192, 204, 207, 210, 216, 218, 221, 222, 224, 227, 236, 238, 242, 256, 257, 260, 263, 268, 272, 275, 276, 281, 290, 291, 293, 297, 299, 302, 304, 306, 309, 312, 324, 335, 336, 342, 345, 347, 359, 362, 373, 375, 385, 390, 391, 398, 403, 418, 421, 443, 449, 452, 454, 459, 461, 462, 464, 465, 468, 471, 473, 474, 481, 484, 487, 489, 490, 493, 495, 498, 502, 505, 510, 533, 534, 541, 544, 546, 556, 563, 568, 574, 578, 581, 584, 587, 589, 603, 605, 606, 609, 618, 624, 627, 628, 633, 638, 640, 644, 653, 656, 658, 662, 664, 677, 683, 689, 692 |
| Rv2488c | *-* | PROBABLE TRANSCRIPTIONAL REGULATORY PROTEIN (LUXR-FAMILY) | 33, 94, 140, 159, 264, 267, 376, 457, 482, 497, 508, 509, 510, 559, 577, 578, 586, 604, 615, 643, 675, 705, 707, 711, 751, 787, 812, 833, 839, 841, 848, 862, 870, 871, 876, 962, 985, 1056 |
| Rv2489c | *-* | HYPOTHETICAL ALANINE RICH PROTEIN | 10, 25, 28, 33, 39, 62, 70 |
| Rv2490c | *PE_PGRS43* | PE-PGRS FAMILY PROTEIN | 40, 41, 68, 80, 82, 94, 129, 132, 140, 145, 152, 154, 155, 158, 164, 166, 170, 175, 178, 179, 189, 192, 201, 202, 214, 216, 219, 226, 227, 230, 242, 250, 257, 266, 272, 273, 285, 287, 291, 304, 306, 310, 313, 315, 318, 321, 322, 324, 325, 327, 330, 336, 341, 342, 345, 347, 348, 351, 353, 354, 357, 360, 362, 366, 378, 381, 384, 396, 398, 399, 407, 408, 411, 413, 414, 426, 428, 433, 435, 446, 453, 457, 469, 470, 473, 476, 478, 482, 490, 491, 494, 498, 501, 514, 517, 524, 526, 529, 530, 537, 540, 542, 543, 545, 546, 551, 552, 554, 570, 576, 578, 579, 581, 587, 591, 605, 610, 611, 617, 622, 625, 637, 638, 646, 652, 653, 655, 662, 665, 675, 678, 691, 694, 696, 700, 706, 708, 709, 714, 717, 745, 748, 751, 753, 756, 758, 759, 764, 772, 775, 783, 784, 787, 789, 790, 792, 795, 805, 809, 811, 812, 817, 822, 824, 829, 840, 841, 844, 847, 850, 853, 856, 858, 859, 862, 864, 865, 867, 869, 879, 882, 885, 888, 890, 891, 894, 897, 903, 906, 909, 912, 921, 924, 932, 941, 943, 960, 962, 969, 972, 980, 981, 989, 1021, 1022, 1024, 1027, 1030, 1033, 1041, 1043, 1044, 1047, 1049, 1050, 1053, 1058, 1061, 1073, 1075, 1078, 1079, 1082, 1085, 1087, 1088, 1094, 1100, 1102, 1113, 1120, 1123, 1125, 1126, 1129, 1131, 1132, 1135, 1142, 1144, 1145, 1148, 1151, 1154, 1157, 1159, 1166, 1171, 1185, 1186, 1189, 1192, 1198, 1201, 1202, 1205, 1208, 1211, 1214, 1220, 1222, 1230, 1236, 1239, 1241, 1248, 1251, 1257, 1260, 1278, 1286, 1292, 1293, 1300, 1303, 1305, 1306, 1314, 1317, 1318, 1320, 1327, 1330, 1333, 1337, 1339, 1341, 1342, 1350, 1369, 1371, 1378, 1380, 1383, 1384, 1388, 1394, 1396, 1400, 1406, 1423, 1426, 1432, 1435, 1437, 1438, 1441, 1443, 1444, 1448, 1449, 1458, 1461, 1464, 1467, 1476, 1491, 1497, 1500, 1505, 1506, 1509, 1521, 1526, 1527, 1533, 1536, 1552, 1558, 1561, 1566, 1573, 1580, 1582, 1583, 1585, 1586, 1597, 1601, 1604, 1606, 1610, 1612, 1614, 1619, 1620, 1631, 1640, 1641, 1643, 1644, 1647, 1650 |
| Rv2491 | *-* | hypothetical protein Rv2491 | 6, 33, 61, 103, 127, 149, 186 |
| Rv2492 | *-* | hypothetical protein Rv2492 | 22 |
| Rv2493 | *-* | hypothetical protein Rv2493 | 12, 39, 55 |
| Rv2494 | *-* | hypothetical protein Rv2494 | 27, 42, 111, 120 |
| Rv2495c | *pdhC* | branched-chain alpha-keto acid dehydrogenase subunit E2 | 1, 45, 57, 64, 82, 106, 135, 143, 144, 154, 158, 162, 189, 252, 291, 300, 304, 346, 387 |
| Rv2496c | *pdhB* | PROBABLE PYRUVATE DEHYDROGENASE E1 COMPONENT (BETA SUBUNIT) PDHB (PYRUVATE DECARBOXYLASE) (PYRUVATE DEHYDROGENASE) (PYRUVIC DEHYDROGENASE) | 82, 127, 142, 161, 172, 201, 221, 236, 275, 292, 295, 314, 331 |
| Rv2497c | *pdhA* | PROBABLE PYRUVATE DEHYDROGENASE E1 COMPONENT (ALPHA SUBUNIT) PDHA (PYRUVATE DECARBOXYLASE) (PYRUVATE DEHYDROGENASE) (PYRUVIC DEHYDROGENASE) | 76, 80, 125, 154, 211, 246, 251, 270, 349 |
| Rv2498c | *citE* | PROBABLE CITRATE (PRO-3S)-LYASE (BETA SUBUNIT) CITE (CITRASE) (CITRATASE) (CITRITASE) (CITRIDESMOLASE) (CITRASE ALDOLASE) | 4, 5, 24, 45, 67, 82, 112, 114, 119, 143, 168, 170, 197, 235, 266 |
| Rv2499c | *-* | POSSIBLE OXIDASE REGULATORY-RELATED PROTEIN | 4, 47, 138, 147 |
| Rv2500c | *fadE19* | POSSIBLE ACYL-CoA DEHYDROGENASE FADE19 (MMGC) | 67, 70, 96, 128, 181, 184, 186, 201, 243, 264, 315, 352 |
| Rv2501c | *accA1* | PROBABLE ACETYL-/PROPIONYL-COENZYME A CARBOXYLASE ALPHA CHAIN (ALPHA SUBUNIT) ACCA1: BIOTIN CARBOXYLASE + BIOTIN CARBOXYL CARRIER PROTEIN (BCCP) | 9, 52, 72, 117, 123, 131, 136, 145, 160, 161, 163, 261, 270, 278, 345, 352, 353, 362, 363, 365, 375, 439, 461, 462, 480, 483, 486, 496, 542, 563, 602 |
| Rv2502c | *accD1* | PROBABLE ACETYL-/PROPIONYL-CoA CARBOXYLASE (BETA SUBUNIT) ACCD1 | 27, 29, 44, 70, 72, 86, 92, 107, 138, 139, 207, 216, 226, 235, 264, 345, 370, 380, 381, 392, 407, 418, 439, 443, 451, 456, 458 |
| Rv2503c | *scoB* | PROBABLE SUCCINYL-COA:3-KETOACID-COENZYME A TRANSFERASE (BETA SUBUNIT) SCOB (3-OXO-ACID:COA TRANSFERASE) (OXCT B) (SUCCINYL CoA:3-OXOACID CoA-TRANSFERASE) | 15, 82, 95, 112, 121, 129, 148, 165, 192 |
| Rv2504c | *scoA* | PROBABLE SUCCINYL-COA:3-KETOACID-COENZYME A TRANSFERASE (ALPHA SUBUNIT) SCOA (3-OXO ACID:CoA TRANSFERASE) (OXCT A) (SUCCINYL-COA:3-OXOACID-COENZYME A TRANSFERASE) | 6, 28, 41, 53, 108, 129, 139, 187, 197, 236 |
| Rv2505c | *fadD35* | acyl-CoA synthetase | 2, 15, 34, 97, 109, 123, 140, 180, 196, 226, 307, 311, 313, 330, 383, 398, 419, 428, 444, 526, 543 |
| Rv2506 | *-* | PROBABLE TRANSCRIPTIONAL REGULATORY PROTEIN (PROBABLY TETR-FAMILY) | 3, 9, 32, 52, 57, 85, 98, 205, 209 |
| Rv2507 | *-* | POSSIBLE CONSERVED PROLINE RICH MEMBRANE PROTEIN | 14, 19, 29, 45, 89, 113, 114, 139, 148, 167, 175, 183, 207, 234, 253, 261 |
| Rv2508c | *-* | PROBABLE CONSERVED INTEGRAL MEMBRANE LEUCINE AND ALANINE RICH PROTEIN | 23, 26, 51, 66, 95, 97, 117, 125, 153, 179, 183, 224, 232, 236, 297, 327, 331, 348, 386, 410, 413, 421, 435 |
| Rv2509 | *-* | PROBABLE SHORT-CHAIN TYPE DEHYDROGENASE/REDUCTASE | 3, 14, 29, 31, 38, 94, 106, 135, 136, 145, 157, 171, 175, 177, 217 |
| Rv2510c | *-* | hypothetical protein Rv2510c | 6, 10, 12, 58, 61, 63, 93, 101, 106, 112, 210, 381, 388, 418, 427, 488, 496, 515, 526 |
| Rv2511 | *orn* | oligoribonuclease | 12, 25, 49, 80, 98, 102, 175, 181, 213 |
| Rv2512c | *-* | IS1081 transposase | 25, 33, 44, 71, 157, 164, 187, 204, 209, 221, 224, 235, 239, 246, 305, 313, 322, 401 |
| Rv2513 | *-* | hypothetical protein Rv2513 | 65, 135 |
| Rv2514c | *-* | hypothetical protein Rv2514c | 35, 119 |
| Rv2515c | *-* | hypothetical protein Rv2515c | 20, 41, 71, 103, 146, 191, 199, 209, 274, 357, 389 |
| Rv2516c | *-* | hypothetical protein Rv2516c | 48, 123, 151, 156, 174, 227 |
| Rv2517c | *-* | hypothetical protein Rv2517c | 23 |
| Rv2518c | *lppS* | PROBABLE CONSERVED LIPOPROTEIN LPPS | 20, 25, 34, 36, 38, 62, 81, 101, 147, 157, 255, 330, 370, 402 |
| Rv2519 | *PE26* | PE FAMILY PROTEIN | 29, 40, 99, 136, 137, 144, 153, 157, 159, 160, 163, 171, 174, 175, 177, 179, 180, 196, 199, 224, 255, 281, 283, 309, 310, 333, 335, 356, 357, 374, 386, 404, 421, 447, 462, 479 |
| Rv2520c | *-* | POSSIBLE CONSERVED MEMBRANE PROTEIN |  |
| Rv2521 | *bcp* | PROBABLE BACTERIOFERRITIN COMIGRATORY PROTEIN BCP | 8, 65, 144 |
| Rv2522c | *-* | hypothetical protein Rv2522c | 11, 71, 82, 114, 136, 148, 154, 171, 181, 212, 230, 234, 267, 366, 378, 403, 413, 424, 429, 450 |
| Rv2523c | *acpS* | 4'-phosphopantetheinyl transferase | 22, 64, 97, 119, 121 |
| Rv2524c | *fas* | PROBABLE FATTY ACID SYNTHASE FAS (FATTY ACID SYNTHETASE) | 9, 12, 55, 111, 116, 176, 177, 185, 238, 267, 335, 353, 358, 370, 414, 435, 440, 447, 448, 465, 491, 502, 504, 513, 540, 567, 570, 571, 601, 615, 636, 666, 671, 694, 702, 722, 753, 756, 809, 825, 880, 884, 903, 911, 929, 944, 977, 1012, 1033, 1034, 1065, 1068, 1105, 1122, 1139, 1161, 1181, 1183, 1192, 1214, 1226, 1237, 1242, 1256, 1263, 1279, 1288, 1311, 1323, 1336, 1362, 1389, 1415, 1434, 1442, 1448, 1463, 1499, 1503, 1509, 1532, 1548, 1549, 1558, 1621, 1674, 1675, 1676, 1721, 1745, 1753, 1759, 1774, 1829, 1835, 1874, 1910, 1925, 1940, 1952, 1956, 1963, 1973, 1981, 2001, 2012, 2088, 2102, 2103, 2110, 2114, 2121, 2129, 2139, 2231, 2251, 2271, 2280, 2290, 2320, 2322, 2333, 2338, 2374, 2412, 2423, 2444, 2469, 2489, 2496, 2584, 2601, 2606, 2620, 2641, 2663, 2666, 2698, 2717, 2721, 2743, 2744, 2761, 2763, 2770, 2772, 2795, 2803, 2844, 2861, 2910, 2915, 2948, 2950, 2963, 2970, 2999, 3015, 3025, 3034 |
| Rv2525c | *-* | hypothetical protein Rv2525c | 29, 34, 44, 45, 49, 56, 71, 89, 111, 115, 128, 130, 131, 171, 207 |
| Rv2526 | *-* | hypothetical protein Rv2526 | 21, 38, 44, 58 |
| Rv2527 | *-* | hypothetical protein Rv2527 | 15, 21, 98, 104, 130 |
| Rv2528c | *mrr* | PROBABLE RESTRICTION SYSTEM PROTEIN MRR | 48, 68, 123, 128, 165, 183, 192, 233, 254 |
| Rv2529 | *-* | hypothetical protein Rv2529 | 55, 56, 86, 92, 114, 152, 155, 198, 223, 239, 263, 266, 267, 290, 300, 308, 340, 345, 401, 421, 458 |
| Rv2530c | *-* | hypothetical protein Rv2530c | 22, 75, 91, 112, 124, 131 |
| Rv2530A | *-* | hypothetical protein Rv2530A |  |
| Rv2531c | *-* | PROBABLE AMINO ACID DECARBOXYLASE | 13, 16, 45, 117, 180, 252, 313, 363, 381, 393, 414, 449, 450, 509, 572, 709, 772, 820, 835, 856, 905, 915 |
| Rv2532c | *-* | hypothetical protein Rv2532c | 25, 78, 116 |
| Rv2533c | *nusB* | transcription antitermination protein NusB | 7, 26, 38, 55, 65, 73, 85, 147 |
| Rv2534c | *efp* | elongation factor P | 55, 61, 137, 155, 167 |
| Rv2535c | *pepQ* | PROBABLE CYTOPLASMIC PEPTIDASE PEPQ | 16, 36, 63, 83, 86, 89, 124, 136, 151, 159, 195, 226, 258, 283, 311, 312, 325, 330, 332, 345, 353, 354 |
| Rv2536 | *-* | PROBABLE CONSERVED TRANSMEMBRANE PROTEIN | 6, 11, 32, 78, 82, 86, 102, 116, 150, 156, 163, 172, 184, 222 |
| Rv2537c | *aroD* | 3-dehydroquinate dehydratase | 21, 25, 65, 76, 77, 122 |
| Rv2538c | *aroB* | 3-dehydroquinate synthase | 23, 45, 50, 59, 75, 104, 107, 112, 121, 141, 144, 150, 181, 189, 207, 212, 232, 266, 282, 289, 319, 348, 353, 356 |
| Rv2539c | *aroK* | shikimate kinase | 12, 40, 78, 85, 92, 103, 110, 111, 120, 154 |
| Rv2540c | *aroF* | chorismate synthase | 5, 23, 45, 60, 69, 89, 95, 140, 186, 199, 219, 238, 248, 268, 272, 282, 294, 299, 330, 349, 370, 382, 395, 399 |
| Rv2541 | *-* | HYPOTHETICAL ALANINE RICH PROTEIN | 16, 22, 25, 35, 52, 59, 64, 84, 92, 103, 119, 123 |
| Rv2542 | *-* | hypothetical protein Rv2542 | 66, 80, 104, 109, 169, 177, 184, 193, 257, 384, 396, 400 |
| Rv2543 | *lppA* | PROBABLE CONSERVED LIPOPROTEIN LPPA | 26, 45, 64, 70, 72, 111, 137, 145, 187, 201 |
| Rv2544 | *lppB* | PROBABLE CONSERVED LIPOPROTEIN LPPB | 26, 45, 64, 70, 72, 137, 145, 187, 201 |
| Rv2545 | *-* | hypothetical protein Rv2545 | 76, 84, 88 |
| Rv2546 | *-* | hypothetical protein Rv2546 | 26, 46, 84, 99, 120, 123, 133 |
| Rv2547 | *-* | hypothetical protein Rv2547 | 17, 47, 62 |
| Rv2548 | *-* | hypothetical protein Rv2548 | 13, 16, 44, 74, 86, 98 |
| Rv2549c | *-* | hypothetical protein Rv2549c | 14, 21, 51, 58, 122 |
| Rv2550c | *-* | hypothetical protein Rv2550c | 68 |
| Rv2551c | *-* | hypothetical protein Rv2551c | 29, 41, 43, 49, 52, 55, 66, 71, 81, 84, 98, 103, 106 |
| Rv2552c | *aroE* | shikimate 5-dehydrogenase | 7, 25, 42, 70, 79, 109, 124, 127, 174, 182, 200, 204, 222, 229, 234, 251, 254, 265 |
| Rv2553c | *-* | PROBABLE CONSERVED MEMBRANE PROTEIN | 42, 43, 76, 78, 89, 115, 120, 127, 140, 261, 269, 274, 357, 362, 369 |
| Rv2554c | *-* | Holliday junction resolvase-like protein | 11, 30, 56, 67, 144, 161 |
| Rv2555c | *alaS* | alanyl-tRNA synthetase | 38, 86, 97, 112, 113, 114, 142, 152, 164, 167, 177, 183, 185, 207, 229, 263, 287, 298, 303, 317, 385, 391, 416, 440, 459, 465, 494, 495, 497, 527, 529, 537, 558, 573, 591, 687, 710, 735, 761, 777, 781, 783, 799, 800, 803, 815, 844, 867, 869, 883, 887 |
| Rv2556c | *-* | hypothetical protein Rv2556c | 5, 22, 38, 45, 47 |
| Rv2557 | *-* | hypothetical protein Rv2557 | 1, 5, 34, 45, 64, 75, 98, 167, 173, 201 |
| Rv2558 | *-* | hypothetical protein Rv2558 | 4, 13, 17, 56, 75, 104, 109, 177 |
| Rv2559c | *-* | recombination factor protein RarA | 12, 18, 26, 51, 75, 88, 100, 196, 217, 225, 237, 276, 282, 293, 315, 325, 351, 363, 371, 374, 387, 429, 436 |
| Rv2560 | *-* | PROBABLE PROLINE AND GLYCINE RICH TRANSMEMBRANE PROTEIN | 15, 26, 47, 56, 67, 110, 117, 129, 135, 149, 170, 174, 202, 220, 289, 319 |
| Rv2561 | *-* | hypothetical protein Rv2561 | 13, 56, 75 |
| Rv2562 | *-* | hypothetical protein Rv2562 | 118, 124 |
| Rv2563 | *-* | PROBABLE GLUTAMINE-TRANSPORT TRANSMEMBRANE PROTEIN ABC TRANSPORTER | 22, 33, 59, 88, 108, 117, 165, 275, 292 |
| Rv2564 | *glnQ* | PROBABLE GLUTAMINE-TRANSPORT ATP-BINDING PROTEIN ABC TRANSPORTER GLNQ | 1, 14, 41, 43, 53, 60, 76, 100, 112, 118, 144, 170, 232, 263, 270 |
| Rv2565 | *-* | hypothetical protein Rv2565 | 2, 16, 45, 53, 83, 94, 114, 145, 163, 193, 227, 302, 311, 313, 330, 338, 347, 356, 361, 381, 413, 418, 440, 500, 508, 528, 531, 561 |
| Rv2566 | *-* | LONG CONSERVED HYPOTHETICAL PROTEIN | 8, 10, 16, 19, 73, 159, 201, 212, 234, 239, 258, 276, 289, 302, 309, 358, 367, 400, 415, 422, 457, 471, 486, 503, 509, 523, 526, 564, 625, 658, 689, 699, 733, 735, 736, 744, 748, 778, 816, 818, 838, 867, 954, 965, 979, 983, 1060, 1064, 1075, 1100 |
| Rv2567 | *-* | CONSERVED HYPOTHETICAL ALANINE AND LEUCINE RICH PROTEIN | 6, 18, 22, 38, 88, 98, 118, 127, 164, 169, 209, 270, 343, 354, 382, 388, 411, 455, 460, 461, 481, 489, 492, 528, 588, 594, 598, 602, 642, 691, 697, 700, 721, 726, 739, 770, 832, 845, 859, 866, 867 |
| Rv2568c | *-* | hypothetical protein Rv2568c | 8, 17, 22, 65, 83, 84, 135, 216, 255, 259, 311 |
| Rv2569c | *-* | hypothetical protein Rv2569c | 36, 60, 101, 106, 118, 119, 122, 226, 249, 274, 285 |
| Rv2570 | *-* | hypothetical protein Rv2570 | 16, 48, 54 |
| Rv2571c | *-* | PROBABLE TRANSMEMBRANE ALANINE AND VALINE AND LEUCINE RICH PROTEIN | 10, 33, 94, 115, 128, 131, 184, 211, 271, 290, 293, 308, 313 |
| Rv2572c | *aspS* | aspartyl-tRNA synthetase | 7, 16, 23, 33, 75, 92, 137, 149, 187, 322, 341, 344, 358, 371, 381, 389, 436, 438, 449, 456, 470, 489, 533, 543, 561, 562, 576, 583 |
| Rv2573 | *-* | 2-dehydropantoate 2-reductase | 3, 10, 16, 31, 42, 59, 98, 116, 119, 125, 127, 133, 137, 139, 169, 273 |
| Rv2574 | *-* | hypothetical protein Rv2574 | 13, 61, 70, 113, 127, 161 |
| Rv2575 | *-* | POSSIBLE CONSERVED MEMBRANE GLYCINE RICH PROTEIN | 18, 20, 28, 46, 63, 72, 87, 100, 111, 116, 118, 154, 203, 218, 237, 251, 264, 287 |
| Rv2576c | *-* | POSSIBLE CONSERVED MEMBRANE PROTEIN | 2, 8, 30, 32, 35, 95, 102, 115, 117, 142, 144 |
| Rv2577 | *-* | hypothetical protein Rv2577 | 38, 40, 59, 142, 154, 157, 176, 196, 283, 290, 291, 315, 319, 349, 353, 382, 395, 429, 449, 456, 494, 495, 526 |
| Rv2578c | *-* | hypothetical protein Rv2578c | 57, 69, 106, 152, 175, 213, 217, 218, 236, 249, 253, 267, 291 |
| Rv2579 | *dhaA* | haloalkane dehalogenase | 17, 175, 197, 217, 245, 250, 269, 297 |
| Rv2580c | *hisS* | histidyl-tRNA synthetase | 37, 50, 86, 87, 100, 121, 152, 258, 266, 282, 295, 353, 373, 376, 391 |
| Rv2581c | *-* | POSIBLE GLYOXALASE II (HYDROXYACYLGLUTATHIONE HYDROLASE) (GLX II) | 3, 21, 50, 64, 80, 125, 139, 159, 174, 175, 177, 200 |
| Rv2582 | *ppiB* | PROBABLE PEPTIDYL-PROLYL CIS-TRANS ISOMERASE B PPIB (CYCLOPHILIN) (PPIASE) (ROTAMASE) (PEPTIDYLPROLYL ISOMERASE) | 93, 95, 130, 195, 204, 234, 242, 284, 287, 289, 295 |
| Rv2583c | *relA* | PROBABLE GTP PYROPHOSPHOKINASE RELA (ATP:GTP 3'-PYROPHOSPHOTRANSFERASE) (PPGPP SYNTHETASE I) ((P)PPGPP SYNTHETASE) (GTP DIPHOSPHOKINASE) | 22, 53, 100, 127, 137, 170, 181, 260, 282, 387, 393, 412, 435, 465, 516, 550, 561, 592, 613, 646, 674, 687, 741, 788 |
| Rv2584c | *apt* | adenine phosphoribosyltransferase | 3, 69, 87, 98, 109, 131, 149, 175, 176, 188, 194, 206 |
| Rv2585c | *-* | POSSIBLE CONSERVED LIPOPROTEIN | 11, 18, 23, 28, 57, 60, 70, 129, 133, 141, 176, 195, 292, 312, 347, 370, 381, 383, 420, 432, 449, 453, 455, 458, 470, 497, 507 |
| Rv2586c | *secF* | preprotein translocase subunit SecF | 6, 37, 52, 54, 70, 94, 130, 183, 228, 229, 371, 384, 390, 406, 433, 438 |
| Rv2587c | *secD* | preprotein translocase subunit SecD | 30, 35, 45, 52, 83, 99, 112, 128, 144, 147, 191, 240, 258, 268, 282, 304, 331, 332, 338, 345, 348, 452, 479, 498 |
| Rv2588c | *yajC* | preprotein translocase subunit YajC | 40, 68, 105 |
| Rv2589 | *gabT* | 4-aminobutyrate aminotransferase | 14, 16, 31, 44, 45, 124, 140, 143, 163, 171, 205, 223, 234, 265, 270, 305, 317, 320, 324, 332, 376, 387, 394, 402, 406, 407, 414 |
| Rv2590 | *fadD9* | PROBABLE FATTY-ACID-CoA LIGASE FADD9 (FATTY-ACID-CoA SYNTHETASE) (FATTY-ACID-CoA SYNTHASE) | 22, 37, 94, 99, 105, 147, 176, 201, 207, 221, 248, 275, 303, 305, 374, 382, 384, 388, 413, 425, 461, 470, 482, 548, 575, 601, 640, 653, 661, 720, 734, 755, 773, 776, 820, 834, 841, 869, 890, 899, 951, 963, 982, 999, 1001, 1005, 1072, 1074, 1122 |
| Rv2591 | *PE_PGRS44* | PE-PGRS FAMILY PROTEIN | 41, 68, 81, 82, 110, 133, 143, 151, 152, 156, 158, 161, 168, 173, 176, 179, 180, 182, 183, 185, 186, 195, 196, 198, 199, 206, 211, 212, 214, 215, 224, 229, 230, 240, 241, 244, 247, 254, 256, 257, 260, 267, 269, 270, 272, 273, 277, 284, 287, 289, 290, 297, 302, 303, 305, 314, 322, 324, 330, 335, 336, 348, 350, 351, 354, 360, 361, 364, 367, 369, 379, 386, 391, 395, 398, 409, 410, 412, 416, 427, 430, 433, 437, 444, 445, 447, 448, 450, 451, 462, 465, 469, 480, 483, 492, 495, 498, 514, 517, 523, 525, 526, 528 |
| Rv2592c | *ruvB* | Holliday junction DNA helicase B | 10, 51, 61, 76, 94, 121, 168, 178, 186, 196, 197, 208, 277, 294, 316, 321 |
| Rv2593c | *ruvA* | Holliday junction DNA helicase motor protein | 5, 20, 21, 47, 77, 106, 135, 136, 173 |
| Rv2594c | *ruvC* | Holliday junction resolvase | 7, 12, 26, 81, 84, 85, 116, 123, 185 |
| Rv2595 | *-* | hypothetical protein Rv2595 | 23, 44 |
| Rv2596 | *-* | hypothetical protein Rv2596 | 10, 21, 79, 91, 107 |
| Rv2597 | *-* | PROBABLE MEMBRANE PROTEIN | 14, 33, 54, 68, 87, 118, 134, 137, 149, 150, 161, 186, 192, 199 |
| Rv2598 | *-* | hypothetical protein Rv2598 | 24, 71, 81, 112, 116, 120, 124, 127, 130, 136, 150, 151 |
| Rv2599 | *-* | PROBABLE CONSERVED MEMBRANE PROTEIN | 9, 15, 54, 108, 129, 136, 139 |
| Rv2600 | *-* | PROBABLE CONSERVED INTEGRAL MEMBRANE PROTEIN | 11, 45, 58, 116, 128 |
| Rv2601 | *speE* | spermidine synthase | 22, 30, 47, 62, 93, 96, 121, 125, 141, 147, 150, 168, 184, 189, 196, 197, 210, 216, 222, 241, 267, 310, 323, 335, 350, 370, 372, 406, 412, 413, 439, 440, 471, 490, 495 |
| Rv2601A | *-* | hypothetical protein Rv2601A | 35, 42, 59, 75, 87 |
| Rv2602 | *-* | hypothetical protein Rv2602 | 14, 32, 73, 84, 120, 130, 136 |
| Rv2603c | *-* | hypothetical protein Rv2603c | 1, 20, 39, 43, 71, 73, 78, 109, 159, 177, 190, 245 |
| Rv2604c | *-* | hypothetical protein Rv2604c | 24, 47, 93, 173, 180, 195 |
| Rv2605c | *tesB2* | PROBABLE ACYL-CoA THIOESTERASE II TESB2 (TEII) | 37, 139, 242, 256, 273 |
| Rv2606c | *-* | pyridoxine biosynthesis protein | 8, 15, 23, 41, 53, 60, 76, 113, 158, 215, 216, 222, 240, 245, 252, 273, 296 |
| Rv2607 | *pdxH* | pyridoxamine 5'-phosphate oxidase | 28, 54, 85, 95, 188 |
| Rv2608 | *PPE42* | PPE FAMILY PROTEIN | 16, 39, 49, 82, 86, 134, 148, 155, 184, 189, 232, 242, 263, 268, 273, 299, 302, 316, 336, 386, 412, 416, 445, 457, 526, 547, 563 |
| Rv2609c | *-* | PROBABLE CONSERVED MEMBRANE PROTEIN | 6, 62, 71, 76, 94, 101, 105, 150, 174, 188, 195, 229, 238, 339, 343 |
| Rv2610c | *pimA* | ALPHA-MANNOSYLTRANSFERASE PIMA | 14, 30, 44, 52, 234, 253, 270, 271, 285, 319, 342, 367 |
| Rv2611c | *-* | lipid A biosynthesis lauroyl acyltransferase | 2, 20, 28, 47, 56, 125, 137, 143, 185, 186, 216, 240, 254, 268, 287, 290 |
| Rv2612c | *pgsA1* | PROBABLE PI SYNTHASE PGSA1 (PHOSPHATIDYLINOSITOL SYNTHASE) (CDP-DIACYLGLYCEROL--INOSITOL3-PHOSPHATIDYLTRANSFERASE) | 38, 77, 79, 101, 140, 143, 146, 160, 162, 202 |
| Rv2613c | *-* | hypothetical protein Rv2613c | 88, 144 |
| Rv2614c | *thrS* | threonyl-tRNA synthetase | 3, 6, 12, 24, 27, 30, 40, 73, 87, 184, 189, 208, 228, 246, 287, 296, 362, 383, 406, 488, 497, 538, 642, 643, 688 |
| Rv2614A | *-* | hypothetical protein Rv2614A | 13, 43, 68, 70 |
| Rv2615c | *PE_PGRS45* | PE-PGRS FAMILY PROTEIN | 13, 26, 29, 39, 50, 111, 126, 129, 130, 133, 140, 143, 148, 151, 152, 154, 155, 158, 166, 167, 170, 178, 182, 184, 185, 187, 195, 197, 198, 201, 206, 213, 219, 222, 227, 228, 230, 231, 233, 236, 237, 242, 246, 250, 253, 256, 263, 266, 268, 269, 282, 291, 294, 300, 303, 305, 306, 308, 313, 322, 325, 330, 331, 338, 340, 341, 344, 346, 347, 350, 360, 362, 365, 369, 379, 383, 385, 386, 392, 396, 398, 399, 402, 405, 408, 420, 423, 425, 436, 438, 439, 441, 450, 453 |
| Rv2616 | *-* | hypothetical protein Rv2616 | 20, 24, 38, 46, 59, 69, 73, 99, 112, 145, 146 |
| Rv2617c | *-* | PROBABLE TRANSMEMBRANE PROTEIN | 56, 64, 66, 81, 101, 109, 111, 139 |
| Rv2618 | *-* | hypothetical protein Rv2618 | 55, 58, 60, 62, 65, 102, 123, 130, 133, 206 |
| Rv2619c | *-* | hypothetical protein Rv2619c | 9, 21, 45, 70, 71, 80 |
| Rv2620c | *-* | PROBABLE CONSERVED TRANSMEMBRANE PROTEIN | 2, 76, 106, 114 |
| Rv2621c | *-* | POSSIBLE TRANSCRIPTIONAL REGULATORY PROTEIN | 33, 36, 64, 66, 102, 113, 127, 175 |
| Rv2622 | *-* | POSSIBLE METHYLTRANSFERASE (METHYLASE) | 7, 37, 38, 67, 97, 99, 110, 207, 231, 239 |
| Rv2623 | *TB31.7* | hypothetical protein Rv2623 | 18, 133, 175, 204, 265, 269 |
| Rv2624c | *-* | hypothetical protein Rv2624c | 24, 71, 75, 88, 91, 184, 240, 244, 254 |
| Rv2625c | *-* | PROBABLE CONSERVED TRANSMEMBRANE ALANINE AND LEUCINE RICH PROTEIN | 10, 76, 89, 92, 95, 103, 130, 183, 190, 227, 242, 251, 258, 322, 330 |
| Rv2626c | *-* | hypothetical protein Rv2626c | 2, 21, 23, 38, 66 |
| Rv2627c | *-* | hypothetical protein Rv2627c | 22, 31, 93, 109, 219, 230, 272, 306, 376, 411 |
| Rv2628 | *-* | hypothetical protein Rv2628 | 8, 19, 48, 76 |
| Rv2629 | *-* | hypothetical protein Rv2629 | 10, 78, 121, 126, 155, 156, 198, 224, 241, 265, 301, 335, 341, 363 |
| Rv2630 | *-* | hypothetical protein Rv2630 | 13, 84, 96 |
| Rv2631 | *-* | hypothetical protein Rv2631 | 30, 46, 54, 86, 107, 130, 148, 177, 193, 241, 269, 330, 341, 344, 352, 365, 370, 386 |
| Rv2632c | *-* | hypothetical protein Rv2632c |  |
| Rv2633c | *-* | hypothetical protein Rv2633c | 57, 59, 88, 114, 141 |
| Rv2634c | *PE_PGRS46* | PE-PGRS FAMILY PROTEIN | 42, 107, 130, 134, 141, 144, 156, 165, 168, 178, 181, 187, 195, 197, 210, 213, 224, 228, 231, 233, 240, 241, 244, 246, 250, 259, 261, 266, 267, 268, 279, 281, 283, 291, 295, 305, 313, 316, 320, 322, 323, 330, 332, 333, 345, 348, 351, 354, 362, 366, 371, 381, 389, 397, 404, 409, 411, 412, 419, 422, 425, 437, 438, 443, 447, 451, 453, 454, 457, 460, 469, 470, 474, 483, 494, 510, 513, 516, 527, 530, 532, 535, 542, 549, 551, 554, 555, 558, 560, 565, 566, 570, 573, 576, 586, 591, 592, 598, 601, 603, 604, 609, 613, 616, 628, 631, 632, 635, 637, 641, 646, 647, 652, 653, 659, 660, 666, 668, 672, 681, 683, 684, 686, 687, 690, 691, 693, 696, 697, 706, 707, 709, 712, 721, 724, 727, 730, 732, 735, 736, 739, 745, 750, 751, 761, 770 |
| Rv2635 | *-* | hypothetical protein Rv2635 | 2, 15, 41, 52 |
| Rv2636 | *-* | hypothetical protein Rv2636 | 29, 46, 163, 168, 191 |
| Rv2637 | *dedA* | POSSIBLE TRANSMEMBRANE PROTEIN DEDA | 32, 40, 57, 96, 127, 143, 148, 153, 154, 163, 166, 185, 189, 212, 215 |
| Rv2638 | *-* | hypothetical protein Rv2638 | 36, 45, 63, 65, 70, 78, 89, 101, 142 |
| Rv2639c | *-* | hypothetical protein Rv2639c | 62, 70, 97, 107 |
| Rv2640c | *-* | POSSIBLE TRANSCRIPTIONAL REGULATORY PROTEIN (PROBABLY ARSR-FAMILY) | 10, 55, 60, 84 |
| Rv2641 | *cadI* | CADMIUM INDUCIBLE PROTEIN CADI | 14, 27, 32, 52, 54, 55, 81, 104, 125, 150 |
| Rv2642 | *-* | POSSIBLE TRANSCRIPTIONAL REGULATORY PROTEIN (PROBABLY ARSR-FAMILY) | 26, 53, 54, 63, 117, 119 |
| Rv2643 | *arsC* | PROBABLE ARSENIC-TRANSPORT INTEGRAL MEMBRANE PROTEIN ARSC | 7, 32, 33, 42, 46, 84, 121, 148, 165, 207, 256, 279, 300, 321, 326, 353, 374, 380, 381, 388, 397, 401, 402, 411, 421, 429, 430, 444, 464, 465, 493 |
| Rv2644c | *-* | hypothetical protein Rv2644c |  |
| Rv2645 | *-* | hypothetical protein Rv2645 | 24, 57, 70, 81, 123 |
| Rv2646 | *-* | PROBABLE INTEGRASE | 2, 59, 84, 85, 102, 110, 114, 174, 197, 200, 207, 212, 218, 240, 256, 257, 261, 274, 325, 329 |
| Rv2647 | *-* | hypothetical protein Rv2647 | 33, 53, 93, 120 |
| Rv2648 | *-* | PROBABLE TRANSPOSASE FOR INSERTION SEQUENCE ELEMENT IS6110 | 24, 60, 64, 71 |
| Rv2649 | *-* | PROBABLE TRANSPOSASE FOR INSERTION SEQUENCE ELEMENT IS6110 | 7, 84, 121, 125, 141, 275, 289, 305, 325, 326 |
| Rv2650c | *-* | POSSIBLE phiRv2 PROPHAGE PROTEIN | 22, 36, 60, 73, 80, 84, 85, 121, 128, 144, 173, 179, 182, 193, 194, 195, 282, 283, 307, 309, 315, 319, 322, 324, 330, 339, 374, 392, 410, 442, 448, 451, 477 |
| Rv2651c | *-* | POSSIBLE phiRv2 PROPHAGE PROTEASE | 54, 141, 152 |
| Rv2652c | *-* | PROBABLE phiRv2 PROPHAGE PROTEIN | 5, 10, 36, 50, 61, 70, 159, 163 |
| Rv2653c | *-* | POSSIBLE phiRv2 PROPHAGE PROTEIN | 12, 35 |
| Rv2654c | *-* | POSSIBLE phiRv2 PROPHAGE PROTEIN | 1, 12, 27, 31, 34, 40, 65, 68 |
| Rv2655c | *-* | POSSIBLE phiRv2 PROPHAGE PROTEIN | 78, 140, 145, 159, 177, 228, 235, 261, 263, 269, 273, 279, 313, 328, 335, 390, 393, 402, 406, 439, 454, 455 |
| Rv2656c | *-* | POSSIBLE phiRv2 PROPHAGE PROTEIN | 1, 4, 20, 32, 51, 59, 76, 94, 104, 106 |
| Rv2657c | *-* | PROBABLE phiRv2 PROPHAGE PROTEIN | 19, 37, 58, 62, 84 |
| Rv2658c | *-* | POSSIBLE PROPHAGE PROTEIN | 29, 30, 31, 70, 118 |
| Rv2659c | *-* | PROBABLE phiRv2 PROPHAGE INTEGRASE | 3, 19, 27, 72, 120, 129, 150, 169, 178, 262, 274, 306, 307, 320, 328, 344, 346, 353, 362 |
| Rv2660c | *-* | hypothetical protein Rv2660c | 59 |
| Rv2661c | *-* | hypothetical protein Rv2661c | 49, 78 |
| Rv2662 | *-* | hypothetical protein Rv2662 | 50, 65 |
| Rv2663 | *-* | hypothetical protein Rv2663 | 45 |
| Rv2664 | *-* | hypothetical protein Rv2664 | 31, 45, 52, 53, 70 |
| Rv2665 | *-* | HYPOTHETICAL ARGININE RICH PROTEIN | 23, 77, 79 |
| Rv2666 | *-* | truncated IS1081 transposase | 25, 33, 44, 71, 157, 164, 187, 204, 209, 221, 224, 235, 239, 246 |
| Rv2667 | *clpC2* | POSSIBLE ATP-DEPENDENT PROTEASE ATP-BINDING SUBUNIT CLPC2 | 6, 36, 75, 112, 116, 135, 172, 216, 235, 240, 245, 250 |
| Rv2668 | *-* | POSSIBLE EXPORTED ALANINE AND VALINE RICH PROTEIN | 13, 28, 44, 70, 107, 129, 141, 163 |
| Rv2669 | *-* | hypothetical protein Rv2669 | 7, 10, 53, 68, 88, 90, 101 |
| Rv2670c | *-* | hypothetical protein Rv2670c | 20, 67, 97, 125, 146, 162, 195, 211, 254, 257, 291, 296, 320, 327, 341 |
| Rv2671 | *ribD* | hypothetical protein Rv2671 | 50, 58, 62, 64, 93, 122, 165, 184, 193, 220, 230, 247 |
| Rv2672 | *-* | POSSIBLE SECRETED PROTEASE | 13, 27, 64, 89, 107, 135, 168, 209, 223, 230, 286, 327, 330, 334, 335, 348, 369, 373, 380, 473, 475, 484, 526 |
| Rv2673 | *-* | POSSIBLE CONSERVED INTEGRAL MEMBRANE PROTEIN | 13, 21, 27, 32, 43, 77, 97, 123, 129, 145, 147, 193, 226, 237, 289, 305, 417 |
| Rv2674 | *-* | hypothetical protein Rv2674 | 27, 34, 66, 109, 132 |
| Rv2675c | *-* | hypothetical protein Rv2675c | 27, 58, 60, 68, 75, 129, 182, 207, 233 |
| Rv2676c | *-* | hypothetical protein Rv2676c | 24, 48, 53, 101, 125, 158, 160, 216 |
| Rv2677c | *hemY* | protoporphyrinogen oxidase | 10, 41, 50, 85, 131, 143, 167, 182, 187, 192, 209, 246, 254, 255, 285, 303, 304, 306, 312, 317, 337, 398, 409 |
| Rv2678c | *hemE* | uroporphyrinogen decarboxylase | 13, 16, 31, 86, 106, 111, 144, 157, 210, 247, 260, 281, 287, 310, 324, 327, 343 |
| Rv2679 | *echA15* | enoyl-CoA hydratase | 31, 63, 71, 72, 136, 149, 178, 200, 209, 232, 255, 262, 271, 273 |
| Rv2680 | *-* | hypothetical protein Rv2680 | 2, 3, 19, 142, 146, 157, 193, 198 |
| Rv2681 | *-* | CONSERVED HYPOTHETICAL ALANINE RICH PROTEIN | 25, 26, 40, 42, 48, 50, 66, 79, 90, 113, 137, 141, 227, 249, 281, 299, 316, 346, 396, 404, 420, 429 |
| Rv2682c | *dxs1* | 1-deoxy-D-xylulose-5-phosphate synthase | 5, 7, 22, 34, 68, 79, 92, 130, 147, 148, 159, 214, 226, 304, 311, 337, 344, 347, 372, 415, 418, 438, 494, 571, 616, 620, 624 |
| Rv2683 | *-* | hypothetical protein Rv2683 | 7, 54, 55, 85, 101, 129, 153 |
| Rv2684 | *arsA* | PROBABLE ARSENIC-TRANSPORT INTEGRAL MEMBRANE PROTEIN ARSA | 28, 50, 79, 83, 88, 149, 220, 282, 294, 308, 309, 315, 323, 370, 374 |
| Rv2685 | *arsB1* | PROBABLE ARSENIC-TRANSPORT INTEGRAL MEMBRANE PROTEIN ARSB1 | 10, 36, 50, 73, 89, 102, 147, 220, 226, 255, 282, 294, 308, 309, 327, 348, 370, 374, 408 |
| Rv2686c | *-* | PROBABLE ANTIBIOTIC-TRANSPORT INTEGRAL MEMBRANE LEUCINE AND ALANINE AND VALINE RICH PROTEIN ABC TRANSPORTER | 13, 41, 71, 82, 86, 135, 186 |
| Rv2687c | *-* | PROBABLE ANTIBIOTIC-TRANSPORT INTEGRAL MEMBRANE LEUCINE AND VALINE RICH PROTEIN ABC TRANSPORTER | 22, 26, 63, 123, 169 |
| Rv2688c | *-* | PROBABLE ANTIBIOTIC-TRANSPORT ATP-BINDING PROTEIN ABC TRANSPORTER | 20, 43, 53, 72, 107, 120, 131, 174, 194, 207, 249, 254, 295 |
| Rv2689c | *-* | CONSERVED HYPOTHETICAL ALANINE AND VALINE AND GLYCINE RICH PROTEIN | 3, 14, 17, 20, 39, 48, 77, 82, 125, 160, 256, 258, 261, 283, 296, 298, 301, 313, 324, 325, 348 |
| Rv2690c | *-* | PROBABLE CONSERVED INTEGRAL MEMBRANE ALANINE AND VALINE AND LEUCINE RICH PROTEIN | 5, 57, 73, 92, 107, 108, 168, 181, 205, 283, 337, 340, 394, 449, 463, 627, 653 |
| Rv2691 | *ceoB* | TRK SYSTEM POTASSIUM UPTAKE PROTEIN CEOB | 7, 33, 43, 69, 73, 135, 142, 168, 180, 192, 202, 205 |
| Rv2692 | *ceoC* | TRK SYSTEM POTASSIUM UPTAKE PROTEIN CEOC | 5, 37, 69, 71, 160, 173, 180, 195 |
| Rv2693c | *-* | PROBABLE CONSERVED INTEGRAL MEMBRANE ALANINE AND LEUCINE RICH PROTEIN | 14, 15, 36, 37, 41, 69, 72, 124, 182, 185, 206, 219 |
| Rv2694c | *-* | hypothetical protein Rv2694c | 32, 42, 88, 99 |
| Rv2695 | *-* | CONSERVED HYPOTHETICAL ALANINE RICH PROTEIN | 15, 27, 33, 53, 58, 67, 92, 98, 106, 180, 217, 226 |
| Rv2696c | *-* | CONSERVED HYPOTHETICAL ALANINE AND GLYCINE AND VALINE RICH PROTEIN | 15, 38, 80, 91, 116, 117, 124, 153, 154, 235, 242, 257 |
| Rv2697c | *dut* | deoxyuridine 5'-triphosphate nucleotidohydrolase | 11, 24, 64, 99, 106, 132, 139, 147, 148 |
| Rv2698 | *-* | PROBABLE CONSERVED ALANINE RICH TRANSMEMBRANE PROTEIN | 58, 72, 73, 79, 85 |
| Rv2699c | *-* | hypothetical protein Rv2699c | 42, 54, 98 |
| Rv2700 | *-* | POSSIBLE CONSERVED SECRETED ALANINE RICH PROTEIN | 8, 24, 84, 98, 101, 104, 120, 128, 147, 196, 204 |
| Rv2701c | *suhB* | POSSIBLE EXTRAGENIC SUPPRESSOR PROTEIN SUHB | 7, 26, 75, 87, 127, 139, 166, 178, 242, 257, 266, 268 |
| Rv2702 | *ppgK* | POLYPHOSPHATE GLUCOKINASE PPGK (POLYPHOSPHATE-GLUCOSE PHOSPHOTRANSFERASE) | 3, 25, 27, 31, 38, 81, 91, 113, 125, 126, 135, 150, 154, 174, 182, 219, 220, 242, 248, 249 |
| Rv2703 | *sigA* | RNA polymerase sigma factor RpoD | 16, 21, 24, 26, 30, 39, 52, 59, 78, 82, 84, 91, 107, 131, 139, 178, 181, 198, 221, 223, 268, 274, 310 |
| Rv2704 | *-* | hypothetical protein Rv2704 | 9, 35, 37, 42, 59, 103, 108, 131, 139 |
| Rv2705c | *-* | hypothetical protein Rv2705c | 13, 24, 48, 103, 124 |
| Rv2706c | *-* | hypothetical protein Rv2706c | 13, 78 |
| Rv2707 | *-* | PROBABLE CONSERVED TRANSMEMBRANE ALANINE AND LEUCINE RICH PROTEIN | 67, 102, 161, 190, 265, 277 |
| Rv2708c | *-* | hypothetical protein Rv2708c | 21 |
| Rv2709 | *-* | PROBABLE CONSERVED TRANSMEMBRANE PROTEIN | 35, 60, 65, 122 |
| Rv2710 | *sigB* | RNA polymerase sigma factor SigB | 21, 41, 42, 53, 151, 194, 197, 240 |
| Rv2711 | *ideR* | IRON-DEPENDENT REPRESSOR AND ACTIVATOR IDER | 36, 158, 186, 201 |
| Rv2712c | *-* | hypothetical protein Rv2712c | 17, 65, 71, 119, 121, 130, 133, 174, 209, 283, 289, 328 |
| Rv2713 | *sthA* | soluble pyridine nucleotide transhydrogenase | 13, 17, 33, 38, 60, 136, 152, 182, 239, 254, 281, 288, 314, 317, 327, 382, 386, 427, 451 |
| Rv2714 | *-* | CONSERVED HYPOTHETICAL ALANINE AND LEUCINE RICH PROTEIN | 17, 49, 56, 132, 165, 222, 224, 235, 298 |
| Rv2715 | *-* | POSSIBLE HYDROLASE | 33, 35, 112, 136, 173, 259, 297, 303, 313, 319 |
| Rv2716 | *-* | hypothetical protein Rv2716 | 54, 58, 73, 98, 110, 134, 138, 156, 166, 177, 179, 215 |
| Rv2717c | *-* | hypothetical protein Rv2717c | 21, 86, 99, 120, 125, 156 |
| Rv2718c | *-* | hypothetical protein Rv2718c | 42, 64, 93, 132 |
| Rv2719c | *-* | POSSIBLE CONSERVED MEMBRANE PROTEIN | 34, 49, 75, 93, 94, 104 |
| Rv2720 | *lexA* | LexA repressor | 2, 25, 72, 77, 86, 105, 106, 144, 182, 183 |
| Rv2721c | *-* | POSSIBLE CONSERVED TRANSMEMBRANE ALANINE AND GLYCINE RICH PROTEIN | 24, 46, 77, 78, 87, 104, 120, 123, 133, 152, 157, 183, 188, 222, 235, 250, 269, 286, 315, 334, 339, 345, 370, 371, 387, 395, 413, 426, 453, 468, 500, 510, 512, 534, 615, 621, 626, 669 |
| Rv2722 | *-* | hypothetical protein Rv2722 | 14, 18, 69 |
| Rv2723 | *-* | PROBABLE CONSERVED INTEGRAL MEMBRANE PROTEIN | 3, 15, 59, 115, 122, 150, 231, 233, 287, 313, 321, 336, 355, 359, 375, 390 |
| Rv2724c | *fadE20* | PROBABLE ACYL-CoA DEHYDROGENASE FADE20 | 45, 51, 66, 85, 89, 122, 127, 134, 136, 194, 201, 215, 257, 322 |
| Rv2725c | *hflX* | PROBABLE GTP-BINDING PROTEIN HFLX | 100, 101, 137, 154, 168, 201, 212, 215, 220, 225, 227, 290, 292, 411, 417, 420, 437, 440, 450 |
| Rv2726c | *dapF* | diaminopimelate epimerase | 25, 49, 50, 86, 114, 203, 217, 227, 232, 235, 244, 253, 276 |
| Rv2727c | *miaA* | tRNA delta(2)-isopentenylpyrophosphate transferase | 11, 21, 42, 72, 81, 100, 116, 150, 171, 176, 178, 234, 246, 250, 259, 290 |
| Rv2728c | *-* | CONSERVED HYPOTHETICAL ALANINE RICH PROTEIN | 9, 18, 20, 30, 48, 95, 121, 157, 158, 165, 177, 187, 200, 225 |
| Rv2729c | *-* | PROBABLE CONSERVED INTEGRAL MEMBRANE ALANINE VALINE AND LEUCINE RICH PROTEIN | 13, 17, 27, 32, 58, 84, 113, 127, 163, 200, 226, 254, 287, 288, 293 |
| Rv2730 | *-* | hypothetical protein Rv2730 | 27, 50, 80, 81, 94 |
| Rv2731 | *-* | CONSERVED HYPOTHETICAL ALANINE AND ARGININE RICH PROTEIN | 11, 29, 68, 78, 83, 108, 110, 155, 166, 169, 176, 183, 192, 207, 244, 267, 300, 311, 313, 344, 367, 418, 441 |
| Rv2732c | *-* | PROBABLE CONSERVED TRANSMEMBRANE PROTEIN | 7, 53, 57, 68, 71, 115, 117, 129, 130, 133, 135, 193, 199 |
| Rv2733c | *-* | CONSERVED HYPOTHETICAL ALANINE, ARGININE-RICH PROTEIN | 5, 10, 14, 50, 168, 233, 249, 284, 292, 335, 356, 366, 370, 415, 428, 440, 441, 448, 468, 478, 483, 484, 507 |
| Rv2734 | *-* | hypothetical protein Rv2734 | 17, 55, 158, 186, 211, 222 |
| Rv2735c | *-* | hypothetical protein Rv2735c | 16, 91, 100, 142, 251, 320 |
| Rv2736c | *recX* | recombination regulator RecX | 25, 35, 41, 56, 108, 165 |
| Rv2737c | *recA* | recombinase A | 42, 54, 86, 121, 179, 211, 263, 312, 333, 334, 422, 435, 480, 559, 561, 598, 601, 605, 613, 617, 784 |
| Rv2737A | *-* | CONSERVED HYPOTHETICAL CYSTEINE RICH PROTEIN (FRAGMENT) | 18, 22, 24, 46, 47, 51 |
| Rv2738c | *-* | hypothetical protein Rv2738c | 32, 39 |
| Rv2739c | *-* | POSSIBLE ALANINE RICH TRANSFERASE | 6, 26, 34, 41, 44, 64, 99, 131, 137, 140, 158, 164, 177, 188, 219, 220, 222, 232, 235, 237, 238, 266, 278, 295, 308, 316, 318, 341, 361, 366, 370 |
| Rv2740 | *-* | hypothetical protein Rv2740 | 37, 54, 84, 106 |
| Rv2741 | *PE_PGRS47* | PE-PGRS FAMILY PROTEIN | 29, 40, 80, 88, 91, 127, 131, 133, 134, 140, 149, 151, 155, 161, 167, 169, 170, 172, 178, 181, 183, 184, 187, 189, 196, 199, 203, 211, 215, 216, 218, 219, 221, 228, 230, 231, 234, 236, 239, 244, 245, 249, 252, 260, 261, 263, 264, 266, 267, 272, 275, 276, 279, 281, 282, 288, 292, 300, 303, 304, 307, 309, 316, 318, 319, 328, 329, 332, 335, 337, 338, 345, 347, 348, 351, 363, 366, 372, 375, 376, 378, 379, 382, 385, 388, 391, 394, 400, 402, 403, 405, 408, 415, 418, 419, 422, 428, 429, 431, 432, 434, 435, 443, 446, 449, 452, 458, 461, 464, 474, 478, 487, 490, 493, 498, 508, 509 |
| Rv2742c | *-* | CONSERVED HYPOTHETICAL ARGININE RICH PROTEIN | 72, 122, 125, 147, 167, 182, 185, 191, 202, 217, 221, 235, 274 |
| Rv2743c | *-* | POSSIBLE CONSERVED TRANSMEMBRANE ALANINE RICH PROTEIN | 16, 20, 21, 57, 83, 114, 126, 129, 139, 164, 176, 205, 210, 220, 237, 247, 264 |
| Rv2744c | *35kd_ag* | CONSERVED 35 KDA ALANINE RICH PROTEIN | 86, 88, 100, 110, 128, 177, 220, 225, 238, 244, 245, 259 |
| Rv2745c | *-* | POSSIBLE TRANSCRIPTIONAL REGULATORY PROTEIN | 13, 42, 90 |
| Rv2746c | *pgsA3* | PROBABLE PGP SYNTHASE PGSA3 (CDP-DIACYLGLYCEROL--GLYCEROL-3-PHOSPHATE 3-PHOSPHATIDYLTRANSFERASE) (PHOSPHATIDYLGLYCEROPHOSPHATE SYNTHASE) | 50, 55, 67, 139, 143, 147, 179, 206 |
| Rv2747 | *-* | N-acetylglutamate synthase | 20, 30, 64, 87, 135 |
| Rv2748c | *ftsK* | POSSIBLE CELL DIVISION TRANSMEMBRANE PROTEIN FTSK | 60, 108, 112, 127, 128, 142, 161, 168, 195, 246, 255, 263, 267, 283, 368, 369, 373, 376, 403, 418, 439, 452, 460, 482, 485, 506, 543, 602, 604, 637, 641, 669, 687, 688, 705, 772, 792, 848, 874, 880 |
| Rv2749 | *-* | hypothetical protein Rv2749 | 9, 33, 43, 63, 66, 73, 75, 92, 93 |
| Rv2750 | *-* | 3-ketoacyl-(acyl-carrier-protein) reductase | 13, 15, 17, 52, 66, 67, 104, 111, 125, 143, 153, 164, 171, 200, 257 |
| Rv2751 | *-* | hypothetical protein Rv2751 | 4, 8, 46, 65, 67, 99, 157, 196, 244, 258, 271 |
| Rv2752c | *-* | hypothetical protein Rv2752c | 12, 13, 18, 21, 44, 54, 159, 167, 193, 208, 289, 304, 341, 372, 377, 409, 412, 432, 438, 453, 482, 505, 519 |
| Rv2753c | *dapA* | dihydrodipicolinate synthase | 16, 23, 30, 32, 54, 84, 86, 91, 129, 178, 186, 192, 218, 226, 235, 279, 289 |
| Rv2754c | *thyX* | FAD-dependent thymidylate synthase | 3, 30, 32, 38, 53, 57, 82, 119, 134, 137, 169, 185, 219, 223 |
| Rv2755c | *hsdS.1* | POSSIBLE TYPE I RESTRICTION/MODIFICATION SYSTEM SPECIFICITY DETERMINANT (FRAGMENT) HSDS.1 (S PROTEIN) | 40, 51, 58, 64 |
| Rv2756c | *hsdM* | POSSIBLE TYPE I RESTRICTION/MODIFICATION SYSTEM DNA METHYLASE HSDM (M PROTEIN) (DNA METHYLTRANSFERASE) | 24, 29, 228, 230, 327, 392, 416, 454, 497 |
| Rv2757c | *-* | hypothetical protein Rv2757c | 29, 35, 121 |
| Rv2758c | *-* | hypothetical protein Rv2758c | 2, 28, 43, 47, 53, 66 |
| Rv2759c | *-* | hypothetical protein Rv2759c | 12, 33, 78, 87, 89, 109, 127 |
| Rv2760c | *-* | hypothetical protein Rv2760c | 17, 20, 52, 84 |
| Rv2761c | *hsdS* | POSSIBLE TYPE I RESTRICTION/MODIFICATION SYSTEM SPECIFICITY DETERMINANT HSDS (S PROTEIN) | 32, 33, 48, 67, 111, 132, 151, 159, 185, 207, 318, 322, 338, 354, 360 |
| Rv2762c | *-* | hypothetical protein Rv2762c | 2, 21, 24, 28, 102 |
| Rv2763c | *dfrA* | DIHYDROFOLATE REDUCTASE DFRA (DHFR) (TETRAHYDROFOLATE DEHYDROGENASE) | 15, 57, 73, 95, 122, 146 |
| Rv2764c | *thyA* | thymidylate synthase | 21, 23, 39, 63, 104, 188, 191, 201 |
| Rv2765 | *-* | PROBABLE ALANINE RICH HYDROLASE | 6, 28, 36, 37, 47, 51, 76, 124, 129, 137, 145, 152, 153, 177, 186, 200, 211, 225 |
| Rv2766c | *fabG* | 3-ketoacyl-(acyl-carrier-protein) reductase | 6, 9, 30, 37, 67, 89, 133, 139, 143, 159, 183, 228, 233, 241, 253 |
| Rv2767c | *-* | POSSIBLE MEMBRANE PROTEIN | 17, 106 |
| Rv2768c | *PPE43* | PPE FAMILY PROTEIN | 16, 18, 25, 26, 78, 94, 172, 176, 179, 182, 188, 190, 227, 253, 280, 282, 289, 326, 330, 353, 357, 372, 376 |
| Rv2769c | *PE27* | PE FAMILY PROTEIN | 14, 30, 32, 41, 43, 56, 92, 96, 98, 113, 136, 146, 156, 184, 192, 193, 230, 236, 242 |
| Rv2770c | *PPE44* | PPE FAMILY PROTEIN | 16, 59, 78, 94, 155, 157, 160, 176, 182, 188, 191, 219, 223, 242, 256, 272, 274, 278, 282, 294, 299, 326, 356, 364 |
| Rv2771c | *-* | hypothetical protein Rv2771c | 23, 39, 111 |
| Rv2772c | *-* | PROBABLE CONSERVED TRANSMEMBRANE PROTEIN | 38, 41, 64 |
| Rv2773c | *dapB* | dihydrodipicolinate reductase | 21, 28, 33, 79, 118, 143, 146, 187, 218 |
| Rv2774c | *-* | hypothetical protein Rv2774c | 2, 12, 34, 48, 61, 118, 123 |
| Rv2775 | *-* | hypothetical protein Rv2775 | 10, 38, 49, 85, 113, 148, 151 |
| Rv2776c | *-* | PROBABLE OXIDOREDUCTASE | 5, 26, 28, 36, 55, 58, 74, 76, 114, 127, 131, 140, 142, 158, 180, 191, 194, 267, 276, 282, 301 |
| Rv2777c | *-* | hypothetical protein Rv2777c | 68, 112, 152, 209, 258, 280, 306, 313, 336, 349 |
| Rv2778c | *-* | hypothetical protein Rv2778c | 77, 87, 128, 154 |
| Rv2779c | *-* | POSSIBLE TRANSCRIPTIONAL REGULATORY PROTEIN (PROBABLY LRP/ASNC-FAMILY) | 28, 104, 106, 129, 139, 173 |
| Rv2780 | *ald* | SECRETED L-ALANINE DEHYDROGENASE ALD (40 KDA ANTIGEN) (TB43) | 20, 38, 59, 97, 109, 121, 136, 140, 155, 160, 163, 175, 177, 178, 211, 271, 301, 365 |
| Rv2781c | *-* | POSSIBLE ALANINE RICH OXIDOREDUCTASE | 17, 18, 26, 33, 39, 53, 57, 60, 156, 161, 174, 175, 178, 187, 210, 220, 225, 229, 234, 242, 246, 250, 272, 328 |
| Rv2782c | *pepR* | PROBABLE ZINC PROTEASE PEPR | 8, 21, 31, 35, 54, 84, 91, 124, 200, 201, 234, 262, 264, 277, 280, 295, 303, 311, 332, 349 |
| Rv2783c | *gpsI* | polynucleotide phosphorylase/polyadenylase | 2, 13, 30, 37, 38, 56, 81, 85, 152, 157, 161, 191, 229, 240, 244, 252, 286, 292, 325, 349, 375, 417, 420, 483, 492, 538, 545, 562, 624, 648, 740, 742, 746 |
| Rv2784c | *lppU* | PROBABLE LIPOPROTEIN LPPU | 6, 36, 49, 99, 146, 165, 166 |
| Rv2785c | *rpsO* | 30S ribosomal protein S15 | 21 |
| Rv2786c | *ribF* | bifunctional riboflavin kinase/FMN adenylyltransferase | 35, 47, 51, 138, 139, 184, 206, 218, 236, 248, 259, 270, 284, 301 |
| Rv2787 | *-* | CONSERVED HYPOTHETICAL ALANINE RICH PROTEIN | 12, 19, 49, 58, 82, 131, 133, 164, 178, 183, 228, 238, 247, 277, 328, 347, 370, 425, 441, 469, 509, 555, 556 |
| Rv2788 | *sirR* | PROBABLE TRANSCRIPTIONAL REPRESSOR SIRR | 22, 77, 198, 219 |
| Rv2789c | *fadE21* | PROBABLE ACYL-CoA DEHYDROGENASE FADE21 | 31, 94, 118, 147, 153, 195, 211, 232, 331, 350, 363, 387 |
| Rv2790c | *ltp1* | lipid-transfer protein | 22, 45, 90, 103, 120, 124, 159, 170, 241, 284, 314, 331, 339, 365, 367, 377, 387, 389, 393 |
| Rv2791c | *-* | PROBABLE TRANSPOSASE | 21, 56, 85, 144, 215, 233, 332, 373, 419 |
| Rv2792c | *-* | POSSIBLE RESOLVASE | 32, 74, 135, 184 |
| Rv2793c | *truB* | tRNA pseudouridine synthase B | 3, 5, 13, 45, 70, 104, 110, 156, 221, 241, 248, 296 |
| Rv2794c | *-* | hypothetical protein Rv2794c | 28, 94, 97, 184, 217, 222 |
| Rv2795c | *-* | hypothetical protein Rv2795c | 24, 48, 123, 224, 229, 290, 297, 310 |
| Rv2796c | *lppV* | PROBABLE CONSERVED LIPOPROTEIN LPPV | 18, 27, 74, 86, 88, 132, 149, 152, 167 |
| Rv2797c | *-* | hypothetical protein Rv2797c | 37, 49, 81, 93, 98, 101, 117, 171, 219, 245, 292, 301, 312, 332, 335, 355, 429, 477, 491, 511, 512, 520, 528, 529, 547, 553 |
| Rv2798c | *-* | hypothetical protein Rv2798c | 35, 76, 97 |
| Rv2799 | *-* | PROBABLE MEMBRANE PROTEIN | 47, 48, 57, 140, 144 |
| Rv2800 | *-* | POSSIBLE HYDROLASE | 4, 16, 25, 59, 89, 91, 101, 106, 117, 134, 161, 179, 200, 205, 211, 220, 260, 274, 317, 346, 369, 380, 391, 398, 407, 425, 506, 525 |
| Rv2801c | *-* | hypothetical protein Rv2801c | 3, 13, 24, 36, 42, 99 |
| Rv2802c | *-* | HYPOTHETICAL ARGININE AND ALANINE RICH PROTEIN | 63, 67, 100, 147, 149, 176, 179, 255, 266, 279, 281 |
| Rv2803 | *-* | hypothetical protein Rv2803 | 12, 87, 91, 101, 122 |
| Rv2804c | *-* | hypothetical protein Rv2804c | 7, 47, 130, 149, 165, 192 |
| Rv2805 | *-* | hypothetical protein Rv2805 | 27, 67, 83, 85, 116 |
| Rv2806 | *-* | POSSIBLE MEMBRANE PROTEIN | 38, 54 |
| Rv2807 | *-* | hypothetical protein Rv2807 | 4, 16, 56, 72, 74, 104, 151, 170, 208, 218, 261, 328, 363 |
| Rv2808 | *-* | hypothetical protein Rv2808 | 24 |
| Rv2809 | *-* | hypothetical protein Rv2809 | 41, 49 |
| Rv2810c | *-* | PROBABLE TRANSPOSASE | 8, 46, 53, 55, 122 |
| Rv2811 | *-* | hypothetical protein Rv2811 | 16, 24, 40, 56, 58, 144, 175, 190 |
| Rv2812 | *-* | PROBABLE TRANSPOSASE | 28, 74, 75, 141, 145, 165, 193, 207, 217, 277, 314, 324, 325, 331, 351, 389, 415, 421, 431, 439, 444, 447, 454, 461 |
| Rv2813 | *-* | hypothetical protein Rv2813 | 50, 55, 63, 97, 111, 116, 122, 178, 198, 209, 245, 248, 261 |
| Rv2814c | *-* | PROBABLE TRANSPOSASE | 68, 105, 109, 125, 259, 273, 289, 309, 310 |
| Rv2815c | *-* | PROBABLE TRANSPOSASE | 24, 60, 64, 71, 93 |
| Rv2816c | *-* | hypothetical protein Rv2816c | 19, 47, 56 |
| Rv2817c | *-* | hypothetical protein Rv2817c | 157, 163, 289, 319, 335 |
| Rv2818c | *-* | hypothetical protein Rv2818c | 18, 72, 93, 141, 183, 203, 209, 325, 342 |
| Rv2819c | *-* | hypothetical protein Rv2819c | 21, 109, 258, 260, 277, 306, 309 |
| Rv2820c | *-* | hypothetical protein Rv2820c | 27, 98, 161, 178, 181, 186, 199, 226, 229, 261, 262, 280, 299 |
| Rv2821c | *-* | hypothetical protein Rv2821c | 10, 23, 28, 114, 132, 145, 162, 185, 196, 198, 215 |
| Rv2822c | *-* | hypothetical protein Rv2822c | 15, 38, 72, 112 |
| Rv2823c | *-* | hypothetical protein Rv2823c | 91, 104, 137, 171, 240, 284, 320, 321, 334, 391, 402, 456, 516, 522, 543, 581, 608, 623, 689, 730, 765, 793 |
| Rv2824c | *-* | hypothetical protein Rv2824c | 5, 149, 189, 194, 238, 255, 258, 266, 287 |
| Rv2825c | *-* | hypothetical protein Rv2825c | 3, 30, 60, 100, 112, 118, 138, 178, 200, 206 |
| Rv2826c | *-* | hypothetical protein Rv2826c | 1, 21, 50, 92, 216, 228, 274 |
| Rv2827c | *-* | hypothetical protein Rv2827c | 4, 19, 49, 74, 77, 101, 104, 150, 157, 172, 237, 262 |
| Rv2828c | *-* | hypothetical protein Rv2828c | 26, 66, 78, 84, 104, 144, 166, 172 |
| Rv2829c | *-* | hypothetical protein Rv2829c | 37, 80, 91, 102 |
| Rv2830c | *-* | hypothetical protein Rv2830c | 1, 54, 63 |
| Rv2831 | *echA16* | enoyl-CoA hydratase | 54, 62, 70, 73, 96, 99, 100, 153, 158, 165, 180, 211, 213, 228, 233, 242 |
| Rv2832c | *ugpC* | PROBABLE Sn-GLYCEROL-3-PHOSPHATE TRANSPORT ATP-BINDING PROTEIN ABC TRANSPORTER UGPC | 13, 39, 41, 51, 58, 94, 104, 132, 136, 137, 178, 245, 330 |
| Rv2833c | *ugpB* | PROBABLE Sn-GLYCEROL-3-PHOSPHATE-BINDING LIPOPROTEIN UGPB | 12, 14, 16, 20, 23, 26, 28, 33, 35, 73, 110, 166, 171, 192, 217, 231, 236, 262, 272, 276, 279, 288, 299, 304, 335, 342, 350, 388, 395, 396, 404 |
| Rv2834c | *ugpE* | PROBABLE Sn-GLYCEROL-3-PHOSPHATE TRANSPORT INTEGRAL MEMBRANE PROTEIN ABC TRANSPORTER UGPE | 22, 111, 138, 162 |
| Rv2835c | *ugpA* | PROBABLE Sn-GLYCEROL-3-PHOSPHATE TRANSPORT INTEGRAL MEMBRANE PROTEIN ABC TRANSPORTER UGPA | 86, 88, 125, 127, 132, 187, 247, 252, 271 |
| Rv2836c | *dinF* | POSSIBLE DNA-DAMAGE-INDUCIBLE PROTEIN F DINF | 8, 18, 24, 79, 84, 110, 125, 147, 153, 167, 192, 210, 261, 293, 297, 300, 333, 344, 376, 429 |
| Rv2837c | *-* | hypothetical protein Rv2837c | 25, 31, 50, 61, 70, 121, 139, 175, 241, 300, 308, 314, 320 |
| Rv2838c | *rbfA* | ribosome-binding factor A | 31, 41, 65, 68, 86, 117, 170, 174 |
| Rv2839c | *infB* | translation initiation factor IF-2 | 62, 66, 80, 93, 95, 98, 101, 102, 112, 130, 134, 161, 174, 176, 179, 188, 195, 212, 216, 219, 227, 234, 238, 240, 245, 248, 250, 252, 256, 260, 262, 263, 275, 296, 312, 327, 388, 427, 428, 462, 480, 498, 521, 535, 561, 564, 589, 599, 610, 646, 648, 708, 734, 737, 824 |
| Rv2840c | *-* | hypothetical protein Rv2840c | 23, 68, 74 |
| Rv2841c | *nusA* | transcription elongation factor NusA | 27, 51, 82, 127, 147, 213, 226, 250, 285, 288, 316, 323, 331, 336, 340 |
| Rv2842c | *-* | hypothetical protein Rv2842c | 2, 21, 55, 85, 116, 176, 180 |
| Rv2843 | *-* | PROBABLE CONSERVED TRANSMEMBRANE ALANINE RICH PROTEIN | 22, 25, 28, 34, 36, 39, 44, 65, 77, 101, 107, 162, 166 |
| Rv2844 | *-* | CONSERVED HYPOTHETICAL ALANINE RICH PROTEIN | 5, 43, 73, 75, 78, 84, 153, 158 |
| Rv2845c | *proS* | prolyl-tRNA synthetase | 16, 30, 38, 101, 148, 168, 169, 200, 204, 217, 239, 258, 281, 308, 372, 377, 386, 391, 398, 411, 418, 437, 500, 503, 506, 525, 545, 559, 567, 580 |
| Rv2846c | *efpA* | POSSIBLE INTEGRAL MEMBRANE EFFLUX PROTEIN EFPA | 15, 32, 58, 87, 94, 101, 107, 160, 177, 186, 197, 225, 240, 250, 279, 315, 316, 374, 407, 445, 465, 468, 480, 494, 501, 526 |
| Rv2847c | *cysG* | POSSIBLE MULTIFUNCTIONAL ENZYME SIROHEME SYNTHASE CYSG: UROPORPHYRIN-III C-METHYLTRANSFERASE (UROGEN III METHYLASE) (SUMT) (UROPORPHYRINOGEN III METHYLASE) (UROM) + PRECORRIN-2 OXIDASE + FERROCHELATASE | 20, 48, 126, 158, 165, 168, 177, 235, 263, 280, 305, 359, 384, 396 |
| Rv2848c | *cobB* | cobyrinic acid a,c-diamide synthase | 3, 8, 14, 30, 46, 52, 56, 100, 104, 109, 127, 134, 145, 150, 173, 198, 224, 230, 248, 259, 276, 277, 301, 302, 309, 313, 326, 328, 337, 352, 356, 395, 405, 436, 439 |
| Rv2849c | *cobO* | cob(I)yrinic acid a,c-diamide adenosyltransferase | 28, 36, 84, 105, 113, 169, 180 |
| Rv2850c | *-* | POSSIBLE MAGNESIUM CHELATASE | 45, 48, 55, 60, 101, 123, 213, 253, 255, 258, 316, 318, 401, 420, 421, 428, 458, 511, 520, 521, 532, 560, 561, 571, 574 |
| Rv2851c | *-* | hypothetical protein Rv2851c | 71, 89, 147, 149 |
| Rv2852c | *mqo* | malate:quinone oxidoreductase | 43, 47, 53, 55, 58, 135, 141, 160, 174, 199, 226, 241, 252, 261, 266, 272, 278, 318, 391, 405, 423, 427, 434, 473, 491 |
| Rv2853 | *PE_PGRS48* | PE-PGRS FAMILY PROTEIN | 11, 40, 42, 82, 91, 107, 129, 130, 132, 133, 140, 144, 145, 151, 152, 154, 155, 158, 164, 167, 169, 176, 179, 189, 191, 192, 195, 204, 206, 222, 224, 225, 230, 236, 245, 265, 266, 268, 274, 281, 284, 286, 287, 297, 304, 305, 307, 314, 320, 327, 330, 345, 346, 348, 349, 352, 365, 369, 382, 385, 387, 391, 394, 397, 401, 404, 406, 407, 409, 413, 415, 418, 421, 422, 424, 425, 427, 428, 444, 448, 451, 460, 466, 476, 483, 485, 489, 492, 495, 497, 498, 501, 504, 506, 507, 509, 515, 516, 522, 523, 524, 530, 533, 536, 539, 541, 542, 545, 551, 553, 554, 564, 565, 569, 570, 572, 575, 578, 582, 584, 595, 603, 610, 613 |
| Rv2854 | *-* | hypothetical protein Rv2854 | 9, 35, 43, 87, 93, 132, 133, 134, 148, 157, 184, 201, 225, 246, 274, 288 |
| Rv2855 | *mtr* | mycothione/glutathione reductase | 10, 35, 52, 93, 133, 140, 141, 154, 179, 191, 240, 277, 400, 431, 437 |
| Rv2856 | *nicT* | POSSIBLE NICKEL-TRANSPORT INTEGRAL MEMBRANE PROTEIN NICT | 51, 57, 84, 124, 152, 179, 194, 227, 241, 243, 258, 259, 294, 328 |
| Rv2857c | *-* | short chain dehydrogenase | 8, 16, 19, 25, 45, 49, 89, 157, 239 |
| Rv2858c | *aldC* | PROBABLE ALDEHYDE DEHYDROGENASE ALDC | 8, 49, 66, 73, 84, 95, 102, 109, 110, 145, 146, 154, 205, 216, 218, 246, 249, 252, 294, 314, 333, 346, 378, 383, 403, 421, 426 |
| Rv2859c | *-* | POSSIBLE AMIDOTRANSFERASE | 78, 89, 99, 129, 177, 187, 201, 217, 230, 293, 295 |
| Rv2860c | *glnA4* | PROBABLE GLUTAMINE SYNTHETASE GLNA4 (GLUTAMINE SYNTHASE) (GS-II) | 1, 3, 19, 37, 53, 72, 81, 137, 162, 165, 197, 275, 296, 356, 357, 407 |
| Rv2861c | *mapB* | methionine aminopeptidase | 4, 8, 31, 69, 132, 148, 175, 222, 264, 276 |
| Rv2862c | *-* | hypothetical protein Rv2862c | 3, 70, 78, 81, 170, 175 |
| Rv2863 | *-* | hypothetical protein Rv2863 | 21, 91, 107 |
| Rv2864c | *-* | POSSIBLE PENICILLIN-BINDING LIPOPROTEIN | 31, 40, 47, 49, 73, 89, 156, 173, 176, 229, 312, 321, 333, 343, 354, 359, 367, 368, 386, 431, 455, 492, 493, 503, 536, 550, 557, 564, 579 |
| Rv2865 | *-* | hypothetical protein Rv2865 | 34, 57, 72 |
| Rv2866 | *-* | hypothetical protein Rv2866 | 9, 49, 56 |
| Rv2867c | *-* | hypothetical protein Rv2867c | 20, 38, 52, 59, 68, 76, 142, 158, 178, 185, 190, 228, 237, 247, 264 |
| Rv2868c | *ispG* | 4-hydroxy-3-methylbut-2-en-1-yl diphosphate synthase | 60, 62, 104, 111, 137, 147, 199, 219, 229, 282, 297, 298, 331, 365, 378 |
| Rv2869c | *-* | PROBABLE CONSERVED TRANSMEMBRANE PROTEIN | 4, 31, 51, 70, 101, 103, 153, 155, 161, 165, 184, 203, 223, 238, 250, 259, 271, 285, 304, 340, 364, 392 |
| Rv2870c | *dxr* | 1-deoxy-D-xylulose 5-phosphate reductoisomerase | 20, 44, 46, 60, 86, 114, 132, 143, 154, 161, 184, 281, 283, 291, 306, 314, 317, 322, 364 |
| Rv2871 | *-* | hypothetical protein Rv2871 | 18, 21, 26, 44, 45, 56, 60, 68 |
| Rv2872 | *-* | hypothetical protein Rv2872 | 44, 76, 111 |
| Rv2873 | *mpt83* | CELL SURFACE LIPOPROTEIN MPT83 (LIPOPROTEIN P23) | 8, 54, 72, 74, 78, 87, 118, 129, 159, 193, 198, 202 |
| Rv2874 | *dipZ* | POSSIBLE INTEGRAL MEMBRANE C-TYPE CYTOCHROME BIOGENESIS PROTEIN DIPZ | 17, 21, 38, 51, 123, 127, 143, 209, 219, 263, 273, 287, 304, 326, 343, 353, 362, 373, 390, 396, 406, 412, 508, 516, 545, 564, 581, 583, 607, 624, 642, 659, 669 |
| Rv2875 | *mpt70* | MAJOR SECRETED IMMUNOGENIC PROTEIN MPT70 | 14, 92, 133, 156, 176 |
| Rv2876 | *-* | POSSIBLE CONSERVED TRANSMEMBRANE PROTEIN | 11, 25, 33, 83, 99 |
| Rv2877c | *-* | PROBABLE CONSERVED INTEGRAL MEMBRANE PROTEIN | 10, 11, 20, 53, 75, 99, 152, 158, 186, 188, 231, 243, 245, 277 |
| Rv2878c | *mpt53* | SOLUBLE SECRETED ANTIGEN MPT53 PRECURSOR | 44, 49, 87, 155, 164 |
| Rv2879c | *-* | hypothetical protein Rv2879c | 178, 180, 184, 187 |
| Rv2880c | *-* | hypothetical protein Rv2880c | 70, 79, 81, 88, 123, 125, 137, 154, 156, 178, 196, 206, 247, 260 |
| Rv2881c | *cdsA* | PROBABLE INTEGRAL MEMBRANE PHOSPHATIDATE CYTIDYLYLTRANSFERASE CDSA (CDP-DIGLYCERIDE SYNTHETASE) (CDP-DIGLYCERIDE PYROPHOSPHORYLASE) (CDP-DIACYLGLYCEROL SYNTHASE) (CDS) (CTP:PHOSPHATIDATE CYTIDYLYLTRANSFERASE) (CDP-DAG SYNTHASE) (CDP-DG SYNTHETASE) | 5, 7, 10, 29, 92, 95, 135, 136, 191, 218, 223, 226, 231, 254, 278, 281, 293 |
| Rv2882c | *frr* | ribosome recycling factor | 28, 147 |
| Rv2883c | *pyrH* | uridylate kinase | 6, 20, 27, 37, 42, 65, 75, 125, 155, 167, 204, 214, 222 |
| Rv2884 | *-* | PROBABLE TRANSCRIPTIONAL REGULATORY PROTEIN | 73, 116, 117, 153, 178, 226 |
| Rv2885c | *-* | PROBABLE TRANSPOSASE | 86, 118, 145, 254, 401, 420, 430, 444, 448 |
| Rv2886c | *-* | PROBABLE RESOLVASE | 25, 26, 30, 76, 128, 142, 174, 283, 288 |
| Rv2887 | *-* | PROBABLE TRANSCRIPTIONAL REGULATORY PROTEIN | 24, 75, 81, 86, 95, 111, 121 |
| Rv2888c | *amiC* | amidase | 36, 87, 116, 166, 177, 185, 197, 321, 346, 429, 443 |
| Rv2889c | *tsf* | elongation factor Ts | 5, 15, 17, 100, 151, 165, 170, 255 |
| Rv2890c | *rpsB* | 30S ribosomal protein S2 | 11, 80, 122, 148, 209, 218, 228, 241, 265, 275 |
| Rv2891 | *-* | hypothetical protein Rv2891 | 4, 56, 66, 71, 74, 89, 134, 137, 141, 196, 198, 202, 216, 218, 221, 240 |
| Rv2892c | *PPE45* | PPE FAMILY PROTEIN | 11, 25, 41, 42, 52, 59, 66, 78, 79, 92, 148, 156, 167, 176, 179, 196, 267, 269, 286, 296, 301, 320, 325, 343, 362, 365, 367, 381, 385, 386 |
| Rv2893 | *-* | POSSIBLE OXIDOREDUCTASE | 5, 36, 37, 59, 70, 85, 90, 106, 115, 116, 122, 175, 185, 195, 227, 253, 257, 261, 287 |
| Rv2894c | *xerC* | site-specific tyrosine recombinase XerC | 15, 39, 56, 63, 79, 85, 89, 145, 153, 162, 185, 202, 205, 232 |
| Rv2895c | *viuB* | POSSIBLE MYCOBACTIN UTILIZATION PROTEIN VIUB | 26, 28, 71, 87, 105, 123, 124, 132, 146, 149, 168, 177, 189, 201, 214, 244, 255, 260, 279 |
| Rv2896c | *-* | hypothetical protein Rv2896c | 23, 34, 40, 51, 70, 88, 93, 100, 112, 123, 143, 144, 155, 181, 182, 211, 228, 243, 245, 248, 263, 302, 308, 329, 331, 341, 345, 358, 373, 375 |
| Rv2897c | *-* | hypothetical protein Rv2897c | 55, 123, 168, 171, 174, 206, 208, 246, 281, 297, 345, 353, 392, 397, 417, 446, 453, 457, 463, 479, 490, 498 |
| Rv2898c | *-* | hypothetical protein Rv2898c | 63, 86 |
| Rv2899c | *fdhD* | formate dehydrogenase accessory protein | 98, 117, 137, 140, 144, 165, 216, 236, 239, 252 |
| Rv2900c | *fdhF* | POSSIBLE FORMATE DEHYDROGENASE H FDHF (FORMATE-HYDROGEN-LYASE-LINKED, SELENOCYSTEINE-CONTAINING POLYPEPTIDE) (FORMATE DEHYDROGENASE-H ALPHA SUBUNIT) (FDH-H) | 56, 80, 81, 119, 140, 152, 238, 242, 272, 303, 310, 324, 338, 359, 370, 413, 421, 464, 481, 491, 521, 583, 604, 608, 656, 682, 686, 720, 758 |
| Rv2901c | *-* | hypothetical protein Rv2901c |  |
| Rv2902c | *rnhB* | ribonuclease HII | 24, 35, 42, 58, 65, 105, 120, 126, 153, 157, 179, 223 |
| Rv2903c | *lepB* | PROBABLE SIGNAL PEPTIDASE I LEPB (SPASE I) (LEADER PEPTIDASE I). | 179, 189, 229 |
| Rv2904c | *rplS* | 50S ribosomal protein L19 | 50, 102 |
| Rv2905 | *lppW* | PROBABLE CONSERVED ALANINE RICH LIPOPROTEIN LPPW | 9, 85, 115, 166, 183, 197, 212, 213, 220, 233, 272, 290, 295 |
| Rv2906c | *trmD* | tRNA (guanine-N(1)-)-methyltransferase | 9, 53, 84, 85, 93, 121, 138, 139, 156, 162, 200, 207 |
| Rv2907c | *rimM* | 16S rRNA-processing protein | 14, 62, 82, 129 |
| Rv2908c | *-* | hypothetical protein Rv2908c | 54, 56, 63, 68 |
| Rv2909c | *rpsP* | 30S ribosomal protein S16 | 76, 95, 107, 110 |
| Rv2910c | *-* | hypothetical protein Rv2910c | 93, 107 |
[truncated: 153,710 more chars]
