# Supplementary material for: The EXIT Strategy: an Approach for Identifying Bacterial Proteins Exported during Host Infection
Source: mBio. 2017 Apr 25;8(2):e00333-17. doi: 10.1128/mBio.00333-17 (PMC5405230; doi:10.1128/mBio.00333-17)
Supplement: TABLE S5 [file mbo002173284st5.docx]

| **Supplemental Table 5. EXIT exported fusions in unannotated regions** | | |
| --- | --- | --- |
| **Strand location** | **Upstream annotated gene** | **Genome position** |
| - | Rv0066c | 65092 |
| - | Rv0066c | 65050 |
| + | Rv0397 | 476536 |
| + | Rv0397 | 476632 |
| - | Rv2307A | 2578951 |
| - | Rv2307A | 2578942 |
| - | Rv2307A | 2578876 |
| - | Rv2307c | 2575499 |
| - | Rv2307c | 2575451 |
| - | Rv2307c | 2575403 |
| - | Rv2307c | 2575385 |
| - | Rv2307c | 2575379 |
| - | Rv2307c | 2575358 |
| - | Rv2307c | 2575340 |
| + | Rv2964 | 3318177 |
| + | Rv2964 | 3318183 |
| + | Rv2964 | 3318189 |
| + | Rv2964 | 3318198 |
| + | Rv3033 | 3395236 |
| + | Rv3033 | 3395269 |
| + | Rv3033 | 3395302 |

**Supplemental Table 5. EXIT exported fusions in unannotated regions.** Enriched sites data located in intergenic non-annotated regions of the genome were identified. Regions of the genome likely to contain a non-annotated exported protein were identified as containing at least two statistically enriched sites in frame of each other at a maximum distance of 100 bp apart. Six intergenic regions were identified containing 21 statistically enriched sites. The genome location, strand, and upstream annotated gene are identified.
