# Supplementary material for: The EXIT Strategy: an Approach for Identifying Bacterial Proteins Exported during Host Infection
Source: mBio. 2017 Apr 25;8(2):e00333-17. doi: 10.1128/mBio.00333-17 (PMC5405230; doi:10.1128/mBio.00333-17)
Supplement: TABLE S6 [file mbo002173284st6.docx]

| **Supplemental Table 6. Reagents used in this study** | | | | |
| --- | --- | --- | --- | --- |
| **A. Plasmids** | | | | |
| **Plasmid** | **Antibiotic resistance** | | **Notes** | **Source** |
| pMV261 | kan | | Multi-copy mycobacterial vector with *hsp60* promoter | ([1](#_ENREF_1)) |
| pJSC77 | kan | | Multi-copy mycobacterial vector, HA tag cloned into pMV261 | ([2](#_ENREF_2)) |
| pJES102 | kan | | *'blaTEM-1* reporter in pMV261 | ([3](#_ENREF_3)) |
| pMB227 | kan | | *Mtb mpt63* signal peptide in pMV261 | ([4](#_ENREF_4)) |
| pJES103 | kan | | *'blaTEM-1* in pMB227 (*mpt63* sp-*'blaTEM-1*) | ([3](#_ENREF_3)) |
| pEP207 | kan | | P_hsp60_-*rv3707c* cloned into pJSC77 | This study |
| pEP210 | kan | | P_hsp60_-*rv1728* cloned into pJSC77 | This study |
| pEP213 | kan | | P_hsp60_-*rv3811* cloned into pJSC77 | This study |
| pMB219 | kan | | Multi-copy mycobacterial vector | ([3](#_ENREF_3)) |
| pYUB412 | hyg | | Single copy integrating mycobacterial vector | ([5](#_ENREF_5)) |
| pJES110 | kan, amp | | *'blaTEM* reporter in cloning vector pCR2.1 Topo (Invitrogen) | This study |
| pDW31 | hyg | | Multi-copy mycobacterial vector, with *'blaTEM* reporter downstream of ClaI restriction site. Plasmid backbone derived from pMB219. Hygromycin cassette from pYUB412. | This study |
| pMV306 | kan | | Single-copy mycobacterial vector, with *attP* integration site | ([1](#_ENREF_1)) |
| **B. Bacterial Strains** | | | | |
| ***M. tuberculosis* strains** | | **Description** | | **Source** |
| PM638 | | *M. tuberculosis* H37Rv *ΔblaC* | | ([6](#_ENREF_6)) |
| MBTB58 | | PM638 + pJES102 (*'blaTEM-1*) | | ([3](#_ENREF_3)) |
| MBTB59 | | PM638 + pJES103 (sp-*'blaTEM-1*) | | ([3](#_ENREF_3)) |
| EXIT Library | | PM638 *+* pDW31 containing PM638 genomic DNA fragments (~500-5,000 bp) in ClaI site. Strain is hyg^R^ | | This study |
| MBTB542 | | H37Rv + pEP207 (expressing *rv3707c-HA*). Strain is kan^R^ | | This study |
| MBTB543 | | H37Rv + pEP210 (expressing *rv1728-HA*). Strain is kan^R^ | | This study |
| MBTB544 | | H37Rv + pEP213 (expressing *rv3811-HA*). Strain is kan^R^ | | This study |
| NR-13649 | | *Mycobacterium tuberculosis* CDC1551 parental strain | | ([7](#_ENREF_7)) |
| MBTB578 | | *Mycobacterium tuberculosis* CDC1551 parental strain (NR-13649) + pMV306. Strain is kan^R^ | | This study |
| NR-18234 | | *Mycobacterium tuberculosis* CDC1551 Transposon Mutant 1815 (MT3810, Rv3707c). Strain is kan^R^ | | ([7](#_ENREF_7)) |
| NR-14987 | | *Mycobacterium tuberculosis* CDC1551 Transposon Mutant 431 (MT1556, Rv1508c). Strain is kan^R^ | | ([7](#_ENREF_7)) |
| NR-18775 | | *Mycobacterium tuberculosis* CDC1551 Transposon Mutant 297 (MT2611, Rv2536). Strain is kan^R^ | | ([7](#_ENREF_7)) |
| NR-18600 | | *Mycobacterium tuberculosis* CDC1551 Transposon Mutant 2659 (MT0585, Rv0559c). Strain is kan^R^ | | ([7](#_ENREF_7)) |
| NR-18478 | | *Mycobacterium tuberculosis* CDC1551 Transposon Mutant 2361 (MT2342, Rv2284). Strain is kan^R^ | | ([7](#_ENREF_7)) |
| NR-18554 | | *Mycobacterium tuberculosis* CDC1551 Transposon Mutant 2539 (MT0274, Rv0261c). Strain is kan^R^ | | ([7](#_ENREF_7)) |
| **C. Primers** | | | | |
| **Name** | **Primer sequence** | | | **Source** |
| Adaptor 1.1 | TACCACGACCA-NH2 | | | ([8](#_ENREF_8)) |
| Adaptor 2.1 | ATGATGGCCGGTGGATTTGTGNNANNANNNTGGTCGTGGTAT | | | ([8](#_ENREF_8)) |
| 'Bla IL | AATGATACGGCGACCACCGAGATCTACACTCTTTCCCTACACGACGCTCTTCCGATCTTTTACTTTCACCAGCGTTTC | | | This study |
| 'Bla IL 3b | AATGATACGGCGACCACCGAGATCTACACTCTTTCCCTACACGACGCTCTTCCGATCTTTTTACTTTCACCAGCGTTTC | | | This study |
| 'Bla IL 4b | AATGATACGGCGACCACCGAGATCTACACTCTTTCCCTACACGACGCTCTTCCGATCTGATATTTACTTTCACCAGCGTTTC | | | This study |
| 'Bla IL 5b | AATGATACGGCGACCACCGAGATCTACACTCTTTCCCTACACGACGCTCTTCCGATCTATCTATTTACTTTCACCAGCGTTTC | | | This study |
| Adaptor 1 | CAAGCAGAAGACGGCATACGAGATAAGTAGAGGTGACTGGAGTTCAGACGTGTGCTCTTCCGATCTATGATGGCCGGTGGATTTGTG | | | ([8](#_ENREF_8)) |
| Adaptor 2 | CAAGCAGAAGACGGCATACGAGATACACGATCGTGACTGGAGTTCAGACGTGTGCTCTTCCGATCTATGATGGCCGGTGGATTTGTG | | | ([8](#_ENREF_8)) |
| Adaptor 3 | CAAGCAGAAGACGGCATACGAGATCGCGCGGTGTGACTGGAGTTCAGACGTGTGCTCTTCCGATCTATGATGGCCGGTGGATTTGTG | | | ([8](#_ENREF_8)) |
| *rv3707c*_HA_F2 | GAATGCCTTCCTGCGAATCGGTCCGACGGCCGGTAC | | | This study |
| *rv3707c*_HA_R1 | AAGCTTGCGGGTCTGACCAGGGCTTGAAC | | | This study |
| *rv1728c*_HA_F1 | CGAATGCCTTCATGAGCGTGAACGGCTTGC | | | This study |
| *rv1728c*_HA_R1 | AAGCTTGTTCTGGCGGCGTAGGGCTC | | | This study |
| *rv3811*_HA_F1 | GAATGCCTTCGCAGCGACCGTCGTCATCGTCGCGTGGATAG | | | This study |
| *rv3811*_HA_R1 | AAGCTTGGGTGATCGGATGCGTTGGCAGCGTGAAG | | | This study |

**Supplemental Table 6. Reagents used in this study. A.** Plasmids. **B.** Bacterial strains. **C.** Primers used to prepare fragments for next-generation sequencing are color coded for additional information. The adaptor ligation sites represent where the double stranded adaptor primers attach for Adaptor 1.1 and Adaptor 2.1 (pink), or where the PCR amplification primer has homology to the adaptor for PCR amplification (yellow, Adaptor 2.1 and Adaptor primers). Two barcodes were used, a random barcode integrated into the Adaptor 2.1 sequence (bright green) to be able to quantify PCR biases in amplification, and an index or multiplexing barcode to allow for multiple sequences to be simultaneously sequenced in Adaptor 1, 2, and 3 primers (purple). PCR amplification using ‘Bla IL and Adaptor 1,2,3 primers was used to attach the sequences necessary for attachment to the Illumina chip (blue) and for sequencing with the Illumina primers (red, Read 1 sequence in Bla IL primers Read 2 sequence in Adaptor primers). The ‘Bla IL primers additionally possessed homology to the ‘BlaTEM reporter for PCR amplification (dark green), and a variable site for sequencing (grey).

**References**

1. Stover CK, de la Cruz VF, Fuerst TR, Burlein JE, Benson LA, Bennett LT, Bansal GP, Young JF, Lee MH, Hatfull GF. 1991. New use of BCG for recombinant vaccines. Nature 351:456-60.

2. Glickman MS, Cox JS, Jacobs WR, Jr. 2000. A novel mycolic acid cyclopropane synthetase is required for cording, persistence, and virulence of *Mycobacterium tuberculosis*. Mol Cell 5:717-27.

3. McCann JR, McDonough JA, Pavelka MS, Braunstein M. 2007. Beta-lactamase can function as a reporter of bacterial protein export during *Mycobacterium tuberculosis* infection of host cells. Microbiology 153:3350-9.

4. McDonough JA, Hacker KE, Flores AR, Pavelka MS, Jr., Braunstein M. 2005. The twin-arginine translocation pathway of *Mycobacterium smegmatis* is functional and required for the export of mycobacterial beta-lactamases. J Bacteriol 187:7667-79.

5. Bange FC, Collins FM, Jacobs WRJ. 1999. Survival of mice infected with *Mycobacterium smegmatis* containing large DNA fragments from *Mycobacterium tuberculosis*. Tubercle and Lung Disease 79:171-180.

6. Flores AR, Parsons LM, Pavelka MS, Jr. 2005. Genetic analysis of the beta-lactamases of *Mycobacterium tuberculosis* and *Mycobacterium smegmatis* and susceptibility to beta-lactam antibiotics. Microbiology 151:521-32.

7. Lamichhane G, Zignol M, Blades NJ, Geiman DE, Dougherty A, Grosset J, Broman KW, Bishai WR. 2003. A postgenomic method for predicting essential genes at subsaturation levels of mutagenesis: application to *Mycobacterium tuberculosis*. Proc Natl Acad Sci U S A 100:7213-8.

8. Long JE, DeJesus M, Ward D, Baker RE, Ioerger T, Sassetti CM. 2015. Identifying essential genes in *Mycobacterium tuberculosis* by global phenotypic profiling. Methods Mol Biol 1279:79-95.
