# Supplementary material for: The EXIT Strategy: an Approach for Identifying Bacterial Proteins Exported during Host Infection
Source: mBio. 2017 Apr 25;8(2):e00333-17. doi: 10.1128/mBio.00333-17 (PMC5405230; doi:10.1128/mBio.00333-17)
Supplement: TEXT S1 [file mbo002173284s1.docx]

Supplemental Methods:

*‘BlaTEM reporter plasmid pDW31.* Detailed plasmid information can be found in Supplemental Table 6. pDW31 is a multi-copy mycobacterial vector built from a reduced pMB219 backbone, with a hygromycin resistance cassette derived from pYUB412. pDW31 contains the ‘*blaTEM* reporter (lacking a promoter), which was derived from pJES110 and cloned immediately downstream of a ClaI site. The ClaI site in pDW31 was used for cloning genomic DNA fragments for library construction.

*Isolation of plasmid DNA from recovered M. tuberculosis.* The following modifications were made to the QIAfilter Plasmid Maxi Kit protocol (Qiagen) for isolation of library plasmids from *M. tuberculosis*. 1) Colonies were pooled from agar plates, incubated in roller bottles with broth media 7AGT plus 1% glycine at 37°C for 24 hours, pelleted, and frozen at -20°C. 2) Pellets were resuspended in P1 buffer with RNase and 10 mg/mL lysozyme, and incubated at 37°C for one hour. 3) After filtration through Qiafilter cartridge, lysate was filtered twice through 0.22µm Steriflip units (Millipore) to ensure sterility before removal from the BSL-3 facility. 4) After filtration of lysate through pre-equilibrated Qiatips, the column was washed with a 1:1 mixture of chloroform and methanol prior to additional washes.

*Sample preparation for next-generation (Illumina) sequencing.* Detailed primer information can be found in Supplemental Table 8. Plasmids were linearized by DraI digestion and then sheared by nebulization at 45 psi for 3 minutes in a glycerol TE buffer (53% Glycerol, 37mM Tris-HCl, 5.5mM EDTA), generating fragments ranging from 200-800bp in length. The nebulized DNA was then purified with the QiaQuick PCR Purification kit (Qiagen) and prepared for sequencing as previously described ([1](#_ENREF_1)). Fragments were end repaired using Blunting kit (New England Biolabs E1201L), purified using PCR Purification kit and resuspended in DNase free dH_2_O. Fragments were tailed with dATP (Roche) by Taq polymerase (Invitrogen) for 45 minutes at 72°C. A double stranded adaptor was constructed from Adaptor 1.1 and Adaptor 2.1 primers mixed with MgCl_2_ and incubated in a thermocycler at 1% ramp from 95°C to 20°C. Double stranded adaptors were ligated to the sheared plasmid fragments with T4 DNA ligase (Invitrogen) at 16°C overnight. Using the adaptor-ligated DNA as template, amplification of ‘*blaTEM* fusion junctions was performed with adaptor and ‘*blaTEM* targeting primers (‘Bla IL, ‘Bla IL 3b, ‘Bla IL 4b, ‘Bla IL 5b and Adaptor 1 (containing multiplex barcode 1), Adaptor 2 (containing multiplex barcode 2), and Adaptor 3 (containing multiplex barcode 3)) with Phusion High Fidelity Polymerase (New England Biolabs). 200-400 bp fragments were isolated from the PCR amplification and purified with QiaQuick Gel Purification kit (Qiagen). Samples were sequenced using next-generation sequencing (Illumina HiSeq) generating paired end multiplexed sequencing reads.

*Computational analysis of sequencing data to identify ‘blaTEM reporter fusion sites.* Paired end reads included adapter regions containing two barcodes: a multiplexing barcode, and a random barcode (NNANNANNN), as previously described with minor modifications ([1](#_ENREF_1)). The reads were first de-multiplexed. The adapter regions were identified using custom R code which used the Bioconductor biostrings package ([2](#_ENREF_2)), along with the expected adapter sequence. The random barcodes were then extracted from these identified adapter regions, and the adapter regions were trimmed from each read. Following trimming, the right end read was ~24bp, and the left end read was ~12bp.The trimmed reads were aligned to the H37Rv reference genome. The right end read was aligned by the BWA backtrack algorithm, using the “samse”, command. The left end read was aligned by a custom implementation of smith-waterman to locations within 500 bp of the right end read alignment. Reads in which at least one end failed to align, or in which the only alignment was disconcordant were discarded. Reads were aligned to known AciI or HpaII restriction enzyme sites (the restriction enzymes used to digest gDNA in initial library construction). Reads whose right end start was aligned near to a AciI or HpaII restriction enzyme site (within 4 bp), but not perfectly aligned, were assumed to have been trimmed or aligned incorrectly, and were assigned to the nearby site. Reads whose right end was more than 4bp away from a known AciI or HpaII restriction site were discarded. The random barcodes (NNANNANNN) were employed to control for artificially high counts generated by biased PCR amplification. Sequences with identical fusion positions and the same random barcode were counted as one unique sequence. The gene containing the alignment position of the right end start of each read was identified using the H37Rv RefSeq genome annotation released January 9 2012. For alignments landed in positions contained by more than one annotated gene (positions where the end of an upstream gene in an operon overlaps with the start of a downstream gene), and the gene was assigned to the first gene in numerical order. Unambiguous unique sequences were each counted as a value of 1. Sequences that were ambiguous (could align to more than one location in the genome) were assigned to each of those fusion positions at a value of 1/# of potential alignment positions (e.g. for a sequence that could align to two potential fusion sites, each fusion was assigned a ½ read count). As different sequencing runs/samples yielded different total number of reads (average 1.5x10^7^ reads), all read counts were normalized to the total number of sequenced reads in that sample.

*Statistical analysis used to identify in vivo exported proteins (spleen).* Unique reads for each fusion site in the genome were counted in the output from the spleen of β-lactam treated mice. The most abundant fusion position within each annotated gene was identified individually within the output for each of two replicate experiments. The lower of these two numbers between replicates was used as the abundance value for the gene in the subsequent statistical analysis to increase stringency and require that any identified gene was highly abundant in both samples. Log_10_ values were used to generate a histogram, which was bi-modal. A Gaussian mixture model was used to identify the mean and variance for each population, and determine the probability that each value identified with the higher or lower abundance population (Figure 2A). Genes with a higher probability of belonging to the higher abundance group were identified as *in vivo* exported proteins. Three genes in regions with enriched out of frame fusions were excluded from the results, resulting in 593 genes identified as encoding *in vivo* exported proteins. The abundance levels in the unselected input library were relatively uniform, thus computing enrichment ratios was not required and the statistical analysis was done on the distribution of abundances.

*Statistical analysis used to identify proteins exported significantly more* in vivo *than* in vitro *(*in vivo *induced*). To identify *M. tuberculosis* clones that were β-lactam resistant both *in vivo* and *in vitro,* bacteria recovered from β-lactam treated mice were plated in parallel on agar media containing or lacking β-lactam antibiotics*.* From each *in vitro* plating condition, plasmids were isolated, sequenced, and unique reads for each fusion site counted, as described above. For *in vivo* β-lactam treated samples the lower abundance value between replicates was used as a representative abundance value for the gene, to identify the most stringent list of proteins exported in both experiments, as described above. For dual *in vivo* and *in vitro* β-lactam-treated (recovered on β-lactam containing agar media *in vitro*) samples, the higher abundance value between replicates was used as a representative abundance value for the gene, to identify the most stringent list of proteins that were not exported *in vitro* in either experiment. Pseudo-counts of 100 were added to the *in vivo* and *in vitro* β-lactam treated dataset. The log10 value of the ratio between abundance following *in vivo* treatment and *in vivo* plus *in vitro* treatment was calculated. The top and bottom 5% were trimmed for robustness. This data fit a normal uni-modal distribution, where genes of interest fell on the right shoulder of the curve (with high ratios of *in vivo* vs. *in vivo* and *in vitro* reads). A normal fit distribution was used to identify outliers, with higher ratios than would be predicted by chance. The Benjamini-Hochberg procedure was used to correct for multiple comparisons, and identified genes with p<0.0005 (False Discovery Rate <0.05). Corrected p values (q values) are reported (Table 1). 38 genes were identified that encode for proteins exported significantly more *in vivo* than *in vitro* (Table 1).

*Analysis used to identify* in vivo *exported proteins (lungs).* The lower starting burden in the lungs after intravenous infection resulted in incomplete coverage of the EXIT library in lungs and prevented us from applying statistical modeling to the data as was done with the spleens. Unique reads for each fusion site in the genome were counted in the output from the lungs of β-lactam treated mice, and the most abundant fusion site within each annotated gene was identified separately for each replicate. Genes that displayed 3.5 fold more unique reads in the output than the input (3.5 fold enrichment) in both replicates were used to predict proteins exported *in vivo* in the lungs. 3.5 fold enrichment correlated well with the enrichment level identified for the 593 statistically identified genes encoding proteins exported in the spleen.

*Statistical analysis used to identify individual enriched fusion junctions for topology determination.* The number of sequenced reads for each fusion site in the output from β-lactam treated mice was divided by the number of sequenced reads in the corresponding input for each experiment, with pseudo-counts of 10 added across the board. Values of zero were excluded from analysis. Log10 enrichment values were used to generate histograms, which produced a uni-modal distribution with a right shoulder of enriched sites. A Gaussian mixture model was fitted to the distribution using Mclust in R ([3](#_ENREF_3)). The resulting mixture models had two peaks, one representing the majority of the sites, and a second, smaller peak representing points in the right shoulder. The derived cutoffs for Experiment A corresponded to an enrichment of log10 1.756 fold above input, and Experiment B corresponded to an enrichment of log10 3.203 fold. 2,516 individual fusion sites that were identified as being in the statistically enriched populations in both experiments were considered to be exported.

*Construction of strains expressing HA tagged proteins for* in vitro *export analysis.* Detailed plasmid information can be found on Supplemental Table 6 and primer sequences are provided on Supplemental Table 8. The *rv3707c* gene was PCR amplified using primers *rv3707c*_HA_F2 and *rv3707c*_HA_R1, the *rv1728c* gene with primers *rv1728c*_HA_F1 and *rv1728c*_HA_R1, and the *rv3811* gene with primers *rv3811*_HA_F1 and *rv3811*_HA_R1, and PCR fragments were cloned into pCR2.1 (Invitrogen). The resulting plasmids were sequenced and confirmed error-free. Plasmids containing *rv3707c*, *rv1728c*, and *rv3811* were digested from pCR2.1 with XmnI and HindIII, gel purified, and ligated into MscI and HindIII digested pJSC77 ([4](#_ENREF_4)), containing an in-frame C-terminal HA tag. Plasmids were transformed into *M. tuberculosis* H37Rv as previously described ([5](#_ENREF_5)).

**References**

1. Long JE, DeJesus M, Ward D, Baker RE, Ioerger T, Sassetti CM. 2015. Identifying essential genes in Mycobacterium tuberculosis by global phenotypic profiling. Methods Mol Biol 1279:79-95.

2. H. Pages PA, R. Gentleman, and S. DebRoy Biostrings: String objects representing biological sequences, and matching algorithms.,

3. Fraley C, Raftery AE, Scrucca L, Murphy TB, Fop M. Normal Mixture Modeling for Model-Based Clustering, Classification, and Density Estimation (mclust),

4. Glickman MS, Cox JS, Jacobs WR, Jr. 2000. A novel mycolic acid cyclopropane synthetase is required for cording, persistence, and virulence of *Mycobacterium tuberculosis*. Mol Cell 5:717-27.

5. Braunstein M, Bardarov SS, Jacobs WRJ. 2002. Genetic methods for deciphering virulence determinants of *Mycobacterium tuberculosis*, p 67-99. *In* Clark VL, Bavoil PM (ed), Methods in Enzymology, vol 358. Academic Press, London.
